# The miR-183/96/182 cluster regulates sensory innervation, resident myeloid cells and functions of the cornea through cell type-specific target genes

**DOI:** 10.1038/s41598-024-58403-1

**Published:** 2024-04-01

**Authors:** Naman Gupta, Mallika Somayajulu, Katherine Gurdziel, Giovanni LoGrasso, Haidy Aziz, Rita Rosati, Sharon McClellan, Ahalya Pitchaikannu, Manoranjan Santra, Muhammed Farooq Abdul Shukkur, Paul Stemmer, Linda D. Hazlett, Shunbin Xu

**Affiliations:** 1 Department of Ophthalmology, Visual and Anatomical Sciences, School of Medicine, Wayne State University, 540 E Canfield Street, Detroit, MI 48201 USA; 2 Genome Sciences Core, Wayne State University, Detroit, MI USA; 3 School of Biological Sciences, Wayne State University, Detroit, MI USA; 4 Institute of Environmental Health Sciences, Wayne State University, Detroit, MI USA

**Keywords:** Cell biology, Computational biology and bioinformatics, Immunology, Molecular biology, Neuroscience, Systems biology, Experimental models of disease, Corneal diseases

## Abstract

The conserved miR-183/96/182 cluster (miR-183C) is expressed in both corneal resident myeloid cells (CRMCs) and sensory nerves (CSN) and modulates corneal immune/inflammatory responses. To uncover cell type-specific roles of miR-183C in CRMC and CSN and their contributions to corneal physiology, myeloid-specific miR-183C conditional knockout (MS-CKO), and sensory nerve-specific CKO (SNS-CKO) mice were produced and characterized in comparison to the conventional miR-183C KO. Immunofluorescence and confocal microscopy of flatmount corneas, corneal sensitivity, and tear volume assays were performed in young adult naïve mice; 3′ RNA sequencing (Seq) and proteomics in the trigeminal ganglion (TG), cornea and CRMCs. Our results showed that, similar to conventional KO mice, the numbers of CRMCs were increased in both MS-CKO and SNS-CKO vs age- and sex-matched WT control littermates, suggesting intrinsic and extrinsic regulations of miR-183C on CRMCs. The number of CRMCs was increased in male vs female MS-CKO mice, suggesting sex-dependent regulation of miR-183C on CRMCs. In the miR-183C KO and SNS-CKO, but not the MS-CKO mice, CSN density was decreased in the epithelial layer of the cornea, but not the stromal layer. Functionally, corneal sensitivity and basal tear volume were reduced in the KO and SNS-CKO, but not the MS-CKO mice. Tear volume in males is consistently higher than female WT mice. Bioinformatic analyses of the transcriptomes revealed a series of cell-type specific target genes of miR-183C in TG sensory neurons and CRMCs. Our data elucidate that miR-183C imposes intrinsic and extrinsic regulation on the establishment and function of CSN and CRMCs by cell-specific target genes. miR-183C modulates corneal sensitivity and tear production through its regulation of corneal sensory innervation.

## Introduction

The cornea is an avascular, transparent tissue between the eye and the external environment. It provides visual clarity and two thirds of the refractive power of the eye^1–3^. It also serves as first-line defense against microbial infection and other insults^1–3^. Multiple cell types of different origins, including corneal epithelium, stromal keratocytes, endothelial cells, resident immune cells (CRICs) and nerves^1–4^, work in sync to confer the unique architecture and functionalities of the cornea^1–4^. Although rare (1.2–5% in mouse^5–7^), CRICs are a diverse population and have essential roles in almost every aspect of the development and functions of the cornea under both physiological and pathological conditions^8–16^. The cornea is the most densely sensory-innerved tissue in the body^17–19^. Sensory innervation of the cornea not only provides bodily sensation to various stimuli but also plays important roles in the homeostasis of the cornea as well as the pathogenesis of corneal diseases through neuroimmune interactions^18–33^.

microRNAs (miRNAs) are small, non-coding RNAs and are post-transcriptional regulators of gene expression^34–37^. They play an important role in human diseases^38–45^ and are viable therapeutic targets^46–49^. However, their roles in the neuroimmune interaction in health and diseases of the cornea are still largely unknown. The evolutionarily-conserved, paralogous miRNA cluster, miR-183/96/182 cluster (referred to as the miR-183C from here on) is highly, specifically expressed in and required for the normal development and functions of sensory neurons^50–52^. Inactivation of miR-183C in conventional knockout (KO) mouse models results in multisensory defects^51,53–55^. Point mutations in the seed sequence of miR-96 results in non-syndromic hearing loss in both mouse^56^ and human^57^. In addition, this miRNA cluster is also expressed and plays important roles in both innate^4,7,58–62^ and adaptive immune cells^63–70^.

In the cornea, we demonstrated that inactivation of miR-183C in a conventional KO mouse model results in decreased nerve density and reduced expression of capsaicin receptor TRPV1 and pro-inflammatory neuropeptide substance P (sP) precursor gene Tac1^60^. Inactivation or knockdown of miR-183C in innate immune cells, e.g. macrophages (Mϕ) and neutrophils, reduces their production of pro-inflammatory cytokines, however, enhances their phagocytosis and intracellular bacterial killing capacity^60,61^. These result in a reduced inflammatory response to bacterial infection, e.g. *Pseudomonas aeruginosa* (PA) and contribute to a decreased severity of PA keratitis^60,61,71^. In addition to its effect on peripheral Mϕ and neutrophils, we showed that inactivation of the miR-183C results in an increased number of steady-state CRMCs in naïve mice^4,7^ and myeloid cell infiltration into the cornea in the event of PA infection^7^. Collectively, these findings suggest a pivotal regulatory role of miR-183C in fine-tuning neuroimmune interactions in the cornea.

However, since in the conventional KO mice, miR-183C is simultaneously inactivated in all miR-183C-expressing cells, including both CSN and CRMCs^7,60^, the phenotypes observed reflect a composite effect of loss-of-function of miR-183C in both cell types. This precludes distinguishing whether the changes observed in CSN and CRMCs are intrinsic functions of miR-183C in sensory nerves and myeloid cells, respectively, or an extrinsic effect through neuroimmune interaction. To resolve this conundrum, we produced sensory neuron-specific (SNS) and myeloid cell-specific (MS) conditional knockout mice (CKO). Here we report the first characterization of these CKO mice in comparison to their corresponding wild type (WT) littermate control mice at their steady state. Our results reveal sensory neuron- and myeloid cell-specific functions of miR-183C and their impact on the homeostasis of the cornea.

## Methods

### Mice

All experiments and procedures involving animals and their care were pre-reviewed and approved by the Wayne State University Institutional Animal Care and Use Committee and carried out in accordance with National Institute of Health and Association for Research in Vision and Ophthalmology (ARVO) guidelines (Approved protocol number: IACUC-22-05-4618). The study is reported in accordance with ARRIVE guidelines. Euthanasia was performed by cervical dislocation under anesthesia with isoflurane followed by thoracotomy.

The miR-183C KO—the miR-183C^GT/GT^ mice, are on a 129S2/C57BL/6-mixed background^51^ and were originally derived from a gene-trap (GT) embryonic stem cell clone^51,72,73^. Csf1r-EGFP or MacGreen mice^74^ were purchased from the Jackson Laboratory (Stock number. 018549). In this strain, the EGFP transgene is under the control of the 7.2-kb mouse colony stimulating factor 1 receptor (Csf1r*)* promoter, allowing specific expression of EGFP in the mononuclear phagocyte system (MPS) myeloid cells, including monocytes (MCs), Mϕ and dendritic cells (DCs)^74,75^. The Csf1r-EGFP mice were bred with miR-183C^GT/+^ to produce miR-183C KO [Csf1r-EGFP(+);miR-183C^GT/GT^] and WT mice [Csf1r-EGFP( +);miR-183C^+/+^] on the background of Csf1r-EGFP as described previously^7^.

Mice with miR-183C CKO allele, the miR-183C^f/f^, were provided by Dr. Patrick Ernfors, Karolinska Institutet, Sweden through the European Mouse Mutant Archive (EMMA. ID: EM12387). The miR183C^f^ allele has two loxP sites flanking the 5′ and 3′ ends of the miR-183C for robust Cre-mediated miR-183C CKO^76^. The myeloid-specific, LysM-Cre mice^77^ were purchased from The Jackson Laboratory (Stock number: 004781). The LysM-Cre knock-in/knock-out allele has a nuclear-localized (NLS)-Cre recombinase inserted into the first coding ATG of the lysozyme 2 gene (*Lyz2*), both abolishing endogenous *Lyz2* gene function and placing NLS-Cre expression under the control of the endogenous *Lyz2* promoter/enhancer elements. Therefore, the LysM-Cre allows myeloid-specific expression of Cre recombinase. The sensory nerve specific Na_v_1.8-Cre mice^78^ were kindly provided by Dr. John N. Wood, University College London, through Dr. Theodore J. Price, University of Texas Dallas. This strain is now available at the Jackson Laboratory (Stock Number: 036564). The voltage-gated sodium channel Na_v_1.8 (encoded by the Scn10a gene) is one of the signature genes of the majority of nociceptive sensory neurons in the TG and dorsal root ganglia (DRG)^33,79–81^. Na_v_1.8 promoter-driven Cre recombinase (Na_v_1.8-Cre) is expressed in nearly all corneal nociceptive sensory nerves^33,78,82^. The reporter strain, R26^LSL-RFP(+/+)^ mice^83^, also known as Ai14 (Stock number: 007914, the Jackson Laboratory) has a loxP-flanked STOP cassette (LSL) in front of a tdTomato RFP cassette, all of which are inserted into the ROSA26 locus^83^. The LSL prevents the transcription of tdTomato RFP; however, when Cre recombinase is present, the LSL cassette will be excised to allow the expression of tdTomato RFP.

miR-183C MS-CKO mice [LysM-Cre(+/−);miR-183C^f/f^;Csf1r-EGFP(+/+)], in which MPS myeloid cells are labeled with EGFP, were produced by breeding of the LysM-Cre(+/−), miR-183C^f/f^ and Csf1r-EGF(+/−) mice. Their littermates [LysM-Cre(−/−);miR-183C^f/f^;Csf1r-EGFP(+/+)] are used as WT controls. SNS-CKO mice [Na_v_1.8-Cre(+/−);miR-183C^f/f^;R26^LSL-RFP(+/+)^;Csf1r-EGFP(+/+)] and their WT littermates [Na_v_1.8-Cre(+/−);miR-183C^+/+^;R26^LSL-RFP(+/+)^;Csf1r-EGFP(+/+)] were produced by breeding of Na_v_1.8-Cre (+/−), miR-183C^f/f^, R26^LSL-RFP^ and Csf1r-EGF(+/−) mice. All breeding showed a normal mendelian inheritance pattern.

Sex is considered as a biological variance in all studies. 8–12 weeks old, male and female mice were used as separate groups in all experiments. The age, sex and number of the mice in each experiment are specified in the figure legends and/or the text.

### Hematoxylin and eosin (H&E) staining of paraffin sections of mouse cornea

Mouse eyes were enucleated and fixed in 4% formalin. The embedding, sectioning and H&E staining were performed by Excalibur Pathology Inc (Oklahoma City, OK). Comparable sagittal sections through the optic nerves were imaged under a DM4000b brightfield microscope (Leica).

### Fluorescence-activated cell sorting (FACS)

8–12 weeks old, naïve MS-CKO and KO mice and age- and sex matched WT littermate controls were used to isolate Csf1r-EGFP + myeloid cells from the cornea and spleen for DNA and RNA preparations (see below). For corneal samples, corneas anterior to the limbi from 6–7 mice/genotype were carefully dissected and pooled for single cell preparation as described before^4,7^. For spleen cells, mononuclear cells were isolated following a standard protocol^84^. FACS was conducted on a Sony SY3200 cell sorter at the Microscopy, Imaging and Cytometry Resources Core (MICR), Wayne State University (WSU) to isolate Csf1r-EGFP + myeloid cells as we described previously^4,7^. EGFP- corneal and spleen mononuclear cells were used as negative controls to optimize the gating.

### DNA and genotyping PCR

Genomic DNA was prepared from mouse tail, cornea, TG, FACS-sorted Csf1-EGFP + myeloid cells using the gMax DNA mini kit (IBI Scientific) following manufacturer’s instruction as described previously^85^. Subsequently, DNA concentration and quality were assayed on the Nanodrop 2000 (ThermoFisher Scientific). 100 ng (from mouse cornea, TG and tails) or 4 ng (from FACS-sorted myeloid cells of the cornea and spleen) genomic DNA was used for genotyping PCR to detect tissue and/or cell type-specific recombination at genomic DNA level as we described previously^51,85^. PCR of 18 s rRNA was amplified and used as a loading control. The genomic structure and primers are illustrated in Fig. S1. The sequences of the primers are: 7Fintron1: 5′-TACCCTGAGTGTGTCTCAATC-3′; CKO-R: 5′-GCAGAGTCACAAACATGTGTAGC-3′; 18 s rRNA Forward: 5′-GTAACCCGTTGAACCCCATT-3′; 18 s rRNA Reverse: 5′-CCATCCAATCGGTAGTAG CG-3′.

### RNA preparation and qRT-PCR

Total RNA was prepared using the miRVana miRNA isolation kit (Life Technologies, Foster City, CA, USA) or the RNeasy (Qiagen, Frederick, MD, USA) for miRNA or mRNA studies, respectively, as described previously^50,86,87^. Quantitative (q)RT-PCR for miRNAs was performed using Taqman miRNA primers and RT-PCR kit (Life Technologies) with snRNA U6 as an endogenous control as described before^50,87^. For protein-coding genes, qRT-PCR was performed using a QuantiFast SYBR Green RT-PCR kit and QuantiTect primers (Qiagen) with 18 s rRNA as endogenous controls^50,51,87^.

### Tear volume measurement

Tear volume was measured by the Zone-Quick Phenol Red Thread (PRT) test (Yokota Co. Ltd). Manufacturer’s instruction was followed with modifications. Briefly, under light anesthesia by isoflurane, the lower eyelid was pulled down slightly; the PRT is placed between the palpebral conjunctiva of the lower eyelid and the eyeball at a point approximately 1/3 of the distance from the lateral canthus for 15 s. The length of the thread which turned red was measured.

### Corneal sensitivity test

Corneal sensitivity to mechanical stimuli was measured by a blink threshold test using a Cochet and Bonnet aesthesiometer (Western Ophthalmics). Briefly, the tip of a nylon filament, starting from 6 cm, was applied perpendicularly to the central cornea. The length at which the mouse blinks was registered as the blink threshold.

### Corneal flatmount, immunofluorescence (IF), confocal microscopy, and quantification of Csf1r-EGFP + corneal resident MPS cells and corneal nerve density

Corneal flatmount and IF was performed as described previously^7,71^. Briefly, mice were euthanized; eyes were enucleated and transferred to cold phosphate buffered saline (PBS). Under a dissecting scope (VWR International, Radnor, PA), the cornea anterior to the limbus was carefully dissected out. The corneas were transferred to cold 1% paraformaldehyde (PFA) in 0.1 M phosphate buffer (PB), pH 7.4 for 1 hour (h) at 4 °C. For direct confocal microscopy, the cornea was flattened by six evenly spaced cuts from the periphery toward the center and mounted in Vectashield media with DAPI (Vector Laboratories, Burlingame, CA) on Superfrost Plus slides (Fisherbrand). For IF, after fixation, the corneas were incubated in a blocking buffer with 2% normal goat serum (NGS) (Vector labs) in PBS for 30 min at room temperature (RT); then, permeabilized with 0.1% Triton X-100 in the blocking buffer for 30 min at RT. Subsequently, corneal tissues were incubated with mouse anti-*β*-Tubulin III (1/600 dilution. Cat. No. 801201, BioLegend, San Diego, CA, USA) antibodies in the blocking buffer for 72 h at 4 °C. After washes with PBS, the corneas were incubated with AF546–conjugated goat anti-mouse IgG (1/1000 dilution. Cat. No. A-11003, Thermo Fisher Scientific) for overnight at 4 °C. After washing, the corneas were flattened and mounted on slides. Negative controls were treated similarly with omission of the primary antibody. All slides were imaged using a TCS SP8 laser confocal microscope (Leica Microsystems Inc. Buffalo Grove, IL). To capture the images of the entire cornea, a series of Z-stacked images under 10 × objective were taken across the entire cornea and stitched together. These stitched Z-stacked images were merged/flattened for cell counting or nerve density quantification using Adobe Photoshop CS6 (64 bit) and ImageJ 1.52p software (http://imagej.nih.gov/ij. NIH, Bethesda, MD, USA) as described previously^7^. Briefly, the raw image was first converted to the 16-bit grayscale; then the black and white binary image is optimized for the threshold to faithfully represent the original image and the cell density. The EGFP + cells in the entire cornea were counted. To quantify nerve density, the mean value of pixels in a 500-μm^2^ square covering the center of the whorl-like subbasal plexus was quantified as the nerve density of the center; the ones in three 500-μm^2^ squares randomly placed in the periphery of the cornea were measured, the average of which was recorded as nerve density of the peripheral cornea. Corneas from at least n = 3 mice/sex/genotype were quantified.

### 3′ RNA sequencing and data analysis

mRNAs were isolated from TG, corneas or FACS-sorted Csf1r-EGFP + myeloid cells of young adult (8–12 weeks old) male mice. For miR-183C KO strain, n = 3 for both KO and WT controls; for SNS-CKO strain, n = 3 for SNS-CKO mice, n = 4 for WT controls; for MS-CKO strain, n = 5 for both MS-CKO and WT controls were used. To isolate Csf1r-EGFP + myeloid cells, 12 corneas of 6 KO and 14 corneas of 7 WT control mice or 20 corneas of 10 MS-CKO or 10 WT control mice were pooled for FACS isolation. Subsequently, 3′ mRNA-Seq libraries were prepared by the Genome Sciences Core (GSC), WSU using the QuantSeq 3′ mRNA-Seq Library Prep kit FWD (Lexogen, Greenland, NH)^88^. High sensitivity D1000 ScreenTape peak range between 100 and 700 bp were in accordance with Lexogen guidelines. The libraries were sequenced on NovaSeq sequencer (Illumina). Reads were aligned to the mouse genome (Build mm10) using a public available software *STAR*^89^ and tabulated for each gene region using *HTSeq*^90^. Differential expressed genes (DEGs) between KO or CKO vs WT controls were identified by *edgeR*^91^. miR-183C predicted target genes were identified by TargetScan algorithm (Targetscan.org)^92–96^ in the upregulated genes in CKO or KO vs WT control and are recognized as miR-183C target genes in these tissues. Statistical significance of enrichment of miR-183C targets in upregulated genes was analyzed by Chi-square with Yates’ correction using the Analyze a 2 × 2 Contingency Table (GraphPad. https://www.graphpad.com/quickcalcs/contingency1/). Functional annotation analysis was performed using the Database for Annotation, Visualization and Integrated Discovery (DAVID)^97^ as described before^51^.

### ELISA assay of CX3CL1 in the TG and cornea

Mouse CX3CL1/Fractalkine Quantikine ELISA kit (Cat. No. MCX310. R&D Systems) was used for analysis of protein levels of CX3CL1 in the TG and the cornea of miR-183C KO mice. Sensitivity of this kit is 0.08–0.32 ng/ml. 12 weeks old, male, naïve KO and age- and sex-matched WT littermate control mice were used for this assay. n = 5/genotype. ELISA assay was performed following the manufacturer’s instruction as described before^60,71^.

### Proteomics in TG and corneas

TG and corneas of adult, male miR-183C KO mice and age- and sex-matched WT littermate controls mice (n = 3/genotype) were harvested, snap-frozen in liquid nitrogen and subjected to proteomics in the WSU Proteomics Core as described before^98^. Briefly, tissue samples were homogenized in 100 ul of 2% Lithium dodecyl sulfate, 40 mM triethylammonium bicarbonate buffer (TEAB, Honeywell Fluka Cat. No. 60-044-974). Then, the samples were reduced and alkylated by incubating with 5 mM DL-Dithiothretol (DTT, Sigma Cat. No. D5545) for 1 h at 37 °C followed by addition of 15 mM Iodoacetamide (IAA, Sigma Cat. No. I1149) for 30 min (min) at RT in the dark. After IAA quenching with 5 mM DTT, samples were acidified by addition of 2 μl of 12% phosphoric acid. Then, proteins were precipitated by adding 700 μl 90% MeOH, 100 mM TEAB and incubating for 1 h at 37 °C and overnight at − 20 °C. Precipitates were pelleted by centrifugation for 1 min at 16,000×*g*, washed with 90% MeOH, 10 mM TEAB, and dried on the bench before resuspended in 25 μl of 50 mM HEPES (pH 8.2). Then, 0.25 ug of Trypsin (Promega, Cat. No. V5113) was added to each sample and incubated at 47 °C for 1 h and at 37 °C, overnight to complete the digestion. Subsequently, samples were labeled with Tandem Mass Tag 6plex (TMT-6plex) reagents by adding the selected reagent to each sample and 2-h incubation at RT. 140 µg of each TMT-6plex reagent (ThermoFisher, USA) was used for each 20 μg sample. All samples were then pooled and speed-vac’ed to dryness. Fractionation was achieved using high pH reversed phase spin cartridges (Pierce, Cat. No. 84868). A step gradient of increasing ACN concentrations was applied to elute bound peptides in nine fractions. Each fraction was dried in a vacuum centrifuge and stored until analysis by mass spectrometry. The mass spectrometry analysis was performed using a Vanquish-Neo chromatography system with an Acclaim PepMap 100 trap column (100 µm × 2 cm, C18, 5 µm, 100 Å), and an Easy-Spray PepMap RSLC C18 75 μm × 25 cm column (Thermo scientific). LC–MS/MS was performed on an Orbitrap Eclipse MS system operated with Real Time Search (RTS) active. Subsequently, mass spectrometry data were processed by Proteome Discoverer version 2.4 using the Sequest HT algorithm with Percolator and the mus musculus Uniprot FASTA database (downloaded March 8, 2021, 17,035 entries). False discovery rate (FDR) was calculated by enabling the peptide sequence analysis using a decoy database, and a cut-off of 1% was used for identifications. Quantitative values were normalized using the total Master Protein signal in each TMT channel.

### Statistical analysis

When the comparison was made among more than 2 conditions, one-way ANOVA with Bonferroni’s multiple comparison test was employed (GraphPad Prism)*;* adjusted p < 0.05 was considered significant. Otherwise, a two-tailed Student’s t test was used to determine the significance; p < 0.05 was considered significant. Each experiment was repeated at least once to ensure reproducibility and data from a representative experiment are shown. Quantitative data is expressed as the mean ± SEM.

## Results

### Sensory nerve- and myeloid cell-specific knockout of miR-183C results in no major morphological changes of the cornea

**Figure 1:**
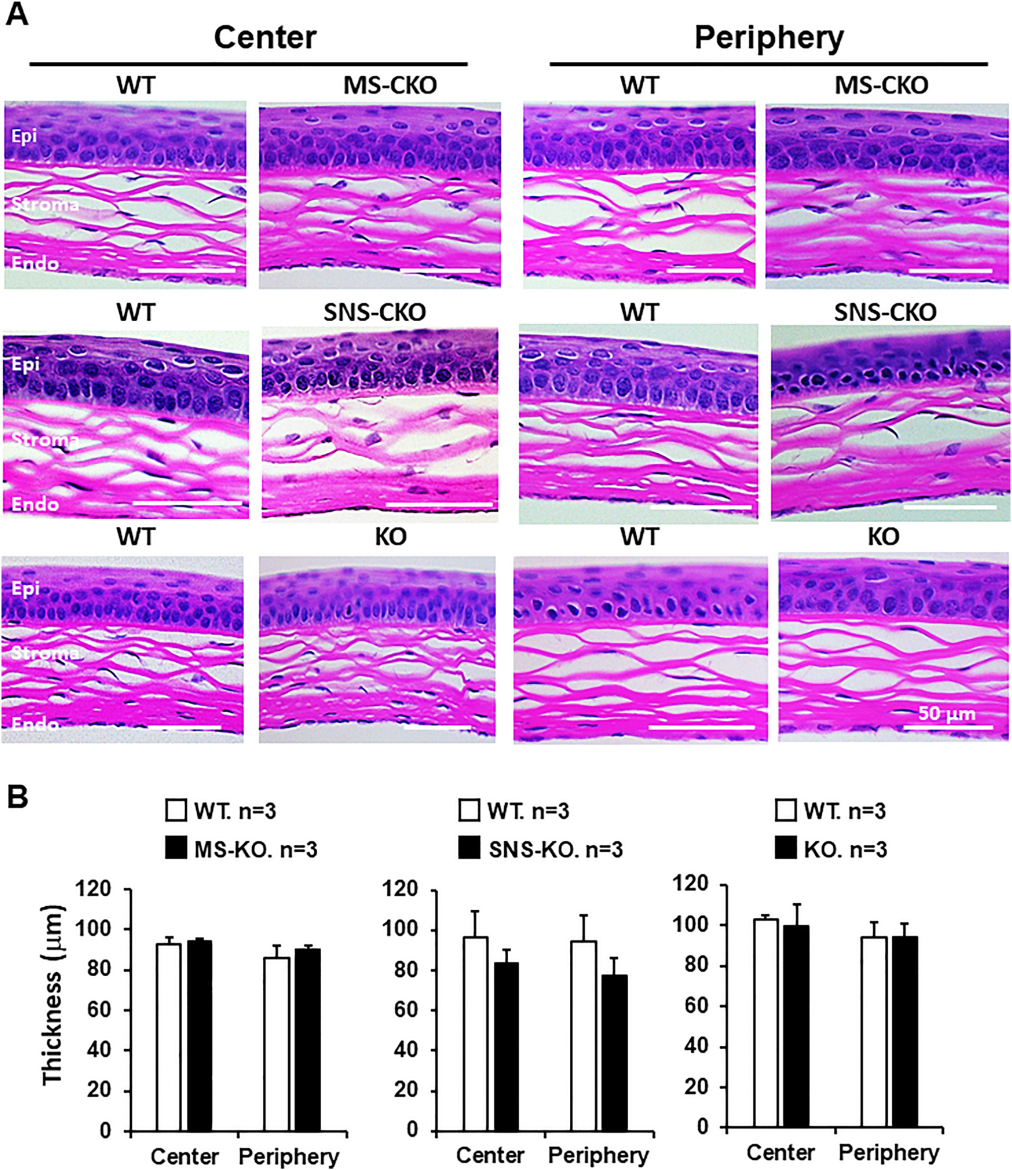
Inactivation of miR-183C has no significant impact on the gross histological architecture of the cornea. (**A**) H&E staining of cross-sections of the corneas of MS-CKO, SNS-CKO and miR-183C conventional KO and age- and sex-matched WT control littermates. (**B**) Measurement of the thickness of the corneas. *Epi* epithelium, *Endo* endothelium.

To uncover tissue-specific functions of the miR-183C, we produced sensory neuron- and myeloid cell-specific miR-183C CKO mice (Fig. S1). To detect whether inactivation of miR-183C in sensory nerves and/or myeloid cells causes major histological and morphological changes in the cornea, we performed H&E staining in cross-sections of the corneas of SNS-CKO, MS-CKO and KO mice and their WT control littermates. No gross morphological changes were observed in the cornea of miR-183C SNS-CKO, MS-CKO and conventional KO mice, when compared to their WT controls (Fig. 1). The total thickness (Fig. 1) and thickness of the epithelial and stromal layers (not shown) had no significant differences between the CKOs and KO vs their age- and sex-matched WT controls.

### Inactivation of miR-183C in either sensory neurons or myeloid cells results in increased number of CRMCs

**Figure 2:**
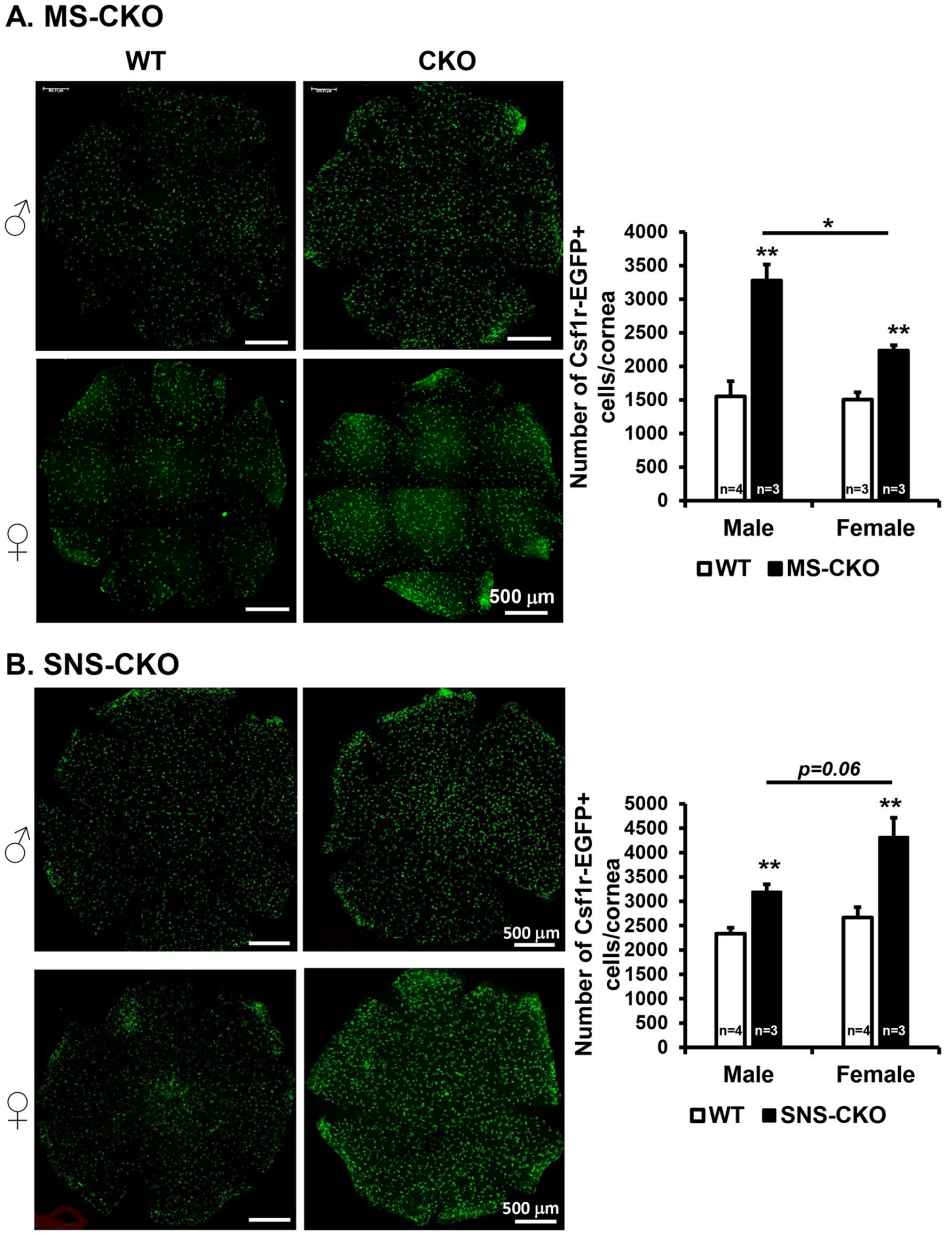
Inactivation of miR-183C in either sensory neurons or myeloid cells results in increased number of corneal resident myeloid cells (CRMCs). Compressed confocal images of flatmount cornea of young adult MS-CKO (**A**) and SNS-CKO sand their age- and sex-matched WT control littermates (**B**). **p < 0.01.

Quantification of Csf1r-GFP + CRMCs of young adult, naïve mice showed that the numbers of Csf1r-EGFP + resident myeloid cells in MS-miR-183C CKO mice [3278 ± 268/cornea (n = 3) in male; 2234 ± 80/cornea (n = 3) in female] were significantly increased in both male and female mice, when compared to age-matched WT controls [1697 ± 225/cornea (n = 3) in male, 1507 ± 107/cornea (n = 3) in female] (Fig. 2A), suggesting miR-183C intrinsically regulates the number of CRMCs under homeostatic condition. In WT controls, male and female mice showed no difference in the number of CRMCs, however, in MS-CKO, the male vs female mice showed increased number of CRMCs (Fig. 2A), suggesting a potential sex-related modulation.

To test whether miR-183C in CSN has an extrinsic regulation on the CRMC population, we quantified the number of CRMCs in the SNS-CKO mice. Intriguingly, sensory nerve-specific inactivation of miR-183C also resulted in an increased number of CRMCs when compared to age- and sex-matched WT controls (Fig. 2B). In male, it was increased by ~ 35% [3197 ± 151/cornea in SNS-CKO (n = 3) vs 2335 ± 121/cornea in WT controls (n = 4)]; while in the female by ~ 56% in female [3197 ± 151/cornea in SNS-CKO (n = 3) vs 2772 ± 298/cornea in WT controls (n = 4)] (Fig. 2B). The numbers of CRMCs showed no significant differences between male and female in WT, however, an increased trend (*p* = 0.06) in the female vs male in the SNS-CKO mice.

### Inactivation of miR-183C in sensory neurons, but not in CRMCs, results in decreased sensory nerve density in the epithelial layer

**Figure 3:**
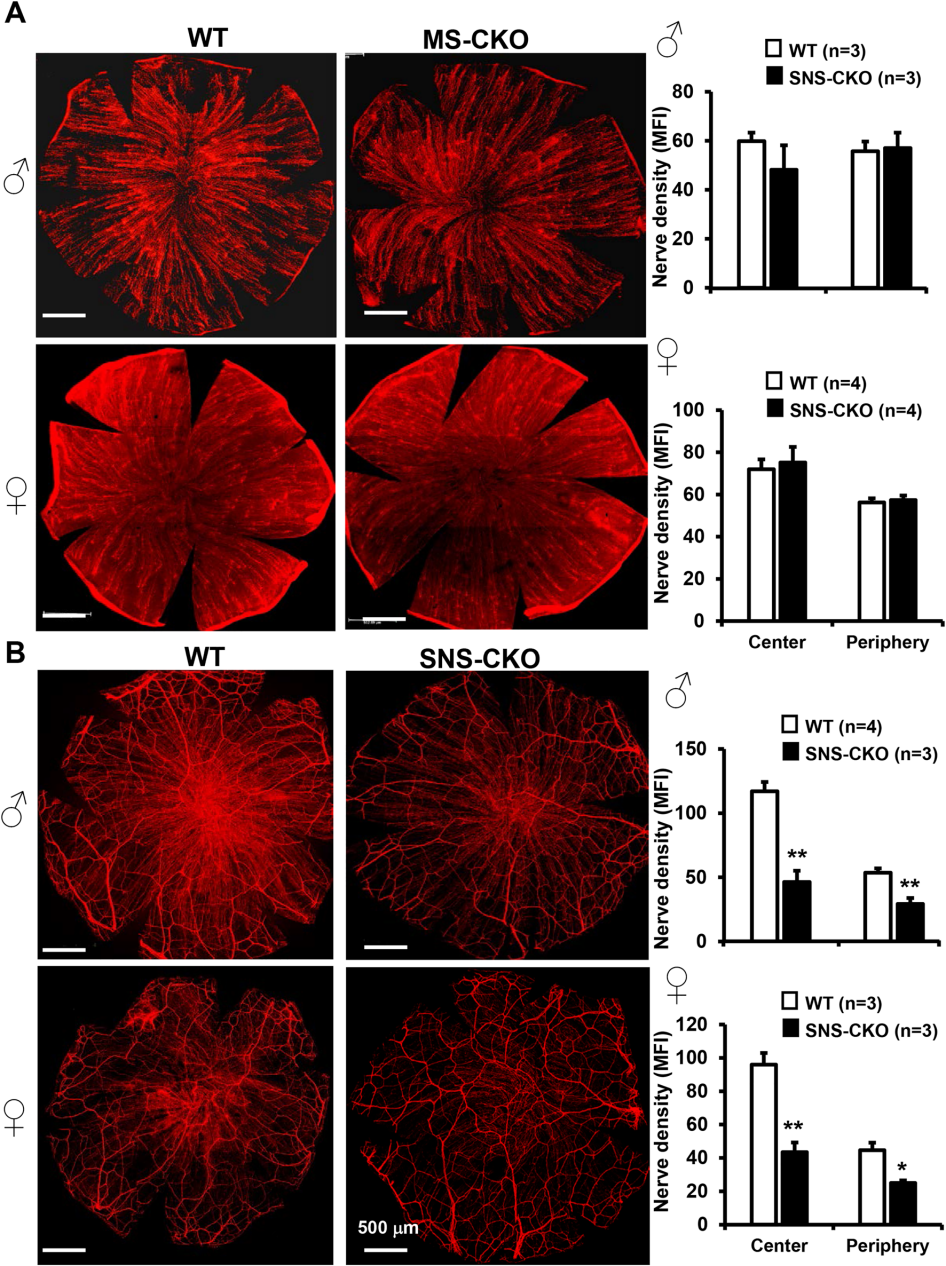
Inactivation of miR-183C in sensory neurons, but not in the CRMCs, results in decreased sensory nerve density. Compressed confocal images of flatmount corneas of young adult MS-CKO (**A**) and SNS-CKO sand their age- and sex-matched WT control littermates (**B**). *p < 0.05; **p < 0.01. *MFI* mean fluorescence intensity.

Previously, we showed that corneal nerve density is significantly decreased in the miR-183C KO mice^60^. To dissect the contribution of miR-183C in corneal sensory nerves or resident myeloid cells to this phenotype, we quantified corneal nerve density in the SNS-CKO and MS-CKO mice. β-III tubulin IF of flatmount cornea showed no difference in corneal nerve density of the MS-CKO vs their WT littermate control mice in both male and female (Fig. 3A), suggesting loss of miR-183C in CRMCs had no contribution to the decreased corneal nerve density in the miR-183C KO mice^60^.

However, the corneal nerve density was significantly decreased in the SNS-CKO vs WT littermate control mice in both male and female (Fig. 3B), in both the whorl center and peripheral regions of the cornea, suggesting that decreased nerve density in the miR-183C conventional KO mice^60^ is a result of loss of miR-183C in corneal sensory nerves; miR-183C imposes an intrinsic regulation of corneal sensory innervation.

**Figure 4:**
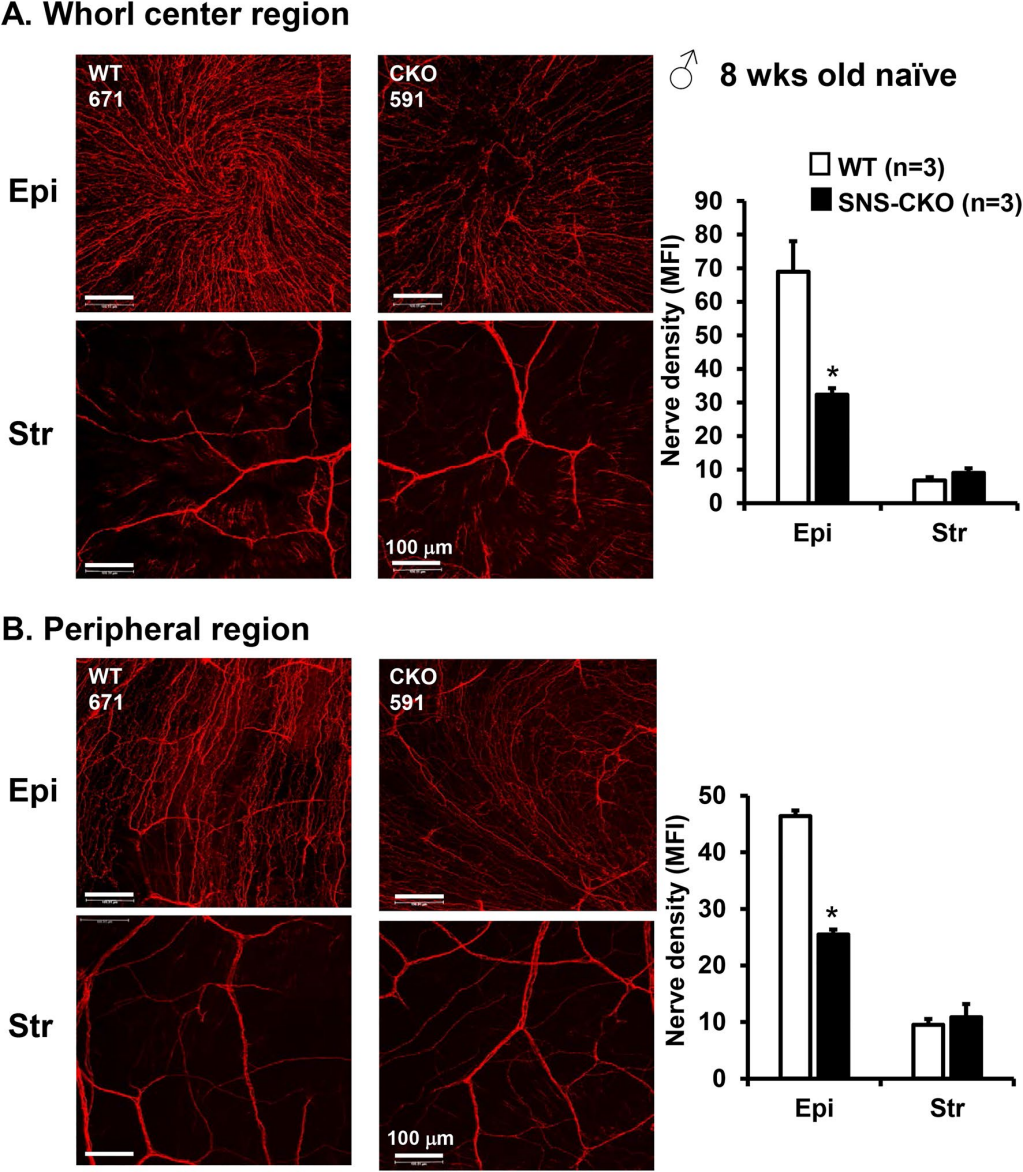
Reduction of corneal sensory nerve density in the SNS-CKO is caused by decreased sensory nerve density in the epithelial layer but not the stromal layer. Compressed confocal images of corneal sensory nerves of the epithelial layers (Epi) and stromal layers (Str) of the whorl center areas (**A**) and peripheral regions (**B**) of flatmount corneas of young adult, male SNS-CKO sand their age- and sex-matched WT control littermates. *p < 0.05.

To further probe into the reduction of sensory nerve density, we quantified the sensory nerve density in the epithelial and stromal layers separately (Fig. 4). The layer-specific analyses showed that, in both the whorl center and peripheral regions of male mice, it is the density of the fine terminal nerves in the epithelial layer that is significantly decreased, but not the large nerves in the stromal layer (Fig. 4), suggesting that miR-183C regulates the terminal differentiation and/or extension of sensory nerves in the epithelial layer of the cornea, but has no significant impact on the establishment of large sensory nerves in the stromal layer. Similar effect was observed in female mice (Fig. S2).

### Inactivation of miR-183C in sensory nerves results in decreased corneal sensitivity to mechanical stimuli

**Figure 5:**
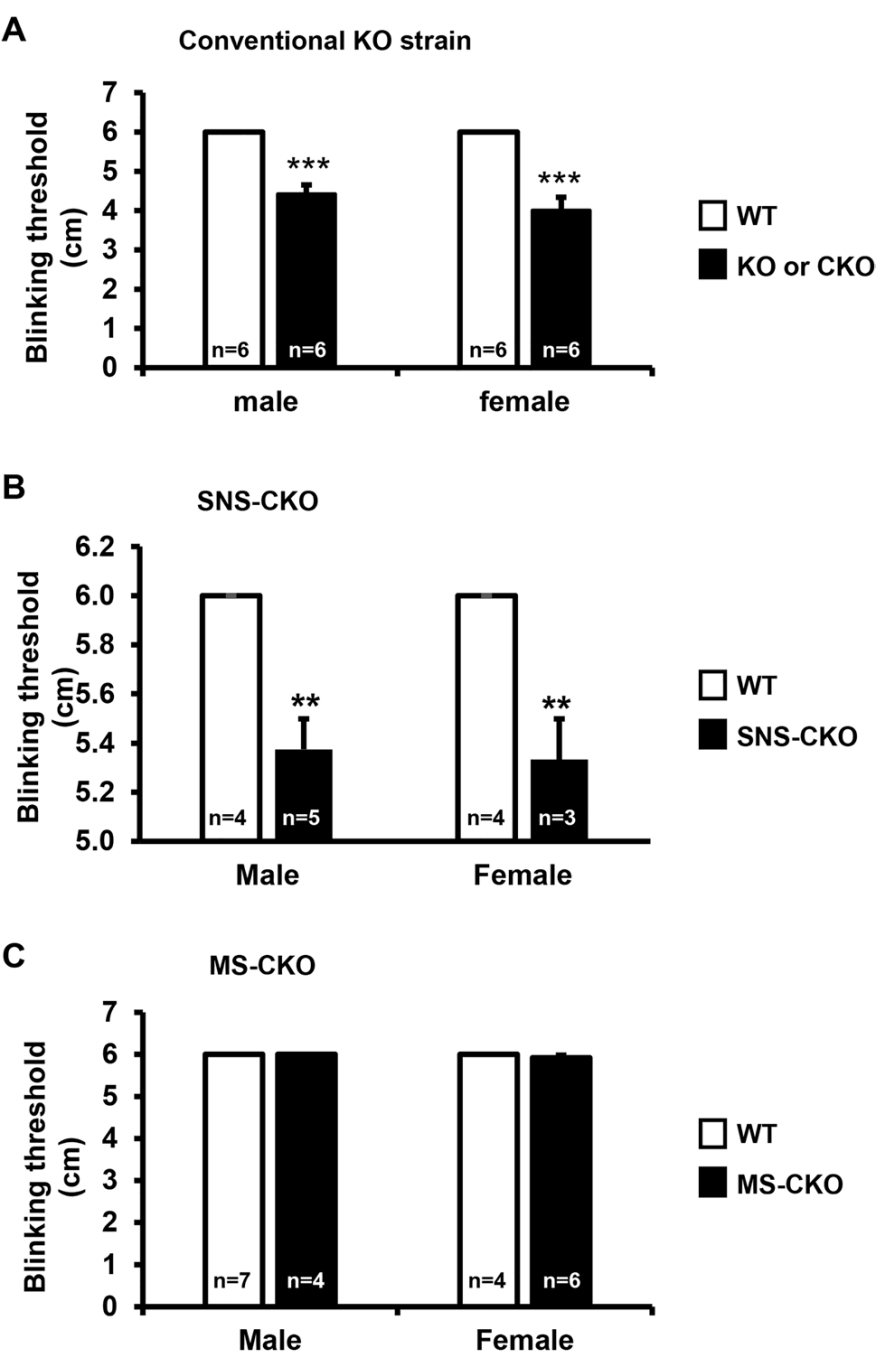
Inactivation of miR-183C in sensory nerves, but not in the myeloid cells, results in decreased corneal sensitivity to mechanical stimuli. Sensitivity test using a Cochet and Bonnet aesthesiometer in young adult (8–12 weeks old) miR-183C conventional KO (**A**), SNS-CKO (**B**) and MS-CKO mice and their age- and sex-matched WT control littermates (**C**). **p < 0.01; ***p < 0.001.

Since inactivation of miR-183C in sensory neurons resulted in decreased sensory nerve density (Figs. 3, 4), we hypothesize that the functions of the sensory nerve are compromised in the cornea. To test this hypothesis, we first tested corneal sensitivity to mechanical stimuli using a Cochet and Bonnet aesthesiometer. Our result showed in both miR-183C KO (Fig. 5A) and SNS-CKO mice (Fig. 5B), corneal sensitivity was significantly decreased, when compared to their WT littermate. In the conventional KO, corneal sensitivity was decreased by ~ 23% and 33% in male and female mice, respectively. In the SNS-CKO, corneal sensitivity was significantly reduced by ~ 10 and 11% in male and female mice, respectively. However, in MS-CKO mice, inactivation of miR-183C showed no impact on corneal sensitivity. These data suggest that miR-183C in corneal sensory nerves intrinsically regulate its function. Decreased corneal sensitivity in miR-183C KO is a result of loss of miR-183C in the sensory nerve.

### Inactivation of miR-183C in sensory nerves results in decreased basal tear production

**Figure 6:**
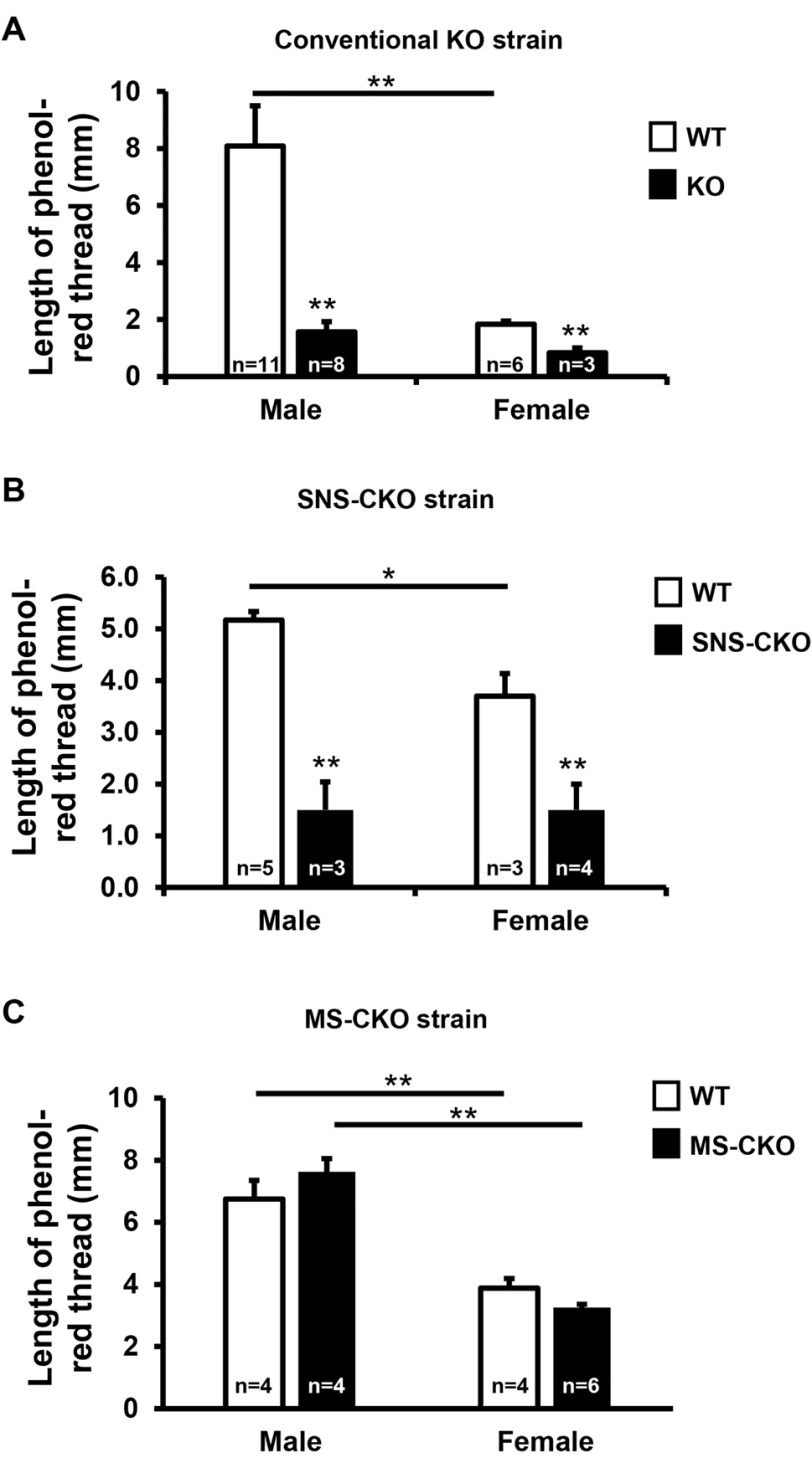
Inactivation of miR-183C in sensory nerves, but not in myeloid-cells, results in decreased basal tear volume. Phenol-red thread assays in young adult (8–12 weeks old) miR-183C conventional KO (**A**), SNS-CKO (**B**) and MS-CKO mice and their age- and sex-matched WT control littermates (**C**). *p < 0.05; **p < 0.01.

It is known that corneal sensory innervation plays important role in regulating tear production^27,99^. Since inactivation of miR-183C in sensory neurons resulted in decreased sensory nerve density and sensitivity to mechanical stimuli (Figs. 3, 4, 5), we further hypothesize that miR-183C may play a role in tear production through its regulation of corneal sensory innervation. To test this hypothesis, we measured tear volumes using the PRT test. Our result showed that in both the miR-183C KO and SNS-CKO mice, tear volume was significantly decreased when compared to their WT littermates (Fig. 6A,B). In male mice, the tear volume was decreased by ~ 81% in the miR-183C KO and ~ 71% in the SNS-CKO mice; while in female, it was decreased by ~ 55% and 59% in the conventional KO and SNS-CKO, respectively (Fig. 6A,B). However, tear volume of MS-CKO mice showed no difference compared to their WT littermate controls (Fig. 6C). These data suggest that miR-183C modulates basal tear production through regulation of corneal sensory innervation.

In addition, our data showed that the tear volume in female vs male WT mice was consistently reduced in all three strains, by ~ 77%, 28%, and 43% in the conventional KO, SNS-CKO, and MS-CKO mice, respectively, suggesting a sex-dependent difference of tear production (Fig. 6A–C).

### miR-183C modulates the functions of sensory neurons and myeloid cells through its regulation of cell type-specific target genes

To start to understand the molecular mechanisms underlying miR-183C’s regulation of corneal functions, we performed 3′ mRNA sequencing^88,100^ in the TG and corneas of young adult, naïve SNS-CKO, MS-CKO and miR-183C KO mice and their corresponding WT littermate controls. Comparison of the transcriptomes identified a series of DEGs in the TG and corneas of SNS-CKO, MS-CKO and miR-183C KO mice vs their WT controls (Fig. S3; Tables S1–S6). Since miRNAs negatively regulate gene expression^35^, to identify tissue-specific targets of miR-183C, we searched predicted target genes of miR-183C among the upregulated genes in KO or CKO vs their WT controls in both TG and cornea. This analysis yielded a series of tissue-specific target genes of miR-183C (Tables S7–S12). Statistical analysis showed that miR-183C predicted target genes are significantly enriched among the upregulated genes in the TG and cornea of SNS-CKO or KO mice vs their corresponding WT littermate controls. However, no enrichment of miR-183C predicted target genes was detected in the upregulated genes of either TG or cornea of miR-183C MS-CKO mice, possibly a result of the rarity of the CRMCs in the TG or cornea^4,7^.

**Figure 7:**
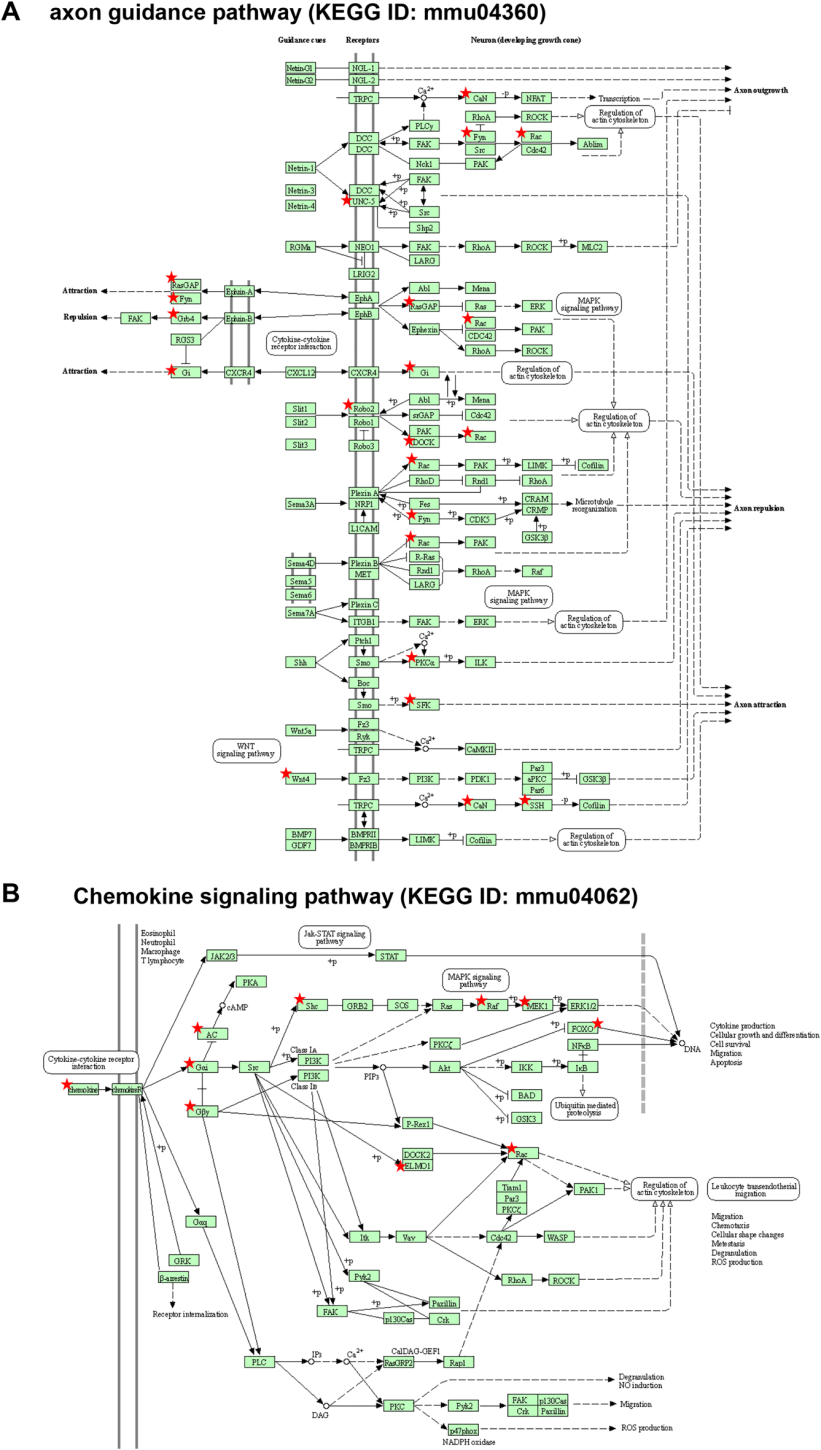
miR-183C target genes in TG sensory neurons are enriched in axon guidance (**A**) chemokine signaling pathways (**B**). Modified from www.genome.jp/kegg/pathway.html. Star-labeled molecules or complexes are or contain target genes of miR-183C.

Functional annotation analyses of the tissue-specific target genes identified in the TG and cornea of SNS-CKO and KO mice showed similar overlapping results (Tables S13–S20). Since miR-183C is predominantly expressed in the TG sensory neurons when compared to myeloid cells (> 50 folds; Fig. S4) and that CRMCs are a rare population in the cornea (1–3% of total corneal cells)^4,7^, we reasoned that gene expression changes and miR-183C targets identified in the TG and cornea of SNS-CKO and KO mice represents TG sensory neuron-specific changes and miR-183C targets. Gene ontology (GO) analysis of the tissue-specific target genes identified in the TG of SNS-CKO and KO mice (Tables S13, S17, S21) showed significant enrichment in neuronal axon projection-, immune response- and epithelial cell proliferation and migration-related biological processes (Table 1). Consistent with the GO analysis, Kyoto Encyclopedia of Genes and Genomes (KEGG) pathway analysis showed enrichment in axon guidance (Fig. 7A), synaptic function pathways (Table 2; Tables S14, S18, S22). In the cornea, miR-183C target genes identified in the SNS-CKO and miR-183C KO mice (Tables S15, S16, S19, S20, S23, S24) are prominently enriched in regulation of synaptic functions, in addition to axonogenesis and axon projection (Tables 3 and 4). These data collectively suggest that miR-183C regulates sensory neuronal projection and synaptic function through directly targeting genes involved in these biological processes. Besides neuronal related functions, miR-183C targets in TG sensory neurons are also enriched in epithelial cell proliferation and migration-, cell–cell adhesion-, fibroblast and endothelial cell migration-related biological processes, suggesting indirect regulation of miR-183C on other cell types in the cornea through its modulation of corneal sensory innervation (Tables 1, 2, 3, 4). Furthermore, genes involved in chemokine signaling pathway, e.g. CX3CL1, GNG5, GNG12, GNAI3, ADCY6, SHC1, BRAF, ELMO1, RAC1, MAP2K1, FOXO3 (Fig. 7B), leukocyte trans-endothelial migration, e.g. GNAI3, MSN, PRKCA, RAC1, and FcγR-mediated phagocytosis pathways, e.g. MAP2K1, PRKCE, PRKCA, RAC1, are also enriched in miR-183C target genes of TG sensory neurons (Tables 2, 4), suggesting potential roles in neuropeptide production and neuroimmune interactions.

**Table 1:** Neuronal, immune and epithelial-related GO terms enriched in miR-183C targets in TG sensory neurons.

GO ID	Terms	P	Genes	Fold enrichment
Neuronal related				
0010976	Positive regulation of neuron projection development	0.0023	EHD1, RGS2, ANKRD27, ITGA3, STMN2, FYN, CX3CL1	5.17
1902667	Regulation of axon guidance	0.0428	NOVA2, MYCBP2	45.59
0031175	Neuron projection development	0.0431	EHD1, GDNF, SHC1, STMN2, FYN	3.78
0007411	Axon guidance	0.0508	KLF7, GAP43, MYCBP2, PAX6, ANK3	3.58
Immune related				
0050729	Positive regulation of inflammatory response	0.0355	CEBPA, CD47, LDLR, CX3CL1	5.53
0071560	Cellular response to transforming growth factor beta stimulus	0.0003	ZEB1, NOX4, FYN, WNT2, TGFBR1, WNT4	10.39
0036120	Cellular response to platelet-derived growth factor stimulus	0.0152	PRKCE, RASA1, FYN	15.78
Epithelial related				
0010634	Positive regulation of epithelial cell migration	0.0373	ITGA3, PRKCE, PPM1F	9.77
0050680	Negative regulation of epithelial cell proliferation	0.0279	ZEB1, CELF1, PAX6, MTSS1	6.08

**Table 2:** Neuronal and immune-related KEGG pathways enriched in miR-183C targets in TG sensory neurons.

KEGG ID	Term	P	Genes	Fold enrichment
Neuronal related				
mmu04725	Cholinergic synapse	0.0124	GNA11, KCNJ14, FYN, GNG12, ADCY6	5.48
mmu04724	Glutamatergic synapse	0.0132	GRIA1, PPP3R1, SLC1A1, GNG12, ADCY6	5.39
mmu04722	Neurotrophin signaling pathway	0.0161	MAP3K3, SHC1, PSEN2, BRAF, FOXO3	5.08
mmu04360	Axon guidance	0.0574	PPP3R1, RASA1, NCK2, FYN, WNT4	3.39
Immune related				
mmu04062	Chemokine signaling pathway	0.0189	SHC1, BRAF, FOXO3, GNG12, CX3CL1, ADCY6	3.84

**Table 3:** Neuronal, cellular adhesion and migration-related GO terms enriched in miR-183C targets in the cornea of SNS-CKO and KO mice.

GO ID	Term	P	Genes	Fold Enrichment
Neuronal function related				
0010976	Positive regulation of neuron projection development	3.21E-04	ANKRD27, FUT9, AVIL, DAB2IP, CNTN1, FYN, PRKD1, CX3CL1	6.16
2000300	Regulation of synaptic vesicle exocytosis	0.002954	NRN1, SV2C, PRKCE, PRKCA, CACNA1E	8.38
0001764	Neuron migration	0.004744	MEF2C, TRIM46, FYN, FAT3, RAC1, CELSR3	5.48
0048813	Dendrite morphogenesis	0.005398	KLF7, ELAVL4, FYN, RAC1	11.18
0030030	Cell projection organization	0.014212	UBXN10, RAB34, GPR22, TSC1, FAM149B, TAPT1	4.19
0030517	Negative regulation of axon extension	0.0173	TRIM46, AATK, RTN4	14.75
0048812	Neuron projection morphogenesis	0.022254	MAP2K1, ANKRD27, DAB2IP, RAC1	6.63
0010977	Negative regulation of neuron projection development	0.024328	TSC1, HES1, RTN4, PTPRG	6.41
0048870	Cell motility	0.030182	MAP2K1, ELMO1, RAC1	10.96
0050772	Positive regulation of axonogenesis	0.033101	ROBO2, ZEB2, MAP2K1	10.43
0007411	Axon guidance	0.044784	ROBO2, KLF7, CNTN1, UNC5D, RAC1	3.73
Cellular adhesion and migration related				
0007155	Cell adhesion	0.001191	CD164, PRKCE, FIBCD1, PRKCA, CX3CL1, PCDH17, CELSR3, CNTN1, HES1, FAT3, TGFBI, RAC1, DGCR2	3.03
0010595	Positive regulation of endothelial cell migration	9.78E-04	ANXA3, PDCD6, PRKCA, PRKD1, RAC1	11.31
0010764	Negative regulation of fibroblast migration	0.002543	ZEB2, RAC1, CYGB	38.87
0022409	Positive regulation of cell–cell adhesion	0.01618	SOX2, MAGI1, PDE4D	15.27

**Table 4:** Neuronal and immune-related KEGG pathways enriched in miR-183C targets in the cornea of SNS-CKO and KO mice.

KEGG ID	Term	P	Genes	Fold enrichment
Neuronal and synaptic function related				
mmu04360	Axon guidance	0.0011	ROBO2, GNAI3, FYN, PRKCA, UNC5D, RAC1, SSH2	5.84
mmu04725	Cholinergic synapse	0.0060	MAP2K1, GNG5, GNAI3, FYN, PRKCA	6.74
mmu04726	Serotonergic synapse	0.0103	MAP2K1, GNG5, DUSP1, GNAI3, PRKCA	5.76
mmu04921	Oxytocin signaling pathway	0.0174	MAP2K1, MEF2C, CACNA2D1, GNAI3, PRKCA	4.94
mmu04727	GABAergic synapse	0.0201	GABARAPL2, GNG5, GNAI3, PRKCA	6.79
mmu04713	Circadian entrainment	0.0258	GNG5, GNAI3, PRKCA, GRIA3	6.16
mmu04724	Glutamatergic synapse	0.0380	GNG5, GNAI3, PRKCA, GRIA3	5.30
Immune function related				
mmu04062	Chemokine signaling pathway	0.0081	MAP2K1, GNG5, ELMO1, GNAI3, RAC1, CX3CL1	4.72
mmu04666	Fc gamma R-mediated phagocytosis	0.0225	MAP2K1, PRKCE, PRKCA, RAC1	6.50
mmu04670	Leukocyte transendothelial migration	0.0414	GNAI3, MSN, PRKCA, RAC1	5.12

**Figure 8:**
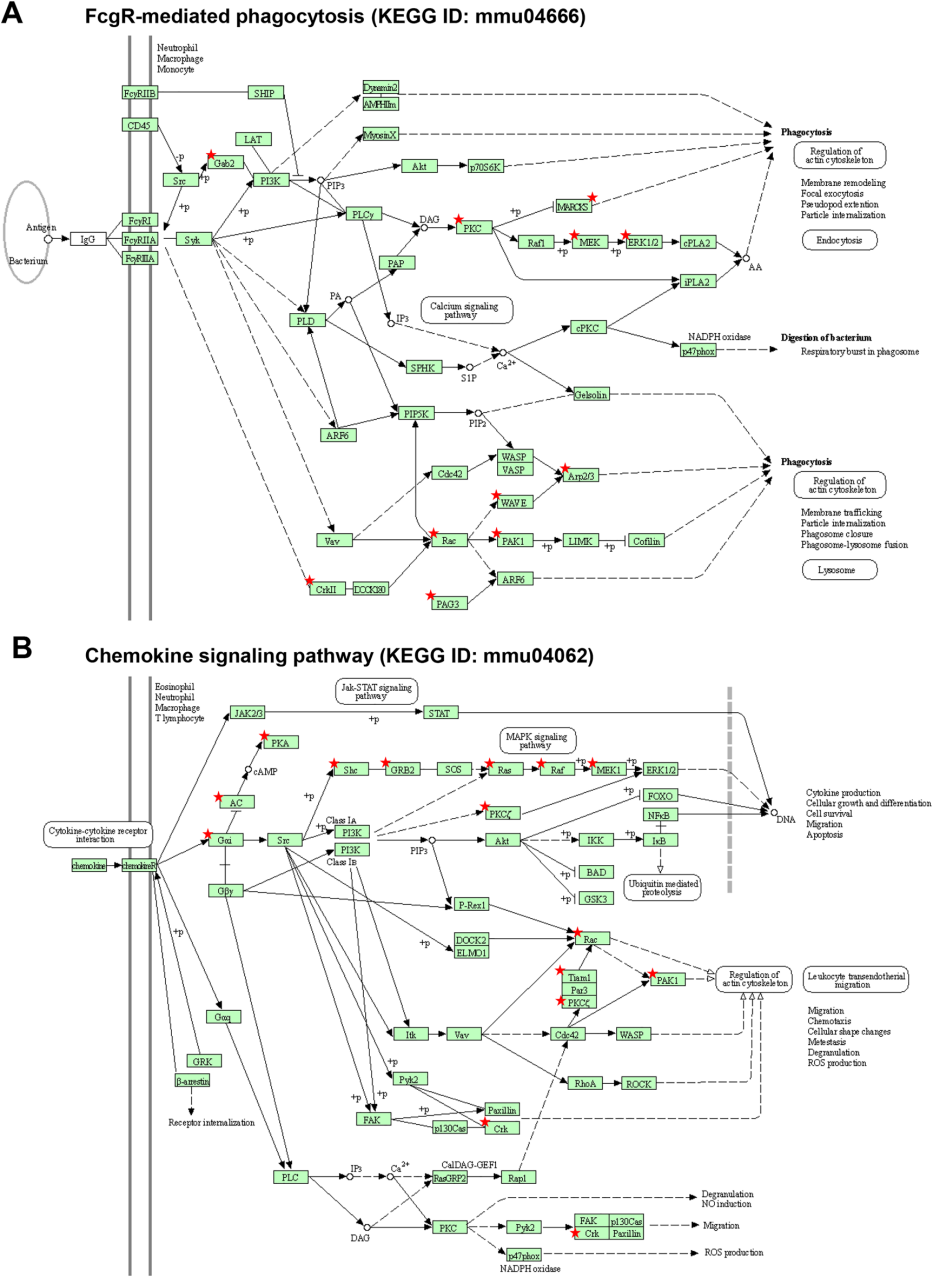
miR-183C target genes in CRMCs are enriched in FcγR-mediated phagocytosis (**A**) and chemokine signaling pathways (**B**). Modified from www.genome.jp/kegg/pathway.html. Star-labeled molecules or complexes are or contain target genes of miR-183C.

To further identify myeloid cell-specific target genes of miR-183C, we isolated the Csf1r-EGFP + CRMCs from young adult miR-183C MS-CKO and conventional KO mice and their age- and sex-matched WT control mice. KEGG pathway analyses of miR-183C targets identified in CRMCs of both MS-CKO and conventional KO (Tables S25 and S26) are consistently enriched in multiple immune/inflammation-related pathways (Table 5; Table S27), including FcγR-mediated phagocytosis (Fig. 8A), chemokine signaling (Fig. 8B), FcεRI signaling, TGFβ signaling pathway, TNF signaling pathways, and several microbial infection pathways (Table 5; Table S27). These data suggest that miR-183C regulates the functions of myeloid cells through simultaneously targeting multiple genes in different pathways. Intriguingly, miR-183C targets are also enriched in neuronal function, especially synaptic function-related pathways (Table S28), and cell–cell interaction pathways (Table S29), suggesting potential indirect modulation of neuronal innervation and other cell types of the cornea.

**Table 5:** Immune/infection-related KEGG pathways enriched in miR-183C targets of CRMCs of KO and MS-CKO mice.

KEGG ID	Term	P	Genes	Fold enrichment
mmu05100	Bacterial invasion of epithelial cells	2.98E-04	ITGB1, ACTR2, CTTN, SHC1, CAV1, ARPC1B, RAC1, WASL, CRK, WASF2, CD2AP	4.12
mmu04666	Fc gamma R-mediated phagocytosis	3.87E-04	ACTR2, MAP2K1, MARCKS, PAK1, PRKCE, ARPC1B, MAPK1, ASAP1, GAB2, RAC1, CRK, WASF2	3.67
mmu04664	Fc epsilon RI signaling pathway	0.002041	MAP2K3, MAPK9, MAP2K1, MAPK1, GRB2, FYN, KRAS, GAB2, RAC1	3.88
mmu05135	Yersinia infection	0.002728	ITGB1, MAP2K3, ACTR2, GIT2, MAP2K1, ARHGEF12, ARPC1B, WASL, MAPK9, MAPK1, RAC1, CRK, WASF2	2.74
mmu04062	Chemokine signaling pathway	0.003045	MAP2K1, SHC1, GNAI3, BRAF, GNG12, PRKCZ, ADCY6, TIAM1, PAK1, GNG5, MAPK1, GRB2, KRAS, RAC1, CRK, PRKACB	2.37
mmu05166	Human T-cell leukemia virus 1 infection	0.006333	ATF2, MAP3K3, EGR1, MAP2K1, CRTC1, XIAP, TGFBR1, ADCY6, PPP3CA, MAPK9, ZFP36, CCND2, CREB3L1, CREB3L2, MAPK1, EP300, KRAS, PRKACB	2.06
mmu05132	Salmonella infection	0.007681	MAP2K3, ACTR2, RALA, MAP2K1, ARPC1B, WASL, PIK3C2A, RHOB, MAPK9, SNX18, PAK1, BCL2, EXOC4, MAPK1, RAC1, PFN1, KPNA3, PFN2	2.02
mmu05163	Human cytomegalovirus infection	0.007979	ATF2, MAP2K1, ARHGEF12, GNAI3, TSC1, GNG12, ADCY6, PPP3CA, GNG5, GNA11, CREB3L1, CREB3L2, MAPK1, GRB2, KRAS, RAC1, CRK, PRKACB	2.02
mmu05165	Human papillomavirus infection	0.015215	MAGI1, ITGB1, PRKCI, MAP2K1, ITGA3, TSC1, PPP2R5C, LAMC1, PRKCZ, PPP2CA, PPP2CB, CCND2, CREB3L1, CREB3L2, MAPK1, EP300, GRB2, HES1, KRAS, WNT2, PRKACB, WNT4	1.74
mmu04650	Natural killer cell mediated cytotoxicity	0.020466	PPP3CA, MAP2K1, PAK1, SHC1, MAPK1, GRB2, FYN, BRAF, KRAS, RAC1	2.45
mmu05145	Toxoplasmosis	0.037313	ITGB1, MAP2K3, MAPK9, GNAI3, BCL2, XIAP, MAPK1, LAMC1, LDLR	2.35
mmu04350	TGF-beta signaling pathway	0.039069	ACVR1, PPP2CA, SMAD1, PPP2CB, EP300, MAPK1, SKIL, TGFBR1, SMAD7	2.33
mmu05170	Human immunodeficiency virus 1 infection	0.040389	MAP2K3, MAP2K1, FBXW11, GNAI3, GNG12, PPP3CA, MAPK9, PAK1, GNG5, GNA11, BCL2, MAPK1, KRAS, RAC1, CRK	1.79
mmu04668	TNF signaling pathway	0.048672	MAP2K3, CYLD, MAPK9, ATF2, MAP2K1, CREB3L1, CREB3L2, XIAP, MAPK1	2.23

### Cx3cl1 and a dozen of other genes were validated as miR-183C targets in TG sensory neurons.

**Figure 9:**
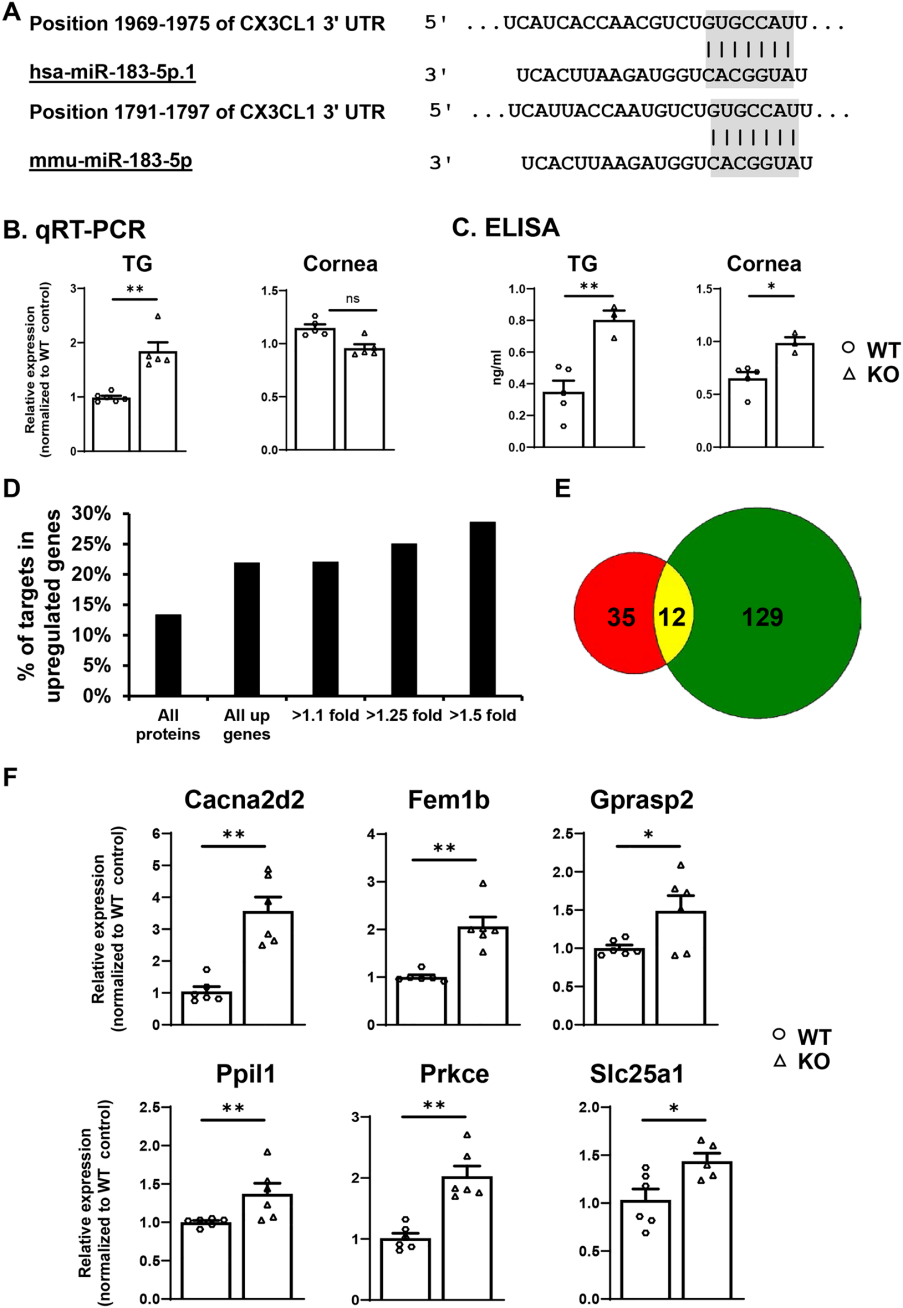
Validation of miR-183C target genes by qRT-PCR, ELISA assay and proteomics. (**A**) Sequence alignment of miR-183C with its target site in in 3′ untranslated region (UTR) of human (upper) and mouse Cx3cl1 transcript (lower panel). (**B**) qRT-PCR; (**C**) ELISA assay on Cx3cl1 in the TG and cornea of miR-183C KO and WT control mice. (**D**,**E**) Proteomics study in the TG of KO and WT control mice. (**D**) Percentage of miR-183C target genes in upregulated proteins of the TG of KO vs WT control mice; (**E**) Venn diagram of miR-183C target genes detected by proteomics (Red) and 3′ RNA seq (Green). 12 target genes (yellow) were detected by both methods; 6 of which were further validated by qRT-PCR (**F**).

Cx3cl1, also called fractalkine or neurotactin, is a type I transmembrane (TM) chemokine and is the only member of the CX3C or delta chemokine subfamily^101–103^. It is known to promote chemotactic migration of microglia in the central nervous system (CNS)^104,105^, mediate the homing of resident myeloid cells in the cornea^106^ and recruitment of Mϕ in other tissues^107^. RNA seq data revealed that Cx3cl1 was a potential target gene of miR-183C. It is significantly increased in the TG of miR-183C KO (Table S4) and the cornea of SNS-CKO mice (Table S1) by ~ 1.89 and 1.42 folds, respectively, when compared to their WT controls. Target prediction showed that Cx3cl1 held a conserved target site for miR-183 in its transcript of both human and mouse (Fig. 9A). To validate these findings, we performed qRT-PCR in the TG and corneas of the miR-183C KO and age- and sex-matched WT control mice. Our results confirmed significant upregulation of Cx3cl1 in the TG of the KO vs WT control mice, although no significant change of Cx3cl1 was detected in the cornea (Fig. 9B).

miRNAs regulate downstream genes by base-pairing to the target sites in their transcripts to induce the breakdown and/or translation inhibition of the mRNAs^35^. This regulation ultimately results in decreased expression at protein level^35^. To further validate the observation of Cx3cl1 at the protein level, we performed ELISA assay in the TG and corneas of miR-183C KO and age- and sex-matched WT controls. Our results showed that CX3CL1 was significantly upregulated in both TG and cornea in the KO vs WT control mice at its protein level (Fig. 9C), supporting that miR-183C targets Cx3cl1 in TG sensory neurons in vivo.

To systemically validate the 3′ RNA seq-identified miR-183C targets at the protein level, we performed proteomics in the TG and corneas of the miR-183C KO mice and age- and sex-matched WT littermate controls. Our results showed that predicted target genes of miR-183C (Table S31) were significantly enriched (*p* = 0.0006) in the upregulated proteins in the TG of the KO vs WT control mice (Table S30), suggesting specific detection of miR-183C target genes. Consistently, the percentage of miR-183C target genes among the upregulated proteins increased in correlation with the extent of upregulation (Fig. 9D). Twelve (25.5%) of the 47 target genes detected by proteomics (Table S31), including Cacna2d2, Eea1, Femb1, Gprasp2, Hook3, Nedd4l, Ppil1, Prkce, Rbm25, Slc25a1, were also detected by 3′ RNA sequencing (Fig. 9E). Therefore, these 12 target genes of miR-183C are validated at the protein level. To further validate these target genes at RNA level, we performed qRT-PCR on six of the 12 targets validated by proteomics, including Cacna2d2, Fem1b, Gprasp2, Ppil1, Prkce, and Slc25a1. All 6 were confirmed to be significantly upregulated in the TG of the KO vs age- and sex-matched WT control mice (Fig. 9F). Among these, Gprasp2 and Prkce were also upregulated in the cornea of the KO (Fig. S5A) and in the TG of SNS-CKO vs their corresponding age- and sex-matched WT control mice (Fig. S5B), respectively.

For the proteomics in the cornea, similar with the RNA seq data, predicted miR-183C target genes (Table S33) showed no enrichment in the upregulated proteins (Table S32), possibly a result of the low representation of proteins from corneal sensory nerves and CRMCs in total corneal proteins^4,7^.

## Discussion

To delineate the cell type-specific functions of miR-183C in corneal sensory nerves and innate immune cells, we created and characterized a SNS- and a MS-CKO mouse models, in comparison to the conventional KO mice. Our data showed that inactivation of miR-183C in corneal nerves, but not in innate immune cells, resulted in decreased corneal sensory nerve density and reduced sensitivity to mechanical stimuli, suggesting intrinsic regulation of miR-183C on corneal sensory innervation. Our data showed that, in the SNS-CKO mice, the reduction of sensory nerve density occurs specifically in the fine, terminal neurites in the epithelial layer, including the subbasal plexus, while the large-diameter nerves in the stromal layer remain unaffected, suggesting a critical role of miR-183C in the terminal differentiation and projection of corneal sensory nerves. This is consistent with its functions in the other sensory organs, e.g. the photoreceptors of the retina^51,54^ and the inner ear hair cells^108,109^, as well as myeloid cells^59^, suggesting a common feature of miR-183C regulating terminal functional differentiation of primary sensory neurons and innate immune cells. Consistent with this observation, our RNA seq data showed that target genes of miR-183C in the TG were significantly enriched with genes involved in axonal guidance and neuronal projection regulation as well as synaptic functions, suggesting that miR-183C regulates corneal sensory nerve functions by directly targeting neuronal projection and synaptic release-related genes. Multiple genes in the same pathway are simultaneously targeted. This exemplifies the mode of action of miRNAs—quantitatively regulating multiple genes in the same pathway. Their effect on each individual gene may not cause a major phenotypic change; however, working in concert, they impose significant functional consequence^7,110–113^. In this case, the simultaneous regulation of multiple genes in the neuronal projection and synaptic functional pathways contributes to the decreased sensory nerve density and levels of neuropeptides in the cornea of the SNS-CKO and miR-183C KO mice, which affects the neuroimmune response of the cornea to bacterial infection^60^.

Comparing to the conventional miR-183C KO mice, the decrease of the corneal sensitivity to mechanical stimuli was modest (~ 10% vs 23–33% in conventional KO mice). This is most possibly a result of the incomplete inactivation of miR-183C in the SNS-CKO mice. In the conventional miR-183C KO model, the miR-183C is inactivated in all cells that normally express miR-183C, including all TG sensory neurons^51^. However, the SNS-CKO is based on Na_v_1.8-promoter driven Cre recombinase-induced deletion of miR-183C^76,78,82^. Although it is expressed in nearly all small-diameter nociceptors, Na_v_1.8-Cre is expressed in less than a third of the large-diameter mechanoreceptive and proprioceptive neurons^78,114–116^. Therefore, miR-183C is possibly intact in two thirds of the mechanoreceptors in the SNS-CKO mice, resulting in only modest decrease of corneal sensitivity to mechanical stimuli measured by the Cochet and Bonnet aesthesiometer in this study.

MS-CKO of miR-183C resulted in increased CRMCs, suggesting intrinsic regulation of miR-183C in CRMCs. Our comparative study of the transcriptomes of CRMCs of miR-183C KO and MS-CKO mice vs their WT controls revealed myeloid-specific target genes of miR-183C, which are significantly enriched in a range of immune/infection related pathways, including chemokine signaling and phagocytosis pathways. These data provide new insight into the mechanisms of intrinsic regulation of miR-183C on the functions of innate myeloid cells, e.g. cytokine production and phagocytosis and intracellular killing capacity that we reported previously^7,60,61^.

Intriguingly, SNS-CKO of miR-183C also led to increased number of CRMCs in naïve mouse cornea, suggesting that normal function of miR-183C in sensory nerves has an extrinsic regulation on the establishment of CRMCs, indicative of neuroimmune interaction in the maintenance of the homeostasis of the cornea. KEGG pathway analysis of miR-183C target genes in TG sensory neurons showed significant enrichment of genes involved in chemokine signaling pathway, including Cx3cl1. Inactivation of miR-183C-resulted disinhibition/upregulation of Cx3cl1 was further validated by qRT-PCR and ELISA assays. Neuron-produced chemokine Cx3cl1, also called fractalkine, is known to promote chemotactic migration of microglia in the CNS^104,105^, mediate the homing of CRMCs^106^ and recruitment of Mϕ in other tissues^107^. These observations support a hypothesis that inactivation of miR-183C disinhibits Cx3cl1 in the TG sensory neurons and resulted in increased expression of Cx3cl1 in the cornea of the SNS-CKO mice, which enhances the recruitment of myeloid cells to the cornea, resulting in an increased number of the CRMCs. Additional studies are warranted to further test this hypothesis. If proven, it will uncover a new mechanism underlying the extrinsic regulation of miR-183C in TG neurons on the establishment of the CRMCs in the cornea.

Furthermore, our RNA seq data showed that miR-183C target genes in TG sensory neurons are also enriched in pathways regulating other corneal cell types, e.g. epithelial, endothelial cell and fibroblast migration; while target genes in CRMCs are also enriched in neuronal synaptic functions and cell–cell interaction and migration pathway. These data suggest that, in addition to the intrinsic regulation of sensory innervation and CRMCs, miR-183C exerts extrinsic regulation on neuro-immune-epithelial-stromal-endothelial interactions. This is consistent with our recent report of a single-cell transcriptome study that miR-183C serves as a checkpoint of corneal resident immune cells and shapes the cellular landscape of the cornea^4^. Different cell types form the microenvironment or niche of the cornea and work in concert to endow the cornea with its unique architecture and functionalities and maintain homeostasis^1–4^. Perturbation of the functions of one cell type imposes global impact on other cell types and the overall function of the cornea in both physiological and various pathological conditions^4^.

In addition to increased numbers of CRMCs in both MS-CKO and SNS-CKO vs their corresponding WT control mice, we identified sex-dependent differences of the number of CRMCs in both MS-CKO and SNS-CKO. Myeloid-specific inactivation of miR-183C resulted in an increased number of CRMCs in male vs female MS-CKO mice, while sensory neuron-specific knockout of miR-183C led to a decreased trend of CRMCs in the male vs female SNS-CKO mice, although there were no differences of CRMC numbers between male and female WT mice of both MS-CKO and SNS-CKO strains. Sex-dependent differences in immune cell composition and immune responses are affected by sex chromosome-linked genes, sex hormones and variations in levels of sex hormones and their receptors overtime^118^. Although sex differences in both innate and adaptive immunity have been well documented^119,120^, insights into sexual dimorphism of tissue *resident* immunity remains sparse^118,121–123^ and completely unknown in CRICs. Based on our observations, we predict that miR-183C regulates sex chromosome-linked genes, and/or sex hormones and/or their receptors, and/or other genes involved in the establishment of CRICs. Inactivation of miR-183C alters the expression levels of these genes, leading to changes of the numbers of CRMCs through intrinsic and/or extrinsic mechanisms, in which neuroimmune interaction may play an important role. The fact that tissue-specific inactivation of miR-183C in myeloid cells or sensory neurons resulted in opposite trends of the numbers of CRMCs suggests tissue-specific mechanisms of miR-183C in TG sensory neurons and myeloid cells regulating the establishment of CRMCs. Major efforts in the future are warranted to test these hypotheses.

For the first time, here we discovered that both naïve miR-183C KO and SNS-CKO, but not the MS-CKO mice, had significantly decreased tear volume, when compared to their age- and sex-matched WT controls. These data suggest miR-183C enhances basal tear production through its regulation of corneal sensory innervation. It is known that the primary afferent sensory neurons innervating the cornea play important roles in the basal tear production and secretion evoked by noxious stimulation of the cornea through the brainstem tear reflex arc^27^. Corneal sensory neurons sense dry condition and other environmental stressors, send primary afferent projections to activate the neurons in the spinal trigeminal nucleus (Vsp) and regulates lacrimation through the efferent autonomic arm of the reflex arc, which include the preganglionic parasympathetic neurons in/around the superior and inferior salivatory nuclei (SSN and ISN) and postganglionic parasympathetic neurons in the pterygopalatine ganglion (PG)^27^. The autonomic reflex promotes the production of the watery component of the tears by the lacrimal glands; mucin component of the tears by the conjunctival goblet cells, and lipid content by the meibomian glands^27^. We speculate that decreased corneal sensory innervation caused by loss of miR-183C in sensory neurons imposes a negative impact on proper lacrimation through the cornea-brainstem tear reflex arc. In addition, tear producing tissues, including lacrimal glands (Fig. S6), meibomian glands and conjunctival goblet cells are subjected to sensory innervation themselves^124–131^. Based on its effect in corneal sensory innervation, we predict that inactivation of miR-183C in sensory neurons innervating these tissues may result in defects in sensory nerve density in them. Currently, little is known about whether and how sensory innervation of lacrimal and meibomian glands contributes to tear volume^128,132^. Nevertheless, our data suggest that miR-183C may play a role in dry eye disease (DED). Additional studies dedicated to these topics are warranted to confirm these hypotheses and uncover the underlying mechanisms.

To our surprise, we observed significant decrease of basal tear volume in female vs male mice in all three strains used in this study, which are in a mixed genetic background of C57BL/6 and129S^51^. It is well known that DED is more prevalent in women compared to men^133–143^, especially in the autoimmune DED, Sjögren’s syndrome^144^. Over 90% of all patients with Sjögren’s syndrome are women^144^. The female gender is considered a risk factor for the development of DED^133^. Gender and sex steroids are known to have significant and multitudes of impact on the morphology and functions of tear producing tissues including the lacrimal gland^141–143,145–148^, meibomian glands^128,131^ and conjunctival goblet cells^149^. The acinar area of the lacrimal glands of males are larger than that of females in multiple species including human^146^. Sex hormones modulates tear volume, secretory components, protein contents and lipid profiles of the tears^128,141–143,146–148^. Androgen promotes lipogenesis in the meibomian gland; androgen deficiency leads to meibomian gland dysfunction (MGD) and evaporative dry eye, while estrogen reduces lipid synthesis in the meibomian gland and promote both MGD and evaporative dry eye^128^. Androgen receptors are only detected in male but not female goblet cells^149^. Human male and female goblet cells have different responses to pro-inflammatory and pro-resolving mediators^149^. In spite of these observations, sex-related difference in basal tear volumes has not been reported in either humans or animals^141,145,148^. Our observation of decreased tear volume in female mice appears to be in-line with the fact that female gender has been considered a risk factor for the development of DED^133^. The discrepancy between our observation and others^145,150^ could come from different species and/or strains of animals as well as different methodology of measurement^151^. Nevertheless, our data suggest a sex-dependent difference in basal tear volume and further studies are warranted to uncover the underlying mechanisms.

To our knowledge, this is the first example that a miRNA cluster plays significant, functional roles in the corneal homeostasis through direct regulations of both sensory innervation and CRMCs and modulation of neuroimmune interaction. The SNS-CKO and MS-CKO mouse models developed in this report provide critical tools to fully uncover the roles of miR-183C in sensory nerves or myeloid cells under different pathological conditions, e.g., bacterial keratitis and DED. Such knowledge will be essential to develop cell type-specific strategies targeting miRNAs for the treatment of corneal diseases.

## Supplementary Information

Supplementary Information.

## Data Availability

The datasets of 3′ RNA sequencing generated during the current study are available in the Gene Expression Omnibus (GEO) repository (accession number: GSE250213). The proteomics data have been submitted to the repository Proteomics IDEntifications Database (PRIDE. Accession number: PXD05033). All data and protocols generated in this study are available upon request from the corresponding author.
