# Correction: The miR-183/96/182 cluster regulates sensory innervation, resident myeloid cells and functions of the cornea through cell type-specific target genes

**DOI:** 10.1038/s41598-025-08233-6

**Published:** 2025-06-25

**Authors:** Naman Gupta, Mallika Somayajulu, Katherine Gurdziel, Giovanni LoGrasso, Haidy Aziz, Rita Rosati, Sharon McClellan, Ahalya Pitchaikannu, Manoranjan Santra, Muhammed Farooq Abdul Shukkur, Paul Stemmer, Linda D. Hazlett, Shunbin Xu

**Affiliations:** 1 Department of Ophthalmology, Visual and Anatomical Sciences, School of Medicine, Wayne State University, 540 E Canfield Street, Detroit, MI 48201 USA; 2 Genome Sciences Core, Wayne State University, Detroit, MI USA; 3 School of Biological Sciences, Wayne State University, Detroit, MI USA; 4 Institute of Environmental Health Sciences, Wayne State University, Detroit, MI USA

Correction to: *Scientific Reports* 10.1038/s41598-024-58403-1, published online 01 April 2024

The original version of the Article contained errors in Figure 1A, where images demonstrating inactivation of miR-183C were mislabeled. This error does not affect the conclusions of the article.

The original Figure 1 and accompanying legend appear below.

**Fig. 1:**
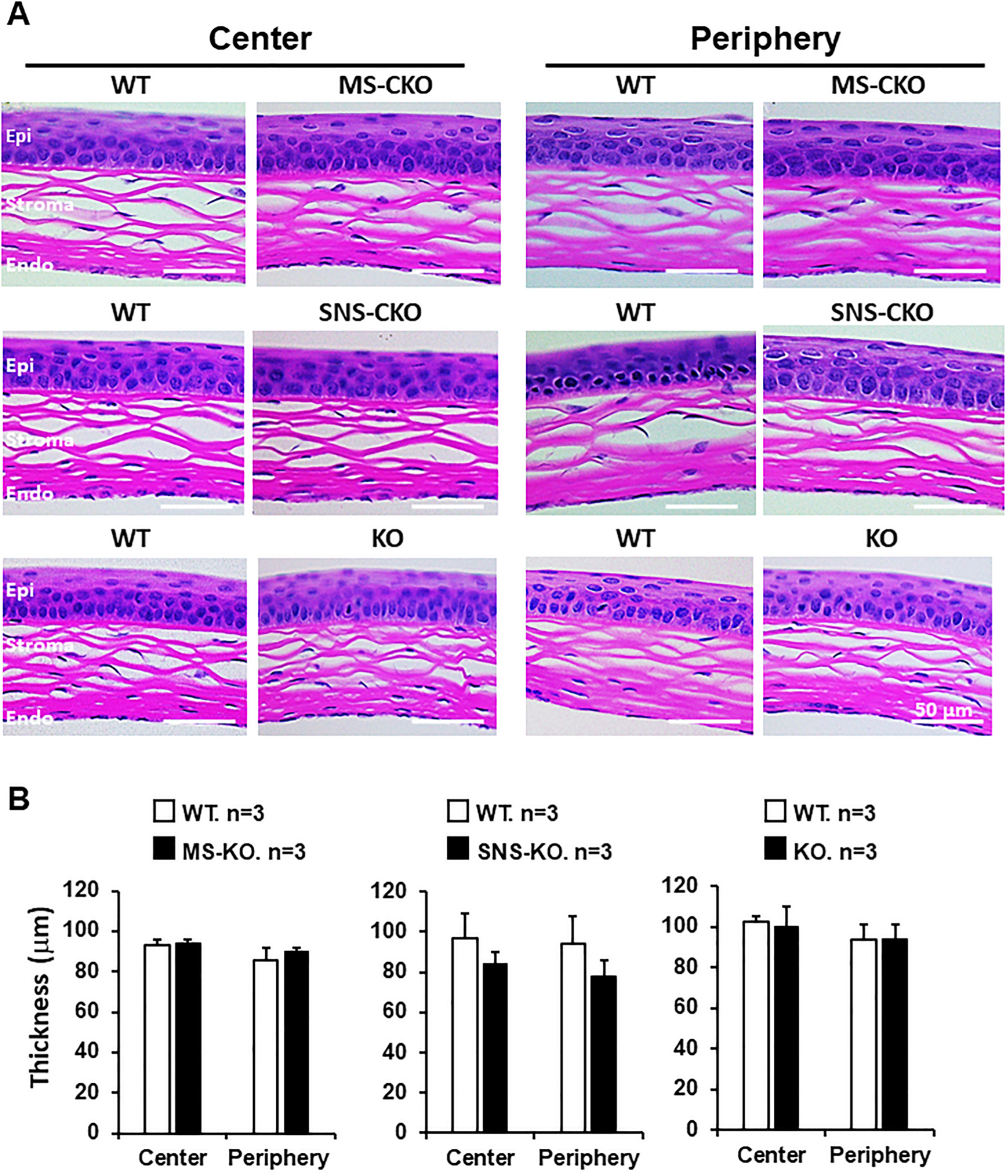
Inactivation of miR-183C has no significant impact on the gross histological architecture of the cornea. (**A**) H&E staining of cross-sections of the corneas of MS-CKO, SNS-CKO and miR-183C conventional KO and age- and sex-matched WT control littermates. (**B**) Measurement of the thickness of the corneas. *Epi* epithelium, *Endo* endothelium.

The original Article has been corrected.

