# Supplementary material for: The miR-183/96/182 cluster regulates sensory innervation, resident myeloid cells and functions of the cornea through cell type-specific target genes
Source: Sci Rep. 2024 Apr 1;14:7676. doi: 10.1038/s41598-024-58403-1 (PMC10985120; doi:10.1038/s41598-024-58403-1)
Supplement: Supplementary file 1 — Supplementary Information. [file 41598_2024_58403_MOESM1_ESM.pdf]

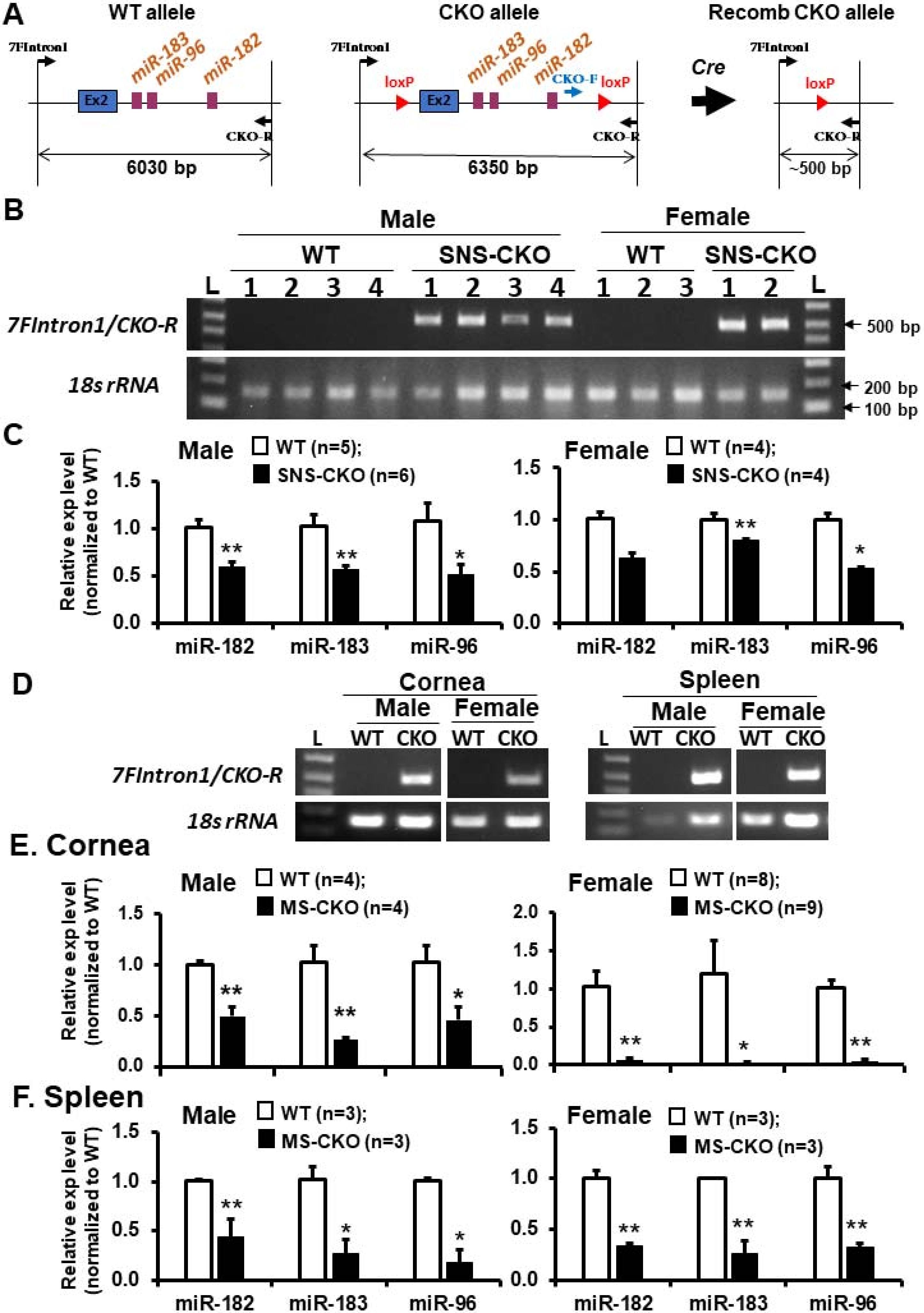

A
WT allele
CKO allele
Recomb CKO allele
7FIntron1
miR-183
miR-96
miR-182
Ex2
loxP
CKO-F
CKO-R
Cre
6030 bp
6350 bp
~500 bp
B
Male
Female
WT
SNS-CKO
L
7FIntron1/CKO-R
18s rRNA
500 bp
200 bp
100 bp
C
WT (n=5);
SNS-CKO (n=6)
WT (n=4);
SNS-CKO (n=4)
Relative exp level (normalized to WT)
D
Cornea
Spleen
E. Cornea
WT (n=4);
MS-CKO (n=4)
WT (n=8);
MS-CKO (n=9)
F. Spleen
WT (n=3);
MS-CKO (n=3)

Figure S1. miR-183C is inactivated in the TG of SNS-CKO and in Csf1r-EGFP+ myeloid cells of MS-CKO mice. A. Illustration of the miR-183C WT allele, floxed CKO allele before and after Cre-induced recombination. 7FIntron1 and CKO-R are primers to detect the recombination event. CKO-F and CKO-R are primers used in routine genotyping PCR. The sizes of the amplicons are labeled under the double arrows. B, D. Genotyping PCR using genomic DNAs of TG of SNS-CKO (B) or Csrf1-EGFP+ myeloid cells of the cornea and spleen of MS-CKO and their age- and sex-matched WT control mice (D). Only the SNS-CKO or MS-CKO samples, but not the WT controls, are positive to recombinant allele. The amplicon of the WT allele is too big to be amplified under this condition. C, E, F. qRT-PCR of miR-183C in TG of SNS-CKO (C) or Csrf1-EGFP+ myeloid cells of the cornea and spleen of MS-CKO and their age- and sex-matched WT control mice (E,F). *: $p<0.05$; **: $p<0.01$.

Appendix 1 of Fig.S1

Original gel images for Panel B

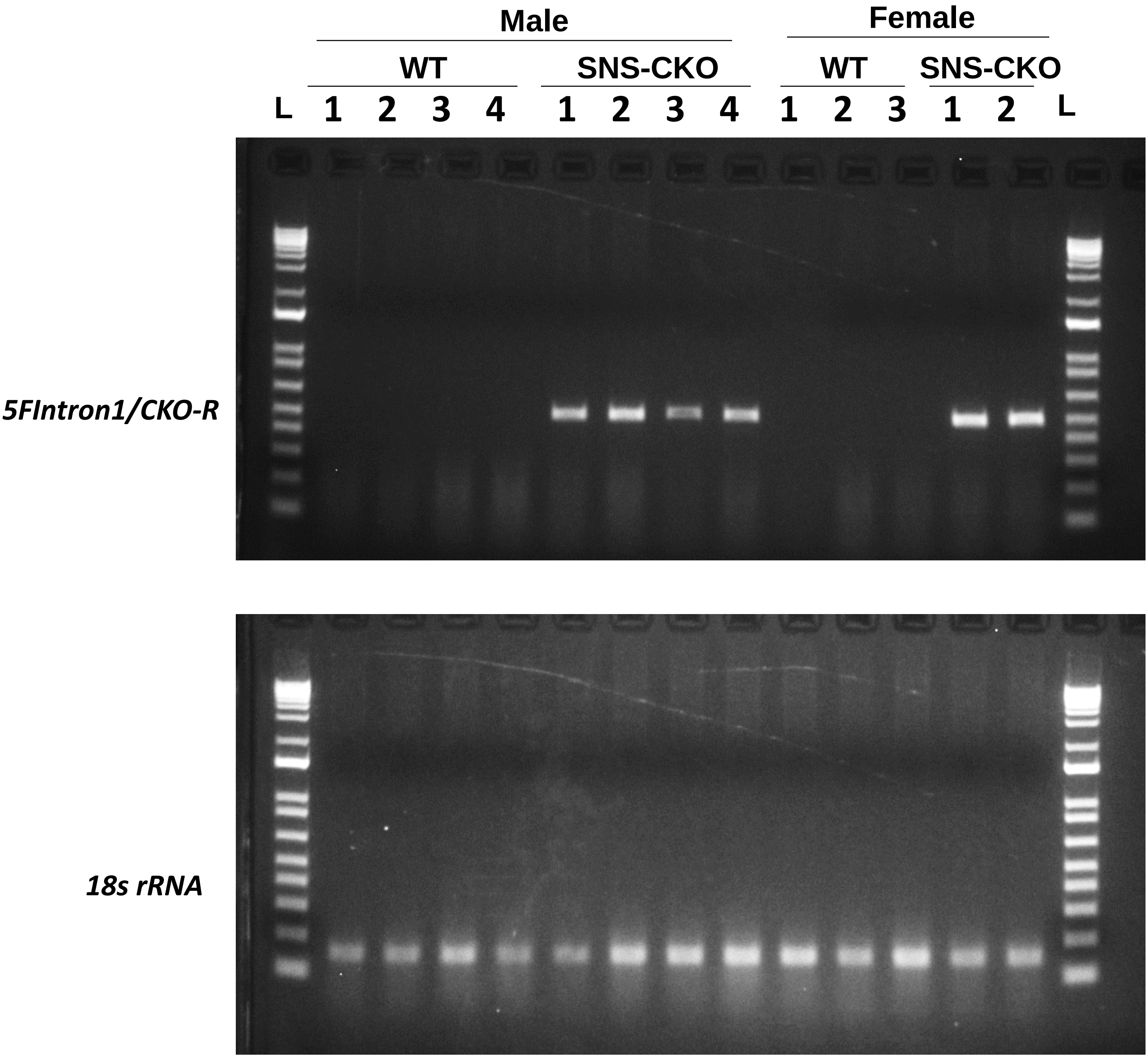

Appendix 2 of Fig.S1

Original gel images for Panel D

This challenging project involves FACS cell sorting of rare myeloid cells in the cornea and spleen followed by DNA prep and PCR. Multiple experiments were performed to obtain data in two different tissues in both male and female, CKO and WT mice.

Panel D male cornea
5Fintron1/CKO-R amplicon

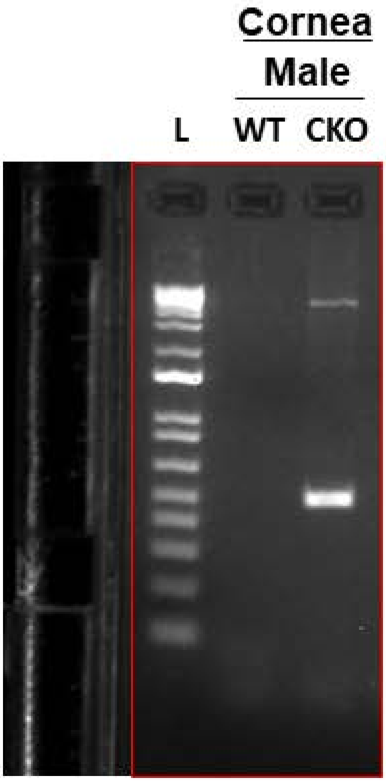

For Panel D female cornea
5Fintron1/CKO-R amplicon

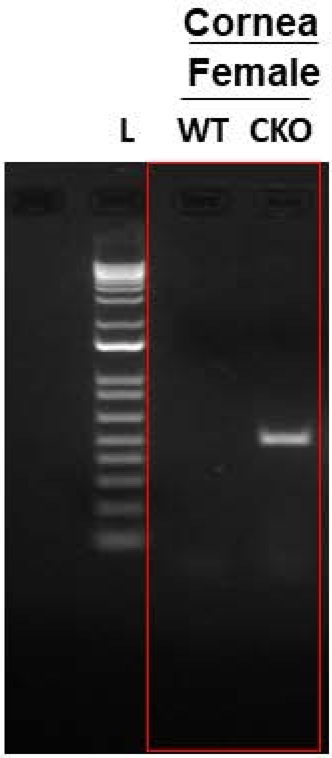

18s RNA amplicon

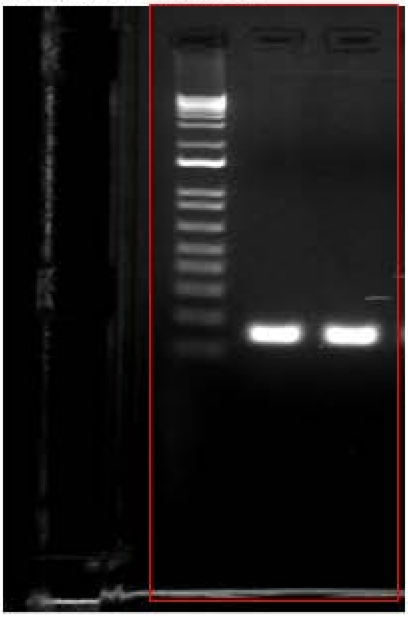

18s RNA amplicon

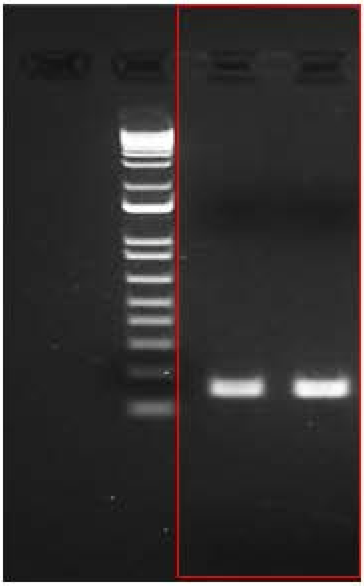

The red squares delineate the lanes displayed in Fig.S1

Appendix 3 of Fig.S1

Original gel images for Panel D

For Panel D male spleen
5Fintron1/CKO-R amplicon

For Panel D female spleen

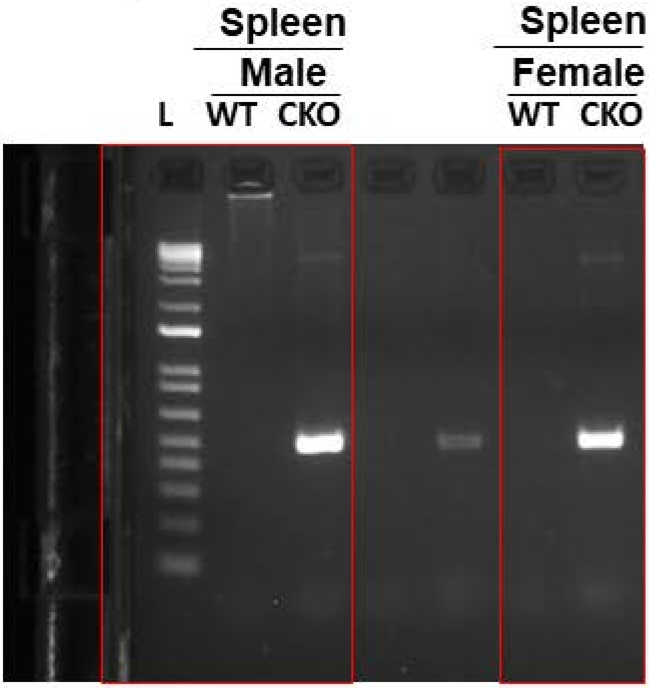

18s rRNA amplicon

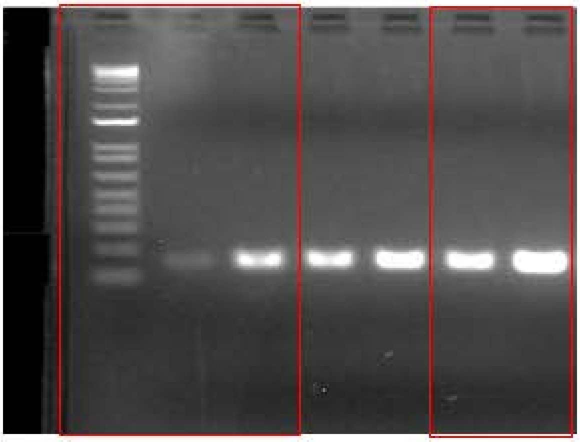

The red squares delineate the lanes displayed in Fig.S1

A. Whorl center region

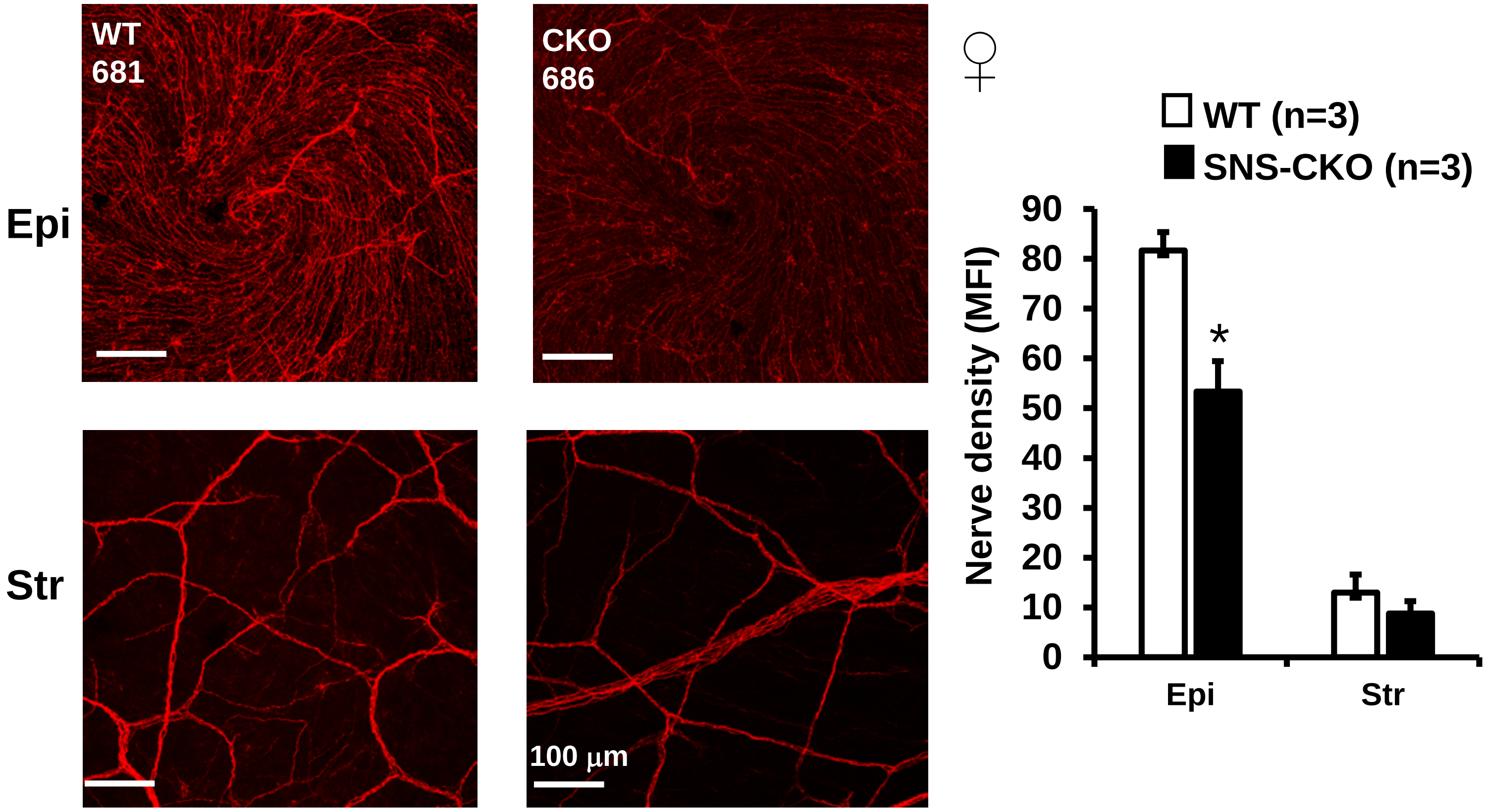

B. Peripheral cornea

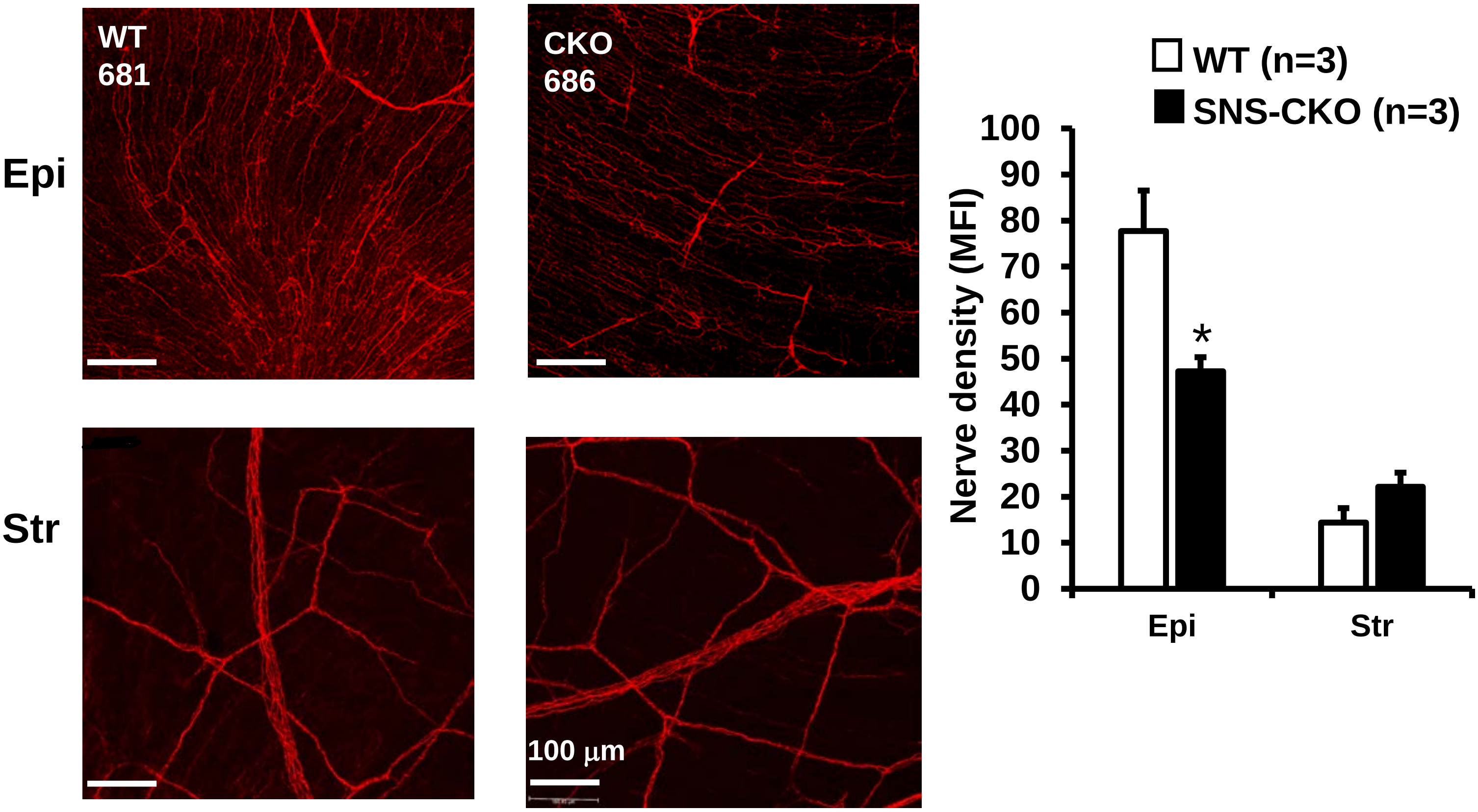

Figure S2. Sensory nerve density in the epithelial layer is decreased in female SNS-CKO vs age-matched WT control mice. Epi: epithelial; Str: stromal. *: $p<0.05$. MFI: mean fluorescence intensity.

A. Cornea

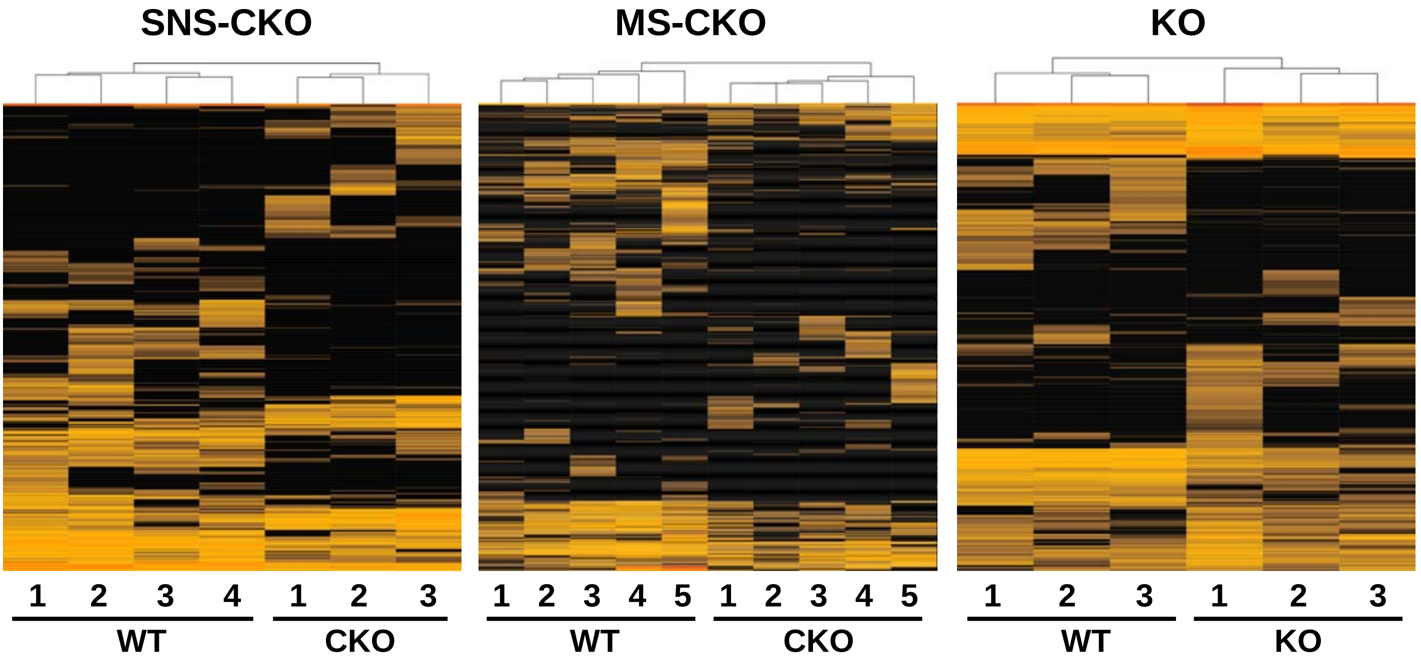

B. TG

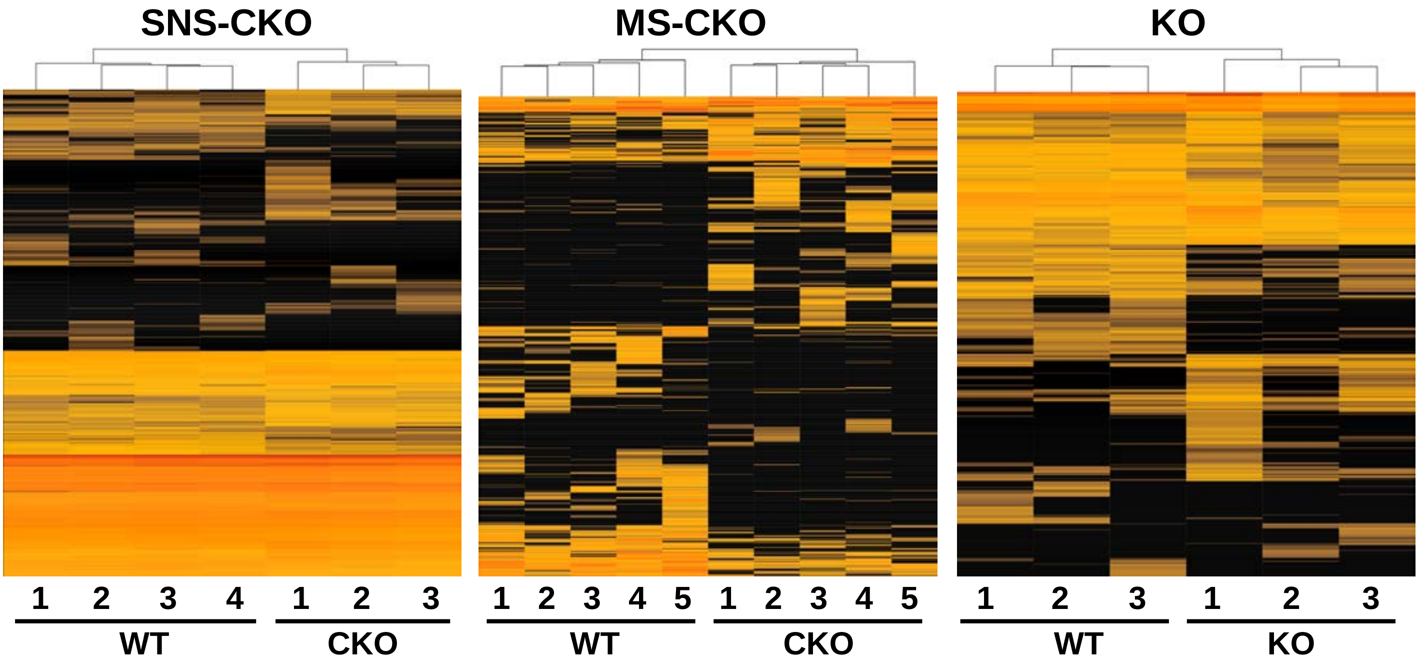

Figure S3. Heatmap of differentially expressed genes in the cornea (A) and TG (B) of miR-183C SNS-CKO, MS-CKO and KO vs their age- and sex-matched WT control mice.

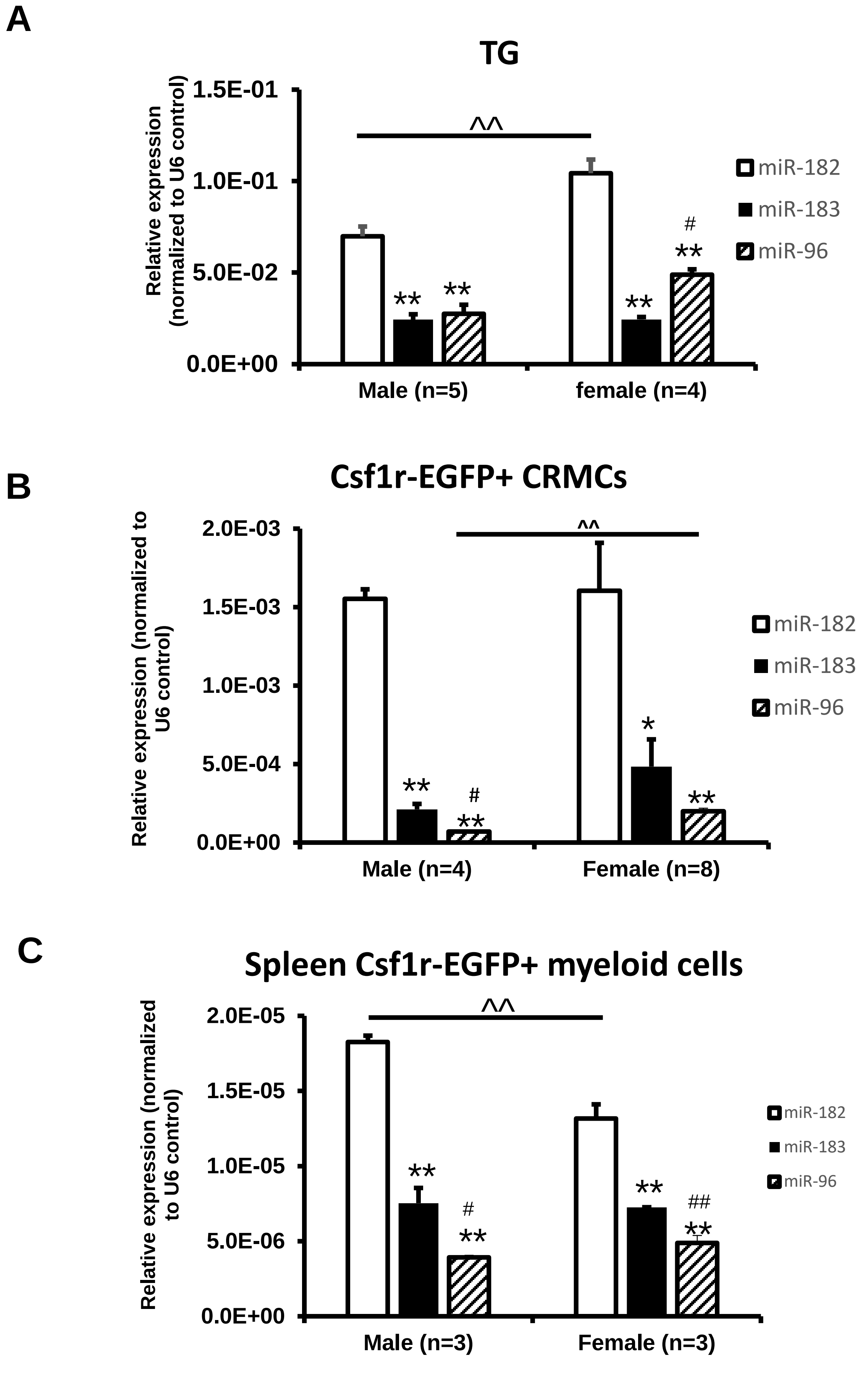

Figure S4. qRT-PCR of miR-183C in the TG (A) and Csf1r-EGFP+ myeloid cells of the cornea (B) and spleen (C) of naïve WT controls mice. **: p<0.01 (compared to miR-182); #: p<0.05; ##: p<0.01 (compared to miR-183); ^^: p<0.01 male vs female.

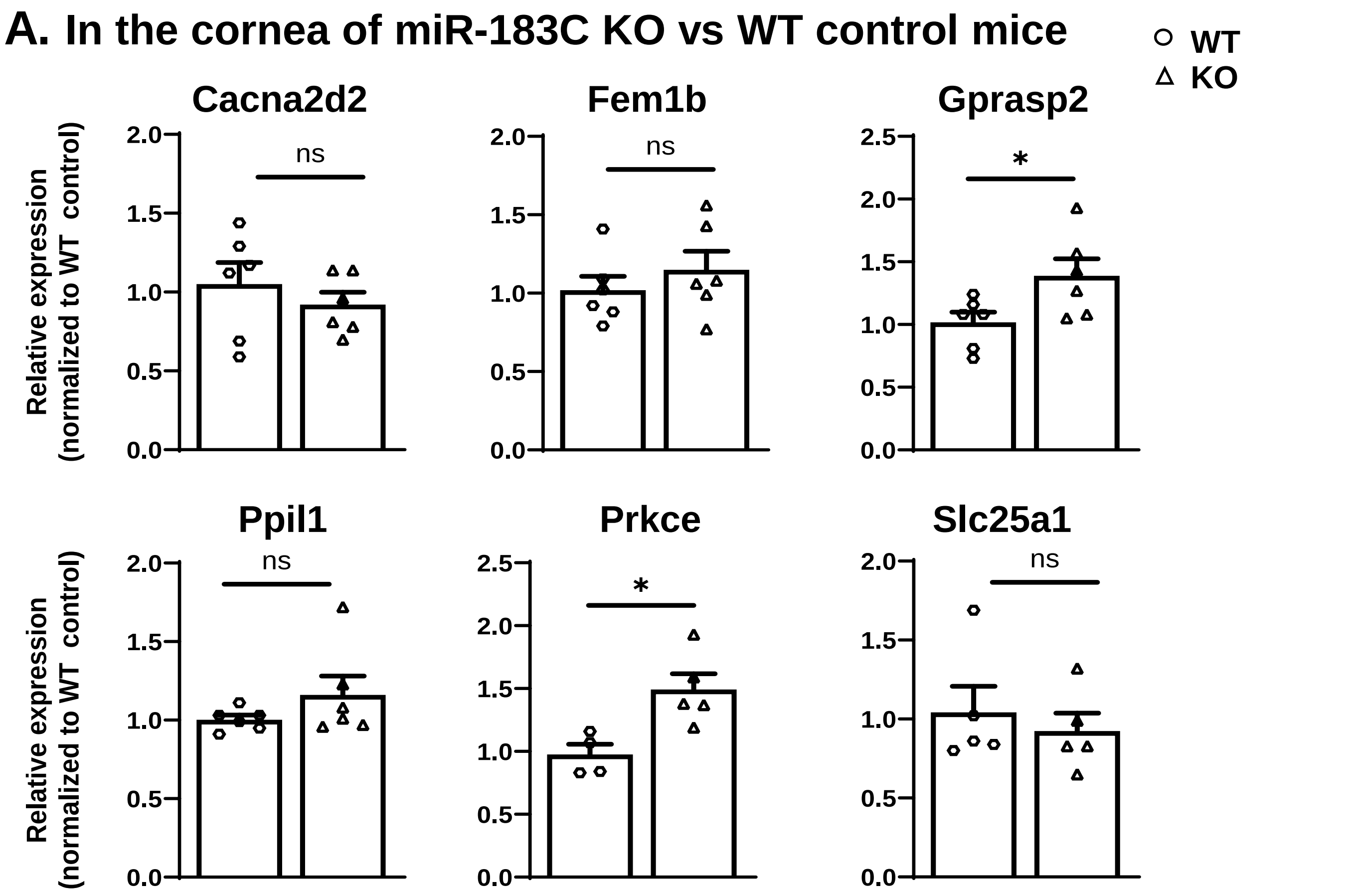

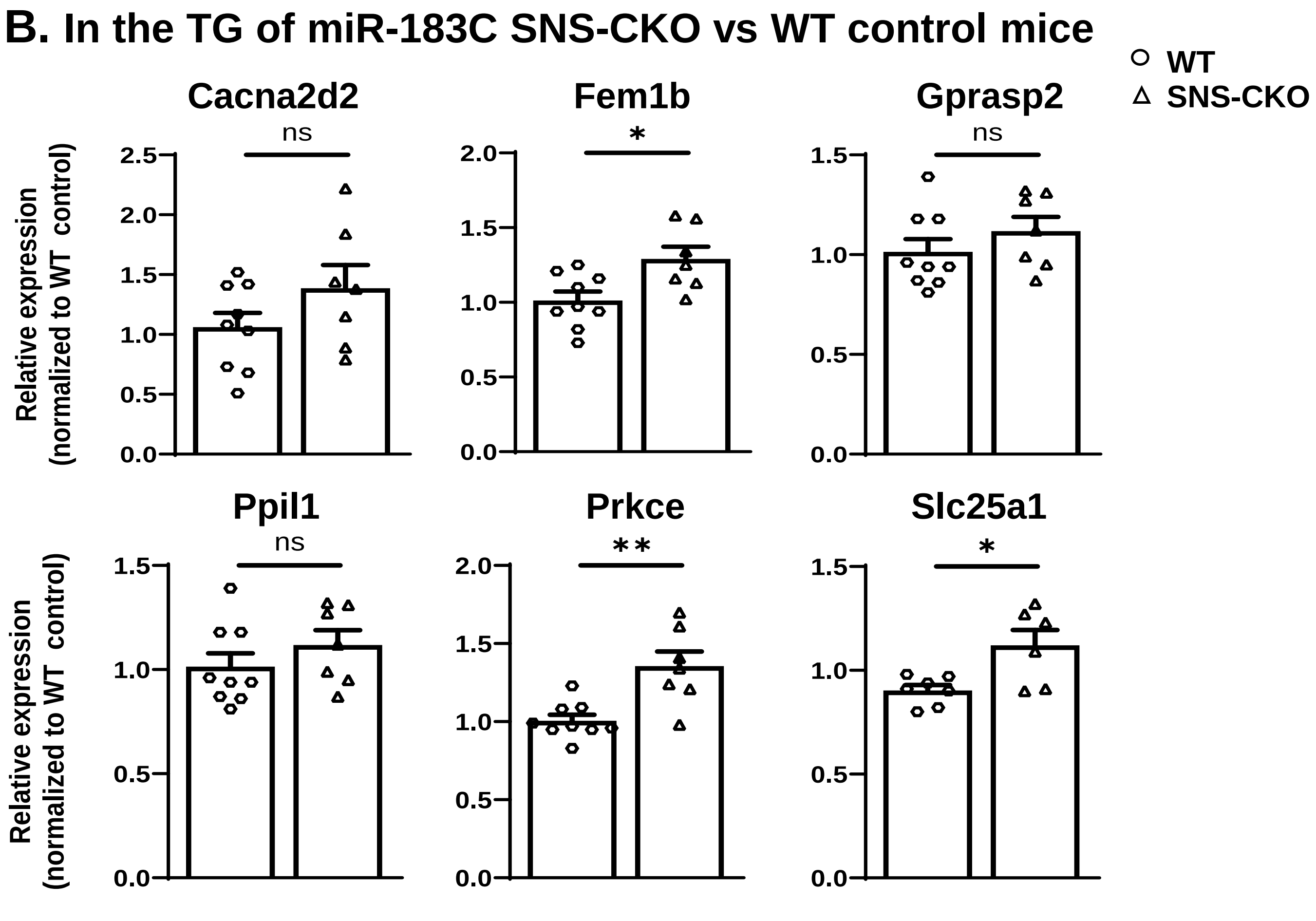

Figure S5. Validation study by qRT-PCR of six miR-183C target genes in the cornea of miR-183C KO mice (A) and in the TG of SNS-CKO vs corresponding age- and sex-matched WT controls (B). 8-12 weeks old male mice were used. *: p<0.05; **: p<0.01

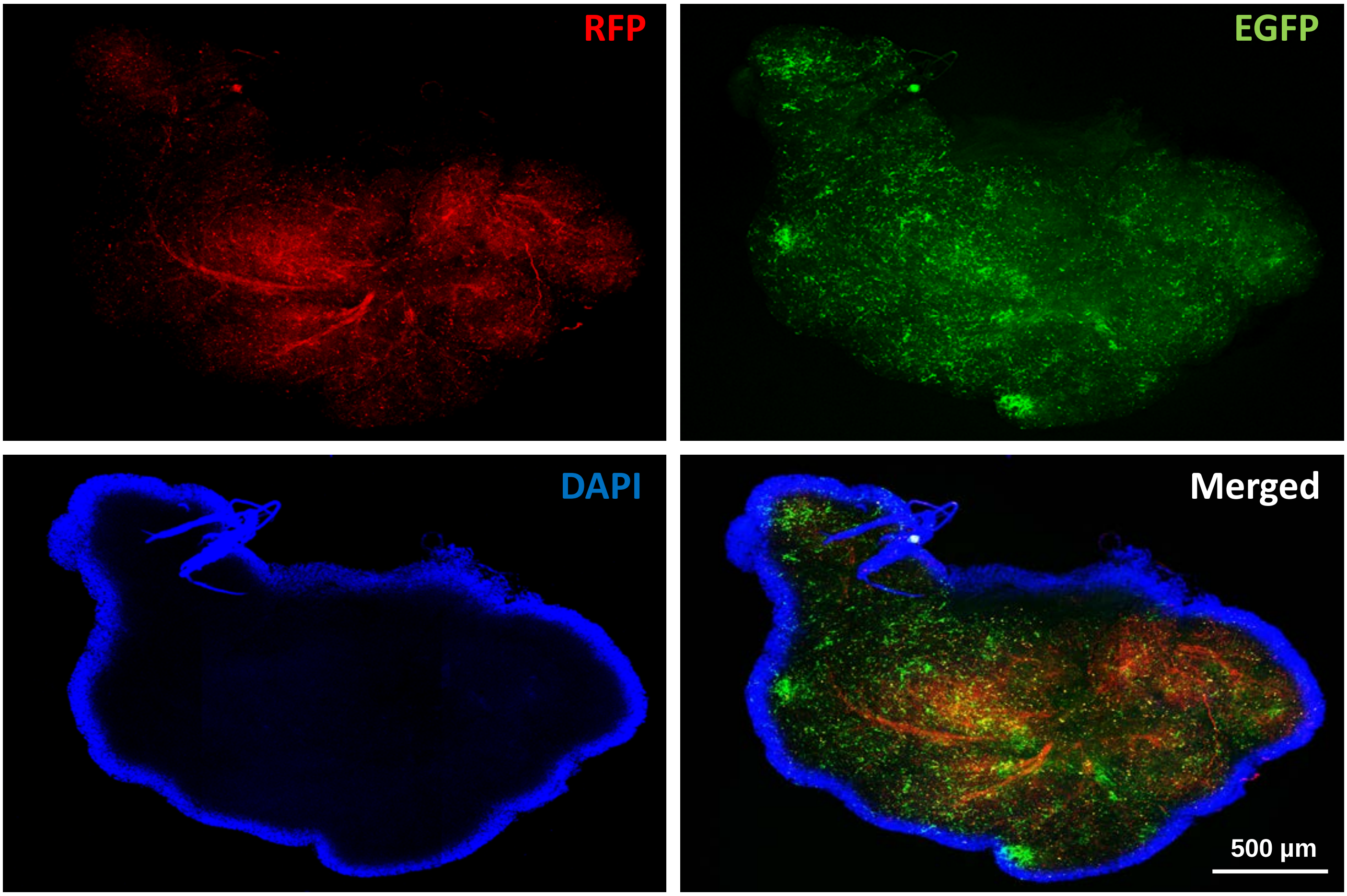

Figure S6. Confocal microscopy of the lacrimal gland of a naïve 8-week old, male miR-183C SNS-CKO mouse demonstrating Nav1.8+ sensory nerves (RFP), resident myeloid cells (Csf1r-EGFP+).

Table S1. Differentially expressed genes in the cornea of SNS-CKO vs age- and sex-matched WT control mice.

	log2FC	logCPM	LR	PValue	FDR	WT1	WT2	WT3	WT4	SNS-CKO1	SNS-CKO2	SNS-CKO3
**Upregulated genes**												
Alox12e	5.744557	1.895268	8.708509	0.003167282	1	0	0	0	0	11	2	2
Map6d1	5.393847	1.196294	17.44917	2.95093E-05	0.176938	0	0	0	0	3	4	6
Krt33a	5.206359	-0.53559	6.651936	0.009904815	1	0	0	0	0	0	6	5
Gm12250	5.038457	0.426501	7.784959	0.0052683	1	0	0	0	0	0	5	5
Bsn	4.96783	1.965668	12.01708	0.000527152	0.629451	0	0	0	0	8	0	1
Gm4491	4.854643	-0.43549	7.167472	0.007423732	1	0	0	0	0	0	4	5
1700124L1	4.804593	0.070478	10.92271	0.000949925	0.68349	0	0	0	0	1	5	2
LOC10816	4.792791	-0.038804	8.413856	0.003723721	1	0	0	0	0	0	5	3
Gm10145	4.651099	-0.408357	3.854262	0.049619759	1	0	0	0	0	0	6	0
Pcdhb4	4.623123	0.605903	10.74319	0.001046637	0.719982	0	0	0	0	2	0	6
Gm31054	4.600008	-0.413184	4.166684	0.041226418	1	0	0	0	0	2	4	0
Gm35189	4.598662	-0.111219	5.734792	0.016632037	1	0	0	0	0	1	5	0
Lta	4.578327	-0.481214	3.955598	0.046715705	1	0	0	0	0	3	3	0
AI607873	4.564751	0.134738	7.07993	0.007795206	1	0	0	0	0	1	5	0
Fer1l6	4.560222	-0.527156	5.400802	0.020127498	1	0	0	0	0	3	3	0
4933405L1	4.553783	-0.82898	3.979162	0.046066468	1	0	0	0	0	4	2	0
Tssk2	4.545363	-0.322582	6.060421	0.013824473	1	0	0	0	0	2	2	3
A3galt2	4.54153	3.993757	11.48057	0.000703275	0.632525	0	0	0	0	6	0	1
Cnr1	4.535296	3.569055	10.9956	0.000913283	0.679882	0	0	0	0	1	3	3
Ptprt	4.530924	5.136015	11.63198	0.000648275	0.632525	0	0	0	0	3	2	2
Plk3	4.519882	1.091222	9.806614	0.001738854	0.919956	0	0	0	0	4	0	3
Cct8l1	4.504517	-0.703861	3.971084	0.046287962	1	0	0	0	0	6	0	0
Gm36569	4.467852	-0.774805	4.773009	0.028909254	1	0	0	0	0	3	2	1
Gm35466	4.466101	0.541478	8.51455	0.003523181	1	0	0	0	0	3	0	4
Gm36482	4.405166	0.200164	7.151246	0.007491193	1	0	0	0	0	0	4	2
Gm38918	4.388242	-0.722148	3.910592	0.047982698	1	0	0	0	0	0	5	0
Asb17os	4.369891	-0.085744	4.766244	0.029023082	1	0	0	0	0	0	5	0
Gm31613	4.355823	-0.771534	4.192121	0.040612246	1	0	0	0	0	1	4	0
Gm40515	4.338819	-0.743056	4.083312	0.043308691	1	0	0	0	0	0	0	8
9530080O	4.338708	-0.429781	4.302277	0.038061384	1	0	0	0	0	4	0	2
BB283400	4.338654	-0.185832	4.860435	0.027479409	1	0	0	0	0	4	0	2
Gm36139	4.335562	0.352401	5.86347	0.015458285	1	0	0	0	0	0	5	0
A930017K	4.32587	-0.114393	6.097008	0.013541094	1	0	0	0	0	0	5	0
Gm34061	4.308417	-0.156284	5.537291	0.01861538	1	0	0	0	0	0	1	6
Gm4632	4.302383	-0.67111	4.119027	0.042403462	1	0	0	0	0	3	2	0
4930443O	4.283508	-0.605242	5.504857	0.018963734	1	0	0	0	0	2	1	3
Gm38615	4.26878	0.043245	6.785908	0.009188026	1	0	0	0	0	1	4	0
Gm40520	4.268254	-0.886897	4.017815	0.045022015	1	0	0	0	0	0	4	1
Tmprss12	4.243762	-0.579083	5.281516	0.021552979	1	0	0	0	0	0	4	1
Klhl6	4.218319	0.3796	5.920856	0.014962691	1	0	0	0	0	5	0	0
Oxt	4.213641	-0.459048	5.980759	0.014462777	1	0	0	0	0	1	1	4
E030042O	4.206104	-0.164046	6.53715	0.010564447	1	0	0	0	0	2	0	4
1700057H	4.199506	-0.755279	4.946305	0.026146497	1	0	0	0	0	2	2	1
Gm40835	4.199153	-0.42061	4.923669	0.026491207	1	0	0	0	0	1	1	4
5033417F2	4.18175	0.448342	7.041082	0.007966088	1	0	0	0	0	5	0	0
Gm16168	4.172342	1.504658	7.926606	0.004871308	1	0	0	0	0	0	5	0
Colq	4.143395	1.719034	7.811403	0.005191757	1	0	0	0	0	4	1	0
Gm33622	4.108331	-0.10624	6.787702	0.009178797	1	0	0	0	0	0	3	2
Gm33787	4.108168	-0.832368	5.251361	0.021929606	1	0	0	0	0	1	2	2
Kcnh5	4.106478	1.675033	7.513322	0.006124431	1	0	0	0	0	4	0	1
Gm26871	4.106259	3.99989	8.219217	0.004144907	1	0	0	0	0	1	4	0
Nlgn1	4.07725	3.576275	8.334873	0.003889119	1	0	0	0	0	1	4	0
Gm42105	4.066364	0.614005	7.039444	0.00797338	1	0	0	0	0	3	0	2
Gm32725	4.064916	-0.326632	3.844676	0.049904155	1	0	0	0	0	0	4	0
Gm41665	4.064003	-0.138997	6.192383	0.012830133	1	0	0	0	0	3	0	2
Gm8369	4.027311	-0.652544	4.029073	0.044722519	1	0	0	0	0	1	3	0
A330102I1	4.015847	2.562897	7.791544	0.005249133	1	0	0	0	0	0	0	5
F420015M	3.979298	0.076942	6.748129	0.009384607	1	0	0	0	0	0	1	4
Gm33037	3.971179	-0.889015	4.801257	0.02843898	1	0	0	0	0	2	0	3
Gm30938	3.952387	0.182705	5.800756	0.016019284	1	0	0	0	0	0	4	0
Fer1l5	3.948356	0.47178	6.317916	0.011952402	1	0	0	0	0	1	0	4
Igfbpl1	3.932841	-0.040255	5.772514	0.016278726	1	0	0	0	0	1	3	0
Dnah9	3.923767	-0.166202	4.445617	0.034990953	1	0	0	0	0	4	0	0
Gm8615	3.914328	-0.573253	4.799392	0.028469787	1	0	0	0	0	4	0	0
A230052G	3.913774	0.609562	6.102117	0.013502001	1	0	0	0	0	0	4	0
3830403N	3.894174	-0.673578	5.034889	0.024841682	1	0	0	0	0	1	0	4
Thpo	3.89381	-0.359214	4.219753	0.039955983	1	0	0	0	0	0	1	4
Gm26908	3.881896	-0.272107	6.033974	0.014033102	1	0	0	0	0	0	0	5
Gm42312	3.854632	-0.507917	4.087092	0.043211926	1	0	0	0	0	2	1	1
Gm11454	3.841327	1.209212	6.359561	0.011675039	1	0	0	0	0	2	2	0
LOC10524	3.825534	1.396114	6.49229	0.010834331	1	0	0	0	0	1	3	0

Arhgef15	3.79753	1.403113	5.923182	0.014942949	1	0	0	0	0	3	0	1
Amer3	3.796936	2.644347	6.617686	0.010097096	1	0	0	0	0	0	4	0
Nell2	3.796099	2.80924	6.604533	0.010171946	1	0	0	0	0	1	3	0
Gm34403	3.771931	-0.812694	5.196747	0.022629194	1	0	0	0	0	0	2	2
Ncf4	3.769818	-0.304105	4.144931	0.041759353	1	0	0	0	0	0	0	5
Endou	3.769396	0.505831	5.734012	0.016639425	1	0	0	0	0	0	2	2
Gm29485	3.749456	-0.446947	4.586711	0.032220789	1	0	0	0	0	1	1	2
Sec14l5	3.736502	2.944059	6.428949	0.011227502	1	0	0	0	0	0	0	4
Oas1f	3.72608	-0.814972	4.666736	0.030752317	1	0	0	0	0	2	0	2
Prr32	3.723024	-0.541392	4.456201	0.034774751	1	0	0	0	0	2	0	2
Gm33248	3.662406	-0.065116	4.565253	0.032626858	1	0	0	0	1	10	0	2
Gm40586	3.64722	0.084761	4.190835	0.040643064	1	0	0	0	0	0	1	3
LOC10816:	3.638919	-0.503654	4.593127	0.032100407	1	0	0	0	0	1	0	3
Bank1	3.621975	0.897016	5.279415	0.021578995	1	0	0	0	0	0	0	4
Gm41611	3.616353	0.630983	5.216869	0.022368784	1	0	0	0	0	0	0	4
LOC10816:	3.612206	-0.727699	3.957326	0.046667776	1	0	0	0	0	1	0	3
Cd37	3.546553	0.033408	4.304032	0.038022122	1	0	0	0	0	1	2	0
Ngf	3.531348	-0.282098	4.17831	0.0409445	1	0	0	0	0	0	0	4
Gm41561	3.531185	-0.561138	4.17612	0.04099745	1	0	0	0	0	0	0	4
Gm32315	3.516864	-0.181754	3.929662	0.047441488	1	0	0	0	0	3	0	0
Gm35312	3.51571	-0.246059	3.951413	0.046832022	1	0	0	0	0	3	0	0
Stmnd1	3.514988	-0.708603	4.339578	0.037236146	1	0	0	0	0	2	1	0
Gm5486	3.488931	0.11353	4.555973	0.032804113	1	0	0	0	0	2	1	0
Gm36082	3.48382	0.447698	4.406433	0.035803638	1	0	0	0	0	3	0	0
Gm40647	3.46649	1.500094	4.779108	0.028807044	1	0	0	0	0	1	2	0
Lrguk	3.459668	1.338756	4.856081	0.027548838	1	0	0	0	0	0	3	0
Hspa12b	3.455881	0.680033	4.659324	0.030885351	1	0	0	0	0	3	0	0
Mgat4c	3.444737	2.063534	4.860388	0.027480149	1	0	0	0	0	1	2	0
Ikzf1	3.444717	1.486266	4.737584	0.02951049	1	0	0	0	0	3	0	0
D630003M	3.441893	1.472033	4.823453	0.028075047	1	0	0	0	0	2	1	0
Gm30363	3.43696	0.560761	4.916763	0.02659731	1	0	0	0	0	0	3	0
Cxcl10	3.434563	0.973066	4.800433	0.028452593	1	0	0	0	0	3	0	0
Cntnap5b	3.434518	1.353137	4.89403	0.026949707	1	0	0	0	0	1	2	0
Stx1b	3.432291	5.57315	11.96398	0.000542388	0.629451	0	1	0	0	3	6	2
LOC10263	3.430924	-0.907512	3.938893	0.047181816	1	0	0	0	0	1	1	1
Mmp17	3.428427	1.830258	4.835108	0.027885896	1	0	0	0	0	3	0	0
LOC10263:	3.41783	0.475805	10.07881	0.001499842	0.884563	1	1	0	1	13	8	3
Trp53cor1	3.409102	1.329926	4.772828	0.028912291	1	0	0	0	0	0	2	1
B3gnt5	3.409028	2.607441	4.952026	0.026060112	1	0	0	0	0	2	1	0
Nanos3	3.401309	0.161121	4.291655	0.038299867	1	0	0	0	0	2	0	1
Pde10a	3.399045	2.695419	4.890128	0.027010681	1	0	0	0	0	0	2	1
Mfsd2b	3.398817	0.480283	4.481607	0.034261486	1	0	0	0	0	2	0	1
B3galt1	3.388696	5.062203	4.986901	0.025539911	1	0	0	0	0	1	1	1
Slc7a14	3.387554	5.47207	5.005009	0.025274066	1	0	0	0	0	1	1	1
Tmem233	3.383453	5.293149	4.976878	0.025688299	1	0	0	0	0	0	1	2
Trpa1	3.380646	3.587977	4.933464	0.026341471	1	0	0	0	0	0	1	2
Apc2	3.372017	4.320179	4.92707	0.026439115	1	0	0	0	0	0	0	3
Galnt16	3.368704	3.440185	4.904921	0.026780283	1	0	0	0	0	0	0	3
Tldc2	3.334304	0.967186	4.310253	0.037883326	1	0	0	0	0	1	0	2
Scrg1	3.329266	0.868509	4.63084	0.031402192	1	0	0	0	0	0	0	3
Gm34489	3.310411	-0.652716	3.902505	0.048214167	1	0	0	0	0	1	0	2
Slc9a3	3.277828	0.284198	4.242805	0.039417002	1	0	0	0	0	0	0	3
Itga10	3.259853	0.709244	14.07303	0.000175847	0.486636	0	1	0	2	3	14	5
Gm40207	3.245348	-0.572539	3.977911	0.046100708	1	0	0	0	0	0	0	3
Dubr	3.233191	3.33905	9.407239	0.002161304	1	1	0	0	0	4	1	5
LOC10816!	3.145839	0.112797	6.279622	0.012213418	1	1	0	0	0	4	0	6
A230103J1	3.102962	0.437967	5.071359	0.024324303	1	1	0	0	0	1	6	1
LOC10524(	3.098762	-0.217429	5.543584	0.018548557	1	0	1	0	0	2	2	5
Gm10369	3.073928	0.101199	4.043939	0.044330236	1	0	1	0	0	0	3	6
Gm41283	3.062227	1.133226	5.829611	0.015758608	1	1	0	0	0	3	4	1
Gm13032	3.026761	-0.457513	5.607958	0.017879083	1	0	1	0	0	1	4	3
Hist1h2af	2.977426	-0.725121	6.762213	0.009310823	1	0	1	0	0	3	2	3
Neurl3	2.96028	-0.031128	4.17447	0.041037399	1	0	0	0	1	6	1	0
Gm13383	2.845092	-0.462837	3.849483	0.049761326	1	1	0	0	0	0	3	5
Clgn	2.771918	6.318102	6.672551	0.009790895	1	0	0	0	1	0	5	2
Rbm11	2.767744	4.61677	6.70261	0.00962719	1	0	0	1	0	4	2	1
Ccdc146	2.753038	0.123495	3.994717	0.045643114	1	0	0	0	1	0	3	4
Tmem8	2.746229	4.489702	6.571632	0.010361687	1	1	0	0	0	1	1	5
Ceacam10	2.738624	0.68087	5.746941	0.016517393	1	0	1	0	0	3	0	4
Trpv1	2.732715	3.886204	6.314237	0.011977225	1	0	0	1	0	0	2	5
1700024P1	2.705243	0.165624	4.672368	0.030651629	1	0	0	0	1	2	4	0
Gm10037	2.65408	0.277629	4.436123	0.035186065	1	0	0	0	1	2	3	1
Vash1	2.548649	5.281637	5.393488	0.020212034	1	0	0	1	0	0	4	2
Lrrc16b	2.515554	3.812149	5.081573	0.024181426	1	0	1	0	0	0	0	6

Casp1	2.448184	1.703573	5.514702	0.018857287	1	2	0	0	0	6	3	0
Erdr1	2.433583	1.227452	4.725655	0.029715872	1	0	7	1	7	13	0	57
5430431A1	2.390039	0.53371	5.662808	0.01732836	1	1	0	0	2	2	1	12
Rbfox3	2.350032	7.779519	6.851179	0.008858279	1	1	0	0	1	3	6	0
LOC10816E	2.347042	-0.04942	4.24949	0.039262128	1	0	0	1	2	1	2	12
Apba1	2.338504	7.011977	6.798444	0.009123735	1	0	0	0	2	1	4	4
Stard6	2.332403	0.606954	4.256898	0.039091232	1	1	0	0	1	3	2	4
Gm17399	2.310033	0.709472	4.208644	0.040218484	1	0	0	1	2	1	3	10
Diras1	2.304331	6.615922	4.054499	0.044053781	1	0	0	0	1	3	1	1
P2rx6	2.303249	4.027186	4.016282	0.04506295	1	0	1	0	0	1	2	2
Ccdc74a	2.284994	4.189504	3.965535	0.046440772	1	0	0	1	0	0	1	4
Nfkbid	2.25879	0.308962	3.852889	0.049660383	1	0	0	0	2	5	1	2
Lyve1	2.226739	1.715069	4.251924	0.039205898	1	0	0	2	0	8	0	0
Foxn1	2.197128	-0.192117	4.16111	0.041362281	1	2	1	1	0	10	3	1
Lrrc32	2.152644	0.869213	4.435365	0.035201694	1	1	0	2	0	1	5	6
Gm32462	2.145036	0.099154	4.485217	0.034189206	1	2	0	0	1	5	0	7
Kcng2	2.079045	1.986629	4.178185	0.040947529	1	1	0	0	1	1	6	0
Il15	2.066023	1.067528	8.780174	0.003045221	1	4	3	0	3	5	7	23
Neil2	2.065633	1.99628	4.066288	0.043747315	1	1	0	1	0	2	0	6
Ccdc177	2.058998	1.829891	4.827421	0.028010507	1	0	3	0	0	3	0	8
Gper1	2.058236	0.405049	4.302319	0.038060448	1	0	0	3	0	2	2	8
Mrc1	2.054748	3.547076	7.319689	0.006820322	1	0	4	0	0	3	1	10
Mum1l1	2.021368	1.028288	3.986917	0.045854881	1	1	2	0	0	4	2	4
Mkx	2.008812	2.295658	5.451559	0.019550872	1	1	1	0	1	5	2	3
Sptbn4	1.997318	3.954989	4.384598	0.036265044	1	1	0	0	1	4	2	1
Pcsk2os1	1.995741	1.901172	3.998537	0.045539781	1	0	1	1	0	0	4	3
Serpind1	1.978245	3.530247	4.242462	0.039424944	1	2	0	0	0	2	1	4
Adam11	1.977587	5.858257	4.328561	0.037477921	1	1	0	0	1	1	0	6
Gm15663	1.96812	1.589493	5.034369	0.02484914	1	1	0	1	3	6	3	7
Radil	1.92603	1.986357	4.380778	0.036346408	1	1	0	2	0	3	2	5
Calhm2	1.88592	1.505015	9.237378	0.002371243	1	2	5	3	3	9	8	22
Rasl10b	1.859218	4.316665	6.320775	0.011933144	1	3	0	0	1	5	0	7
Zfp423	1.842787	4.007499	15.12183	0.000100791	0.302172	1	3	8	0	14	6	14
Ggt7	1.839568	3.304212	4.380395	0.036354571	1	0	2	1	0	3	2	4
Gm7120	1.82645	2.278979	6.89876	0.008625553	1	4	3	0	3	9	1	20
Nipa1	1.824424	3.432074	13.15431	0.000286859	0.509334	4	4	2	4	16	5	18
Gjc1	1.820244	2.369408	4.264216	0.038923215	1	1	0	0	2	3	1	5
Gm31686	1.816295	0.185901	3.951946	0.046817189	1	1	3	1	3	11	5	4
Zscan18	1.814652	1.879812	4.109741	0.042636887	1	2	0	1	1	3	6	2
Gria2	1.782612	4.818001	10.86	0.000982636	0.693163	2	5	2	0	7	1	17
Slc7a11	1.77667	2.067259	5.055935	0.024541731	1	0	4	1	0	1	1	13
6330549D2	1.768939	1.345885	6.946931	0.008396318	1	2	8	3	1	5	19	10
Ebf1	1.75531	7.425499	13.00014	0.000311467	0.509334	4	5	2	0	10	8	11
Cldn19	1.748	6.710272	5.378835	0.020382484	1	0	1	2	1	5	2	4
Ncf1	1.743232	1.675562	4.190254	0.040657007	1	1	0	3	0	4	4	3
Snap91	1.741611	6.158616	5.424572	0.019855302	1	1	2	0	1	2	1	8
Gm14322	1.716397	1.851711	8.79543	0.003019861	1	2	5	3	8	7	11	30
P4ha3	1.664026	3.505528	4.760009	0.029128392	1	0	1	1	4	4	8	2
2410131K1	1.663336	3.114597	15.48964	8.29588E-05	0.298452	9	6	3	7	20	15	26
B230307C2	1.6329	1.610812	5.149057	0.023259001	1	9	9	1	7	12	19	30
Fat3	1.629068	4.882104	4.327546	0.037500283	1	2	0	0	2	6	2	2
Slc1a6	1.607899	2.114425	6.144413	0.013182794	1	0	3	4	4	9	12	3
Gm41491	1.605604	1.139565	5.894333	0.015189685	1	6	4	2	9	12	16	20
Jph1	1.60426	2.280723	5.134151	0.023459562	1	3	4	1	2	3	13	6
Atg4d	1.570049	0.960032	4.646007	0.031125866	1	1	5	1	9	11	3	24
Kcnn1	1.551288	3.590368	5.734146	0.016638155	1	1	3	4	0	6	8	4
Igf2bp3	1.536261	1.704858	4.160128	0.041386277	1	3	1	1	7	10	7	9
Klhdc9	1.533501	1.805518	6.677713	0.009762581	1	2	0	5	5	8	10	9
9330151L1	1.525604	1.827966	4.825988	0.028033799	1	2	6	0	1	5	5	10
Snord104	1.523003	1.254315	4.561598	0.032696546	1	5	3	0	5	8	3	20
Podxl2	1.519355	2.889246	3.929809	0.047437332	1	3	2	0	0	0	3	9
Dusp18	1.482326	3.470024	6.890767	0.008664209	1	6	1	3	1	9	3	13
Kcnmb3	1.441188	1.386567	3.993284	0.045681952	1	6	8	3	0	6	13	17
Ccdc38	1.422929	1.385634	5.230998	0.022187794	1	6	1	4	11	19	12	13
Ercc1	1.3595	2.133134	5.752027	0.016469643	1	3	9	2	6	10	12	16
Prkg2	1.357261	4.378489	5.863957	0.01545401	1	2	3	0	5	4	6	10
Gm527	1.353778	1.680453	4.298308	0.03815032	1	5	2	0	1	6	4	6
Neu2	1.347199	1.5253	8.406358	0.003739109	1	9	11	2	13	14	25	26
Il18bp	1.343387	1.101893	4.66958	0.03070143	1	2	6	3	7	8	8	20
Plscr1	1.33333	1.176525	4.018899	0.04499308	1	3	6	6	10	11	13	26
Tmem17	1.326181	2.655171	5.914732	0.015014789	1	0	14	4	9	16	17	14
Lysmd2	1.321246	4.630641	9.955585	0.001603621	0.88998	4	8	0	6	7	7	21
Cacna2d3	1.317245	4.677597	7.020958	0.00805611	1	4	3	6	1	6	12	9
Rab40b	1.310456	1.630487	4.196329	0.040511592	1	3	8	1	3	7	8	13

Klhl13	1.296232	4.035675	11.50008	0.000695934	0.632525	6	11	4	5	19	9	21
Amot	1.255365	2.482666	3.898514	0.048328839	1	3	6	0	3	6	5	11
Hs3st1	1.246133	2.601967	6.685221	0.009721547	1	4	11	3	2	10	14	11
Pdgfc	1.245231	2.02222	3.867677	0.049224601	1	5	7	3	5	16	4	17
Dbn1	1.239039	5.360596	5.978499	0.014481325	1	6	3	6	1	9	4	18
Eepd1	1.229643	5.537117	6.226052	0.01258841	1	0	7	2	4	1	5	18
Lpcat2	1.219071	2.264138	6.145593	0.013173996	1	12	4	7	11	19	19	23
Cyp2d22	1.216971	2.7193	3.881779	0.048812797	1	4	1	4	3	9	5	8
St3gal6	1.216751	4.256527	5.914093	0.015020236	1	4	2	0	7	4	2	18
Lancl3	1.206817	3.20783	4.435644	0.035195949	1	4	2	4	1	7	5	8
Gstm6	1.199012	1.441401	4.584651	0.032259539	1	4	11	0	11	10	16	16
Kcnip3	1.179971	6.230212	5.633461	0.017620805	1	2	5	3	3	8	3	12
Bves	1.17994	2.460761	5.939885	0.014801984	1	8	18	4	10	12	11	51
Ust	1.152655	4.47925	4.477644	0.034341027	1	5	4	2	0	5	7	7
Zxdb	1.124193	2.825844	7.036899	0.007984718	1	12	20	19	16	36	32	44
4831440E1	1.122879	2.200511	5.337832	0.020867414	1	7	11	13	6	14	19	32
Slc19a1	1.116142	3.47448	6.954368	0.008361483	1	7	10	5	8	8	12	32
Zfp850	1.11539	1.665441	3.886642	0.048671655	1	3	7	7	3	12	10	11
Igdcc4	1.107145	2.637296	5.180147	0.022846389	1	6	1	10	4	10	9	18
Gm17059	1.099211	1.385467	4.156898	0.041465283	1	13	5	4	5	18	12	14
Ppp4r4	1.092939	4.268135	3.890302	0.048565682	1	2	3	1	5	6	7	5
LOC10816	1.092089	2.902805	7.623257	0.005762034	1	6	14	8	16	16	18	39
Nceh1	1.047095	5.730114	8.722294	0.003143424	1	10	8	5	5	11	8	26
Pcbd1	1.045178	3.407442	3.949447	0.046886779	1	3	6	0	8	5	11	10
Kcnd3	1.018481	4.118082	5.306453	0.021246568	1	7	4	3	10	8	10	20
Dennd4b	1.006291	3.442235	5.749232	0.01649587	1	8	11	3	6	10	13	20
Mapk10	0.999596	7.803019	7.733945	0.005419221	1	11	4	6	8	17	7	21
Ugcg	0.985433	4.522355	9.532832	0.002018285	0.981214	9	26	9	17	30	15	48
1700001L0	0.973321	3.689078	4.287488	0.038393858	1	12	16	2	4	15	9	28
Tmem86a	0.958362	3.471779	4.621113	0.031580737	1	9	10	7	5	8	19	19
Irx3	0.954685	3.931653	9.06179	0.002610064	1	29	72	23	43	51	75	114
Hacl1	0.939817	2.334452	5.076937	0.024246165	1	10	17	8	11	16	10	46
0610009B2	0.939672	3.682178	6.682931	0.009734042	1	30	61	17	42	56	51	110
Pear1	0.936035	2.301163	4.40447	0.035844867	1	2	15	6	9	16	10	20
Fam149b	0.928495	2.836587	5.315205	0.021140102	1	15	19	17	22	24	16	78
Cenpv	0.92753	3.147169	4.853903	0.02758365	1	9	18	10	8	14	14	41
Get4	0.925307	2.822492	5.36763	0.020513833	1	14	13	8	18	16	19	45
Nelfcd	0.917706	3.72935	7.479316	0.006241179	1	10	30	12	34	31	22	74
Kdelc1	0.915247	2.49792	4.436922	0.035169595	1	9	18	4	16	11	25	29
Lrfn1	0.913694	4.568803	5.047903	0.024655757	1	9	11	8	5	12	15	21
Mettl22	0.912674	3.421195	5.157705	0.02314346	1	12	4	4	9	13	12	17
Rnf225	0.902946	2.333878	6.295163	0.012106791	1	16	28	11	20	23	33	50
Zfp41	0.900799	2.452503	4.371903	0.036536176	1	12	12	9	10	10	17	38
Rimbp2	0.896033	4.617634	3.996327	0.045599535	1	7	2	2	8	17	3	7
P2rx7	0.895075	3.313621	4.29139	0.038305842	1	13	16	8	11	26	16	25
Mir703	0.890102	5.058246	7.006146	0.008123038	1	106	165	80	134	138	93	541
Bop1	0.887272	3.528752	7.107157	0.007677677	1	44	24	22	35	33	38	125
Map1s	0.86601	4.168351	5.327015	0.02099732	1	6	7	11	19	22	11	28
Zfp2	0.865868	3.261108	4.86195	0.027455287	1	12	10	6	20	17	16	35
Per2	0.865335	2.870615	4.016074	0.04506852	1	12	17	21	21	24	31	47
Iba57	0.862988	3.038767	4.033638	0.044601676	1	11	33	1	22	16	20	56
Angptl4	0.857394	3.747261	5.042304	0.024735575	1	10	15	14	25	26	19	46
Trappc2	0.846041	4.198287	4.646034	0.03112538	1	23	7	2	6	10	16	28
Tgfb1i1	0.844929	4.350356	3.936403	0.047251723	1	6	11	1	9	10	13	13
Mettl9	0.843944	4.779333	8.811324	0.002993667	1	104	109	53	92	111	100	315
Ooep	0.843219	2.755858	4.07401	0.043547789	1	25	39	20	30	46	45	61
Cryl1	0.839541	3.106454	4.423456	0.035448166	1	17	23	15	31	41	20	59
Pard6a	0.83027	3.950387	4.09112	0.04310907	1	10	22	7	15	25	15	32
Ercc8	0.828987	2.430403	4.648293	0.031084444	1	14	19	12	17	18	20	50
Sh3pxd2b	0.822605	4.558274	8.475138	0.003600331	1	9	20	14	30	18	28	54
Mtx3	0.822335	3.802158	7.465589	0.006288946	1	23	22	10	30	30	35	48
Exd2	0.816777	2.622813	4.105597	0.042741511	1	5	20	12	12	12	13	45
Syvn1	0.8157	3.443917	5.446317	0.019609626	1	32	41	21	22	43	36	82
Smyd2	0.804287	4.955644	11.21568	0.000811091	0.679882	35	36	27	40	40	47	104
Pepd	0.794618	4.45653	11.64578	0.000643482	0.632525	43	47	29	60	64	57	120
Gm33585	0.794147	2.754978	4.698295	0.030192554	1	18	23	38	47	41	55	75
Glrb	0.788022	6.118486	11.03917	0.000892067	0.679882	35	41	24	40	83	20	84
Fam136a	0.787259	3.640449	5.979142	0.014476041	1	40	57	22	28	55	43	99
Rtn4rl1	0.780179	5.014445	4.035251	0.044559049	1	7	13	3	4	7	7	22
Cygb	0.778878	6.32644	5.863405	0.015458861	1	5	25	4	10	21	11	25
Xk	0.77718	3.894724	9.007037	0.00268942	1	21	32	10	30	38	36	44
Tmem177	0.758994	2.999904	4.311513	0.037855277	1	8	14	11	16	11	13	43
Tm7sf2	0.755785	4.011984	6.166543	0.01301888	1	27	54	31	26	50	35	101
Reep1	0.755529	6.726211	5.98371	0.014438597	1	13	8	4	21	16	12	32

Usb1	0.749651	4.133819	7.932305	0.004855989	1	53	68	24	51	45	84	120
Slc35b3	0.749591	3.268317	4.825845	0.028036118	1	31	34	16	41	31	41	89
Cacnb3	0.745777	6.483171	4.7517	0.02926937	1	14	9	8	10	19	17	16
Zfp51	0.744585	2.640263	4.553732	0.032847085	1	21	31	17	24	46	22	51
Mcm4	0.743099	4.031768	7.561016	0.005964435	1	60	94	29	77	59	64	229
Scarb1	0.738694	4.343585	6.625317	0.010053925	1	31	68	49	71	66	58	171
Bet1	0.73648	3.307959	5.635416	0.017601172	1	25	35	17	29	33	30	76
Nxt1	0.732544	3.228375	4.285926	0.038429146	1	22	40	32	21	26	16	129
Pigg	0.731365	3.138817	4.150421	0.041624167	1	44	43	21	39	64	45	75
Tmem170l	0.731253	3.209245	5.54738	0.018508375	1	24	32	15	33	54	29	44
Jam3	0.73018	5.749406	5.527639	0.018718344	1	11	8	4	21	17	12	27
Fermt2	0.724987	6.322164	6.099121	0.013524912	1	13	18	26	18	27	24	47
Sdhaf4	0.721732	4.624933	7.315073	0.006837863	1	33	61	42	58	55	44	164
B4galt7	0.716121	3.732593	6.513034	0.010708662	1	29	49	27	27	46	43	78
Xylt2	0.715189	3.380212	4.311334	0.037859261	1	10	16	10	23	18	18	39
Asb1	0.714117	5.029162	5.637046	0.017584809	1	12	13	12	17	14	18	37
Atg16l2	0.707914	3.111049	4.212038	0.040138087	1	17	11	16	22	25	20	40
Ampd2	0.706658	3.74973	5.040261	0.024764757	1	20	25	8	20	14	31	46
Mapk7	0.704029	3.268333	4.380324	0.036356096	1	31	28	19	19	29	28	71
Dnajc27	0.696148	4.428801	4.273569	0.038709554	1	15	11	11	13	20	18	24
Sec13	0.69301	5.05381	5.655159	0.017404094	1	97	122	30	88	68	86	288
Ppp1cb	0.69135	6.605714	8.634378	0.003298779	1	325	467	211	386	343	282	1261
1190002N:	0.683157	4.260906	6.631865	0.010017032	1	26	27	17	19	35	20	57
Cry2	0.683066	5.084101	7.601412	0.005832262	1	27	53	54	63	57	70	121
Dus3l	0.682072	4.088797	5.119173	0.023662893	1	10	29	33	34	30	35	70
Tmed4	0.678988	5.096216	7.965825	0.004766876	1	63	82	40	63	78	76	154
Tmem9b	0.676494	6.013565	13.22452	0.000276311	0.509334	102	197	89	174	132	154	429
Dnase1l1	0.675046	3.306054	4.239651	0.039490279	1	31	57	16	28	35	33	98
Pnp	0.67349	6.182599	8.684504	0.003209269	1	332	624	176	353	537	285	1021
Ptprg	0.671251	5.348863	15.19753	9.683E-05	0.302172	74	139	81	113	113	112	289
Fam212b	0.669744	4.595499	6.421811	0.011272713	1	15	20	10	25	24	22	39
Pkia	0.668515	5.483327	6.038306	0.013998711	1	9	12	16	27	25	19	34
Uba2	0.666726	5.272134	7.299454	0.006897556	1	98	174	52	132	128	95	353
Hs1bp3	0.665304	3.835239	4.088001	0.04318869	1	50	43	39	57	63	38	150
Dusp1	0.665225	4.488691	4.223672	0.039863804	1	101	132	39	79	164	46	226
Zdhhc2	0.655737	7.747822	4.592577	0.032110698	1	7	15	5	19	14	8	34
Rmdn2	0.655701	3.719083	3.93835	0.047197042	1	9	9	10	20	15	8	37
Dnaja3	0.651123	4.271631	5.662376	0.017332626	1	42	78	32	50	64	53	129
Rarres1	0.650298	4.81164	5.351376	0.020705926	1	28	32	17	29	43	31	52
Sdhc	0.650291	5.647094	11.57408	0.000668776	0.632525	67	89	47	93	93	62	216
Appbp2	0.648186	4.807111	7.315369	0.006836737	1	70	117	63	96	114	75	245
Oard1	0.647204	3.959574	9.718216	0.001824505	0.937691	61	57	36	60	78	68	110
Mfn1	0.646542	5.125044	16.17421	5.77753E-05	0.230947	77	118	54	106	127	81	223
Bin3	0.644705	3.29124	4.36749	0.036630914	1	22	42	17	28	31	39	58
Pxylp1	0.642384	4.107915	4.233095	0.039643082	1	78	93	51	72	66	82	229
Psma5	0.63805	5.202508	7.135654	0.007556608	1	69	99	35	92	88	77	190
Psmb3	0.63439	7.012351	5.386381	0.020294515	1	195	365	211	375	233	237	1041
Mzt1	0.628622	3.552345	4.887192	0.027056659	1	23	38	13	34	27	22	84
Tdrp	0.626582	3.793607	5.3142	0.021152307	1	27	41	22	48	58	25	82
Cct4	0.622865	6.102901	7.233703	0.007154747	1	196	274	126	274	238	181	672
Nfs1	0.621898	4.077508	5.046765	0.02467196	1	25	66	30	63	64	40	114
Tbc1d14	0.621788	4.082381	7.502638	0.00616087	1	57	75	32	47	64	68	116
Zfp141	0.620152	2.99654	4.230816	0.039696352	1	29	33	15	19	28	24	65
Mtx2	0.619201	4.565987	7.237771	0.007138554	1	50	92	44	66	73	59	175
Esrra	0.616926	4.152027	5.615373	0.017803594	1	47	86	42	91	72	75	169
Ptrhd1	0.614063	3.975123	4.987576	0.025529954	1	16	34	21	31	31	28	62
Hdac11	0.606785	5.022128	6.11249	0.013422992	1	12	19	23	40	34	26	50
Wtip	0.60617	3.879157	5.20444	0.022529274	1	60	45	29	47	44	48	133
Yod1	0.604726	3.950145	4.362222	0.036744358	1	33	65	37	69	36	45	177
Vhl	0.599194	4.177006	4.466059	0.034574655	1	32	73	42	82	76	60	129
Cln5	0.597608	4.511008	6.263452	0.012325385	1	45	80	36	66	75	72	109
Sash1	0.596352	5.465516	12.00684	0.000530055	0.629451	53	73	45	62	84	58	130
Efna1	0.593056	4.575163	4.827704	0.028005909	1	77	92	41	123	76	73	259
Pmp22	0.591192	10.03476	7.417894	0.006457835	1	56	104	57	50	63	65	199
Nubp2	0.590401	4.734688	6.637468	0.009985578	1	43	54	24	58	53	44	113
Sec14l1	0.589505	5.019232	8.530874	0.00349172	1	40	80	45	86	83	66	140
Tppp3	0.585622	9.779929	9.496583	0.002058549	0.987445	62	101	46	132	109	104	168
Peg13	0.585394	6.360433	4.627168	0.031469474	1	15	13	17	25	37	15	28
Cirh1a	0.585353	4.210785	5.002358	0.025312815	1	51	60	42	81	70	66	138
Notch2	0.577907	5.103559	6.534264	0.010581601	1	137	147	78	168	136	141	353
Rasa3	0.577108	4.081705	4.371166	0.036551972	1	25	45	19	19	35	31	57
Gas2l1	0.574035	4.816108	5.947944	0.014734459	1	60	65	34	54	74	52	121
Slc36a1	0.570858	6.833301	5.659741	0.017358684	1	23	31	24	13	27	16	64
Card19	0.570793	6.016134	6.66936	0.009808443	1	180	249	109	227	228	159	520

Dcun1d5	0.568193	5.399288	10.53026	0.001174355	0.75068	107	149	75	136	137	121	282
Nip7	0.56774	4.073709	4.604596	0.031886359	1	37	58	39	66	68	55	104
Taf5l	0.566047	4.378501	6.544977	0.01051807	1	65	103	55	101	89	84	203
Rbp1	0.564892	6.226507	6.244511	0.012457875	1	272	537	183	295	328	272	924
Gnai3	0.562814	5.516361	7.065488	0.007858292	1	184	330	149	243	227	231	602
Tpp1	0.562464	5.910603	11.10389	0.000861467	0.679882	185	244	121	224	233	155	531
Cbfb	0.55779	6.509976	6.864915	0.008790444	1	116	150	84	125	124	105	342
Hes1	0.552941	7.753112	8.201101	0.004186498	1	1023	1560	703	1337	1326	1097	2916
Aco2	0.552437	5.217303	5.426073	0.01983824	1	64	79	48	70	72	68	164
Apex1	0.546307	4.717414	7.298622	0.006900752	1	66	107	49	75	69	91	176
Mcc	0.542328	3.662587	4.658824	0.030894339	1	51	82	35	47	68	57	115
Cfap20	0.541337	4.518032	5.68847	0.017076737	1	59	60	36	80	65	54	153
Mpv17	0.539437	5.615272	11.75874	0.000605581	0.632525	75	117	63	125	111	97	219
Arnt	0.538576	4.592783	5.102395	0.023892834	1	114	122	70	78	99	88	273
Pdcd6	0.532905	5.892531	8.927656	0.002808846	1	176	314	159	275	231	225	606
Seh1l	0.532326	4.30801	4.692155	0.03030063	1	81	94	40	60	83	57	178
1110051M	0.527644	5.212519	4.376194	0.036444284	1	24	17	15	34	25	36	37
Znhit2	0.526667	4.733295	6.396318	0.011435728	1	41	66	44	90	72	60	137
Pdhb	0.52588	5.955593	5.762441	0.016372315	1	88	143	51	124	93	117	238
Uckl1	0.525014	4.490461	4.001796	0.045451799	1	63	151	52	103	78	91	247
Fbxo4	0.52349	4.673573	4.968234	0.025816988	1	98	104	101	94	101	100	276
Wfdc2	0.522775	5.527928	6.615338	0.010110415	1	235	357	125	333	362	218	566
Fzd6	0.521218	5.444109	5.004159	0.025286481	1	217	308	168	305	234	228	705
Ctps	0.519699	4.375658	3.993394	0.045678972	1	41	71	34	47	54	50	111
Ak1	0.519288	5.87956	5.787069	0.016144482	1	54	111	37	56	83	63	134
Iqcb1	0.516427	4.073414	4.199433	0.040437509	1	30	52	38	55	48	57	86
Dgcr2	0.515802	5.443384	7.564705	0.00595224	1	114	172	93	200	149	139	365
G3bp1	0.513917	7.536123	16.88812	3.9649E-05	0.203773	699	1196	542	999	886	849	2112
Pitx2	0.509343	3.989668	7.580369	0.005900736	1	87	109	66	106	107	95	207
Cyth1	0.508675	4.90596	5.096448	0.023974887	1	93	146	51	133	120	101	240
Gabarapl2	0.507126	6.068137	4.385304	0.036250029	1	69	111	60	119	84	50	295
Klhl12	0.506696	4.526038	4.886647	0.027065202	1	40	77	41	62	47	50	154
Aacs	0.506236	5.680819	7.200983	0.007286368	1	169	224	133	152	192	147	444
Cx3cl1	0.504743	4.23381	5.456129	0.019499804	1	73	81	47	106	79	79	184
Anxa7	0.503123	6.881612	7.666478	0.005625616	1	338	575	299	467	357	385	1206
Tomm20	0.502438	5.535423	6.323953	0.011911779	1	58	57	49	71	83	52	126
Trex2	0.501035	4.823577	5.417413	0.019936878	1	106	223	101	173	172	135	358
Gprc5c	0.500021	5.2471	3.905372	0.048131979	1	35	52	25	32	41	56	53
Rac1	0.498464	8.117123	9.921526	0.001633569	0.88998	761	1225	644	1106	1075	751	2414
Anxa3	0.497979	4.04637	4.189782	0.040668331	1	66	113	73	85	124	64	188
Eef1akmt1	0.496836	4.416637	4.367013	0.036641163	1	55	50	31	73	56	52	124
AW549877	0.49412	4.832544	4.186188	0.040754646	1	69	122	59	109	101	98	186
4833420G1	0.492252	4.529903	4.00656	0.045323547	1	79	94	38	74	88	64	160
Kdelr2	0.4893	5.674421	3.962209	0.046532598	1	172	248	110	141	166	142	460
Limk1	0.488633	6.666855	4.552342	0.032873743	1	13	40	25	31	38	20	60
Mmadhc	0.487065	6.072032	5.009563	0.025207666	1	217	315	168	284	210	206	726
Rtn1	0.484575	9.020087	4.434603	0.03521741	1	23	30	23	38	31	40	50
Cul2	0.484417	5.084036	5.518943	0.018811617	1	60	62	53	75	67	85	116
Sertad1	0.48437	5.106047	8.932128	0.002801977	1	113	158	76	192	154	142	278
Vps29	0.482942	5.892887	4.317246	0.037727936	1	113	159	99	107	114	101	340
Fundc1	0.481889	5.865674	7.035769	0.007989756	1	112	107	74	116	116	95	245
4930453N2	0.480606	5.162608	6.855438	0.008837187	1	136	173	83	199	174	136	329
Znrd1	0.479766	4.488576	4.066225	0.043748954	1	52	91	71	96	89	64	196
Fth1	0.47965	10.45482	5.407726	0.020047819	1	2461	3659	1984	2726	2633	1972	8113
Sowahb	0.47809	4.212724	6.446624	0.011116336	1	91	127	81	133	134	107	225
Derl2	0.47744	4.881065	4.524174	0.033419199	1	103	126	54	105	127	66	236
Bloc1s4	0.473351	4.29503	5.544392	0.01854	1	67	100	33	75	81	77	128
Gna14	0.472069	4.955451	5.775292	0.016253018	1	41	55	45	78	92	53	83
Agps	0.471887	5.073022	4.511272	0.033672197	1	121	159	65	140	133	116	273
Mrpl3	0.470619	4.868666	4.149258	0.041652779	1	87	97	50	102	84	79	207
Stx3	0.469752	5.012492	5.099406	0.023934043	1	138	170	95	196	151	164	329
Idh1	0.468406	6.195179	5.208982	0.022470485	1	194	302	150	228	246	154	584
Sema4a	0.468249	5.95613	6.356405	0.011695831	1	271	408	219	363	311	272	828
Arf4	0.467742	6.187378	5.797137	0.016052291	1	235	351	204	363	292	240	760
Ptk2	0.467044	4.564646	4.096489	0.042972369	1	64	101	39	55	63	68	147
Psat1	0.465838	5.576929	6.161963	0.013052625	1	132	256	115	180	197	130	420
Asb13	0.464417	5.254514	6.545988	0.010512092	1	46	64	57	78	64	72	126
Socs2	0.462725	5.207952	4.2522	0.039199515	1	22	41	28	42	40	33	68
Clptm1l	0.462398	5.894919	5.109805	0.023790996	1	199	251	150	206	204	136	594
Arfrp1	0.457885	5.225223	6.593036	0.010237841	1	110	108	63	152	142	91	229
Ttc9	0.451099	6.289359	5.875203	0.015355606	1	134	162	82	155	146	159	246
Rpa1	0.450154	5.413764	4.488558	0.034122447	1	104	142	81	132	106	92	315
Tsc1	0.449295	4.893901	4.558431	0.032757078	1	51	82	32	68	63	59	121
Trappc12	0.448823	5.03566	5.151551	0.023225624	1	70	87	66	96	79	96	163

Ube2e2	0.44668	4.890207	4.206762	0.040263136	1	89	109	62	97	83	104	192
Cnot8	0.446298	4.853867	4.882696	0.027127225	1	48	95	32	99	75	68	138
Msrb1	0.444322	5.62259	8.453438	0.003643541	1	90	119	64	90	90	104	187
Mta1	0.444201	5.405769	5.590031	0.01806298	1	142	194	90	126	159	126	303
Cant1	0.442083	5.224731	4.579405	0.032358456	1	82	165	67	105	95	103	246
Elavl1	0.441214	5.181866	5.319116	0.021092712	1	140	203	89	163	160	145	320
Sec23b	0.440487	5.023886	4.812251	0.028258116	1	87	142	62	114	127	87	208
Glmp	0.437709	4.963958	5.364778	0.020547409	1	92	106	78	109	104	104	200
Rnpepl1	0.436304	4.816586	4.346196	0.0370917	1	96	171	96	166	130	142	281
Jade1	0.436167	5.374691	5.68742	0.017086957	1	130	164	121	189	159	149	340
Cnep1r1	0.435756	4.765483	4.162918	0.041318172	1	55	106	39	76	63	73	150
Pcx	0.434268	4.725673	4.274146	0.038696404	1	75	109	50	83	66	77	197
Txndc12	0.432393	5.128982	5.543725	0.018547068	1	158	157	84	171	179	145	263
Ndufa8	0.431757	6.607461	5.503009	0.018983775	1	195	296	187	280	236	193	627
Sec22c	0.431136	5.049272	5.000164	0.025344923	1	57	84	48	84	75	82	121
Xlr4c	0.430775	5.092478	6.635754	0.009995186	1	156	284	145	219	224	190	426
Dazap2	0.429379	7.388363	8.501478	0.003548581	1	593	939	479	768	706	566	1743
Wfs1	0.429129	6.422561	7.601041	0.00583346	1	369	606	306	433	451	425	924
Rhot1	0.428941	5.592336	4.445111	0.035001316	1	61	72	34	61	75	52	108
Id1	0.42837	5.516252	3.950714	0.046851477	1	155	195	116	233	239	158	319
Cryab	0.428175	8.141059	6.384975	0.011509039	1	410	543	356	566	487	374	1203
Apmap	0.427298	4.590947	3.94975	0.046878328	1	47	68	33	76	53	68	107
Nme1	0.426828	7.222221	6.991727	0.008188731	1	150	222	87	229	202	137	375
Anxa1	0.426774	7.461816	4.48147	0.034264229	1	1032	1552	606	1148	1396	1137	1741
Rfc2	0.426407	4.903965	5.475616	0.019283564	1	100	129	52	121	106	113	189
Marveld2	0.42582	4.263142	4.085434	0.043254352	1	102	125	64	112	93	112	216
Hs2st1	0.425641	4.490859	3.907629	0.048067372	1	41	54	34	41	42	62	68
Golph3l	0.4255	4.523199	3.914976	0.047857685	1	78	102	61	60	70	84	165
Ankrd46	0.424453	6.530503	4.214981	0.040068517	1	70	119	38	79	94	44	185
Abi2	0.424348	5.944114	3.973883	0.046211089	1	72	89	38	63	68	65	140
Asb8	0.421948	5.666526	5.813909	0.015899917	1	113	177	86	154	130	126	299
Polr2g	0.421895	5.461968	5.372684	0.020454482	1	67	101	55	126	86	96	175
2410004B1	0.421185	4.563457	4.171436	0.041110943	1	51	80	39	72	70	54	126
Lgmn	0.421015	6.28211	4.930482	0.026386954	1	90	149	92	182	128	92	335
Uap1	0.418383	5.362642	3.863075	0.049359787	1	125	178	86	149	101	122	361
Tapt1	0.417561	4.87601	3.937445	0.047222444	1	72	103	56	84	82	78	168
Polr1d	0.417292	6.643758	4.080564	0.043379185	1	411	540	305	591	451	348	1221
Slc45a4	0.411686	5.28085	4.416609	0.035590706	1	57	58	37	102	70	69	118
Cyth2	0.411307	5.479695	6.001082	0.014297105	1	87	139	81	112	127	114	181
Tecpr1	0.409438	5.567305	5.059286	0.024494333	1	44	66	60	61	64	60	115
Rab33b	0.40903	4.814843	4.278226	0.038603622	1	58	74	47	45	62	64	103
Tfam	0.408428	4.845419	5.73049	0.016672829	1	106	115	54	105	112	82	198
Cyb5a	0.405322	6.096793	4.060847	0.043888473	1	217	266	160	272	237	191	548
Trib2	0.405016	6.677058	4.433196	0.035246451	1	108	81	60	76	73	83	191
Cnpy2	0.404781	5.295378	4.198875	0.040450801	1	143	176	95	156	170	132	283
Id2	0.40442	8.839623	6.234584	0.012527901	1	1966	3286	1596	2772	2184	1785	6475
Prkab1	0.403685	5.150107	5.93679	0.014828004	1	155	223	165	229	225	173	409
Lgalsl	0.402699	5.45172	4.475485	0.034384445	1	155	330	160	269	197	205	557
Gm2a	0.402359	5.737459	4.128761	0.04216021	1	107	156	171	214	223	97	386
Lamtor4	0.400182	6.339943	6.941434	0.008422159	1	189	267	122	276	243	188	436
Golph3	0.398517	7.022396	7.867307	0.005033656	1	471	773	413	687	581	573	1257
Cnst	0.394298	6.031513	5.639656	0.017558652	1	81	98	59	74	82	73	166
Efhd2	0.392787	4.999869	4.643873	0.031164589	1	135	179	110	202	170	141	333
Man1b1	0.391852	5.54013	4.890904	0.026998545	1	131	167	90	174	188	118	258
Arpc5	0.390489	7.294336	4.642007	0.031198493	1	494	815	416	689	595	470	1487
Fam195b	0.390024	5.77742	4.86009	0.027484907	1	78	142	90	155	129	93	257
Cldn23	0.389105	5.518099	5.176399	0.022895724	1	289	403	181	308	304	264	645
Cyp2s1	0.387412	3.923612	4.38887	0.036174273	1	85	115	69	120	114	97	178
Mrpl45	0.387366	5.419803	4.492984	0.034034219	1	72	121	64	107	94	75	205
Srsf6	0.386463	6.587195	6.51113	0.010720134	1	393	529	258	464	420	397	853
Prkaca	0.383902	6.999078	4.196921	0.040497441	1	130	227	146	185	154	156	414
Oat	0.382193	7.191508	6.447082	0.011113471	1	656	924	500	842	773	625	1619
Degs1	0.379546	6.935045	7.891319	0.004967264	1	130	211	102	170	185	140	283
Adprh	0.378042	6.308532	4.415565	0.035612475	1	93	144	76	128	113	94	243
Map2k1	0.377261	5.738861	4.747782	0.02933608	1	180	246	151	222	213	211	376
Ythdf2	0.376702	6.418754	4.544889	0.033017154	1	265	388	229	374	301	251	769
Glyr1	0.376088	6.286558	7.720209	0.005460607	1	186	322	174	219	265	211	423
Rab40c	0.375043	5.453221	4.666546	0.030755721	1	127	226	118	180	166	139	359
Gpx8	0.374735	4.621219	3.943478	0.047053406	1	70	73	59	108	104	90	105
Prdx1	0.374718	7.718587	5.873533	0.015370178	1	673	966	466	915	654	636	1860
Zfp185	0.374053	6.981554	7.319146	0.006822383	1	647	997	494	751	698	648	1620
Alkbh8	0.373469	5.195991	4.372865	0.036515542	1	84	113	66	110	123	84	161
Prkcd	0.371026	6.371449	4.590021	0.032158615	1	188	278	178	286	249	179	537
Gstm1	0.370989	5.473223	4.64574	0.03113072	1	134	214	122	223	226	159	290

Sgms2	0.368699	5.504029	4.513919	0.033620124	1	153	236	133	205	180	151	417
Dpp3	0.367463	6.201457	4.130739	0.042110959	1	195	391	160	270	240	203	591
Rchy1	0.367436	5.605883	6.2967	0.012096292	1	168	272	128	229	205	189	397
Dusp3	0.366528	7.036772	5.671138	0.01724626	1	97	143	77	147	168	108	169
Kctd1	0.365471	4.952394	6.065772	0.013782647	1	83	139	82	124	134	109	173
Samd8	0.365425	6.046384	5.345212	0.020779258	1	93	150	71	111	119	118	174
Ier5	0.365322	7.256974	5.090909	0.024051583	1	791	960	526	826	644	735	1853
Vopp1	0.364102	5.522654	5.72893	0.016687645	1	148	203	103	170	168	138	319
Mt2	0.356463	9.152711	4.366903	0.036643531	1	2695	4431	1976	3685	3299	2631	6880
S100a10	0.355535	9.903885	3.981944	0.045990458	1	3584	6457	2407	4389	4443	3143	9277
Strap	0.353442	6.496282	5.809752	0.015937542	1	260	339	172	266	263	220	566
Ctsl	0.352974	8.936353	4.3399	0.03722912	1	1731	2346	1304	2160	2062	1375	4327
9530068E0	0.352879	6.050662	4.390259	0.03614483	1	181	255	139	241	214	166	443
Serpinb5	0.352794	7.299767	4.022165	0.044906026	1	883	1317	541	1068	920	824	2058
Bub3	0.352266	6.602414	5.30775	0.021230756	1	259	353	222	404	304	262	692
Cd164	0.35143	7.732428	5.046398	0.024677192	1	936	1397	658	1241	1049	920	2260
Pqlc1	0.350041	7.719331	8.318566	0.003924188	1	458	716	315	627	603	412	1073
Camsap1	0.348582	6.02392	5.332733	0.020928545	1	134	165	86	172	125	129	302
Arhgap1	0.345747	6.112594	4.64493	0.031145415	1	275	351	203	333	269	270	632
Zfp664	0.344621	6.575488	6.495978	0.010811883	1	185	254	138	237	204	172	437
Ube2z	0.344161	6.583447	4.784382	0.028718948	1	290	400	231	395	313	270	756
Atox1	0.340939	7.301427	6.055826	0.01386049	1	474	899	448	684	847	544	971
Itfg1	0.340877	6.684132	5.694126	0.017021792	1	125	199	99	219	183	139	298
Mreg	0.336495	6.661494	5.292371	0.021419044	1	438	690	422	643	557	447	1207
Auh	0.334797	5.756313	3.932177	0.047370575	1	50	80	61	88	85	57	130
H3f3b	0.333261	8.262382	4.572077	0.032497142	1	1470	1989	1018	1696	1542	1243	3429
Raph1	0.332366	6.636581	5.411903	0.019999902	1	188	279	182	256	206	186	526
Sdcbp	0.331847	7.278997	4.213474	0.040104125	1	282	457	180	362	331	231	705
Snx27	0.331208	6.866231	5.563032	0.018343613	1	198	309	173	251	219	245	438
Rapgef5	0.331182	6.130051	4.404491	0.035844421	1	246	279	172	225	230	226	457
Saraf	0.330465	7.181279	6.342742	0.011786256	1	176	283	161	262	226	174	474
Arf3	0.330275	7.047052	8.537246	0.003479517	1	198	302	166	268	219	233	455
Selt	0.330009	6.528718	4.131689	0.042087316	1	200	274	130	202	176	187	436
Gsn	0.329802	8.516696	4.568526	0.032564582	1	1302	2296	1186	1958	1722	1286	3730
Mbp	0.328988	10.88539	4.936174	0.026300185	1	496	656	341	582	576	422	1044
Rbx1	0.328231	7.177504	5.692013	0.017042292	1	218	337	211	357	295	206	623
Vapa	0.327237	7.317957	6.149194	0.013147202	1	380	639	385	591	485	441	1048
Bag6	0.324956	6.681055	5.983218	0.014442629	1	340	443	177	395	368	299	628
Mgat1	0.323543	5.277712	3.941734	0.04710221	1	102	158	80	192	155	125	219
Trappc4	0.322145	6.539951	5.333257	0.02092225	1	176	217	114	188	180	153	344
Il17re	0.319966	5.902536	5.193187	0.022675591	1	281	398	220	369	313	284	641
Wsb2	0.319826	6.344035	4.64826	0.031085035	1	175	207	114	252	200	182	329
Dpysl3	0.319414	8.555654	3.988967	0.045799122	1	439	414	363	540	704	349	612
C330006A1	0.318896	6.749733	4.373765	0.036496276	1	156	228	122	225	207	129	380
Azin1	0.318424	6.514475	5.806329	0.015968593	1	180	260	136	266	197	180	445
2010111I0	0.318418	5.631222	4.649367	0.031064992	1	118	175	124	161	147	128	292
Dcaf7	0.316879	6.103888	4.942696	0.026201135	1	228	287	153	267	229	230	440
Cd81	0.315901	7.083424	5.088518	0.024084768	1	226	341	205	351	299	228	569
Ndufs2	0.315259	6.842066	4.434465	0.035220257	1	241	280	170	343	256	208	557
Mapkapk3	0.314252	5.805963	3.970175	0.046312968	1	270	385	217	347	319	291	557
Ola1	0.31345	7.115394	5.408842	0.020034997	1	354	514	244	426	404	338	733
Brd2	0.313204	7.74721	5.148861	0.023261622	1	478	766	380	592	507	487	1176
Psma2	0.313176	7.364281	4.314272	0.037793945	1	423	567	295	623	415	407	1059
Tgoln1	0.312683	7.250828	6.910085	0.008571091	1	223	320	174	292	284	198	493
Rbbp7	0.311273	6.4138	3.869433	0.04917313	1	231	347	189	325	264	213	601
Map1lc3b	0.308899	8.254318	5.562959	0.018344382	1	358	520	314	582	539	383	750
Cops4	0.306642	6.059581	4.987703	0.025528075	1	155	252	124	210	189	182	326
Hnrnpa0	0.304987	7.786256	6.214201	0.012672956	1	785	1156	577	1081	878	729	1895
Fam129b	0.303696	6.669919	4.152685	0.041568553	1	550	700	369	625	563	469	1155
Hspb1	0.303186	8.492267	4.023477	0.044871128	1	1368	2064	1329	1953	1545	1383	3781
Wbp5	0.299143	6.925017	4.81185	0.028264688	1	221	346	180	345	275	222	550
Ankrd13a	0.298886	6.205595	4.805288	0.028372529	1	324	474	252	432	443	320	623
Spg21	0.296522	6.583337	4.419427	0.035531957	1	441	689	295	556	483	406	1005
Ctsb	0.289951	8.730476	5.075217	0.024270238	1	582	920	434	723	694	523	1321
Fam84a	0.287055	7.158107	4.626561	0.031480601	1	781	1244	564	950	900	712	1753
Med28	0.282782	6.377434	4.673165	0.030637424	1	220	342	157	247	265	189	455
Igsf3	0.280669	7.370984	3.974314	0.046199271	1	482	875	443	637	568	525	1227
Col4a1	0.277841	6.596407	4.223627	0.039864866	1	214	319	179	243	286	189	419
Tacstd2	0.277085	8.937077	3.889423	0.04859111	1	2556	3793	2055	3470	2761	2507	6107
Acat2	0.275559	6.685171	3.93015	0.047427713	1	230	391	163	286	262	214	528
Sypl	0.275002	7.483564	4.2311	0.039689705	1	822	1131	670	1133	883	764	1976
Ube3c	0.272835	6.032109	3.896656	0.048382318	1	114	247	111	201	182	146	286
Tprgl	0.271582	7.682192	5.183424	0.022803339	1	351	476	312	521	411	376	773
Foxo1	0.270173	6.397819	4.082758	0.043322885	1	317	438	223	401	333	287	680

Hip1r	0.264716	6.413314	4.759764	0.02913254	1	254	429	214	343	349	290	479
Acaa2	0.263632	6.440724	4.020373	0.044953768	1	262	415	245	300	313	247	596
Prr13	0.261678	7.645859	4.844511	0.02773425	1	559	844	464	777	671	525	1288
Klf7	0.259814	8.761076	4.197128	0.040492495	1	207	308	162	290	241	233	406
Gmfb	0.259151	7.578692	4.201023	0.040399598	1	318	422	263	361	313	292	692
Tex261	0.257965	6.918742	4.387888	0.036195128	1	351	498	241	446	388	304	737
Mtpn	0.256363	7.190975	4.319585	0.037676105	1	406	569	292	495	426	383	825
Cenpb	0.255634	6.259594	4.334522	0.037346893	1	256	352	211	318	288	246	522
Hnrnpk	0.255551	8.318113	7.356632	0.006681582	1	1052	1723	781	1358	1203	1089	2193
Rab3d	0.255058	7.78717	5.005507	0.025266806	1	842	1362	748	1118	1076	771	1970
Paqr8	0.252296	6.333061	4.254979	0.039135427	1	308	413	264	330	325	308	587
Abhd12	0.249611	7.540435	3.938068	0.04720495	1	292	370	235	409	290	295	633
Plekhb2	0.247244	7.477556	4.065946	0.043756175	1	422	589	367	512	529	413	785
Mbnl1	0.247215	7.851061	4.049696	0.044179303	1	413	691	357	605	456	408	1075
Tor1aip2	0.244001	6.711552	3.935773	0.047269422	1	444	673	340	624	489	452	964
Myl12b	0.241041	7.413594	4.158168	0.041434204	1	496	809	413	710	639	508	1049
Ddr1	0.239093	7.937609	4.122182	0.042324454	1	1041	1629	894	1452	1196	1010	2451
Ddx3x	0.236071	8.532091	4.538578	0.033139115	1	1060	1690	898	1423	1110	1006	2638
Cnbp	0.233846	7.144725	4.279823	0.03856737	1	427	615	380	651	541	408	952
Cpe	0.230814	8.81282	4.441878	0.035067655	1	662	1072	608	973	874	621	1546
Ivns1abp	0.221838	7.486647	3.958901	0.046624124	1	590	877	511	741	759	549	1136
2310022B(	0.209668	6.762892	3.855359	0.049587317	1	375	640	324	518	464	402	772
Cpne3	0.201805	8.228367	4.397879	0.035983661	1	1029	1504	807	1501	1155	1065	2041
Ctnnb1	0.189719	8.847734	5.012473	0.025165332	1	1554	2519	1209	2104	1903	1514	2981
**Downregulated genes**												
LOC10263!	-5.752825	-0.055144	12.17156	0.000485237	0.629451	7	6	5	3	0	0	0
Gm41222	-5.367518	-0.587583	7.279046	0.006976358	1	1	6	6	3	0	0	0
Pde6a	-5.311882	0.219871	6.183949	0.012891428	1	7	3	3	2	0	0	0
Serpina3n	-5.304747	5.647164	33.34957	7.69947E-09	0.000277	24	15	123	350	3	4	2
Drd1	-5.058286	0.6166	7.547003	0.006010997	1	4	5	3	1	0	0	0
AI182371	-4.981802	-0.154456	9.31909	0.002267785	1	2	5	2	4	0	0	0
4930473A(	-4.943416	-0.262424	5.555953	0.018417943	1	5	5	2	0	0	0	0
Cd84	-4.904438	0.396385	10.96995	0.000926012	0.679882	4	1	1	6	0	0	0
Lrtm1	-4.902627	-0.440331	4.57924	0.032361567	1	4	7	0	2	0	0	0
Gm39423	-4.894826	-0.458121	6.011277	0.014214737	1	5	5	1	1	0	0	0
LOC10816!	-4.887474	0.262441	7.244087	0.007113489	1	3	6	2	1	0	0	0
Frmpd4	-4.881339	3.048396	12.66546	0.000372473	0.558336	0	4	1	7	0	0	0
Gm32639	-4.878904	-0.450055	5.230276	0.022197007	1	2	4	0	7	0	0	0
Gm26744	-4.792023	0.149143	5.780522	0.016204728	1	0	7	4	0	0	0	0
LOC10263:	-4.791967	-0.702569	4.510063	0.033695996	1	1	4	5	0	0	0	0
Map3k19	-4.743318	-0.133753	6.676426	0.009769632	1	2	1	1	7	0	0	0
Cfap157	-4.721722	-0.273147	7.384326	0.006579473	1	5	3	2	0	0	0	0
Sdhaf3	-4.704416	0.087753	7.095443	0.007728021	1	3	4	0	4	0	0	0
Irx3os	-4.679219	-0.621751	6.311987	0.01199244	1	1	6	1	3	0	0	0
Gm40126	-4.67537	-0.167668	6.754036	0.009353585	1	0	4	1	6	0	0	0
Gm30116	-4.665929	1.235155	8.616741	0.003330873	1	0	0	3	7	0	0	0
Pcdhgc3	-4.650776	-0.447899	5.141202	0.023364464	1	2	2	4	1	0	0	0
Stxbp5l	-4.646476	4.572881	11.14538	0.000842411	0.679882	2	2	1	5	0	0	0
Crygb	-4.601072	3.733414	6.336136	0.011830231	1	0	4	4	40	1	0	0
Tmprss5	-4.59678	2.560525	10.18349	0.001417034	0.864055	0	10	0	0	0	0	0
Gm31893	-4.589278	-0.576155	5.470957	0.019335037	1	1	1	1	7	0	0	0
Gm38822	-4.562104	-0.63901	6.062341	0.013809451	1	2	2	0	6	0	0	0
Gm35639	-4.560615	-0.776069	4.93786	0.026274545	1	0	7	2	1	0	0	0
Tcam1	-4.531186	-0.082885	6.434604	0.011191809	1	4	2	1	2	0	0	0
4930405A:	-4.527408	0.230652	6.59475	0.010227993	1	2	2	2	3	0	0	0
Gm34858	-4.517019	-0.159551	4.238619	0.039514301	1	0	3	5	0	0	0	0
Rp1	-4.507303	0.055978	4.894961	0.026935185	1	5	0	2	1	0	0	0
Adam23	-4.502717	6.778601	10.14145	0.001449719	0.869252	5	3	0	1	0	0	0
Gm33234	-4.495222	-0.65052	4.904659	0.026784341	1	3	1	3	1	0	0	0
Gm30627	-4.495155	-0.031477	7.28273	0.006962065	1	1	3	2	3	0	0	0
Stac	-4.491769	4.285059	9.970782	0.001590439	0.88998	0	6	0	3	0	0	0
Mlc1	-4.491459	3.899752	9.894808	0.001657457	0.88998	0	2	0	7	0	0	0
Cerkl	-4.488455	0.885266	7.438036	0.006385955	1	0	1	2	6	0	0	0
Gm34628	-4.452289	-0.794073	4.088336	0.043180142	1	2	3	3	0	0	0	0
Gm33703	-4.429516	-0.029279	5.620758	0.017748975	1	1	0	3	4	0	0	0
9630028I0	-4.413932	-0.054115	6.538496	0.010556459	1	1	0	3	4	0	0	0
Gm38516	-4.409171	-0.528302	4.848665	0.027667532	1	2	0	2	4	0	0	0
Lama1	-4.389929	0.440573	6.390424	0.011473759	1	0	0	3	5	0	0	0
H2-Q7	-4.378188	0.672591	5.842855	0.015640425	1	5	0	0	3	0	0	0
Reck	-4.373113	0.442222	7.052889	0.007913752	1	1	4	3	0	0	0	0
a	-4.357529	-0.049012	4.580007	0.032347087	1	0	5	3	0	0	0	0
Gm39648	-4.351223	-0.045146	5.024175	0.024995826	1	5	3	0	0	0	0	0
Syt5	-4.344812	4.335251	8.851657	0.002928229	1	5	1	0	2	0	0	0
Tmem178l	-4.337294	5.064478	8.961871	0.002756725	1	1	1	0	6	0	0	0

Evl	-4.333955	1.722686	8.250938	0.004073094	1	1	5	2	0	0	0	0
Chst7	-4.318482	-0.023666	3.866154	0.049269297	1	0	0	6	0	0	0	0
Gm30992	-4.312581	-0.514095	4.735129	0.029552639	1	1	0	1	6	0	0	0
Plcb2	-4.296054	0.032452	4.33313	0.037377443	1	0	0	6	0	0	0	0
Gm12248	-4.293082	-0.353228	4.830823	0.027955288	1	1	2	1	4	0	0	0
Naip6	-4.274043	-0.195181	3.934567	0.047303313	1	2	0	0	6	0	0	0
Ccdc36	-4.212214	-0.636381	4.697356	0.030209062	1	1	0	2	4	0	0	0
Cd160	-4.20329	0.191791	6.014605	0.014187954	1	0	6	0	2	0	0	0
Cntf	-4.199386	0.019567	3.891733	0.04852433	1	3	0	3	0	0	0	0
Gm34283	-4.186615	0.064735	5.201398	0.022568728	1	1	2	2	2	0	0	0
Gm33195	-4.176034	0.079939	6.393549	0.01145358	1	1	2	2	2	0	0	0
Nudt10	-4.173544	2.464838	7.772129	0.005305847	1	1	2	4	0	0	0	0
Gm32720	-4.170046	0.759447	6.785922	0.009187954	1	3	3	1	0	0	0	0
1700065J1	-4.163479	-0.141701	5.378299	0.020388751	1	4	2	0	1	0	0	0
Rasgrf2	-4.162706	5.019307	7.774973	0.005297499	1	1	0	2	4	0	0	0
Gm36917	-4.160373	-0.774196	4.481055	0.034272561	1	1	1	4	0	0	0	0
Gm15396	-4.154802	-0.720107	6.324269	0.011909658	1	0	2	2	3	0	0	0
Kcnmb2	-4.140111	2.600112	7.586559	0.005880509	1	0	3	0	4	0	0	0
Anxa11	-4.13782	-0.545831	4.802368	0.028420653	1	2	4	1	0	0	0	0
4931423N:	-4.136097	-0.468177	4.846925	0.02769546	1	1	1	1	4	0	0	0
LOC10816	-4.121761	-0.643989	3.852205	0.049680636	1	3	2	0	2	0	0	0
Spata17	-4.121057	-0.453748	3.882421	0.048794144	1	5	0	1	0	0	0	0
Gc	-4.103963	0.239476	4.51938	0.033512968	1	5	0	1	0	0	0	0
Ahrr	-4.08398	-0.449125	3.90872	0.048036184	1	1	5	1	0	0	0	0
Gm32694	-4.072721	-0.3054	3.981035	0.04601529	1	2	3	0	2	0	0	0
Il1rl1	-4.069509	0.718163	6.043647	0.013956425	1	3	0	3	0	0	0	0
Lrrc18	-4.067495	0.010919	4.614996	0.031693576	1	1	2	3	0	0	0	0
Gm31402	-4.065579	-0.396254	4.171896	0.041099788	1	0	0	3	3	0	0	0
Vaultrc5	-4.048575	-0.015661	3.953211	0.046782024	1	2	2	2	0	0	0	0
A830082K:	-4.048328	1.075979	6.088125	0.013609342	1	0	7	0	0	0	0	0
Fam167a	-4.040828	0.507363	5.944798	0.014760783	1	0	7	0	0	0	0	0
4933413J0	-4.03917	-0.573391	4.403103	0.035873603	1	2	2	2	0	0	0	0
LOC10263	-4.027167	-0.886794	4.886842	0.027062138	1	2	2	2	0	0	0	0
Gm40706	-4.0237	-0.466374	4.344871	0.037120584	1	0	3	3	0	0	0	0
Gm34268	-4.017874	-0.121433	4.745958	0.029367194	1	1	0	2	3	0	0	0
Pxdn	-4.014389	2.299819	6.535245	0.010575764	1	2	0	4	0	0	0	0
Gal3st3	-4.010738	1.881369	5.906172	0.015087919	1	1	2	3	0	0	0	0
Gm35193	-3.985455	-0.887149	3.998477	0.045541393	1	4	1	0	1	0	0	0
Gm19461	-3.971489	1.850681	6.644164	0.009948117	1	5	0	1	0	0	0	0
Sh2b2	-3.966155	0.790634	5.306233	0.021249256	1	0	1	2	3	0	0	0
Ppp1r16b	-3.952411	3.781512	6.71505	0.009560262	1	0	3	3	0	0	0	0
Adgrb1	-3.948307	3.85242	6.652049	0.009904188	1	0	3	2	1	0	0	0
Dner	-3.946981	6.751018	6.749089	0.009379557	1	1	1	0	4	0	0	0
Gm35143	-3.942958	-0.761623	4.731765	0.029610476	1	1	1	1	3	0	0	0
LOC10816	-3.941067	-0.443753	3.843825	0.04992949	1	0	0	4	1	0	0	0
Inpp5j	-3.937555	4.693939	6.647956	0.009926964	1	0	5	0	1	0	0	0
Slitrk2	-3.9331	3.079538	6.547149	0.010505234	1	0	3	0	3	0	0	0
P2ry12	-3.928499	1.5783	6.10249	0.013499154	1	2	3	0	1	0	0	0
Extl1	-3.925329	1.644354	6.377677	0.011556462	1	0	2	0	4	0	0	0
C130060K:	-3.924384	0.040335	4.584836	0.032256065	1	2	0	0	4	0	0	0
Gm35165	-3.923931	-0.868829	5.682632	0.017133648	1	2	2	0	2	0	0	0
Trim46	-3.921355	3.011813	6.414336	0.011320262	1	0	5	0	1	0	0	0
2610307P1	-3.919683	1.270753	6.319164	0.011943995	1	0	3	0	3	0	0	0
Itpka	-3.914633	-0.183241	4.111639	0.042589061	1	0	0	1	5	0	0	0
Gpr183	-3.889692	0.650759	10.50679	0.00118937	0.75068	7	6	3	10	1	0	0
Cpsf4l	-3.879379	-0.152728	4.471889	0.034456886	1	2	4	0	0	0	0	0
Pnma3	-3.877341	0.842892	5.140175	0.023378286	1	0	0	5	0	0	0	0
Rec114	-3.852641	0.5716	4.626061	0.031489782	1	3	0	2	0	0	0	0
Ajap1	-3.843902	0.429459	4.032451	0.044633065	1	1	3	0	2	0	0	0
Il1rapl1	-3.84219	0.816949	4.943649	0.026186701	1	0	0	4	1	0	0	0
Mx2	-3.835942	0.979165	10.23421	0.001378599	0.855111	1	16	1	8	0	0	1
4930432J0	-3.835548	-0.443999	3.999646	0.045509817	1	2	1	2	0	0	0	0
Slc47a2	-3.831632	0.034201	4.633975	0.031344857	1	1	1	3	0	0	0	0
Gpr182	-3.808454	0.210147	4.686196	0.030405896	1	0	6	0	0	0	0	0
Gm31279	-3.800583	-0.38064	4.535245	0.033203709	1	0	1	3	1	0	0	0
LOC10263	-3.799944	-0.566275	3.923273	0.047622072	1	3	1	1	0	0	0	0
Kcnq4	-3.765534	5.642545	18.5556	1.65019E-05	0.118735	6	6	5	6	1	0	0
Gm16035	-3.760932	1.045479	5.396956	0.020171904	1	3	0	2	0	0	0	0
Gm41556	-3.74838	0.809916	4.746009	0.029366327	1	2	0	1	2	0	0	0
Slc13a3	-3.741668	3.157903	5.354516	0.020668673	1	5	0	0	0	0	0	0
Shisa9	-3.737446	0.118338	4.290459	0.038326811	1	2	2	1	0	0	0	0
Kcnk12	-3.730539	1.177266	5.161611	0.02309147	1	4	1	0	0	0	0	0
Sptbn5	-3.724562	0.549872	4.375468	0.036459825	1	1	0	1	3	0	0	0
Gm33753	-3.722009	-0.689542	4.066515	0.043741442	1	0	3	2	0	0	0	0

Lepr	-3.715931	0.890517	4.538283	0.033144816	1	0	3	2	0	0	0	0
Maneal	-3.709627	4.037997	5.569998	0.018270781	1	0	0	2	3	0	0	0
Adamts15	-3.707739	2.850974	5.606273	0.017896288	1	2	1	1	1	0	0	0
Gjb1	-3.707394	4.306355	5.583246	0.018133079	1	2	2	1	0	0	0	0
Nptx1	-3.704137	6.035924	5.636172	0.01759358	1	2	1	0	2	0	0	0
Arhgdig	-3.703099	6.290981	5.6281	0.017674776	1	0	2	1	2	0	0	0
Mpped1	-3.694308	1.271261	4.866959	0.027375697	1	2	1	0	2	0	0	0
E130114P1	-3.686847	1.398398	5.478742	0.019249103	1	0	5	0	0	0	0	0
Usp29	-3.682834	2.535857	5.389028	0.020263754	1	0	3	0	2	0	0	0
Gm33392	-3.679388	-0.200677	4.208531	0.040221145	1	0	1	1	3	0	0	0
Hist1h2ad	-3.656164	-0.690432	4.310193	0.037884663	1	1	1	0	3	0	0	0
Gm14808	-3.63962	0.433133	4.363996	0.036706112	1	0	0	0	5	0	0	0
Gm33040	-3.634172	1.406952	4.85298	0.027598417	1	0	5	0	0	0	0	0
LOC10524	-3.609561	-0.301499	3.920442	0.04770234	1	1	4	0	0	0	0	0
Gm16897	-3.559467	-0.162555	4.047941	0.044225245	1	1	0	3	0	0	0	0
LOC10263	-3.517438	0.101747	4.085436	0.043254284	1	6	1	7	4	1	0	0
Slc10a6	-3.438825	0.975019	4.15029	0.041627397	1	0	1	2	1	0	0	0
Trim9	-3.429308	4.345109	13.19618	0.000280521	0.509334	12	3	1	2	1	0	0
Lck	-3.42235	0.581034	4.176864	0.040979473	1	1	1	1	1	0	0	0
Mir124-2h	-3.416657	0.806869	4.112847	0.042558652	1	2	0	0	2	0	0	0
Stac2	-3.414052	4.686021	4.510299	0.033691357	1	2	0	1	1	0	0	0
Dusp15	-3.413617	5.422177	4.506716	0.033761994	1	0	0	2	2	0	0	0
Cpne4	-3.411873	4.910811	4.507995	0.033736761	1	3	1	0	0	0	0	0
Tmeff2	-3.411865	5.004289	4.508139	0.033733927	1	3	1	0	0	0	0	0
Kcnt1	-3.408732	3.777935	4.491228	0.034069187	1	1	0	0	3	0	0	0
Celsr3	-3.408219	3.38608	4.483083	0.034231921	1	0	2	1	1	0	0	0
Lrrn1	-3.408129	5.423629	4.515307	0.033592856	1	0	1	0	3	0	0	0
Gm10419	-3.407756	4.614146	4.492154	0.034050749	1	0	3	1	0	0	0	0
Notch4	-3.40279	0.918817	3.975854	0.046157035	1	0	1	1	2	0	0	0
Ptprn2	-3.402385	4.055296	4.456092	0.034776966	1	0	2	0	2	0	0	0
Srrm4	-3.402339	2.803423	4.361467	0.036760644	1	1	1	0	2	0	0	0
Cldn5	-3.401366	3.74065	4.389792	0.036154729	1	1	2	0	1	0	0	0
Aard	-3.401299	2.350652	4.40748	0.035781669	1	0	0	0	4	0	0	0
Adamts4	-3.398523	1.389901	3.852289	0.049678161	1	2	2	0	0	0	0	0
Rian	-3.394051	1.963441	4.296409	0.03819294	1	0	0	0	4	0	0	0
Itgb3	-3.392379	3.192407	4.383305	0.036292551	1	0	4	0	0	0	0	0
Gm7616	-3.387173	-0.424862	6.565448	0.010397756	1	2	8	6	2	0	1	0
Myo16	-3.387118	0.724691	3.983382	0.045951211	1	0	3	1	0	0	0	0
Nsg1	-3.348778	4.186213	12.31225	0.000449994	0.622654	8	1	3	5	0	1	0
Rasl10a	-3.343697	0.611745	3.850186	0.049740471	1	0	3	0	1	0	0	0
Tnfrsf13c	-3.338571	0.389558	3.867913	0.0492177	1	0	4	0	0	0	0	0
Tnc	-3.333613	0.930741	10.56234	0.001154152	0.75068	6	9	10	5	1	1	0
Ltbp2	-3.328065	1.698738	8.831867	0.002960154	1	2	8	2	6	0	1	0
Unkl	-3.184805	-0.05042	4.163706	0.041298948	1	5	4	2	5	1	0	0
Tnfaip8l1	-3.177117	2.55769	8.37014	0.003814362	1	1	6	2	7	0	1	0
LOC10816	-3.177082	0.254543	4.491898	0.03405585	1	7	4	6	12	1	1	0
1700018L0	-3.176231	-0.040777	6.055677	0.013861661	1	4	4	2	6	0	1	0
Serpina3h	-3.133323	-0.068309	4.108086	0.042678644	1	8	1	3	2	1	0	0
Rinl	-3.092362	0.963263	6.740629	0.009424137	1	3	0	4	7	1	0	0
Ptprz1	-3.07315	5.336789	9.924145	0.001631246	0.88998	5	1	3	5	0	0	1
LOC10816	-2.881381	2.53905	6.849351	0.008867349	1	3	0	2	7	0	0	1
Gm42289	-2.880623	0.249333	4.630262	0.031412763	1	2	12	1	11	2	0	0
Gm26721	-2.87249	0.296724	4.301637	0.038075701	1	4	3	3	1	0	0	1
LOC10264	-2.862242	0.996483	6.496398	0.010809324	1	2	1	4	5	0	1	0
Cadm3	-2.851791	7.767142	8.057137	0.004532476	1	3	5	3	1	0	0	1
Dysf	-2.850681	4.811228	8.061779	0.004520878	1	3	4	4	1	1	0	0
Tmod1	-2.836094	2.263423	6.749707	0.009376307	1	0	0	1	11	0	0	1
Skida1	-2.829939	3.063744	7.239949	0.007129902	1	3	4	1	4	1	0	0
Cxcl1	-2.823399	-0.025287	4.78353	0.028733163	1	1	8	1	3	1	0	0
Crabp2	-2.819607	1.465597	6.162288	0.013050233	1	0	8	4	0	1	0	0
Acr	-2.805672	0.238746	4.568076	0.032573121	1	2	6	0	4	0	0	1
Ppp1r14a	-2.790978	2.741422	6.719782	0.009534926	1	5	0	4	2	0	0	1
Ttll8	-2.754377	1.723184	6.441969	0.011145505	1	4	3	2	2	0	0	1
Gm40571	-2.743961	0.632659	8.644221	0.003281005	1	8	5	1	6	0	1	1
Ankrd33b	-2.734713	1.971212	6.301226	0.012065447	1	2	0	1	8	0	0	1
LOC10264	-2.70783	0.904844	5.305333	0.02126024	1	0	1	3	7	1	0	0
Gm29883	-2.692984	1.489204	5.820453	0.015840861	1	3	9	9	9	0	3	0
Gm41577	-2.683928	3.159376	6.043307	0.01395911	1	108	17	89	23	9	13	8
LOC10263	-2.640052	0.992065	4.53499	0.033208642	1	2	7	1	1	1	0	0
March1	-2.607978	1.508503	4.73805	0.029502486	1	3	0	0	7	0	0	1
Tvp23bos	-2.596969	1.873543	6.62618	0.010049057	1	1	4	11	1	0	2	0
Gm16263	-2.583067	0.08403	6.772655	0.009256503	1	4	4	5	5	0	2	0
Car7	-2.581111	1.342408	5.029799	0.024914795	1	0	2	3	5	1	0	0
Col9a2	-2.566301	2.924113	8.485938	0.003579021	1	11	1	2	3	1	0	1

Prrx2	-2.558336	1.079589	4.379	0.036384333	1	3	8	4	1	0	0	2
B3gnt6	-2.542935	0.180224	5.267206	0.021730863	1	3	7	4	4	2	0	0
Gm41622	-2.540118	1.793573	13.02553	0.000307273	0.509334	16	7	9	27	1	1	6
Insm2	-2.536125	1.969905	8.715673	0.003154859	1	1	3	7	13	1	0	2
Gm30368	-2.531189	0.580871	4.89364	0.026955796	1	3	6	4	4	0	1	1
Nos1	-2.499244	4.466061	11.60165	0.000658933	0.632525	7	10	6	1	1	1	1
Greb1l	-2.481419	1.560441	8.240636	0.004096278	1	3	5	4	11	0	0	3
6430550D2	-2.470848	0.296381	5.543143	0.018553237	1	3	6	10	7	0	0	4
Slc26a8	-2.461043	-0.132587	4.07354	0.043559901	1	2	7	3	6	1	1	0
Cyp17a1	-2.460488	0.184633	7.596868	0.005846979	1	8	8	3	11	0	1	3
LOC102632	-2.458851	-0.288007	5.795509	0.016067165	1	2	5	5	11	0	1	2
Synpr	-2.452401	5.66366	5.330019	0.020961161	1	5	2	1	1	0	0	1
Chga	-2.451018	6.951591	5.341372	0.020825071	1	4	1	1	3	0	0	1
6330403K0	-2.446971	4.309478	5.191209	0.022701423	1	1	5	3	0	1	0	0
Lrriq1	-2.441309	2.492961	5.034513	0.024847066	1	1	2	0	6	0	0	1
LOC108168	-2.432777	0.303683	5.658661	0.017369383	1	13	4	7	10	1	1	3
Aqp11	-2.395472	1.674428	4.354043	0.0369212	1	1	3	0	5	1	0	0
BC025920	-2.383869	0.906987	4.687842	0.030376767	1	3	7	1	5	1	0	1
Vat1l	-2.36408	6.458393	11.13174	0.00084863	0.679882	4	5	1	12	1	0	2
Zic1	-2.356987	2.837442	11.23659	0.000802005	0.679882	14	11	8	13	1	1	5
Drp2	-2.350431	7.035631	7.943057	0.004827222	1	3	2	8	2	1	1	0
1500004A1	-2.343867	3.603099	7.367772	0.006640317	1	4	4	4	3	0	2	0
Mmrn2	-2.311469	2.569498	6.401949	0.011399513	1	4	7	0	4	0	1	1
Ptgir	-2.295135	5.002621	4.294116	0.038244467	1	0	3	3	2	0	0	1
Gm31166	-2.293813	1.404617	4.602545	0.031924523	1	7	3	3	0	0	0	2
Fabp7	-2.293727	6.956099	4.325765	0.037539536	1	3	2	1	2	0	0	1
Gm31633	-2.282568	0.466104	4.275831	0.038658063	1	10	4	7	4	0	2	2
Fstl4	-2.276807	2.602653	12.53997	0.000398338	0.573225	3	13	7	10	1	0	4
Gm40142	-2.245542	0.579779	5.22678	0.022241666	1	8	4	3	5	1	1	1
Pde1a	-2.239312	2.975759	8.485923	0.00357905	1	15	6	0	5	1	1	2
Tmod2	-2.23038	7.824253	9.552036	0.001997277	0.981214	5	8	3	4	1	1	1
LOC102633	-2.208225	0.930841	4.139344	0.041897415	1	4	10	7	5	3	0	1
Coro2b	-2.207544	6.191746	13.45511	0.00024434	0.509334	7	9	1	15	1	1	3
Srl	-2.200961	1.666259	4.785107	0.02870686	1	7	3	0	4	1	1	0
Slc6a13	-2.18931	3.084767	5.799563	0.016030159	1	10	1	2	0	1	0	1
Rasip1	-2.157082	2.856901	5.519258	0.018808234	1	0	3	5	5	0	1	1
Pcp4	-2.152615	7.95484	20.66738	5.4639E-06	0.098285	17	16	8	7	4	2	2
Lgals4	-2.104636	1.776446	7.206262	0.007264966	1	9	19	15	2	3	2	3
L3mbtl1	-2.098597	1.028991	5.154055	0.023192157	1	1	1	3	13	1	0	2
Rassf10	-2.080501	1.923703	4.894659	0.026939888	1	2	3	6	1	1	0	1
Gm16894	-2.073337	0.671947	3.999517	0.045513315	1	4	5	3	0	1	0	1
Ccdc106	-2.068754	1.26961	6.764725	0.009297726	1	4	7	1	12	2	0	2
Spats1	-2.03979	0.887575	4.646694	0.031113413	1	3	2	0	7	0	0	2
Syt14	-2.029522	3.114973	7.088217	0.00775924	1	4	5	12	6	3	1	1
Mnda	-2.016902	1.090737	5.956042	0.014666932	1	14	16	10	12	0	4	6
Cyp7b1	-2.013839	2.012273	4.14028	0.04187423	1	5	3	2	2	0	2	0
Syp	-2.004911	6.965339	7.180097	0.007371672	1	6	4	1	6	1	0	2
Gulp1	-2.000409	4.655951	8.818239	0.002982343	1	5	3	10	4	4	0	0
Slc4a5	-1.970564	1.041745	6.456901	0.011052221	1	24	30	5	14	4	0	11
Pcsk2	-1.913028	6.453685	6.255673	0.012379624	1	2	9	2	3	0	1	2
Platr14	-1.911853	0.44118	3.994993	0.045635628	1	3	5	3	4	0	0	3
Gm16118	-1.910292	0.546932	4.287965	0.038383088	1	7	6	10	4	1	1	4
Scarna17	-1.909848	1.532545	6.535949	0.010571582	1	23	19	4	13	8	1	2
Tspyl4	-1.882739	6.172304	18.94601	1.3447E-05	0.118735	9	34	14	19	7	4	4
Gm15411	-1.870529	1.606648	6.590711	0.010251225	1	1	13	4	9	4	1	0
Nr2f1	-1.859445	5.713022	13.07239	0.000299681	0.509334	5	17	3	15	2	4	2
Rasa4	-1.858106	1.955218	3.892742	0.048495184	1	7	0	4	3	1	0	2
Gm15866	-1.836228	1.560613	5.917105	0.014994574	1	17	23	15	17	3	6	6
Fam19a1	-1.827198	3.531299	7.744818	0.00538669	1	5	11	3	5	0	0	5
Slc35f1	-1.825131	5.818456	5.389953	0.020253021	1	1	2	5	7	3	0	0
Camk2b	-1.822722	4.839911	5.339636	0.020845827	1	4	6	4	1	2	1	0
Lrrc75a	-1.818881	2.551867	9.611871	0.001933236	0.979579	5	10	15	7	2	3	3
Zic2	-1.815801	2.945054	5.042259	0.024736207	1	5	4	1	11	0	4	0
Negr1	-1.811073	3.736321	7.956049	0.004792691	1	4	4	9	7	2	3	0
Hmgcll1	-1.808365	2.12137	4.936855	0.026289825	1	5	9	0	6	2	0	2
Gm10677	-1.806863	1.843324	6.775209	0.009243266	1	11	19	20	11	3	1	11
Brsk1	-1.788102	5.979026	6.495362	0.010815627	1	8	2	2	7	1	1	2
Snap25	-1.777069	8.981876	12.01274	0.000528381	0.629451	6	11	10	10	1	5	2
BC034090	-1.745773	1.809209	6.043845	0.013954861	1	7	8	3	13	2	0	5
BC025446	-1.73378	0.590683	6.500139	0.010786608	1	6	14	6	10	3	1	4
Ppm1e	-1.731097	4.465033	5.804011	0.015989661	1	4	5	6	3	1	0	3
Gm3716	-1.702562	1.437545	5.2785	0.021590341	1	18	21	6	11	4	5	3
Cdh6	-1.70121	4.794791	5.794286	0.016078345	1	5	3	3	7	1	2	1
Pycr1	-1.687331	1.29774	4.85742	0.027527471	1	5	8	5	4	1	2	2

Pcsk1n	-1.683	8.288263	7.082467	0.007784177	1	9	9	2	2	3	1	1
Sorbs2os	-1.665897	0.873847	4.738052	0.029502454	1	18	10	12	16	6	4	3
Adam19	-1.663656	4.50717	11.5858	0.000664572	0.632525	18	7	17	2	1	3	7
Rdh12	-1.663279	1.316214	4.033765	0.044598295	1	8	8	3	5	1	0	5
1700030K(	-1.656955	0.99009	4.501834	0.033858519	1	4	13	4	14	3	1	4
Runx3	-1.632762	3.876376	5.665281	0.017303942	1	1	9	7	4	0	2	3
Eml6	-1.61579	2.084295	4.112318	0.042571964	1	3	12	10	16	2	6	1
Prrg3	-1.615147	5.311076	6.051611	0.013893614	1	7	5	3	6	2	2	1
Tiam2	-1.607076	2.226622	6.472459	0.010955884	1	23	26	19	25	4	5	16
Cacna2d1	-1.572114	6.767556	7.024979	0.008038042	1	3	4	9	8	1	1	4
Gria1	-1.558952	3.28212	9.167338	0.002463726	1	13	33	8	11	8	2	6
Chn1	-1.557955	5.565382	5.791062	0.016107854	1	7	8	2	3	1	0	4
Cfap69	-1.539853	2.272463	10.61052	0.001124464	0.749143	22	17	12	21	5	5	9
Kcnb2	-1.533082	6.964807	4.609547	0.03179444	1	2	6	0	8	0	1	3
Akap5	-1.528591	6.473028	18.68606	1.54105E-05	0.118735	21	18	17	36	8	8	8
Cabyr	-1.528216	2.868753	5.362163	0.02057823	1	7	5	6	5	0	3	3
Ogdhl	-1.526668	4.033356	7.586364	0.005881145	1	2	11	6	13	3	5	0
Vcan	-1.51706	2.451974	6.244794	0.012455889	1	20	11	2	6	2	5	3
Spin2c	-1.512983	0.992779	3.933218	0.047341274	1	2	15	1	17	3	3	2
LOC10816	-1.512431	1.678466	3.978603	0.046081765	1	12	16	9	7	1	7	3
Arid5a	-1.506166	2.314471	3.995134	0.045631812	1	2	10	0	4	2	0	2
Erbb3	-1.492865	1.74751	6.121012	0.01335843	1	15	28	20	16	9	5	7
Pou4f1	-1.482361	7.215058	5.205853	0.022510966	1	2	5	7	5	1	2	2
Ppp1r1a	-1.477026	4.721839	6.378136	0.011553472	1	4	16	3	4	2	4	1
Snhg7	-1.463035	3.003114	16.26381	5.51065E-05	0.230947	30	43	33	28	9	10	19
Lgi4	-1.455532	5.591779	3.895603	0.048412653	1	4	4	6	1	0	3	1
Nxpe4	-1.451669	1.173718	3.864864	0.049307204	1	13	5	8	4	3	1	5
Srgn	-1.444888	3.616581	5.127594	0.023548349	1	6	6	2	8	1	1	4
9330104G(	-1.41786	2.030485	6.813584	0.009046699	1	8	25	11	20	7	9	0
Rad51c	-1.406249	1.870233	5.091435	0.024044294	1	15	10	8	11	4	6	2
Tubb4a	-1.394338	6.835143	4.481833	0.034256968	1	2	6	2	8	1	3	1
Ptgds	-1.387065	10.45326	6.03753	0.014004864	1	365	435	147	350	157	86	106
Med12l	-1.361376	3.721723	9.253247	0.002350782	1	25	24	15	14	4	6	14
Als2	-1.357341	2.369368	4.430122	0.035309979	1	9	16	3	3	2	2	5
Snx32	-1.355779	5.385438	7.613716	0.005792599	1	9	11	3	15	4	3	4
Trim36	-1.346483	5.985265	6.395613	0.011440273	1	14	10	4	2	2	0	7
Slc15a2	-1.337716	4.102183	5.410738	0.020013252	1	139	53	94	162	3	51	98
Pld5	-1.333962	5.621073	6.576537	0.010333172	1	21	24	3	16	7	6	5
1700099I0	-1.321838	1.585695	4.93024	0.026390658	1	19	24	27	28	10	11	8
Atp2b2	-1.317997	6.9242	3.895509	0.04841536	1	5	6	1	5	0	3	2
Ildr2	-1.308009	3.712202	4.182585	0.040841365	1	7	6	0	10	0	1	6
Nmb	-1.278681	6.106419	6.035499	0.01402099	1	8	5	3	13	1	1	7
Klc3	-1.278633	0.790103	4.090128	0.043134385	1	12	10	13	8	8	4	1
Car15	-1.24931	1.974369	3.964759	0.046462171	1	5	12	5	7	2	3	4
Gm36582	-1.248781	5.137063	4.252924	0.039182818	1	175	269	202	238	0	115	191
Tet1	-1.246103	3.409714	7.408214	0.006492675	1	14	16	11	16	5	1	13
Reep2	-1.234346	5.65528	3.903064	0.048198121	1	9	4	2	4	0	3	3
Prrg1	-1.202646	2.898797	4.106843	0.04271002	1	11	8	5	1	4	4	0
Reln	-1.201857	5.128322	8.035656	0.00458654	1	24	20	3	14	8	10	1
Zfp382	-1.193781	4.351185	6.164743	0.013032128	1	12	16	6	8	5	1	8
Tmem45a	-1.181198	3.921098	6.4292	0.011225914	1	20	21	42	53	11	14	23
Gm35260	-1.170477	1.892179	5.567062	0.018301444	1	15	28	11	25	11	4	11
Stmn3	-1.169254	9.277166	6.54472	0.01051959	1	7	10	6	13	5	4	3
Helq	-1.158098	1.819247	6.88186	0.00870749	1	18	14	11	23	6	9	7
Sema6a	-1.152347	4.925386	6.662706	0.00984513	1	14	20	7	19	12	1	7
Plxnd1	-1.150817	4.563334	5.168341	0.023002178	1	4	11	3	15	1	5	5
Slc2a3	-1.129727	3.92486	4.902237	0.026821928	1	12	8	6	6	5	3	3
Arhgef26	-1.123143	2.956409	4.203688	0.040336174	1	19	19	5	11	6	3	10
Tmem45b	-1.120469	3.232846	4.08749	0.043201749	1	6	8	7	8	6	1	3
Slc26a7	-1.117466	2.764997	4.449965	0.034901952	1	13	63	18	46	26	8	9
Gm38978	-1.115509	1.444557	4.198833	0.040451799	1	32	53	13	27	8	17	16
2900076A(	-1.095626	1.914312	4.136733	0.041962073	1	21	25	7	16	11	6	6
Fxyd6	-1.095568	6.311253	10.01024	0.001556721	0.88998	9	33	21	29	10	11	11
Arhgap31	-1.083514	4.497926	4.083274	0.04330966	1	4	10	17	16	6	5	6
C1qa	-1.070564	3.70672	4.923373	0.026495734	1	10	15	9	16	7	3	8
A530001N	-1.066277	1.049845	4.35845	0.03682579	1	9	27	9	17	9	6	6
Dhdh	-1.061721	3.122685	8.154167	0.004296233	1	26	25	20	20	10	12	11
Cidea	-1.060547	3.843303	5.145289	0.023309535	1	10	15	18	26	9	7	9
Hist1h1c	-1.046683	2.960584	4.540079	0.033110071	1	44	68	35	39	39	13	11
Fmo2	-1.045564	3.004302	4.869651	0.027333029	1	32	21	15	42	14	8	19
Senp3	-1.027998	3.853395	8.20275	0.004182694	1	45	76	26	45	30	18	19
Tubg2	-1.024843	4.790474	4.841684	0.027779751	1	5	13	7	10	5	1	7
LOC10816!	-1.024246	2.294267	6.550246	0.010486967	1	43	40	31	69	18	27	20
Nfatc4	-1.008995	2.469072	8.176806	0.00424294	1	28	37	16	42	15	10	21

Exosc9	-1.008786	2.415114	4.421068	0.035497815	1	16	15	21	24	13	9	6
Cmtm3	-0.990962	2.431937	3.969338	0.046335976	1	8	18	2	24	6	4	9
Cdk5r1	-0.987324	6.182848	7.9592	0.004784354	1	14	21	17	16	9	8	9
Mthfd1l	-0.984696	3.408463	4.278939	0.038587431	1	7	19	7	10	1	2	14
Haus7	-0.971977	2.401448	4.754691	0.029218544	1	17	35	24	39	16	10	18
Gas2	-0.970317	3.625374	4.20043	0.040413738	1	15	21	17	9	10	3	12
Dhx37	-0.968555	2.824659	5.054239	0.024565773	1	31	26	24	16	11	6	24
P3h3	-0.966036	3.05859	3.855064	0.049596034	1	27	34	28	18	17	4	24
Abca8a	-0.965512	6.28091	4.255553	0.039122216	1	9	8	13	3	3	2	8
Synm	-0.960343	8.015117	11.81895	0.00058631	0.632525	23	51	4	40	17	8	20
Nrxn2	-0.959161	7.825502	4.748571	0.029322632	1	12	10	6	8	3	5	6
Smyd5	-0.956052	3.374024	5.048148	0.024652268	1	12	28	18	16	13	8	7
Cntn1	-0.947473	8.846069	5.856787	0.015517097	1	12	14	6	17	6	9	4
Smarcd1	-0.946485	4.170431	9.540264	0.002010129	0.981214	31	26	23	52	14	8	32
Fxyd1	-0.93513	6.543698	4.657747	0.030913727	1	8	14	10	6	9	2	4
Pik3cd	-0.922791	4.698176	5.944814	0.014760645	1	15	20	10	23	11	5	11
Cdca7l	-0.922572	2.440866	4.205877	0.040284137	1	30	42	29	47	22	15	21
C1ra	-0.91671	2.751836	5.463277	0.019420198	1	27	42	20	26	9	16	21
Dgki	-0.906492	6.654007	4.847338	0.027688835	1	7	22	6	7	1	4	12
Cep112	-0.904157	3.529165	8.298405	0.003967992	1	24	26	26	36	17	5	25
Pced1a	-0.899557	4.022551	7.662507	0.005638011	1	45	36	30	45	17	18	30
LOC10263!	-0.89926	2.825797	4.835558	0.027878621	1	47	60	38	58	14	32	36
Rcn3	-0.889002	4.523314	8.831834	0.002960208	1	33	49	45	69	32	21	26
Arhgef11	-0.887411	3.363863	6.538287	0.010557695	1	22	48	24	47	18	11	29
B3galt4	-0.883124	3.098592	4.686815	0.030394941	1	14	39	17	43	15	16	12
Tspyl2	-0.881945	4.355604	7.06859	0.007844697	1	18	40	22	28	19	17	6
Dclre1a	-0.874178	3.780647	7.730614	0.005429229	1	62	100	49	49	31	31	45
Traf3ip1	-0.856414	3.032847	6.418102	0.011296284	1	28	27	18	29	15	10	18
Ltb4r1	-0.854469	3.36368	6.650487	0.009912871	1	66	87	54	60	41	33	35
Aak1	-0.85249	5.144567	4.941175	0.026224204	1	25	20	23	23	8	15	16
Atp1a2	-0.836403	8.523142	4.583748	0.032276537	1	81	70	42	64	25	42	40
Cers4	-0.826826	6.199799	7.969291	0.004757758	1	12	29	25	29	16	6	19
Cp	-0.821562	6.10463	6.919485	0.008526153	1	33	39	23	22	19	18	12
Gnao1	-0.818113	8.948373	4.850863	0.027632309	1	23	25	16	35	12	17	12
Zfp90	-0.807999	3.861119	7.932958	0.004854238	1	50	80	40	58	28	28	42
Gm8995	-0.794181	4.398639	5.665148	0.017305255	1	85	104	59	109	55	53	37
Prune2	-0.788961	9.122257	5.600054	0.01795992	1	26	17	10	22	11	9	13
Iqgap2	-0.784028	4.290936	5.264045	0.021770362	1	47	84	38	57	20	28	53
Cstf2	-0.778865	4.791241	9.307179	0.002282576	1	86	93	77	79	45	41	66
Trub2	-0.77386	4.366531	8.519825	0.003512983	1	45	71	39	84	33	21	53
Ccdc92	-0.772854	7.722905	6.669805	0.009805992	1	28	18	19	33	17	9	18
Brca2	-0.770554	3.551933	4.039749	0.044440438	1	72	114	31	69	43	16	71
Col6a2	-0.767666	5.148259	5.137012	0.023420928	1	143	297	175	121	143	71	107
Mtrf1l	-0.756838	3.457557	5.116807	0.02369518	1	27	35	38	48	19	24	23
Fam73a	-0.753107	6.096133	6.170454	0.012990124	1	20	35	11	17	7	16	14
Fastkd2	-0.751983	3.717427	5.914142	0.01501982	1	36	34	27	27	14	14	30
Mpdu1	-0.751346	3.51732	5.216388	0.022374985	1	68	48	28	63	28	25	41
LOC10524	-0.751056	4.18189	8.103761	0.00441735	1	117	92	95	103	45	54	95
Slc24a3	-0.750838	5.563742	3.850637	0.04972709	1	20	21	5	51	16	9	17
Lrrc17	-0.744564	10.97108	8.117709	0.004383491	1	117	207	112	144	95	68	92
Rnaseh2b	-0.740538	4.373576	8.686665	0.003205466	1	89	150	72	138	66	50	84
Mtss1l	-0.738218	5.324729	5.438186	0.019701118	1	22	19	19	37	15	13	16
Sgtb	-0.736109	5.164139	4.39208	0.036106238	1	11	16	12	20	8	6	13
Plcxd2	-0.727828	5.416823	5.458009	0.019478832	1	24	14	22	33	18	4	22
Dis3l2	-0.711794	3.840535	5.885562	0.015265521	1	59	57	43	62	23	34	47
Dctn1	-0.711772	6.0518	6.525638	0.010633041	1	199	230	125	157	126	82	115
Rel	-0.711214	3.703174	4.24958	0.039260041	1	83	95	55	120	66	37	55
Stk39	-0.708206	7.531603	5.817038	0.01587165	1	28	26	25	34	14	17	22
Chek2	-0.706733	3.494383	5.371696	0.020466065	1	92	99	56	91	29	52	79
Morc4	-0.704247	3.733463	5.784582	0.016167345	1	34	39	22	38	22	14	26
Cd59a	-0.702143	7.789429	13.37174	0.000255445	0.509334	149	318	179	375	164	104	199
Matn4	-0.70008	5.994897	4.480474	0.03428421	1	158	245	154	109	134	50	137
Oaz2	-0.691865	6.805707	13.34148	0.000259599	0.509334	120	181	108	166	78	85	102
Nup98	-0.691832	2.946886	3.922022	0.047657517	1	51	52	26	47	26	29	24
1110012L1	-0.690852	3.538918	4.466876	0.034558115	1	22	46	17	33	20	13	21
Pstk	-0.686097	3.710446	4.793903	0.028560639	1	35	53	36	62	19	21	51
Gas2l3	-0.685117	6.080801	6.481223	0.010901994	1	72	62	77	68	50	25	65
Cyp2j9	-0.678762	4.264795	3.983359	0.045951852	1	16	31	19	35	25	6	16
Wdr60	-0.675436	4.626401	6.299941	0.012074196	1	75	75	62	102	50	45	52
Map1a	-0.672544	10.33004	8.69771	0.003186102	1	36	58	46	38	37	24	23
Kansl1l	-0.672495	4.738701	9.030905	0.002654531	1	151	164	87	192	76	92	107
Ccdc18	-0.671601	3.621202	4.568822	0.032558941	1	99	111	72	112	78	54	45
Sestd1	-0.671306	5.462496	5.144227	0.023323787	1	25	36	19	26	22	11	17
Bach2	-0.669593	4.287879	4.777747	0.028829809	1	29	37	24	53	22	26	17

S100a4	-0.666837	5.38147	5.999012	0.014313891	1	24	63	20	29	22	5	39
Rnf146	-0.665087	3.190881	3.94552	0.046996335	1	31	42	29	25	23	15	23
Mars	-0.664669	3.894934	4.44702	0.034962204	1	69	103	65	85	32	45	82
Rnf215	-0.660582	4.069646	4.709391	0.029998275	1	32	67	32	47	27	30	24
Lsm14b	-0.660025	5.561882	4.251528	0.039215027	1	216	96	57	180	76	73	118
Mtmr9	-0.659141	3.815663	5.447074	0.019601133	1	54	84	30	49	25	24	57
Naip1	-0.654691	4.308703	4.335675	0.03732161	1	73	180	64	105	51	51	99
LOC10263!	-0.652547	3.95134	7.122992	0.007610158	1	44	76	44	76	36	33	45
Dennd4c	-0.648749	3.810576	5.264056	0.021770229	1	89	103	93	102	67	61	55
Ece2	-0.646172	4.494542	6.359139	0.01167782	1	36	47	34	39	22	23	31
Gsdmd	-0.642947	2.362274	4.076989	0.043471051	1	23	40	23	37	19	20	19
Pitrm1	-0.639371	4.173496	5.276008	0.021621271	1	50	76	31	74	32	41	33
Utp20	-0.633775	4.632107	6.847199	0.008878036	1	137	155	91	153	81	73	106
Ttll4	-0.632131	3.096305	3.902414	0.048216778	1	51	77	44	34	32	22	50
Slc16a1	-0.625058	5.682956	5.926557	0.014914351	1	34	31	13	52	10	16	39
Hemk1	-0.619927	3.649569	4.065249	0.043774242	1	39	44	30	40	25	28	20
Vars2	-0.619249	4.5363	6.054935	0.013867487	1	98	110	77	100	66	37	94
Slc4a1ap	-0.614905	5.436537	12.8561	0.00033638	0.526158	152	202	114	191	79	104	142
Trmt1	-0.609874	4.899097	7.803116	0.005215621	1	102	128	50	84	61	44	74
Copg2	-0.609572	4.662087	6.201098	0.012767111	1	143	175	112	176	98	71	136
Ccdc112	-0.602227	4.511569	5.44408	0.019634755	1	63	93	60	62	45	34	62
Kbtbd11	-0.595679	5.002706	6.262085	0.012334898	1	72	65	49	82	42	28	69
Cap1	-0.593544	5.471359	7.548758	0.006005144	1	201	256	134	221	162	104	125
Anln	-0.585492	4.610141	3.96723	0.046394034	1	118	184	114	193	104	51	165
LOC10105!	-0.583494	3.990286	4.534448	0.033219168	1	145	139	94	170	52	106	117
Sema3b	-0.581105	6.56736	5.712368	0.016845801	1	21	49	18	49	14	22	33
B230206H(	-0.571895	5.138302	4.156819	0.0414672	1	24	74	23	28	18	14	45
BC005537	-0.569954	6.20829	10.71853	0.00106068	0.719982	213	287	156	270	118	151	198
Asap2	-0.567377	4.815517	6.586766	0.010273966	1	148	191	119	164	102	103	103
Fam213a	-0.567049	5.517966	6.031953	0.014049181	1	39	71	49	70	44	26	47
2810006K2	-0.566127	4.226647	4.826865	0.028019534	1	33	71	38	79	26	29	59
Map1b	-0.565117	12.64239	5.587258	0.018091591	1	98	153	77	132	76	68	84
Bud13	-0.562446	3.709329	5.660544	0.017350744	1	47	85	58	92	47	35	63
Clcc1	-0.562031	4.656005	6.547561	0.010502801	1	121	157	92	156	79	62	135
Hist1h1e	-0.559398	6.244754	5.8548	0.015534619	1	511	731	426	437	406	309	325
Ncoa7	-0.558067	7.911115	4.084641	0.043274635	1	23	28	38	25	22	15	23
Cdc25b	-0.557965	3.745875	4.521795	0.033465694	1	80	80	74	90	44	46	84
Maoa	-0.552277	5.553135	9.317795	0.002269389	1	89	99	60	119	66	44	80
Ccdc34	-0.552266	6.068548	7.616469	0.005783761	1	239	345	232	307	221	144	206
Ppargc1b	-0.551443	4.939043	4.299028	0.038134167	1	69	107	55	72	31	47	82
Dgat1	-0.547284	4.354789	6.443619	0.011135156	1	69	71	67	101	54	43	64
Nufip1	-0.543619	4.14796	4.239041	0.03950447	1	59	76	42	62	33	31	63
Ggact	-0.539955	5.739192	4.694746	0.03025497	1	56	130	69	82	61	43	69
Ndrg4	-0.527789	9.467668	4.136967	0.041956269	1	32	53	24	51	24	17	44
Pdzd11	-0.527659	5.584146	9.779172	0.001764998	0.920255	150	217	129	175	122	100	124
Rabl3	-0.524585	3.923256	3.947007	0.046954812	1	28	41	26	48	22	18	36
Taf7	-0.524396	4.634184	6.027582	0.014084013	1	65	80	53	94	34	38	87
Wdr13	-0.520526	5.89094	7.178496	0.007378253	1	151	218	129	211	97	117	157
Fam83b	-0.520287	4.016422	4.284136	0.03846962	1	104	183	114	154	100	85	99
Lmbrd2	-0.518298	5.969409	5.822525	0.015822217	1	97	192	82	138	93	49	128
Cep89	-0.514367	3.761765	5.099372	0.023934502	1	72	81	53	93	55	33	73
Tamm41	-0.513871	4.346171	3.86033	0.049440615	1	55	103	36	82	26	42	79
Eif1b	-0.509847	7.954032	7.541539	0.006029251	1	83	132	89	110	66	52	107
Nr2c2	-0.506157	5.815329	7.652295	0.005670016	1	154	217	108	195	92	121	137
Slc35c2	-0.498616	5.196567	4.946347	0.026145854	1	87	125	103	174	74	93	87
Gm10516	-0.484943	3.740162	4.127382	0.042194583	1	54	62	33	67	40	33	42
Bcs1l	-0.481712	4.360481	4.626864	0.031475047	1	50	94	62	72	44	41	67
Pik3r2	-0.477468	5.430861	8.712154	0.003160955	1	173	261	142	205	111	101	225
Mrpl16	-0.476071	5.087278	5.702249	0.016943188	1	83	136	60	152	73	57	101
Crybg3	-0.475013	6.512589	11.05838	0.000882873	0.679882	666	813	496	771	460	440	573
Ndufaf2	-0.472227	5.109978	5.532414	0.01866733	1	74	102	45	85	55	38	74
Zfhx2	-0.468958	5.312342	5.815999	0.015881029	1	78	110	68	109	62	50	89
Abcf2	-0.467914	5.014807	5.950174	0.014715832	1	101	108	64	102	69	63	70
Pds5b	-0.467643	6.236988	7.374572	0.006615255	1	162	212	172	205	122	139	146
Nek4	-0.467184	5.137966	5.580092	0.018165771	1	89	120	74	131	48	74	106
Ogfod1	-0.464704	4.834745	4.022715	0.044891402	1	96	101	51	86	58	49	76
Smim13	-0.459837	6.171104	4.731093	0.029622062	1	84	74	34	74	44	30	76
Tnks	-0.458392	5.426166	4.352681	0.036950734	1	109	138	65	116	65	83	79
Papolg	-0.452991	4.354741	4.462471	0.034647344	1	123	131	66	112	54	65	127
Atf5	-0.452375	6.008625	4.845818	0.027713248	1	170	187	99	119	107	81	132
Cdkal1	-0.451455	4.819207	4.242333	0.039427955	1	89	165	135	178	105	96	106
Tcof1	-0.450782	6.014039	8.174012	0.00424948	1	192	305	210	298	167	141	257
Golim4	-0.449417	6.767376	8.046351	0.004559541	1	395	497	303	377	319	239	295
Znhit6	-0.447956	5.371429	7.904267	0.004931833	1	165	258	146	223	121	130	186

Vipas39	-0.447241	5.2917	5.074813	0.02427589	1	96	123	75	120	63	57	115
Jmjd1c	-0.446266	6.626456	9.036222	0.00264682	1	561	794	458	621	409	383	551
Dtd1	-0.445946	4.975229	5.014284	0.025139029	1	77	90	63	104	66	37	86
Alg11	-0.443907	5.368718	5.219924	0.022329532	1	85	157	84	177	95	73	104
Rbm10	-0.442249	5.496647	6.263399	0.012325748	1	179	248	104	193	126	110	159
Maf	-0.441628	7.197622	5.425961	0.019839514	1	69	92	48	84	51	44	68
Rab11fip2	-0.439355	6.325878	9.014393	0.002678618	1	190	211	161	233	135	146	158
Tenm3	-0.435002	5.682748	4.080931	0.043369756	1	87	115	103	82	69	73	75
Agtpbp1	-0.430124	7.57473	5.551015	0.018469976	1	72	99	42	100	56	43	76
Nfkbib	-0.422779	5.835155	6.493994	0.010823953	1	130	236	153	252	126	118	191
Git1	-0.419121	5.777417	4.235378	0.039589813	1	87	160	94	135	63	53	169
Uimc1	-0.411973	4.783683	4.283126	0.038492502	1	97	191	106	159	71	87	162
Lnp	-0.411203	5.793984	5.251812	0.021923927	1	85	107	63	109	72	60	72
Plcb3	-0.403692	6.045386	4.419193	0.03553683	1	216	303	157	191	172	112	216
Klc2	-0.401656	7.232528	3.911893	0.047945551	1	65	125	46	82	66	51	58
Dmxl1	-0.400058	5.756127	4.224188	0.039851675	1	208	227	170	198	135	121	221
Ttc4	-0.399588	5.397153	4.82935	0.027979176	1	107	174	116	117	89	71	143
Cdadc1	-0.395995	5.88978	5.407333	0.020052328	1	155	214	119	188	104	110	177
Peli2	-0.392871	5.720375	5.007916	0.025231657	1	119	201	93	162	98	71	167
Cd63	-0.392374	7.375539	4.220667	0.039934451	1	194	267	156	189	143	159	146
Ncoa5	-0.391617	5.057361	4.21621	0.040039495	1	126	172	84	138	86	79	136
Slc25a28	-0.390849	4.725609	4.045954	0.044277357	1	72	99	62	96	55	61	72
Cpq	-0.389714	4.360329	4.543104	0.033051604	1	111	133	90	109	87	59	115
Pum3	-0.389398	6.460343	9.237641	0.002370902	1	283	397	216	340	200	196	321
Tmem256	-0.388924	6.461298	5.725373	0.016721484	1	180	336	174	383	217	144	243
Farsa	-0.384053	6.346672	5.07852	0.024224033	1	302	409	175	387	201	201	328
Tfe3	-0.381101	5.015999	4.598971	0.03199115	1	89	113	66	92	58	66	85
Dffa	-0.379522	6.229016	5.20181	0.022563377	1	92	134	86	123	79	65	111
Nsrp1	-0.379322	6.461606	5.566487	0.018307457	1	343	507	295	424	313	242	342
Cast	-0.377144	8.755898	5.901408	0.015128788	1	2223	3319	1883	2855	2025	1741	1969
Aarsd1	-0.372794	5.503402	5.075011	0.02427312	1	108	175	86	159	88	91	125
Zmym4	-0.363922	5.659438	5.732577	0.016653025	1	165	259	164	263	179	111	211
Atp11b	-0.360898	6.237763	4.707673	0.030028269	1	323	476	306	417	245	303	328
Snrnp48	-0.358701	6.523424	3.968538	0.046358006	1	268	405	286	318	207	228	327
BC004004	-0.357288	6.222737	4.874824	0.027251216	1	220	303	183	274	169	165	244
Chd8	-0.356703	6.830674	4.196657	0.040503759	1	352	543	337	436	300	258	437
Srcap	-0.356559	5.144885	3.877542	0.048936156	1	175	194	125	150	125	106	151
Tmed2	-0.355556	6.785883	5.425723	0.019842221	1	436	712	310	602	388	310	489
Uxs1	-0.348398	5.682707	4.091751	0.043092975	1	106	142	109	150	94	73	141
Zc3h13	-0.347853	7.144206	5.030484	0.024904931	1	469	777	433	564	389	409	506
Dohh	-0.345265	6.093824	4.242181	0.03943149	1	130	222	132	166	109	109	171
Tecr	-0.34428	8.945805	6.825543	0.008986323	1	926	1233	839	1479	959	592	1123
Tmem63b	-0.335984	6.874256	4.747622	0.029338815	1	201	244	144	234	161	140	186
Nbea	-0.335783	5.770799	4.05747	0.043976329	1	272	312	210	314	190	213	254
Atxn1	-0.333562	5.931666	4.412659	0.0356732	1	163	222	167	260	120	102	296
Smap2	-0.331799	6.261361	4.334515	0.037347055	1	98	170	91	126	107	81	97
Prrc2a	-0.331653	6.972171	5.454705	0.019515698	1	448	635	318	516	389	346	365
Rhog	-0.32838	5.549142	4.7265	0.029701275	1	194	346	152	261	177	168	210
Akt3	-0.328299	6.472965	4.776876	0.028844398	1	133	173	105	161	114	92	138
Stx4a	-0.327644	6.121734	3.919079	0.047741039	1	250	324	200	338	190	215	251
Nrp1	-0.324577	6.896003	5.410397	0.020017162	1	163	257	127	204	153	117	178
Stim2	-0.323001	6.038974	4.155088	0.041509612	1	164	204	131	240	133	121	194
Tars	-0.322089	6.085929	3.895093	0.048427358	1	309	370	166	320	196	175	343
Cep350	-0.318206	6.65204	4.654652	0.030969505	1	469	655	395	542	406	357	467
Golgb1	-0.316706	7.695402	5.747019	0.016516656	1	862	1091	673	963	665	636	859
Map2k7	-0.315518	6.013556	4.681349	0.030491797	1	184	203	147	228	151	117	201
Nsd1	-0.31545	7.61032	6.17632	0.012947125	1	771	1027	611	965	595	621	802
Mlec	-0.314819	7.42985	5.386102	0.020297769	1	447	742	346	488	375	308	545
Rassf3	-0.313506	6.463374	6.00666	0.014251977	1	393	507	274	456	328	268	381
Cpeb2	-0.304627	6.55577	4.947853	0.026123087	1	401	500	284	455	300	308	377
Tnks1bp1	-0.301909	6.487916	4.216783	0.040025978	1	425	594	332	417	336	352	361
Kansl1	-0.298903	5.617414	4.028833	0.04472888	1	156	252	185	206	136	145	217
Nav1	-0.297682	7.493915	5.787991	0.016136023	1	295	375	247	299	214	201	350
Synj1	-0.297559	7.745005	4.683057	0.0304615	1	143	199	137	203	111	134	175
Rfk	-0.295515	6.821858	4.323688	0.037585379	1	271	242	163	323	206	143	276
Rsrc2	-0.288915	7.597108	4.099635	0.042892473	1	652	1064	565	855	563	529	838
Ankrd11	-0.288123	8.92673	4.547042	0.032975674	1	1626	2131	1301	1695	1248	1185	1760
Ubr5	-0.287714	7.214105	5.205026	0.022521684	1	562	805	492	641	477	441	623
Rc3h2	-0.286219	6.747439	4.707809	0.030025893	1	298	414	244	418	269	228	349
Ndufb10	-0.28539	6.980168	4.09247	0.043074647	1	256	343	240	357	235	203	305
Smarca5	-0.280614	6.598426	5.271706	0.021674764	1	397	659	282	532	344	285	529
Dnttip2	-0.280428	5.969424	4.425048	0.035415114	1	216	317	162	329	222	139	274
Appl1	-0.276113	7.16362	4.514212	0.033614375	1	342	520	274	511	281	277	471
Ralgapa1	-0.274573	7.263433	4.420876	0.03550181	1	246	382	222	299	174	218	333

Ash1l	-0.271494	8.049575	6.105575	0.013475613	1	981	1395	807	1355	821	776	1243
Rsl1d1	-0.267604	6.495591	4.001171	0.045468658	1	303	516	265	445	261	269	425
C2cd2	-0.264275	6.327821	4.280402	0.038554239	1	361	521	267	558	352	242	479
Eea1	-0.263685	8.05712	3.86682	0.049249763	1	600	970	525	704	528	480	748
Prdm2	-0.260171	6.742872	4.002103	0.045443532	1	368	428	296	442	310	264	398
Fndc3a	-0.259975	6.549559	4.054845	0.044044763	1	513	709	367	609	373	375	652
Slk	-0.254793	9.074764	4.286586	0.038414231	1	3020	4475	2524	3516	2548	2594	3239
Dnajc21	-0.245101	7.344042	4.433665	0.035236775	1	817	930	600	862	625	590	832
Rexo2	-0.243777	7.378526	4.060813	0.043889353	1	335	488	218	399	240	227	467
Stx12	-0.226439	7.144286	4.764586	0.029051046	1	315	414	250	382	238	260	386

Table S2. Differentially expressed genes in the cornea of MS-CKO vs age- and sex-matched WT control mice.

	log2FC	logCPM	LR	PValue	FDR	WT1	WT2	Wt3	Wt4	WT5	MS-CKO1	MS-CKO2	MS-CKO3	MS-CKO4	MS-CKO5
**Upregulated genes**															
**Gm16299**	5.18	-0.1353	11.664	0.0006	0.1658	0	0	0	0	0	2	14	12	0	0
**Tssk2**	4.962	-0.3226	11.2	0.0008	0.1805	0	0	0	0	0	0	16	0	2	5
**Snora62**	4.918	-0.5139	5.4763	0.0193	0.6697	0	0	0	0	0	0	23	3	0	0
**Myo15b**	4.7816	-0.3822	9.1479	0.0025	0.3165	0	0	0	0	0	5	8	0	0	7
**Cox16**	4.7704	0.1935	13.81	0.0002	0.0895	0	0	0	0	0	0	2	0	6	7
**Mir7051**	4.7029	-0.4897	6.9191	0.0085	0.5048	0	0	0	0	0	5	7	6	0	1
**Gm40941**	4.6819	-0.4631	7.4628	0.0063	0.4478	0	0	0	0	0	0	16	5	0	0
**Gm30248**	4.4798	-0.7259	4.8135	0.0282	0.7493	0	0	0	0	0	5	0	10	0	0
**Fscn2**	4.44	-0.1479	12.048	0.0005	0.151	0	0	0	0	0	5	0	4	2	2
**Gm33655**	4.4213	0.0683	8.3873	0.0038	0.3726	0	0	0	0	0	0	13	0	0	4
**Fam83c**	4.3809	-0.6616	6.4777	0.0109	0.5486	0	0	0	0	0	4	11	0	0	1
**Gstp2**	4.3755	-0.4826	12.432	0.0004	0.1332	0	0	0	0	0	1	5	4	1	3
**Gm30938**	4.3646	0.1827	12.974	0.0003	0.1116	0	0	0	0	0	2	10	0	0	3
**Gm39957**	4.3608	-0.6659	4.4132	0.0357	0.811	0	0	0	0	0	2	15	0	0	0
**Prss30**	4.318	-0.4614	7.432	0.0064	0.4537	0	0	0	0	0	0	13	0	0	3
**Atoh7**	4.2839	0.0119	8.9265	0.0028	0.3251	0	0	0	0	0	0	16	0	0	0
**LOC10816[illegible]**	4.2812	-0.3886	9.3292	0.0023	0.3028	0	0	0	0	0	1	0	1	6	1
**Pla2g4e**	4.2453	1.6599	14.402	0.0001	0.0808	0	0	0	0	0	7	0	0	0	5
**LOC10816[illegible]**	4.2411	-0.791	5.2956	0.0214	0.6958	0	0	0	0	0	0	2	0	6	0
**Sec31b**	4.2221	-0.6785	5.1708	0.023	0.7051	0	0	0	0	0	5	9	0	0	0
**Gm39662**	4.2188	-0.8474	3.8548	0.0496	0.8726	0	0	0	0	0	0	0	0	5	3
**Atp2b3**	4.2171	5.2153	17.36	3E-05	0.0412	0	0	0	0	0	0	5	5	2	0
**LOC10816[illegible]**	4.2117	-0.3946	4.5951	0.0321	0.7798	0	0	0	0	0	0	0	7	3	0
**Gbp6**	4.1736	-0.2499	10.579	0.0011	0.2213	0	0	0	0	0	1	1	7	2	0
**Ptpn5**	4.1711	1.4578	14.238	0.0002	0.0813	0	0	0	0	0	0	13	0	0	0
**Myh2**	4.1289	-0.1602	8.345	0.0039	0.3742	0	0	0	0	0	3	3	6	0	0
**H2-Bl**	4.1254	-0.6321	4.1755	0.041	0.8353	0	0	0	0	0	9	0	0	1	0
**Lep**	4.1253	-0.6246	5.898	0.0152	0.6148	0	0	0	0	0	1	11	0	1	0
**F420015M[illegible]**	4.1184	0.0769	12.983	0.0003	0.1116	0	0	0	0	0	0	6	6	0	0
**Lgr5**	4.1171	3.0689	15.26	9E-05	0.0631	0	0	0	0	0	5	1	0	1	4
**Gm41074**	4.11	-0.8136	3.9761	0.0462	0.8611	0	0	0	0	0	0	0	8	2	0
**Gm10653**	4.1074	-0.3827	12.023	0.0005	0.151	0	0	1	0	0	7	17	4	0	2
**Grid1**	4.1057	3.0743	15.677	8E-05	0.055	0	0	0	0	0	5	0	4	0	2
**Gm2164**	4.1045	-0.5022	7.2884	0.0069	0.4711	0	0	0	0	0	0	12	0	1	0
**A3galt2**	4.1031	3.9938	15.752	7E-05	0.055	0	0	0	0	0	2	0	0	0	9
**LOC10816[illegible]**	4.0996	-0.8468	3.8517	0.0497	0.8726	0	0	0	0	0	0	0	0	5	2
**Gm39786**	4.0922	-0.8088	4.1545	0.0415	0.8377	0	0	0	0	0	0	0	0	5	2
**Gm12257**	4.0582	-0.4003	3.9336	0.0473	0.8678	0	0	0	0	0	0	8	5	0	0
**Amhr2**	4.0474	0.6646	13.413	0.0002	0.1045	0	0	0	0	0	0	4	5	0	2
**Gm34921**	4.0348	-0.5752	5.9623	0.0146	0.6057	0	0	0	0	0	1	0	9	0	1
**Gm39798**	4.0154	-0.6941	6.1786	0.0129	0.5851	0	0	0	0	0	2	7	3	0	0
**Nlrp1c-ps**	4.0068	-0.4974	4.2488	0.0393	0.8224	0	0	0	0	0	0	0	11	0	0
**Gm16168**	3.9941	1.5047	13.291	0.0003	0.1078	0	0	0	0	0	0	10	1	0	0
**4930547M**	3.9902	-0.7761	5.5987	0.018	0.6481	0	0	0	0	0	1	0	6	2	0
**Tex36**	3.981	-0.8304	3.8963	0.0484	0.8678	0	0	0	0	0	0	0	5	3	0
**Megf10**	3.9715	2.9754	14.447	0.0001	0.0808	0	0	0	0	0	0	0	3	0	7
**Gm39567**	3.9683	-0.7674	4.0521	0.0441	0.8538	0	0	0	0	0	0	12	1	0	0
**C130083M**	3.9656	-0.1511	12.134	0.0005	0.1486	0	0	0	0	0	3	1	2	0	4
**Gabrd**	3.9569	0.166	11.111	0.0009	0.1872	0	0	0	0	0	0	0	2	0	8
**Pcdhb13**	3.9453	-0.2687	8.1263	0.0044	0.3914	0	0	0	0	0	0	11	0	0	1
**Gm32143**	3.9441	-0.6936	4.8937	0.027	0.7403	0	0	0	0	0	5	6	0	0	0
**LOC10263[illegible]**	3.9435	-0.5098	8.3866	0.0038	0.3726	0	0	0	0	0	0	4	0	3	2
**Atf7ip2**	3.9219	-0.7568	5.1272	0.0236	0.7109	0	0	0	0	0	5	0	1	2	0
**Slc6a20b**	3.9213	-0.5885	6.8586	0.0088	0.5121	0	0	0	0	0	1	0	7	0	2

**Gm38506**	3.91	-0.2032	6.4962	0.0108	0.5486	0	0	0	0	0	0	0	6	0	4
**BC049715**	3.9031	0.3025	10.348	0.0013	0.2316	0	0	0	0	0	0	10	1	0	0
**Gm36745**	3.9007	-0.6591	5.3504	0.0207	0.6895	0	0	0	0	0	0	5	6	0	0
**Gm41253**	3.8732	0.4733	9.8235	0.0017	0.261	0	0	0	0	0	0	11	0	0	0
**Gm3740**	3.8679	-0.3044	7.6448	0.0057	0.434	0	0	0	0	0	0	6	3	1	0
**LOC108167**	3.8641	0.0445	9.5436	0.002	0.2787	0	0	0	0	0	4	6	0	0	0
**Gm35437**	3.8537	-0.6719	5.3084	0.0212	0.6958	0	0	0	0	0	6	0	0	0	3
**Gareml**	3.8523	-0.2918	7.2484	0.0071	0.4745	0	0	0	0	0	6	0	3	0	0
**Gm33032**	3.849	-0.8326	3.9736	0.0462	0.8611	0	0	0	0	0	6	0	0	0	3
**Snora78**	3.8395	-0.7087	6.3823	0.0115	0.5577	0	0	0	0	0	4	5	1	0	0
**Gm39318**	3.8361	-0.4777	8.9439	0.0028	0.3247	0	0	0	0	0	3	0	0	0	6
**Gm10419**	3.829	4.6141	13.027	0.0003	0.1116	0	0	0	0	0	0	0	7	0	2
**Wasf1**	3.8287	3.9036	13.059	0.0003	0.1116	0	0	0	0	0	4	4	0	1	0
**Gm38907**	3.8265	-0.7775	5.6108	0.0179	0.6454	0	0	0	0	0	4	4	0	1	0
**Heyl**	3.8119	2.5914	12.523	0.0004	0.1315	0	0	0	0	0	4	5	0	0	0
**Rab39**	3.809	3.401	12.813	0.0003	0.1168	0	0	0	0	0	0	8	1	0	0
**Gm1043**	3.8087	1.9661	11.89	0.0006	0.1538	0	0	0	0	0	0	4	4	1	0
**Lta**	3.7952	-0.4812	4.6579	0.0309	0.7726	0	0	0	0	0	3	0	6	0	0
**Gm36847**	3.7939	-0.6907	6.075	0.0137	0.6008	0	0	0	0	0	3	1	0	0	5
**2610005L0**	3.7927	-0.3012	5.8805	0.0153	0.6176	0	0	0	0	0	0	0	4	3	0
**LOC105244**	3.772	-0.2775	6.008	0.0142	0.604	0	0	0	0	0	0	11	0	0	0
**Gm34802**	3.7623	-0.0137	7.9766	0.0047	0.4019	0	0	0	0	0	0	1	7	0	1
**Gm30316**	3.7433	-0.2657	7.2661	0.007	0.4716	0	0	2	0	0	9	20	0	1	5
**LOC108167**	3.7223	-0.4118	6.6677	0.0098	0.5335	0	0	0	0	0	0	0	5	1	2
**Gm33235**	3.7179	-0.6406	4.8441	0.0277	0.747	0	0	0	0	0	0	7	0	0	3
**AI662270**	3.7062	1.4526	10.389	0.0013	0.2316	0	0	0	0	0	2	0	5	1	0
**Snord55**	3.6857	-0.3637	6.3442	0.0118	0.5589	0	0	0	0	0	4	0	0	0	4
**Gm11110**	3.6804	-0.4427	7.9631	0.0048	0.4019	0	0	0	0	0	0	5	3	0	1
**4930483J1**	3.6761	-0.7289	5.0312	0.0249	0.7219	0	0	0	0	0	1	9	0	0	0
**Gm41349**	3.6622	0.1149	8.9623	0.0028	0.324	0	0	0	0	0	1	8	0	0	0
**Gm34825**	3.662	-0.8678	5.3512	0.0207	0.6895	0	0	0	0	0	1	0	0	3	2
**Gm26691**	3.6406	0.0909	7.4689	0.0063	0.4472	0	0	0	0	0	0	0	3	0	5
**Gm41061**	3.6244	-0.7448	3.9871	0.0459	0.8611	0	0	0	0	0	3	6	0	0	0
**5830444B0**	3.6165	-0.1605	5.7317	0.0167	0.6337	0	0	0	0	0	0	0	5	0	3
**Fv1**	3.6147	-0.7462	4.8721	0.0273	0.7445	0	0	0	0	0	0	5	4	0	0
**Gm35125**	3.6084	-0.1226	7.501	0.0062	0.4459	0	0	0	0	0	0	0	0	4	2
**Gm29826**	3.6077	-0.7041	4.2088	0.0402	0.8291	0	0	0	0	0	0	0	3	0	5
**4921511C1**	3.6061	-0.8332	4.0247	0.0448	0.8574	0	0	0	0	0	0	0	3	0	5
**Gm32835**	3.6011	-0.7218	4.5773	0.0324	0.7817	0	0	0	0	0	4	0	0	2	0
**F630042J0**	3.5949	-0.6375	6.8911	0.0087	0.5068	0	0	0	0	0	3	0	2	1	1
**Fndc7**	3.5837	-0.7215	7.206	0.0073	0.4784	0	0	0	0	0	2	0	0	1	4
**2810039B1**	3.5808	-0.4374	6.536	0.0106	0.5456	0	0	0	0	0	2	0	4	1	0
**Galnt6**	3.5777	1.7007	10.035	0.0015	0.2523	0	0	0	0	0	0	8	0	0	0
**Zmynd12**	3.5754	-0.4704	5.3108	0.0212	0.6958	0	0	0	0	0	0	7	0	0	2
**Olfml2a**	3.5738	-0.3507	6.5382	0.0106	0.5456	0	0	0	0	0	0	0	0	5	0
**Rhoh**	3.561	-0.4681	6.9239	0.0085	0.5048	0	0	0	0	0	1	0	5	1	0
**Gm34360**	3.5584	-0.3611	6.7047	0.0096	0.525	0	0	0	0	0	0	9	0	0	0
**Platr10**	3.5462	-0.8373	3.8996	0.0483	0.8678	0	0	0	0	0	0	7	0	0	2
**Hormad2**	3.5441	-0.2612	6.1026	0.0135	0.5973	0	0	0	0	0	6	0	0	0	1
**Cort**	3.5394	-0.7396	4.001	0.0455	0.8587	0	0	0	0	0	6	0	0	0	1
**AV039307**	3.5386	0.0422	7.9397	0.0048	0.4028	0	0	0	0	0	0	0	5	1	1
**Pcdh20**	3.5303	-0.1713	5.8058	0.016	0.628	0	0	0	0	0	0	9	0	0	0
**4931403E2**	3.5199	-0.3285	6.0901	0.0136	0.6001	0	0	0	0	0	0	4	0	0	4
**Gm36440**	3.5184	-0.4204	6.195	0.0128	0.5841	0	0	0	0	0	4	0	0	0	3
**Gm31348**	3.5182	-0.5556	4.375	0.0365	0.8112	0	0	0	0	0	5	0	2	0	0
**1700080G1**	3.5139	-0.8168	3.8452	0.0499	0.8743	0	0	0	0	0	0	7	0	1	0
**Gm39663**	3.5081	-0.6576	5.6611	0.0173	0.6381	0	0	0	0	0	0	4	1	0	3
**Gm11747**	3.5024	-0.7021	3.982	0.046	0.8611	0	0	0	0	0	0	0	2	3	0
**Slc2a13**	3.4947	1.6849	9.1534	0.0025	0.3165	0	0	0	0	0	0	0	0	0	7
**Mpp3**	3.4932	1.2782	8.8956	0.0029	0.3276	0	0	0	0	0	0	0	0	0	7
**Fam71f2**	3.4851	-0.6069	6.1758	0.013	0.5852	0	0	0	0	0	0	5	3	0	0
**Wdr95**	3.4817	-0.5455	4.026	0.0448	0.8574	0	0	0	0	0	0	9	0	0	0

**Gm34296**	3.4785	-0.7225	5.8995	0.0151	0.6148	0	0	0	0	0	2	0	4	0	1
**Tram1l1**	3.4769	3.4826	9.9687	0.0016	0.2567	0	0	0	0	0	1	6	0	0	0
**Dmbx1**	3.4766	-0.5742	6.032	0.014	0.604	0	0	0	0	0	2	0	5	0	0
**Fcgr2b**	3.476	0.2356	7.9862	0.0047	0.4019	0	0	0	0	0	0	0	5	0	2
**Gm28802**	3.4666	-0.7177	3.9083	0.048	0.8678	0	0	0	0	0	0	0	0	4	0
**Gm33723**	3.4518	-0.3019	6.5217	0.0107	0.546	0	0	0	0	0	0	0	7	0	0
**Fsd2**	3.4472	-0.259	7.1288	0.0076	0.4871	0	0	0	0	0	0	8	0	0	0
**Gm34050**	3.4439	-0.6899	5.5571	0.0184	0.6548	0	0	0	0	0	0	4	0	1	2
**Gm31042**	3.4427	-0.6628	4.181	0.0409	0.8344	0	0	0	0	0	0	0	0	4	0
**Scgb1c1**	3.4373	-0.7591	4.0014	0.0455	0.8587	0	0	0	0	0	0	0	2	0	5
**Lrp2bp**	3.4294	-0.2798	5.764	0.0164	0.6285	1	0	0	0	0	4	12	0	1	2
**Npas1**	3.427	-0.7078	4.3927	0.0361	0.811	0	0	0	0	0	0	0	6	0	1
**Tfap2d**	3.4237	-0.8696	5.4095	0.02	0.6772	0	0	0	0	0	3	0	2	1	0
**1700088E0**	3.4227	-0.7972	4.8548	0.0276	0.7469	0	0	0	0	0	1	5	0	1	0
**Fam98c**	3.4027	-0.1867	7.8597	0.0051	0.4133	0	0	1	0	0	2	10	3	0	3
**Gpr1**	3.3759	-0.1097	5.1847	0.0228	0.7051	0	0	0	0	0	0	8	0	0	0
**Pdia2**	3.3566	-0.594	4.588	0.0322	0.7798	0	0	0	0	0	0	8	0	0	0
**1700011L2**	3.355	-0.2912	4.6972	0.0302	0.7699	0	0	0	0	0	6	0	0	0	0
**Slc19a3**	3.3495	-0.4569	4.3567	0.0369	0.8131	0	0	0	0	0	0	8	0	0	0
**A430010J1**	3.3459	-0.4626	7.5252	0.0061	0.4459	0	0	0	0	0	1	0	3	1	1
**Gm4737**	3.3403	-0.4991	5.7683	0.0163	0.6285	0	0	0	0	0	5	0	0	0	1
**Siglece**	3.34	-0.5232	5.2746	0.0216	0.6958	0	0	0	0	0	5	0	0	0	1
**Gm35186**	3.3353	-0.6412	3.8695	0.0492	0.8715	0	0	0	0	0	0	8	0	0	0
**Ulbp1**	3.3269	-0.6185	6.2357	0.0125	0.5758	0	0	0	0	0	4	0	0	0	2
**Ms4a4c**	3.3232	-0.5903	5.7839	0.0162	0.6285	0	0	0	0	0	4	0	1	0	1
**Gm32002**	3.3204	-0.7559	4.6395	0.0312	0.7726	0	0	0	0	0	0	0	0	2	3
**Gm36198**	3.3182	-0.4962	4.4032	0.0359	0.811	0	0	0	0	0	0	0	3	2	0
**Gm40139**	3.3139	-0.7075	4.4634	0.0346	0.8027	0	0	0	0	0	2	3	0	1	0
**Ncam2**	3.3118	5.0203	8.7879	0.003	0.3378	0	0	0	0	0	0	0	0	4	2
**Gm27042**	3.3051	-0.588	4.4131	0.0357	0.811	0	0	0	0	0	3	0	0	0	3
**Fsd1**	3.2994	4.6003	8.7176	0.0032	0.3385	0	0	0	0	0	5	0	0	0	1
**Dlgap3**	3.2968	4.2569	8.7715	0.0031	0.3382	0	0	0	0	0	0	0	3	1	2
**Il12rb1**	3.296	-0.671	4.747	0.0293	0.7602	0	0	0	0	0	3	0	3	0	0
**Kcnj3**	3.2952	4.4333	8.762	0.0031	0.3382	0	0	0	0	0	1	0	0	0	5
**Gm19345**	3.2908	0.9875	7.4775	0.0062	0.4459	0	0	0	0	0	0	0	0	0	6
**Jakmip3**	3.2892	3.5407	8.6853	0.0032	0.3414	0	0	0	0	0	0	2	4	0	0
**Rnd1**	3.2856	0.8026	7.1096	0.0077	0.4899	0	0	0	0	0	1	0	5	0	0
**Slc2a6**	3.2829	3.1957	8.634	0.0033	0.3481	0	0	0	0	0	0	4	2	0	0
**Vstm2b**	3.28	1.438	8.3666	0.0038	0.3742	0	0	0	0	0	0	4	0	1	1
**Msr1**	3.2788	0.4799	7.3864	0.0066	0.4591	0	0	0	0	0	0	0	6	0	0
**Astn1**	3.2754	3.3011	8.5822	0.0034	0.3532	0	0	0	0	0	0	6	0	0	0
**LOC10816§**	3.266	-0.1012	5.986	0.0144	0.6046	0	0	0	0	0	0	0	3	0	3
**Gm34505**	3.2636	0.0253	6.392	0.0115	0.5577	0	0	0	0	0	0	0	6	0	0
**Gm2427**	3.2593	-0.5864	4.1912	0.0406	0.8329	0	0	0	0	0	0	0	0	3	1
**Gm38800**	3.2499	0.0774	5.7301	0.0167	0.6337	0	0	0	0	0	0	7	0	0	0
**Lrrc3b**	3.2495	2.3618	8.1444	0.0043	0.3895	0	0	0	0	0	0	5	0	0	1
**Gm32316**	3.2463	-0.413	5.3107	0.0212	0.6958	0	0	0	0	0	0	0	6	0	0
**LOC10816§**	3.2416	-0.3743	4.9546	0.026	0.7335	0	0	1	0	0	0	12	0	1	3
**Aim2**	3.2332	0.5513	6.9125	0.0086	0.5048	0	0	0	0	0	2	0	0	3	0
**Asb5**	3.2286	-0.3253	4.1615	0.0414	0.8377	0	0	0	0	0	0	0	6	0	0
**Gm35623**	3.2167	1.1176	11.531	0.0007	0.171	0	0	0	1	0	0	8	6	1	0
**4930528A1**	3.2084	-0.2533	4.7076	0.03	0.7694	0	0	0	0	0	0	7	0	0	0
**Dnali1**	3.1968	-0.6013	5.9006	0.0151	0.6148	0	0	0	0	0	0	1	1	2	1
**Gm32724**	3.1938	-0.4279	4.3169	0.0377	0.8131	0	0	0	0	0	0	7	0	0	0
**Ttbk1**	3.1844	4.9043	14.193	0.0002	0.0813	0	1	0	0	0	0	13	1	0	0
**Pcdhgb7**	3.1724	-0.326	5.1103	0.0238	0.7149	0	1	0	0	0	0	0	1	5	4
**LOC10264(**	3.1659	-0.7033	4.0128	0.0452	0.8577	0	0	0	0	0	0	0	4	1	0
**Gm33025**	3.1568	-0.5876	5.0642	0.0244	0.7207	0	0	0	0	0	0	0	1	1	3
**Mrgprf**	3.146	0.8806	8.0911	0.0044	0.3937	0	0	0	1	0	1	0	11	0	2
**Kdm4d**	3.1394	0.0963	6.1314	0.0133	0.5913	0	0	0	0	0	0	5	0	0	1
**Gm3739**	3.1352	-0.7422	4.9566	0.026	0.7335	0	0	0	0	0	1	4	0	0	1
**Nron**	3.1293	-0.2964	4.1663	0.0412	0.8377	0	0	0	0	1	0	19	0	0	1

**Gabrg3**	3.1222	-0.2249	6.2521	0.0124	0.5751	0	0	0	0	0	0	6	0	0	0
**Gm34205**	3.1198	-0.6701	4.5446	0.033	0.7852	0	0	0	0	0	5	0	0	0	0
**Cfap54**	3.1183	0.0408	5.0512	0.0246	0.7214	0	0	0	0	0	5	0	0	0	0
**Lbx2**	3.1173	-0.3939	5.14	0.0234	0.7084	0	0	0	0	0	0	0	0	3	1
**Efcab9**	3.1171	-0.2756	5.2685	0.0217	0.6963	0	0	0	0	0	5	0	0	0	0
**Gm42232**	3.1161	-0.7733	3.899	0.0483	0.8678	0	0	0	0	0	1	0	1	2	0
**Dcdc2a**	3.1139	0.9179	6.7618	0.0093	0.5214	0	0	0	0	0	2	0	0	1	2
**Mdh1b**	3.1026	-0.0621	5.9026	0.0151	0.6148	0	0	0	0	0	0	6	0	0	0
**Sycp1-ps1**	3.1018	-0.5768	10.005	0.0016	0.2542	0	1	0	0	0	2	6	2	2	1
**2610305D1**	3.0967	0.9985	5.7656	0.0163	0.6285	0	0	0	0	0	4	0	1	0	0
**Ano2**	3.0818	-0.4237	5.6547	0.0174	0.6381	0	0	2	0	0	3	8	0	6	0
**Hpca**	3.0775	4.9361	7.0708	0.0078	0.4937	0	0	0	0	0	5	0	0	0	0
**Adamts16**	3.0692	0.1145	6.8731	0.0088	0.5102	0	0	0	0	0	2	0	1	0	2
**Morn5**	3.0658	0.1242	6.2371	0.0125	0.5758	0	0	0	0	0	1	0	0	0	4
**Pirt**	3.0632	8.0307	7.341	0.0067	0.4636	0	0	0	0	0	0	0	0	2	3
**Carm1**	3.0623	-0.5752	4.3484	0.037	0.8131	0	0	0	0	0	0	5	0	0	1
**Aard**	3.0608	2.3507	7.1818	0.0074	0.4817	0	0	0	0	0	0	0	1	0	4
**Plekhd1**	3.0599	5.4074	7.2703	0.007	0.4716	0	0	0	0	0	0	0	5	0	0
**Grik3**	3.059	1.9567	7.1626	0.0074	0.4834	0	0	0	0	0	0	0	5	0	0
**Chrna2**	3.059	-0.7035	4.9512	0.0261	0.7335	0	0	0	0	0	1	3	0	1	0
**Snord123**	3.0589	-0.4403	5.0698	0.0243	0.7207	0	0	0	0	0	0	6	0	0	0
**Gm41858**	3.0578	-0.4462	4.387	0.0362	0.811	0	0	2	0	0	0	8	4	3	4
**Gm37915**	3.0535	-0.4715	4.2219	0.0399	0.8261	0	0	0	0	0	0	5	1	0	0
**Tpsb2**	3.0533	1.0631	5.7592	0.0164	0.6285	0	0	0	0	0	0	0	0	4	0
**Tdrd6**	3.0523	0.2411	6.6297	0.01	0.5402	0	0	0	0	0	2	1	2	0	0
**Gm35850**	3.0493	0.0592	5.9886	0.0144	0.6045	0	0	0	0	0	0	0	2	0	3
**Tldc2**	3.0481	0.9672	6.3463	0.0118	0.5589	0	0	0	0	0	0	0	5	0	0
**LOC10816£**	3.0456	-0.3273	5.7988	0.016	0.6285	0	0	0	0	0	2	1	0	0	2
**AA465934**	3.0396	-0.6088	4.6753	0.0306	0.7712	0	0	0	0	0	0	6	0	0	0
**Cfap74**	3.0385	0.4924	5.6433	0.0175	0.6392	0	0	0	0	0	0	0	4	0	1
**Spag8**	3.0382	-0.7578	4.4534	0.0348	0.8037	0	0	0	0	0	1	0	3	0	1
**Dazl**	3.0339	-0.6548	4.5012	0.0339	0.7959	0	0	0	0	0	0	0	0	0	5
**Abca13**	3.0321	-0.3328	4.3806	0.0364	0.811	0	0	0	0	0	0	0	0	0	5
**Hes7**	3.0318	-0.5741	4.8575	0.0275	0.7469	0	0	0	0	0	0	0	2	0	3
**Casr**	3.0293	1.0036	6.8453	0.0089	0.5138	0	0	0	0	0	0	3	0	0	2
**Gm39861**	3.0291	0.0154	4.6905	0.0303	0.7699	0	0	0	0	0	0	0	2	0	3
**Nr5a2**	3.0256	0.822	5.8622	0.0155	0.6198	0	0	0	0	0	0	1	0	0	4
**Gpr135**	3.0221	-0.0139	4.922	0.0265	0.7377	0	0	0	0	0	0	0	5	0	0
**Aldoart2**	3.0211	-0.4401	5.4175	0.0199	0.6772	0	0	0	0	0	3	2	0	0	0
**Amn**	3.0134	-0.6606	4.1035	0.0428	0.845	0	0	0	0	0	0	6	0	0	0
**Nphs2**	3.0122	-0.455	4.0756	0.0435	0.85	0	0	0	0	0	0	6	0	0	0
**1700003M**	3.0111	-0.5447	4.3364	0.0373	0.8131	0	0	0	0	0	0	0	5	0	0
**Gps1**	3.0021	-0.5466	3.842	0.05	0.8743	0	0	0	0	0	0	6	0	0	0
**LOC108167**	2.995	-0.438	4.4024	0.0359	0.811	1	0	0	0	0	3	0	4	1	4
**Smpd5**	2.9846	0.9348	10.117	0.0015	0.2469	0	2	0	0	0	2	14	3	0	2
**Gm32720**	2.9702	0.7594	13.795	0.0002	0.0895	0	0	3	0	0	4	13	3	4	2
**Gm29948**	2.9591	0.3145	8.2752	0.004	0.3802	2	0	1	0	0	0	3	17	3	3
**Gm41640**	2.9477	0.1749	9.7432	0.0018	0.2632	0	2	1	1	1	11	13	4	7	3
**Gh**	2.9458	6.9024	5.2285	0.0222	0.6995	0	0	0	0	0	0	0	0	2	2
**Gm40710**	2.9457	-0.4417	4.5614	0.0327	0.7852	0	0	0	0	0	3	0	0	1	0
**Col2a1**	2.9424	0.5753	5.9494	0.0147	0.6067	0	0	0	0	0	0	5	0	0	0
**Gm29901**	2.9422	-0.4659	4.0633	0.0438	0.8522	0	0	0	0	0	0	2	0	0	3
**LOC10524€**	2.9377	-0.6581	4.1333	0.042	0.8426	0	0	0	0	0	2	3	0	0	0
**Gpx2**	2.9341	1.1724	8.2718	0.004	0.3802	1	0	0	1	0	1	0	3	8	3
**Gm35531**	2.9264	-0.5079	4.4789	0.0343	0.7996	0	0	0	0	0	2	0	1	1	0
**Gm35380**	2.9165	-0.6242	3.9407	0.0471	0.8678	0	0	0	0	0	0	0	0	1	3
**Camkv**	2.9006	0.2243	5.4196	0.0199	0.6771	0	0	0	0	0	0	0	2	2	0
**Gm30149**	2.8911	-0.2371	6.0563	0.0139	0.6019	1	0	0	0	0	2	6	3	1	0
**Gm33533**	2.878	-0.4479	7.9615	0.0048	0.4019	1	1	0	0	0	2	12	3	0	3
**C5ar1**	2.8538	-0.4837	4.6518	0.031	0.7726	0	0	0	0	0	0	5	0	0	0
**Saxo1**	2.8424	-0.6724	5.9627	0.0146	0.6057	1	0	0	0	0	1	2	3	1	4
**Ankub1**	2.8326	-0.2939	4.5851	0.0323	0.7798	0	0	0	0	0	4	0	0	0	0

**Npy5r**	2.8293	-0.5578	4.7916	0.0286	0.7541	0	0	0	0	0	4	0	0	0	0
**Tmem181c**	2.8239	0.5709	5.3979	0.0202	0.6801	0	0	0	0	0	0	0	3	1	0
**Htr1f**	2.8147	1.5094	5.3213	0.0211	0.6946	0	0	0	0	0	4	0	0	0	0
**0610040J0**	2.7991	3.5586	9.2406	0.0024	0.3131	1	0	0	0	0	0	11	0	0	0
**Kcnd2**	2.7925	1.4764	5.6784	0.0172	0.6364	0	0	0	0	0	3	0	0	0	1
**Gm11627**	2.791	-0.267	4.1106	0.0426	0.8447	0	0	0	0	0	2	0	2	0	0
**Dcc**	2.7896	0.9658	5.7806	0.0162	0.6285	0	0	0	0	0	4	0	0	0	0
**1700039M**	2.7834	-0.3135	5.0484	0.0246	0.7214	2	0	0	0	0	1	3	6	5	0
**Crmp1**	2.7817	3.9165	5.8005	0.016	0.6285	0	0	0	0	0	0	0	0	0	4
**Gm9899**	2.7788	0.5443	5.5821	0.0181	0.6489	0	0	0	0	0	0	0	3	0	1
**Arap3**	2.7774	1.3462	5.4676	0.0194	0.6697	0	0	0	0	0	0	0	3	0	1
**Epha8**	2.7719	-0.293	4.248	0.0393	0.8224	0	0	0	0	0	0	0	0	3	0
**Gm11266**	2.7699	0.2395	5.0117	0.0252	0.7246	0	0	0	0	0	0	0	3	0	1
**C030016D1**	2.7639	-0.4329	4.4778	0.0343	0.7996	0	0	1	0	0	0	0	3	2	4
**LOC105243**	2.7601	-0.3015	4.6747	0.0306	0.7712	0	0	0	0	0	0	0	4	0	0
**Gm32258**	2.7575	-0.0261	4.5612	0.0327	0.7852	0	0	0	0	0	0	0	4	0	0
**Gm26744**	2.7538	0.1491	4.5553	0.0328	0.7852	2	0	0	0	0	0	11	9	0	0
**Gm40200**	2.7518	-0.7386	3.8673	0.0492	0.8715	0	1	0	0	0	0	0	3	3	2
**Ctla2b**	2.7516	-0.2165	4.3055	0.038	0.8131	0	0	0	0	0	0	0	4	0	0
**LOC108167**	2.7429	-0.8708	4.8518	0.0276	0.7469	0	0	0	0	0	0	1	1	0	2
**Lilr4b**	2.7425	-0.2862	3.9127	0.0479	0.8678	0	0	0	0	0	0	0	4	0	0
**Myl1**	2.7416	0.0464	3.871	0.0491	0.8715	0	0	0	0	0	0	0	4	0	0
**Serpinb1b**	2.738	1.0219	5.5021	0.019	0.6647	0	0	0	0	0	0	4	0	0	0
**6720489N1**	2.7343	1.4687	10.876	0.001	0.1957	0	0	2	1	0	2	19	4	0	0
**Tbc1d9**	2.7282	5.6428	9.11	0.0025	0.3165	0	0	0	0	1	0	6	2	0	2
**Zbtb32**	2.7241	0.8577	8.3498	0.0039	0.3742	0	0	0	0	2	3	14	0	1	1
**Gm32317**	2.7078	-0.1282	6.0525	0.0139	0.6019	0	0	0	1	0	5	0	1	2	1
**Gm39334**	2.6826	-0.2957	4.2814	0.0385	0.8178	0	0	0	0	0	0	2	2	0	0
**Slc4a1**	2.6742	0.2104	6.912	0.0086	0.5048	0	2	0	0	0	0	9	3	3	0
**LOC108167**	2.6682	-0.7631	5.3408	0.0208	0.6907	0	0	1	0	0	3	5	1	0	1
**Mast1**	2.6669	5.2688	16.412	5E-05	0.0459	2	0	1	0	0	8	0	7	5	1
**Gm32819**	2.6647	0.1614	5.6812	0.0171	0.6364	0	1	2	0	0	1	0	7	5	4
**Lbp**	2.6584	2.2698	15.095	0.0001	0.0634	4	0	0	0	0	3	16	5	4	0
**Gm40731**	2.6532	-0.5657	4.0283	0.0447	0.8574	0	0	0	0	0	0	0	0	2	1
**Smtnl1**	2.6495	0.1996	4.9945	0.0254	0.7278	1	0	0	0	0	2	9	0	0	0
**LOC108168**	2.6465	0.8322	6.4878	0.0109	0.5486	0	1	0	0	0	0	7	0	0	3
**Nek11**	2.6349	0.1113	4.428	0.0354	0.8086	0	0	0	0	0	0	4	0	0	0
**Myh7b**	2.6335	0.3959	5.8741	0.0154	0.6181	0	0	0	0	1	1	9	1	0	0
**Gm6878**	2.6324	-0.1281	3.9299	0.0474	0.8678	0	0	0	0	0	0	3	0	0	1
**9330102E0**	2.6062	3.1343	21.864	3E-06	0.0162	0	3	3	0	0	4	19	5	1	9
**Gm6306**	2.6052	-0.484	4.0713	0.0436	0.85	0	0	0	0	0	0	4	0	0	0
**Gm30855**	2.6048	0.2915	4.133	0.0421	0.8426	0	0	0	0	0	0	0	1	2	0
**Sv2b**	2.5977	6.6576	7.9918	0.0047	0.4019	1	0	0	0	0	8	0	0	0	1
**Gm32618**	2.5955	0.0385	3.9497	0.0469	0.8678	0	0	0	0	0	0	4	0	0	0
**Insc**	2.5856	2.9433	7.5168	0.0061	0.4459	0	0	0	0	1	0	0	4	0	5
**Gm29483**	2.5767	1.2797	8.848	0.0029	0.3309	0	3	0	0	0	9	0	6	2	1
**Nanos1**	2.5682	1.3964	6.0271	0.0141	0.604	0	0	0	1	0	0	0	8	0	1
**Lsmem2**	2.5626	0.3225	3.9421	0.0471	0.8678	0	0	0	0	0	2	0	0	1	0
**Gm16287**	2.5532	0.5855	5.8622	0.0155	0.6198	0	0	0	0	2	1	4	9	0	3
**Tmsb15b2**	2.5276	-0.0111	4.4376	0.0352	0.8066	0	0	1	0	0	0	10	0	0	0
**Plin1**	2.5264	-0.4367	3.9171	0.0478	0.8678	0	0	0	0	0	0	0	0	1	2
**Hgf**	2.5102	1.1026	4.4063	0.0358	0.811	0	0	0	0	0	0	0	0	3	0
**Kcnj9**	2.4755	1.6092	4.4109	0.0357	0.811	0	0	0	0	0	0	0	0	3	0
**Fgd1**	2.4753	0.5788	8.6264	0.0033	0.3485	0	1	0	0	2	1	8	4	4	2
**4930412L0**	2.47	0.2944	4.1826	0.0408	0.8344	0	0	0	0	1	0	6	0	0	4
**Gpc5**	2.4584	1.4454	4.3079	0.0379	0.8131	0	0	0	0	0	0	0	0	1	2
**Gm31985**	2.4549	0.6646	4.1949	0.0405	0.8329	0	0	0	0	0	3	0	0	0	0
**LOC108168**	2.4537	1.4653	4.3177	0.0377	0.8131	0	0	0	0	0	0	0	2	1	0
**5430416O(**	2.4537	0.095	4.042	0.0444	0.8561	0	0	0	0	0	2	0	0	0	1
**Adam8**	2.4526	0.2651	4.0733	0.0436	0.85	0	0	0	0	0	2	0	0	0	1
**A330032B1**	2.447	0.0843	4.4311	0.0353	0.8076	4	0	0	0	1	0	12	20	1	2
**Plcxd3**	2.4443	4.3518	4.4024	0.0359	0.811	0	0	0	0	0	0	0	0	2	1

**Agt**	2.4427	2.7511	4.3368	0.0373	0.8131	0	0	0	0	0	3	0	0	0	0
**Hlx**	2.4426	0.5983	4.1157	0.0425	0.844	0	0	0	0	0	1	0	0	0	2
**Gm29543**	2.4425	1.5817	4.3389	0.0373	0.8131	0	0	0	0	0	3	0	0	0	0
**Lrrtm3**	2.4423	3.0744	4.3801	0.0364	0.811	0	0	0	0	0	0	0	0	1	2
**9530052E0**	2.4407	-0.744	4.6386	0.0313	0.7726	0	0	0	0	1	1	3	3	0	2
**Icam2**	2.4389	0.6624	4.0282	0.0447	0.8574	0	0	0	0	0	1	0	2	0	0
**C130050O1**	2.4383	-0.2014	4.1625	0.0413	0.8377	0	0	0	0	0	1	0	2	0	0
**Elmo1**	2.4368	3.9687	4.3923	0.0361	0.811	0	0	0	0	0	3	0	0	0	0
**LOC105244**	2.4353	1.3961	4.2844	0.0385	0.8177	0	0	0	0	0	0	0	0	0	3
**5330417C2**	2.4351	1.4995	4.1588	0.0414	0.8377	0	0	0	0	0	0	0	0	0	3
**Slain1**	2.4346	1.314	4.0742	0.0435	0.85	0	0	0	0	0	0	0	0	0	3
**6720432D0**	2.4342	0.323	4.0336	0.0446	0.8574	0	0	0	0	0	0	0	0	0	3
**P2rx2**	2.434	0.7088	4.3607	0.0368	0.8127	0	0	1	0	0	0	0	8	0	0
**Panx2**	2.4339	4.869	4.3651	0.0367	0.8126	0	0	0	0	0	0	0	3	0	0
**Extl1**	2.4332	1.6444	4.3184	0.0377	0.8131	0	0	0	0	0	0	0	3	0	0
**Tmem198**	2.4325	2.9818	6.2986	0.0121	0.5668	1	0	0	0	0	0	0	6	0	2
**A230009B1**	2.4324	-0.3275	3.8782	0.0489	0.8696	0	0	0	0	0	0	0	0	0	3
**Gm31683**	2.4308	1.2799	4.1783	0.0409	0.8351	0	0	0	0	0	0	0	3	0	0
**Olfr1033**	2.429	0.2861	4.0909	0.0431	0.849	0	0	0	0	0	0	0	3	0	0
**Stfa2**	2.421	-0.4683	4.2323	0.0397	0.8235	1	0	0	0	1	1	8	0	3	2
**Il10ra**	2.3982	0.7062	4.7632	0.0291	0.7563	1	0	0	0	0	0	6	0	2	0
**Ticam2**	2.3918	-0.1092	4.4429	0.035	0.8062	0	0	0	1	1	3	8	1	2	0
**Srl**	2.369	1.6663	11.027	0.0009	0.1888	0	2	0	3	0	1	7	10	2	7
**Gm10432**	2.36	0.0429	3.8947	0.0484	0.8678	0	0	0	0	0	0	3	0	0	0
**Tmem52**	2.3483	-0.2015	4.0579	0.044	0.8533	1	1	4	0	0	4	0	10	8	1
**Mpped1**	2.3447	1.2713	8.8951	0.0029	0.3276	5	0	0	0	0	10	7	0	6	2
**Bglap3**	2.3445	-0.2445	5.1745	0.0229	0.7051	1	0	1	2	2	2	20	10	0	8
**Rgsl1**	2.3384	0.5229	4.143	0.0418	0.8403	0	0	0	0	1	0	1	0	3	3
**Ncf1**	2.3349	1.6756	5.3027	0.0213	0.6958	0	1	0	0	0	0	2	1	4	0
**LOC105242**	2.333	-0.1182	4.2861	0.0384	0.8177	0	1	0	0	0	0	0	4	1	2
**Cnrip1**	2.3024	4.4009	19.865	8E-06	0.0206	0	0	3	2	2	3	6	11	9	5
**4931440P2**	2.3	0.2527	8.5584	0.0034	0.3555	0	5	1	1	0	5	6	8	3	10
**Tvp23bos**	2.2985	1.8735	14.338	0.0002	0.0808	7	3	2	1	0	9	36	16	2	5
**Lin28b**	2.2809	1.5901	8.486	0.0036	0.3649	1	0	0	2	0	3	0	7	1	5
**Azin2**	2.2792	3.7552	10.341	0.0013	0.2316	1	0	1	0	1	2	0	3	1	10
**Tmem200c**	2.2687	1.2543	5.2143	0.0224	0.6995	0	1	0	0	0	0	0	2	0	5
**Hmha1**	2.2684	1.3735	9.6004	0.0019	0.2767	5	1	0	0	1	7	8	15	2	4
**Gm36478**	2.2524	0.7124	7.4894	0.0062	0.4459	0	1	3	0	0	0	7	7	4	1
**Gm32954**	2.2457	0.4658	4.9223	0.0265	0.7377	0	0	1	1	0	1	12	0	0	0
**Sdk1**	2.2422	3.414	5.328	0.021	0.6933	1	0	0	0	0	0	6	0	0	1
**Radil**	2.2288	1.9864	6.5809	0.0103	0.5435	0	0	0	1	1	0	10	0	1	1
**Tnfaip8l3**	2.2287	0.3744	4.5983	0.032	0.7798	1	1	0	0	0	1	6	0	0	5
**Pcdh10**	2.2031	4.6575	7.3984	0.0065	0.4569	1	0	1	0	0	0	8	0	1	2
**Zswim5**	2.2018	1.9253	5.9357	0.0148	0.6085	0	1	2	0	0	14	0	0	0	0
**Slc6a19**	2.1957	1.4252	4.6982	0.0302	0.7699	0	0	1	0	0	0	7	0	0	0
**Tmem132e**	2.1924	5.5489	9.5841	0.002	0.2774	1	0	2	0	0	0	6	0	5	4
**Siglecg**	2.1769	0.9546	14.949	0.0001	0.0662	2	3	9	2	2	7	27	16	9	18
**4921504A2**	2.15	1.6831	5.5913	0.018	0.6487	1	0	0	0	1	2	7	0	2	0
**Ednra**	2.1399	1.8111	5.9578	0.0147	0.6066	0	2	0	0	0	0	1	6	3	0
**LOC108167**	2.1221	1.4052	7.4486	0.0063	0.4505	3	1	0	0	0	6	8	3	1	1
**Dbp**	2.1107	0.7819	4.5228	0.0334	0.7916	0	13	2	2	0	5	37	13	7	2
**Gm41883**	2.1056	0.5963	4.0542	0.0441	0.8533	0	3	0	0	0	6	0	0	0	7
**BC048546**	2.0919	7.2328	10.452	0.0012	0.2316	1	0	3	0	0	0	9	6	1	2
**Ngb**	2.0895	1.1879	4.962	0.0259	0.7335	0	0	0	2	0	2	8	0	0	1
**Tubb3**	2.085	9.577	6.4349	0.0112	0.5522	0	0	0	0	2	0	0	0	3	7
**Ubxn11**	2.0702	0.4683	5.6209	0.0177	0.643	3	0	2	0	0	11	10	2	0	0
**Mirg**	2.0668	3.0401	4.1371	0.042	0.8418	0	1	0	0	0	0	0	3	0	3
**Gm42372**	2.0619	3.6379	4.2562	0.0391	0.8215	0	0	1	0	0	0	0	6	0	0
**Sult4a1**	2.0612	8.2555	4.3595	0.0368	0.8128	0	0	0	0	1	0	0	0	1	5
**C1ql4**	2.0554	2.0972	4.111	0.0426	0.8447	1	0	0	0	0	0	0	4	0	2
**Dok2**	2.0418	0.2338	4.1314	0.0421	0.8427	0	2	0	0	0	1	2	1	2	3
**Lrrc73**	2.0398	3.2196	12.696	0.0004	0.1232	0	5	2	0	0	10	4	13	1	0

**Myo1a**	2.0353	5.4404	12.998	0.0003	0.1116	0	1	3	0	2	0	7	9	0	9
**Gm20684**	2.0334	-0.0791	4.1977	0.0405	0.8329	1	0	0	2	0	0	9	0	1	5
**1700016C1**	2.0124	0.0268	4.8643	0.0274	0.7467	3	8	0	0	0	9	3	13	5	7
**Pcdhb3**	1.9768	1.1195	6.1911	0.0128	0.5841	2	0	1	0	0	4	2	6	0	1
**Kcne1**	1.9713	-0.1153	4.0095	0.0452	0.8577	3	0	2	1	0	4	9	0	0	13
**Samd11**	1.9662	0.4124	5.8386	0.0157	0.6238	0	3	3	1	0	7	7	4	5	1
**Gm16432**	1.9503	1.5625	6.4086	0.0114	0.5544	4	2	3	3	0	7	26	1	7	4
**Ggt7**	1.9474	3.3042	16.73	4E-05	0.0442	3	5	2	3	0	23	19	0	0	7
**Vstm4**	1.9363	2.4807	9.7842	0.0018	0.2615	1	4	0	0	1	3	13	8	0	0
**Gm33357**	1.9355	0.1875	5.5405	0.0186	0.6585	0	0	11	3	0	6	23	6	3	13
**AI593442**	1.932	5.8942	5.2652	0.0218	0.697	0	0	0	2	0	0	7	0	1	1
**Rin3**	1.9268	0.7565	5.177	0.0229	0.7051	0	1	0	3	0	3	1	3	3	5
**L3mbtl1**	1.9249	1.029	7.7597	0.0053	0.4269	2	2	3	0	0	3	9	9	3	2
**Cyp1b1**	1.9213	2.8436	11.081	0.0009	0.1879	2	3	1	0	3	18	5	0	3	7
**LOC10524!**	1.9196	-0.0789	4.9556	0.026	0.7335	0	2	0	2	0	1	0	8	2	4
**Fbxo44**	1.9034	4.9918	17.2	3E-05	0.0415	1	4	1	5	0	0	25	11	1	4
**LOC10816!**	1.8482	0.6215	3.9861	0.0459	0.8611	0	1	2	0	0	5	7	0	0	0
**Kirrel3**	1.8347	2.8196	8.3208	0.0039	0.375	0	0	3	1	1	8	0	2	1	7
**Gm2011**	1.8116	-0.2268	3.9038	0.0482	0.8678	0	0	0	3	1	0	7	2	2	5
**Amigo3**	1.8109	4.009	15.57	8E-05	0.055	2	5	2	3	3	3	32	6	5	7
**Enpp6**	1.8105	2.1922	3.9144	0.0479	0.8678	0	2	0	0	0	2	0	0	0	6
**Gm2573**	1.7938	1.4209	5.2781	0.0216	0.6958	1	3	7	2	2	2	4	10	7	23
**Vwa5b2**	1.7923	3.4223	6.0259	0.0141	0.604	0	4	0	0	0	3	2	7	2	0
**Gls2**	1.7858	1.4063	3.8955	0.0484	0.8678	2	0	0	0	0	4	0	0	0	4
**Pcdhb19**	1.7415	0.9971	6.9947	0.0082	0.4992	0	6	2	1	0	1	8	6	8	3
**Gal3st3**	1.7254	1.8814	4.9285	0.0264	0.7377	0	3	1	0	2	8	0	12	0	0
**Rinl**	1.7232	0.9633	5.4106	0.02	0.6772	0	3	3	0	2	6	0	8	6	3
**Adamts6**	1.7084	0.4167	3.9344	0.0473	0.8678	0	1	1	0	1	0	3	8	0	0
**Armc2**	1.7071	0.8545	4.9146	0.0266	0.7377	1	5	0	0	0	2	3	0	3	10
**Gpatch3**	1.6896	1.1031	8.7521	0.0031	0.3382	2	1	5	3	3	6	20	5	4	11
**Zfp811**	1.6856	2.0356	9.9093	0.0016	0.2583	2	3	6	4	3	1	33	10	6	9
**Fbxo16**	1.6778	1.6975	4.8567	0.0275	0.7469	2	0	1	1	0	0	12	0	2	0
**Obsl1**	1.6752	2.3872	8.1575	0.0043	0.3886	2	1	3	2	1	9	4	6	5	3
**Slc7a4**	1.6538	3.2659	21.16	4E-06	0.0162	5	9	5	12	0	17	20	18	6	30
**Nrn1l**	1.6305	2.8049	8.3132	0.0039	0.3756	1	4	0	1	2	3	11	0	0	11
**Scg2**	1.6294	6.412	13.963	0.0002	0.0871	1	4	2	5	1	19	7	2	6	4
**Gm6637**	1.6269	-0.1334	4.117	0.0425	0.8438	1	2	3	2	1	8	3	8	1	7
**Gm34939**	1.6162	0.8566	5.3883	0.0203	0.6822	4	4	5	4	3	7	39	18	0	5
**Fcrls**	1.6063	1.6387	4.0584	0.044	0.8533	0	0	4	0	0	0	0	10	2	0
**Trim46**	1.6053	3.0118	5.5025	0.019	0.6647	4	0	1	0	0	1	0	2	4	8
**Dcaf12l2**	1.6047	0.4182	5.1626	0.0231	0.707	8	3	1	9	0	21	20	6	8	5
**Zfp454**	1.5944	1.1737	3.8619	0.0494	0.8719	1	3	0	0	2	4	3	1	7	1
**Gm28609**	1.5798	0.7643	4.0661	0.0438	0.8517	3	12	0	4	7	13	15	34	11	2
**Gm40695**	1.579	1.7507	6.0562	0.0139	0.6019	0	2	5	1	0	4	12	7	0	1
**Gm30554**	1.5742	0.4718	4.4694	0.0345	0.8015	3	1	6	0	0	2	12	7	4	3
**Apoo**	1.5443	1.5823	6.7117	0.0096	0.525	3	1	0	6	2	8	24	1	2	3
**Pkd2l1**	1.5191	0.6566	4.2386	0.0395	0.823	0	6	1	2	1	10	6	6	3	1
**Fbln7**	1.4992	2.3575	5.2862	0.0215	0.6958	0	12	0	0	1	15	18	0	0	2
**Dcdc2b**	1.441	0.4531	4.3744	0.0365	0.8112	3	1	6	0	3	8	18	4	2	5
**Creg2**	1.4353	3.9036	7.0215	0.0081	0.499	0	0	11	6	0	1	7	3	21	3
**Tmem100**	1.4339	4.6173	5.3145	0.0211	0.6958	1	0	3	2	0	4	0	11	1	0
**Echdc2**	1.4226	2.2386	15.576	8E-05	0.055	6	24	7	9	7	14	52	28	10	32
**Eif5a**	1.3953	1.817	7.9752	0.0047	0.4019	5	24	7	0	7	11	62	11	8	19
**Kcna6**	1.3861	6.161	7.0204	0.0081	0.499	6	0	0	3	0	3	9	5	0	6
**A330069E1**	1.3682	2.0629	5.0433	0.0247	0.7217	2	1	7	4	0	8	8	9	2	7
**Tmem240**	1.3679	1.7154	4.845	0.0277	0.747	4	2	1	0	1	5	9	4	0	3
**C330013E1**	1.3625	1.3847	5.835	0.0157	0.6238	5	11	4	11	2	24	29	3	9	13
**Lurap1**	1.3542	2.2509	4.0746	0.0435	0.85	1	6	0	0	0	3	0	11	0	3
**Zfp783**	1.3295	1.2313	4.8293	0.028	0.7488	1	4	0	4	4	5	5	18	2	3
**Fhod3**	1.3249	3.3177	10.349	0.0013	0.2316	5	2	9	3	2	10	22	11	2	6
**Acacb**	1.308	2.3173	8.0434	0.0046	0.3978	7	3	0	5	1	7	8	7	3	13
**D630045J1**	1.3068	3.4204	7.196	0.0073	0.4797	6	0	3	1	3	3	15	8	2	4

**Ndst3**	1.2961	3.231	8.3342	0.0039	0.3742	0	10	3	0	2	5	11	7	0	12
**Serp2**	1.2809	5.0201	6.5313	0.0106	0.546	0	2	9	0	0	15	0	6	2	2
**Nr1h3**	1.2764	2.4537	6.4366	0.0112	0.5522	0	4	12	0	0	7	15	9	2	3
**Plppr3**	1.2626	3.1535	4.3727	0.0365	0.8115	2	0	3	0	3	0	11	0	5	3
**Zfp759**	1.2594	1.9483	9.7112	0.0018	0.2651	7	11	20	11	10	24	25	31	22	24
**Rtn2**	1.2473	5.3868	6.009	0.0142	0.604	7	0	0	3	0	2	9	6	0	6
**Lancl3**	1.2291	3.2078	6.5498	0.0105	0.5454	1	3	8	1	1	7	12	8	3	1
**Camk1**	1.2252	2.4995	8.7719	0.0031	0.3382	3	6	11	4	7	11	35	11	10	3
**Fgf14**	1.2241	2.7455	7.2074	0.0073	0.4784	6	4	13	13	7	5	42	33	9	13
**Mirc35hg**	1.2165	2.7892	4.6494	0.0311	0.7726	2	5	6	3	0	15	6	2	3	7
**Slc24a3**	1.2124	5.5637	13.209	0.0003	0.1101	12	14	10	10	2	24	58	11	1	16
**Dclk2**	1.2102	5.3325	4.5034	0.0338	0.7954	2	5	0	0	1	4	8	5	1	0
**Rbm12b1**	1.2091	1.4867	5.1335	0.0235	0.7095	4	14	2	4	3	17	15	19	2	6
**Iglon5**	1.2036	4.8875	7.2894	0.0069	0.4711	3	2	6	4	4	14	14	13	0	2
**Tomm5**	1.2002	2.3294	7.0968	0.0077	0.4918	1	3	17	9	4	11	18	11	6	27
**Rhof**	1.1912	3.524	6.7142	0.0096	0.525	0	2	11	5	3	12	27	3	0	6
**Kcnk13**	1.1875	1.529	8.0987	0.0044	0.3937	4	3	7	5	1	7	12	7	3	14
**Fam69b**	1.1621	5.4321	13.135	0.0003	0.1116	5	0	15	8	6	11	28	22	3	9
**Filip1**	1.1504	4.8424	4.6581	0.0309	0.7726	5	3	1	1	0	0	21	0	1	0
**Fcgr3**	1.1405	2.3061	5.697	0.017	0.6337	8	10	5	2	2	14	23	6	3	11
**Gria2**	1.1389	4.818	9.9401	0.0016	0.2574	5	12	2	7	0	13	8	23	3	6
**Dalrd3**	1.1376	1.6853	5.2419	0.022	0.6995	2	10	11	4	4	3	27	11	9	13
**Tspyl3**	1.1342	2.4191	4.4538	0.0348	0.8037	7	2	0	4	1	4	0	7	7	10
**Spink10**	1.1339	2.599	8.7176	0.0032	0.3385	9	11	30	6	10	23	51	22	21	15
**Abcb1a**	1.1257	4.1697	4.3132	0.0378	0.8131	4	4	0	2	0	6	5	7	0	3
**S1pr2**	1.1147	2.3042	5.8277	0.0158	0.6251	7	13	24	1	6	11	51	26	3	18
**Wdr54**	1.109	2.9125	5.1979	0.0226	0.7032	1	5	5	0	3	9	13	2	1	4
**Usp27x**	1.1061	0.9056	4.764	0.0291	0.7563	4	3	12	5	0	10	13	5	4	15
**Lix1l**	1.1016	2.2033	5.1377	0.0234	0.7084	5	6	10	2	5	5	32	3	3	18
**Fam20c**	1.0803	4.0657	8.6742	0.0032	0.3425	3	4	16	2	4	7	16	14	11	8
**Rab6b**	1.0795	8.8355	8.9566	0.0028	0.324	7	7	5	1	5	16	0	18	4	11
**Gpr155**	1.078	4.1895	8.0819	0.0045	0.3937	8	4	8	3	1	9	15	14	1	9
**Gbp3**	1.075	2.8373	7.4039	0.0065	0.4564	21	25	16	18	3	22	32	49	25	22
**Fam43b**	1.0738	4.568	6.1908	0.0128	0.5841	0	1	5	5	6	4	20	4	1	6
**Ltf**	1.0735	2.1452	4.7977	0.0285	0.7527	1	44	29	17	12	25	44	32	21	66
**Armcx3**	1.0728	3.9705	5.0841	0.0241	0.7184	5	0	7	0	3	8	0	15	0	7
**Gm5113**	1.0545	2.0446	5.1576	0.0231	0.7074	4	5	3	13	4	12	14	20	4	9
**Immp2l**	1.0542	2.0445	4.2483	0.0393	0.8224	1	6	5	4	1	6	13	4	0	11
**Rnls**	1.0521	0.9901	4.0864	0.0432	0.85	5	11	6	6	2	16	17	5	10	6
**Eaf2**	1.0368	0.9604	5.4672	0.0194	0.6697	9	6	12	6	5	6	24	17	11	15
**Zfp939**	1.0366	2.3597	4.8543	0.0276	0.7469	26	28	6	5	5	24	42	36	4	34
**Ccdc138**	1.0342	1.4366	4.3022	0.0381	0.8131	3	11	11	7	2	10	8	33	5	6
**Hexim2**	1.0222	2.4987	6.5953	0.0102	0.5434	5	22	16	11	2	30	41	14	7	11
**Cfap100**	1.0212	3.8222	4.6248	0.0315	0.7738	5	5	2	2	1	5	13	2	3	6
**Cntnap4**	1.0184	3.1372	3.9742	0.0462	0.8611	2	4	5	3	3	1	16	0	2	15
**Prx**	1.0168	7.3955	5.4512	0.0196	0.6713	0	5	1	3	7	2	11	7	1	10
**Afap1l1**	1.012	2.695	6.4674	0.011	0.5486	12	14	16	2	7	7	19	22	4	46
**Rnf122**	1.0088	3.5247	5.6486	0.0175	0.6381	4	5	13	7	2	3	12	30	2	12
**Zfp874a**	1.0075	2.7884	9.5599	0.002	0.2785	10	17	22	9	7	6	26	44	14	29
**Trib3**	1.0022	1.7691	7.9628	0.0048	0.4019	15	12	11	13	10	14	43	20	19	20
**Kcnc3**	0.9888	6.7981	9.4519	0.0021	0.2865	16	5	8	2	2	3	35	10	8	6
**Brdt**	0.9854	3.3223	5.2394	0.0221	0.6995	14	3	5	6	1	5	19	13	12	4
**H2-DMb1**	0.9806	3.8725	11.999	0.0005	0.151	26	21	41	11	9	50	41	58	19	24
**Cercam**	0.9707	2.4822	5.0334	0.0249	0.7219	2	20	12	7	2	18	35	7	3	15
**Tmem45b**	0.9696	3.2328	5.532	0.0187	0.6586	4	3	5	3	8	13	5	12	3	10
**Mylk3**	0.9504	1.2243	3.9951	0.0456	0.8597	11	13	12	7	9	33	28	14	10	10
**Hacl1**	0.9502	2.3345	8.3279	0.0039	0.3745	24	13	22	19	7	31	48	21	15	40
**Napb**	0.9497	5.3558	3.9355	0.0473	0.8678	2	0	7	3	1	2	16	0	1	5
**Sgcd**	0.9234	5.0296	7.3769	0.0066	0.4606	5	17	1	5	5	3	28	21	1	7
**Zfp868**	0.9224	2.6012	8.0797	0.0045	0.3937	19	19	22	15	12	20	17	50	24	36
**Fbxo2**	0.909	8.0864	5.7682	0.0163	0.6285	3	7	0	11	2	11	5	8	8	8
**Tor2a**	0.9067	1.8247	6.6206	0.0101	0.5413	11	22	22	18	7	15	45	23	20	32

**Ahr**	0.9038	3.0744	7.6081	0.0058	0.4373	46	26	35	19	19	56	27	51	33	78
**Arhgap31**	0.8911	4.4979	5.3001	0.0213	0.6958	25	5	8	2	13	12	25	25	15	20
**Arxes2**	0.8872	3.9578	5.237	0.0221	0.6995	2	15	20	2	1	24	7	5	11	14
**Zfp93**	0.8836	2.495	7.6704	0.0056	0.434	19	23	28	10	10	21	55	25	21	30
**Gapdh**	0.8821	4.8692	7.1263	0.0076	0.4871	39	53	43	23	38	30	84	55	61	99
**Tmem25**	0.8752	5.3243	6.5981	0.0102	0.5433	7	10	12	1	11	0	24	33	5	11
**Sipa1**	0.875	3.1564	7.634	0.0057	0.4356	12	17	12	9	2	23	22	20	4	19
**Dand5**	0.8748	2.7933	4.4404	0.0351	0.8064	14	10	11	17	9	12	5	43	19	21
**Pear1**	0.8742	2.3012	6.5148	0.0107	0.546	16	15	6	6	17	24	19	25	13	25
**Ttc21b**	0.87	2.5131	5.42	0.0199	0.6771	11	10	16	5	3	8	16	10	20	16
**Akr1b8**	0.8663	2.2981	4.9496	0.0261	0.7335	10	24	27	13	6	43	13	31	12	26
**Shisa2**	0.8595	2.2381	6.5758	0.0103	0.5435	20	25	20	14	12	26	24	45	15	42
**Naalad2**	0.8524	2.4546	4.4853	0.0342	0.7986	13	18	31	10	16	26	58	29	22	14
**Creb5**	0.8502	3.8531	5.1063	0.0238	0.7155	23	20	8	9	0	23	12	42	7	13
**Catsper2**	0.8484	1.9078	4.2204	0.0399	0.8263	10	11	19	10	6	9	35	23	7	23
**Fads3**	0.8479	3.6058	8.5812	0.0034	0.3532	11	15	19	13	2	24	15	37	7	15
**Armcx1**	0.8478	5.0558	5.5409	0.0186	0.6585	3	4	20	0	1	8	17	10	0	12
**Col5a3**	0.843	5.3368	4.6947	0.0303	0.7699	9	5	7	0	2	7	18	7	2	5
**B9d1**	0.8395	3.25	5.9438	0.0148	0.6079	15	39	55	25	25	70	54	33	27	74
**Timeless**	0.8351	3.6541	11.055	0.0009	0.1883	24	53	32	39	10	55	92	59	15	43
**5930412G1**	0.8327	1.9612	3.9341	0.0473	0.8678	9	31	5	12	5	19	28	25	12	14
**Galns**	0.8296	2.1894	4.5178	0.0335	0.7919	32	12	21	14	12	42	64	35	10	13
**Lrrk2**	0.817	4.1271	3.9766	0.0461	0.8611	5	2	14	6	4	16	5	12	8	8
**1810043G0**	0.8161	4.2241	10.339	0.0013	0.2316	23	37	25	33	7	49	74	44	10	31
**Pex12**	0.8137	2.8973	6.9976	0.0082	0.4992	23	58	41	26	18	51	35	66	26	79
**Mypop**	0.8111	4.1724	4.0554	0.044	0.8533	6	10	2	9	0	10	14	11	4	5
**Gprasp2**	0.8026	5.8317	8.1322	0.0043	0.3911	9	10	15	9	10	21	33	19	9	5
**Tgif2**	0.7932	2.9249	9.8395	0.0017	0.261	27	38	47	29	14	51	68	63	18	49
**Oxld1**	0.7905	2.3119	4.0018	0.0455	0.8587	16	5	16	11	11	28	14	24	7	26
**Dpp4**	0.7781	2.8949	8.4309	0.0037	0.3687	38	63	46	28	10	46	61	40	37	89
**Robo1**	0.7689	3.0133	4.0217	0.0449	0.8577	7	24	17	6	1	17	9	33	7	14
**Dlg4**	0.7688	5.8854	7.4067	0.0065	0.4564	17	6	23	8	8	12	55	15	6	15
**Fermt3**	0.7686	2.7068	4.5917	0.0321	0.7798	33	56	47	54	18	34	56	52	46	120
**Prrt1**	0.7679	3.0869	3.9757	0.0462	0.8611	6	3	19	5	3	3	21	20	4	10
**Plekho2**	0.7563	2.8044	4.4836	0.0342	0.7989	11	16	22	11	2	25	10	25	7	25
**Nrbf2**	0.7557	2.884	4.3046	0.038	0.8131	18	23	17	23	4	19	23	18	24	38
**Fam149a**	0.7542	3.5194	3.9745	0.0462	0.8611	4	8	9	3	5	12	14	12	2	6
**Tmem29**	0.7501	3.3735	8.2467	0.0041	0.3832	36	40	38	27	16	35	42	65	31	64
**Uxt**	0.7472	2.726	5.2331	0.0222	0.6995	30	41	15	22	6	44	31	22	24	44
**4930430F0**	0.7388	3.7772	8.2197	0.0041	0.3832	15	35	25	36	15	29	41	55	16	55
**Particl**	0.7358	2.5513	4.1004	0.0429	0.8461	8	21	6	9	3	16	23	10	8	14
**Ap1g2**	0.7267	2.1332	3.878	0.0489	0.8696	24	38	18	16	19	30	71	25	21	36
**Ccdc28a**	0.7249	5.3755	11.807	0.0006	0.1573	19	40	29	32	19	45	92	50	9	26
**Ppm1m**	0.72	2.9343	4.0173	0.045	0.8577	12	20	19	8	3	8	37	39	5	7
**Sh3pxd2b**	0.7193	4.5583	11.342	0.0008	0.1795	34	48	21	31	9	49	64	48	19	37
**Snx21**	0.7156	3.5698	6.4639	0.011	0.5486	11	33	33	15	14	37	46	22	5	54
**Rtp4**	0.714	3.6326	4.8073	0.0283	0.7513	51	129	46	74	28	56	125	40	100	118
**Colgalt2**	0.709	4.0511	5.9306	0.0149	0.6085	12	25	22	10	12	42	23	24	14	16
**Trappc2**	0.7054	4.1983	4.3371	0.0373	0.8131	8	10	12	8	9	6	16	17	8	25
**Gusb**	0.7051	3.3502	5.7795	0.0162	0.6285	27	29	50	27	31	71	55	37	38	43
**Il1rap**	0.7022	2.4927	4.0063	0.0453	0.8577	9	16	16	7	3	12	26	14	7	17
**Adamts1**	0.6995	4.1029	5.4216	0.0199	0.6771	43	34	83	82	38	75	140	176	26	41
**Slc30a5**	0.6994	3.4628	6.7057	0.0096	0.525	27	30	43	40	20	35	61	54	20	77
**Abhd11**	0.6987	2.5541	4.4175	0.0356	0.811	30	28	20	22	9	26	71	20	12	43
**LOC108167**	0.697	3.6349	10.132	0.0015	0.2468	26	46	29	21	25	60	64	55	16	30
**B3galt5**	0.6966	3.7808	4.9641	0.0259	0.7335	7	15	12	11	11	24	9	11	13	25
**Pfkp**	0.6925	6.944	16.307	5E-05	0.0461	51	70	53	35	22	82	56	73	48	72
**Angptl7**	0.6876	5.5251	6.0366	0.014	0.604	132	514	618	266	124	255	659	952	172	337
**Gab2**	0.6819	4.5415	4.5445	0.033	0.7852	13	22	7	13	8	7	26	14	28	13
**H1fx**	0.6782	4.7366	5.0257	0.025	0.7219	19	7	29	17	9	10	36	24	28	18
**Atp6v0e2**	0.6775	7.4838	18.717	2E-05	0.0264	33	65	55	43	37	75	84	83	43	57
**Nin**	0.6759	3.815	6.5565	0.0105	0.5454	23	21	19	15	5	27	49	14	11	23

**Kyat3**	0.675	2.9849	4.5481	0.033	0.7852	39	45	44	54	17	33	62	93	26	82
**Cenpt**	0.6729	2.4231	4.1541	0.0415	0.8377	18	32	38	18	20	41	37	44	17	47
**Fbxl18**	0.6726	3.2992	4.3263	0.0375	0.8131	12	23	38	25	17	20	55	37	28	27
**Fbl**	0.6721	2.9162	4.9054	0.0268	0.7377	35	35	39	42	30	59	47	88	24	59
**Tmsb10**	0.668	6.0001	6.5534	0.0105	0.5454	62	81	54	47	81	85	89	84	100	115
**Rarres1**	0.6655	4.8116	8.0528	0.0045	0.3978	18	64	28	21	13	39	32	45	30	51
**Lmnb2**	0.6644	3.7546	11.542	0.0007	0.171	69	97	88	59	40	96	126	114	43	140
**Apbb1**	0.6613	6.9813	8.7257	0.0031	0.3385	20	23	35	10	5	36	32	30	11	25
**Ttc32**	0.6583	2.5992	3.9718	0.0463	0.8616	16	34	54	19	10	37	56	37	10	52
**Ptchd1**	0.657	2.4063	3.9966	0.0456	0.8597	4	17	26	12	15	25	14	36	13	18
**Ostm1**	0.6482	4.2969	9.2944	0.0023	0.3074	42	66	59	63	22	41	119	64	39	103
**Fxyd6**	0.6398	6.3113	6.4915	0.0108	0.5486	31	26	35	3	2	17	57	23	22	17
**Zfp358**	0.6392	3.5803	6.0423	0.014	0.604	37	42	42	42	23	59	104	57	29	27
**Gnaz**	0.6384	6.2114	4.8519	0.0276	0.7469	18	15	5	12	1	5	42	8	8	12
**Scarf2**	0.638	2.4647	4.7057	0.0301	0.7694	36	37	33	15	20	26	64	66	19	35
**Pop4**	0.6356	4.8316	4.7795	0.0288	0.7541	93	201	96	78	56	127	178	96	97	212
**Cebpd**	0.6311	3.0602	5.2721	0.0217	0.6958	19	29	30	27	12	21	47	35	15	51
**Matn2**	0.6247	5.1143	5.1039	0.0239	0.7157	8	19	23	9	5	20	39	11	9	12
**Mad2l2**	0.6237	2.9876	4.0264	0.0448	0.8574	40	15	24	15	15	19	55	30	16	45
**Wdr45**	0.6149	3.7959	5.3955	0.0202	0.6801	26	40	44	48	11	51	89	25	22	53
**Kctd7**	0.6102	3.5088	6.6527	0.0099	0.5356	33	16	32	18	13	24	52	40	21	23
**Ccpg1os**	0.6102	2.9199	4.1745	0.041	0.8353	25	20	49	51	11	46	36	59	26	46
**Zfyve1**	0.6075	3.6915	9.2018	0.0024	0.3137	26	52	46	32	24	56	95	43	23	40
**Fam189b**	0.6045	6.7863	10.206	0.0014	0.2422	33	36	41	20	22	33	52	55	15	61
**Col5a1**	0.6023	4.4851	4.1656	0.0413	0.8377	54	123	244	102	32	88	266	261	47	108
**Aldh1l1**	0.6012	2.811	4.8802	0.0272	0.7422	26	64	36	43	23	58	67	77	24	43
**Slc9a7**	0.5993	5.8178	8.5227	0.0035	0.3595	10	45	32	30	9	26	31	55	18	42
**Znhit2**	0.5967	4.7333	13.229	0.0003	0.1101	96	106	103	95	40	117	172	86	62	177
**Eid2**	0.5939	5.4073	6.6807	0.0097	0.531	9	29	15	34	18	46	30	31	18	21
**Telo2**	0.5938	3.0463	4.8872	0.0271	0.7414	16	28	46	33	23	30	73	52	10	52
**Gm36449**	0.5879	4.2688	5.1747	0.0229	0.7051	84	67	71	40	57	66	94	69	50	181
**Trappc6b**	0.5861	3.7594	5.8169	0.0159	0.6274	28	59	46	37	24	40	74	50	15	97
**Tmem14a**	0.5859	4.8352	7.7047	0.0055	0.4317	58	41	43	30	22	46	87	72	31	38
**Anks1**	0.5852	4.7715	11.239	0.0008	0.1801	80	104	124	85	47	127	160	140	66	110
**Ltbp3**	0.5845	3.2179	4.2651	0.0389	0.8206	20	40	25	34	31	24	74	53	27	41
**Ftl1**	0.584	6.272	7.6854	0.0056	0.434	148	157	159	144	138	217	286	164	147	249
**Unk**	0.5839	3.2737	3.8817	0.0488	0.8694	34	35	40	40	39	39	73	63	34	66
**Arhgap28**	0.5837	4.5321	5.0181	0.0251	0.7232	15	13	21	14	7	16	19	28	8	26
**Dnajc14**	0.5836	2.6796	4.8894	0.027	0.741	20	37	19	23	20	36	51	42	14	27
**Slc52a2**	0.5826	4.8164	5.0469	0.0247	0.7214	13	24	13	26	19	17	48	28	12	33
**Kank4**	0.5795	5.8703	6.2553	0.0124	0.5751	9	26	27	21	5	23	29	43	11	14
**Flad1**	0.5771	3.6933	5.3763	0.0204	0.6838	51	36	40	22	32	41	72	82	21	49
**Lrwd1**	0.5758	3.6587	6.7871	0.0092	0.5176	57	78	58	45	30	96	83	66	27	98
**Cdyl2**	0.5743	3.7812	4.7695	0.029	0.7557	15	23	21	16	7	20	31	37	5	21
**Ugcg**	0.5652	4.5224	4.556	0.0328	0.7852	15	27	25	15	14	12	47	13	23	35
**Fibp**	0.5635	3.6019	4.8197	0.0281	0.7493	56	62	30	55	14	34	100	70	19	85
**Bphl**	0.5604	3.7498	5.404	0.0201	0.6787	57	77	101	69	31	61	107	83	43	157
**Aldh16a1**	0.5593	3.4017	4.1267	0.0422	0.8429	47	77	84	52	22	63	116	52	35	111
**Xk**	0.5533	3.8947	7.0045	0.0081	0.4992	23	23	42	37	20	29	65	46	23	37
**Mkl1**	0.5505	3.6759	6.0044	0.0143	0.604	55	51	59	51	19	39	119	54	41	66
**Mboat2**	0.55	5.6042	6.0539	0.0139	0.6019	7	36	30	26	4	26	36	37	15	22
**Mfsd8**	0.5492	2.6854	3.9511	0.0468	0.8678	27	36	25	16	8	22	54	38	5	37
**Prelid1**	0.5487	4.2123	7.2836	0.007	0.4715	70	74	65	50	44	56	128	64	47	125
**Ttll7**	0.547	8.8792	6.9135	0.0086	0.5048	11	12	38	28	16	25	26	45	13	33
**Acox3**	0.5465	4.8201	10.886	0.001	0.1957	62	99	89	82	46	91	119	160	55	83
**Zfp937**	0.5453	3.5567	7.2045	0.0073	0.4784	30	61	85	44	19	57	101	64	39	51
**Smarca1**	0.5417	4.5037	4.3272	0.0375	0.8131	22	18	17	21	15	41	31	12	22	18
**Memo1**	0.5408	3.6704	4.6916	0.0303	0.7699	42	63	82	53	28	70	90	52	30	117
**Ubfd1**	0.5395	4.2711	6.809	0.0091	0.5171	46	64	62	48	39	69	63	66	46	98
**Lcmt1**	0.5378	4.964	9.5996	0.0019	0.2767	92	121	86	63	41	88	131	89	68	151
**Cyb561d2**	0.5375	4.1446	4.9763	0.0257	0.7331	35	41	41	32	44	53	76	46	33	63
**Bet1**	0.5371	3.308	4.5114	0.0337	0.794	34	27	56	35	22	25	57	60	24	71

**Heatr3**	0.5364	3.9849	5.1815	0.0228	0.7051	43	94	94	61	29	73	140	62	63	70
**Vps16**	0.5354	5.0715	13.464	0.0002	0.1029	89	173	133	97	83	135	215	181	86	154
**Sbk1**	0.5347	4.6056	4.2607	0.039	0.8215	17	31	34	37	6	20	64	32	11	43
**Ift43**	0.5336	3.909	7.5118	0.0061	0.4459	53	71	65	81	25	85	121	83	30	82
**Tdrp**	0.5322	3.7936	6.144	0.0132	0.5897	43	69	71	57	20	77	94	83	16	81
**Ahcyl2**	0.532	5.1188	5.6507	0.0174	0.6381	35	40	48	29	47	56	49	48	42	73
**Snhg18**	0.5272	4.6044	7.3496	0.0067	0.4632	58	68	90	63	22	77	146	84	29	71
**Srprb**	0.5269	4.3568	6.4317	0.0112	0.5523	62	75	88	55	31	67	128	76	44	98
**Bnip3**	0.5223	5.8122	5.4152	0.02	0.6772	33	40	21	21	15	61	32	22	23	30
**Car9**	0.521	2.7114	3.9192	0.0477	0.8678	30	88	50	52	22	77	106	58	29	46
**Rbm43**	0.5204	3.4444	4.1571	0.0415	0.8377	23	60	41	45	23	45	62	48	36	55
**Kcnk1**	0.5167	6.1925	4.2435	0.0394	0.823	30	18	46	38	25	34	30	71	32	39
**Fermt2**	0.5132	6.3222	4.8766	0.0272	0.7431	20	31	45	17	16	25	73	33	8	37
**Gm6988**	0.5106	3.0228	5.0273	0.025	0.7219	45	71	58	38	30	59	77	53	49	68
**Zfp592**	0.5103	4.3184	7.5343	0.0061	0.4459	54	90	116	94	40	93	159	107	43	122
**Uqcrfs1**	0.51	6.1937	9.8352	0.0017	0.261	204	233	201	245	143	231	342	205	187	388
**Lyrm1**	0.5095	3.7989	4.0408	0.0444	0.8563	33	23	45	21	25	39	86	43	9	33
**Hip1**	0.5073	5.2739	5.4643	0.0194	0.6697	22	23	31	25	18	38	60	23	6	35
**Nudcd1**	0.507	3.4022	4.6886	0.0304	0.7699	51	58	49	28	31	36	93	72	23	73
**Glcci1**	0.5051	4.3741	5.1381	0.0234	0.7084	27	38	37	14	17	43	42	33	7	51
**Cisd3**	0.5047	5.1466	4.039	0.0445	0.8567	49	65	48	35	25	68	125	47	17	49
**Mzt1**	0.5043	3.5523	4.679	0.0305	0.7712	41	32	31	34	26	44	49	44	26	56
**Zfyve19**	0.5011	4.0821	6.5366	0.0106	0.5456	45	40	71	55	20	57	92	62	31	61
**Vhl**	0.4967	4.177	4.6443	0.0312	0.7726	78	105	114	76	42	122	129	92	43	153
**Gabbr1**	0.4961	7.3531	4.6645	0.0308	0.772	44	62	47	20	19	27	128	61	11	38
**Tmem170b**	0.4956	3.2092	3.883	0.0488	0.8694	26	45	52	44	9	36	66	55	26	39
**Spata13**	0.4927	5.4925	11.099	0.0009	0.1872	106	244	195	180	68	181	306	220	73	252
**Dmap1**	0.491	4.1232	6.6789	0.0098	0.531	56	91	94	41	39	99	113	83	35	85
**Abcb10**	0.4896	4.3645	6.3964	0.0114	0.5574	79	79	90	77	43	85	139	110	53	96
**Psmg2**	0.4864	4.4333	6.5259	0.0106	0.546	67	63	82	75	52	88	125	70	54	111
**Gamt**	0.4851	4.7937	6.4516	0.0111	0.5501	28	46	57	35	24	49	71	36	23	67
**Kctd3**	0.4847	4.3904	4.5869	0.0322	0.7798	63	104	65	48	28	91	86	78	32	103
**Ptpmt1**	0.4842	5.0693	9.1938	0.0024	0.3137	94	161	120	132	60	125	222	121	87	172
**Ddx28**	0.4815	3.8113	4.14	0.0419	0.8413	34	87	76	29	22	51	110	42	46	53
**Ldha**	0.4806	5.3394	6.4748	0.0109	0.5486	184	296	196	128	100	195	253	185	149	331
**Wdr35**	0.479	4.191	5.8092	0.0159	0.628	31	78	62	49	34	61	58	103	34	65
**Mras**	0.4777	6.8429	5.7123	0.0168	0.6337	40	100	137	37	32	116	112	114	25	67
**Ulk1**	0.4764	5.3362	9.219	0.0024	0.3133	122	234	197	159	78	256	235	226	80	204
**Pdcl**	0.476	4.4701	6.5779	0.0103	0.5435	83	108	81	82	35	90	148	106	53	101
**Slc39a11**	0.4759	4.1156	4.511	0.0337	0.794	63	115	88	75	34	99	118	77	70	89
**Pus10**	0.4734	3.1709	3.9387	0.0472	0.8678	51	46	64	51	30	56	58	88	33	76
**Slx4ip**	0.473	3.8557	7.204	0.0073	0.4784	61	94	128	74	37	80	175	101	44	108
**Nes**	0.4702	5.0478	6.1966	0.0128	0.5841	42	55	45	38	33	54	98	77	18	37
**Serpinh1**	0.4608	5.1089	5.047	0.0247	0.7214	80	102	124	88	42	78	219	109	40	130
**Polr3c**	0.46	3.8363	5.0168	0.0251	0.7232	54	52	75	68	39	75	92	83	49	67
**Srp9**	0.4576	4.8754	7.0017	0.0081	0.4992	67	100	118	51	28	76	167	80	48	84
**Bud13**	0.4498	3.7093	5.8802	0.0153	0.6176	71	86	87	52	47	107	113	87	50	74
**Gabpb1**	0.4493	3.337	4.0222	0.0449	0.8577	54	64	62	40	17	57	83	45	34	72
**Zbtb17**	0.4487	4.4907	7.0459	0.0079	0.4954	119	180	144	99	54	158	236	153	84	105
**Slc7a8**	0.4428	4.4525	4.3835	0.0363	0.811	97	169	141	167	77	118	192	182	116	187
**Ptcd2**	0.4422	4.8077	8.0463	0.0046	0.3978	88	108	95	102	28	102	139	103	56	120
**Kat2a**	0.442	4.4751	6.3803	0.0115	0.5577	68	152	131	85	60	124	168	107	64	150
**Tmc4**	0.4402	3.0478	4.6173	0.0317	0.7767	66	76	64	49	38	62	90	76	47	90
**Fam73b**	0.4392	4.5216	4.3401	0.0372	0.8131	32	64	45	61	27	43	86	31	48	72
**Thbd**	0.4392	4.1384	6.5899	0.0103	0.5434	61	123	126	97	69	99	110	147	66	168
**Idnk**	0.4386	4.7812	6.726	0.0095	0.525	75	90	109	44	46	82	137	81	60	90
**Maged1**	0.4368	7.11	9.9838	0.0016	0.2559	94	177	171	169	76	162	273	154	68	218
**Ankrd34c**	0.4348	4.1549	4.3706	0.0366	0.8119	75	109	103	54	64	98	147	77	64	121
**Pkia**	0.4343	5.4833	5.039	0.0248	0.7219	40	30	35	29	6	37	56	24	20	37
**Slc25a46**	0.4329	6.2372	10.36	0.0013	0.2316	134	178	162	80	98	180	209	141	115	154
**Tigar**	0.4327	5.2577	5.3564	0.0206	0.689	27	75	63	60	38	33	124	83	28	71
**Map6**	0.4324	7.0168	7.8248	0.0052	0.4185	98	155	145	94	72	100	265	181	80	88

**Zfp532**	0.4318	4.8884	6.2565	0.0124	0.5751	111	159	173	120	50	109	195	206	86	146
**Akr7a5**	0.4301	4.7871	4.7372	0.0295	0.7616	82	87	79	112	29	85	112	96	45	146
**Rgl2**	0.4293	4.1282	3.9957	0.0456	0.8597	69	135	138	93	53	91	239	117	55	117
**Acsl4**	0.4284	5.4485	6.6453	0.0099	0.5362	116	172	189	132	60	112	158	150	105	267
**Kifc3**	0.4272	4.5226	5.6932	0.017	0.6337	141	170	195	118	93	160	253	179	87	226
**Fam89a**	0.4264	5.1941	3.8889	0.0486	0.8678	33	99	101	85	27	75	144	76	42	83
**Sipa1l2**	0.4259	4.54	6.5568	0.0104	0.5454	58	91	126	75	63	85	113	127	54	135
**Rap1gap**	0.4254	5.5718	9.0402	0.0026	0.3188	107	187	164	144	80	173	318	151	81	139
**Manf**	0.4253	6.0875	7.3715	0.0066	0.4611	245	345	462	283	165	282	492	313	192	566
**Tpm2**	0.4215	6.6972	5.4141	0.02	0.6772	575	1137	940	740	374	834	848	714	793	953
**Sac3d1**	0.4198	4.8122	6.8318	0.009	0.5138	108	134	167	93	61	134	165	179	60	156
**Ptch1**	0.4126	4.7563	4.2684	0.0388	0.8197	54	77	67	54	34	41	77	108	55	60
**Slc36a4**	0.4125	4.4063	4.6381	0.0313	0.7726	40	63	55	35	17	63	78	40	34	36
**Lyrm5**	0.411	4.3583	5.2873	0.0215	0.6958	55	78	89	78	40	80	135	75	53	74
**Porcn**	0.4085	4.5068	5.9729	0.0145	0.6057	73	163	118	70	38	91	188	107	46	128
**Ptprm**	0.4067	4.4183	4.7177	0.0299	0.7677	58	64	83	75	43	63	136	75	55	71
**Ubac2**	0.4041	4.7112	5.5659	0.0183	0.6536	101	142	92	102	80	130	185	157	42	153
**Tctex1d2**	0.4036	3.8382	5.5126	0.0189	0.662	72	90	88	75	44	94	125	88	45	102
**Acox1**	0.4034	5.5212	4.8169	0.0282	0.7493	131	124	199	130	92	178	188	131	90	243
**Pgs1**	0.403	5.6137	5.6991	0.017	0.6337	95	98	142	83	64	132	204	100	66	96
**Rpl28**	0.3989	7.5202	8.6582	0.0033	0.3445	1054	1893	1361	1250	785	1731	2095	1336	673	1996
**Rpap3**	0.3984	4.9131	6.1384	0.0132	0.5897	83	141	156	72	63	106	198	119	75	121
**Cd24a**	0.3982	7.7129	5.1059	0.0238	0.7155	665	848	590	529	382	459	766	560	533	1231
**Mapk8ip1**	0.3975	7.1831	4.0062	0.0453	0.8577	22	30	35	33	9	30	24	45	27	27
**Sec24c**	0.3971	5.8051	5.2516	0.0219	0.6995	291	372	441	319	143	268	483	389	179	571
**Ifi27**	0.3959	7.1729	10.588	0.0011	0.2213	325	626	551	493	218	539	757	455	259	647
**Coasy**	0.3959	5.5405	6.8369	0.0089	0.5138	147	200	176	142	88	227	277	135	116	147
**Apex1**	0.3952	4.7174	5.9695	0.0146	0.6057	107	122	154	111	59	122	158	157	67	165
**Snrpe**	0.3933	5.1293	4.582	0.0323	0.7806	192	209	201	148	101	173	287	179	91	328
**Dtd1**	0.3932	4.9752	6.1144	0.0134	0.5948	64	104	77	96	43	89	154	75	44	111
**Sema4d**	0.3931	5.7954	7.0309	0.008	0.4986	334	478	404	334	187	348	515	357	257	576
**Cldn23**	0.3927	5.5181	7.6662	0.0056	0.434	326	435	442	335	206	342	504	415	233	606
**Ehd3**	0.3889	8.062	9.1412	0.0025	0.3165	99	201	172	143	78	139	193	176	110	195
**Rnf126**	0.3873	5.2434	7.2773	0.007	0.4716	202	265	216	179	146	252	352	213	111	329
**Tceb1**	0.3866	6.5136	7.2308	0.0072	0.4766	274	320	262	250	121	248	351	251	182	417
**Rnf115**	0.3839	5.1248	5.7037	0.0169	0.6337	95	120	145	86	85	129	133	148	50	196
**Tmem43**	0.3801	5.1978	4.9072	0.0267	0.7377	186	344	288	216	108	269	356	213	133	368
**Sh3gl1**	0.3798	4.5588	4.665	0.0308	0.772	120	185	132	139	63	118	249	164	99	128
**Capn7**	0.3795	5.6704	7.6723	0.0056	0.434	234	238	338	186	142	249	334	253	175	335
**Nampt**	0.3757	5.1803	8.8338	0.003	0.3324	150	201	194	196	73	199	293	190	95	199
**Dpy19l4**	0.3746	4.7309	6.0526	0.0139	0.6019	77	94	113	78	62	91	152	85	52	137
**Srxn1**	0.3746	7.1378	7.332	0.0068	0.4647	151	148	158	137	67	180	211	184	91	121
**Tsg101**	0.3742	6.0599	7.01	0.0081	0.4992	258	386	361	304	182	293	516	313	171	521
**Ltb4r2**	0.3739	3.4363	5.8355	0.0157	0.6238	69	141	114	72	40	97	141	107	58	104
**Clpp**	0.3737	5.0617	7.6816	0.0056	0.434	120	156	169	154	89	152	243	170	61	225
**Pi4ka**	0.3734	5.7475	7.5045	0.0062	0.4459	85	148	146	100	67	94	232	138	67	130
**Csrp1**	0.3727	5.9448	6.2547	0.0124	0.5751	157	155	139	132	76	118	215	150	96	213
**Limk1**	0.3726	6.6669	4.4149	0.0356	0.811	28	56	52	24	27	43	68	40	28	43
**Rps27l**	0.3723	6.2016	3.8774	0.0489	0.8696	422	546	481	400	263	384	446	334	315	980
**Iars**	0.3718	5.2603	4.318	0.0377	0.8131	104	122	159	90	73	88	175	127	75	193
**Ambra1**	0.3717	4.4544	4.296	0.0382	0.8147	94	127	125	92	35	97	97	163	62	122
**Rpl37a**	0.3715	8.2798	5.635	0.0176	0.6392	2060	2385	2320	1606	1302	2130	2982	1679	1186	3798
**Rpp25l**	0.3713	4.205	4.5393	0.0331	0.7861	74	103	103	84	29	103	125	100	45	88
**Slc39a1**	0.3712	5.113	4.9413	0.0262	0.7359	193	252	223	192	139	205	375	186	164	267
**Acaca**	0.3711	5.2726	5.4871	0.0192	0.6691	136	185	193	114	90	146	212	160	95	238
**Brox**	0.3695	5.1606	6.8687	0.0088	0.5106	104	200	158	126	84	126	222	157	75	226
**Nenf**	0.3628	5.4045	4.2189	0.04	0.8266	89	119	116	71	56	96	170	78	59	135
**Klhdc3**	0.3627	5.1799	4.8186	0.0282	0.7493	92	167	118	134	36	150	111	123	75	154
**Sash1**	0.3616	5.4655	7.0468	0.0079	0.4954	85	96	107	83	35	109	117	106	39	109
**Timm10b**	0.3613	5.4954	7.8752	0.005	0.4129	131	193	198	142	106	204	205	167	106	222
**Tpi1**	0.3611	6.949	6.992	0.0082	0.4992	310	411	308	259	160	340	360	319	163	523
**Ap3s2**	0.3595	6.1253	5.8944	0.0152	0.6154	99	179	144	151	76	143	234	150	68	186

**Fadd**	0.3576	4.1255	4.4721	0.0345	0.8007	114	171	139	111	60	157	175	131	79	145
**Sharpin**	0.3572	5.1808	5.7884	0.0161	0.6285	103	154	195	113	77	156	218	157	94	122
**Rpl19**	0.3545	6.2071	4.6293	0.0314	0.7737	347	449	441	295	282	306	478	414	329	572
**Uqcr11**	0.3541	6.7347	7.94	0.0048	0.4028	290	404	412	275	167	332	515	271	183	518
**Ndrg2**	0.354	7.011	4.5044	0.0338	0.7954	72	98	86	56	38	72	114	81	60	76
**Tmem164**	0.3538	4.8402	5.5827	0.0181	0.6489	154	201	193	146	81	152	212	179	107	246
**2010107E0**	0.3503	7.8817	4.1739	0.0411	0.8353	376	504	541	354	369	422	591	381	296	896
**Plk2**	0.3487	5.1486	4.3225	0.0376	0.8131	170	349	316	241	138	250	300	304	138	409
**Abcg2**	0.3473	5.3835	3.9001	0.0483	0.8678	139	241	145	144	87	197	133	132	113	269
**Tex264**	0.3461	5.5227	6.081	0.0137	0.6008	140	184	228	172	60	171	214	202	88	225
**Zfp612**	0.3435	4.9413	5.3015	0.0213	0.6958	60	132	113	86	43	92	178	114	44	82
**Dbi**	0.3431	9.4489	7.2983	0.0069	0.4703	1635	2134	2051	2015	1280	2074	2065	2057	1346	3047
**Isca2**	0.3421	5.0172	4.9051	0.0268	0.7377	70	98	103	93	54	82	152	80	46	139
**Fth1**	0.3414	10.455	3.9518	0.0468	0.8678	4198	5833	5877	4026	2525	3418	6365	5211	2188	9489
**Psme2**	0.3409	5.1997	5.1402	0.0234	0.7084	222	254	274	208	174	299	366	230	145	317
**Rpl7a**	0.3403	7.2286	5.9159	0.015	0.612	974	1436	1304	867	490	953	1394	1031	560	1878
**Dcaf12**	0.3402	6.2115	11.82	0.0006	0.1573	406	497	491	345	175	453	621	483	211	455
**Fbrsl1**	0.3356	5.3533	4.4311	0.0353	0.8076	221	380	342	272	154	196	491	368	151	411
**Ccdc124**	0.3353	5.7603	5.8239	0.0158	0.6257	130	181	234	128	90	190	263	166	77	199
**Xbp1**	0.3351	5.8269	5.085	0.0241	0.7184	287	349	422	251	173	240	467	333	190	493
**Rnaseh2c**	0.3349	4.8265	3.9057	0.0481	0.8678	100	120	112	86	80	107	147	106	77	147
**Igf2r**	0.3321	5.0862	6.4706	0.011	0.5486	140	225	194	163	91	135	240	224	107	230
**Znrf2**	0.3318	4.7416	4.3067	0.038	0.8131	148	240	271	209	126	189	263	224	128	342
**Abr**	0.3315	5.367	4.1805	0.0409	0.8344	66	112	92	68	42	114	106	79	32	109
**Trappc13**	0.3313	4.9722	4.1619	0.0413	0.8377	122	187	139	101	60	112	204	121	69	197
**Uqcc2**	0.3305	6.5221	4.8645	0.0274	0.7467	231	389	330	181	178	236	305	285	177	484
**Mrps9**	0.3303	5.0944	4.4959	0.034	0.7963	130	128	102	90	72	121	182	119	48	161
**Wdr3**	0.3303	4.5997	4.3077	0.0379	0.8131	98	158	167	90	46	109	234	124	52	131
**Lage3**	0.3295	4.908	5.6378	0.0176	0.6392	181	235	214	195	88	221	287	249	98	205
**Cstb**	0.3295	7.5103	4.5803	0.0323	0.7809	1281	1564	1544	1178	788	1406	1650	1216	801	2273
**Ncbp2**	0.3278	5.8572	5.4355	0.0197	0.6748	207	290	262	224	169	228	307	260	200	315
**Flot2**	0.3273	6.7012	5.0658	0.0244	0.7207	75	132	105	84	38	120	142	114	47	73
**Adrm1**	0.3233	4.9739	5.4702	0.0193	0.6697	107	144	143	103	83	149	187	107	86	139
**Cox10**	0.3226	5.438	6.2508	0.0124	0.5751	129	158	175	113	70	126	198	144	109	149
**Agtpbp1**	0.3223	7.5747	5.0572	0.0245	0.7214	57	123	89	66	46	78	158	86	46	73
**Slc4a11**	0.3214	7.4873	4.9259	0.0265	0.7377	799	2145	1930	1632	831	1642	2514	1756	798	1724
**Fbxo31**	0.3211	6.53	6.0288	0.0141	0.604	64	118	97	80	44	83	133	107	34	110
**Ndufc2**	0.3208	7.0244	4.6084	0.0318	0.7776	279	385	299	248	226	284	379	286	210	507
**Zfp668**	0.3207	4.4781	4.0769	0.0435	0.85	76	108	143	101	49	136	179	79	51	106
**Ubc**	0.3195	9.4279	3.9096	0.048	0.8678	2477	5144	5805	4592	1839	4007	5566	5256	1943	5752
**Tmem41a**	0.3187	5.4557	6.663	0.0098	0.5336	215	295	243	196	160	257	329	220	173	288
**1810058I2**	0.3184	5.7963	5.1782	0.0229	0.7051	81	106	144	87	70	104	156	121	52	137
**Tox4**	0.3182	5.0803	4.2385	0.0395	0.823	139	229	196	126	56	162	211	175	82	200
**Higd1a**	0.3169	5.6706	4.7042	0.0301	0.7694	101	157	105	78	76	96	123	128	79	160
**Enpp5**	0.3155	5.6829	4.1483	0.0417	0.8381	86	118	115	71	33	68	108	114	55	127
**Rpl30**	0.3121	7.4404	5.7051	0.0169	0.6337	1201	1398	1426	1128	700	1198	1798	1165	666	1985
**Zmiz2**	0.3115	5.5847	5.848	0.0156	0.6238	247	335	287	224	130	231	442	279	125	346
**Madd**	0.3112	5.6355	4.2751	0.0387	0.8192	157	192	134	105	86	111	235	134	125	153
**Ifitm1**	0.3112	8.0216	4.2646	0.0389	0.8206	2206	2464	2368	1801	1116	1617	2831	2126	1741	2588
**Rpl22l1**	0.3099	5.9204	4.6925	0.0303	0.7699	393	484	419	351	160	418	570	428	164	502
**Ptgr2**	0.3095	4.711	5.649	0.0175	0.6381	116	168	171	137	67	131	233	141	76	174
**Kif1bp**	0.3094	5.7873	4.3336	0.0374	0.8131	103	110	111	69	82	79	147	125	55	155
**Myl6**	0.3089	7.3087	4.3179	0.0377	0.8131	735	884	889	710	475	727	1040	702	460	1330
**Rps10**	0.3085	8.616	3.9827	0.046	0.8611	2399	2977	2874	2614	1890	2690	3281	2353	1685	4809
**Tmem230**	0.3077	5.1182	4.4259	0.0354	0.8091	105	153	112	84	63	111	129	109	64	171
**Tjp3**	0.3075	5.3896	5.0424	0.0247	0.7217	299	452	425	308	213	283	607	411	174	517
**Cdk2ap1**	0.3064	4.6169	4.1539	0.0415	0.8377	108	152	113	119	40	121	160	134	58	127
**Fgfr1**	0.3064	6.2628	4.3018	0.0381	0.8131	56	134	146	110	40	101	136	90	52	162
**Pnrc1**	0.3013	5.3568	4.0686	0.0437	0.851	129	245	149	136	88	123	224	249	60	210
**Ptpra**	0.3002	6.5519	6.7888	0.0092	0.5176	302	406	291	285	143	357	404	396	172	274
**Tomm70a**	0.3	6.7109	5.2711	0.0217	0.6958	274	275	330	186	158	222	331	277	181	364
**Cct3**	0.3	6.8729	6.0076	0.0142	0.604	394	499	507	443	224	468	590	477	210	624

**Tm9sf2**	0.2991	6.3513	5.4751	0.0193	0.6697	354	454	377	360	199	261	640	428	199	503
**Mgat4b**	0.2972	5.217	3.8683	0.0492	0.8715	85	134	141	86	54	98	159	155	47	110
**Lpin1**	0.2969	6.575	4.6746	0.0306	0.7712	104	105	87	92	60	89	120	130	55	120
**Wdtc1**	0.2952	6.0067	6.7238	0.0095	0.525	347	449	419	349	199	394	642	353	191	451
**Dctn4**	0.2945	6.412	5.2384	0.0221	0.6995	212	283	270	201	167	228	355	280	131	319
**Cnot2**	0.2911	5.2466	4.3901	0.0361	0.811	181	262	239	197	101	187	273	205	142	269
**Rplp0**	0.2907	8.7677	4.8132	0.0282	0.7493	2644	3770	3896	2921	2327	3374	4587	3137	1764	5237
**D10Jhu81e**	0.2905	6.1783	5.0959	0.024	0.7183	243	350	312	222	141	276	418	261	133	351
**Aqp1**	0.2892	8.2278	6.5264	0.0106	0.546	1345	2919	3065	2108	1305	2415	3549	2988	1087	2172
**Asprv1**	0.2891	11.091	4.715	0.0299	0.7684	18063	21985	23481	19099	11259	21192	25534	22978	11611	24391
**H3f3b**	0.2889	8.2624	4.9806	0.0256	0.7319	1986	2704	2350	1866	1172	1971	2658	2217	1123	3407
**Eif3d**	0.2883	6.661	7.2687	0.007	0.4716	497	585	584	553	292	590	800	587	313	560
**Pdpn**	0.288	6.8428	4.46	0.0347	0.8027	559	961	750	907	320	704	1057	922	448	727
**Csnk1g3**	0.288	5.9505	5.262	0.0218	0.6976	230	331	300	251	173	307	352	295	151	351
**Senp7**	0.2868	4.8937	4.1963	0.0405	0.8329	110	168	144	115	84	117	183	185	69	153
**Prdx1**	0.286	7.7186	4.9978	0.0254	0.7274	1077	1461	1318	1032	586	1236	1397	1128	588	1759
**Psma6**	0.2809	7.0251	6.9096	0.0086	0.5048	526	678	592	406	316	607	632	609	279	695
**Dtx3l**	0.2807	5.7825	7.4848	0.0062	0.4459	395	513	482	455	236	387	636	455	261	599
**Pdcd5**	0.2804	6.0022	4.474	0.0344	0.8003	258	331	220	222	161	304	300	245	169	306
**Gtf2i**	0.2799	6.5389	4.681	0.0305	0.7712	292	376	429	259	188	320	419	349	144	519
**Mrpl11**	0.2789	5.3437	4.2269	0.0398	0.8246	125	172	127	101	70	117	174	156	66	155
**Aldh3b2**	0.2784	6.6932	6.5738	0.0103	0.5435	798	1091	991	743	520	914	1053	934	532	1151
**Dctn6**	0.2763	5.9567	5.0657	0.0244	0.7207	223	324	226	200	123	205	317	268	139	286
**Acat2**	0.2752	6.6852	6.0111	0.0142	0.604	302	539	493	444	188	468	528	518	184	479
**Dazap2**	0.273	7.3884	5.0252	0.025	0.7219	1031	1328	1300	914	548	897	1404	1234	557	1607
**Nop16**	0.2724	4.9147	4.1921	0.0406	0.8329	135	160	166	127	65	128	236	181	69	122
**Otud5**	0.272	5.3076	4.8936	0.027	0.7403	158	239	234	174	98	176	283	224	102	219
**2410015M**	0.2713	5.9651	5.6574	0.0174	0.6381	343	459	391	309	205	419	538	297	197	457
**Myeov2**	0.2664	7.7162	4.4637	0.0346	0.8027	519	721	557	462	384	582	776	455	340	813
**Rap2c**	0.2644	5.3315	5.0238	0.025	0.7219	129	159	179	138	92	159	246	157	70	157
**Tmsb4x**	0.2642	12.325	4.6248	0.0315	0.7738	36652	43220	44653	36924	22121	35277	49614	33989	24003	59386
**Med16**	0.2637	5.1663	4.6405	0.0312	0.7726	141	179	214	160	83	171	277	187	79	157
**Eif1ax**	0.2618	6.0547	4.3043	0.038	0.8131	245	400	335	302	164	316	411	246	178	429
**Aldh9a1**	0.2606	5.6098	4.2315	0.0397	0.8235	126	231	195	199	90	183	267	217	80	190
**Lsm3**	0.2596	5.585	4.0491	0.0442	0.8548	260	322	326	213	163	303	366	278	166	296
**Zfand3**	0.2587	6.1036	3.9335	0.0473	0.8678	292	441	356	353	171	313	421	356	198	467
**Elf3**	0.2576	7.8841	4.9628	0.0259	0.7335	2048	2781	2418	1670	1116	2093	3156	2005	1053	2828
**Cysrt1**	0.2575	6.4582	4.2697	0.0388	0.8196	656	925	945	689	548	833	1067	796	490	991
**Hspb1**	0.2574	8.4923	4.2218	0.0399	0.8261	2519	3340	3312	2271	1523	2505	3716	2628	1394	4147
**Bsg**	0.2541	8.3779	5.5877	0.0181	0.6487	1431	2454	2093	1578	1026	1662	2324	1877	894	2693
**Timm17a**	0.2539	6.073	4.193	0.0406	0.8329	176	222	222	192	99	243	239	179	119	205
**Mdp1**	0.2536	5.9027	5.0504	0.0246	0.7214	312	405	358	310	164	348	557	400	154	276
**Uba52**	0.2521	6.9437	4.6884	0.0304	0.7699	854	968	944	638	478	916	1167	769	451	993
**Romo1**	0.2495	6.7735	4.1038	0.0428	0.845	431	471	477	421	204	454	611	434	190	545
**Trove2**	0.2486	6.2844	5.7193	0.0168	0.6337	292	377	346	235	156	342	435	293	146	328
**Itm2c**	0.2485	7.9307	6.0155	0.0142	0.604	455	742	630	484	280	520	862	613	263	598
**2310007B0**	0.2478	7.2007	5.7412	0.0166	0.6322	1308	1503	1702	1297	674	1109	2149	1683	736	1492
**Rps7**	0.2449	7.9259	4.6633	0.0308	0.772	1555	2229	2017	1500	1196	1964	2285	1749	990	2395
**Slc3a2**	0.2422	6.9962	4.4157	0.0356	0.811	558	827	702	545	215	509	859	719	298	656
**Peli1**	0.2374	5.5687	4.1375	0.0419	0.8418	214	264	255	198	114	175	322	227	113	308
**Emd**	0.236	5.8181	4.0168	0.0451	0.8577	310	359	387	285	174	384	358	373	168	351
**Rps13**	0.2339	8.9246	4.7803	0.0288	0.7541	3511	4107	4403	3202	2084	3855	4685	3415	1778	5286
**Ech1**	0.2338	6.3261	4.6944	0.0303	0.7699	377	556	512	433	245	454	628	426	223	585
**Pcmt1**	0.2325	6.9294	4.6564	0.0309	0.7726	190	287	283	177	125	219	299	241	114	273
**Vapa**	0.2316	7.318	4.5461	0.033	0.7852	567	888	925	673	379	672	795	747	390	1039
**Srsf3**	0.2281	7.0924	4.6845	0.0304	0.7706	736	1014	1004	697	447	762	1072	834	428	1104
**Sec31a**	0.223	6.76	5.0244	0.025	0.7219	506	684	613	547	271	568	838	664	270	504
**Eif3f**	0.2226	7.9159	4.0117	0.0452	0.8577	1543	2016	2143	1468	774	1636	2078	1533	821	2361
**Mgat4a**	0.2222	5.7947	6.3155	0.012	0.5636	331	468	411	299	167	331	484	399	189	382
**Rbx1**	0.2211	7.1775	4.0162	0.0451	0.8577	481	628	583	475	267	464	703	517	245	711
**Mrps23**	0.2201	5.9281	4.3552	0.0369	0.8131	301	338	353	272	137	284	465	303	141	327
**Dnpep**	0.22	6.1739	5.367	0.0205	0.6861	344	497	456	382	199	361	568	380	238	448

**Ndufb8**	0.2119	8.2806	4.6358	0.0313	0.7726	694	809	767	592	440	749	915	705	365	849
**Acly**	0.2109	7.804	4.3107	0.0379	0.8131	755	1169	1252	938	532	828	1342	994	517	1270
**Hspa5**	0.201	8.9417	4.67	0.0307	0.772	2152	3169	3174	2288	1263	2373	3301	2660	1471	2652
**Tsn**	0.2007	7.8372	4.5915	0.0321	0.7798	785	983	977	805	436	850	1248	983	352	891
**Eif3g**	0.19	6.8086	4.1616	0.0413	0.8377	604	730	767	569	349	647	891	592	347	702
**Puf60**	0.181	7.2924	3.9947	0.0456	0.8597	615	793	727	531	338	590	903	589	378	651
**Downregulated genes**															
**Crybb1**	-5.8537	2.6156	23.178	1E-06	0.0158	205	59	0	0	0	0	0	1	2	0
**Alox12e**	-5.7134	1.8953	14.239	0.0002	0.0813	415	0	2	1	7	2	0	2	1	2
**Lgsn**	-5.6375	0.1608	7.2142	0.0072	0.4784	78	5	0	0	0	0	0	1	0	0
**Crygc**	-5.5019	2.9154	17.11	4E-05	0.0415	799	183	7	2	0	5	0	10	2	2
**Cplx4**	-5.434	-0.4588	8.2151	0.0042	0.3832	25	2	3	0	0	0	0	0	0	0
**Crygd**	-5.4191	2.6982	11.981	0.0005	0.151	711	107	1	3	0	0	0	6	7	0
**Sult1c1**	-5.2849	-0.5745	5.459	0.0195	0.6702	21	0	0	0	3	0	0	0	0	0
**Zfp385b**	-5.1447	5.4046	31.474	2E-08	0.0004	20	0	0	5	0	0	0	0	0	0
**Crygb**	-5.0943	3.7334	14.103	0.0002	0.0841	1512	272	4	7	3	6	10	23	8	0
**Ajap1**	-5.0285	0.4295	12.167	0.0005	0.1486	0	2	3	0	12	0	0	0	0	0
**Nrl**	-4.998	-0.4131	5.2265	0.0222	0.6995	21	0	0	0	0	0	0	0	0	0
**Rorb**	-4.9942	0.8477	18.688	2E-05	0.0264	18	2	2	0	0	0	0	0	0	0
**BC040756**	-4.9311	-0.3597	6.8548	0.0088	0.5121	0	0	0	3	11	0	0	0	0	0
**Aipl1**	-4.8726	-0.136	10.427	0.0012	0.2316	31	18	1	4	0	0	0	0	0	1
**Arl4aos**	-4.8286	-0.5825	11.335	0.0008	0.1795	6	8	5	0	2	0	0	0	0	0
**Gm15446**	-4.8043	0.0484	11.654	0.0006	0.1658	11	8	0	0	1	0	0	0	0	0
**Cryba2**	-4.7675	2.8083	12.16	0.0005	0.1486	544	299	0	1	0	4	0	15	5	0
**Serpine2**	-4.7588	6.2714	20.969	5E-06	0.0162	176	0	1	0	15	0	8	0	0	0
**Ermn**	-4.7251	4.1184	20.855	5E-06	0.0162	18	0	0	0	0	0	0	0	0	0
**Adra1d**	-4.6668	-0.6319	4.9516	0.0261	0.7335	4	0	0	0	8	0	0	0	0	0
**Hist1h4m**	-4.659	-0.0948	10.725	0.0011	0.2112	3	0	0	5	6	0	0	0	0	0
**LOC105247**	-4.6411	-0.5402	6.005	0.0143	0.604	0	3	0	5	6	0	0	0	0	0
**Gnat2**	-4.602	-0.1326	11.225	0.0008	0.1804	5	12	0	2	0	0	0	0	0	0
**Rp1**	-4.5564	0.056	10.411	0.0013	0.2316	45	6	7	0	3	0	1	0	0	1
**Crybb2**	-4.5432	3.5299	13.905	0.0002	0.0876	1100	394	1	0	0	8	14	25	9	0
**Cryaa**	-4.5285	3.7426	10.101	0.0015	0.2474	1167	690	24	6	0	12	0	26	19	0
**5033417F2**	-4.5234	0.4483	14.342	0.0002	0.0808	1	4	7	4	1	0	0	0	0	0
**Gm42072**	-4.5216	-0.621	6.3267	0.0119	0.5628	0	0	5	2	6	0	0	0	0	0
**Astn2**	-4.4805	3.6378	19.565	1E-05	0.0206	0	9	2	4	1	0	0	0	0	0
**Gm40491**	-4.4752	-0.6178	6.3863	0.0115	0.5577	1	5	13	0	0	0	0	0	0	0
**Gm31963**	-4.4656	-0.3769	6.82	0.009	0.5147	5	13	0	0	0	0	0	0	0	0
**LOC10263**	-4.4423	-0.0603	11.61	0.0007	0.1674	7	3	1	0	3	0	0	0	0	0
**2810408B1**	-4.4158	-0.5989	5.7703	0.0163	0.6285	0	0	0	0	9	0	0	0	0	0
**Gm41668**	-4.4138	-0.2254	10.424	0.0012	0.2316	2	0	6	3	3	0	0	0	0	0
**Gper1**	-4.4133	0.405	14.847	0.0001	0.0688	2	0	7	5	1	0	0	0	0	0
**Xlr3a**	-4.4065	0.3731	9.0961	0.0026	0.3165	2	0	4	0	6	0	0	0	0	0
**Ciita**	-4.4018	1.3129	15.615	8E-05	0.055	5	3	3	4	0	0	0	0	0	0
**Col10a1**	-4.3655	-0.3152	8.8685	0.0029	0.3294	0	1	10	2	2	0	0	0	0	0
**4931428F0**	-4.3529	1.1713	15.136	0.0001	0.0631	0	2	0	5	6	0	0	0	0	0
**Rpe65**	-4.341	-0.0405	4.4379	0.0352	0.8066	33	0	0	0	0	0	0	0	0	1
**Xist**	-4.3326	-0.366	7.0852	0.0078	0.4918	0	6	5	5	0	0	0	0	0	0
**Aadac**	-4.3325	-0.0961	13.018	0.0003	0.1116	26	2	1	4	1	1	0	0	0	0
**Dhh**	-4.3168	5.5734	18.047	2E-05	0.031	1	2	4	6	1	0	0	0	0	0
**Cysltr1**	-4.3015	0.2999	10.671	0.0011	0.2152	2	1	0	2	6	0	0	0	0	0
**5430427M**	-4.2848	-0.6068	8.2354	0.0041	0.3832	7	1	0	5	0	0	0	0	0	0
**Wdr17**	-4.2811	3.1305	16.703	4E-05	0.0442	0	8	4	1	1	0	0	0	0	0
**Xrra1**	-4.2706	-0.4014	11.678	0.0006	0.1658	0	5	3	5	1	0	0	0	0	0
**Gm31420**	-4.2697	-0.5697	6.2516	0.0124	0.5751	5	0	0	3	3	0	0	0	0	0
**Crygs**	-4.2588	3.3231	17.085	4E-05	0.0415	805	279	1	3	0	7	0	15	15	4
**4930500A**	-4.2248	-0.219	8.8647	0.0029	0.3294	0	6	0	2	4	0	0	0	0	0
**LOC10816**	-4.2231	-0.0646	11.846	0.0006	0.1563	1	6	5	1	1	0	0	0	0	0
**LOC10816**	-4.2215	-0.6948	4.5465	0.033	0.7852	0	6	0	8	0	0	0	0	0	0
**Ina**	-4.2057	3.6798	16.437	5E-05	0.0459	2	4	6	0	1	0	0	0	0	0
**Hist1h2ad**	-4.196	-0.6904	11.037	0.0009	0.1888	4	3	2	0	3	0	0	0	0	0
**Gm39412**	-4.1902	-0.5032	7.3301	0.0068	0.4647	4	0	10	0	0	0	0	0	0	0

**Gm11744**	-4.1858	0.5224	11.478	0.0007	0.1735	5	0	4	1	2	0	0	0	0	0
**Pnliprp2**	-4.1213	-0.6299	5.9725	0.0145	0.6057	0	7	6	0	1	0	0	0	0	0
**D630024D(**	-4.1123	-0.3307	7.9086	0.0049	0.4069	3	2	3	0	3	0	0	0	0	0
**Cryge**	-4.1103	-0.2577	4.0202	0.045	0.8577	38	2	0	0	0	0	2	0	0	0
**Gm30575**	-4.0998	0.1703	10.645	0.0011	0.217	4	9	0	0	0	0	0	0	0	0
**Gm10384**	-4.0966	-0.5256	4.0275	0.0448	0.8574	9	0	0	2	0	0	0	0	0	0
**Gm4876**	-4.096	-0.5453	10.223	0.0014	0.2411	5	4	0	3	0	0	0	0	0	0
**C330024D2**	-4.0891	0.2662	9.7627	0.0018	0.2615	4	0	5	3	0	0	0	0	0	0
**Gm35658**	-4.076	0.1875	8.9964	0.0027	0.3201	0	2	0	4	4	0	0	0	0	0
**Dscaml1**	-4.0729	1.4247	11.504	0.0007	0.1723	11	0	0	0	0	0	0	0	0	0
**Gm10710**	-4.0723	-0.1708	8.4031	0.0037	0.3726	0	12	1	1	0	0	0	0	0	0
**Gm39443**	-4.0629	-0.3657	5.2308	0.0222	0.6995	1	0	0	10	0	0	0	0	0	0
**Gm15934**	-4.0491	-0.6541	4.9241	0.0265	0.7377	6	0	0	0	3	0	0	0	0	0
**Hist1h4k**	-4.0297	-0.7573	4.341	0.0372	0.8131	0	0	0	6	3	0	0	0	0	0
**Ptgs2os2**	-4.0223	-0.6267	8.5674	0.0034	0.3548	0	1	6	5	0	0	0	0	0	0
**Slc9b1**	-4.0208	0.0655	6.9965	0.0082	0.4992	0	13	1	0	0	0	0	0	0	0
**Hsf5**	-3.9937	-0.2899	7.2993	0.0069	0.4703	2	4	3	0	2	0	0	0	0	0
**Tmem59l**	-3.9914	5.433	14.199	0.0002	0.0813	3	0	2	6	0	0	0	0	0	0
**Gm36012**	-3.9904	0.3662	19.904	8E-06	0.0206	3	13	7	4	14	0	0	1	0	1
**Psmb11**	-3.9889	-0.3707	6.8262	0.009	0.5138	1	0	3	7	0	0	0	0	0	0
**Ablim3**	-3.9864	3.8246	13.932	0.0002	0.0874	1	4	0	6	0	0	0	0	0	0
**Pi16**	-3.9713	0.1847	6.8351	0.0089	0.5138	10	0	0	0	0	0	0	0	0	0
**Ckm**	-3.9576	-0.3574	6.1829	0.0129	0.5845	0	0	9	0	2	0	0	0	0	0
**LOC108167**	-3.95	-0.7277	7.2671	0.007	0.4716	6	2	3	0	0	0	0	0	0	0
**Clec14a**	-3.9449	0.5915	11.263	0.0008	0.1801	2	4	0	0	4	0	0	0	0	0
**Lrtm1**	-3.9421	-0.4403	5.88	0.0153	0.6176	9	0	1	0	0	0	0	0	0	0
**LOC10816ɛ**	-3.9267	-0.4527	7.2604	0.007	0.4722	3	0	0	4	2	0	0	0	0	0
**1700015E1**	-3.9254	-0.4482	6.3692	0.0116	0.5577	0	3	4	0	3	0	0	0	0	0
**Gm10433**	-3.9236	-0.7237	6.9247	0.0085	0.5048	2	0	3	4	1	0	0	0	0	0
**Gm6329**	-3.9234	-0.8494	5.2852	0.0215	0.6958	0	0	1	3	4	0	0	0	0	0
**Meox1**	-3.9198	1.8613	11.636	0.0006	0.1663	10	0	0	0	0	0	0	0	0	0
**Gm36591**	-3.8998	-0.4667	7.1757	0.0074	0.4825	0	6	1	0	3	0	0	0	0	0
**Erich6**	-3.897	0.2108	7.6475	0.0057	0.434	1	5	0	2	2	0	0	0	0	0
**4930407I0**	-3.8929	0.3065	9.4477	0.0021	0.2865	0	12	0	0	0	0	0	0	0	0
**Rho**	-3.8887	2.5792	16.864	4E-05	0.0438	364	15	24	5	9	9	8	0	0	12
**Gata1**	-3.8827	-0.6452	5.7118	0.0169	0.6337	0	3	3	5	0	0	0	0	0	0
**Gm32707**	-3.8595	-0.8108	6.325	0.0119	0.5628	2	0	0	3	3	0	0	0	0	0
**Gm39098**	-3.859	-0.6603	6.9251	0.0085	0.5048	3	1	2	4	0	0	0	0	0	0
**9630013D2**	-3.8561	0.2424	10.359	0.0013	0.2316	1	8	2	0	0	0	0	0	0	0
**Cyp4a14**	-3.8515	-0.8095	5.3819	0.0203	0.6835	1	0	1	0	5	0	0	0	0	0
**Frmd7**	-3.8513	0.9653	10.688	0.0011	0.2144	1	4	1	3	1	0	0	0	0	0
**Chad**	-3.8402	0.5817	8.1679	0.0043	0.3886	35	0	0	3	0	1	0	1	0	0
**LOC105246**	-3.8246	-0.6583	8.1192	0.0044	0.392	0	2	2	6	0	0	0	0	0	0
**Cryba1**	-3.8188	3.6716	8.2354	0.0041	0.3832	1094	588	11	4	0	14	28	33	22	0
**Snord42a**	-3.8091	-0.587	5.4274	0.0198	0.6761	3	0	0	0	4	0	0	0	0	0
**Gm40588**	-3.8088	-0.5948	7.9679	0.0048	0.4019	4	3	2	1	0	0	0	0	0	0
**Gm36317**	-3.8061	-0.3212	5.6792	0.0172	0.6364	0	9	1	0	1	0	0	0	0	0
**D17Ertd64**	-3.8038	-0.7984	6.3718	0.0116	0.5577	2	0	5	3	0	0	0	0	0	0
**Gm35200**	-3.8037	-0.4287	6.71	0.0096	0.525	3	3	1	0	2	0	0	0	0	0
**Gm33753**	-3.7896	-0.6895	8.0074	0.0047	0.4019	3	2	0	3	1	0	0	0	0	0
**Wisp2**	-3.7858	-0.4421	5.9951	0.0143	0.6045	14	0	5	0	3	0	0	0	0	1
**Ttr**	-3.7851	2.06	9.6521	0.0019	0.2722	287	8	13	1	0	15	0	1	0	5
**Gm39011**	-3.7796	-0.7932	6.0911	0.0136	0.6001	0	2	0	3	3	0	0	0	0	0
**Sun3**	-3.7757	-0.1958	9.5049	0.002	0.2825	1	3	2	0	3	0	0	0	0	0
**Flnc**	-3.7754	0.7485	9.1013	0.0026	0.3165	0	0	0	4	4	0	0	0	0	0
**Asgr1**	-3.7658	1.6832	10.078	0.0015	0.2478	1	0	2	5	1	0	0	0	0	0
**E030003E1**	-3.7644	-0.471	6.0363	0.014	0.604	1	6	4	0	0	0	0	0	0	0
**Gm5464**	-3.7587	-0.4239	5.2982	0.0213	0.6958	0	0	1	8	0	0	0	0	0	0
**Mcoln2**	-3.7516	-0.5652	6.5389	0.0106	0.5456	4	6	0	0	0	0	0	0	0	0
**Tmem72**	-3.7474	3.7131	10.924	0.0009	0.1951	8	1	0	0	0	0	0	0	0	0
**Bpifb9a**	-3.7436	-0.4979	7.1297	0.0076	0.4871	0	6	0	4	0	0	0	0	0	0
**1700016J1**	-3.741	-0.3233	5.8401	0.0157	0.6238	4	0	2	0	2	0	0	0	0	0

**Slc6a13**	-3.7316	3.0848	11.29	0.0008	0.1801	2	1	0	6	0	0	0	0	0	0
**LOC10816**	-3.7202	-0.1563	6.471	0.011	0.5486	0	2	0	7	0	0	0	0	0	0
**Hmgcs2**	-3.7152	5.2192	11.557	0.0007	0.171	1	0	7	0	1	0	0	0	0	0
**Nkain1**	-3.7141	3.301	11.205	0.0008	0.1805	5	4	0	0	0	0	0	0	0	0
**Gm36486**	-3.7137	-0.5448	7.4903	0.0062	0.4459	0	1	6	0	2	0	0	0	0	0
**LOC10263**	-3.7124	0.0632	7.6258	0.0058	0.4358	0	0	9	1	0	0	0	0	0	0
**Gm41055**	-3.7122	0.1683	12.905	0.0003	0.1145	2	6	6	2	6	0	0	0	0	1
**Gria4**	-3.7047	4.9637	11.41	0.0007	0.1775	0	0	9	0	0	0	0	0	0	0
**Cryba4**	-3.6984	1.9539	9.8694	0.0017	0.261	243	81	9	13	0	9	0	0	4	8
**Fgfr4**	-3.6976	-0.6431	3.8634	0.0493	0.8716	0	2	5	3	0	0	0	0	0	0
**Apobr**	-3.6865	-0.6258	4.6016	0.0319	0.7798	5	0	4	0	0	0	0	0	0	0
**Matk**	-3.6821	0.5236	8.9122	0.0028	0.3266	0	7	0	0	2	0	0	0	0	0
**Stmn1-rs1**	-3.6779	-0.7041	5.08	0.0242	0.7184	0	3	0	0	4	0	0	0	0	0
**Tnn**	-3.6708	-0.4423	4.3154	0.0378	0.8131	2	0	0	0	4	0	0	0	0	0
**Casp4**	-3.656	0.2037	7.7168	0.0055	0.4316	8	0	0	0	0	0	0	0	0	0
**Snora7a**	-3.6503	0.6559	13.335	0.0003	0.107	14	3	0	3	1	0	0	1	0	0
**Gm40617**	-3.6437	-0.6737	5.7271	0.0167	0.6337	0	4	0	5	0	0	0	0	0	0
**Slc7a15**	-3.6319	-0.144	5.9912	0.0144	0.6045	0	0	0	8	0	0	0	0	0	0
**Vax2**	-3.6318	-0.7282	4.1507	0.0416	0.8381	1	8	0	1	0	0	0	0	0	0
**Gm30918**	-3.6315	-0.7215	3.8895	0.0486	0.8678	0	0	0	8	0	0	0	0	0	0
**Gm33325**	-3.6307	-0.7606	5.5248	0.0187	0.6593	0	1	4	4	0	0	0	0	0	0
**Gm29940**	-3.6172	-0.4067	5.5559	0.0184	0.6548	0	2	0	2	3	0	0	0	0	0
**Gck**	-3.6151	0.3063	7.9826	0.0047	0.4019	1	2	0	4	1	0	0	0	0	0
**Acat3**	-3.6137	-0.406	6.6077	0.0102	0.543	0	9	1	0	0	0	0	0	0	0
**Gm38908**	-3.6135	-0.5718	7.573	0.0059	0.4423	3	0	3	1	1	0	0	0	0	0
**Myrf**	-3.6064	1.7636	9.7118	0.0018	0.2651	8	0	0	0	0	0	0	0	0	0
**Gm39666**	-3.6017	1.6381	9.8118	0.0017	0.261	8	0	0	0	0	0	0	0	0	0
**Pkd2l2**	-3.567	0.4274	8.9299	0.0028	0.3251	0	4	1	1	2	0	0	0	0	0
**4933413J0**	-3.5657	-0.5734	6.5835	0.0103	0.5435	0	2	2	3	1	0	0	0	0	0
**Kcnh5**	-3.5619	1.675	9.1686	0.0025	0.3163	0	5	0	1	2	0	0	0	0	0
**Gdap1l1**	-3.5596	4.8342	10.324	0.0013	0.2316	0	4	0	4	0	0	0	0	0	0
**Il1rl1**	-3.5596	0.7182	8.7633	0.0031	0.3382	0	0	3	5	0	0	0	0	0	0
**Gm31902**	-3.5594	-0.5913	7.6816	0.0056	0.434	2	0	3	3	0	0	0	0	0	0
**Cela1**	-3.5581	3.2141	10.189	0.0014	0.2424	4	4	0	0	0	0	0	0	0	0
**Gm38637**	-3.5563	-0.7983	6.9311	0.0085	0.5048	0	4	2	0	2	0	0	0	0	0
**Gm31473**	-3.5544	-0.5435	4.9114	0.0267	0.7377	3	0	0	0	3	0	0	0	0	0
**A930016O**	-3.5506	-0.657	3.8644	0.0493	0.8716	1	2	0	5	0	0	0	0	0	0
**Gm38760**	-3.5496	-0.7253	6.7547	0.0094	0.5215	1	0	3	4	0	0	0	0	0	0
**LOC10816**	-3.5416	-0.7941	4.5955	0.0321	0.7798	2	0	3	0	2	0	0	0	0	0
**Mpp4**	-3.5336	-0.4393	4.5493	0.0329	0.7852	5	3	0	0	0	0	0	0	0	0
**Gm36243**	-3.5301	0.3476	9.819	0.0017	0.261	8	15	16	0	1	0	0	0	1	1
**Gm41113**	-3.5282	-0.1565	6.8959	0.0086	0.5068	0	4	5	0	0	0	0	0	0	0
**Itgae**	-3.5217	0.0118	5.7766	0.0162	0.6285	0	4	0	0	3	0	0	0	0	0
**Slc26a1**	-3.5208	0.0972	6.3602	0.0117	0.5582	0	0	0	6	1	0	0	0	0	0
**Gm38732**	-3.5186	0.9412	8.1612	0.0043	0.3886	5	0	0	0	2	0	0	0	0	0
**Gm40320**	-3.5179	-0.4671	5.9661	0.0146	0.6057	2	0	4	2	0	0	0	0	0	0
**Ano7**	-3.5163	-0.2266	6.8578	0.0088	0.5121	0	1	5	1	1	0	0	0	0	0
**Gm41450**	-3.5115	-0.6583	4.7598	0.0291	0.7563	3	0	4	1	0	0	0	0	0	0
**Gm10560**	-3.5085	-0.1215	6.2337	0.0125	0.5758	1	0	1	0	4	0	0	0	0	0
**Rlbp1**	-3.5083	2.7119	18.511	2E-05	0.0276	37	2	2	0	1	1	0	1	0	1
**Klk14**	-3.5056	-0.4533	6.4165	0.0113	0.5538	4	4	0	0	0	0	0	0	0	0
**Nlrp10**	-3.4969	-0.5603	4.4902	0.0341	0.7976	7	0	0	0	0	0	0	0	0	0
**Pde6a**	-3.4926	0.2199	6.3208	0.0119	0.5634	50	0	10	0	0	1	3	0	0	1
**LOC10524**	-3.4852	-0.4002	4.5537	0.0328	0.7852	4	4	0	0	0	0	0	0	0	0
**Gm32694**	-3.4833	-0.3054	5.6483	0.0175	0.6381	0	0	7	0	1	0	0	0	0	0
**Gm40187**	-3.4815	-0.5312	6.971	0.0083	0.5043	1	3	2	2	0	0	0	0	0	0
**Lrcol1**	-3.4739	-0.8133	4.3014	0.0381	0.8131	3	0	0	4	0	0	0	0	0	0
**Prkag2os1**	-3.4514	-0.542	4.9354	0.0263	0.7367	3	0	6	1	6	0	0	0	0	1
**Gdf10**	-3.4511	0.8003	8.0172	0.0046	0.4007	7	0	0	0	0	0	0	0	0	0
**Gm12315**	-3.4508	-0.2127	4.4885	0.0341	0.7977	0	3	0	7	6	1	0	0	0	0
**Fam169b**	-3.4489	-0.0817	9.9614	0.0016	0.2567	2	0	9	2	5	1	0	0	0	0
**Arhgef15**	-3.4458	1.4031	8.0898	0.0045	0.3937	6	0	0	1	0	0	0	0	0	0

**4930447F2**	-3.444	-0.7822	4.3619	0.0368	0.8127	0	6	3	0	0	0	0	0	0	0
**Lrrc23**	-3.443	-0.1483	5.5599	0.0184	0.6548	0	0	0	0	5	0	0	0	0	0
**Hist1h2ai**	-3.4341	-0.6882	4.4036	0.0359	0.811	4	1	0	2	0	0	0	0	0	0
**Gm38609**	-3.4332	0.414	12.094	0.0005	0.1491	4	11	17	3	0	1	0	1	0	0
**Cngb1**	-3.4145	0.0965	9.5518	0.002	0.2785	12	2	2	2	1	0	0	0	1	0
**Gm38805**	-3.4134	-0.5101	5.2217	0.0223	0.6995	0	4	1	0	2	0	0	0	0	0
**Ptgfr**	-3.4071	2.9946	8.8879	0.0029	0.3278	7	0	0	0	0	0	0	0	0	0
**LOC102637**	-3.4027	-0.6737	5.4514	0.0196	0.6713	0	5	0	0	2	0	0	0	0	0
**Zfp853**	-3.3998	0.039	6.219	0.0126	0.5792	4	0	0	0	2	0	0	0	0	0
**LOC108167**	-3.3996	-0.8892	4.8829	0.0271	0.7422	0	0	1	3	2	0	0	0	0	0
**Plppr4**	-3.3981	2.4114	9.0047	0.0027	0.3197	7	0	0	0	0	0	0	0	0	0
**Krt79**	-3.3978	-0.3757	7.1725	0.0074	0.4825	4	2	0	1	0	0	0	0	0	0
**Jchain**	-3.3906	-0.3887	5.2137	0.0224	0.6995	0	6	0	2	0	0	0	0	0	0
**Adam23**	-3.3885	6.7786	9.1049	0.0025	0.3165	0	1	3	0	3	0	0	0	0	0
**Nrcam**	-3.3869	6.3202	9.0837	0.0026	0.3165	4	1	2	0	0	0	0	0	0	0
**Chst8**	-3.3838	3.9813	9.0212	0.0027	0.3197	0	0	6	0	1	0	0	0	0	0
**Gm31776**	-3.3829	-0.5616	4.7697	0.029	0.7557	0	0	2	5	0	0	0	0	0	0
**Tusc5**	-3.3821	7.8203	9.0459	0.0026	0.3188	0	0	7	0	0	0	0	0	0	0
**Frmd8os**	-3.3763	-0.3842	5.6152	0.0178	0.6444	0	5	2	1	0	0	0	0	0	0
**Gm34591**	-3.3755	0.7937	8.3085	0.0039	0.3756	1	3	0	3	0	0	0	0	0	0
**Pamr1**	-3.3663	0.2442	6.9163	0.0085	0.5048	4	3	0	0	0	0	0	0	0	0
**Gm31613**	-3.3641	-0.7715	4.2089	0.0402	0.8291	2	0	0	0	3	0	0	0	0	0
**LOC105246**	-3.3641	0.4784	12.674	0.0004	0.1235	6	1	17	15	2	1	0	1	0	1
**1700092M**	-3.3621	-0.726	4.1069	0.0427	0.8447	0	2	0	5	0	0	0	0	0	0
**Cd83**	-3.3494	0.6412	8.084	0.0045	0.3937	2	3	2	0	0	0	0	0	0	0
**4930511M**	-3.348	-0.1655	6.1698	0.013	0.5858	0	2	1	4	0	0	0	0	0	0
**Carmn**	-3.3477	-0.1042	4.4502	0.0349	0.8042	0	0	8	0	0	0	0	0	0	0
**Gm7967**	-3.3393	0.4357	7.5153	0.0061	0.4459	0	4	0	3	0	0	0	0	0	0
**Ccdc146**	-3.3302	0.1235	6.7515	0.0094	0.5216	3	4	0	0	0	0	0	0	0	0
**Gm16897**	-3.3291	-0.1626	7.0467	0.0079	0.4954	0	3	3	0	1	0	0	0	0	0
**Notch4**	-3.3245	0.9188	11.007	0.0009	0.1888	3	9	0	5	1	0	0	0	0	1
**Gm32702**	-3.3244	-0.2996	5.3863	0.0203	0.6824	2	0	7	9	0	0	0	0	0	1
**Has2**	-3.3226	0.6101	8.2287	0.0041	0.3832	17	0	2	0	5	0	0	2	0	0
**LOC105242**	-3.3204	-0.643	4.0858	0.0432	0.85	0	7	0	1	0	0	0	0	0	0
**Gm31344**	-3.3095	-0.5403	5.4994	0.019	0.6651	3	0	1	1	1	0	0	0	0	0
**LOC108168**	-3.3082	-0.5606	5.7021	0.0169	0.6337	5	2	2	7	1	1	0	0	0	0
**Rapsn**	-3.3007	1.4526	11.29	0.0008	0.1801	6	5	5	2	0	1	0	0	0	0
**Gm30769**	-3.3	-0.2668	5.5905	0.0181	0.6487	2	0	5	0	0	0	0	0	0	0
**Gm33260**	-3.2972	-0.3171	4.9382	0.0263	0.736	3	4	0	0	0	0	0	0	0	0
**Dnajc5b**	-3.2949	-0.3186	5.3391	0.0209	0.6908	2	0	5	0	0	0	0	0	0	0
**Gm41689**	-3.2924	-0.2466	4.5354	0.0332	0.7874	6	0	0	0	0	0	0	0	0	0
**Mospd4**	-3.2817	-0.5301	4.6378	0.0313	0.7726	2	0	5	0	0	0	0	0	0	0
**Alox8**	-3.2813	-0.3851	6.8284	0.009	0.5138	1	4	2	0	0	0	0	0	0	0
**Rxfp1**	-3.2588	-0.3794	4.8274	0.028	0.7488	0	0	1	1	3	0	0	0	0	0
**Gm40007**	-3.2555	-0.7058	4.0992	0.0429	0.8462	0	0	0	6	0	0	0	0	0	0
**Gm10364**	-3.2552	-0.5788	5.4453	0.0196	0.6729	0	0	6	1	0	0	0	0	0	0
**Igsf1**	-3.25	0.0693	5.7276	0.0167	0.6337	0	0	0	6	0	0	0	0	0	0
**Spag17**	-3.2481	-0.5741	5.6393	0.0176	0.6392	0	1	3	0	2	0	0	0	0	0
**Gm30340**	-3.2451	-0.5268	4.4546	0.0348	0.8037	3	0	0	0	2	0	0	0	0	0
**Gm10584**	-3.2418	-0.4251	5.0935	0.024	0.7183	0	5	0	2	0	0	0	0	0	0
**Prss55**	-3.2379	-0.7613	4.6275	0.0315	0.7737	0	4	1	2	0	0	0	0	0	0
**LOC108168**	-3.2374	0.2864	12.145	0.0005	0.1486	10	3	18	5	2	0	0	0	0	3
**Krtap2-4**	-3.2263	-0.9264	3.9796	0.0461	0.8611	0	0	0	0	4	0	0	0	0	0
**LOC108168**	-3.2263	-0.9264	3.9796	0.0461	0.8611	0	0	0	0	4	0	0	0	0	0
**Npr1**	-3.218	0.6516	7.5007	0.0062	0.4459	14	0	0	1	0	0	0	1	0	0
**Prr32**	-3.2169	-0.5414	5.4565	0.0195	0.6705	0	0	1	5	0	0	0	0	0	0
**Slc25a21**	-3.2159	-0.373	5.2187	0.0223	0.6995	0	0	7	0	0	0	0	0	0	0
**Olr1**	-3.2157	-0.6918	4.0242	0.0449	0.8574	0	5	0	2	0	0	0	0	0	0
**Gm32461**	-3.2155	-0.7977	3.8908	0.0486	0.8678	0	0	6	1	0	0	0	0	0	0
**Gm39664**	-3.214	-0.4205	4.2577	0.0391	0.8215	0	0	1	5	0	0	0	0	0	0
**Scarna3b**	-3.2133	-0.741	4.0568	0.044	0.8533	0	0	1	5	0	0	0	0	0	0
**Ptprz1**	-3.2007	5.3368	14.359	0.0002	0.0808	3	1	4	7	1	0	0	0	0	1

**Sema3g**	-3.1996	3.6733	7.7067	0.0055	0.4317	6	0	0	0	0	0	0	0	0	0
**Gm33319**	-3.1992	-0.6078	5.3431	0.0208	0.6905	0	3	4	0	0	0	0	0	0	0
**Nanos3**	-3.1963	0.1611	6.4297	0.0112	0.5523	2	0	2	2	0	0	0	0	0	0
**Gm38822**	-3.1954	-0.639	5.6495	0.0175	0.6381	0	3	0	2	1	0	0	0	0	0
**A530020G2**	-3.1935	-0.8316	3.8533	0.0496	0.8726	0	1	0	2	2	0	0	0	0	0
**Calb2**	-3.1925	4.7797	7.7208	0.0055	0.4316	5	1	0	0	0	0	0	0	0	0
**Prss12**	-3.1889	4.8445	7.759	0.0053	0.4269	4	0	2	0	0	0	0	0	0	0
**Rims4**	-3.1883	3.411	7.7096	0.0055	0.4317	4	2	0	0	0	0	0	0	0	0
**Cmtm5**	-3.1877	5.565	7.7739	0.0053	0.4266	4	2	0	0	0	0	0	0	0	0
**Cadps**	-3.1862	6.9073	7.7824	0.0053	0.4256	3	3	0	0	0	0	0	0	0	0
**Gm40279**	-3.1805	-0.6723	4.3077	0.0379	0.8131	4	2	0	0	0	0	0	0	0	0
**Gm32605**	-3.1759	0.4925	6.2147	0.0127	0.5799	0	2	0	4	0	0	0	0	0	0
**Gm36904**	-3.1707	-0.4394	4.0465	0.0443	0.8549	0	0	2	4	0	0	0	0	0	0
**Galnt16**	-3.1695	3.4402	7.6105	0.0058	0.4373	0	6	0	0	0	0	0	0	0	0
**Syt6**	-3.1644	4.4738	13.66	0.0002	0.095	0	15	0	0	1	0	0	0	0	1
**Gm30034**	-3.1606	-0.7437	4.7985	0.0285	0.7527	0	3	1	1	1	0	0	0	0	0
**Nr1h5**	-3.1567	-0.8924	6.8906	0.0087	0.5068	1	1	3	1	0	0	0	0	0	0
**Gm35655**	-3.1553	-0.1568	8.4675	0.0036	0.3649	3	8	3	11	1	1	0	1	0	0
**Gm13986**	-3.1516	-0.2687	5.0316	0.0249	0.7219	8	4	2	4	0	0	0	0	1	0
**Gm29797**	-3.1514	-0.1332	5.439	0.0197	0.6745	0	0	5	0	1	0	0	0	0	0
**Ube2d2b**	-3.1443	-0.2908	6.797	0.0091	0.5176	3	9	2	0	2	0	0	1	0	0
**Hist1h2af**	-3.1434	-0.7251	7.0269	0.008	0.4989	1	3	2	0	0	0	0	0	0	0
**Gm33269**	-3.143	0.0239	6.1172	0.0134	0.5946	2	0	4	0	0	0	0	0	0	0
**Gm6559**	-3.1405	-0.9087	4.3924	0.0361	0.811	3	0	0	1	1	0	0	0	0	0
**Gm32650**	-3.1376	-0.6572	4.6755	0.0306	0.7712	0	0	3	3	0	0	0	0	0	0
**Gypa**	-3.1363	-0.1076	6.5224	0.0107	0.546	1	3	1	1	0	0	0	0	0	0
**Col19a1**	-3.1353	0.8483	6.5513	0.0105	0.5454	2	4	0	0	0	0	0	0	0	0
**Gm30339**	-3.1329	-0.1851	8.3489	0.0039	0.3742	2	1	9	5	0	0	0	0	1	0
**Edn3**	-3.1231	1.4256	13.495	0.0002	0.1024	11	11	0	0	3	0	0	0	1	1
**Rgcc**	-3.1201	4.2994	13.326	0.0003	0.107	9	0	0	5	1	0	0	0	0	1
**Gm32688**	-3.1158	-0.4975	4.9108	0.0267	0.7377	2	0	4	0	0	0	0	0	0	0
**Gm40768**	-3.1059	-0.4369	4.343	0.0372	0.8131	0	3	0	3	0	0	0	0	0	0
**Nr4a3**	-3.1051	-0.198	5.2748	0.0216	0.6958	4	0	0	0	1	0	0	0	0	0
**Gm32113**	-3.104	0.3539	6.3626	0.0117	0.5582	0	0	6	0	0	0	0	0	0	0
**C920021L1**	-3.0942	-0.0736	4.4427	0.0351	0.8062	0	0	4	2	0	0	0	0	0	0
**Gm39671**	-3.0905	-0.8162	4.272	0.0387	0.8196	0	0	4	2	0	0	0	0	0	0
**LOC10816**	-3.0903	0.1731	8.6208	0.0033	0.3486	1	4	7	4	0	1	0	0	0	0
**Gm36696**	-3.0813	-0.6909	4.9148	0.0266	0.7377	0	3	1	2	0	0	0	0	0	0
**LOC10816**	-3.0804	-0.3452	4.7855	0.0287	0.7541	2	4	0	0	0	0	0	0	0	0
**Ecm2**	-3.0798	2.6013	16.896	4E-05	0.0438	22	6	1	3	1	0	1	0	2	0
**Plcxd1**	-3.0734	-0.455	6.3669	0.0116	0.5577	3	5	4	1	2	1	0	0	0	0
**Far2**	-3.0602	2.023	10.965	0.0009	0.192	5	0	3	4	2	0	0	1	0	0
**Chrm3**	-3.0571	1.8977	12.827	0.0003	0.1168	22	0	5	0	3	0	0	0	0	3
**Spred3**	-3.0547	1.9448	10.426	0.0012	0.2316	3	9	0	3	0	0	0	0	0	1
**4930578C1**	-3.0458	-0.1522	4.8356	0.0279	0.7488	5	0	0	0	0	0	0	0	0	0
**Gm29891**	-3.0431	-0.8698	3.9389	0.0472	0.8678	3	0	0	2	0	0	0	0	0	0
**Gm10109**	-3.0419	0.0395	6.0821	0.0137	0.6008	2	0	1	0	2	0	0	0	0	0
**Ptafr**	-3.0405	0.3101	5.7591	0.0164	0.6285	0	6	0	0	0	0	0	0	0	0
**Fam83e**	-3.0368	-0.3394	6.0357	0.014	0.604	14	6	4	9	0	2	0	0	0	1
**2010320O**	-3.0356	-0.498	4.0093	0.0452	0.8577	2	0	0	3	0	0	0	0	0	0
**Onecut3**	-3.0219	1.2284	5.6568	0.0174	0.6381	5	0	0	0	0	0	0	0	0	0
**4930557K0**	-3.017	-0.5944	3.921	0.0477	0.8678	0	9	0	5	1	0	0	0	0	1
**Csrnp3**	-3.0124	5.1642	12.1	0.0005	0.1491	2	0	6	6	0	0	0	0	0	1
**Zdhhc22**	-3.0109	4.505	11.89	0.0006	0.1538	7	4	0	3	0	0	0	0	1	0
**B3galnt1**	-3.0094	3.8125	11.244	0.0008	0.1801	7	0	7	0	0	0	0	1	0	0
**Cyp4b1**	-3.009	-0.2817	4.0827	0.0433	0.85	4	0	0	0	6	0	0	0	0	1
**Gm40117**	-3.0042	-0.5319	4.1095	0.0426	0.8447	0	0	6	0	0	0	0	0	0	0
**Fhad1os2**	-3.0032	-0.7088	4.013	0.0451	0.8577	1	5	0	0	0	0	0	0	0	0
**Acr**	-3.0015	0.2387	5.6666	0.0173	0.6381	0	0	0	5	0	0	0	0	0	0
**Gm39988**	-3	-0.2896	3.9956	0.0456	0.8597	0	0	6	0	0	0	0	0	0	0
**Zfp42**	-2.9977	-0.6743	4.3843	0.0363	0.811	0	2	4	0	0	0	0	0	0	0
**2810407A1**	-2.9966	-0.7765	4.1883	0.0407	0.833	3	0	1	1	0	0	0	0	0	0

**Nlrc5**	-2.9964	0.0031	3.8946	0.0484	0.8678	0	0	6	0	0	0	0	0	0	0
**Gm30927**	-2.9951	-0.6564	4.106	0.0427	0.8447	0	1	5	0	0	0	0	0	0	0
**Dnah17**	-2.993	-0.324	4.4575	0.0347	0.8034	4	1	0	0	0	0	0	0	0	0
**Dynlrb2**	-2.9926	-0.3406	4.591	0.0321	0.7798	4	1	0	0	0	0	0	0	0	0
**BC048609**	-2.9925	-0.1411	6.4152	0.0113	0.5538	6	6	1	0	1	0	0	0	0	1
**Txlnb**	-2.9919	0.0572	5.5373	0.0186	0.6585	2	2	0	0	1	0	0	0	0	0
**Gm5486**	-2.9885	0.1135	5.6935	0.017	0.6337	4	0	1	0	0	0	0	0	0	0
**4632427E1**	-2.9865	-0.5432	4.6447	0.0311	0.7726	3	1	0	1	0	0	0	0	0	0
**Gm40434**	-2.9793	-0.8359	4.1545	0.0415	0.8377	0	3	3	0	0	0	0	0	0	0
**Hoxd9**	-2.9779	-0.1373	4.6092	0.0318	0.7776	0	0	1	4	0	0	0	0	0	0
**Gm35321**	-2.9775	0.5117	5.9132	0.015	0.6123	4	1	0	0	0	0	0	0	0	0
**Gm33729**	-2.9742	2.1802	19.055	1E-05	0.0254	4	12	17	9	0	0	2	0	1	1
**B230344G1**	-2.9732	-0.5603	4.5606	0.0327	0.7852	0	6	0	0	0	0	0	0	0	0
**Gm36044**	-2.9672	-0.8509	4.2602	0.039	0.8215	0	1	0	4	0	0	0	0	0	0
**Gnat1**	-2.9666	1.1075	6.6622	0.0098	0.5336	145	11	10	0	3	4	0	2	4	8
**Prokr1**	-2.9646	4.1685	6.4513	0.0111	0.5501	5	0	0	0	0	0	0	0	0	0
**Hspa12b**	-2.9607	0.68	5.8392	0.0157	0.6238	3	0	2	0	0	0	0	0	0	0
**Kcnq2**	-2.9606	4.9614	6.4847	0.0109	0.5486	5	0	0	0	0	0	0	0	0	0
**Trpm2**	-2.9598	3.5699	6.4262	0.0112	0.5527	1	0	1	3	0	0	0	0	0	0
**4933424G(**	-2.9578	-0.1214	6.9155	0.0085	0.5048	1	5	4	0	3	0	1	0	0	0
**A230070E(**	-2.9578	3.2827	6.3535	0.0117	0.5582	3	0	2	0	0	0	0	0	0	0
**Zmat4**	-2.9541	3.3861	6.4494	0.0111	0.5501	0	0	4	0	1	0	0	0	0	0
**Gpr37**	-2.9533	3.0695	6.4364	0.0112	0.5522	1	0	3	1	0	0	0	0	0	0
**Sorcs1**	-2.9522	4.9391	6.4768	0.0109	0.5486	1	3	1	0	0	0	0	0	0	0
**Gm42278**	-2.9504	-0.3771	4.929	0.0264	0.7377	1	0	6	4	3	0	0	0	1	0
**Tmem88b**	-2.9472	3.5431	6.3776	0.0116	0.5577	1	1	3	0	0	0	0	0	0	0
**Selplg**	-2.9422	0.4912	5.7462	0.0165	0.6311	0	2	0	3	0	0	0	0	0	0
**LOC10263**3	-2.9417	1.7732	6.2475	0.0124	0.5751	1	0	4	0	0	0	0	0	0	0
**Gm3764**	-2.9416	0.5053	5.2854	0.0215	0.6958	2	2	0	1	0	0	0	0	0	0
**Gm39342**	-2.9322	-0.2327	5.2148	0.0224	0.6995	0	6	0	5	2	0	0	0	0	1
**Gm31984**	-2.9313	-0.3295	5.6347	0.0176	0.6392	0	8	4	0	3	0	0	0	1	0
**Abhd16b**	-2.9307	-0.5186	4.5051	0.0338	0.7954	7	2	10	0	4	0	0	1	1	0
**Grid2**	-2.9305	-0.1181	4.8333	0.0279	0.7488	1	2	0	2	0	0	0	0	0	0
**LOC10816**8	-2.9273	0.6669	7.3497	0.0067	0.4632	0	5	7	0	2	1	0	0	0	0
**4930444A1**	-2.925	-0.672	4.9625	0.0259	0.7335	0	2	2	0	1	0	0	0	0	0
**Appbp2os**	-2.9249	-0.4217	4.9731	0.0257	0.7331	3	5	0	4	1	0	0	1	0	0
**Dok7**	-2.919	2.602	6.1407	0.0132	0.5897	0	5	0	0	0	0	0	0	0	0
**Il25**	-2.9167	0.8531	5.9812	0.0145	0.6056	0	0	5	0	0	0	0	0	0	0
**Gm10406**	-2.9109	-0.6557	4.0822	0.0433	0.85	0	3	1	0	1	0	0	0	0	0
**Zfp641**	-2.9076	1.8152	5.928	0.0149	0.6085	0	2	3	0	0	0	0	0	0	0
**Hbb-bt**	-2.9062	7.3405	18.2	2E-05	0.0298	145	27	2	4	16	14	0	5	2	3
**Apba1**	-2.9014	7.012	16.411	5E-05	0.0459	9	0	10	1	1	0	1	0	0	1
**Pax1**	-2.9012	-0.6404	4.0863	0.0432	0.85	2	0	3	0	0	0	0	0	0	0
**Gm35164**	-2.898	-0.852	3.9236	0.0476	0.8678	2	0	3	0	0	0	0	0	0	0
**Gm14304**	-2.8964	-0.3671	9.1931	0.0024	0.3137	2	3	5	0	3	0	0	0	1	0
**Snord35a**	-2.8926	-0.8517	4.1292	0.0421	0.8429	2	1	2	0	0	0	0	0	0	0
**Lor**	-2.8826	-0.1913	4.11	0.0426	0.8447	2	2	1	0	0	0	0	0	0	0
**1700028E1**	-2.8708	0.022	4.7799	0.0288	0.7541	0	5	0	9	9	0	0	0	0	3
**Gm9801**	-2.8677	0.7514	5.2163	0.0224	0.6995	0	0	5	0	0	0	0	0	0	0
**Ccl5**	-2.8588	0.1474	5.4378	0.0197	0.6745	0	5	0	0	0	0	0	0	0	0
**Gm31152**	-2.8548	-0.4401	4.7047	0.0301	0.7694	0	2	2	1	0	0	0	0	0	0
**Tpsab1**	-2.8525	-0.0039	4.7194	0.0298	0.7675	0	0	0	2	2	0	0	0	0	0
**Adamts4**	-2.8517	1.3899	5.3483	0.0207	0.6897	0	5	0	0	0	0	0	0	0	0
**Myoc**	-2.8423	2.7204	10.622	0.0011	0.2185	70	3	1	0	5	7	0	2	1	0
**Hrh1**	-2.8423	0.429	4.7794	0.0288	0.7541	0	0	5	0	0	0	0	0	0	0
**Bpifb1**	-2.8265	0.4573	4.8805	0.0272	0.7422	2	0	0	0	2	0	0	0	0	0
**Gm31954**	-2.8263	-0.3839	5.7174	0.0168	0.6337	0	1	5	14	5	1	0	0	0	2
**4930538K1**	-2.8207	-0.0698	4.3801	0.0364	0.811	0	0	5	0	0	0	0	0	0	0
**Gm34414**	-2.8173	0.9546	9.4547	0.0021	0.2865	2	0	4	3	3	0	0	0	1	0
**Gm26536**	-2.8139	-0.3068	4.7594	0.0291	0.7563	0	4	1	0	0	0	0	0	0	0
**Rgs22**	-2.813	-0.2242	4.2298	0.0397	0.8236	0	0	5	0	0	0	0	0	0	0
**Gm40706**	-2.8123	-0.4664	4.1901	0.0407	0.8329	0	1	1	0	2	0	0	0	0	0

**Pcdhb10**	-2.8076	-0.0939	4.6656	0.0308	0.772	0	4	1	0	0	0	0	0	0	0
**Casq2**	-2.8074	1.0297	5.1895	0.0227	0.7048	0	0	0	0	4	0	0	0	0	0
**Ppp2r2c**	-2.8068	8.1678	19.605	1E-05	0.0206	11	7	1	1	7	0	0	0	0	3
**Lin7a**	-2.804	5.9002	9.9202	0.0016	0.2581	6	4	0	1	1	1	0	0	0	0
**Gm35455**	-2.8036	-0.7971	4.3623	0.0367	0.8127	0	2	3	0	0	0	0	0	0	0
**LOC108167**	-2.7995	0.9057	16.679	4E-05	0.0442	14	13	7	6	4	1	0	1	2	1
**Abcg4**	-2.7978	4.0536	9.7841	0.0018	0.2615	7	4	1	0	0	0	0	0	1	0
**Ccdc36**	-2.7936	-0.6364	4.1305	0.0421	0.8427	2	0	1	0	1	0	0	0	0	0
**Gm32807**	-2.7907	-0.0473	4.5216	0.0335	0.7916	0	5	0	0	0	0	0	0	0	0
**Mir3109**	-2.7895	-0.5568	5.4651	0.0194	0.6697	3	0	3	3	2	0	0	0	0	1
**Gm30970**	-2.7893	-0.0246	4.6635	0.0308	0.772	0	4	5	0	3	1	0	0	0	0
**Gm31092**	-2.7892	-0.8354	4.3849	0.0363	0.811	0	4	1	0	0	0	0	0	0	0
**Gpr182**	-2.7832	0.2101	4.6359	0.0313	0.7726	0	0	0	3	1	0	0	0	0	0
**Itga5**	-2.7813	-0.4716	4.12	0.0424	0.8429	0	3	2	0	0	0	0	0	0	0
**Tmprss12**	-2.7811	-0.5791	4.3286	0.0375	0.8131	2	8	0	4	3	0	2	0	0	0
**Il21r**	-2.7763	0.105	4.8042	0.0284	0.7519	3	0	0	0	1	0	0	0	0	0
**Tbx1**	-2.7733	0.2473	5.9551	0.0147	0.6067	6	0	3	0	2	1	0	0	0	0
**Pcdhga5**	-2.7671	0.2972	4.7633	0.0291	0.7563	0	0	0	3	1	0	0	0	0	0
**Sytl5**	-2.767	-0.2515	6.0366	0.014	0.604	2	5	0	5	0	0	0	1	0	0
**Gm5103**	-2.7578	-0.2332	7.6447	0.0057	0.434	4	13	4	6	1	0	0	2	0	1
**Nrk**	-2.7573	-0.1366	4.231	0.0397	0.8235	4	0	0	0	0	0	0	0	0	0
**Als2cr11b**	-2.7425	-0.5732	4.3107	0.0379	0.8131	2	0	0	2	0	0	0	0	0	0
**2410124H1**	-2.7423	-0.8894	4.0438	0.0443	0.8557	1	0	2	0	1	0	0	0	0	0
**Hbb-bs**	-2.7396	9.4766	15.158	1E-04	0.0631	790	71	15	7	51	74	14	31	8	10
**Gm41223**	-2.7379	-0.7386	3.9502	0.0469	0.8678	0	0	0	4	0	0	0	0	0	0
**Lepr**	-2.7372	0.8905	4.6557	0.031	0.7726	4	0	0	0	0	0	0	0	0	0
**Kcnj8**	-2.7358	0.6004	4.6774	0.0306	0.7712	4	0	0	0	0	0	0	0	0	0
**Rab20**	-2.7355	1.0617	10.335	0.0013	0.2316	9	5	2	7	2	0	0	1	0	2
**Itgb1bp2**	-2.7353	-0.7388	4.7363	0.0295	0.7616	0	0	1	2	1	0	0	0	0	0
**Prdm6**	-2.7352	0.3531	4.3659	0.0367	0.8126	1	0	0	3	0	0	0	0	0	0
**Them5**	-2.7348	2.6715	8.3863	0.0038	0.3726	6	0	2	0	3	0	0	0	0	1
**Gm1821**	-2.7291	0.3986	8.4478	0.0037	0.3673	12	2	0	3	5	0	1	0	0	2
**Gpr3**	-2.7289	1.6722	5.2145	0.0224	0.6995	0	0	0	0	4	0	0	0	0	0
**Plekha4**	-2.7262	6.4798	22.513	2E-06	0.0158	7	12	3	9	2	0	0	0	1	3
**P2ry13**	-2.7229	0.384	4.8509	0.0276	0.7469	4	0	0	0	0	0	0	0	0	0
**Gm39643**	-2.7202	-0.3501	6.2427	0.0125	0.5756	7	5	10	2	2	0	0	3	0	0
**Ifnk**	-2.7184	-0.4197	3.8522	0.0497	0.8726	0	0	3	1	5	0	0	0	0	1
**B230311B0**	-2.7169	1.3594	5.149	0.0233	0.7084	0	0	0	2	2	0	0	0	0	0
**Ttll9**	-2.7161	0.7922	4.7381	0.0295	0.7616	0	0	0	4	0	0	0	0	0	0
**LOC108168**	-2.7157	0.019	4.3785	0.0364	0.8112	7	1	1	13	0	0	0	0	2	0
**B4galnt3**	-2.7149	0.862	4.7613	0.0291	0.7563	0	0	0	4	0	0	0	0	0	0
**AI115009**	-2.7144	0.7102	4.9446	0.0262	0.735	4	0	0	0	0	0	0	0	0	0
**Sez6l**	-2.7125	1.5115	5.1663	0.023	0.7063	2	0	0	0	2	0	0	0	0	0
**Sfrp2**	-2.7117	1.0853	7.518	0.0061	0.4459	23	0	1	3	2	0	0	4	0	0
**Gm41313**	-2.7098	-0.3367	4.2138	0.0401	0.8286	3	0	1	0	0	0	0	0	0	0
**Gjd2**	-2.7016	1.63	5.0618	0.0245	0.7207	4	0	0	0	0	0	0	0	0	0
**Abca6**	-2.6993	3.8468	5.2185	0.0223	0.6995	0	0	0	0	4	0	0	0	0	0
**Lrrtm4**	-2.6948	2.2324	5.1717	0.023	0.7051	3	0	0	0	1	0	0	0	0	0
**Angptl6**	-2.6939	-0.6531	4.7269	0.0297	0.7647	1	0	1	2	0	0	0	0	0	0
**Trpv1**	-2.6939	3.8862	5.1746	0.0229	0.7051	3	0	0	0	1	0	0	0	0	0
**Hus1b**	-2.6888	-0.8317	3.9747	0.0462	0.8611	1	1	0	2	0	0	0	0	0	0
**Poln**	-2.6875	-0.1448	4.5606	0.0327	0.7852	0	0	11	0	1	0	0	0	0	1
**Gm35715**	-2.6866	0.0557	6.2671	0.0123	0.5747	10	7	2	12	1	0	2	0	1	1
**Haghl**	-2.686	0.1492	4.3309	0.0374	0.8131	0	0	3	0	1	0	0	0	0	0
**Hecw1**	-2.6841	6.8525	5.2165	0.0224	0.6995	0	0	0	1	3	0	0	0	0	0
**Opn3**	-2.6826	2.8218	5.1229	0.0236	0.712	2	1	0	1	0	0	0	0	0	0
**Cacng2**	-2.6821	5.4409	5.1743	0.0229	0.7051	0	0	2	1	1	0	0	0	0	0
**Gm33185**	-2.6812	-0.6606	3.9304	0.0474	0.8678	0	1	2	0	1	0	0	0	0	0
**Scn1a**	-2.6807	6.6996	5.2116	0.0224	0.6995	4	0	0	0	0	0	0	0	0	0
**Scn9a**	-2.6793	7.7029	5.2039	0.0225	0.702	0	0	3	0	1	0	0	0	0	0
**Rab33a**	-2.6779	3.0773	5.1413	0.0234	0.7084	0	0	2	2	0	0	0	0	0	0
**Hapln1**	-2.677	3.7686	5.1589	0.0231	0.7074	0	3	0	0	1	0	0	0	0	0

**Saxo2**	-2.6762	1.6685	9.2498	0.0024	0.3127	1	0	7	10	0	0	0	0	0	2
**Cntn4**	-2.6719	3.5493	5.1398	0.0234	0.7084	0	0	4	0	0	0	0	0	0	0
**Rem2**	-2.6683	3.9779	5.1288	0.0235	0.7108	0	4	0	0	0	0	0	0	0	0
**Adgrb1**	-2.6675	3.8524	8.4842	0.0036	0.3649	0	8	0	3	0	0	0	0	0	1
**Lingo4**	-2.6669	2.8181	5.1116	0.0238	0.7149	0	3	1	0	0	0	0	0	0	0
**Gm35362**	-2.6634	2.1052	5.0459	0.0247	0.7214	0	0	4	0	0	0	0	0	0	0
**Clec10a**	-2.6628	0.3687	4.548	0.033	0.7852	0	3	0	0	1	0	0	0	0	0
**Gm35721**	-2.6569	1.2937	4.9729	0.0257	0.7331	0	0	4	0	0	0	0	0	0	0
**Gm5432**	-2.6508	-0.2718	3.9267	0.0475	0.8678	3	1	8	0	0	1	0	0	0	0
**Tnr**	-2.6506	2.9251	11.347	0.0008	0.1795	2	4	3	2	6	0	1	1	0	0
**Gm31734**	-2.6474	0.2464	5.8421	0.0156	0.6238	9	16	1	7	0	2	1	1	0	0
**Gm31436**	-2.646	-0.6883	4.1203	0.0424	0.8429	0	1	1	2	0	0	0	0	0	0
**Gm33656**	-2.6383	-0.0071	7.4897	0.0062	0.4459	3	10	4	3	3	0	3	0	0	0
**LOC10263**	-2.632	-0.2262	4.5219	0.0335	0.7916	1	2	1	0	0	0	0	0	0	0
**Tub**	-2.6305	0.1568	4.3928	0.0361	0.811	1	1	2	0	0	0	0	0	0	0
**Plch1**	-2.6281	0.2181	4.6332	0.0314	0.7727	0	0	4	0	0	0	0	0	0	0
**Gm32709**	-2.6147	0.3145	7.5678	0.0059	0.4426	1	16	8	1	1	0	0	1	0	2
**Slc7a7**	-2.6145	0.4266	4.6522	0.031	0.7726	0	4	0	0	0	0	0	0	0	0
**Gm39256**	-2.607	1.0424	4.4917	0.0341	0.7976	0	2	2	0	0	0	0	0	0	0
**Sncg**	-2.6046	9.5047	19.582	1E-05	0.0206	12	8	3	0	7	1	0	0	1	2
**Ddc**	-2.5995	1.2144	8.7877	0.003	0.3378	5	2	4	6	0	1	0	0	0	1
**Slc25a47**	-2.5986	0.9249	12.971	0.0003	0.1116	12	28	5	11	5	5	0	0	1	2
**9530026P0**	-2.5948	0.436	4.462	0.0347	0.8027	0	4	0	0	0	0	0	0	0	0
**Zfp804b**	-2.5803	1.3261	8.8619	0.0029	0.3294	8	3	6	0	0	0	0	1	0	1
**Ptprb**	-2.5753	3.6781	18.263	2E-05	0.0298	31	1	4	5	5	1	0	0	1	5
**A830052D1**	-2.574	0.1893	4.2515	0.0392	0.8224	0	4	0	0	0	0	0	0	0	0
**Obscn**	-2.5737	0.1286	3.9103	0.048	0.8678	0	0	4	0	0	0	0	0	0	0
**Ccdc65**	-2.5657	0.8938	5.9312	0.0149	0.6085	2	1	4	0	3	0	0	0	1	0
**Acsl6**	-2.5614	5.841	18.723	2E-05	0.0264	10	0	8	6	5	0	0	3	0	1
**Syt5**	-2.561	4.3353	7.5885	0.0059	0.4403	6	3	0	0	1	1	0	0	0	0
**Esr2**	-2.5588	-0.1124	6.9163	0.0085	0.5048	4	9	0	8	2	2	0	1	0	0
**Vtn**	-2.5563	3.6645	7.3656	0.0066	0.4617	3	3	0	4	0	1	0	0	0	0
**Gpr179**	-2.5417	1.2001	7.504	0.0062	0.4459	6	2	0	7	1	0	0	0	1	1
**1700024P1**	-2.5383	0.1656	7.6726	0.0056	0.434	6	5	6	0	0	0	0	0	0	2
**Rtbdn**	-2.5333	0.8554	5.761	0.0164	0.6285	7	2	0	1	0	0	0	0	1	0
**Gfra2**	-2.5295	5.8986	14.444	0.0001	0.0808	15	0	1	1	5	2	0	0	0	1
**LOC10263**	-2.5246	1.18	5.7798	0.0162	0.6285	4	0	6	0	0	0	0	0	0	1
**Mpeg1**	-2.5177	1.5344	9.0781	0.0026	0.3165	15	1	2	2	1	0	0	0	0	3
**Slamf9**	-2.5139	0.9004	5.3767	0.0204	0.6838	1	0	6	3	0	0	0	1	0	0
**LOC10524**	-2.5128	0.4265	9.1272	0.0025	0.3165	3	6	8	5	2	0	0	0	2	1
**LOC10524**	-2.5124	-0.1269	4.0729	0.0436	0.85	9	1	1	3	8	0	0	3	0	1
**Gm16062**	-2.5084	0.196	4.5935	0.0321	0.7798	5	4	7	0	0	0	2	0	0	0
**Gpr34**	-2.5082	0.8544	8.6873	0.0032	0.3414	0	3	9	1	3	0	0	2	0	0
**Dlk1**	-2.5021	0.9746	4.3745	0.0365	0.8112	1	0	7	8	4	0	0	0	0	3
**Spdya**	-2.4952	-0.072	5.0338	0.0249	0.7219	3	2	4	8	1	0	0	0	2	0
**Gm34064**	-2.4909	-0.05	4.6846	0.0304	0.7706	3	2	5	6	0	0	0	2	0	0
**Crybb3**	-2.4862	1.5583	10.327	0.0013	0.2316	93	75	7	6	4	14	1	4	1	8
**Fam47e**	-2.4618	-0.3395	4.0077	0.0453	0.8577	3	2	5	0	9	1	0	0	1	1
**C130074G1**	-2.4434	3.3473	6.3494	0.0117	0.5588	8	0	0	1	0	0	0	0	0	1
**Gata3**	-2.4372	0.8516	5.6927	0.017	0.6337	4	1	6	2	6	3	0	0	0	0
**4930429B2**	-2.4204	0.7219	5.952	0.0147	0.6067	0	5	0	9	1	2	0	0	0	0
**Moxd1**	-2.4173	3.0246	6.3049	0.012	0.5662	6	3	0	0	0	0	0	0	0	1
**Kcnrg**	-2.4151	0.1841	5.528	0.0187	0.6593	9	5	5	1	1	1	0	1	0	1
**Adgrl4**	-2.4143	2.5996	6.372	0.0116	0.5577	3	3	1	0	2	0	0	0	1	0
**Samd15**	-2.41	0.8184	7.5728	0.0059	0.4423	3	4	3	5	0	0	0	0	0	2
**Padi2**	-2.4077	4.1599	36.209	2E-09	6E-05	26	29	23	4	12	2	7	0	0	7
**2610020C0**	-2.4006	-0.1154	5.466	0.0194	0.6697	7	4	3	1	5	2	0	0	1	0
**Enpp2**	-2.3979	5.6482	16.604	5E-05	0.0448	46	1	3	7	8	0	0	4	1	7
**Gm40136**	-2.386	-0.2452	8.2845	0.004	0.3796	4	6	7	3	1	1	0	0	0	2
**Nr4a1**	-2.3825	3.3685	8.3678	0.0038	0.3742	2	0	0	11	1	0	0	2	0	0
**Neurod1**	-2.374	1.3472	6.888	0.0087	0.5068	9	0	0	0	4	2	0	0	0	0
**Gpr173**	-2.3673	2.3342	10.129	0.0015	0.2468	0	2	4	7	6	0	2	0	1	0

**Gm40268**	-2.3668	-0.3927	3.9385	0.0472	0.8678	0	0	5	1	6	1	0	1	0	0
**Mettl7a3**	-2.3663	1.0169	3.859	0.0495	0.8726	2	0	0	0	1	0	0	0	0	0
**Cited1**	-2.3576	1.6734	5.9281	0.0149	0.6085	0	5	4	0	0	0	0	0	1	0
**Nell2**	-2.3571	2.8092	3.9015	0.0482	0.8678	1	0	0	0	2	0	0	0	0	0
**2610307P1**	-2.3501	1.2708	3.8517	0.0497	0.8726	3	0	0	0	0	0	0	0	0	0
**Scrt2**	-2.3435	3.7137	3.8862	0.0487	0.8683	3	0	0	0	0	0	0	0	0	0
**Apba2**	-2.3428	4.3794	3.8896	0.0486	0.8678	3	0	0	0	0	0	0	0	0	0
**Brinp1**	-2.3398	5.003	3.9041	0.0482	0.8678	3	0	0	0	0	0	0	0	0	0
**Akap6**	-2.3397	7.5926	9.0092	0.0027	0.3197	6	0	7	0	1	0	0	0	0	2
**Paqr9**	-2.3395	3.9833	3.9055	0.0481	0.8678	3	0	0	0	0	0	0	0	0	0
**Igsf21**	-2.3393	4.6314	3.9065	0.0481	0.8678	3	0	0	0	0	0	0	0	0	0
**Ralyl**	-2.3391	5.3312	3.9071	0.0481	0.8678	3	0	0	0	0	0	0	0	0	0
**Pnma2**	-2.339	5.606	3.9064	0.0481	0.8678	1	1	0	0	1	0	0	0	0	0
**Ptprn**	-2.3387	6.4263	3.895	0.0484	0.8678	0	0	2	0	1	0	0	0	0	0
**Gabra4**	-2.3386	1.8795	3.8542	0.0496	0.8726	2	1	0	0	0	0	0	0	0	0
**2900011O(**	-2.3342	5.1622	3.8931	0.0485	0.8678	0	2	1	0	0	0	0	0	0	0
**Cacng5**	-2.334	4.331	3.8897	0.0486	0.8678	0	1	2	0	0	0	0	0	0	0
**Rspo2**	-2.3324	4.3269	3.8738	0.049	0.8705	0	0	3	0	0	0	0	0	0	0
**Lama4**	-2.329	4.8289	11.328	0.0008	0.1795	13	1	0	1	4	2	0	1	0	0
**Acap1**	-2.324	-0.3855	4.9722	0.0258	0.7331	3	4	6	2	4	3	0	0	0	0
**1700012D1**	-2.3229	0.0534	7.0927	0.0077	0.4918	6	8	12	3	5	2	3	0	0	1
**Cntd1**	-2.3212	-0.0179	4.2978	0.0382	0.8143	0	4	7	0	3	0	0	0	0	2
**Gm31909**	-2.3193	-0.1197	4.0961	0.043	0.8473	0	18	5	5	4	2	0	3	0	0
**Gm40996**	-2.3192	0.2002	5.5253	0.0187	0.6593	0	13	1	5	7	0	0	2	2	0
**Tnfsf15**	-2.3178	0.1546	4.5593	0.0327	0.7852	3	0	7	2	3	0	0	0	2	0
**Igfbp6**	-2.2952	4.2855	11.176	0.0008	0.1817	18	0	0	4	1	0	0	3	0	1
**3200001D2**	-2.2929	-0.1396	6.0027	0.0143	0.604	8	6	8	9	4	0	0	5	1	0
**Gm14808**	-2.2921	0.4331	5.0268	0.025	0.7219	0	13	0	1	1	0	0	1	0	1
**Cxcl12**	-2.29	3.9478	15.596	8E-05	0.055	14	3	0	10	2	0	0	1	1	3
**Larp6**	-2.2851	3.7911	5.5566	0.0184	0.6548	4	0	0	1	3	0	0	1	0	0
**Rbp7**	-2.2765	0.5259	4.5727	0.0325	0.7828	2	0	2	4	0	0	0	0	0	1
**Dnaaf3**	-2.2759	0.6598	4.2497	0.0393	0.8224	0	0	18	0	0	0	0	0	2	0
**A030001D2**	-2.2751	2.3064	22.42	2E-06	0.0158	15	21	17	8	7	1	2	1	3	5
**Gm35946**	-2.2746	0.3343	9.0165	0.0027	0.3197	11	9	8	18	10	0	6	4	0	2
**Gabrb3**	-2.2712	4.976	5.513	0.0189	0.662	8	0	0	0	0	0	0	0	0	1
**St8sia1**	-2.2631	3.9498	5.5467	0.0185	0.6576	3	2	1	1	1	0	1	0	0	0
**Tmem169**	-2.2488	2.8983	7.0637	0.0079	0.4947	4	2	3	3	1	0	0	1	0	1
**2210406O1**	-2.2409	1.3386	9.0422	0.0026	0.3188	5	14	0	6	4	0	0	1	1	3
**Chil1**	-2.2409	1.3466	5.1411	0.0234	0.7084	71	3	8	3	0	1	1	8	4	1
**Stxbp6**	-2.2381	4.4201	7.7566	0.0054	0.4269	1	3	5	0	4	0	0	1	1	0
**A230052G(**	-2.2282	0.6096	4.1856	0.0408	0.8338	1	0	4	3	0	1	0	0	0	0
**Chrnb2**	-2.2242	3.2811	7.6613	0.0056	0.434	0	5	4	4	0	0	0	0	0	2
**Gm35150**	-2.2191	0.7088	5.1807	0.0228	0.7051	2	10	0	7	0	0	0	0	0	3
**Krt20**	-2.217	-0.0525	5.0838	0.0242	0.7184	0	10	5	9	4	1	0	0	0	4
**LOC108167**	-2.2132	0.2756	5.2203	0.0223	0.6995	10	8	18	6	5	0	0	9	0	0
**Slc9a5**	-2.1937	2.5673	6.5086	0.0107	0.547	0	4	5	4	0	0	0	0	0	2
**LOC108167**	-2.1894	1.3677	7.9161	0.0049	0.4069	0	22	3	3	2	1	1	0	0	3
**Lilrb4a**	-2.179	1.1632	6.5149	0.0107	0.546	13	3	5	2	0	0	0	3	1	0
**Slco1a5**	-2.1761	0.5153	4.3383	0.0373	0.8131	8	6	0	3	2	0	0	0	2	1
**Nxph3**	-2.173	1.3555	7.2152	0.0072	0.4784	5	2	4	3	3	0	0	2	1	0
**Gm33370**	-2.1713	-0.4403	3.8415	0.05	0.8743	0	2	4	2	0	1	0	0	0	0
**Gm39795**	-2.1682	0.6406	5.7812	0.0162	0.6285	7	20	15	5	3	2	0	7	0	0
**Sh3bgr**	-2.1641	4.6034	15.007	0.0001	0.0653	13	8	2	7	2	2	0	0	3	1
**Fam222a**	-2.1635	1.8627	6.0767	0.0137	0.6008	3	1	0	8	0	0	0	2	0	0
**Inha**	-2.1619	1.5034	6.1843	0.0129	0.5845	6	1	3	1	1	0	1	0	0	1
**Amer2**	-2.1587	5.2492	9.5578	0.002	0.2785	10	2	1	0	4	0	0	1	0	2
**Rftn1**	-2.1566	1.4469	7.9474	0.0048	0.4028	8	4	3	1	1	0	0	2	0	1
**Draxin**	-2.1415	0.3382	3.8639	0.0493	0.8716	0	7	7	0	0	0	0	2	0	0
**Gjc1**	-2.1397	2.3694	6.4842	0.0109	0.5486	5	2	0	4	1	0	0	0	1	1
**Pecam1**	-2.1393	2.7688	7.185	0.0074	0.4817	21	1	0	0	5	0	0	6	0	0
**Slfn3**	-2.1351	0.1308	5.7461	0.0165	0.6311	5	8	17	0	2	1	3	2	0	0
**Slc5a5**	-2.1329	3.1812	4.3357	0.0373	0.8131	4	0	1	0	2	0	0	1	0	0

**Gm34389**	-2.1326	0.337	7.3578	0.0067	0.4629	4	15	6	4	14	0	6	1	1	2
**1700047M**	-2.1296	0.6564	3.9767	0.0461	0.8611	2	0	1	3	1	1	0	0	0	0
**Npm3-ps1**	-2.1247	-0.312	4.2746	0.0387	0.8192	8	8	6	1	0	0	0	0	0	4
**Clvs1**	-2.121	0.0438	5.2899	0.0214	0.6958	7	4	3	3	0	1	0	1	0	1
**Hba-a2**	-2.1158	6.3823	5.4637	0.0194	0.6697	121	4	0	1	15	21	2	11	0	0
**Fam155a**	-2.0923	4.7378	4.5497	0.0329	0.7852	4	0	0	1	2	0	0	0	0	1
**Lin7b**	-2.0881	2.0257	6.0616	0.0138	0.6019	3	7	0	2	0	0	0	0	1	1
**Abcd2**	-2.0877	4.2244	4.5326	0.0333	0.7882	6	0	0	0	1	0	0	0	1	0
**Vstm2l**	-2.0873	6.587	4.5527	0.0329	0.7852	0	0	0	6	1	0	0	0	0	1
**Slit1**	-2.0857	4.7805	4.4959	0.034	0.7963	5	1	1	0	0	0	0	0	0	1
**Nap1l2**	-2.0838	4.7919	4.5183	0.0335	0.7919	1	0	2	3	1	0	0	0	1	0
**Gm3435**	-2.0801	0.9206	6.2485	0.0124	0.5751	6	3	3	16	4	2	0	5	0	0
**Gm39581**	-2.0793	0.8753	10.568	0.0012	0.2214	10	9	21	6	7	0	1	6	1	3
**Gm10575**	-2.0765	1.3647	7.8129	0.0052	0.4203	8	8	1	4	0	0	0	2	0	2
**Rasgrf2**	-2.0756	5.0193	4.4341	0.0352	0.8074	3	0	4	0	0	0	0	0	1	0
**Cd80**	-2.0659	0.2439	5.2713	0.0217	0.6958	2	7	14	0	1	1	0	0	1	2
**Tm6sf2**	-2.0638	-0.0797	4.5981	0.032	0.7798	7	8	3	5	0	2	0	0	1	1
**Gpr161**	-2.0587	1.7608	7.9599	0.0048	0.4019	7	7	9	5	2	0	0	0	0	6
**Slmo1**	-2.0577	3.3869	7.9561	0.0048	0.4019	0	5	5	4	2	0	0	0	0	3
**Necab2**	-2.0574	2.2387	4.1205	0.0424	0.8429	0	0	6	1	0	0	0	1	0	0
**Nckap5l**	-2.0558	2.8469	15.669	8E-05	0.055	11	10	9	17	1	4	0	4	0	2
**Olfr856-ps**	-2.0509	0.3652	6.022	0.0141	0.604	4	4	4	6	2	0	0	0	0	4
**Rax**	-2.0501	-0.3075	4.823	0.0281	0.7493	8	6	2	6	0	2	0	0	1	1
**Gm36283**	-2.0458	1.075	9.7739	0.0018	0.2615	18	19	20	11	11	5	4	2	0	7
**Aqp4**	-2.0214	4.5439	5.9325	0.0149	0.6085	6	1	1	2	1	1	0	0	0	1
**Fabp3**	-2.02	4.8735	15.174	1E-04	0.0631	11	9	5	4	4	1	0	1	0	5
**Grb14**	-2.0169	5.3422	6.0751	0.0137	0.6008	0	0	0	7	4	0	0	0	2	0
**Pde6b**	-2.0159	0.4915	3.9525	0.0468	0.8678	48	2	2	0	1	2	3	0	2	5
**Prox1**	-2.013	1.7252	5.3239	0.021	0.6943	28	0	1	0	0	1	0	0	2	3
**Kcnh2**	-2.0105	4.785	6.0202	0.0141	0.604	6	0	5	0	0	1	0	0	0	1
**Gm34006**	-2.0068	0.7253	4.3449	0.0371	0.8131	0	0	4	7	0	0	0	0	0	2
**Pcdhga11**	-2.0062	1.8006	11.273	0.0008	0.1801	5	5	10	13	8	4	0	3	2	0
**Zfp85os**	-1.9933	0.5375	6.3169	0.012	0.5636	2	8	10	11	3	0	0	2	1	4
**Fcor**	-1.9911	1.1482	6.5987	0.0102	0.5433	1	1	5	6	2	1	0	0	0	2
**Cdk5r2**	-1.9799	7.4116	7.6589	0.0056	0.434	10	3	0	0	2	0	0	0	1	2
**2310016G1**	-1.9792	2.2348	15.963	6E-05	0.053	3	12	14	18	4	3	1	2	3	2
**LOC10816**	-1.9788	1.1106	5.6031	0.0179	0.6476	4	13	33	12	11	1	0	14	0	2
**Usp29**	-1.9627	2.5359	5.3198	0.0211	0.6946	2	7	2	0	0	0	0	0	1	1
**Ctnna2**	-1.9425	6.4747	12.463	0.0004	0.1332	14	4	2	5	2	0	2	0	0	4
**Clcn1**	-1.9416	0.437	4.2899	0.0383	0.8171	6	5	3	8	1	4	1	0	0	0
**Cd1d1**	-1.9404	0.7038	4.284	0.0385	0.8177	3	10	2	1	0	0	0	0	0	3
**Tnfrsf4**	-1.9326	0.5214	4.6338	0.0313	0.7727	5	3	18	9	8	2	3	5	0	1
**Timm8a1**	-1.9324	1.0593	8.2338	0.0041	0.3832	5	5	14	7	3	0	5	0	0	3
**Lrrc14b**	-1.9265	0.5487	3.9157	0.0478	0.8678	0	5	7	0	4	0	0	0	2	1
**Asb2**	-1.9258	2.2233	6.2933	0.0121	0.5677	0	9	2	4	0	1	0	0	0	2
**Gm34736**	-1.9195	0.7561	5.1417	0.0234	0.7084	5	3	11	1	1	2	0	0	2	0
**Slc17a7**	-1.8854	5.8646	8.3373	0.0039	0.3742	16	2	0	0	0	1	0	0	1	2
**Slc22a4**	-1.8805	1.3844	11.346	0.0008	0.1795	21	10	48	14	8	2	14	5	1	4
**Greb1**	-1.8804	3.4972	4.8298	0.028	0.7488	3	3	1	3	0	0	0	1	0	1
**Hba-a1**	-1.8794	7.1255	6.4112	0.0113	0.5543	189	6	6	4	13	31	14	14	0	4
**Ogg1**	-1.8792	0.8297	6.951	0.0084	0.5048	5	7	6	3	8	3	0	1	1	2
**Rgs5**	-1.879	5.3495	9.0219	0.0027	0.3197	27	0	1	3	3	1	3	0	0	5
**Gm36885**	-1.8739	1.0576	6.1561	0.0131	0.5875	14	29	16	24	5	2	17	0	0	5
**Ccdc3**	-1.862	2.2303	6.0657	0.0138	0.6019	117	1	6	9	7	4	10	18	0	10
**Ppef2**	-1.8617	0.2874	4.9238	0.0265	0.7377	3	12	26	8	0	3	4	3	0	1
**Gpr35**	-1.8572	3.1121	8.3936	0.0038	0.3726	3	3	11	4	1	2	0	0	0	3
**Col14a1**	-1.8486	4.4045	10.914	0.001	0.1951	21	0	10	1	2	2	0	1	5	0
**Gm14502**	-1.848	0.511	4.2427	0.0394	0.823	0	25	13	11	0	0	3	5	0	3
**Zfp418**	-1.8411	0.3229	5.5151	0.0189	0.662	3	9	2	7	2	1	0	0	2	2
**Prrt3**	-1.8377	4.2428	13.963	0.0002	0.0871	0	18	13	8	3	0	0	8	0	2
**Gm34079**	-1.8315	1.0883	7.6138	0.0058	0.4373	4	21	13	20	8	7	0	5	1	3
**Prokr2**	-1.828	2.7839	14.039	0.0002	0.0859	5	15	11	8	2	0	5	1	1	3

**Tmem44**	-1.8182	1.6081	9.1237	0.0025	0.3165	8	8	26	17	12	2	0	10	0	7
**Col15a1**	-1.8179	4.154	7.1419	0.0075	0.4864	16	1	0	0	0	0	0	0	1	3
**Ttll8**	-1.8154	1.7232	9.8478	0.0017	0.261	5	9	7	9	2	1	5	0	0	2
**LOC105244**	-1.8056	0.3828	4.3251	0.0376	0.8131	4	6	8	8	10	0	0	3	3	3
**Gm20036**	-1.8033	0.9426	6.3534	0.0117	0.5582	10	5	9	9	1	0	8	0	1	0
**Eva1c**	-1.7948	2.504	9.8247	0.0017	0.261	13	4	5	5	1	2	0	2	0	3
**Flt1**	-1.7874	3.9971	5.6544	0.0174	0.6381	11	0	2	0	0	0	0	1	1	1
**8430431K1**	-1.7849	0.4914	4.2827	0.0385	0.8177	7	4	5	5	0	1	0	2	0	2
**Glis1**	-1.7837	1.0249	10.097	0.0015	0.2474	15	20	25	15	3	3	5	1	3	7
**Glb1l**	-1.7826	0.7753	6.9972	0.0082	0.4992	7	7	6	5	0	2	0	2	0	2
**Gm40578**	-1.7772	0.8259	4.231	0.0397	0.8235	0	3	3	8	0	0	0	0	2	1
**Calml4**	-1.777	2.0612	4.7533	0.0292	0.7585	8	5	0	0	0	0	0	1	0	2
**Gm31282**	-1.7728	2.8228	12.6	0.0004	0.1273	6	28	8	5	11	5	0	2	6	1
**Ino80dos**	-1.7713	1.4714	6.0773	0.0137	0.6008	14	33	18	4	11	0	15	0	1	7
**Gm38398**	-1.7691	0.5458	5.4824	0.0192	0.6697	5	10	13	11	8	1	1	8	0	3
**Gm527**	-1.7675	1.6805	10.186	0.0014	0.2424	6	5	7	10	3	2	1	2	0	3
**Cldn5**	-1.767	3.7406	6.2413	0.0125	0.5756	13	0	0	0	3	0	0	0	1	3
**C4b**	-1.7665	3.1884	8.7315	0.0031	0.3385	3	6	6	7	2	0	0	4	0	2
**Syp**	-1.74	6.9653	4.1934	0.0406	0.8329	6	3	0	0	0	0	0	1	1	0
**Gab3**	-1.7376	1.6187	4.6084	0.0318	0.7776	3	7	0	3	0	3	0	0	0	0
**Mlc1**	-1.7286	3.8998	9.9438	0.0016	0.2574	9	7	8	1	2	2	1	0	0	4
**Sh2d5**	-1.7281	2.924	5.8641	0.0155	0.6198	1	14	2	0	0	0	0	3	0	1
**Gabrb1**	-1.7233	3.0987	3.8471	0.0498	0.8741	0	5	1	2	1	2	0	0	0	0
**Cox4i2**	-1.7227	0.7169	4.8511	0.0276	0.7469	3	12	4	5	0	1	0	0	4	0
**Caskin1**	-1.7223	5.7686	13.845	0.0002	0.0893	8	14	5	11	0	3	0	0	3	4
**Hipk4**	-1.7203	1.1851	4.4986	0.0339	0.7963	1	23	15	1	0	1	0	6	0	2
**Fkbp1b**	-1.72	6.8829	6.6069	0.0102	0.543	2	0	8	4	2	0	0	2	0	2
**Gm973**	-1.7165	1.4193	5.3031	0.0213	0.6958	26	6	6	0	7	9	0	4	0	0
**C3ar1**	-1.7156	2.2285	10.262	0.0014	0.2372	4	20	13	6	4	0	0	4	1	7
**A930015D0**	-1.7118	0.8573	4.5666	0.0326	0.7847	16	4	2	2	6	6	2	1	0	0
**Slc1a6**	-1.7068	2.1144	8.8037	0.003	0.3369	10	12	13	0	4	1	0	5	2	2
**Gm39069**	-1.7052	1.0978	5.1987	0.0226	0.7032	7	10	8	4	2	2	0	6	0	0
**Gm40074**	-1.7036	1.5554	4.1272	0.0422	0.8429	0	2	6	7	1	0	0	0	0	4
**Fbp2**	-1.6982	2.0445	4.7308	0.0296	0.7635	0	6	0	3	13	5	0	2	0	0
**Padi1**	-1.6938	2.4026	5.0387	0.0248	0.7219	110	66	203	52	71	8	32	0	26	69
**Gm34845**	-1.6791	0.7962	9.0874	0.0026	0.3165	16	19	22	8	15	2	5	9	1	7
**Gm41678**	-1.6651	0.5544	4.0638	0.0438	0.8522	0	12	2	9	6	0	5	3	0	1
**Slc35f1**	-1.6644	5.8185	4.7922	0.0286	0.7541	6	1	5	0	0	0	0	0	1	2
**Snhg11**	-1.6631	7.8529	9.2327	0.0024	0.3133	17	10	6	0	0	1	0	6	0	2
**Pcdh11x**	-1.662	3.3115	6.2117	0.0127	0.5801	7	0	1	7	3	1	1	3	0	0
**2410002F2**	-1.6608	0.5686	5.0087	0.0252	0.7253	3	12	17	2	2	1	1	5	1	1
**Gm38863**	-1.6593	1.434	4.0736	0.0436	0.85	0	7	2	3	1	0	0	0	3	0
**Plek**	-1.6568	2.0983	4.9597	0.0259	0.7335	0	6	0	9	5	1	4	0	0	1
**LOC102637**	-1.6422	1.0403	7.1222	0.0076	0.4874	20	21	19	9	10	9	10	4	1	0
**Slc38a4**	-1.6339	1.4881	7.6527	0.0057	0.434	7	12	14	15	4	2	0	7	3	2
**Rasl10b**	-1.6259	4.3167	7.8663	0.005	0.4129	1	15	3	0	3	0	0	0	4	2
**Palm2**	-1.6247	5.3892	15.188	1E-04	0.0631	11	12	18	2	9	1	0	8	2	4
**Coro1a**	-1.6219	4.6673	5.5377	0.0186	0.6585	3	1	6	4	1	0	0	2	2	0
**Gm38720**	-1.6205	1.4834	6.7848	0.0092	0.5176	13	28	48	30	19	10	8	18	2	4
**Gm41834**	-1.6054	1.4328	7.1292	0.0076	0.4871	5	5	12	30	10	4	9	1	0	7
**Dmtn**	-1.6039	5.9393	21.234	4E-06	0.0162	3	20	14	9	21	2	7	2	5	4
**Clec7a**	-1.6	1.6521	3.9897	0.0458	0.8609	14	0	5	0	5	0	2	4	0	2
**Kcnj16**	-1.599	2.9725	4.1496	0.0416	0.8381	48	13	11	0	7	0	0	26	0	0
**Glis3**	-1.5931	1.0444	6.7878	0.0092	0.5176	26	17	13	4	4	3	2	5	6	1
**Cfh**	-1.5928	3.1074	11.912	0.0006	0.1538	24	5	16	8	8	3	1	4	0	11
**Sox8**	-1.591	2.8391	4.3984	0.036	0.811	1	0	18	0	0	1	2	0	0	2
**Ryr3**	-1.5819	2.6544	6.646	0.0099	0.5362	4	5	4	4	4	5	0	0	1	0
**Cntfr**	-1.5797	2.9352	8.1917	0.0042	0.3872	2	10	12	4	0	1	5	0	0	2
**Crispld1**	-1.5726	2.8979	4.0729	0.0436	0.85	7	2	0	0	2	2	1	0	0	0
**Gm6607**	-1.5716	-0.0465	4.2778	0.0386	0.8189	4	6	7	5	2	1	4	0	1	1
**Avil**	-1.5712	7.7765	9.1021	0.0026	0.3165	7	1	7	3	9	6	0	0	0	2
**Gm40409**	-1.5672	0.9019	9.8862	0.0017	0.2605	9	11	25	10	13	4	6	6	0	6

**Zfr2**	-1.5627	3.0985	14.254	0.0002	0.0813	4	34	16	18	3	7	8	5	1	1
**LOC102631**	-1.5575	1.4586	4.9545	0.026	0.7335	15	3	20	3	2	3	5	4	1	0
**Zfp711**	-1.5563	2.1146	6.3861	0.0115	0.5577	2	2	8	9	3	0	0	0	2	5
**Zfp382**	-1.5458	4.3512	17.204	3E-05	0.0415	29	18	12	12	5	9	0	3	5	6
**A430090L1**	-1.5434	0.8936	6.7906	0.0092	0.5176	6	17	11	11	7	3	2	3	4	3
**Gm33350**	-1.5426	0.8134	5.1617	0.0231	0.707	11	27	12	10	5	3	0	1	5	8
**Spp1**	-1.533	4.281	4.8322	0.0279	0.7488	5	5	4	0	0	0	3	0	0	1
**Scarna17**	-1.5126	1.5325	6.7205	0.0095	0.525	12	24	27	17	3	2	6	1	4	11
**Ildr2**	-1.5101	3.7122	8.2228	0.0041	0.3832	11	9	3	5	4	5	0	5	0	0
**Tyrp1**	-1.5097	1.9516	4.2451	0.0394	0.823	27	17	15	11	0	11	0	8	0	2
**Gm26559**	-1.5089	1.3942	7.5008	0.0062	0.4459	6	20	7	10	7	4	6	0	5	0
**Tom1**	-1.5086	1.468	6.9129	0.0086	0.5048	12	11	5	11	4	3	0	1	3	6
**Hmgcll1**	-1.5002	2.1214	5.2577	0.0218	0.6987	0	11	5	4	4	1	0	3	2	1
**Cfap126**	-1.4985	1.2536	4.8476	0.0277	0.747	16	5	13	10	9	1	0	6	1	10
**Gm39165**	-1.4971	0.1203	4.7436	0.0294	0.7606	2	10	5	10	4	1	5	2	1	1
**St3gal6**	-1.4907	4.2565	7.0816	0.0078	0.4918	6	6	8	3	0	2	0	0	2	3
**Dock2**	-1.478	1.2228	4.3187	0.0377	0.8131	7	4	0	6	6	3	1	3	0	1
**Slc4a3**	-1.4582	3.8608	16.313	5E-05	0.0461	17	23	48	33	15	7	0	17	11	7
**LOC10816**	-1.4573	1.025	6.1609	0.0131	0.5875	11	26	4	9	4	6	0	3	3	4
**B3gat2**	-1.4504	1.3362	5.4272	0.0198	0.6761	14	22	20	9	2	3	0	10	2	5
**Alox15**	-1.4483	1.8178	5.6746	0.0172	0.6371	39	44	35	13	1	6	5	8	5	15
**Ctxn1**	-1.4481	4.1656	9.1905	0.0024	0.3137	3	25	4	4	2	1	4	5	1	1
**Apbb1ip**	-1.4474	1.3653	6.6266	0.01	0.5403	13	17	11	8	1	7	0	2	2	4
**Rtn4r**	-1.4399	2.3644	10.127	0.0015	0.2468	11	13	9	15	2	5	0	4	3	4
**Scn8a**	-1.4356	7.4576	4.2369	0.0396	0.823	6	2	0	3	2	0	0	2	2	0
**Ap3b2**	-1.4348	7.2095	4.1688	0.0412	0.8374	9	3	0	0	1	3	0	0	1	0
**Efr3b**	-1.4253	4.3173	5.6709	0.0172	0.6378	5	11	2	0	1	2	0	0	3	1
**Slc38a3**	-1.4233	1.5032	3.8643	0.0493	0.8716	5	8	7	0	0	2	0	4	0	0
**Ccdc177**	-1.4058	1.8299	5.7803	0.0162	0.6285	8	8	17	9	1	0	10	2	0	3
**Osbp2**	-1.4057	5.1718	11.962	0.0005	0.1514	20	9	12	5	2	2	0	5	6	3
**LOC10816**	-1.4033	0.5679	4.5046	0.0338	0.7954	7	7	10	12	9	0	13	4	1	0
**Pde1a**	-1.4026	2.9758	5.3684	0.0205	0.6861	1	2	5	14	4	0	2	3	1	3
**Adcy1**	-1.4016	4.9162	12.86	0.0003	0.1161	17	6	10	17	0	4	6	1	4	2
**Ccdc148**	-1.4013	2.7387	8.1601	0.0043	0.3886	10	20	12	9	2	6	0	5	1	5
**Thsd7a**	-1.379	6.8014	5.2283	0.0222	0.6995	15	0	0	1	2	0	0	0	1	5
**Tmc7**	-1.3736	2.2548	5.7551	0.0164	0.6292	3	4	3	19	4	4	3	2	0	3
**Gm33977**	-1.3656	0.5856	3.9192	0.0477	0.8678	8	15	13	11	11	4	0	8	3	5
**Gm33219**	-1.3641	1.1524	5.2762	0.0216	0.6958	3	19	11	39	10	2	13	6	1	10
**Lrp11**	-1.3529	5.6816	11.986	0.0005	0.151	12	21	5	11	0	0	2	4	4	7
**Shox2**	-1.3497	5.2686	9.4858	0.0021	0.2843	2	22	11	0	6	8	0	1	1	4
**Itih2**	-1.3475	2.139	4.1569	0.0415	0.8377	2	8	15	3	1	0	7	2	0	1
**Gm41491**	-1.3448	1.1396	5.77	0.0163	0.6285	9	38	8	15	4	5	8	9	0	4
**Chst1**	-1.3397	3.4798	4.4337	0.0352	0.8074	19	0	2	0	3	0	2	2	1	4
**Gm42259**	-1.3386	2.129	12.008	0.0005	0.151	16	36	24	33	11	9	9	11	6	7
**Gypc**	-1.3324	3.0201	5.1935	0.0227	0.7044	2	8	4	5	4	3	0	4	0	1
**C77370**	-1.3199	2.8271	7.8557	0.0051	0.4133	13	17	14	3	4	2	0	6	6	3
**Ap2a1**	-1.3196	1.4963	4.5182	0.0335	0.7919	6	13	9	2	10	0	10	1	1	4
**Cpb1**	-1.3163	1.6615	5.8729	0.0154	0.6181	10	12	4	8	9	0	8	2	1	6
**Sdpr**	-1.3161	3.9105	5.1489	0.0233	0.7084	33	2	2	9	1	8	1	4	0	5
**Ntn4**	-1.3033	0.7738	4.1254	0.0422	0.8429	6	15	9	10	9	0	0	5	7	4
**Eml1**	-1.2972	5.0709	6.9151	0.0085	0.5048	2	3	17	4	2	7	0	0	0	3
**Syt1**	-1.2968	7.4763	9.8543	0.0017	0.261	27	0	8	0	8	11	0	0	1	4
**Slc35g2**	-1.2959	2.5843	4.3615	0.0368	0.8127	4	11	1	7	0	0	0	3	0	5
**LOC10263**	-1.2931	1.7201	6.3724	0.0116	0.5577	10	25	17	26	5	5	4	5	4	11
**Foxp2**	-1.2873	3.4751	10.297	0.0013	0.2339	21	39	8	16	3	16	5	3	1	6
**Gm42339**	-1.2859	1.52	4.6952	0.0302	0.7699	4	11	22	12	4	3	0	11	0	5
**9330104G**	-1.2795	2.0305	10.354	0.0013	0.2316	27	24	42	21	18	11	7	11	9	10
**Adamts9**	-1.2752	2.3724	4.905	0.0268	0.7377	1	8	12	13	1	4	0	3	2	3
**Daam2**	-1.2749	4.0999	6.5999	0.0102	0.5433	2	7	6	6	9	0	0	4	5	2
**Pcsk4**	-1.2671	0.8916	6.1484	0.0132	0.5893	9	7	9	10	6	1	5	7	1	2
**Runx3**	-1.2658	3.8764	6.7987	0.0091	0.5176	14	16	3	2	0	1	9	1	1	1
**Gm35248**	-1.2583	1.4788	4.7778	0.0288	0.7543	24	36	45	12	3	9	2	19	3	8

**Hist1h2be**	-1.2564	2.4234	6.9184	0.0085	0.5048	11	1	17	5	8	0	3	2	4	7
**Lzts1**	-1.2545	3.7029	4.033	0.0446	0.8574	2	12	2	0	1	0	0	0	2	4
**Scn2a1**	-1.2537	3.3964	3.9605	0.0466	0.8665	5	9	3	0	0	0	0	2	2	2
**BC068281**	-1.2513	3.279	12.471	0.0004	0.1332	20	39	50	21	7	7	3	18	11	8
**Kctd14**	-1.2488	1.0413	3.9318	0.0474	0.8678	5	6	7	3	4	1	1	3	3	1
**Lncpint**	-1.2475	2.2897	9.7687	0.0018	0.2615	23	24	43	43	16	8	6	25	4	15
**Dclk1**	-1.2442	6.2388	4.6393	0.0312	0.7726	11	2	5	0	1	0	3	0	2	2
**Gm7467**	-1.2408	1.141	4.1241	0.0423	0.8429	13	3	36	16	5	4	7	8	2	7
**Neurl1a**	-1.2338	4.3001	4.376	0.0364	0.8112	6	6	4	1	2	2	0	0	1	4
**Adam19**	-1.2335	4.5072	5.2442	0.022	0.6995	6	6	9	1	2	0	3	2	0	4
**Zfp882**	-1.2284	2.1355	7.4121	0.0065	0.4561	13	30	15	7	5	4	8	5	3	6
**Gm16599**	-1.2246	1.2586	7.7575	0.0053	0.4269	16	19	26	28	14	7	15	7	4	9
**Gm40208**	-1.2229	1.9813	7.8662	0.005	0.4129	17	22	28	35	10	5	16	11	4	9
**Tspyl4**	-1.219	6.1723	13.19	0.0003	0.1101	17	30	19	14	8	10	5	9	1	9
**Car14**	-1.2144	2.3127	8.4795	0.0036	0.3649	8	21	14	16	9	5	12	2	3	5
**Mfsd7c**	-1.2143	1.476	8.1676	0.0043	0.3886	13	20	39	24	5	6	17	7	4	5
**BC031361**	-1.2127	1.3016	4.8273	0.028	0.7488	6	17	11	9	4	8	3	1	3	2
**9430020K0**	-1.2123	4.6311	20.407	6E-06	0.0188	42	38	30	10	22	11	8	16	2	20
**Atp6v1g2**	-1.2091	5.9025	4.8917	0.027	0.7405	1	6	3	4	7	1	0	3	1	3
**Gdap1**	-1.2089	6.8363	3.8516	0.0497	0.8726	2	0	7	1	6	0	0	2	2	2
**Pou4f1**	-1.2015	7.2151	5.8445	0.0156	0.6238	1	5	11	5	4	5	0	2	0	3
**Gm12002**	-1.1994	0.8395	5.0461	0.0247	0.7214	10	18	13	13	2	1	2	5	5	7
**Ptgds**	-1.1962	10.453	6.9387	0.0084	0.5048	1095	307	324	279	100	442	87	183	19	145
**Ehhadh**	-1.1956	0.6562	4.0073	0.0453	0.8577	3	8	7	7	5	4	3	0	0	5
**Sel1l3**	-1.1935	4.134	12.131	0.0005	0.1486	14	24	20	8	6	9	1	0	1	17
**Itga7**	-1.1798	4.296	7.7221	0.0055	0.4316	0	11	16	6	10	1	2	2	3	9
**Rasip1**	-1.1735	2.8569	5.9909	0.0144	0.6045	16	14	2	6	1	2	12	0	1	1
**Gm32853**	-1.1721	1.39	5.8121	0.0159	0.6278	12	41	20	28	7	11	19	9	0	6
**Aldh18a1**	-1.167	2.2898	7.0943	0.0077	0.4918	7	22	5	10	9	8	2	2	3	6
**Lenep**	-1.1618	3.2414	7.3433	0.0067	0.4636	159	44	32	33	19	18	57	25	9	22
**Spata33**	-1.1596	2.7103	6.8434	0.0089	0.5138	10	6	10	10	6	5	0	4	2	6
**Fsd1l**	-1.1593	4.6429	9.3754	0.0022	0.2963	12	8	13	6	10	7	7	1	3	2
**Dnd1**	-1.1531	1.6765	6.5788	0.0103	0.5435	13	17	24	14	10	5	1	11	1	14
**Gm9833**	-1.1474	1.7026	5.7145	0.0168	0.6337	9	27	31	14	11	13	0	14	2	7
**Thy1**	-1.147	8.4865	5.0166	0.0251	0.7232	10	10	0	0	5	1	0	0	3	6
**H19**	-1.1427	3.8566	6.7668	0.0093	0.5212	16	23	11	2	5	4	9	3	3	4
**Snhg14**	-1.1418	4.4438	5.9507	0.0147	0.6067	2	20	8	6	0	0	0	10	1	3
**Bnc2**	-1.1374	4.7239	11.286	0.0008	0.1801	22	13	9	11	10	4	0	9	4	10
**Gadd45g**	-1.1293	3.5516	10.486	0.0012	0.2302	27	61	41	23	22	12	3	20	9	25
**Rgs4**	-1.1281	8.7798	4.1493	0.0417	0.8381	5	2	10	0	3	2	0	0	4	2
**Rn7sk**	-1.1227	1.7904	4.0541	0.0441	0.8533	17	16	20	34	8	18	6	8	4	3
**Ciart**	-1.1218	1.9202	6.4739	0.0109	0.5486	8	42	33	18	16	7	26	9	1	9
**Dtwd2**	-1.1156	1.9243	4.478	0.0343	0.7996	14	23	6	21	4	4	9	2	4	9
**Nap1l5**	-1.0979	7.2485	8.6127	0.0033	0.3491	5	10	16	14	3	4	0	0	7	9
**Snap25**	-1.0901	8.9819	4.9137	0.0266	0.7377	11	6	0	0	9	1	0	0	2	8
**Slc29a2**	-1.0899	2.8158	9.581	0.002	0.2774	13	26	20	12	2	12	8	3	4	3
**Nap1l3**	-1.0867	4.6919	10.892	0.001	0.1957	22	28	17	11	3	3	9	7	5	10
**Grin1**	-1.0735	2.7471	4.3543	0.0369	0.8131	8	7	28	5	1	5	0	6	3	5
**Gm41461**	-1.0677	1.3962	4.1326	0.0421	0.8426	20	28	21	12	14	15	0	15	4	6
**Gap43**	-1.0635	7.4252	13.016	0.0003	0.1116	22	22	43	6	11	11	8	18	2	6
**Traip**	-1.0564	1.7734	7.4287	0.0064	0.4537	22	24	32	22	9	16	4	16	2	9
**Cdk5r1**	-1.0547	6.1828	12.445	0.0004	0.1332	9	15	43	16	2	7	2	7	9	11
**Usp13**	-1.0508	5.8336	9.4438	0.0021	0.2865	16	11	24	4	5	9	1	2	3	11
**Fndc1**	-1.0383	2.2938	6.3802	0.0115	0.5577	69	17	56	34	31	21	18	22	6	33
**Zfp780b**	-1.038	2.8467	9.9191	0.0016	0.2581	16	42	50	43	13	7	7	19	18	15
**Tmem150c**	-1.0367	4.8035	7.0807	0.0078	0.4918	7	11	7	10	10	2	0	11	2	5
**Gm9222**	-1.0363	2.5435	8.1519	0.0043	0.3888	20	25	15	16	9	5	21	5	4	4
**Adhfe1**	-1.033	1.0914	5.7216	0.0168	0.6337	6	10	12	13	3	4	3	5	4	3
**Slc24a2**	-1.0252	7.735	12.302	0.0005	0.1416	10	20	27	24	8	14	0	7	8	10
**Rab36**	-1.0252	3.0469	5.7896	0.0161	0.6285	13	9	23	7	4	2	3	10	5	4
**Gm39656**	-1.0165	1.8931	4.646	0.0311	0.7726	11	26	14	22	16	4	9	18	4	7
**Gm30294**	-1.0155	1.9903	4.4964	0.034	0.7963	32	45	35	29	16	15	9	23	15	2

**Mmd**	-1.014	2.7338	5.1441	0.0233	0.7084	25	20	48	11	1	2	5	7	8	20
**Brsk1**	-1.0089	5.979	3.9739	0.0462	0.8611	5	10	5	2	3	6	0	0	0	5
**Garnl3**	-1.0066	4.2538	6.4963	0.0108	0.5486	12	30	4	8	0	5	0	2	4	12
**Wipf3**	-1.006	6.7265	13.019	0.0003	0.1116	27	19	26	11	7	15	10	11	0	5
**Faim2**	-1	6.0903	5.0836	0.0242	0.7184	4	16	5	0	8	2	9	0	0	4
**Bbs12**	-0.9951	1.7508	5.8154	0.0159	0.6274	13	23	25	23	2	7	13	7	4	7
**Brsk2**	-0.9936	5.7935	4.6702	0.0307	0.772	12	7	9	0	1	0	0	9	2	2
**Sdc3**	-0.9887	5.0655	8.5345	0.0035	0.3582	18	13	14	11	3	8	4	9	1	5
**Ankrd6**	-0.9859	3.4565	5.6393	0.0176	0.6392	17	7	23	6	3	3	7	13	0	3
**Tmem120b**	-0.9856	2.6614	6.3562	0.0117	0.5582	8	21	13	5	6	10	7	2	1	4
**Kcnu1**	-0.9853	2.211	4.0314	0.0447	0.8574	14	10	14	9	18	4	5	10	0	15
**Tle6**	-0.9842	1.99	4.8156	0.0282	0.7493	13	30	16	28	9	1	6	17	4	16
**1700010I1**	-0.9704	1.3925	4.9513	0.0261	0.7335	29	25	30	32	17	10	3	27	7	15
**Helq**	-0.9663	1.8192	7.2424	0.0071	0.4752	18	20	17	15	13	8	2	10	8	10
**Mtfp1**	-0.9657	3.1889	6.7211	0.0095	0.525	12	24	17	3	7	4	8	5	2	10
**Nefm**	-0.9619	11.857	5.3375	0.0209	0.6908	52	14	11	11	34	8	6	2	20	22
**Gm41287**	-0.9582	2.1376	3.9287	0.0475	0.8678	18	48	59	30	9	20	0	34	8	6
**Slbp**	-0.957	1.6303	4.4602	0.0347	0.8027	12	32	32	14	9	4	16	8	8	8
**Gm5093**	-0.9554	4.7629	4.1987	0.0405	0.8329	3	6	10	3	6	3	4	4	1	1
**Mecom**	-0.9534	2.0887	6.0556	0.0139	0.6019	39	49	36	17	26	28	9	27	3	14
**Nckap5**	-0.9533	1.7547	4.574	0.0325	0.7827	9	13	41	15	14	6	22	12	3	3
**Tox3**	-0.9481	4.3727	5.0811	0.0242	0.7184	23	9	7	5	1	8	0	2	1	10
**Hap1**	-0.9444	2.3473	5.4821	0.0192	0.6697	7	20	22	13	6	7	7	11	0	7
**LOC10816**	-0.9418	1.4537	4.2116	0.0401	0.8291	7	45	30	21	14	16	12	11	2	14
**LOC10816**	-0.9392	2.2614	3.9705	0.0463	0.8618	15	21	16	14	10	7	6	9	4	10
**Nudt12**	-0.9379	3.5701	9.1342	0.0025	0.3165	35	39	45	38	16	29	7	8	13	21
**Fgf18**	-0.935	4.6327	7.0529	0.0079	0.4954	17	19	31	8	7	4	3	21	4	6
**LOC10816**	-0.9349	2.3085	6.8048	0.0091	0.5175	28	64	66	49	25	14	45	44	5	9
**Areg**	-0.9188	2.4425	3.8441	0.0499	0.8743	62	65	49	57	8	24	12	24	14	33
**Ank2**	-0.9169	8.7876	5.964	0.0146	0.6057	22	4	16	0	4	3	1	3	5	10
**Dennd2a**	-0.909	3.0007	6.5441	0.0105	0.5456	6	19	22	12	12	6	1	7	10	9
**Dennd4b**	-0.9081	3.4422	7.9655	0.0048	0.4019	9	35	29	18	7	14	11	7	4	10
**Cep192**	-0.908	2.562	8.0513	0.0045	0.3978	43	62	47	43	30	11	27	19	19	32
**Gm15764**	-0.9031	3.2401	4.7437	0.0294	0.7606	43	138	99	104	28	9	100	60	15	25
**Basp1**	-0.8958	8.5415	7.8042	0.0052	0.4214	118	35	32	24	13	9	48	37	6	20
**Plxnd1**	-0.8927	4.5633	5.7224	0.0167	0.6337	20	10	10	0	11	6	5	1	8	5
**Entpd3**	-0.8907	6.2468	15.651	8E-05	0.055	16	67	90	69	15	23	36	39	6	21
**Mobp**	-0.8897	6.4906	7.4783	0.0062	0.4459	7	17	19	34	16	8	10	11	1	18
**Gm30189**	-0.8838	2.1034	4.3442	0.0371	0.8131	21	24	45	26	8	5	8	20	12	11
**Zbtb39**	-0.8836	2.6453	4.3866	0.0362	0.811	36	37	65	62	16	27	15	25	10	27
**Fam13a**	-0.8835	4.1503	5.4664	0.0194	0.6697	34	30	16	17	2	24	0	18	1	4
**Pacsin1**	-0.8811	7.2714	4.4434	0.035	0.8062	10	11	8	6	2	2	0	8	2	6
**Hspb6**	-0.8774	3.719	8.2584	0.0041	0.382	46	66	33	41	14	29	27	12	9	21
**Zfp956**	-0.8742	2.6371	4.8222	0.0281	0.7493	29	45	46	34	6	19	10	39	1	9
**Ksr2**	-0.8711	4.0328	4.302	0.0381	0.8131	5	18	10	2	5	0	0	3	7	9
**Arhgap33**	-0.8711	2.8805	4.9665	0.0258	0.7335	14	24	41	26	9	14	17	21	3	2
**Rfx3**	-0.8698	2.9198	9.0597	0.0026	0.3187	21	34	52	34	24	12	31	23	3	18
**LOC10524**	-0.869	2.986	4.408	0.0358	0.811	45	99	84	66	37	17	57	68	9	24
**Klf12**	-0.8635	3.7517	5.8071	0.016	0.628	12	17	12	22	1	10	7	12	0	3
**Gm36800**	-0.8555	2.6617	7.7179	0.0055	0.4316	51	61	51	73	20	26	41	28	13	23
**Pros1**	-0.8549	3.1307	4.1076	0.0427	0.8447	12	5	10	12	7	5	10	2	3	4
**Spire2**	-0.8494	5.2591	11.066	0.0009	0.1883	17	83	48	36	14	16	24	18	17	19
**Amigo1**	-0.8436	5.0186	14.339	0.0002	0.0808	32	67	27	35	15	24	17	24	5	19
**Gm40475**	-0.84	1.9756	3.8923	0.0485	0.8678	17	50	44	32	23	17	29	22	4	17
**Rimbp2**	-0.8363	4.6176	5.7948	0.0161	0.6285	9	6	14	11	14	6	7	3	4	8
**Arhgef39**	-0.835	1.8415	4.9503	0.0261	0.7335	33	37	31	27	12	23	6	17	3	22
**Nog**	-0.8348	2.2708	4.3659	0.0367	0.8126	11	18	19	3	9	8	10	5	1	7
**Scn7a**	-0.8331	7.9336	4.2831	0.0385	0.8177	1	8	9	8	11	0	9	3	4	3
**LOC10816**	-0.8307	2.8808	6.7578	0.0093	0.5214	31	57	38	50	18	10	18	40	8	24
**Gm9769**	-0.8306	2.5472	7.1667	0.0074	0.4832	42	39	37	22	12	22	19	8	8	20
**Hcar1**	-0.8279	2.0604	5.7156	0.0168	0.6337	40	32	53	33	14	19	19	21	8	21
**Gm17296**	-0.8268	2.0213	4.4076	0.0358	0.811	17	25	9	23	10	12	5	12	5	9

**Rsph9**	-0.8215	2.8757	5.6955	0.017	0.6337	16	18	20	17	11	2	17	9	3	13
**Caprin2**	-0.8183	3.8252	7.4232	0.0064	0.4542	23	43	42	33	15	1	24	31	9	16
**Cpne2**	-0.8126	4.3458	6.9966	0.0082	0.4992	19	30	10	17	10	12	11	7	3	12
**Cntnap1**	-0.809	5.038	4.1772	0.041	0.8351	0	27	6	4	5	2	3	3	7	6
**Zfp958**	-0.8063	3.1404	5.048	0.0247	0.7214	40	44	37	48	29	18	3	18	11	54
**Gm35290**	-0.8039	2.145	4.3378	0.0373	0.8131	28	44	65	31	5	9	14	34	7	21
**Gm33585**	-0.8006	2.755	6.5905	0.0103	0.5434	46	69	45	47	38	24	51	37	5	24
**Cep170**	-0.8001	5.4385	4.1599	0.0414	0.8377	2	19	9	13	4	3	9	2	6	4
**LOC10816{**	-0.7927	1.476	4.0272	0.0448	0.8574	22	22	19	28	7	10	12	11	7	11
**Slx1b**	-0.7914	2.8526	4.628	0.0315	0.7737	20	34	45	16	25	17	41	11	1	12
**Syt3**	-0.7903	5.2721	8.9406	0.0028	0.3247	22	40	68	30	13	29	11	32	3	14
**Tmtc1**	-0.7865	6.2103	4.3965	0.036	0.811	14	15	12	0	1	7	4	5	0	6
**Ppp1r9a**	-0.7734	6.1476	9.7078	0.0018	0.2651	19	34	32	20	7	11	9	10	8	21
**Kcnb1**	-0.7692	6.6657	6.6092	0.0101	0.543	41	13	19	10	3	11	14	7	1	14
**Psme2b**	-0.7682	1.7812	4.5663	0.0326	0.7847	19	41	44	26	24	17	38	15	9	7
**Akap5**	-0.7642	6.473	6.7594	0.0093	0.5214	19	24	29	9	7	1	7	16	11	11
**Aff3**	-0.7639	5.053	7.8657	0.005	0.4129	79	30	24	42	22	21	31	29	10	22
**Plekho1**	-0.7618	4.6019	5.9392	0.0148	0.6085	31	23	40	19	9	14	11	10	5	25
**Eml5**	-0.7571	3.8596	8.0386	0.0046	0.3979	61	121	73	64	27	27	33	72	18	31
**Gm31262**	-0.7563	2.6822	8.6983	0.0032	0.341	56	64	63	46	34	31	44	30	12	30
**Hhip**	-0.7488	2.6527	5.8841	0.0153	0.6176	17	57	70	36	23	29	19	21	11	26
**Kctd4**	-0.7461	2.796	4.0479	0.0442	0.8549	13	31	26	30	23	8	18	34	6	5
**AK010878**	-0.7452	2.0426	3.9062	0.0481	0.8678	16	25	16	13	13	14	4	6	4	17
**Cnbd2**	-0.7436	2.1819	3.9578	0.0467	0.8665	17	33	38	20	9	12	30	9	1	14
**Zfp719**	-0.7401	3.1455	6.7072	0.0096	0.525	54	54	39	29	26	24	35	27	11	17
**Gpr153**	-0.7333	4.3349	6.2992	0.0121	0.5668	34	73	69	37	12	22	23	40	7	27
**LOC10524!**	-0.7302	2.77	4.8994	0.0269	0.7395	27	74	54	76	23	21	50	36	7	32
**Nkd2**	-0.7273	2.7648	4.4786	0.0343	0.7996	41	24	50	27	15	21	18	17	10	20
**Sestd1**	-0.7149	5.4625	11.018	0.0009	0.1888	28	68	59	29	31	18	18	42	15	25
**Pcgf6**	-0.7056	2.9195	6.0023	0.0143	0.604	33	51	48	61	27	24	18	22	16	42
**2410131K1**	-0.6999	3.1146	5.1176	0.0237	0.7136	22	27	30	25	19	18	11	22	4	16
**Dtd2**	-0.6974	3.0578	4.9809	0.0256	0.7319	41	60	61	54	38	32	28	27	21	34
**Tceal1**	-0.6966	3.9354	5.0933	0.024	0.7183	19	33	14	25	0	8	20	7	3	13
**Crtc3**	-0.6928	4.5499	17.581	3E-05	0.0381	88	143	125	91	46	50	52	63	34	73
**Med12l**	-0.6928	3.7217	4.0357	0.0445	0.8574	30	34	23	17	3	6	10	18	10	13
**Peg3**	-0.6905	6.9062	8.7256	0.0031	0.3385	25	100	110	62	35	23	54	51	25	29
**Nav2**	-0.689	3.515	5.2803	0.0216	0.6958	51	33	41	54	18	24	21	25	8	37
**Nrbp1**	-0.6875	4.0498	9.5063	0.002	0.2825	57	107	107	63	48	33	76	46	37	21
**Kcnd3**	-0.6869	4.1181	4.7881	0.0287	0.7541	24	39	18	15	5	16	0	16	3	20
**Masp1**	-0.6811	2.9989	6.5675	0.0104	0.5447	35	40	78	39	19	25	26	25	10	33
**Pde1c**	-0.6807	5.5927	6.109	0.0134	0.5959	11	23	36	29	6	29	5	9	6	9
**Ppp2r2b**	-0.6798	6.9679	8.4365	0.0037	0.3685	29	33	34	14	16	19	15	9	6	23
**Supt4a**	-0.674	2.8922	3.8955	0.0484	0.8678	19	47	45	13	25	14	3	27	13	24
**Dock8**	-0.6712	3.0642	4.8329	0.0279	0.7488	49	74	81	37	41	17	54	29	25	37
**Zdhhc17**	-0.6699	5.1857	8.7524	0.0031	0.3382	25	43	27	39	7	22	8	24	7	19
**Gm32394**	-0.6698	2.3389	3.8789	0.0489	0.8696	26	53	55	42	25	10	41	47	10	12
**Igf2**	-0.6658	5.5559	7.8447	0.0051	0.4149	104	114	131	79	70	65	86	60	39	40
**Pigg**	-0.6603	3.1388	4.6421	0.0312	0.7726	39	56	66	70	41	31	30	14	28	51
**Cdc42ep2**	-0.6596	4.6098	4.3698	0.0366	0.8119	12	11	20	22	5	12	13	10	1	5
**Car6**	-0.6594	2.1035	4.0294	0.0447	0.8574	50	52	59	38	29	42	33	25	12	22
**Klhl13**	-0.6505	4.0357	4.2379	0.0395	0.823	7	22	16	22	3	14	5	11	3	7
**Kctd18**	-0.65	2.8387	4.6088	0.0318	0.7776	27	72	43	37	11	20	16	32	7	31
**Cplx2**	-0.6486	6.2204	7.0547	0.0079	0.4954	23	27	28	15	18	8	16	21	11	9
**Dbf4**	-0.6472	3.3849	3.8962	0.0484	0.8678	97	177	92	94	38	104	35	46	34	47
**Tnfrsf11b**	-0.6436	1.7385	3.9937	0.0457	0.8598	31	39	32	30	19	18	21	18	8	25
**Pom121**	-0.6419	3.6074	4.3876	0.0362	0.811	44	82	63	59	62	46	20	41	34	36
**Uhrf1bp1**	-0.6362	3.7675	7.6038	0.0058	0.4375	104	66	96	107	28	51	78	39	26	46
**Dclre1c**	-0.6336	3.368	4.7607	0.0291	0.7563	26	55	61	31	7	20	30	20	3	31
**Msrb3**	-0.6303	3.0538	4.5862	0.0322	0.7798	29	33	39	28	13	9	26	27	2	23
**Zfp941**	-0.6298	3.5148	4.4753	0.0344	0.8002	24	43	20	26	20	13	15	26	10	15
**Poli**	-0.6217	3.0469	4.9978	0.0254	0.7274	31	93	62	35	34	18	48	34	23	25
**Zfp472**	-0.621	3.2336	5.2894	0.0215	0.6958	98	63	124	77	40	49	51	57	22	63

**Zmat1**	-0.6209	4.0196	4.7074	0.03	0.7694	37	49	20	28	43	32	29	22	16	12
**Mef2c**	-0.6196	6.143	4.0057	0.0453	0.8577	38	24	16	5	4	6	18	12	6	10
**Zfp72**	-0.616	2.9371	4.824	0.0281	0.7493	33	52	59	28	13	26	29	25	6	23
**Acta2**	-0.615	3.2312	4.0378	0.0445	0.8569	92	80	64	46	26	44	52	40	21	26
**Pprc1**	-0.6134	3.1335	4.7831	0.0287	0.7541	42	52	82	46	18	33	42	20	10	38
**Vit**	-0.6107	4.537	4.3236	0.0376	0.8131	77	48	49	43	27	44	68	30	8	9
**Cenpj**	-0.6102	3.8543	5.4284	0.0198	0.6761	92	105	98	82	54	76	44	51	35	46
**Rab23**	-0.6062	3.0277	4.5428	0.0331	0.7855	41	53	41	30	30	13	17	30	23	31
**Myadm**	-0.6043	5.594	15.956	6E-05	0.053	110	162	199	138	56	101	120	82	41	52
**Kcnq1ot1**	-0.6037	7.0778	7.7246	0.0054	0.4316	637	1057	1134	853	453	375	755	693	309	336
**B4galt4**	-0.6025	2.9845	4.9613	0.0259	0.7335	23	45	34	30	11	22	23	15	11	13
**9530091C0**	-0.6007	4.6199	5.8329	0.0157	0.6239	174	287	278	246	99	87	238	212	36	118
**Abca8a**	-0.5884	6.2809	6.3339	0.0118	0.5614	22	41	34	23	9	7	33	29	5	5
**Aldh5a1**	-0.5867	3.1092	4.0152	0.0451	0.8577	22	46	28	50	20	16	24	27	12	23
**Sepn1**	-0.5838	3.7875	4.3939	0.0361	0.811	17	55	58	35	9	10	34	30	10	19
**Ap3m2**	-0.5819	4.5572	4.3512	0.037	0.8131	65	49	49	17	22	27	20	33	6	40
**Xrcc4**	-0.5774	3.5911	6.1256	0.0133	0.5925	82	99	86	81	51	64	95	59	9	37
**Abat**	-0.5716	4.3133	4.1488	0.0417	0.8381	17	24	11	36	16	19	20	12	7	8
**Trmt10c**	-0.5622	3.3147	4.0125	0.0452	0.8577	34	59	49	80	28	26	31	38	19	41
**Rap2a**	-0.5566	5.7308	9.6124	0.0019	0.2767	49	75	95	64	33	31	28	48	24	62
**Chpt1**	-0.5495	5.2833	5.2342	0.0221	0.6995	25	53	49	39	24	24	15	14	12	52
**Pigo**	-0.5472	3.3666	3.9387	0.0472	0.8678	45	103	76	70	35	40	55	78	11	27
**Mcm9**	-0.5444	3.4811	5.0631	0.0244	0.7207	71	68	73	48	33	39	58	41	22	26
**Mex3a**	-0.5443	2.9471	3.8823	0.0488	0.8694	44	69	101	52	27	50	37	42	19	29
**Slc22a21**	-0.5425	3.6998	6.4859	0.0109	0.5486	82	187	176	130	63	67	101	110	24	103
**2700097O0**	-0.5395	2.7202	4.3817	0.0363	0.811	35	63	62	59	16	22	40	38	10	38
**Synm**	-0.539	8.0151	7.554	0.006	0.4432	33	58	36	40	36	32	46	20	8	26
**St7l**	-0.5384	3.7964	4.1087	0.0427	0.8447	45	31	71	35	22	21	35	22	25	22
**Tex10**	-0.5368	3.6828	3.9359	0.0473	0.8678	75	103	57	59	73	55	43	64	25	56
**Zfp182**	-0.5358	3.0822	4.6432	0.0312	0.7726	43	60	45	57	33	29	33	49	17	25
**Arhgef9**	-0.5357	5.3989	5.3094	0.0212	0.6958	45	134	77	81	30	64	17	65	19	54
**Ppfia4**	-0.5302	4.8674	4.8383	0.0278	0.7488	28	46	83	25	19	29	53	27	12	6
**Marf1**	-0.5279	7.2053	5.0823	0.0242	0.7184	602	971	750	690	215	426	724	557	136	244
**Fzd7**	-0.5275	3.9473	5.2888	0.0215	0.6958	65	131	133	88	41	80	81	46	29	47
**Zfp280b**	-0.5275	3.1888	5.4606	0.0194	0.6702	28	49	62	56	23	28	34	25	15	36
**Spaca6**	-0.5191	4.0229	4.3284	0.0375	0.8131	73	165	165	108	53	78	143	87	11	59
**Iqgap2**	-0.5187	4.2909	3.8555	0.0496	0.8726	165	94	93	90	28	58	105	69	29	48
**Usp30**	-0.5173	3.6348	4.4635	0.0346	0.8027	52	56	64	61	47	30	34	32	16	75
**Zbtb25**	-0.5168	3.3093	3.8982	0.0483	0.8678	42	69	86	59	29	42	52	42	17	29
**Gm39584**	-0.5152	4.3811	7.5595	0.006	0.4432	166	216	188	163	93	83	164	146	65	76
**Ctdp1**	-0.5136	3.7733	5.3546	0.0207	0.6891	85	95	120	72	40	52	80	49	25	60
**Soat1**	-0.5083	4.2677	3.9042	0.0482	0.8678	62	68	69	30	16	37	25	41	14	35
**Zmym1**	-0.5074	3.2778	4.0753	0.0435	0.85	36	34	59	66	31	32	35	29	24	26
**Slc25a38**	-0.504	4.748	7.1424	0.0075	0.4864	77	134	79	72	45	45	58	72	23	65
**Trerf1**	-0.5036	3.4828	3.8688	0.0492	0.8715	88	163	128	89	50	60	106	96	29	45
**Ilf2**	-0.4997	5.0798	9.2251	0.0024	0.3133	184	235	190	168	117	146	132	151	44	123
**Wdr70**	-0.498	4.7255	5.1101	0.0238	0.7149	110	197	140	110	43	111	96	98	24	54
**Abcc1**	-0.4978	3.4422	5.5347	0.0186	0.6586	59	72	65	46	36	28	34	44	30	41
**Ldoc1l**	-0.4955	4.2433	4.91	0.0267	0.7377	46	92	81	54	25	37	28	40	19	62
**Il4ra**	-0.4903	4.2715	5.5954	0.018	0.6485	54	75	39	58	27	21	44	38	27	34
**Ap1s2**	-0.4896	7.1036	6.1585	0.0131	0.5875	44	35	60	24	23	22	41	31	5	25
**Chka**	-0.4895	5.1751	7.4806	0.0062	0.4459	87	166	174	146	72	59	108	133	33	95
**Ece2**	-0.489	4.4945	5.9705	0.0145	0.6057	40	63	54	47	30	32	39	42	15	26
**Nrp2**	-0.4882	5.3546	6.9105	0.0086	0.5048	59	44	44	47	37	31	50	38	12	26
**Ndufaf2**	-0.4825	5.11	8.9762	0.0027	0.3226	99	94	114	101	51	89	82	44	34	54
**Akap12**	-0.4822	9.1806	5.2817	0.0216	0.6958	38	32	56	9	24	17	30	26	12	20
**Cnnm4**	-0.4773	3.8046	4.5476	0.033	0.7852	33	37	60	42	17	23	41	16	9	37
**L3mbtl3**	-0.4768	4.3659	6.1739	0.013	0.5852	83	133	128	97	45	50	96	87	16	79
**Mbd4**	-0.4758	3.6411	4.5398	0.0331	0.7861	46	65	80	37	31	35	42	31	14	49
**Abi3bp**	-0.474	4.9598	4.9091	0.0267	0.7377	305	351	338	282	172	191	314	256	84	154
**Zfp955b**	-0.4725	4.3593	5.8726	0.0154	0.6181	107	128	117	78	52	49	50	68	43	99
**Ndufaf5**	-0.4717	5.083	4.4132	0.0357	0.811	25	46	37	20	19	17	35	18	8	20

**Ccdc64**	-0.468	4.3904	6.5145	0.0107	0.546	109	130	158	111	56	82	90	59	27	119
**Smim12**	-0.4674	5.52	8.3572	0.0038	0.3742	60	83	93	72	40	43	55	56	29	47
**Hist1h1e**	-0.4656	6.2448	5.9769	0.0145	0.6057	574	794	435	294	329	372	320	310	228	327
**Nr2c2ap**	-0.4617	4.2825	5.7864	0.0162	0.6285	61	49	66	51	43	37	31	54	24	36
**Spopl**	-0.4591	4.6714	9.7941	0.0018	0.2615	110	179	165	141	67	83	138	97	32	99
**Zfp821**	-0.4584	4.2183	4.2709	0.0388	0.8196	76	142	155	104	37	66	122	56	42	46
**Herc1**	-0.4574	7.3753	20.98	5E-06	0.0162	441	593	576	474	261	286	436	371	182	293
**Sox9**	-0.4552	6.661	7.3071	0.0069	0.4698	1260	1377	1372	608	382	696	705	868	339	599
**Lsm7**	-0.4536	4.5037	4.38	0.0364	0.811	62	136	102	77	38	55	79	53	23	65
**Fmnl2**	-0.4521	6.1661	5.3802	0.0204	0.6835	56	60	75	43	27	11	36	53	25	48
**Usp39**	-0.4513	4.6044	5.2517	0.0219	0.6995	135	156	139	164	59	57	102	125	46	110
**Tpgs2**	-0.4511	5.5205	4.397	0.036	0.811	31	64	50	38	44	33	27	18	23	51
**Yars2**	-0.4505	4.0877	5.5325	0.0187	0.6586	51	87	89	62	32	42	51	51	27	40
**Tnik**	-0.4458	5.7222	6.7059	0.0096	0.525	161	229	171	106	62	105	135	120	41	86
**Utp15**	-0.4431	3.9651	4.8459	0.0277	0.747	57	110	110	80	52	31	98	80	27	47
**Paxbp1**	-0.4368	4.9601	5.5861	0.0181	0.6487	192	329	272	241	74	173	227	152	76	101
**Rbm3**	-0.4361	4.076	4.7044	0.0301	0.7694	82	127	143	105	82	56	106	86	50	72
**Arnt2**	-0.4349	5.054	4.7515	0.0293	0.7587	47	43	58	42	18	22	45	43	7	27
**Ercc4**	-0.4348	4.1959	8.4646	0.0036	0.3649	96	122	119	81	46	57	86	71	41	57
**Lamb3**	-0.4329	4.2298	5.0246	0.025	0.7219	199	323	188	206	96	151	148	157	90	113
**Fam89b**	-0.4319	5.8241	4.0929	0.0431	0.8484	32	84	62	22	38	42	26	45	19	26
**Nrg1**	-0.4313	5.6567	8.7382	0.0031	0.3385	107	192	201	130	66	77	97	133	51	104
**Znrf3**	-0.4302	3.6921	5.2799	0.0216	0.6958	80	112	101	102	61	67	67	69	26	88
**Zfp512**	-0.428	5.074	6.4187	0.0113	0.5538	141	223	160	150	82	126	150	103	61	71
**Mapk8ip3**	-0.4251	6.969	5.7751	0.0163	0.6285	50	51	93	53	15	36	46	37	17	41
**Fam160b1**	-0.4204	5.52	7.1561	0.0075	0.4843	166	320	244	195	73	130	182	120	55	184
**Etv5**	-0.4183	4.9144	4.6976	0.0302	0.7699	65	67	69	69	41	39	55	58	18	50
**Smc2**	-0.418	5.3104	6.5518	0.0105	0.5454	309	437	244	252	201	258	215	255	98	181
**D10Wsu10**	-0.4172	4.644	6.2317	0.0125	0.5758	115	150	155	117	66	90	110	86	50	75
**Efna3**	-0.4172	3.8544	4.9175	0.0266	0.7377	143	202	166	123	79	101	134	111	57	83
**Arhgef25**	-0.4166	6.1699	4.0765	0.0435	0.85	43	62	66	27	24	39	47	34	7	27
**Vstm5**	-0.4159	4.3572	3.9336	0.0473	0.8678	26	50	70	35	21	23	41	37	10	28
**Crebl2**	-0.4159	4.9238	4.8799	0.0272	0.7422	71	97	81	95	67	41	85	97	26	49
**Pcf11**	-0.4151	6.3062	10.011	0.0016	0.2542	406	761	584	531	294	427	503	421	146	302
**Pclo**	-0.413	7.4635	5.7204	0.0168	0.6337	126	165	132	87	73	65	93	121	63	51
**B3gat3**	-0.4102	5.8107	8.0886	0.0045	0.3937	110	179	193	98	74	94	146	64	48	98
**Micu3**	-0.4077	6.963	4.8034	0.0284	0.7519	56	57	47	47	32	27	41	57	18	25
**Igf1**	-0.4052	4.9244	4.2772	0.0386	0.8189	58	81	71	59	29	36	46	39	18	67
**E030024N2**	-0.3945	3.8482	3.925	0.0476	0.8678	72	120	103	73	65	58	75	74	29	72
**Cln6**	-0.3945	4.461	4.7402	0.0295	0.7615	188	237	188	200	71	153	150	115	51	146
**Izumo4**	-0.3939	4.0802	4.4034	0.0359	0.811	43	96	62	53	25	22	45	45	26	52
**Npepl1**	-0.3903	4.7158	4.9282	0.0264	0.7377	97	165	131	131	49	80	98	93	48	72
**Vim**	-0.3889	8.277	8.0197	0.0046	0.4007	176	192	187	133	75	107	125	154	42	111
**Afmid**	-0.3835	4.2628	4.3224	0.0376	0.8131	138	230	186	162	71	110	178	150	32	98
**Sobp**	-0.3782	5.7885	5.1715	0.023	0.7051	68	113	116	56	41	67	66	65	26	50
**Morc3**	-0.3767	4.5689	3.9248	0.0476	0.8678	108	224	166	148	130	116	91	148	65	130
**Anks3**	-0.3744	4.326	4.2845	0.0385	0.8177	93	128	113	118	51	64	121	101	24	59
**Celsr2**	-0.3718	6.4453	7.2348	0.0072	0.4764	419	659	652	510	247	371	595	399	152	276
**Zdhhc21**	-0.3717	7.0404	8.5454	0.0035	0.3571	563	897	707	536	254	394	598	553	188	349
**Crlf3**	-0.3715	4.8018	4.0165	0.0451	0.8577	105	215	195	130	67	102	136	152	28	91
**Uspl1**	-0.3696	4.7847	5.7815	0.0162	0.6285	66	135	115	109	40	80	89	62	41	51
**Vps13d**	-0.3687	5.3847	7.0871	0.0078	0.4918	136	198	158	156	91	93	154	130	42	121
**Phf1**	-0.3667	5.6023	4.1843	0.0408	0.834	198	206	262	234	68	139	257	144	65	93
**Vezt**	-0.3649	5.7683	11.435	0.0007	0.1764	186	250	285	190	99	166	191	135	75	147
**Fnbp4**	-0.3623	5.7059	4.7848	0.0287	0.7541	263	559	478	408	145	208	528	256	137	187
**Haus2**	-0.3619	4.3734	4.2564	0.0391	0.8215	104	141	107	79	39	77	96	67	31	62
**Kansl1l**	-0.3578	4.7387	3.9217	0.0477	0.8678	146	209	189	158	98	124	166	128	66	92
**Ankrd12**	-0.3575	8.2715	6.4661	0.011	0.5486	1195	1948	1792	1396	555	1039	1473	1367	405	633
**Rrs1**	-0.3575	4.1072	4.3402	0.0372	0.8131	118	160	126	85	43	59	95	98	45	75
**Acp1**	-0.3559	5.671	4.8608	0.0275	0.7469	227	268	281	214	113	122	261	198	74	155
**Sertad4**	-0.355	4.7986	4.2338	0.0396	0.8235	108	263	236	145	113	93	153	159	60	155
**Xab2**	-0.3531	4.5639	5.3359	0.0209	0.6908	141	196	227	146	73	106	169	137	39	119

**Mysm1**	-0.3531	5.4299	5.5776	0.0182	0.6499	239	320	296	255	122	208	146	216	100	188
**Ccdc9**	-0.3528	4.337	3.9843	0.0459	0.8611	102	173	164	159	65	92	142	97	45	103
**BC003331**	-0.3493	6.3797	10.076	0.0015	0.2478	224	368	312	220	136	180	204	188	95	228
**Lemd2**	-0.346	5.6838	4.6645	0.0308	0.772	307	374	422	387	142	286	366	187	138	185
**Diaph3**	-0.3427	4.9892	4.8271	0.028	0.7488	183	198	258	208	146	101	177	176	95	182
**Pccb**	-0.3421	5.2652	4.3488	0.037	0.8131	128	224	187	145	62	104	115	160	35	122
**Dcaf17**	-0.3419	4.9431	5.6294	0.0177	0.6405	176	269	215	179	117	128	174	186	81	122
**Prkacb**	-0.3418	7.6432	6.716	0.0096	0.525	268	376	273	296	119	213	252	229	92	176
**Skiv2l**	-0.3413	5.5189	3.9525	0.0468	0.8678	144	358	326	231	106	176	165	199	104	148
**Tnks1bp1**	-0.3412	6.4879	8.1617	0.0043	0.3886	509	841	805	552	328	413	505	504	264	449
**Dnm3**	-0.3355	7.68	4.5999	0.032	0.7798	64	118	116	81	53	44	119	67	42	44
**Kmt2a**	-0.3348	7.9799	9.2597	0.0023	0.3121	1278	2421	1971	1523	680	1068	1728	1458	473	988
**Ccdc34**	-0.3309	6.0685	4.3116	0.0379	0.8131	506	736	496	422	244	495	348	424	189	242
**Pgp**	-0.3305	6.0986	3.8606	0.0494	0.8722	127	168	157	125	39	103	117	87	49	80
**Cntln**	-0.3299	5.9954	5.5895	0.0181	0.6487	220	339	255	220	131	197	188	194	92	169
**Lnp**	-0.3269	5.794	5.2753	0.0216	0.6958	80	163	128	84	88	80	138	78	36	76
**Slc25a12**	-0.3266	6.6984	3.9043	0.0482	0.8678	164	239	202	162	143	161	192	119	81	126
**Stoml1**	-0.3262	5.6	4.0317	0.0447	0.8574	63	117	110	73	48	45	105	54	29	71
**Tbc1d10a**	-0.326	5.5786	4.705	0.0301	0.7694	380	569	593	387	190	296	451	384	147	260
**Atf4**	-0.3244	8.1402	7.6267	0.0058	0.4358	1472	2560	2537	1486	962	1497	1547	1371	789	1122
**Dip2c**	-0.324	5.7925	5.0025	0.0253	0.7267	120	221	186	147	59	82	150	124	42	137
**Zfp503**	-0.3237	4.6608	4.2382	0.0395	0.823	91	160	170	88	67	71	111	124	32	87
**Nop14**	-0.3235	5.4703	5.2352	0.0221	0.6995	225	326	297	218	101	147	260	180	113	133
**Upf1**	-0.3234	5.0053	5.2116	0.0224	0.6995	191	298	287	186	106	122	227	190	89	146
**Rfc2**	-0.3229	4.904	4.94	0.0262	0.7359	172	204	274	181	97	137	218	155	41	153
**Lrch3**	-0.3226	5.459	4.9068	0.0268	0.7377	118	201	152	125	88	115	148	98	52	93
**Cxxc1**	-0.3224	5.2859	5.6626	0.0173	0.6381	202	381	317	261	132	166	298	219	113	142
**Srpr**	-0.3216	5.4973	5.0852	0.0241	0.7184	190	358	311	256	126	195	205	206	105	174
**Phf20**	-0.3215	5.4813	4.2377	0.0395	0.823	86	92	71	63	51	52	66	60	24	70
**Vamp2**	-0.3195	7.7893	4.7712	0.0289	0.7557	166	325	322	231	137	164	285	213	99	107
**Mettl16**	-0.3176	5.8077	5.4696	0.0194	0.6697	256	359	407	305	184	196	388	209	141	189
**Pelp1**	-0.3155	5.2818	3.8744	0.049	0.8705	186	324	250	201	81	169	256	125	75	132
**Prkcb**	-0.3139	6.7818	3.9448	0.047	0.8678	233	490	297	247	217	276	236	317	84	206
**Atl3**	-0.3133	5.2848	4.6147	0.0317	0.7768	200	284	221	206	168	148	174	181	95	212
**Mtcl1**	-0.3125	5.5706	3.8489	0.0498	0.8736	136	164	128	99	60	116	111	75	35	99
**Ttc28**	-0.3121	6.1717	6.0577	0.0138	0.6019	347	566	572	510	215	415	426	353	137	303
**Hmg20a**	-0.3106	5.4107	6.9575	0.0083	0.5048	163	235	225	218	133	129	196	181	88	136
**Zfp768**	-0.3099	4.516	4.3235	0.0376	0.8131	158	176	198	188	100	137	145	158	69	103
**Cnot11**	-0.3091	4.735	4.4525	0.0349	0.8037	155	158	195	138	86	126	117	105	62	130
**Pdcd4**	-0.3086	7.1148	3.9814	0.046	0.8611	1009	1659	1448	930	835	1010	1242	1011	512	605
**Git1**	-0.3081	5.7774	3.8668	0.0493	0.8715	202	273	345	214	104	171	231	199	73	167
**Fam192a**	-0.3045	5.225	6.7381	0.0094	0.5248	182	242	256	187	118	137	191	167	84	154
**Crybg3**	-0.3041	6.5126	6.7699	0.0093	0.5211	639	1016	959	691	488	527	806	600	363	504
**Aff1**	-0.3022	5.216	6.9049	0.0086	0.5053	235	320	363	276	191	230	298	231	97	202
**Rexo1**	-0.2994	5.3469	4.1199	0.0424	0.8429	180	330	247	315	87	145	280	197	71	180
**Zcchc2**	-0.299	4.821	3.9098	0.048	0.8678	141	194	272	160	92	101	164	135	84	143
**Ppfia3**	-0.2966	5.5089	5.0558	0.0245	0.7214	87	146	144	123	66	91	130	98	37	72
**H2afj**	-0.2965	5.9631	5.7059	0.0169	0.6337	371	505	418	317	235	303	396	242	122	351
**BC005561**	-0.2958	5.9173	4.1898	0.0407	0.8329	278	553	521	379	177	208	523	372	112	240
**Golim4**	-0.2956	6.7674	5.3605	0.0206	0.6881	503	537	805	531	297	427	617	382	225	359
**Nol11**	-0.2956	4.7925	4.1921	0.0406	0.8329	185	208	198	176	104	131	187	146	64	138
**Pam16**	-0.2938	4.819	3.9163	0.0478	0.8678	82	115	86	95	65	62	87	86	33	71
**Mrpl10**	-0.2934	5.2919	5.0072	0.0252	0.7253	173	247	232	241	116	173	209	162	59	171
**Las1l**	-0.2927	6.1771	5.1514	0.0232	0.7084	447	762	601	497	275	439	435	421	177	445
**Ahdc1**	-0.2917	6.2221	5.9892	0.0144	0.6045	462	782	739	554	302	470	585	524	215	320
**Dnajc13**	-0.2913	6.1662	5.5984	0.018	0.6481	281	341	339	214	193	173	288	218	132	225
**Sgms1**	-0.2895	5.7814	5.2187	0.0223	0.6995	313	467	480	314	204	327	333	238	113	329
**1110032A0**	-0.2894	5.6171	4.8977	0.0269	0.7397	190	336	308	202	129	193	210	196	66	211
**Atp13a2**	-0.2856	6.1212	5.6911	0.0171	0.6337	190	249	257	175	86	152	230	142	65	134
**Dusp3**	-0.2844	7.0368	5.1898	0.0227	0.7048	141	193	270	170	106	112	214	125	73	146
**Smc6**	-0.284	7.3995	7.5546	0.006	0.4432	872	1256	1149	851	497	890	831	790	333	615
**Golgb1**	-0.2834	7.6954	6.9408	0.0084	0.5048	1123	1746	1715	1276	677	1030	1347	1150	558	759

**Grhl2**	-0.2823	5.4357	6.1383	0.0132	0.5897	457	698	541	451	229	348	528	341	184	383
**Zbtb4**	-0.282	7.0262	6.829	0.009	0.5138	300	528	618	364	195	273	400	398	133	297
**Vps13a**	-0.2809	6.3462	4.2564	0.0391	0.8215	385	690	720	383	343	405	500	389	207	398
**Myo10**	-0.2788	6.0939	6.0199	0.0141	0.604	339	516	542	427	188	268	371	357	170	326
**Bdp1**	-0.2788	6.5252	5.9883	0.0144	0.6045	549	789	731	548	253	492	563	462	233	368
**Evi5**	-0.2777	7.6626	7.9119	0.0049	0.4069	876	1545	1253	1031	539	890	1172	883	363	669
**Ilf3**	-0.276	5.8468	4.818	0.0282	0.7493	324	400	331	303	203	231	376	319	87	233
**Pcyox1**	-0.275	6.1295	4.2373	0.0395	0.823	296	312	325	236	202	223	261	252	105	232
**Myo9a**	-0.272	7.3574	6.1844	0.0129	0.5845	286	309	297	269	160	176	334	231	81	225
**Itsn2**	-0.2709	7.6924	5.7097	0.0169	0.6337	1110	1634	1629	1179	632	1070	1286	1105	502	678
**Setd2**	-0.2704	7.0674	11.793	0.0006	0.1573	804	1255	1189	877	469	694	883	802	375	691
**Cdk13**	-0.2701	6.1109	4.7008	0.0301	0.7699	366	510	545	348	219	306	384	342	163	304
**6430548M**	-0.2663	6.7501	4.7745	0.0289	0.7552	277	351	338	205	105	180	185	244	104	230
**Trpm7**	-0.2649	5.975	3.9926	0.0457	0.8599	268	378	331	299	176	179	324	273	130	215
**Tln2**	-0.2648	7.5552	4.3901	0.0361	0.811	202	386	282	210	102	175	208	207	108	169
**Nelfe**	-0.2647	5.6507	5.0838	0.0242	0.7184	325	393	369	328	170	232	292	237	155	279
**Mga**	-0.2612	6.4561	4.5886	0.0322	0.7798	449	574	621	495	266	338	655	403	195	292
**Ash1l**	-0.261	8.0496	8.47	0.0036	0.3649	1533	2063	1877	1371	785	1171	1614	1406	587	1062
**Zcchc11**	-0.2609	6.3916	4.0719	0.0436	0.85	469	725	750	459	349	440	577	470	257	343
**Epn2**	-0.2608	6.5506	5.0629	0.0244	0.7207	259	427	483	355	265	280	335	289	150	334
**Pdzd11**	-0.26	5.5841	3.8576	0.0495	0.8726	254	270	244	230	116	181	204	209	95	165
**Rasal2**	-0.2593	7.1086	6.1574	0.0131	0.5875	473	690	539	477	233	342	502	508	161	350
**Bmpr1a**	-0.2592	6.1745	4.6156	0.0317	0.7768	438	598	522	503	214	386	476	378	176	323
**Tnpo3**	-0.2589	6.7307	5.0639	0.0244	0.7207	611	928	931	697	340	545	839	622	250	447
**Emsy**	-0.2563	5.8605	4.1643	0.0413	0.8377	257	506	409	296	155	252	359	299	95	243
**Slc38a10**	-0.2554	7.035	5.346	0.0208	0.6899	715	958	836	583	301	518	608	660	226	572
**Bbx**	-0.2541	6.7857	3.9761	0.0461	0.8611	727	931	682	617	325	606	586	465	286	528
**Eif4e**	-0.2537	6.1726	5.4116	0.02	0.6772	269	363	375	249	160	201	334	231	120	209
**Vps37a**	-0.2532	6.0151	4.638	0.0313	0.7726	275	390	413	255	161	221	310	254	98	275
**Numa1**	-0.2527	6.5646	4.0813	0.0434	0.85	620	879	769	620	322	584	624	521	256	455
**Fam133b**	-0.2508	6.6326	4.3138	0.0378	0.8131	526	702	711	552	241	534	556	453	182	370
**Alad**	-0.2508	6.0257	4.8465	0.0277	0.747	442	576	512	422	237	336	570	308	166	342
**Thoc2**	-0.2496	8.2647	5.236	0.0221	0.6995	2219	3648	3092	2494	1235	2139	2852	2372	881	1563
**Birc6**	-0.2484	7.1604	8.3393	0.0039	0.3742	585	917	912	719	387	478	729	659	287	565
**Bod1l**	-0.2483	8.086	4.3157	0.0378	0.8131	1033	1438	1167	1038	548	916	1244	959	372	612
**Them6**	-0.2479	7.4903	4.0539	0.0441	0.8533	286	371	359	300	132	211	298	261	137	192
**Mafb**	-0.2458	6.2144	3.8912	0.0485	0.8678	635	1045	914	655	392	490	722	492	360	667
**Phf14**	-0.2441	7.5004	5.2274	0.0222	0.6995	1276	2018	1957	1430	857	1289	1660	1423	561	949
**Arrdc1**	-0.2437	5.4542	3.9237	0.0476	0.8678	255	472	450	339	158	232	325	352	121	252
**Mycbp2**	-0.2431	8.7575	6.4491	0.0111	0.5501	1963	3120	3003	2205	1151	1777	2563	2107	914	1441
**Micall1**	-0.2417	6.4287	4.2707	0.0388	0.8196	708	949	959	632	347	584	757	643	254	547
**Chd6**	-0.2415	7.0756	4.8546	0.0276	0.7469	625	1027	819	679	368	593	748	629	233	547
**Zfp445**	-0.2391	6.6691	6.2734	0.0123	0.5734	441	718	691	550	291	398	547	487	242	393
**Ankrd11**	-0.2365	8.9267	4.5872	0.0322	0.7798	2696	3930	3692	2473	1442	2499	3196	2784	1038	1566
**Cpt1a**	-0.2358	6.7327	5.0639	0.0244	0.7207	542	736	687	629	287	514	522	465	209	529
**Akap11**	-0.2345	7.0718	3.921	0.0477	0.8678	196	253	265	164	149	143	225	170	105	164
**Dnajc2**	-0.232	6.628	4.0462	0.0443	0.8549	510	752	733	540	296	513	652	469	211	373
**Rsf1**	-0.2307	6.988	4.7856	0.0287	0.7541	735	961	862	744	364	642	808	657	270	508
**Ptprd**	-0.229	6.2796	3.9127	0.0479	0.8678	376	688	607	474	295	373	552	404	157	465
**Ppfibp1**	-0.2274	6.6585	4.1255	0.0422	0.8429	805	1030	874	662	558	609	664	646	417	716
**Dnmt3a**	-0.2271	5.9027	3.9596	0.0466	0.8665	331	421	451	325	193	300	365	337	121	243
**Cyfip2**	-0.2211	7.3939	4.3817	0.0363	0.811	480	902	765	554	307	465	585	564	265	427
**Dnttip2**	-0.2208	5.9694	4.4141	0.0356	0.811	369	526	466	318	214	283	347	382	160	307
**Prdm2**	-0.2196	6.7429	4.4897	0.0341	0.7976	588	747	783	523	321	504	731	517	203	425
**Ino80d**	-0.2195	5.9209	4.1205	0.0424	0.8429	360	586	517	467	226	339	461	448	168	286
**Frg1**	-0.2194	7.2484	4.0575	0.044	0.8533	1330	1776	1528	1101	602	1231	1305	1067	460	893
**Wdfy2**	-0.2175	5.9822	3.8771	0.0489	0.8696	157	250	222	179	120	140	189	151	99	149
**Arid4a**	-0.2173	7.2888	3.8893	0.0486	0.8678	1069	1608	1479	1138	539	1058	1125	1183	386	825
**Synj1**	-0.2161	7.745	4.2533	0.0392	0.8222	286	304	347	228	172	216	310	269	101	186
**Dgkh**	-0.2157	8.8139	4.6557	0.031	0.7726	262	380	286	253	168	194	343	219	115	213
**Ncor1**	-0.2153	8.8018	4.9966	0.0254	0.7274	2433	3763	3485	2444	1554	2347	2930	2575	1162	1724
**Tmcc1**	-0.2148	5.6158	4.1894	0.0407	0.8329	237	294	282	229	109	184	235	215	86	191

**Utrn**	-0.2144	8.8286	4.3549	0.0369	0.8131	1648	2483	2278	1826	969	1488	2016	1775	755	1224
**Jund**	-0.2134	7.7794	4.8022	0.0284	0.7519	1156	1564	1700	1024	602	930	1288	1046	530	904
**Polr3g**	-0.213	6.6272	3.9577	0.0467	0.8665	477	585	552	473	276	427	444	428	186	400
**U2surp**	-0.2127	7.3757	4.4189	0.0355	0.811	867	1296	1245	964	509	787	1113	919	405	631
**Prrc2c**	-0.2109	9.0359	4.2105	0.0402	0.8291	3089	4804	4319	3142	1595	2898	3825	3197	1301	2080
**Acbd5**	-0.2098	6.3302	4.3161	0.0378	0.8131	238	385	333	264	146	206	309	243	101	230
**Nfib**	-0.2093	7.3966	4.7805	0.0288	0.7541	844	1177	1076	835	436	735	985	762	297	722
**Pcm1**	-0.2084	7.8335	4.9841	0.0256	0.7316	1069	1529	1516	1126	760	1032	1364	1220	471	772
**Gls**	-0.1996	7.8291	5.3955	0.0202	0.6801	1112	2024	1509	1270	736	1037	1546	1147	506	1109
**Zbtb20**	-0.1964	8.7922	4.4402	0.0351	0.8064	1467	1935	2040	1314	725	1124	1673	1411	586	1176
**Aktip**	-0.196	6.9541	3.891	0.0485	0.8678	465	716	675	527	285	414	639	426	210	461
**Zdhhc20**	-0.1867	6.4771	4.1092	0.0427	0.8447	563	938	754	610	326	478	690	565	277	534
**Wbp11**	-0.1865	6.4727	3.9356	0.0473	0.8678	469	733	635	471	258	408	576	454	192	438
**Hnrnpul2**	-0.1812	6.9233	4.0746	0.0435	0.85	773	1085	1034	818	460	661	978	777	310	702
**Huwe1**	-0.1753	8.4917	4.0139	0.0451	0.8577	1767	2761	2576	1973	1185	1553	2611	1964	849	1481
**Mapre2**	-0.1727	7.747	3.9263	0.0475	0.8678	405	694	592	458	284	396	463	454	238	397
**Nfe2l2**	-0.169	6.7678	3.8876	0.0486	0.8681	646	980	891	663	452	617	853	645	269	639
**Ube3a**	-0.1657	7.6096	4.2559	0.0391	0.8215	698	1071	1024	762	387	628	917	711	318	639

Table S3. Differentially expressed genes in the cornea of miR-183C KO vs age- and sex-matched WT control mice.

?	log2FC	logCPM	LR	PValue	FDR	WT1	WT2	WT3	KO1	KO2	KO3
**Upregulated genes**											
Gbp1	10.09237	2.002584	23.22829	1.43865E-06	0.000755	0	0	0	0	235	102
Sprr2f	8.356159	0.048928	17.44497	2.95745E-05	0.006917	0	0	0	1	63	33
Ccl8	8.26459	0.444292	15.75058	7.2266E-05	0.012641	0	0	0	4	14	49
Saa3	7.973225	4.381648	19.12251	1.22591E-05	0.003598	11	2	0	1	512	1115
2310002L1	7.717483	0.938009	33.98515	5.55342E-09	9.19E-06	1	0	0	18	85	75
Sprr2d	6.861787	-1.21987	5.252055	0.021920874	0.493874	0	0	0	0	9	18
Pcdhb10	6.639641	-0.58049	10.2572	0.001361524	0.095112	0	0	0	6	0	14
Cxcr2	6.602779	-1.28345	5.182495	0.022815538	0.50504	0	0	0	7	1	12
Ccl5	6.443421	0.20881	15.08569	0.000102739	0.016621	0	0	0	7	0	11
4933422H2	6.379781	-0.70556	9.563529	0.001984812	0.12307	0	0	0	1	13	9
Fgr	6.369988	-1.12429	6.597586	0.010211714	0.320752	0	0	0	0	6	13
Hist1h3c	6.367877	-1.13506	4.237244	0.039546303	0.656542	0	0	0	0	8	12
Ncf4	6.3279	-0.53097	12.53011	0.000400446	0.043515	0	0	0	10	6	4
Pou6f2	6.3131	0.229875	11.69114	0.000627984	0.059003	0	0	0	6	21	0
7530420F2	6.304496	-1.51512	4.356335	0.036871549	0.6367	0	0	0	6	4	8
Lrg1	6.175243	0.018776	9.700806	0.001841872	0.117946	0	0	0	0	0	14
Sgk2	6.157538	-0.09919	13.61441	0.000224457	0.028746	0	0	0	0	16	6
Hhex	6.119511	0.066129	9.181364	0.002444917	0.139459	0	0	0	12	0	3
Mak	5.995725	-0.76892	6.099519	0.013521871	0.376391	0	0	0	6	0	7
Chi3l1	5.94974	2.384754	35.84776	2.13353E-09	4.45E-06	6	5	0	15	120	239
Wdr64	5.93475	-1.50541	3.863045	0.049360676	0.721988	0	0	0	3	0	9
Grik1	5.924846	6.098558	29.54174	5.47255E-08	5.89E-05	0	0	0	11	0	5
Cxcr6	5.92031	-0.59604	6.874043	0.008745657	0.295349	0	0	0	5	9	3
Prkag3	5.750946	-0.42192	7.881845	0.004993352	0.218288	0	0	0	3	16	0
Rgs9bp	5.749296	-0.70573	10.18337	0.001417129	0.09727	0	0	0	8	5	1
Mx1	5.746517	-1.04078	6.739835	0.009428332	0.307123	0	0	0	4	8	3
Rwdd2a	5.720253	1.728077	20.74556	5.24529E-06	0.00182	0	0	0	0	13	3
Saa2	5.70446	-1.28416	5.290356	0.021443831	0.489181	0	0	0	0	2	9
Uprt	5.687273	-1.37855	4.577399	0.032396354	0.598041	0	0	0	5	1	5
Esam	5.675584	3.650066	23.86966	1.03084E-06	0.000616	0	0	0	5	7	2
Ppp1r16b	5.653209	3.421284	23.38692	1.32476E-06	0.00075	0	0	0	6	8	0
Rlbp1	5.533137	1.700564	16.98991	3.75791E-05	0.008194	0	0	0	6	4	2
A230057D0	5.475982	-0.73309	7.097883	0.007717505	0.277675	0	0	0	3	0	6
Pex5l	5.44122	5.475629	21.7388	3.12408E-06	0.001293	0	0	0	0	9	3
Zdbf2	5.393512	2.411139	16.29221	5.42867E-05	0.010069	0	0	0	8	0	2
Myrip	5.382911	1.506843	15.27673	9.28533E-05	0.015564	0	0	0	0	11	2
Zfp389	5.370773	-0.24856	10.37877	0.001274729	0.090518	0	0	0	0	8	4
Ak5	5.360171	3.171656	19.10449	1.23753E-05	0.003598	0	0	0	6	4	1
4932438H2	5.346251	-1.00422	3.980559	0.046028291	0.703111	0	0	0	0	10	3
Pacrg	5.343496	2.252178	16.99275	3.75227E-05	0.008194	0	0	0	0	13	0
Fbxl2	5.269457	3.691976	18.7582	1.48384E-05	0.004041	0	0	0	1	10	0
Pla2g4e	5.220657	0.702564	9.505974	0.002048041	0.125775	0	0	0	0	1	7
Edil3	5.202587	5.849651	18.45536	1.73931E-05	0.004564	0	0	0	4	5	1
Npy1r	5.186344	2.420129	38.38609	5.80445E-10	1.39E-06	0	0	1	6	14	12
U46068	5.183956	-0.52745	7.211901	0.007242176	0.266447	0	0	0	0	10	2
Slamf7	5.180348	-0.95919	3.869129	0.049182027	0.720347	0	0	0	1	0	6
1810033B1	5.175067	-1.12769	4.325194	0.037552136	0.642723	0	0	0	1	0	6
L3mbtl	5.163921	-0.07966	8.11534	0.004389225	0.201764	0	0	0	8	0	0
Gm765	5.15392	4.301888	16.57436	4.67792E-05	0.009234	0	0	0	0	0	9
Mfi2	5.151305	-0.81799	5.606715	0.017891778	0.446595	0	0	0	5	5	0
Dio2	5.143224	0.415158	10.96389	0.000929047	0.075755	0	0	0	2	6	2
6430531B1	5.099276	-0.02104	7.255065	0.007070131	0.26273	0	0	0	0	13	0
Fgf7	5.084813	1.817515	13.65473	0.000219687	0.028386	0	0	0	0	11	0
Tagln3	5.081802	6.733713	16.91874	3.90145E-05	0.00823	0	0	0	0	0	9
Dpf3	5.068098	0.405608	9.948573	0.001609742	0.105918	0	0	0	0	12	0
Lrrc2	5.053625	-0.65481	4.990024	0.025493856	0.530489	0	0	0	0	13	0
Amhr2	5.03855	0.179765	9.736196	0.001806744	0.116389	0	0	0	0	10	1
Ubxn10	5.037601	-1.10802	4.09973	0.042890058	0.684586	0	0	0	0	13	0
Krt27	5.02839	1.17352	11.87111	0.000570114	0.054518	0	0	0	0	11	0
Snap91	5.023318	5.80678	16.32951	5.32285E-05	0.009983	0	0	0	0	9	0
Atp6v1c2	4.983194	0.258496	6.564602	0.010402698	0.323446	0	0	0	7	0	0
Rgs6	4.978817	-0.48996	6.960726	0.00833182	0.287118	0	0	0	7	0	0

Gjb1	4.974874	4.454483	15.26733	9.33167E-05	0.015564	0	0	0	0	9	0
Pgf	4.959289	2.465709	14.39961	0.000147833	0.021936	0	0	0	0	8	1
Mmp3	4.956893	2.454486	10.97496	0.000923514	0.075755	2	16	7	0	98	330
Gp49a	4.956206	-0.65893	5.246126	0.021995688	0.494008	0	0	0	0	10	1
4921523A1	4.934342	-0.89314	4.239155	0.039501827	0.656377	0	0	0	0	12	0
Gbp5	4.916291	1.520429	12.08698	0.000507752	0.050813	0	0	0	2	7	0
Rasgef1a	4.911904	6.019029	14.98281	0.000108495	0.017292	0	0	0	8	0	0
Nrn1	4.882861	7.773578	14.87916	0.000114622	0.018134	0	0	0	2	6	0
Cdh5	4.863886	2.841098	12.57166	0.000391641	0.04294	0	0	0	0	0	7
Nxph3	4.861682	1.69664	12.61465	0.000382735	0.04223	0	0	0	0	9	0
Tmem59l	4.860239	5.14902	14.5423	0.000137048	0.020707	0	0	0	0	8	0
Gucy2e	4.838408	-0.73669	5.724256	0.016732127	0.429604	0	0	0	0	5	3
Flt4	4.82291	-0.03489	8.605733	0.003351064	0.17208	0	0	0	0	10	0
Kcnmb3	4.810606	-0.87717	4.775012	0.028875648	0.563783	0	0	0	5	0	1
Tnni3	4.80408	0.163007	12.20915	0.000475556	0.048493	0	1	0	12	16	0
Slc6a1	4.798541	2.773073	12.78164	0.000350037	0.039849	0	0	0	3	0	4
Aim2	4.782379	-0.00985	8.108179	0.004406598	0.201764	0	0	0	1	3	3
Hcrtr1	4.769603	-0.18528	7.408759	0.006490707	0.250929	0	0	0	3	5	0
Gimap5	4.767531	-0.48243	5.710275	0.016865896	0.431494	0	0	0	6	0	0
Fam163b	4.756185	0.798073	9.935269	0.001621418	0.106361	0	0	0	0	9	0
Rnf165	4.751772	2.151821	12.37312	0.00043556	0.045661	0	0	0	0	8	0
Samsn1	4.747829	1.704378	11.5543	0.000675926	0.062151	0	0	0	0	3	4
Fibcd1	4.741054	-0.15127	7.37113	0.006627926	0.2542	0	0	0	6	0	0
BC020535	4.739941	0.219003	7.425428	0.006430852	0.250929	0	0	0	6	0	0
Papolb	4.722821	0.458588	9.042229	0.002638138	0.148592	0	0	0	0	9	0
Pde6b	4.707457	-1.00613	4.348295	0.037046012	0.637156	0	0	0	0	0	5
AW551984	4.696666	-0.71316	4.776814	0.028845448	0.563705	0	0	0	0	0	5
Nat8	4.674652	-1.34499	4.451972	0.034860966	0.620918	0	0	0	5	1	0
Eya1	4.670104	1.593564	10.61138	0.001123938	0.083102	0	0	0	0	8	0
Ncam2	4.641328	4.10654	40.89947	1.60264E-10	4.31E-07	2	0	0	0	30	11
Plvap	4.635919	2.097824	10.15161	0.001441751	0.098167	0	0	0	6	0	0
Il10ra	4.632594	0.847234	19.91836	8.08203E-06	0.002596	0	0	2	7	13	15
Krt16	4.62214	6.024555	25.45291	4.53312E-07	0.000287	301	72	29	104	2852	2599
BC026585	4.610732	-0.71723	5.805667	0.015974606	0.416022	0	0	0	0	9	0
Moxd1	4.604183	3.01317	28.74754	8.24546E-08	8.06E-05	1	0	0	6	16	1
Ctla2b	4.582871	-0.51044	6.797502	0.009128551	0.302732	0	0	0	0	5	2
Adora2a	4.581537	2.201736	11.10543	0.000860752	0.072627	0	0	0	0	7	0
Shank3	4.568202	0.632208	7.660052	0.005645688	0.232403	0	0	0	0	0	5
Slc17a8	4.566284	1.77818	10.8273	0.001000145	0.077407	0	0	0	0	7	0
Lilra5	4.557779	-0.27213	6.498984	0.010793615	0.330635	0	0	0	4	2	0
Galnt6	4.538393	1.357054	9.177513	0.002450068	0.139459	0	0	0	4	2	0
Myt1l	4.504685	6.609825	11.28124	0.000782943	0.068758	0	0	0	6	0	0
Nrxn3	4.501898	6.083184	11.24136	0.000799946	0.069123	0	0	0	5	1	0
Elavl4	4.501532	8.809614	11.29743	0.000776144	0.068441	0	0	0	6	0	0
Syt2	4.482555	6.975247	11.16683	0.000832729	0.071099	0	0	0	1	5	0
Ltc4s	4.467936	0.174699	6.893584	0.008650565	0.29395	0	0	0	5	0	0
Golga7b	4.461519	4.738399	10.90931	0.000956819	0.075755	0	0	0	0	6	0
Cacna1e	4.447176	0.660783	7.43869	0.006383633	0.250156	0	0	0	5	0	0
Kcnh7	4.444156	2.97639	10.37948	0.001274234	0.090518	0	0	0	0	5	1
Kcnn2	4.40959	1.000976	8.134926	0.004342062	0.201618	0	0	0	5	0	0
Dgkk	4.398428	2.509731	9.978912	0.001583432	0.105305	0	0	0	0	6	0
Syt15	4.379065	0.051903	7.13211	0.007571556	0.273798	0	0	0	0	7	0
Trim15	4.364975	-0.76866	5.649715	0.017458208	0.439849	1	0	0	11	6	0
She	4.355982	2.147845	9.322478	0.002263597	0.13454	0	0	0	0	6	0
Tub	4.351791	-0.28171	6.31507	0.011971602	0.348553	0	0	0	0	2	3
Hs6st3	4.343562	-1.07904	4.948584	0.026112041	0.537119	0	0	0	0	0	4
Camk1g	4.277419	4.200058	9.378327	0.002195658	0.131573	0	0	0	0	0	5
Ap3b2	4.253686	7.303381	9.429588	0.002135123	0.129043	0	0	0	0	0	5
Cyb5r2	4.241351	0.352231	6.337309	0.011822408	0.345613	0	0	0	0	0	4
Dhh	4.236613	5.000854	9.265028	0.002335709	0.135357	0	0	0	2	3	0
3110047P2	4.225653	5.464965	9.261468	0.002340254	0.135357	0	0	0	0	5	0
Adam5	4.224937	-0.70408	5.140363	0.023375754	0.512172	0	0	0	1	5	0
Cd300a	4.21381	-0.40745	5.168377	0.023001691	0.506052	0	0	0	3	0	1
Adamts8	4.201214	0.657948	15.67819	7.5085E-05	0.012993	0	2	0	5	13	10
Gm5820	4.19983	-0.61051	4.286623	0.038413381	0.646227	0	0	0	4	0	0

C130060K2	4.199736	-0.50246	4.290893	0.038317039	0.646227	0	0	0	4	0	0
Impg2	4.198973	-0.97926	4.325085	0.037554542	0.642723	0	0	0	4	0	0
Cd38	4.195857	0.626359	6.690796	0.009691192	0.311217	0	0	0	0	0	4
Eltd1	4.181824	3.087958	8.734448	0.003122538	0.165982	0	0	0	0	5	0
Pde1a	4.168494	3.233693	19.22397	1.16244E-05	0.003474	0	0	1	4	3	8
Ccdc85a	4.166248	3.167227	8.54149	0.003471414	0.176158	0	0	0	0	5	0
Nppa	4.149743	0.347473	5.810464	0.015931092	0.416022	0	0	0	4	0	0
Tmprss5	4.136663	2.45455	8.167062	0.004265796	0.199528	0	0	0	0	5	0
Col2a1	4.136177	0.239291	6.064741	0.013790701	0.3809	0	0	0	4	0	0
Rasal3	4.128793	-0.76602	3.977593	0.046109422	0.703111	0	0	0	2	3	0
Scml4	4.099487	1.131376	6.578674	0.010320774	0.323011	0	0	0	4	0	0
Fam163a	4.071937	-0.85019	5.313843	0.021156633	0.485812	0	0	0	2	1	1
4833422C1	4.056722	-0.69996	3.948056	0.046925536	0.705555	0	0	0	0	6	0
Actn3	4.031662	-0.28593	5.108281	0.023811898	0.516307	1	0	0	0	0	10
Tlr13	4.021227	0.14316	8.806814	0.003001076	0.162648	1	0	0	0	12	4
Inha	4.019217	1.723893	25.83695	3.71507E-07	0.000266	3	0	0	8	24	5
Rab39b	3.999246	5.323002	41.26948	1.32623E-10	4.08E-07	1	0	2	13	13	8
Ly6c2	3.99047	-0.63518	4.148326	0.041675712	0.675222	0	0	0	0	2	2
Slc6a13	3.979289	3.775231	7.414768	0.006469067	0.250929	0	0	0	4	0	0
Kcnma1	3.97421	4.275073	7.435603	0.006394593	0.250156	0	0	0	4	0	0
Paqr9	3.955466	4.606858	7.506595	0.006147348	0.246571	0	0	0	4	0	0
BC048546	3.947026	6.903502	7.54008	0.006034137	0.243584	0	0	0	3	0	1
Akap6	3.938452	6.908552	7.497964	0.006176878	0.246571	0	0	0	2	2	0
Cntn2	3.930616	7.40431	7.483613	0.006226301	0.246811	0	0	0	0	4	0
Cadps	3.930336	6.727857	7.481007	0.006235318	0.246811	0	0	0	0	4	0
Nipal4	3.923638	-0.33212	5.170365	0.022975391	0.506052	0	0	0	0	5	0
Bcan	3.917883	5.542172	7.364033	0.006654137	0.254404	0	0	0	0	4	0
Prss12	3.910236	4.114798	7.291381	0.006928619	0.259331	0	0	0	0	4	0
Ncan	3.906468	3.823312	7.255356	0.007068987	0.26273	0	0	0	0	4	0
Nkx2-9	3.89396	0.024651	4.706026	0.030057057	0.575312	0	0	0	0	5	0
Prox1	3.884541	0.204999	4.555503	0.032813132	0.60291	0	0	0	0	5	0
Atp8a2	3.881466	3.365143	7.012544	0.008094057	0.283674	0	0	0	0	4	0
Obp1a	3.867969	-0.09074	4.649926	0.031054886	0.584071	0	0	0	0	0	3
4732456N1	3.836686	3.247104	21.59004	3.37601E-06	0.001369	42	34	15	20	652	255
Fpr2	3.836158	0.438738	11.05988	0.000882157	0.073854	0	2	0	11	11	1
1110020A2	3.829742	0.063903	8.94636	0.00278023	0.153615	0	0	2	3	22	2
Gpr183	3.822547	0.285883	9.289623	0.002304556	0.135109	0	0	3	6	18	8
P2rx2	3.797508	0.142551	5.075826	0.024261714	0.519935	0	0	0	0	0	3
Grm3	3.794554	1.220585	6.119214	0.013372028	0.374139	0	0	0	0	4	0
Hoxd8	3.790268	1.441202	10.36403	0.001284947	0.090944	1	0	0	1	14	0
Htr1f	3.788195	-0.25295	3.87519	0.04900475	0.720347	0	0	0	3	0	0
Pmfbp1	3.752168	0.822467	10.59408	0.001134506	0.083596	0	1	0	0	9	4
Itgb2	3.746522	0.534936	7.931561	0.004857986	0.215071	0	4	0	11	23	8
Kcnh5	3.740489	0.867971	5.526014	0.018735736	0.454474	0	0	0	0	4	0
Trhde	3.738409	-0.13778	4.68722	0.030387769	0.578093	0	0	0	3	0	0
Gpr22	3.732446	2.81456	13.90779	0.000192001	0.025927	1	0	0	0	12	1
Ankrd34a	3.718407	0.832146	5.367728	0.020512674	0.479255	0	0	0	0	0	3
Pvt1	3.695138	0.934873	5.082113	0.024173894	0.51857	0	0	0	3	0	0
Sema6b	3.69273	1.18887	5.435913	0.019726776	0.468392	0	0	0	0	0	3
Fgd5	3.67361	1.133594	5.222361	0.022298255	0.497686	0	0	0	3	0	0
Unc5d	3.648659	1.117715	5.354173	0.020672732	0.481091	0	0	0	3	0	0
C2cd4c	3.647716	1.693491	5.358628	0.020619993	0.480976	0	0	0	3	0	0
Pnpla1	3.647536	-0.63421	4.786296	0.028687046	0.562141	0	4	1	9	19	16
Aard	3.642018	1.754156	5.544173	0.01854232	0.451819	0	0	0	0	0	3
Gm98	3.641385	2.208234	5.545337	0.018529992	0.451819	0	0	0	0	0	3
Ica1l	3.625299	1.598758	5.455043	0.01951192	0.466714	0	0	0	3	0	0
Apba2	3.56945	4.596525	5.635201	0.017603332	0.441171	0	0	0	3	0	0
Cmtm5	3.56552	5.41229	5.645432	0.017500905	0.440226	0	0	0	3	0	0
Slc17a7	3.565249	5.470233	5.659469	0.017361378	0.439468	0	0	0	0	0	3
Bet3l	3.563772	5.667491	5.649899	0.017456374	0.439849	0	0	0	3	0	0
Scn11a	3.546935	6.851326	5.615061	0.017806766	0.4455	0	0	0	0	3	0
Hs3st2	3.539308	4.147418	5.561457	0.018360126	0.449928	0	0	0	0	3	0
Sorcs1	3.538219	4.33729	5.55376	0.018441031	0.450883	0	0	0	0	3	0
Aldh1a2	3.533186	2.194895	5.343506	0.020799596	0.482246	0	0	0	1	2	0
Gabrg1	3.530769	4.134488	5.500839	0.019007352	0.458965	0	0	0	0	3	0

Dusp15	3.529755	5.161516	5.493596	0.019086242	0.459461	0	0	0	0	3	0
Was	3.525714	0.206891	5.78444	0.016168652	0.419417	0	1	0	1	0	7
Ttll11	3.504196	2.776172	21.7714	3.07144E-06	0.001293	3	0	0	8	18	0
Casp4	3.502366	-0.41485	5.958171	0.014649227	0.396469	1	0	0	0	6	4
2900041M	3.477399	-0.22777	5.508909	0.018919846	0.457907	0	1	0	3	9	0
Rac2	3.475242	0.9351	8.052812	0.004543309	0.207106	0	5	0	2	3	26
Crybb1	3.454653	1.182403	4.933082	0.026347286	0.540924	0	0	0	0	3	0
Pcdhb3	3.404226	0.66558	4.530468	0.033296502	0.606098	0	0	0	0	3	0
Pitx1	3.365286	0.517786	8.51457	0.003523141	0.176699	12	2	3	28	101	3
Nr3c2	3.360894	-0.14605	4.167826	0.041198623	0.670522	0	0	0	0	3	0
Hnf1a	3.353097	0.847314	9.123117	0.002523995	0.142911	4	0	0	2	11	12
Ptpn7	3.295345	-0.11755	4.253413	0.039171534	0.654359	3	1	0	5	21	4
Itgal	3.289982	-0.33098	4.060157	0.043906419	0.690382	1	0	0	0	4	4
Kirrel3	3.277683	2.928015	9.225713	0.002386397	0.136922	1	0	0	8	0	0
Derl3	3.274275	-0.67328	4.178407	0.040942156	0.668622	0	3	0	6	14	4
Oas2	3.264355	1.374948	12.6424	0.000377096	0.041823	28	5	3	16	150	59
Sntg1	3.255325	1.743573	9.925329	0.001630197	0.106612	0	2	0	0	6	8
Timp1	3.232747	1.256663	10.24299	0.001372047	0.095388	16	0	8	9	13	80
Hist2h2bb	3.225836	-0.772	3.908	0.048056763	0.717052	0	0	3	3	6	9
Pnpla3	3.215427	3.752492	18.93694	1.35111E-05	0.003727	0	3	4	26	2	9
Lyz1	3.179529	0.379022	5.282386	0.021542202	0.489635	6	0	0	0	5	21
Arhgap9	3.16851	-0.0503	6.760034	0.009322197	0.304924	3	0	0	6	2	7
4930432K2	3.157999	1.255494	9.953147	0.001605747	0.105918	2	0	0	8	5	0
Gm4980	3.145439	5.008337	9.271757	0.002327143	0.135357	1	0	0	0	8	0
Sync	3.132256	1.04342	8.479604	0.003591503	0.178464	0	3	0	0	5	12
Rundc3b	3.107325	3.224422	8.606602	0.003349467	0.17208	1	0	0	0	8	0
Hck	3.071445	-0.30725	7.99398	0.004693312	0.209834	0	3	1	7	11	6
Tmem90b	3.037752	1.629205	9.292572	0.002300849	0.135109	0	2	0	0	8	5
Grap	3.03353	-0.09088	4.790096	0.028623827	0.561925	1	0	0	0	9	0
Colq	3.031425	1.10494	8.727528	0.003134411	0.165982	0	3	0	4	4	8
S100a8	3.030706	2.046719	12.04276	0.000519939	0.0514	23	6	17	16	84	108
Tmem22	3.026875	2.200248	16.32486	5.33594E-05	0.009983	1	4	0	9	5	11
A3galt2	2.990181	4.257566	7.901211	0.004940171	0.217368	0	0	1	0	4	3
Ctla2a	2.986711	2.539001	12.64797	0.000375974	0.041823	3	0	0	1	10	6
Sprr1a	2.984576	4.685653	29.8383	4.69622E-08	5.32E-05	203	122	102	121	1488	673
Serpine2	2.956943	6.312294	12.46669	0.000414273	0.044126	2	0	0	0	12	0
Abcc9	2.955067	1.054783	8.524164	0.003504617	0.176394	1	0	3	9	0	8
Spats1	2.948718	0.533044	7.079821	0.007795678	0.279088	3	1	0	0	10	10
Abcg4	2.944195	4.048814	20.76148	5.20188E-06	0.00182	0	5	0	4	7	14
1110032A(	2.941427	0.183104	7.657165	0.00565473	0.232403	4	0	0	7	5	6
Penk	2.928307	1.055963	7.310481	0.006855359	0.258772	3	0	0	0	12	5
Cd36	2.907979	1.123442	8.196415	0.004197323	0.196971	5	3	0	0	16	20
St18	2.899518	0.84975	5.434308	0.019744916	0.468392	2	0	0	4	8	0
Fut9	2.884179	1.445702	19.60133	9.54028E-06	0.002932	14	4	7	11	78	34
Lyl1	2.870013	-0.19419	4.100125	0.042880045	0.684586	5	0	0	2	13	8
Myo1a	2.867678	5.569114	28.25851	1.06147E-07	9.52E-05	0	6	1	0	35	3
4930511M	2.856395	-0.52426	4.245099	0.039363775	0.655125	2	0	0	0	5	5
Selplg	2.851698	0.80997	8.108992	0.004404621	0.201764	0	3	0	6	10	1
Tifab	2.818701	0.880844	6.224665	0.012598273	0.359026	4	0	0	0	16	5
S100b	2.80236	10.6812	14.36232	0.00015079	0.022222	3	0	0	2	9	4
Gm12250	2.799604	0.134653	4.902112	0.026823876	0.544785	1	0	5	2	17	10
Ccr5	2.764192	1.137127	9.971243	0.001590041	0.105305	10	1	3	5	31	22
B3galt5	2.76185	3.956344	69.19023	8.94099E-17	1.92E-12	75	41	32	89	352	195
Ccdc13	2.70962	3.053109	10.78412	0.001023744	0.078949	0	0	3	4	11	0
Dcc	2.704087	0.34656	4.425469	0.035406376	0.625721	4	0	0	0	10	7
Serp2	2.696836	5.272013	12.75593	0.000354882	0.039977	0	0	3	0	9	5
Serpind1	2.689633	3.077667	11.81074	0.0005889	0.055818	0	0	4	13	4	0
Lpl	2.668868	1.967328	7.59369	0.005857295	0.238667	9	26	0	2	119	49
Abcc3	2.666858	0.404253	6.18953	0.012850835	0.362383	6	1	0	3	5	15
Wdfy4	2.620068	0.570892	6.571593	0.010361916	0.323446	4	0	0	3	8	5
Aldh1b1	2.611228	1.430117	8.974025	0.002738447	0.152644	4	0	0	5	7	4
Cnfn	2.609247	4.629092	59.45017	1.2543E-14	1.35E-10	264	122	138	302	981	655
Avil	2.606653	7.959145	8.521967	0.00350885	0.176394	2	0	0	0	0	9
6720489N:	2.592482	1.572674	10.91098	0.000955961	0.075755	6	1	0	13	12	2
Acta1	2.583548	0.435204	3.964019	0.046482612	0.704916	1	0	0	2	2	1

Gpr123	2.547088	2.22593	6.042124	0.013968472	0.383349	0	1	1	8	0	0
Sox2	2.538395	4.445089	7.857341	0.005061477	0.220451	0	0	2	0	9	0
Lyz2	2.534881	2.182282	18.59316	1.618E-05	0.004298	30	15	3	20	106	53
Mustn1	2.51183	1.492278	8.671686	0.00323192	0.168782	2	3	0	0	12	8
Nfam1	2.505507	0.872797	5.239523	0.022079332	0.494358	8	0	7	0	21	27
Scn2a1	2.483773	3.06872	8.643283	0.003282693	0.169785	0	1	2	4	0	7
Lcn2	2.475035	1.788015	10.471	0.001212629	0.08697	26	19	21	16	63	119
Zfp128	2.460565	0.260369	5.233909	0.022150694	0.494906	3	1	0	5	3	5
Pou3f1	2.45625	6.283834	25.50593	4.41026E-07	0.000287	5	0	5	11	18	7
Nsg1	2.45456	4.048026	13.558	0.000231304	0.029104	0	5	0	4	16	0
Intu	2.452676	0.28285	5.261313	0.021804564	0.492158	7	6	0	13	15	14
Frmd7	2.442264	0.940004	4.871847	0.027298273	0.548134	3	0	0	0	6	5
Shisa4	2.42066	4.15143	20.12327	7.26077E-06	0.00248	0	10	1	7	12	18
Fam181b	2.415974	3.331226	6.266449	0.012304546	0.354886	0	2	0	0	4	4
Clcn5	2.40528	3.111664	9.667104	0.001875968	0.119762	5	0	0	4	0	11
Emilin2	2.39475	0.700761	4.59951	0.031981101	0.593482	0	9	0	6	21	9
Rgs16	2.391369	0.459063	4.402378	0.035888864	0.62938	3	0	0	4	6	1
Dbndd1	2.373083	3.660443	8.351306	0.003854102	0.187189	2	0	1	0	6	5
Trpv2	2.361117	4.254681	8.570931	0.003415724	0.173743	0	0	3	3	6	2
Ace	2.358007	0.793154	4.630693	0.031404873	0.586551	2	1	0	1	9	2
Bend6	2.340067	7.334933	10.94064	0.000940777	0.075755	0	3	1	6	6	2
Ncf1	2.337443	2.3516	21.55684	3.43496E-06	0.001369	21	13	3	18	71	33
Sdk1	2.336527	3.037063	11.12089	0.00085361	0.072308	5	0	2	2	6	13
Nova2	2.33268	2.975271	7.701866	0.005516377	0.23002	0	0	3	3	7	1
Cd163	2.325037	1.800074	6.004572	0.014268856	0.388619	4	0	0	8	5	0
Sqrdl	2.29122	1.492212	11.18053	0.000826601	0.070857	9	9	2	11	59	6
1700029I1	2.282518	0.256491	5.649999	0.017455384	0.439849	4	2	3	6	24	3
Casp1	2.279062	1.56527	6.135017	0.013253026	0.371776	14	3	2	7	69	0
Fa2h	2.27787	4.336233	5.566976	0.018302341	0.449928	0	2	0	1	0	6
Apc2	2.250771	4.221415	14.40189	0.000147654	0.021936	0	1	6	3	16	4
Cpne4	2.227445	4.724704	10.97634	0.000922825	0.075755	0	0	5	2	9	5
Elmo1	2.215123	3.755488	16.44463	5.00917E-05	0.009729	4	2	4	5	14	11
Cmpk2	2.18138	3.536197	16.64125	4.51583E-05	0.009056	13	5	0	5	19	24
Lrrc48	2.164437	0.212031	4.023868	0.044860703	0.69893	3	1	0	1	4	6
4632428N(	2.155179	2.09758	15.5525	8.02456E-05	0.013703	7	9	15	23	41	24
Ptprr	2.152264	3.888012	6.577931	0.010325087	0.323011	0	3	0	0	10	0
Nipal1	2.139494	2.089974	10.75695	0.001038885	0.079488	1	11	6	14	12	21
Ldb3	2.125954	3.059443	13.02983	0.000306569	0.036243	12	3	0	8	11	18
Rasd2	2.125617	2.722287	4.539761	0.033116216	0.605326	0	4	0	0	0	10
Cfp	2.120442	1.517194	10.4932	0.001198147	0.086508	8	21	12	28	89	17
Fxyd5	2.11669	3.66635	21.81921	2.99585E-06	0.001289	38	12	6	20	66	56
Sytl2	2.108998	4.131267	10.57456	0.001146548	0.083909	3	3	0	5	13	0
Rph3a	2.103857	7.872607	6.425151	0.011251532	0.33717	0	0	3	5	0	4
Tm6sf1	2.088373	0.446061	7.570784	0.005932197	0.240371	5	7	2	15	18	6
Celsr3	2.086823	3.733945	4.189912	0.040665209	0.666377	2	0	0	6	0	0
Nalcn	2.074244	3.846983	25.52391	4.36935E-07	0.000287	4	13	9	14	43	17
Pigz	2.066715	2.912178	12.48585	0.000410046	0.043893	5	0	6	2	23	7
Shc2	2.0494	4.636591	4.272141	0.038742092	0.650214	0	2	0	0	0	6
Coro2b	2.047226	6.100321	23.07888	1.55489E-06	0.000797	7	6	2	4	27	10
Ermn	2.045312	4.350566	5.339109	0.020852133	0.482424	0	0	5	8	0	4
Hcn3	2.042643	3.133334	7.402519	0.006513262	0.251146	0	0	5	0	16	0
Lama4	2.034769	4.840982	18.95175	1.34067E-05	0.003727	14	0	0	6	26	6
Stxbp6	2.034087	4.420945	8.44408	0.003662336	0.180514	9	0	0	0	29	0
Gpr179	1.964396	1.406715	6.718201	0.009543382	0.30924	3	6	1	4	11	10
Il22ra1	1.945119	0.773962	6.351565	0.011727781	0.344294	18	7	22	20	98	16
Plekha4	1.944661	6.45366	11.50985	0.000692282	0.063384	1	0	7	11	5	4
Pcdh17	1.940878	5.932583	18.00892	2.19872E-05	0.005379	1	13	0	12	15	8
Crispld1	1.926805	2.620347	8.127399	0.004360126	0.201618	18	1	1	15	32	3
Grip1	1.923959	4.430855	35.59714	2.42643E-09	4.45E-06	57	37	18	44	128	89
Ppp1r3e	1.920289	0.782703	3.95392	0.046762321	0.705555	5	7	0	15	7	5
Pcyt1b	1.889579	5.715672	33.61753	6.70854E-09	1.03E-05	34	17	16	13	110	44
S100a9	1.877071	3.817263	8.824684	0.002971828	0.161878	187	146	73	101	402	379
Cdsn	1.865646	3.314134	10.9085	0.000957242	0.075755	167	72	86	67	331	294
AI428936	1.847384	3.206625	13.19101	0.000281295	0.034002	5	3	12	15	24	8
Sgpp2	1.843926	5.571848	14.74422	0.000123124	0.019059	0	0	15	6	13	15

Trim46	1.842892	3.554254	10.67175	0.001087841	0.082126	0	7	5	2	23	6
D630045J1	1.841322	3.437815	18.41235	1.77901E-05	0.004612	10	15	9	16	28	30
Iffo1	1.828256	4.094497	12.0745	0.000511161	0.050917	3	9	2	10	6	14
Cp	1.823377	5.626036	26.40591	2.76692E-07	0.000205	72	42	37	53	91	147
Nckap1l	1.822577	1.393901	3.869251	0.049178469	0.720347	2	19	10	2	14	45
Prelid2	1.815088	2.896027	14.00954	0.000181886	0.025248	74	69	44	43	229	149
Lilrb4	1.803765	0.661793	4.138228	0.041925031	0.676079	8	3	3	0	22	11
Dfna5	1.803381	1.985125	5.932904	0.014860735	0.400681	7	0	0	5	9	2
Ptpro	1.798469	1.780847	4.599227	0.031986372	0.593482	2	0	7	0	15	7
Ms4a7	1.793197	1.205232	6.935646	0.008449452	0.28995	18	5	12	5	39	30
Chrm1	1.791055	1.053707	7.479585	0.006240245	0.246811	11	5	9	18	25	11
Snph	1.780391	5.192153	20.00605	7.71975E-06	0.002592	16	0	5	11	18	16
Esrrg	1.77696	3.391285	8.107857	0.004407381	0.201764	0	9	0	5	15	2
Gal3st3	1.775574	1.780636	5.64411	0.017514105	0.440226	0	1	7	5	11	3
Dysf	1.773413	4.755647	6.059704	0.013830088	0.381498	3	2	0	0	12	0
AI662270	1.773233	1.661805	5.103896	0.023872171	0.516307	15	14	3	4	15	39
Slc39a14	1.771217	1.67146	6.199553	0.012778261	0.361759	1	6	7	15	5	8
Tmem56	1.766957	4.440873	12.24644	0.000466148	0.04776	10	3	0	12	11	5
Trappc2	1.756962	3.9469	19.70341	9.044E-06	0.00282	13	6	15	24	24	21
Acvrl1	1.756528	2.845543	11.93673	0.00055038	0.053342	7	6	5	4	29	9
Cfh	1.751298	2.976423	6.668243	0.009814589	0.313775	15	3	2	5	6	23
Veph1	1.747748	0.941993	5.317901	0.021107416	0.485632	9	5	27	18	33	31
Sv2c	1.741765	5.891348	5.602654	0.017933287	0.446595	0	8	0	4	11	4
Mpped1	1.741176	1.673161	4.153772	0.041541875	0.674069	8	0	0	1	5	9
Clec7a	1.739956	1.881327	5.724563	0.016729201	0.429604	1	3	16	5	40	7
Klhl29	1.738932	2.061302	8.417451	0.003716365	0.182144	5	4	5	6	23	4
Coro1a	1.738108	4.500067	29.3752	5.96359E-08	6.11E-05	13	11	20	17	45	31
Rasgrp3	1.72296	3.011795	12.81649	0.000343578	0.039321	6	9	11	14	46	3
1810041L1	1.720277	5.794856	4.719736	0.029818326	0.573757	4	0	0	2	5	2
Stk32a	1.7183	2.038226	6.016534	0.014172452	0.386973	16	0	9	6	52	4
Chd5	1.710741	5.107515	16.36514	5.22372E-05	0.009946	7	3	8	4	20	14
Rnf182	1.710458	2.712347	15.88199	6.74172E-05	0.011988	16	14	14	28	63	9
Gpr156	1.706957	1.683897	5.305661	0.021256227	0.48706	0	12	3	11	23	3
Plcxd3	1.702915	4.770777	7.295622	0.006912285	0.259331	0	7	0	0	16	0
Gatm	1.693698	5.918767	4.481266	0.034268331	0.614431	4	0	0	0	8	1
Fabp5	1.664596	7.541009	43.45662	4.33463E-11	1.6E-07	1716	1654	1337	1349	5536	2915
6230409E1	1.658792	5.674642	9.3735	0.002201448	0.131573	1	7	2	5	13	3
9330159F1	1.65587	5.489106	7.677997	0.005589818	0.231735	0	0	8	2	13	2
Celf4	1.652037	6.466236	56.27304	6.30745E-14	4.52E-10	49	30	41	42	154	54
Spp1	1.650366	3.891684	10.3084	0.001324262	0.093419	0	11	5	4	16	13
Cd93	1.632256	3.141299	11.69259	0.000627494	0.059003	14	0	19	12	33	19
Akr1b8	1.626032	2.385735	9.419533	0.002146862	0.129389	40	36	53	27	105	106
Snhg11	1.622243	8.554883	8.276489	0.004016172	0.192444	2	3	20	21	4	17
2900097C1	1.621864	2.002593	9.034281	0.002649632	0.14885	13	9	9	21	32	9
Robo2	1.610326	5.490906	16.88318	3.97524E-05	0.008304	22	8	4	3	41	23
cip7	1.608446	0.936863	3.985664	0.045889	0.703111	0	10	12	8	18	17
Gria3	1.602819	3.88974	15.24158	9.45974E-05	0.015657	15	8	7	16	23	17
Gipc2	1.600912	1.814743	7.590416	0.005867939	0.238667	11	23	31	19	45	54
3110082D(	1.600569	1.905799	7.750462	0.005369882	0.224783	5	8	6	6	28	7
Fancf	1.600407	1.722036	6.489487	0.010851428	0.331647	7	16	7	9	33	19
Oas3	1.598231	1.998645	3.954911	0.046734782	0.705555	69	45	35	11	116	133
Clec12a	1.598098	0.642302	3.926842	0.047521106	0.71178	12	5	3	7	16	13
Cldn1	1.571874	4.22912	32.47661	1.20636E-08	1.73E-05	97	81	61	79	215	147
B3galnt1	1.563781	3.537587	6.932119	0.008466133	0.290059	7	8	14	4	9	33
Irgm2	1.5622	1.837549	9.299226	0.002292507	0.135109	25	16	11	11	69	25
Ttyh2	1.562057	3.455289	14.25603	0.000159549	0.023195	16	9	5	7	29	20
Hmha1	1.561934	1.917173	8.272546	0.004024903	0.192444	20	14	6	17	43	17
Trpc2	1.561711	0.492295	3.950426	0.046859491	0.705555	3	8	12	8	21	15
C3ar1	1.558869	3.078675	17.55321	2.79379E-05	0.006679	62	43	26	35	155	66
Kif5c	1.557242	8.01035	10.84776	0.000989155	0.076833	12	0	2	6	19	2
Kcnab3	1.551746	1.322217	4.636822	0.031292896	0.585856	13	6	3	3	32	10
Beta-s	1.54937	7.985661	4.402775	0.035880504	0.62938	47	47	11	14	260	4
Glipr2	1.547527	3.357239	7.115243	0.007643126	0.27546	6	2	3	8	11	2
Asns	1.544598	5.470378	19.24544	1.14945E-05	0.003474	107	34	47	32	200	112
Jph3	1.541853	5.516859	4.319949	0.037668041	0.643276	4	1	0	0	10	0

Lphn3	1.530869	2.42231	6.455275	0.01106234	0.333966	20	4	8	6	57	6
Prkd1	1.516851	2.537231	5.959152	0.014641081	0.396469	9	0	8	2	23	8
Dclk1	1.495958	5.975602	23.46077	1.27487E-06	0.000741	22	8	19	7	66	20
Gcnt2	1.487562	7.310355	4.632584	0.031370277	0.586414	6	0	0	7	0	4
Ablim3	1.483726	3.92719	5.940513	0.014796712	0.399456	0	5	4	1	17	0
Clec2l	1.479348	5.551773	21.92941	2.82866E-06	0.001274	19	14	12	21	40	19
Ms4a6c	1.476464	2.029484	5.367556	0.020514701	0.479255	25	18	11	25	20	36
Acot7	1.467884	8.174982	43.58975	4.04955E-11	1.6E-07	91	74	77	79	172	152
Tox3	1.46694	4.208456	8.384764	0.003783794	0.184232	9	6	0	7	15	5
Plxnd1	1.464674	4.440187	13.75711	0.000208032	0.02763	11	4	24	18	49	7
Serpina3n	1.462788	6.237498	4.539493	0.033121404	0.605326	33	497	516	219	749	838
Gpr37l1	1.460719	5.876833	5.047697	0.024658688	0.525071	7	0	0	0	13	0
Rsph9	1.455643	2.749184	10.18025	0.001419532	0.09727	17	3	15	15	25	18
Aldh18a1	1.449485	1.008102	4.733727	0.029576725	0.573309	13	4	5	15	19	4
Fcer1g	1.447389	2.853699	16.69131	4.3982E-05	0.008928	32	38	35	42	88	52
Hba-a2	1.441613	7.217656	4.130122	0.042126304	0.678435	54	26	12	3	197	12
Ifi44	1.440674	3.232026	5.347481	0.020752229	0.482187	199	45	53	29	498	77
Kifc2	1.418846	4.158735	14.30856	0.000155157	0.02271	11	6	30	24	49	11
Ica1	1.417627	4.232624	7.843379	0.005100717	0.221455	7	10	23	5	23	35
Rtp4	1.414422	3.728346	12.36624	0.000437168	0.045661	110	124	142	74	452	177
1700030J2	1.409921	2.376171	6.253198	0.012396931	0.356594	22	26	21	5	94	34
Bnip3	1.404131	5.968689	25.51819	4.38231E-07	0.000287	103	78	104	53	309	143
Sox5	1.402133	2.565192	6.970578	0.008286071	0.287118	10	13	7	8	47	5
Atp6v1g2	1.387296	6.185388	10.01459	0.001553047	0.104098	7	11	0	3	21	7
6330407J2	1.380654	7.992673	5.048404	0.024648635	0.525071	0	8	0	0	14	0
Rnf125	1.379058	4.818924	4.803179	0.028407279	0.559869	5	3	0	0	12	2
Ppp4r4	1.338357	4.0046	10.92454	0.000948985	0.075755	18	7	3	9	28	9
Spink5	1.332289	8.432949	15.4025	8.68733E-05	0.014718	8477	3286	3091	3444	15655	5585
Ecm1	1.331355	6.451535	22.92308	1.68615E-06	0.000844	1485	896	1141	702	3302	1724
Aspa	1.328933	4.451994	8.161232	0.00427953	0.199736	8	2	9	5	18	8
E130309D1	1.318313	4.107368	4.501164	0.033871782	0.609862	0	7	2	7	8	0
Krt14	1.31294	8.382095	21.26881	3.99173E-06	0.001562	5661	4881	3281	3294	10834	7290
Tmem25	1.311147	5.430104	6.634985	0.009999505	0.316862	14	1	1	9	17	0
Fyb	1.308742	3.486409	9.530982	0.002020319	0.124911	50	39	22	29	57	71
Ttc39c	1.308329	3.915659	6.217973	0.012645979	0.359306	3	14	4	2	14	17
Fam43b	1.305819	4.364985	13.07361	0.000299486	0.035799	9	22	18	13	38	27
Sncb	1.300131	7.117983	4.261948	0.0389752	0.653108	0	8	0	7	6	0
Dpp6	1.290755	7.290268	4.230489	0.039704003	0.658422	0	0	8	2	10	1
Klhdc9	1.290429	1.890893	6.036815	0.014010536	0.384014	6	21	21	23	38	17
Osr2	1.286384	3.939775	15.66811	7.54861E-05	0.012993	304	145	141	234	519	174
Epb4.9	1.283327	5.662534	13.7453	0.000209344	0.027633	4	22	11	16	35	9
Angel1	1.282588	3.462179	11.56456	0.000672209	0.062074	15	16	17	34	29	10
S100a4	1.275751	5.919353	17.27289	3.23774E-05	0.007411	336	240	229	138	624	446
Rsad2	1.272896	2.633882	5.083478	0.024154873	0.51857	33	5	4	12	41	12
Tfrc	1.251442	5.049938	18.71782	1.51559E-05	0.004076	213	69	110	99	330	157
Cit	1.244489	2.563659	7.168995	0.007417432	0.270497	49	10	23	20	64	35
Crct1	1.238291	4.470045	28.08671	1.16E-07	9.6E-05	407	233	330	308	790	364
Gbp3	1.233431	2.577935	7.288283	0.006940578	0.259331	46	19	11	21	72	24
St8sia4	1.229321	2.958223	10.73899	0.001049016	0.079755	86	31	28	38	125	53
Rnf157	1.220213	6.404902	8.321772	0.00391727	0.189402	9	6	7	8	24	2
Sp110	1.213221	1.132412	4.396748	0.036007537	0.629868	20	20	21	30	40	19
Pld4	1.209638	2.233884	4.698433	0.030190124	0.576787	6	18	20	8	39	22
Ccl6	1.208688	2.169594	4.507771	0.033741191	0.608529	14	11	13	11	44	8
Lcp1	1.191605	5.703614	7.599571	0.00583822	0.238359	71	22	38	10	81	82
Gm1568	1.187515	2.404498	4.777659	0.028831291	0.563705	3	10	10	10	21	5
Car13	1.187473	2.345678	7.337264	0.006753956	0.256746	43	55	36	72	110	24
Slc30a4	1.184213	4.320893	7.059598	0.007884168	0.279927	229	111	181	94	737	90
Mrc1	1.183917	3.872311	15.14477	9.9574E-05	0.016354	71	44	26	71	73	46
Pglyrp4	1.165672	3.328966	5.108504	0.023808841	0.516307	284	65	124	78	458	162
Laptm5	1.161359	3.622262	9.276538	0.002321077	0.135357	50	63	34	30	97	78
Mmd	1.161359	2.957801	5.854945	0.015533342	0.412612	16	15	56	29	42	44
Smo	1.158544	2.068637	7.247011	0.007101914	0.263457	29	29	27	42	59	21
Cntn1	1.156595	8.946245	30.42967	3.46192E-08	4.14E-05	66	35	64	42	158	46
Eno3	1.153122	1.997577	4.463737	0.034621671	0.618296	27	12	35	30	42	27
Cidea	1.128417	4.253781	9.970561	0.00159063	0.105305	33	4	22	21	46	15

Sulf2	1.124048	4.955007	16.04913	6.17201E-05	0.011254	34	14	28	23	68	18
Orai2	1.120475	3.223448	6.503528	0.010766065	0.330446	27	11	38	30	77	9
Ar	1.119673	4.624346	6.15612	0.013095813	0.367845	21	5	0	7	25	5
2310014H(	1.114058	4.544173	13.3445	0.000259182	0.031866	156	83	83	55	223	151
Adora1	1.111321	5.840457	10.54828	0.001162962	0.084535	25	3	17	24	21	14
Tmem8	1.099962	4.609333	4.176484	0.040988663	0.668622	6	5	2	4	9	5
Mfap5	1.086474	5.794197	12.26466	0.000461618	0.047751	37	23	4	20	48	19
Atp1b2	1.07947	4.95322	7.809586	0.005196982	0.222943	8	4	22	18	26	3
Mrs2	1.079091	2.602387	6.940297	0.008427514	0.289659	40	19	42	29	58	42
Nacad	1.074696	6.966956	6.699552	0.009643713	0.310478	2	5	17	5	12	15
Il1a	1.071918	2.693285	4.336561	0.037302192	0.640027	191	64	58	67	263	96
Fam105a	1.068192	1.869203	4.312727	0.037828287	0.643489	24	14	6	9	40	13
C3	1.060369	2.709076	9.137046	0.002504852	0.142202	89	70	98	59	221	83
Cybb	1.059129	3.507033	12.64286	0.000377003	0.041823	67	58	52	38	136	67
Tmem181l	1.054185	2.801514	4.935338	0.026312909	0.540734	80	23	48	59	130	21
Ceacam1	1.050121	7.023366	16.6288	4.54556E-05	0.009056	3152	1435	1551	1170	4654	2264
Fkbp1b	1.046432	6.984057	10.89453	0.000964486	0.075755	12	16	14	13	31	12
Mpeg1	1.045099	1.748727	4.065828	0.043759218	0.689761	25	21	10	13	48	17
Col23a1	1.043078	5.295223	25.19831	5.17276E-07	0.000318	262	163	173	137	437	216
Etv1	1.03965	4.469363	10.90753	0.000957741	0.075755	39	20	15	32	48	16
Mdga1	1.036447	3.608609	4.286617	0.038413528	0.646227	1	17	5	5	18	9
Ust	1.028111	4.559284	12.93287	0.000322862	0.037348	26	26	27	31	60	15
Nap1l5	1.024308	7.157431	4.864104	0.02742104	0.548829	9	8	1	9	4	10
B230206H(	1.020998	5.020254	14.14416	0.000169322	0.024127	46	38	54	26	102	54
Csrnp2	1.020326	2.572434	3.843001	0.049954038	0.725996	54	11	10	14	65	20
2010016I1	1.017205	1.707025	4.174299	0.041041539	0.668977	48	52	53	65	80	46
Cdr2l	1.017102	5.28676	8.723368	0.003141572	0.165982	25	15	36	8	81	20
Chst1	1.014639	4.234149	5.100103	0.023924428	0.516307	4	20	7	8	23	11
Cyp4f39	1.010898	2.093859	3.967285	0.046392518	0.704916	33	23	12	25	40	20
Kcnip2	1.009619	5.814021	8.32953	0.003900575	0.18902	9	15	18	12	38	7
Lce3a	0.999861	8.123519	43.39817	4.46606E-11	1.6E-07	5872	3680	4062	3929	8418	4561
Adcy7	0.999069	2.633515	6.437136	0.011175869	0.33537	61	58	77	63	114	68
Nfasc	0.996458	7.691302	8.923661	0.002814996	0.154904	20	5	19	5	21	28
Mef2c	0.993958	6.128493	17.65589	2.64694E-05	0.006399	33	39	60	49	69	46
Rassf4	0.99321	6.000451	23.28121	1.39961E-06	0.000755	107	75	78	111	115	83
Fam82a1	0.990169	2.22795	3.971815	0.046267875	0.704529	23	14	11	19	27	13
Cx3cr1	0.986871	5.041235	12.41707	0.000425426	0.045091	216	164	126	111	253	229
Wisp1	0.985652	3.239455	4.379971	0.036363621	0.631987	63	90	67	21	197	88
Ccdc84	0.981271	2.046338	3.886962	0.048662387	0.719107	39	19	47	24	97	24
Csf1r	0.981132	5.199999	11.35372	0.000752969	0.067786	155	132	181	89	285	202
Rnf24	0.980306	3.758715	7.070479	0.007836428	0.279616	32	13	21	20	32	26
Mcc	0.978506	2.809821	5.591448	0.018048366	0.447036	136	49	57	81	149	63
Ttc39a	0.974356	2.757242	4.124145	0.042275372	0.679353	11	16	12	8	24	17
Ubash3b	0.970846	7.420903	8.025204	0.004613083	0.208958	39	18	12	14	40	29
Lix1	0.969555	6.264506	4.075139	0.043518677	0.68748	10	2	5	4	6	11
Pla2r1	0.968684	2.865331	7.922279	0.004882974	0.215291	76	78	49	54	107	82
Sh2d3c	0.962537	3.473922	4.141809	0.041836431	0.676079	20	2	5	10	14	8
Drp2	0.961575	7.565775	4.857497	0.027526242	0.549401	3	0	18	0	22	5
Krt19	0.957664	4.845478	9.292878	0.002300464	0.135109	556	427	407	254	831	583
Adamts5	0.953351	5.854356	15.09221	0.000102385	0.016621	36	28	48	44	54	36
C1qc	0.944101	3.768129	7.083517	0.007779618	0.279088	94	64	38	47	127	65
Dyrk1b	0.942764	3.103831	7.440625	0.006376774	0.250156	69	48	43	28	113	58
Trip13	0.941673	2.680215	7.201068	0.007286019	0.266609	144	60	94	96	185	77
AK010878	0.941575	2.090964	4.211969	0.040139724	0.662225	23	27	42	29	67	23
Ifit1	0.938444	4.124354	9.63308	0.001911039	0.120201	228	168	147	123	416	157
Gabrb2	0.936463	4.324564	7.765923	0.005324109	0.224412	24	12	23	15	51	10
Tmem121	0.931006	4.796996	6.355773	0.011699997	0.344294	20	11	22	9	54	8
Pacrgl	0.928252	1.904835	3.897951	0.048345033	0.718364	26	14	26	25	25	23
Daam2	0.927624	3.893067	4.718025	0.029848008	0.573757	29	11	7	25	19	9
Slfn2	0.925944	3.671827	5.247245	0.021981548	0.494008	271	152	168	144	410	172
Klk10	0.919076	3.363513	5.634215	0.017613231	0.441171	253	107	164	98	386	161
Klhdc8b	0.916826	4.035495	10.47848	0.001207734	0.086908	51	56	72	66	90	54
Kprp	0.916734	2.503476	5.127321	0.023552055	0.514986	100	69	114	79	192	78
Pfkp	0.915234	6.936491	21.91953	2.84326E-06	0.001274	141	145	106	84	291	119
Cnp	0.912163	7.691365	18.00797	2.19981E-05	0.005379	131	74	89	72	135	121

Gdap1	0.893312	6.752985	3.978144	0.046094337	0.703111	13	8	0	2	14	9
Kcna1	0.890176	9.5042	6.241299	0.012480493	0.358473	18	9	14	4	14	28
Fam178b	0.886657	6.740089	7.210287	0.007248691	0.266447	20	13	9	12	29	9
Sec24d	0.884938	2.861037	5.991125	0.014378034	0.390603	102	33	60	42	119	62
Cdc42ep2	0.882869	4.831933	10.21568	0.001392513	0.096339	63	37	19	29	77	34
Cd200	0.878569	4.343482	4.010878	0.045207604	0.700601	14	5	12	14	17	5
Camk4	0.8776	3.928188	10.89409	0.000964717	0.075755	85	61	51	46	114	67
Scara3	0.87667	3.570976	6.900143	0.008618888	0.293727	47	29	47	35	59	43
Nmb	0.875652	6.641125	5.821826	0.015828504	0.415865	17	7	10	7	23	10
Scg5	0.875064	5.489247	4.536626	0.033176932	0.605326	10	6	11	10	18	4
Gpr64	0.874934	4.688271	7.407296	0.00649599	0.250929	28	13	24	9	52	18
Isg15	0.874573	5.174099	12.93688	0.000322172	0.037348	712	418	538	336	1274	450
Adamts1	0.870229	5.334972	9.991899	0.001572304	0.105061	450	669	430	645	649	431
Accn2	0.868134	5.143399	7.472033	0.006266475	0.247394	19	11	25	19	32	13
Aatk	0.867533	8.915216	16.0801	6.07188E-05	0.011166	72	58	63	50	98	70
Eepd1	0.866839	5.842042	8.648471	0.003273359	0.169785	21	28	18	12	62	10
Pdzd4	0.866418	4.354298	5.372774	0.020453421	0.479255	30	25	13	34	45	3
Gng5	0.865576	3.24461	5.665791	0.017298916	0.438869	186	70	156	132	253	91
Ifitm1	0.862798	8.806115	21.01567	4.55543E-06	0.001697	9476	6565	6930	4570	16526	6712
Ifitm3	0.862063	7.152773	19.07505	1.25677E-05	0.003605	2038	1568	1190	983	3034	1574
Ankrd43	0.848271	2.827886	4.419029	0.035540258	0.626277	57	49	20	47	43	41
Fam19a5	0.840598	4.273132	7.482724	0.006229378	0.246811	40	15	17	19	42	20
Odz4	0.838093	4.785427	9.107838	0.002545166	0.143732	224	234	178	165	293	231
D17H6S56I	0.825694	4.678549	10.19531	0.001407982	0.097097	75	35	74	47	107	52
Zfp422	0.820316	3.166358	4.848832	0.027664864	0.551656	101	32	92	60	157	45
Lce3f	0.809824	2.140616	3.845787	0.049871109	0.725696	92	76	62	76	122	56
Rnase4	0.80861	4.397148	7.143307	0.007524428	0.273011	227	173	171	113	439	138
Plxnc1	0.807274	5.333388	6.237652	0.012506216	0.358473	51	22	13	16	39	35
Gdpd2	0.805439	4.637227	10.92237	0.000950102	0.075755	441	334	379	345	717	258
Wnt9a	0.802692	4.850909	5.921726	0.014955305	0.402223	546	301	523	322	702	437
Gpr172b	0.800695	2.62636	4.066065	0.043753088	0.689761	37	21	37	20	62	26
Bcl2l15	0.793556	5.098155	7.320885	0.006815783	0.25773	789	374	683	296	1335	510
Noxo1	0.789184	2.708461	4.138708	0.041913156	0.676079	94	72	49	46	114	71
Ier5l	0.784008	2.956932	4.040863	0.044411113	0.693936	72	30	79	68	84	38
Itgbl1	0.780345	3.465857	4.527153	0.033361083	0.606248	189	156	174	124	286	155
Twf2	0.777362	3.77648	4.73687	0.029522743	0.572778	19	37	40	17	57	34
Apba1	0.771741	7.272902	6.028	0.014080676	0.385445	28	13	12	12	44	4
Parp14	0.770146	5.361473	11.24354	0.000799006	0.069123	663	507	489	307	928	550
Irf9	0.769809	5.357071	13.98992	0.000183793	0.025284	562	490	381	290	877	424
Fyn	0.76893	6.334412	6.360166	0.011671064	0.344294	23	27	11	20	21	23
Sall2	0.766531	3.899992	5.01224	0.025168721	0.527777	23	28	15	17	39	17
C1qa	0.765368	4.56947	5.817218	0.015870029	0.416022	171	133	80	76	177	140
Mbd4	0.761914	3.74831	8.654548	0.00326246	0.169785	136	91	81	108	173	54
Sardh	0.759253	2.982124	4.399456	0.035950388	0.62938	38	20	31	24	58	17
Fam171b	0.757738	6.621309	6.621587	0.010075003	0.318317	64	36	32	34	115	9
Il1f9	0.757578	4.450833	5.283558	0.021527711	0.489635	615	337	253	210	749	348
Stk11ip	0.755805	3.293001	5.316351	0.021126201	0.485632	89	55	86	58	153	48
Anxa3	0.75456	4.035785	7.287791	0.006942481	0.259331	211	159	216	112	336	184
B4galnt1	0.751776	5.016262	11.637	0.000646527	0.060219	198	81	116	85	245	97
Qsox1	0.750486	6.760765	13.2153	0.000277674	0.033946	1460	1000	833	573	1950	1020
Ppm1j	0.747011	5.217098	6.116307	0.013394037	0.374268	22	15	32	29	36	9
Lrrc4	0.739406	4.548596	9.238136	0.002370261	0.136725	400	165	320	241	505	193
Slc16a6	0.739109	4.577735	8.194404	0.004201981	0.196971	115	119	89	75	198	83
Acpp	0.736324	6.744303	27.13128	1.90097E-07	0.000151	1090	790	689	664	1429	640
Tmem229I	0.735385	6.653288	12.16565	0.000486776	0.048941	63	47	43	33	84	45
Trmt2b	0.735078	4.166074	8.07029	0.004499692	0.205553	118	127	163	116	169	128
Bnc2	0.730588	3.890631	4.551911	0.032882025	0.603145	22	39	15	22	38	21
Dnajb9	0.728872	3.107109	4.209925	0.040188104	0.662225	57	55	49	41	112	30
Elovl4	0.728064	5.456318	4.717001	0.029865787	0.573757	37	8	14	6	37	19
Itpripl2	0.725027	5.295232	11.96457	0.000542217	0.053029	495	254	323	262	519	300
Serpini1	0.724261	4.900461	6.905545	0.008592881	0.293467	70	67	35	35	121	37
Clspn	0.715635	2.995924	4.377021	0.036426603	0.63257	220	100	122	135	176	114
Prmt10	0.715287	3.700576	4.977772	0.025675035	0.531688	111	83	98	65	196	63
Tfb2m	0.714334	3.053401	5.034861	0.024842082	0.526975	97	59	62	50	120	57
Gm8580	0.709338	3.114513	3.875057	0.049008648	0.720347	136	122	154	91	208	125

Rnf41	0.706557	3.944335	4.402911	0.035877646	0.62938	73	33	39	36	70	39
Lgals9	0.705976	4.557953	8.528369	0.003496528	0.176394	100	104	91	63	146	94
Pop4	0.705793	4.497314	6.099437	0.013522492	0.376391	328	202	148	158	482	118
Marveld3	0.700923	3.657252	4.319819	0.037670929	0.643276	349	145	187	155	400	153
Col11a1	0.700625	4.550383	7.803864	0.005213462	0.222943	290	217	217	160	292	246
Krt7	0.700081	4.172568	8.040059	0.004575406	0.208128	313	368	293	261	491	259
Slc6a14	0.698498	6.22128	9.890956	0.00166093	0.107965	1916	852	1450	837	2573	1031
Kptn	0.698082	2.788439	5.453997	0.019523609	0.466714	66	53	60	50	95	41
Myo5a	0.697933	7.526563	15.9186	6.61255E-05	0.011856	161	77	100	75	191	83
Fam65a	0.696305	5.199431	6.468728	0.010978906	0.333966	54	115	83	66	118	77
Eif4e3	0.691676	5.123406	6.371111	0.011599296	0.343761	41	52	23	27	74	24
I830012O1	0.689489	4.240874	6.884793	0.008693214	0.294093	365	269	262	158	575	231
Nfkbiz	0.68849	5.891434	13.0065	0.000310412	0.036496	1434	800	1007	898	1488	800
Rpusd4	0.683201	3.043725	3.887071	0.048659216	0.719107	66	66	70	74	93	38
Pld2	0.675321	3.044488	4.978743	0.025660625	0.531688	117	112	104	67	233	69
Cd24a	0.671878	7.608128	13.17897	0.000283108	0.03403	1978	1191	1750	991	2528	1392
Lama2	0.671509	4.036423	3.972142	0.046258896	0.704529	77	109	54	58	82	87
4933431E2	0.66472	4.521984	4.381604	0.036328797	0.631987	35	23	45	13	66	29
Arhgdib	0.662678	5.046773	14.09736	0.000173588	0.024411	730	423	500	362	893	410
Wipf3	0.661314	6.986785	4.998589	0.025367996	0.529406	10	25	30	24	25	16
Tspyl4	0.661268	5.613259	5.598192	0.01797902	0.446694	34	17	29	28	30	20
Slc16a13	0.660032	3.187751	3.884645	0.048729559	0.719256	199	123	144	92	335	85
Gramd1a	0.659656	4.427139	4.286579	0.038414396	0.646227	42	20	35	33	54	12
A930005H1	0.655956	3.421953	4.962492	0.025902856	0.534862	73	75	69	41	117	63
Zfp651	0.649275	5.305559	8.764867	0.003070885	0.164571	101	149	121	113	172	87
Als2cr4	0.647658	4.782092	7.122449	0.007612465	0.274815	105	96	124	52	182	97
Matn2	0.647648	4.925273	6.273091	0.012258511	0.354032	47	33	29	34	56	19
Rarres1	0.638227	4.528679	6.177507	0.012938441	0.364376	92	69	85	75	108	55
Arhgef10l	0.637466	4.45641	6.674022	0.009782819	0.313225	125	80	82	52	146	83
Gas2l3	0.634318	5.858749	6.25536	0.012381809	0.356594	233	107	147	95	333	86
Dnm1	0.634087	6.681009	10.07076	0.001506412	0.101287	99	60	61	38	102	72
Adamtsl4	0.63082	5.07523	8.244898	0.004086671	0.194679	575	385	532	293	956	316
Tcirg1	0.629226	3.617897	4.661396	0.0308481	0.581198	135	129	126	93	165	113
Scai	0.628184	4.728846	7.799529	0.005225985	0.222943	220	253	208	171	359	158
Gapdh	0.626676	5.032133	7.237972	0.007137757	0.26433	220	146	219	107	401	115
Kank4	0.621978	5.920637	7.655503	0.00565994	0.232403	67	68	53	37	128	32
Megf11	0.620218	5.172718	4.984057	0.025581929	0.531391	30	48	32	20	58	32
Ankrd27	0.619722	5.271027	11.07438	0.000875288	0.073565	285	210	180	163	376	138
Cacna2d1	0.617079	7.040251	7.499491	0.006171644	0.246571	58	55	34	57	66	20
Mmp15	0.613909	5.537625	11.3107	0.000770618	0.068441	149	109	107	115	171	67
Oasl1	0.610956	6.303554	8.437949	0.003674705	0.180514	1973	1192	1360	753	2680	1115
Krt6a	0.609982	12.23232	8.795434	0.003019855	0.163254	114663	78089	84813	46217	137584	81565
Zeb2	0.609941	6.816022	8.463185	0.003624066	0.179667	119	129	89	82	136	99
Zfp385a	0.603043	3.816662	4.370989	0.036555774	0.634251	92	77	46	49	120	45
Mt1	0.601001	9.061105	10.98624	0.000917909	0.075755	7357	6339	6239	4343	9743	5129
Filip1l	0.600301	4.083625	4.003854	0.045396341	0.70068	357	197	262	234	443	129
Sfxn2	0.59702	3.983535	3.88431	0.0487393	0.719256	188	216	170	119	321	138
Inf2	0.595773	5.307142	8.031979	0.004595861	0.208617	135	102	111	83	144	96
Tmem183a	0.595268	6.259186	13.69119	0.000215463	0.028237	960	543	707	458	1019	584
Shroom3	0.595006	5.545939	8.239845	0.004098062	0.194679	868	457	520	356	876	490
Glrx	0.593146	4.489313	4.151467	0.041598471	0.674478	108	137	129	73	277	60
Pglyrp1	0.592424	6.971844	4.103579	0.042792533	0.684553	3157	1961	2185	1163	4197	1849
Stom	0.59207	6.334152	11.67576	0.000633196	0.059234	1808	1122	1173	863	1988	1025
Ccdc28b	0.587656	3.95575	5.595338	0.018008333	0.446906	101	117	98	77	173	65
Sod3	0.586665	5.556628	8.503708	0.003544234	0.176975	819	687	679	431	920	654
Pkm2	0.583819	9.199122	11.60476	0.000657832	0.061008	5512	3913	4339	2325	6930	3830
Plekho1	0.582559	5.121365	3.950739	0.046850792	0.705555	63	43	45	15	84	47
Impdh1	0.582077	5.621416	4.300078	0.038110631	0.64566	41	40	34	25	32	45
Ifi27l1	0.576546	7.008507	13.04776	0.000303648	0.036096	1133	925	945	620	1556	722
Zfpm1	0.576014	3.551078	4.704648	0.030081165	0.575312	104	101	94	61	158	72
Slc35d1	0.575186	3.401966	3.888424	0.048620023	0.719107	129	108	155	90	181	98
Rbp1	0.571433	6.960831	8.570919	0.003415747	0.173743	2377	1789	2052	1136	3451	1491
Ilk	0.571059	4.008556	3.857181	0.049533479	0.723041	98	76	121	71	138	69
9530091C0	0.570376	4.298111	4.113701	0.042537176	0.682498	276	298	380	205	436	257
Acpl2	0.570072	4.947393	6.462181	0.01101943	0.333966	596	457	449	320	847	312

Pcdh7	0.567522	6.880277	17.52439	2.83645E-05	0.006706	1037	775	871	581	1481	559
Igf1	0.564615	4.406153	4.982073	0.025611276	0.531391	98	100	116	62	161	79
Med11	0.552999	3.802849	5.450234	0.019565705	0.466714	149	109	116	98	189	68
Pcbp4	0.550933	5.68969	4.146473	0.041721342	0.675453	23	40	44	36	44	20
Dock11	0.550848	5.303067	6.912371	0.008560142	0.292814	362	203	225	204	402	138
Ddit4	0.550513	6.587969	6.273603	0.012254968	0.354032	386	328	309	179	469	292
Dusp1	0.548482	4.995334	6.796925	0.0091315	0.302732	376	497	412	289	610	318
Samd9l	0.548245	5.741724	7.412731	0.006476395	0.250929	1256	898	779	615	1556	626
Slc35e4	0.547299	4.212664	4.514072	0.033617119	0.607959	128	98	106	70	119	101
Ly6e	0.546083	8.439314	11.89997	0.00056135	0.054161	8491	4985	5922	3752	10976	3992
Sec11c	0.541859	5.362518	7.416564	0.006462611	0.250929	232	195	310	167	384	153
Zfp358	0.540125	3.8114	4.509114	0.033714703	0.608529	154	100	113	70	179	90
Nudt7	0.539782	3.943892	6.352287	0.011723007	0.344294	212	160	161	129	284	96
Ifit3	0.535975	5.259431	4.386753	0.036219229	0.631987	626	472	451	249	870	369
Neat1	0.535503	8.677158	7.951521	0.004804697	0.21315	4818	5701	5318	3750	7314	3712
Serinc2	0.534732	5.858619	5.172731	0.022944125	0.506052	1237	704	846	374	1633	672
Stfa3	0.527132	3.767406	4.001769	0.045452546	0.701044	310	230	267	157	383	199
Dip2a	0.526233	4.371574	5.288841	0.021462503	0.489181	142	107	147	105	198	68
Tmem176a	0.526198	6.1684	12.1659	0.000486712	0.048941	551	491	576	353	765	376
Tigd2	0.524438	5.160851	9.397781	0.002172483	0.130567	412	335	275	260	505	188
Cxcl16	0.521357	4.384319	5.589745	0.018065925	0.447036	356	267	208	161	402	198
Zfp513	0.520834	4.141898	5.697497	0.016989125	0.433757	185	206	184	137	286	118
Gmppa	0.520001	4.663667	8.001092	0.004674915	0.209553	347	232	303	178	397	218
Spats2	0.518613	4.829132	5.636001	0.017595296	0.441171	220	203	136	135	250	123
Chchd6	0.518438	5.246524	5.873456	0.015370851	0.409306	246	191	175	139	277	138
1600029D2	0.517886	9.969925	11.39737	0.000735482	0.066771	27199	14336	19287	12376	30527	12849
Gpha2	0.516416	4.490986	4.313277	0.037816059	0.643489	447	334	535	231	665	324
Dnm3	0.511519	7.515206	5.272309	0.021667252	0.489818	44	66	39	26	78	35
Lrig2	0.510645	4.937837	6.19102	0.012840019	0.362383	289	199	176	178	247	148
Rnf208	0.508854	7.110025	8.439918	0.003670728	0.180514	1036	566	642	377	1087	552
Arl4c	0.50813	5.520069	10.62671	0.001114657	0.082986	335	233	256	177	379	186
Itih5	0.506896	6.90761	13.64929	0.000220325	0.028386	690	548	491	441	755	353
Mon1b	0.50596	4.042429	4.534628	0.033215675	0.605326	255	121	169	115	222	133
Slc2a8	0.5005	4.246041	4.176992	0.040976364	0.668622	155	129	113	65	194	102
Dsg1b	0.498952	5.995976	10.89731	0.000963041	0.075755	1562	984	1308	811	1754	867
Ckmt1	0.49885	6.793719	8.30165	0.003960907	0.190374	1285	1059	889	602	1313	889
Mxd1	0.494295	7.830339	8.019142	0.004628549	0.209218	5598	3638	4107	2393	6744	3019
Armc5	0.494169	4.206411	3.872083	0.049095565	0.720347	181	167	138	121	280	67
Fam132a	0.492695	4.24115	4.926081	0.026454249	0.541055	239	194	161	156	259	114
Dtna	0.492458	6.087402	4.124039	0.042278032	0.679353	54	82	87	36	111	59
Dab2	0.491641	4.420909	4.16438	0.041282524	0.671202	213	237	177	146	307	126
Tep1	0.489392	4.574768	4.421972	0.035479014	0.625721	287	267	373	200	443	202
Lgals7	0.488635	5.303457	6.021807	0.014130172	0.386308	783	795	654	521	1220	382
Scd1	0.48737	7.454021	4.400296	0.035932686	0.62938	741	535	364	225	798	441
Grin1	0.486879	5.992312	4.257048	0.039087781	0.654359	61	25	48	26	56	35
Sidt2	0.48589	6.073105	6.4088	0.011355612	0.338873	1057	684	878	419	1139	715
Fam162a	0.485429	6.006148	7.991141	0.004700677	0.209834	907	830	948	573	1408	524
Magi1	0.485358	6.487522	7.343513	0.006730519	0.256308	119	113	154	76	204	77
Parm1	0.484455	8.280592	6.753734	0.00935517	0.305441	1047	1077	904	499	1433	798
Rfng	0.481275	5.067725	7.062937	0.007869487	0.279927	386	302	381	205	491	255
Tns1	0.480867	7.16856	12.03257	0.00052279	0.0514	236	191	240	144	303	147
Upp1	0.478862	5.135118	3.841626	0.04999502	0.725996	841	605	603	372	959	487
Vopp1	0.474527	6.067232	8.484862	0.003581137	0.17836	960	703	599	495	943	515
BC100530	0.473746	9.969487	7.490196	0.00620358	0.246811	22406	20169	17727	11333	31186	12966
Dennd4a	0.469705	4.484449	4.091712	0.043093976	0.68529	133	78	101	53	139	79
Tpm2	0.46765	7.326003	5.50409	0.018972049	0.458655	2770	3001	3187	1755	5056	1708
Ifnar2	0.467397	5.499852	4.886121	0.027073453	0.547313	406	265	411	164	499	284
Prr13	0.466843	8.074541	7.780133	0.00528239	0.223293	3723	2377	2876	1413	4713	2015
Slc39a5	0.465075	5.290979	6.653096	0.009898366	0.315049	197	146	194	97	261	121
1110059E2	0.464847	5.704466	8.727039	0.003135253	0.165982	837	484	725	476	848	428
Ankrd29	0.464648	4.239205	4.576074	0.032421424	0.598041	202	146	199	119	282	97
Eps8	0.460867	5.587775	4.285168	0.038446279	0.646258	1169	670	856	503	1463	499
1110051M	0.460241	4.9634	4.050366	0.044161765	0.691546	80	56	57	54	62	45
Pfkl	0.458956	6.01413	5.821686	0.015829761	0.415865	710	710	620	366	1028	458
Slc25a1	0.456443	7.409823	8.300272	0.003963915	0.190374	1313	1097	1249	625	1623	932

Clybl	0.456411	5.483237	4.931141	0.026376889	0.541015	241	198	150	105	258	147
Osmr	0.455187	4.700726	5.131951	0.023489305	0.514136	577	318	485	318	563	288
Btrc	0.452693	4.916223	3.930686	0.047412587	0.711107	109	146	119	74	213	65
Parp12	0.452217	4.650769	5.275226	0.021630982	0.489818	529	347	430	252	553	309
Mtap1a	0.451888	9.880104	7.96945	0.00475734	0.211858	183	121	172	94	243	88
Wnk2	0.451674	4.851456	5.600604	0.017954291	0.446595	585	374	533	296	648	338
Pbp2	0.450399	5.825471	5.876146	0.015347377	0.409187	1577	826	1129	726	1474	778
Fam172a	0.448183	5.438973	7.133144	0.007567194	0.273798	421	366	359	250	606	192
Muc4	0.44636	9.103555	9.232199	0.002377959	0.136803	14521	8182	11479	6649	15579	7274
Sirpa	0.446136	5.38849	4.462284	0.034651145	0.618296	587	360	343	242	680	238
Cdk2ap1	0.445286	5.041951	5.824717	0.015802512	0.415865	453	405	338	236	560	258
Rab3a	0.443464	8.267613	7.227247	0.007180524	0.265002	153	118	117	65	198	81
Cda	0.440216	6.547859	5.601833	0.017941694	0.446595	1495	1199	1122	610	1763	940
Ankrd54	0.437771	4.237666	5.100775	0.02391515	0.516307	280	242	219	173	302	156
Tgfbi	0.437545	8.837142	7.695231	0.005536691	0.230421	10348	8009	8573	5912	11476	5650
2610203C2	0.437486	4.452797	3.868005	0.049214986	0.720347	191	149	217	147	194	119
Oxa1l	0.435223	4.868804	5.27765	0.021600884	0.489741	367	292	270	217	426	168
Smox	0.435154	6.607025	5.716186	0.016809205	0.431069	1757	1095	1431	768	1712	1071
Pik3c3	0.431636	4.866199	5.122425	0.023618583	0.515307	272	205	244	140	321	159
Stat2	0.431072	5.14928	3.987718	0.045833091	0.703111	307	288	243	159	333	212
Rab34	0.43091	4.728428	3.96257	0.046522637	0.704916	313	243	243	165	314	188
Casp9	0.424938	4.624597	4.115841	0.042483408	0.682144	329	212	320	199	387	155
Ankk1	0.422288	4.731514	3.92429	0.047593297	0.712112	702	375	587	363	737	313
2310001A2	0.409218	4.734806	4.104084	0.042779747	0.684553	237	173	176	163	230	95
Il6st	0.408522	5.365105	4.558625	0.032753362	0.602741	352	221	225	134	323	198
Ldha	0.408408	7.704241	10.3859	0.001269815	0.090518	3103	2336	2707	1661	3729	1600
Pde4d	0.404579	5.255696	4.194906	0.040545594	0.665938	195	114	128	96	166	94
Ndufa4	0.40414	7.08726	8.735936	0.003119991	0.165982	1976	1160	1513	1016	2123	815
Nucb2	0.402858	5.055274	4.534097	0.033225973	0.605326	536	462	424	259	642	311
Pdlim7	0.399118	6.555864	4.778317	0.028820278	0.563705	203	147	117	85	180	116
Rab24	0.399017	6.957075	6.452034	0.011082542	0.333966	1167	1004	1045	600	1338	742
BC056474	0.39484	5.339863	5.713738	0.016832662	0.431157	534	376	381	253	580	256
Mrps9	0.393307	5.194357	4.367968	0.036620635	0.634251	382	228	366	194	474	171
Tmsb10	0.393111	5.851843	5.435489	0.01973157	0.468392	322	253	267	155	380	177
Lyst	0.392338	4.725273	4.079729	0.043400613	0.686623	205	172	228	114	257	135
Limd2	0.391347	5.160957	4.899497	0.026864517	0.544785	315	267	275	229	297	165
Mapk11	0.390699	5.300848	5.803991	0.015989841	0.416022	331	202	224	177	329	126
Ube2a	0.390426	5.231424	5.117954	0.023679526	0.515677	493	329	398	269	474	250
Msn	0.388616	7.810328	5.19707	0.022624988	0.502143	324	322	276	165	366	228
Serpinb1a	0.388495	6.334673	5.563403	0.018339732	0.449928	1106	753	666	540	1260	391
Trim25	0.387654	6.575251	6.236134	0.012516943	0.358473	2323	1427	1643	1006	2375	1103
Rgnef	0.3876	4.849792	4.06308	0.043830474	0.690378	458	296	302	238	485	171
Ctsh	0.386932	6.69731	6.770688	0.009266712	0.304866	2297	1818	1892	1240	2730	1130
Col5a2	0.385659	6.033521	8.151862	0.004301697	0.200336	1462	939	1064	807	1382	640
Capn2	0.384951	7.820196	5.771099	0.016291841	0.420306	3644	2587	2610	1477	3884	1985
Ldhb	0.384588	7.357348	3.963342	0.046501285	0.704916	125	71	53	55	99	49
Tgm2	0.381366	9.151631	6.459755	0.011034484	0.333966	13680	9681	11878	6498	15682	7287
Prkce	0.380874	8.399661	5.588775	0.018075929	0.447036	453	258	249	174	368	224
Slc9a9	0.380363	7.318099	4.674369	0.030615949	0.580381	236	186	159	84	294	121
Rnf19a	0.378657	5.899207	5.563338	0.018340415	0.449928	316	304	252	195	379	159
Hdac1	0.376444	5.023254	4.518284	0.033534451	0.607346	632	415	507	309	695	293
Slc39a1	0.375948	5.571921	5.45384	0.019525358	0.466714	952	516	726	454	951	402
Sobp	0.375638	5.579599	4.02103	0.044936273	0.699601	148	137	140	90	217	62
Cat	0.374521	5.912064	5.783023	0.016181683	0.419417	230	232	231	139	328	125
C030046I0	0.371344	7.733351	5.446719	0.019605117	0.467136	4041	2902	3043	1641	4081	2358
Krt6b	0.369826	11.6203	4.716959	0.02986651	0.573757	78886	54924	65844	31431	88328	45097
Oaz1	0.369776	5.672646	6.662707	0.009845125	0.314284	629	485	516	340	697	311
Kcnq1ot1	0.367748	6.31991	4.955794	0.026003381	0.535909	1186	1291	1185	830	1748	591
Tmem176t	0.364974	6.691367	5.166101	0.023031856	0.506183	1005	794	645	581	932	453
Zcchc24	0.364834	5.63815	4.007481	0.045298781	0.700601	237	175	159	127	250	96
Tpi1	0.363888	6.967635	5.491221	0.019112196	0.459461	1069	770	949	447	1405	538
Hspg2	0.363281	5.77803	4.641045	0.031215983	0.585565	296	275	274	156	350	187
Gpi1	0.360888	7.855084	8.867849	0.002902369	0.158899	1870	1442	1563	880	2155	1000
Snd1	0.360585	5.874195	5.017846	0.025087352	0.527777	911	613	550	390	810	452
Sdcbp	0.360455	7.909569	8.316303	0.003929081	0.189547	2024	1399	1629	950	2220	994

Sharpin	0.354511	4.995635	5.170118	0.022978649	0.506052	367	274	282	202	351	184
Cbr2	0.35362	9.820513	4.787808	0.028661885	0.562141	22152	16281	18801	9380	24312	12792
Capg	0.353548	7.911374	3.902096	0.04822591	0.718364	5426	4235	4462	2416	5997	3090
Ttl	0.351995	7.719209	4.181107	0.040876999	0.66832	264	221	334	173	385	133
Plbd2	0.351618	6.175019	4.671536	0.030666497	0.580542	327	276	318	203	312	205
Dlg5	0.351116	5.002561	3.873688	0.049048621	0.720347	512	371	424	251	508	282
Smap2	0.350209	6.076976	5.341448	0.020824173	0.482296	367	281	349	232	376	188
Ehd3	0.347452	8.253484	4.163547	0.041302834	0.671202	807	724	573	334	882	490
Cldn7	0.343423	7.77474	5.051792	0.02460048	0.525071	5663	3975	4391	2473	5821	2971
Tpm4	0.340442	6.700133	4.10147	0.042845952	0.684586	2055	1544	1676	1415	2012	786
Prkca	0.338533	7.179291	4.029655	0.044707072	0.698053	353	298	337	210	492	139
Psma3	0.33537	6.075794	4.724559	0.029734803	0.573757	687	592	532	436	690	316
Klf9	0.335067	8.092786	5.279863	0.021573443	0.489635	1707	1282	1397	889	1518	966
Gyk	0.334953	5.751856	3.888755	0.048610432	0.719107	1146	674	938	699	1026	437
Zfc3h1	0.334712	5.85405	4.730167	0.029638004	0.573463	945	632	675	575	844	355
Ifngr1	0.334123	6.24533	5.696924	0.016994671	0.433757	1363	940	1029	578	1403	691
Aldoa	0.333946	9.774597	6.122384	0.013348065	0.373954	5188	4074	4351	2289	5948	2837
Cstb	0.330112	7.700128	5.235941	0.022124832	0.494842	4850	2926	3685	2001	5206	2136
Apod	0.328027	10.38769	4.306621	0.037964289	0.644349	10398	7724	6216	4008	10759	4955
Itga6	0.313608	9.201915	4.846474	0.027702707	0.551899	10853	8753	10062	5306	10891	6657
Dab2ip	0.312138	6.578239	5.046749	0.024672183	0.525071	1053	837	843	555	950	581
Ufsp2	0.312002	6.39933	3.954324	0.046751102	0.705555	711	561	662	301	943	360
Tspan5	0.307026	6.657717	6.08017	0.013670758	0.378264	1530	1162	1582	918	1670	768
Raph1	0.306265	6.58075	5.776537	0.016241509	0.420015	979	567	690	469	845	407
Fbln5	0.304277	6.288745	4.616921	0.031658021	0.590255	878	658	771	470	848	458
Vps36	0.297004	5.63755	4.926747	0.026444046	0.541055	924	614	729	490	879	389
Dirc2	0.296904	5.624128	3.948329	0.046917935	0.705555	574	365	543	308	627	239
Col6a2	0.296513	6.735618	4.05332	0.044084562	0.691344	2295	2112	1898	1291	2224	1296
Tpp1	0.294448	6.324951	4.23898	0.039505891	0.656377	1175	800	973	574	1187	539
Pptc7	0.293916	7.525937	4.02726	0.044770596	0.698307	2678	1677	1983	1102	2548	1256
Ssh2	0.292401	6.356147	4.431376	0.035284043	0.625347	1198	670	841	612	942	477
Ppp2r2d	0.290973	6.377653	4.648123	0.031087517	0.584174	634	569	559	343	685	344
S100a11	0.290292	10.57709	4.382029	0.036319749	0.631987	38878	26958	31873	16990	39286	19515
1190003J1	0.287957	7.373791	4.090116	0.043134685	0.68529	4269	3105	3447	1892	4504	2085
Atp6v1c1	0.285636	6.457112	4.652201	0.031013757	0.583808	554	417	471	263	584	276
Hsd17b11	0.285246	6.766474	4.957208	0.025982132	0.535909	702	461	482	300	677	302
Tmed9	0.282978	7.646833	6.444173	0.011131686	0.33451	3562	2250	3061	1805	3478	1549
Rnh1	0.279904	7.434222	4.67782	0.030554498	0.580381	3877	2498	3196	1843	3751	1751
H13	0.270527	6.832867	4.29309	0.038267566	0.646227	1198	917	846	626	1151	497
Ccpg1	0.259664	6.455142	4.505848	0.03377915	0.608704	895	540	730	444	818	369
Anxa5	0.259504	8.503199	4.338679	0.037255812	0.639741	2075	1389	1546	830	1979	990
Eif1	0.25046	7.96032	4.963291	0.025890882	0.534862	2562	1851	2046	1378	2450	1050
Aprt	0.250031	6.449095	3.979654	0.046053021	0.703111	1138	927	1051	602	1098	613
Ccni	0.249401	7.271012	4.67527	0.030599894	0.580381	1416	1050	1348	728	1474	686
Ckap4	0.240238	7.856084	4.012426	0.045166117	0.700601	5589	3588	4535	2711	5031	2406
Rtn4	0.239339	9.160383	3.930177	0.047426944	0.711107	1705	1011	1337	715	1636	707
Pgd	0.223017	7.008613	3.995676	0.045617145	0.702576	2549	1790	2149	1213	2559	1090
**Downregulated genes**											
Zbtb10	-1.09129	3.80134	11.47546	0.000705211	0.064294	98	50	60	25	23	11
Gab3	-1.09113	2.396895	4.919374	0.026557135	0.542261	33	23	24	5	10	8
Ptpru	-1.08971	2.503395	5.663925	0.017317326	0.438869	36	23	40	8	20	3
Rhof	-1.0771	3.548735	4.289177	0.038355721	0.646227	23	5	34	6	8	4
Pcdhga3	-1.07208	2.270885	4.294534	0.038235069	0.646227	76	43	26	14	12	13
Necab1	-1.06537	5.203775	16.80892	4.13386E-05	0.008479	437	394	467	169	137	68
Fxyd4	-1.06509	2.86854	5.368552	0.020502993	0.479255	270	204	206	99	48	36
Dok4	-1.06144	4.23792	10.0729	0.001504659	0.101287	75	79	65	34	21	9
Snx31	-1.04517	3.163254	6.64376	0.009950372	0.31577	330	236	277	112	91	41
Med18	-1.04427	2.458836	5.44239	0.019653758	0.467777	91	75	68	36	30	5
Gspt2	-1.04366	3.717762	9.025766	0.002662003	0.149155	49	52	50	12	34	4
Rimbp2	-1.0319	4.485034	7.668737	0.005618577	0.232403	24	28	64	11	16	9
Smyd4	-1.0114	2.772638	4.831169	0.027949667	0.555277	140	61	62	32	18	21
Dpy19l3	-1.01063	3.058532	6.780439	0.009216224	0.304866	133	134	145	28	56	42
Plagl1	-1.01019	4.466814	5.773129	0.016273034	0.420306	17	19	26	8	5	6
Dclk3	-0.99624	4.264692	5.923704	0.01493852	0.402223	33	32	18	0	17	10
Dnalc1	-0.99534	3.479023	7.966263	0.004765724	0.211858	70	95	76	14	42	23

Mctp1	-0.98155	4.968533	6.454282	0.011068529	0.333966	116	51	141	50	31	11
C030030A(	-0.97907	4.20476	8.449999	0.003650436	0.180514	51	33	65	23	19	5
Stfa1	-0.97255	2.91781	5.375731	0.020418786	0.479255	277	224	197	30	164	54
Arhgap31	-0.96966	3.874854	8.384355	0.003784644	0.184232	41	41	37	9	16	13
Sycp3	-0.96932	3.448308	6.466166	0.010994751	0.333966	37	37	38	15	19	3
Klhl23	-0.96708	3.040284	6.75947	0.009325143	0.304924	88	58	91	32	32	11
Mrgpre	-0.96681	3.550541	9.498601	0.002056286	0.125775	62	50	50	19	27	7
Slc25a33	-0.96146	4.310894	10.90593	0.000958568	0.075755	57	41	50	11	29	9
3110021A1	-0.95937	1.936831	4.092109	0.043083859	0.68529	68	94	61	17	54	11
Ryr2	-0.95631	4.845388	5.105071	0.023856007	0.516307	12	23	23	12	7	0
4930473A(	-0.95403	2.986069	8.776154	0.00305194	0.16431	93	81	90	26	51	13
Pcdh9	-0.95125	7.097144	5.092175	0.024034024	0.518152	30	5	58	8	22	2
Ctnnd2	-0.94804	5.165916	8.954302	0.00276817	0.153505	46	26	33	11	15	8
Tmem201	-0.94446	3.184417	5.483618	0.019195484	0.460949	110	49	88	12	45	24
3110062M	-0.93668	2.573116	3.983505	0.04594786	0.703111	66	19	34	14	24	2
Kctd4	-0.93182	2.269698	4.10666	0.042714634	0.684325	47	19	47	11	13	11
Rassf8	-0.9287	2.373811	4.006546	0.045323917	0.700601	91	46	33	19	17	15
Slc13a2	-0.92751	3.677539	5.576975	0.018198125	0.449026	441	397	345	182	128	60
Gm88	-0.92536	3.700579	4.841918	0.027775981	0.552847	33	18	13	8	10	3
D7Ertd443	-0.92228	2.823232	4.425264	0.035410638	0.625721	223	133	244	53	107	43
Sncg	-0.922	9.917157	4.877011	0.027216704	0.547313	10	12	25	1	14	1
Sept4	-0.92042	5.257279	6.221398	0.01262154	0.359213	30	18	28	9	10	6
Galnt14	-0.91948	4.022379	4.542098	0.033071043	0.605326	33	26	11	8	10	5
Chst2	-0.91543	5.591271	14.61349	0.000131967	0.020138	180	97	139	56	65	17
Trmt61a	-0.91451	3.095241	5.289726	0.021451599	0.489181	90	133	65	32	58	14
Mtap9	-0.91173	5.902839	17.00668	3.72486E-05	0.008194	348	313	236	110	94	77
Tox	-0.90049	4.190932	14.62106	0.000131437	0.020138	411	325	330	121	181	63
8430427H1	-0.89282	3.840171	4.910323	0.026696641	0.542916	41	27	18	10	14	5
Ntm	-0.88866	4.037727	4.800275	0.028455191	0.560148	34	16	17	6	14	3
Bend7	-0.8816	3.869089	11.94148	0.00054898	0.053342	212	231	160	62	114	39
Foxp2	-0.87626	3.368451	5.071277	0.024325458	0.520783	28	29	49	12	18	7
Prrx2	-0.86488	2.299044	5.345575	0.020774931	0.482194	128	98	103	30	63	24
Pcgf6	-0.85928	2.669779	4.48414	0.034210762	0.613911	130	98	118	29	91	15
Rimklb	-0.85921	3.632032	7.299721	0.006896534	0.259331	160	110	122	61	53	18
Garnl3	-0.84658	4.859442	7.84137	0.00510639	0.221455	56	35	66	24	17	12
Prkar1b	-0.84588	8.120975	13.6818	0.000216543	0.028237	75	55	62	14	44	11
Pex3	-0.83302	4.476196	14.06554	0.000176549	0.024666	346	199	271	93	124	63
Prss36	-0.83114	2.842386	4.710267	0.029983001	0.574762	76	89	97	22	57	20
Tmem199	-0.83103	4.566329	10.88711	0.000968358	0.075764	134	175	189	58	75	43
Zfp770	-0.829	3.631835	6.102766	0.013497048	0.376391	79	41	97	18	50	13
Hcfc2	-0.8285	2.88612	4.709827	0.029990661	0.574762	204	151	141	44	80	48
Disp1	-0.8239	4.048061	12.5087	0.000405062	0.043646	241	182	238	67	106	59
2900060B1	-0.82377	7.771424	9.661824	0.001881367	0.119762	3117	4347	3407	1640	1412	717
Trib3	-0.80866	3.29283	6.810947	0.009060072	0.301759	232	248	240	85	111	60
Slc15a2	-0.80682	3.999406	5.694216	0.017020917	0.433912	310	214	462	116	199	56
Rbfox1	-0.80478	7.626104	16.93006	3.87825E-05	0.00823	154	80	103	28	70	25
Iqgap2	-0.80356	4.192817	3.952621	0.046798426	0.705555	231	81	102	71	29	27
Ttc36	-0.80356	3.794955	5.086107	0.024118273	0.51857	390	440	404	183	179	77
Fastkd2	-0.79045	3.617737	9.495792	0.002059436	0.125775	129	101	152	42	73	27
Lect1	-0.78809	4.233472	6.475335	0.01093817	0.333351	568	335	382	153	297	52
Dcbld1	-0.77951	3.787546	7.045553	0.00794623	0.280741	133	95	72	31	39	34
Exosc2	-0.77262	3.372819	8.704559	0.003174152	0.166951	178	142	112	48	76	35
Mical2	-0.76944	3.753837	7.92477	0.004876254	0.215291	154	202	177	49	118	43
Carns1	-0.76866	4.982617	6.048746	0.013916178	0.382575	35	55	50	7	23	22
Sgip1	-0.76814	4.140561	4.641819	0.031201919	0.585565	65	18	46	15	24	8
4930430F(	-0.76071	3.046981	4.329076	0.037466569	0.642335	82	68	68	21	61	8
Hibch	-0.75933	2.857743	4.434858	0.035212148	0.625103	132	54	99	24	64	21
Gm347	-0.75899	3.482508	6.835707	0.008935333	0.298993	165	116	117	55	70	23
Arhgap39	-0.74865	4.590903	7.761872	0.005336062	0.224412	97	69	99	21	63	20
Stat5a	-0.73937	2.98387	5.176833	0.022890013	0.506052	138	100	184	35	85	42
Zdhhc2	-0.73548	6.993401	4.6693	0.03070644	0.580562	42	18	16	3	9	16
Mtr	-0.73128	3.579645	6.079273	0.013677707	0.378264	215	131	177	51	104	45
Slc25a19	-0.72814	4.230325	6.981152	0.008237257	0.287118	158	101	118	55	48	32
Ntrk3	-0.71472	5.153132	5.396129	0.020181464	0.476123	66	29	30	11	22	14
Hunk	-0.71332	3.907793	7.32274	0.006808752	0.25773	360	369	308	138	155	93

Med12l	-0.70908	3.56246	4.215762	0.04005007	0.662225	59	56	32	20	33	6
Zfp275	-0.70268	3.960277	7.782694	0.005274907	0.223293	131	107	100	32	75	28
Snord89	-0.69991	6.517628	6.89129	0.008661674	0.29395	704	1247	734	347	541	209
Exosc1	-0.69885	4.256425	5.407539	0.02004996	0.47406	350	341	186	129	125	72
H2-Aa	-0.69583	5.579804	6.397852	0.011425852	0.339574	351	272	178	88	210	41
Pcsk6	-0.69248	3.907196	6.528546	0.010615667	0.326762	199	141	125	54	107	28
Abhd10	-0.68617	4.487208	7.703018	0.005512857	0.23002	94	105	87	22	64	30
Cbwd1	-0.68305	3.228484	4.871111	0.027309906	0.548134	123	108	111	28	77	34
Haus3	-0.67999	3.265228	4.254212	0.039153104	0.654359	116	87	102	24	86	21
Enpp4	-0.67936	5.246512	13.46143	0.000243519	0.030462	441	274	425	120	254	88
Trpv4	-0.67818	3.424873	4.575253	0.032436959	0.598041	339	220	322	96	214	58
Nop56	-0.67552	5.280878	11.0513	0.000886251	0.073909	554	475	453	208	269	107
Sumf2	-0.67546	2.828481	4.719513	0.029822188	0.573757	96	80	74	22	52	26
Rad1	-0.67496	3.673047	3.981991	0.04598916	0.703111	114	77	180	58	55	28
Mocs3	-0.67417	3.162306	4.301973	0.038068176	0.645449	155	133	157	51	99	33
Ptn	-0.66785	6.994023	13.7608	0.000207623	0.02763	146	129	119	31	85	43
Rap1gap2	-0.66783	5.020521	5.992997	0.014362782	0.390603	73	56	81	36	26	18
Dopey1	-0.66641	4.315416	4.694149	0.03026548	0.576787	235	243	219	99	80	76
Fam169a	-0.66629	4.979427	6.341753	0.011792826	0.345217	995	466	1006	298	338	261
Acyp1	-0.66618	3.475723	4.409241	0.03574475	0.629365	100	42	105	31	42	22
Mtap1b	-0.66345	11.53992	8.645791	0.003278177	0.169785	246	154	153	91	83	37
Kbtbd11	-0.66228	5.047356	7.536108	0.006047455	0.243665	440	181	357	126	145	92
Creg2	-0.66066	4.042184	4.380707	0.036347922	0.631987	89	159	113	35	98	27
Mageh1	-0.65962	4.018137	5.103331	0.023879949	0.516307	108	109	116	39	83	20
Cntf	-0.65938	5.676814	6.733539	0.00946167	0.307519	107	122	58	37	55	24
Ankrd13b	-0.65911	4.483775	5.333754	0.020916282	0.482801	72	86	49	17	39	27
Epb4.1l3	-0.65762	7.354412	7.04847	0.0079333	0.280741	55	46	56	17	23	22
Zfp553	-0.65491	3.756508	6.457503	0.011048482	0.333966	249	142	192	66	102	58
Ahcyl2	-0.65407	4.486229	5.804911	0.015981472	0.416022	159	128	93	52	62	34
Rnaseh2a	-0.6511	4.392115	8.207707	0.004171283	0.196819	349	252	281	98	198	69
Tmem69	-0.65052	3.46995	5.55834	0.018392843	0.450217	159	149	117	55	80	36
Akr1b7	-0.64864	5.812455	6.815765	0.009035661	0.301413	1895	1607	1426	790	750	361
Spnb1	-0.6462	6.019232	7.753127	0.005361962	0.224783	118	143	84	43	61	35
Kctd12	-0.64366	5.214055	8.818371	0.002982127	0.162029	1056	858	847	418	442	216
Gpld1	-0.64364	3.62173	5.150564	0.023238818	0.509691	234	196	186	68	115	63
Zc3h10	-0.64138	4.11077	6.414466	0.011319435	0.338262	256	145	203	65	140	46
Pkig	-0.64024	3.773579	4.692127	0.030301109	0.576955	213	117	174	64	109	34
Ccdc137	-0.63862	5.337572	9.602643	0.001942975	0.121526	356	407	364	130	227	108
Ccnd2	-0.63773	7.778711	8.977999	0.002732498	0.152644	5587	5338	5799	2560	2183	1564
Abcg2	-0.63753	5.450239	14.12774	0.000170806	0.024178	587	311	431	153	257	120
Ppp1r9a	-0.63326	5.9254	4.0819	0.043344898	0.686246	39	49	46	31	11	10
Rpusd1	-0.63187	4.168893	4.820322	0.028126098	0.557239	166	105	75	42	48	41
Peg13	-0.62927	6.769945	8.008527	0.004655759	0.209553	119	165	121	56	72	38
C630004H(	-0.62906	5.997179	5.65046	0.01745079	0.439849	51	64	87	15	46	24
Inpp5b	-0.62778	3.440159	3.860866	0.04942481	0.722435	125	96	130	33	104	21
Ecsit	-0.62681	4.008974	6.85504	0.008839156	0.296698	127	137	149	45	96	35
Fam82a2	-0.62031	4.980953	12.25523	0.000463955	0.04776	479	424	420	142	275	132
Zmynd19	-0.6198	3.654208	5.82942	0.015760321	0.415865	201	129	193	69	119	33
Hps6	-0.61763	3.439109	4.21055	0.040173302	0.662225	177	122	105	60	51	42
A830010M	-0.61455	4.582073	4.184302	0.040800004	0.667569	65	89	69	11	57	29
Noc4l	-0.60759	3.684036	4.807794	0.028331293	0.559691	203	199	227	75	142	53
5730494N(	-0.60293	4.738683	6.725331	0.009505309	0.308471	334	186	283	76	224	57
Zfp423	-0.59829	4.026582	4.107786	0.042686217	0.684325	88	50	60	22	38	21
Dusp16	-0.59731	4.653386	6.566604	0.010391003	0.323446	521	434	422	169	286	124
A730011L(	-0.59675	2.963552	4.006464	0.045326122	0.700601	108	83	88	41	59	18
Scrn3	-0.59482	3.993287	3.962968	0.04651163	0.704916	113	74	96	42	54	21
Kcnb1	-0.59334	6.467244	4.518464	0.03353092	0.607346	39	32	37	16	18	11
Osgin2	-0.58833	3.616141	5.030229	0.024908611	0.526975	155	161	198	54	131	44
Xk	-0.5872	3.633316	3.870924	0.049129477	0.720347	109	44	86	26	50	23
Eid2b	-0.58306	3.582221	5.389396	0.02025948	0.476918	119	87	101	38	62	29
Emb	-0.58272	6.346106	3.937432	0.0472228	0.709034	122	213	336	90	113	76
Ssfa2	-0.58103	4.698749	6.047947	0.013922478	0.382575	538	332	496	192	273	107
Tppp3	-0.58012	10.12613	5.907249	0.015078701	0.403524	574	464	309	237	204	99
Homer1	-0.57445	4.236784	5.023349	0.025007756	0.527	230	181	234	89	116	62
Defb1	-0.57216	6.52184	10.76684	0.001033348	0.079405	3187	2131	2665	1110	1639	634

Alg2	-0.57014	5.183794	6.77456	0.009246631	0.304866	162	202	172	70	94	61
Khdrbs3	-0.56917	5.145744	4.9955	0.025413317	0.529416	120	115	91	43	44	44
Mapk8ip3	-0.56914	6.693105	4.247824	0.039300645	0.655094	101	81	84	59	27	19
Taf7	-0.56652	4.59969	5.014081	0.025141974	0.527777	241	271	219	113	118	71
Csrp2	-0.56329	4.863159	6.055193	0.013865463	0.381984	199	130	230	72	138	38
1810012P1	-0.56305	4.704699	6.8983	0.008627775	0.293727	300	283	297	131	170	74
Sorcs3	-0.56219	4.225789	4.402212	0.035892341	0.62938	300	140	161	60	165	44
Ubr7	-0.55169	4.30342	4.802711	0.028414997	0.559869	439	253	300	127	261	61
Ptges2	-0.55138	4.687549	6.610275	0.010139198	0.319407	297	248	244	83	152	98
Zfp955b	-0.54874	4.165696	4.536423	0.033180864	0.605326	197	197	183	82	123	48
Ephb1	-0.54656	4.615915	4.350217	0.037004232	0.636946	359	272	392	162	194	80
Osbpl6	-0.54606	6.106046	9.501843	0.002052657	0.125775	1104	767	1107	379	601	292
Smpd2	-0.54423	3.786111	4.635711	0.031313171	0.585856	287	169	248	94	142	64
Slc25a15	-0.54261	3.831516	4.075918	0.043498615	0.68748	215	223	195	99	148	39
1110038D1	-0.54235	3.648119	5.18757	0.022749005	0.504086	265	160	214	81	157	47
Mrto4	-0.54131	4.676521	4.170161	0.041141888	0.670105	345	353	321	153	201	88
BC017647	-0.52452	5.623682	9.573375	0.001974196	0.122765	666	469	558	195	413	155
Arsk	-0.52444	4.354997	4.557446	0.032775918	0.602741	478	254	404	160	223	102
Enpp2	-0.51926	5.904891	4.369691	0.036583634	0.634251	66	53	57	20	48	12
Tmem47	-0.51819	5.85499	4.877054	0.027216031	0.547313	131	67	67	37	45	30
Rilpl1	-0.51776	4.327068	5.281062	0.02155859	0.489635	216	127	146	60	94	55
Cnst	-0.51711	5.716501	8.700428	0.003181354	0.166951	372	245	280	100	206	92
C230091D0	-0.51535	4.556994	5.896554	0.015170538	0.405477	253	211	184	68	185	52
Mrpl38	-0.51095	4.763801	7.821463	0.005162938	0.222943	310	222	269	78	194	89
Fam53a	-0.50725	3.962075	4.140084	0.041879096	0.676079	363	234	361	105	198	114
Plce1	-0.5031	5.474843	8.010349	0.004651076	0.209553	875	572	601	305	435	166
Pth1r	-0.49952	4.727157	3.945795	0.04698866	0.706011	155	180	213	71	145	44
Adcy2	-0.49754	4.181775	4.527383	0.0333566	0.606248	127	115	101	45	85	28
Asb1	-0.49723	4.994026	4.44163	0.035072746	0.624144	124	107	78	24	86	34
Mboat2	-0.49642	5.80684	5.511218	0.018894881	0.457818	94	80	118	39	65	28
5133401N0	-0.49634	5.112013	5.528747	0.018706497	0.454277	347	314	401	125	267	102
Ogfod1	-0.49413	4.719087	4.867523	0.02736676	0.548388	367	209	227	86	219	69
Tomm34	-0.4939	6.074018	6.871564	0.008757796	0.295349	649	414	485	213	335	141
Irak2	-0.49242	5.817524	5.6681	0.017276162	0.438869	1577	1334	1569	472	1137	477
Clip2	-0.49192	4.917138	4.918952	0.026563622	0.542261	138	74	134	48	71	34
Llph	-0.49091	4.967913	5.168325	0.023002381	0.506052	794	467	589	248	417	165
Bcl7a	-0.48812	6.093438	11.38912	0.000738757	0.066786	1567	929	1176	453	894	332
Pla2g7	-0.48752	6.477034	6.421251	0.011276269	0.337441	236	277	226	118	168	56
Pdhb	-0.48752	5.392331	7.795572	0.005237442	0.222943	393	356	293	132	215	118
Ddx41	-0.48738	4.448375	6.281066	0.01220347	0.353391	496	349	298	149	253	111
Ccdc101	-0.48685	4.048795	4.087991	0.043188956	0.68529	179	147	119	64	90	44
Mphosph1	-0.48537	5.04674	4.821048	0.028114244	0.557239	570	447	467	220	359	110
Dhx29	-0.48529	4.307655	3.969116	0.046342084	0.704662	330	290	248	107	201	89
BC046331	-0.48291	4.426921	5.259935	0.021821836	0.492158	268	169	169	74	133	64
Abca8a	-0.48079	6.98967	4.725051	0.029726307	0.573757	761	832	918	465	485	182
Cdkal1	-0.47624	4.56705	4.552322	0.032874136	0.603145	482	393	452	164	339	122
Morn2	-0.47566	4.743193	4.879986	0.027169846	0.547313	261	290	280	80	212	100
Dkk3	-0.47136	7.149317	13.42746	0.000247967	0.030791	3025	2387	2623	1071	1991	709
Tsc22d2	-0.47058	4.327561	3.898082	0.048341253	0.718364	391	273	197	108	156	108
Ccdc112	-0.46794	4.414842	4.247555	0.039306879	0.655094	190	218	214	74	154	66
Nrxn1	-0.46743	7.569895	12.03119	0.000523176	0.0514	1277	916	1209	457	856	288
Gsta3	-0.46653	5.651377	5.053839	0.024571435	0.525004	1622	1192	1362	651	860	369
Gnl3	-0.46566	5.293803	4.967224	0.025832068	0.534426	520	476	502	264	276	128
Sox9	-0.46496	6.356966	7.649412	0.005679082	0.232745	1874	1123	1817	593	1299	408
Crabp1	-0.46306	4.290561	4.05892	0.043938582	0.690382	525	339	420	153	258	157
Mfge8	-0.45943	6.743611	7.20433	0.007272789	0.266578	2625	2232	2026	919	1405	771
Ift88	-0.45822	4.291409	4.088893	0.043165912	0.68529	256	216	262	101	173	69
Stox2	-0.45694	6.075462	6.711934	0.009576979	0.309862	355	267	232	98	249	68
Ap1ar	-0.45472	5.125992	6.644977	0.009943575	0.31577	635	456	540	227	311	186
Npr3	-0.45228	5.0206	6.607347	0.010155883	0.319465	959	736	668	320	488	259
Kitl	-0.45108	5.725235	6.960351	0.008333566	0.287118	390	230	238	111	205	81
Ric8b	-0.44584	4.726257	4.297402	0.038170644	0.646168	178	112	122	61	87	40
Nvl	-0.44431	4.87248	5.481584	0.019217834	0.460971	589	408	452	211	333	130
Coq7	-0.44414	5.813339	7.363293	0.006656876	0.254404	555	470	442	219	289	157
Timp3	-0.44398	8.83514	11.31496	0.000768852	0.068441	4325	3554	3423	1655	2204	1222

Pwp1	-0.44081	4.521382	4.426017	0.035395012	0.625721	370	374	347	152	246	114
1700021F0	-0.4406	4.793548	5.124011	0.02359702	0.515307	342	261	311	132	211	85
Gtf2f2	-0.43704	4.864771	4.003859	0.045396204	0.70068	722	515	521	215	410	194
Trim37	-0.43624	7.530167	13.89995	0.000192804	0.025927	1449	996	1224	488	822	389
Arrdc4	-0.43447	4.9982	4.597201	0.032024197	0.593482	911	646	671	326	570	176
Tex2	-0.43121	5.89795	7.038467	0.007977729	0.280931	767	391	579	207	460	163
Mapre3	-0.43107	7.01317	6.704215	0.009618527	0.310273	206	233	232	74	185	70
Cdo1	-0.42838	8.322471	4.216601	0.040030283	0.662225	8977	8117	8485	4315	6098	1905
Gfod1	-0.42833	5.897391	6.6977	0.009653739	0.310478	354	359	342	154	235	109
Iffo2	-0.42791	5.581312	4.666885	0.030749659	0.580838	1537	1070	806	519	723	325
Rbms3	-0.42697	8.757206	5.387157	0.020285495	0.477008	553	584	605	290	297	196
Mpi	-0.42677	4.978704	4.058517	0.043949089	0.690382	270	189	206	56	166	92
Cryab	-0.42655	8.098118	5.74499	0.016535747	0.425578	2702	1768	2268	1192	1334	547
Polr3g	-0.42532	6.392551	7.392692	0.006548942	0.251852	1195	806	1034	492	629	276
Got1	-0.42521	6.383215	4.307722	0.037939724	0.644349	204	94	169	48	134	44
Rai14	-0.42461	4.448503	4.054079	0.044064747	0.691344	527	279	424	145	261	153
Pcsk2	-0.42414	7.113682	4.69493	0.03025173	0.576787	410	292	413	195	182	117
Lamb1	-0.42398	7.25249	9.356086	0.002222462	0.132461	2549	2362	2124	964	1708	707
Tanc2	-0.42284	5.676926	4.363636	0.036713863	0.634486	335	254	364	161	217	74
Ints4	-0.42194	4.913049	6.299306	0.012078526	0.350717	472	342	461	167	302	135
Gprc5c	-0.41991	5.502815	3.970391	0.046307009	0.704626	178	142	200	94	114	38
Lmbrd2	-0.41689	6.342418	9.271222	0.002327824	0.135357	761	669	623	267	479	230
Dkc1	-0.41611	6.947305	8.531982	0.003489595	0.176394	2532	1879	1923	982	1355	612
Mbd3	-0.4144	5.203956	5.606598	0.017892967	0.446595	590	436	461	203	406	124
Dnajc11	-0.41264	5.62893	6.009839	0.014226322	0.387951	833	743	767	241	562	314
Trappc4	-0.41247	6.535409	11.15346	0.000838752	0.07133	956	737	881	344	589	285
Pcnp	-0.41226	5.191309	4.354319	0.036915216	0.63679	408	318	269	106	264	114
Gnl1	-0.41176	5.967111	7.878835	0.00500167	0.218288	714	698	632	290	507	198
Tceal8	-0.41102	5.35472	4.746821	0.029352479	0.570504	548	362	593	205	322	172
Socs5	-0.40776	5.451421	4.090433	0.043126605	0.68529	242	203	220	102	111	87
AI316807	-0.40733	5.756797	8.229025	0.004122566	0.195377	1017	691	757	288	620	277
Elavl1	-0.40648	5.130523	4.187639	0.040719779	0.666763	747	432	540	259	411	149
Bcas1	-0.4052	7.200087	5.45118	0.019555109	0.466714	1278	892	918	502	652	287
Bok	-0.4033	5.37386	4.012825	0.045155425	0.700601	1132	948	833	414	781	253
Atp6v0e2	-0.40088	7.426143	5.80566	0.015974667	0.416022	244	297	275	99	217	90
Mrpl41	-0.39866	5.605653	5.061747	0.02445957	0.523133	440	317	357	159	256	116
Gkap1	-0.39836	5.898197	4.912402	0.026664543	0.542916	308	269	230	85	192	110
Krt78	-0.39816	5.368667	4.519423	0.033512132	0.607346	1283	972	1130	531	652	373
Ptpn3	-0.39691	4.911204	4.794252	0.028554855	0.561083	426	349	396	160	238	148
Aif1l	-0.39673	7.50744	6.482882	0.010891823	0.332409	4626	3219	3867	1767	2560	1191
Tmem64	-0.39631	5.564681	7.060372	0.007880762	0.279927	739	534	653	289	457	182
Oxr1	-0.39605	8.358467	9.647032	0.001896578	0.12002	7615	6181	6460	2932	4673	2105
Nceh1	-0.3958	5.858417	4.097824	0.042938438	0.684851	139	91	85	41	68	40
Bcl2	-0.39396	6.522888	9.449217	0.002112393	0.128029	1182	825	1021	388	835	285
Plcd3	-0.39374	4.037495	3.847053	0.049833492	0.725696	406	337	325	134	272	117
Tusc2	-0.39256	6.400867	7.228721	0.007174632	0.265002	560	443	431	182	313	183
Guk1	-0.389	6.733651	4.983715	0.025586976	0.531391	634	592	672	287	492	168
Opa3	-0.38831	5.694709	4.422054	0.035477304	0.625721	487	522	495	236	391	128
Prkag2	-0.38793	6.488367	4.867097	0.027373513	0.548388	609	427	619	194	381	214
Orc2	-0.38767	5.191826	5.808928	0.01594501	0.416022	617	580	538	243	417	187
Rhoj	-0.38636	6.140535	8.222904	0.004136495	0.195606	1353	1010	985	428	759	407
Ppip5k2	-0.38573	6.313646	6.862023	0.008804683	0.296002	661	528	561	278	373	181
Fbxo25	-0.38315	5.194657	4.278596	0.038595225	0.648255	326	274	245	111	226	84
Ppa1	-0.38069	6.851918	8.124662	0.004366712	0.201618	2269	1675	1711	822	1247	623
Utp20	-0.3805	4.547423	3.888983	0.048603858	0.719107	489	408	344	168	308	133
Dnaja1	-0.38033	7.200164	6.565749	0.010395996	0.323446	641	405	588	214	347	208
Col8a1	-0.37985	7.010475	7.838179	0.005115413	0.221455	3539	2471	3609	1236	2302	1118
Arrdc3	-0.37553	5.68333	4.092471	0.043074637	0.68529	1340	833	1056	472	761	326
Gfra1	-0.37155	6.173338	4.323986	0.037578797	0.642723	475	423	448	234	322	110
Gstm1	-0.3689	5.816045	6.963173	0.008320435	0.287118	972	723	696	298	641	257
Mul1	-0.36776	5.2536	5.032488	0.024876131	0.526975	465	361	365	153	266	154
Acsm1	-0.36729	5.606395	4.248034	0.039295801	0.655094	1544	1236	1174	595	917	414
Eps15l1	-0.36688	5.702256	4.611423	0.031759676	0.591126	1062	689	967	349	626	333
Drosha	-0.36679	6.254033	7.148696	0.007501853	0.272652	952	859	752	363	629	277
Nf1	-0.36572	5.588376	4.008978	0.045258578	0.700601	485	442	384	152	320	173

Epas1	-0.36373	7.467466	5.751219	0.016477217	0.424579	4153	3611	3720	1881	2944	971
Nrxn2	-0.36257	8.157801	5.001985	0.025318271	0.528881	290	260	208	117	154	93
Cyp3a13	-0.36254	5.417346	4.462028	0.034656328	0.618296	1199	1010	1188	434	926	366
Wdr37	-0.36155	5.226326	4.306216	0.037973347	0.644349	329	208	251	106	214	75
Enc1	-0.35599	5.795773	4.47448	0.034404661	0.616362	1072	750	781	344	587	324
C80913	-0.35213	6.247666	5.585976	0.01810484	0.447237	1404	1087	1300	517	880	458
S100a13	-0.34921	6.458975	3.98639	0.045869246	0.703111	344	327	338	104	280	132
Igfbp4	-0.3492	7.396413	9.765906	0.001777779	0.115213	2482	1745	1936	851	1570	659
Tsc22d1	-0.34849	7.820361	6.225588	0.012591707	0.359026	2557	1751	1792	792	1708	619
Aldh1a1	-0.34789	5.593699	4.229773	0.03972076	0.658422	1247	883	1124	454	705	407
Ube2n	-0.34573	5.5057	3.926243	0.047538032	0.71178	268	191	204	84	161	83
Pard3b	-0.3417	5.478044	4.137278	0.041948572	0.676079	957	749	764	335	569	308
Gtpbp4	-0.33896	6.754136	3.900608	0.048268637	0.718364	1953	1692	1596	962	1129	483
Xpc	-0.33654	5.535071	5.010651	0.02519183	0.527777	1020	779	913	386	657	310
N6amt1	-0.33625	6.105209	5.353331	0.020682726	0.481091	646	467	568	248	425	174
Phyhip	-0.33607	6.255025	5.032628	0.024874123	0.526975	2106	1902	1812	730	1416	762
Usp46	-0.33313	5.831508	4.927581	0.026431298	0.541055	1218	814	908	435	722	311
Ftsj3	-0.33169	6.118092	5.203822	0.022537286	0.50146	1194	1130	910	483	839	336
Ciapin1	-0.33115	7.054633	6.762441	0.009309632	0.304924	1779	1010	1395	527	1026	517
D5Ertd579	-0.32843	6.279464	4.816466	0.028189082	0.557973	1033	826	962	436	704	290
Lancl1	-0.32699	6.793614	5.271884	0.021672546	0.489818	1632	1523	1621	668	1041	639
Polb	-0.3245	6.347949	4.663901	0.030803136	0.580859	1806	1264	1263	609	1075	492
Ivns1abp	-0.31838	7.464698	6.958916	0.008340255	0.287118	2917	2507	2841	1197	2240	867
Cpt1a	-0.31472	6.556703	5.336816	0.020879573	0.48254	1214	1071	1048	405	977	383
Ddah1	-0.31432	6.777014	4.383344	0.036291725	0.631987	2014	1544	1386	707	1259	555
Ngfrap1	-0.31295	6.142655	4.198856	0.040451254	0.665642	404	295	310	147	255	112
Sipa1l1	-0.31098	6.512046	6.621671	0.010074528	0.318317	1335	1090	1167	465	932	447
Ttc35	-0.3081	6.976146	5.206308	0.02250507	0.501262	1104	829	863	455	708	272
Cacybp	-0.30777	6.810982	4.490862	0.03407649	0.613035	1127	930	977	509	760	292
Hdac2	-0.30636	6.410725	3.919302	0.047734682	0.713236	984	760	739	359	551	322
Mllt6	-0.30548	5.596145	4.042323	0.044372712	0.69384	717	506	521	244	430	210
Gtf3c6	-0.30045	6.085371	3.90283	0.04820483	0.718364	1308	1107	1121	489	1010	379
Zbtb22	-0.29547	5.682917	4.67087	0.030678377	0.580542	854	655	769	329	567	273
Msh2	-0.29456	6.594596	5.843424	0.015635374	0.413789	1946	1473	1424	687	1302	539
Usp5	-0.2912	6.607972	4.583172	0.032287385	0.596817	749	627	809	281	577	282
BC003331	-0.29071	6.153512	4.158387	0.041428846	0.672742	630	521	492	256	401	188
D19Bwg13	-0.2882	6.683538	4.591794	0.032125377	0.594333	1456	1220	1172	604	1072	373
2610101N1	-0.28607	7.201223	5.196073	0.022637972	0.502143	2357	2098	2210	1063	1610	765
Pla2g16	-0.28593	7.913111	5.416287	0.019949738	0.47221	3959	2384	3175	1496	2391	1003
Mapre2	-0.28437	7.696151	5.085393	0.024128211	0.51857	1349	1043	1234	558	970	381
Kctd2	-0.28122	7.185638	4.209575	0.040196413	0.662225	1355	1119	1205	609	860	417
Tacc1	-0.28063	8.041243	5.393687	0.020209729	0.476268	3158	2239	2520	1260	1874	906
Sod1	-0.27598	8.904832	4.826335	0.028028159	0.556323	3951	3017	3600	1377	2955	1290
Mphosph6	-0.27547	6.714327	4.211067	0.040161064	0.662225	2255	1841	1788	903	1523	662
2410015M	-0.27389	6.195448	4.292451	0.038281939	0.646227	1440	1150	1285	571	1087	417
Pgrmc1	-0.27038	5.745484	3.921251	0.047679374	0.712904	955	661	750	341	685	245
Rpl7l1	-0.26981	5.982822	3.993138	0.045685897	0.703111	1154	955	1043	447	813	395
Zdhhc21	-0.26353	7.063315	4.537709	0.033155944	0.605326	1735	1319	1266	665	1080	510
B230208H1	-0.26229	7.723628	5.026087	0.024968255	0.527	2556	1978	2037	993	1589	823
Scpep1	-0.25654	6.343342	4.403982	0.035855109	0.62938	1657	1192	1399	596	1039	570
Hnrnpr	-0.2564	6.914024	3.859522	0.049464422	0.722523	2247	1938	1833	958	1562	681
Tmem30a	-0.2516	7.796615	4.058132	0.043959096	0.690382	1708	1187	1417	621	1256	468
Nsun2	-0.24167	6.738376	3.892608	0.048499052	0.719107	1645	1425	1329	591	1182	591
Syne1	-0.23231	7.19369	3.985825	0.045884622	0.703111	2548	2042	2136	964	1778	873
Napa	-0.23211	7.138979	3.960908	0.046568575	0.705116	1115	845	946	392	867	346
Dynll2	-0.22054	10.00512	4.196954	0.04049665	0.665642	7362	5745	5668	2566	5259	2448

Table S4. Differentially expressed genes in the TG of miR-183C SNS-CKO vs age- and sex-matched WT control mice.

	log2FC	logCPM	LR	PValue	FDR	WT1	WT2	WT3	WT4	SNS-CKO1	SNS-CKO2	SNS-CKO3
**Upregulated genes**												
Pdzd3	5.484182	-0.30227	8.793805	0.003022551	1	0	0	0	0	12	1	2
Fam166a	5.105066	-0.46727	7.702653	0.005513972	1	0	0	0	0	1	8	3
Gm34321	5.084223	-0.66844	5.702995	0.01693599	1	0	0	0	0	2	8	2
Gm34061	5.067769	-0.15628	7.998668	0.004681177	1	0	0	0	0	5	7	0
Gm35315	5.046036	-0.05644	12.15128	0.000490543	0.720657938	0	0	0	0	3	4	4
LOC10263	5.026891	-0.35503	6.130043	0.013290367	1	0	0	0	0	0	11	1
Gm6607	5.017329	-0.04648	13.51026	0.000237263	0.586926344	0	0	0	0	0	7	4
Slc25a21	4.989061	-0.37304	7.885905	0.004982154	1	0	0	0	0	3	6	2
Mirc35hg	4.975647	2.78916	77.6301	1.24262E-18	4.47045E-14	7	1	2	12	176	202	157
Gm29834	4.76388	-0.05256	6.874995	0.008740998	1	0	0	0	0	0	0	8
Alk	4.763251	0.997144	12.31975	0.00044819	0.705016671	0	0	0	0	1	4	4
Gm36273	4.752964	-0.42495	6.966731	0.008303905	1	0	0	0	0	2	4	3
Gm31637	4.672055	-0.79434	6.246886	0.012441182	1	0	0	0	0	4	5	0
Gm14167	4.647216	-0.62137	4.37672	0.036433039	1	0	0	0	0	3	6	0
D830044I1	4.632217	0.07486	7.453979	0.006329639	1	0	0	0	0	6	0	2
Gm29938	4.626876	-0.58117	4.443212	0.035040265	1	0	0	0	0	3	2	3
Msx2	4.555904	-0.02403	9.749721	0.0017935	1	0	0	0	0	5	3	0
Gm30701	4.537694	-0.84863	4.944719	0.026170485	1	0	0	0	0	2	0	5
1700113A	4.520951	0.022096	7.454506	0.006327783	1	0	0	0	0	0	1	6
Gm16252	4.498645	-0.54426	5.026307	0.024965081	1	0	0	0	0	0	2	5
Gm13630	4.484286	-0.75777	6.058468	0.013839766	1	0	0	0	0	2	6	0
Gm34669	4.480871	-0.77568	5.572848	0.018241073	1	0	0	0	0	2	6	0
Gm30732	4.46008	-0.38192	4.341127	0.037202286	1	0	0	0	0	5	0	2
Gm42357	4.431751	-0.48833	9.296978	0.002295321	1	0	0	0	0	0	3	4
Gm33800	4.382871	-0.68929	3.873374	0.049057799	1	0	0	0	0	6	1	0
Esr2	4.364763	-0.11236	8.281421	0.004005277	1	0	0	0	0	4	3	0
4930484H	4.349855	-0.7039	3.902243	0.04822168	1	0	0	0	0	1	0	5
Gm31336	4.34646	-0.86763	4.320321	0.037659814	1	0	0	0	0	1	0	5
Gm33464	4.345743	-0.86944	7.312267	0.006848548	1	0	0	0	0	1	5	1
Snord8	4.34037	-0.51605	4.020664	0.044946009	1	0	0	0	0	4	3	0
LOC10263	4.307981	-0.63932	5.580831	0.018158109	1	0	0	0	0	2	5	0
Gm30409	4.296802	-0.73801	4.015808	0.045075619	1	0	0	0	0	2	5	0
Ccdc36	4.29655	-0.63638	6.334324	0.011842322	1	0	0	0	0	1	6	0
Gm40302	4.295396	-0.70491	6.495954	0.010812028	1	0	0	0	0	2	0	4
LOC10816	4.288701	3.787921	20.33077	6.51445E-06	0.035299197	0	1	0	0	9	7	5
Gm39937	4.268488	-0.37173	5.562439	0.018349836	1	0	0	0	0	0	7	0
D830026I1	4.239594	-0.11479	7.679269	0.005585879	1	0	0	0	0	2	1	3
Tmem253	4.224081	-0.4414	4.881423	0.027147227	1	0	0	0	0	5	0	1
Klk11	4.205336	3.409408	9.797292	0.001747691	1	0	0	0	0	0	2	4
Lpar2	4.196506	2.846724	9.707706	0.001834969	1	0	0	0	0	6	0	0
Gm40652	4.175152	-0.61884	5.849578	0.015580785	1	0	0	0	0	5	1	0
Gm11627	4.169372	-0.267	6.179538	0.0129236	1	0	0	0	1	16	4	0
Gm35198	4.164626	-0.86939	4.874039	0.027263613	1	0	0	0	0	0	4	2
Morf4l1-p	4.152932	1.237727	16.31236	5.37123E-05	0.214706137	0	0	0	1	5	7	7
Gm41694	4.119871	-0.42605	4.162	0.041340568	1	0	0	0	0	0	0	5
Gm34299	4.114654	-0.6216	4.513222	0.03363382	1	0	0	0	0	0	0	5
Alx3	4.107202	-0.84973	4.169502	0.041157907	1	0	0	0	0	0	5	1
A330076C	4.104094	-0.86935	4.793748	0.028563206	1	0	0	0	0	2	4	0
Gm34180	4.098434	-0.7246	4.108864	0.042659009	1	0	0	0	0	2	4	0
LOC10816	4.09419	0.183286	5.803682	0.015992648	1	0	0	0	0	1	5	0
Gm34050	4.083373	-0.68995	4.966738	0.025839342	1	0	0	0	0	1	5	0
Gm40768	4.080365	-0.43687	4.703569	0.030100049	1	0	0	0	0	1	5	0
Raet1e	4.066448	-0.05163	5.324173	0.021031584	1	0	0	0	0	0	6	0
Gm32934	4.054173	-0.62704	4.460853	0.034680171	1	0	0	0	0	0	6	0
C630031E	4.050633	-0.75641	4.247166	0.039315887	1	0	0	0	0	2	0	3
Recql4	4.05005	-0.49546	4.308568	0.03792087	1	0	0	0	0	2	0	3
Gm32672	4.048377	-0.88775	4.478348	0.034326893	1	0	0	0	0	2	0	3
Myo18b	4.04801	-0.55463	4.514288	0.033612883	1	0	0	0	0	2	0	3
Ces1a	4.044787	-0.72058	4.81297	0.02824633	1	0	0	0	0	2	0	3
Gm19424	3.986429	-0.68749	5.925926	0.014919695	1	0	0	0	0	0	2	3

Myog	3.959493	-0.81249	6.089242	0.013600743	1	0	0	0	0	1	2	2
Hapln3	3.95454	0.010572	6.050038	0.013905998	1	0	0	0	0	3	1	1
Stx19	3.952392	2.474292	8.256186	0.004061337	1	0	0	0	0	2	1	2
Gm40338	3.950519	-0.31209	8.531515	0.00349049	1	0	1	0	0	6	9	2
Gm41377	3.947613	-0.32454	4.51073	0.033682865	1	0	0	0	0	5	0	0
Gm35596	3.938481	0.342563	7.327604	0.006790352	1	0	0	0	0	4	1	0
Gm34319	3.931691	-0.79438	5.034855	0.02484217	1	0	0	0	0	2	2	1
Vwa2	3.926627	-0.54089	5.43603	0.019725453	1	0	0	0	0	4	1	0
Tmem139	3.924264	2.492157	7.987508	0.004710119	1	0	0	0	0	0	5	0
Gm42009	3.904925	-0.68833	4.495522	0.03398373	1	0	0	0	0	1	3	1
Gm30325	3.881204	-0.77683	4.680001	0.030515728	1	0	0	0	0	0	4	1
Gm40446	3.880435	-0.85013	4.596105	0.032044675	1	0	0	0	0	0	4	1
BB557941	3.867142	-0.81265	4.059694	0.043918466	1	0	0	0	0	2	3	0
Gm32133	3.865338	-0.72268	3.867324	0.049234973	1	0	0	0	0	2	3	0
Gm40842	3.854154	0.411353	5.955111	0.014674679	1	0	0	0	0	0	5	0
Cpa6	3.839011	-0.68843	3.954793	0.046738055	1	0	0	0	0	1	4	0
LOC10524	3.806537	-0.49969	3.961662	0.046547711	1	0	0	0	0	0	0	4
Gm33234	3.791076	-0.65052	4.561173	0.032704662	1	0	0	0	0	0	0	4
Gm15658	3.767772	-0.04136	5.220821	0.022318014	1	0	0	0	0	0	0	4
9330155M	3.761425	0.360808	6.986728	0.008211635	1	0	0	0	1	4	8	3
Gm40754	3.727737	-0.51121	3.943352	0.047056936	1	0	0	0	0	2	0	2
Gm30314	3.691955	-0.09023	4.545055	0.033013956	1	0	0	0	0	1	1	2
Kcna3	3.687818	-0.8878	4.038263	0.044479582	1	0	0	0	0	3	0	1
Gm33108	3.680804	-0.62444	5.397698	0.020163331	1	0	0	0	0	1	1	2
Tnnt2	3.671485	1.598315	6.377377	0.011558414	1	0	0	0	0	0	1	3
Gm2511	3.666857	-0.84964	4.134811	0.042009759	1	0	0	0	0	0	2	2
LOC10263	3.66255	1.035826	6.313957	0.011979119	1	0	0	0	0	1	1	2
Fndc7	3.65696	-0.72147	4.8494	0.027655751	1	0	0	0	0	2	1	1
Gm40187	3.656508	-0.53119	5.04976	0.024629356	1	0	0	0	0	2	1	1
Agtr2	3.650797	0.35032	4.903822	0.02679733	1	0	0	0	0	4	0	0
Stpg2	3.649808	-0.22959	4.225902	0.039811443	1	0	0	0	0	4	0	0
Ano9	3.649759	3.633016	6.629393	0.010030946	1	0	0	0	0	4	0	0
Gm39534	3.628988	-0.86971	5.097833	0.02395576	1	0	0	0	0	3	1	0
Gm39114	3.626749	-0.48402	4.851344	0.027624601	1	0	0	0	0	3	1	0
Krt90	3.606951	0.990901	5.74632	0.016523232	1	0	0	0	0	1	3	0
Slc10a4	3.598938	-0.60964	3.921589	0.047669815	1	0	0	0	0	0	3	1
Lmod3	3.598601	-0.5337	3.884238	0.04874138	1	0	0	0	0	0	3	1
Gm33433	3.598122	-0.88887	4.513556	0.033627256	1	0	0	0	0	2	2	0
BC051408	3.593609	-0.49616	6.750474	0.009372279	1	0	0	1	0	5	5	3
Gm31268	3.533882	-0.34755	4.000669	0.045482195	1	0	0	0	0	0	4	0
Fgf21	3.53093	-0.70585	3.88478	0.048725647	1	0	0	0	0	0	4	0
Zfp947	3.507429	0.8284	8.961195	0.002757744	1	0	0	1	0	2	5	5
Hpn	3.501319	-0.42256	4.775928	0.02886029	1	1	0	0	0	0	11	2
Mat1a	3.390803	-0.4223	3.87804	0.048921638	1	0	0	0	0	0	0	3
Rasal1	3.381824	-0.0424	4.037576	0.044497684	1	0	0	0	0	0	0	3
Zpbp	3.367883	-0.08478	5.14104	0.023366647	1	1	0	0	0	2	10	0
LOC10264	3.339045	0.167092	8.5439	0.003466821	1	1	1	0	0	2	3	12
Gm30908	3.330536	-0.86831	4.313467	0.037811836	1	0	0	0	0	1	0	2
Gm30021	3.309087	1.484964	4.852245	0.027610167	1	0	0	0	0	0	0	3
9230117E	3.303253	-0.6885	4.219154	0.03997009	1	0	0	0	0	2	0	1
Cldn3	3.286932	3.599401	5.004017	0.025288567	1	0	0	0	0	0	0	3
Ces2e	3.282403	1.639056	4.889735	0.02701683	1	0	0	0	0	0	1	2
Otx1	3.275964	6.584711	5.069471	0.024350819	1	0	0	0	0	0	0	3
Hsd17b2	3.274109	5.635105	5.071143	0.024327338	1	0	0	0	0	1	0	2
Adgrf1	3.27366	5.385168	5.058179	0.024509974	1	0	0	0	0	0	1	2
Majin	3.273396	1.081752	4.799191	0.028473111	1	0	0	0	0	3	0	0
Wnt2	3.27212	3.713093	5.037166	0.024809044	1	0	0	0	0	3	0	0
Spink5	3.266716	6.179182	4.875775	0.027236213	1	0	0	0	0	0	2	1
Kcnj15	3.264777	1.303831	4.779043	0.028808124	1	0	0	0	0	0	2	1
Gm38850	3.263174	2.216487	4.693023	0.030285314	1	0	0	0	0	0	2	1
Gm8942	3.261824	-0.44209	4.61589	0.031677051	1	0	0	0	0	0	2	1
Gm31984	3.252489	-0.32946	3.913007	0.047913788	1	0	0	0	0	0	2	1
LOC10816	3.251997	-0.02334	4.228218	0.039757159	1	0	0	0	0	2	1	0
Kif18b	3.2477	1.037344	4.621143	0.031580188	1	0	0	0	0	1	2	0
Gm36043	3.247685	2.438517	4.781583	0.028765661	1	0	0	0	0	0	3	0
Gm10653	3.220702	-0.38268	3.920675	0.047695734	1	0	0	0	0	1	2	0

Cdc25c	3.181601	1.704399	7.770079	0.005311873	1	0	1	0	0	0	9	1
Sorbs2os	3.157936	0.873847	7.553473	0.005989454	1	1	0	0	0	1	9	0
LOC10004!	3.13523	-0.09232	4.849296	0.027657413	1	0	0	1	0	3	2	4
Grhl2	3.10056	5.435743	21.30568	3.9157E-06	0.028174261	1	1	0	1	3	16	3
Gm36243	3.098184	0.347617	4.882112	0.027136394	1	0	1	0	0	1	5	3
LOC10816!	3.078841	-0.52466	3.90467	0.048152071	1	1	0	0	0	4	3	2
LOC10816	2.912178	4.436268	7.838657	0.005114059	1	0	0	0	1	1	7	0
Speer4b	2.90666	-0.70405	4.826485	0.028025713	1	1	0	0	0	2	4	2
Gm35314	2.900795	-0.3485	3.908378	0.048045942	1	0	0	1	0	4	3	1
Gm33309	2.890968	-0.29963	4.50746	0.033747328	1	0	2	1	0	12	6	1
Gm41561	2.857593	-0.56114	4.167688	0.041201993	1	1	0	0	1	1	11	2
Ccdc168	2.854559	0.256622	5.215699	0.022383843	1	0	1	1	0	7	4	2
Rps15a-ps	2.854214	1.78742	9.99575	0.001569019	1	1	1	0	0	5	5	3
Mss51	2.851988	0.438299	8.942574	0.002785998	1	2	0	1	0	10	8	1
Gm28651	2.827089	-0.40885	3.966466	0.046415099	1	0	0	1	0	3	0	4
9230116N	2.775573	0.413311	3.945975	0.046983623	1	1	1	0	0	2	0	9
LOC10816!	2.758912	0.388746	5.209846	0.022459318	1	0	0	0	1	6	0	1
Tgm1	2.742877	1.948851	6.297124	0.0120934	1	0	1	0	0	1	4	2
Gm11210	2.722079	-0.49985	3.942198	0.047089219	1	1	1	1	0	3	14	1
Arsi	2.715037	1.410592	7.773995	0.005300369	1	0	4	0	0	5	14	3
A730089K	2.639315	-0.13821	4.904781	0.026782451	1	1	2	0	0	0	6	9
Rin1	2.621015	4.147035	9.02942	0.002656688	1	1	1	0	0	3	5	3
Gm40454	2.617576	1.174299	5.805586	0.015975346	1	0	1	1	0	5	4	2
Gm34979	2.602907	1.896823	9.835642	0.001711626	1	0	0	1	2	5	11	0
Mxd3	2.537765	1.264468	4.634301	0.031338903	1	0	1	0	0	1	3	2
Ltc4s	2.52522	-0.40411	4.164499	0.041279616	1	1	0	2	1	10	0	8
4931440P	2.510875	0.252684	6.839555	0.008916107	1	0	0	2	2	2	16	2
Gm17059	2.408893	1.385467	10.85596	0.000984781	1	1	0	2	2	6	13	3
LOC10816!	2.369379	0.367477	4.316835	0.037737043	1	0	3	0	5	10	16	7
Pyroxd2	2.339075	0.637894	6.08841	0.013607148	1	1	1	0	0	1	5	3
Gm38523	2.321312	0.136374	3.987625	0.045835633	1	2	1	0	0	6	6	1
Efna4	2.240543	4.000747	11.37587	0.000744043	0.923024417	2	2	0	1	10	6	3
Gm15866	2.232123	1.560613	5.871583	0.015387206	1	1	2	0	1	0	7	8
Gm42244	2.227465	0.612911	4.1048	0.042761646	1	0	5	0	0	1	14	4
Gm38767	2.214989	0.339908	4.844725	0.027730815	1	3	0	0	1	7	3	5
Gm30110	2.211593	2.123578	9.956197	0.001603088	1	2	4	1	3	10	16	10
Sec14l4	2.20207	2.73907	5.237491	0.022105133	1	1	0	1	0	2	1	5
LOC10263!	2.175944	2.825797	5.05593	0.024541803	1	0	1	1	0	0	5	3
LOC10105!	2.158857	0.723968	3.974717	0.046188215	1	1	0	2	1	7	6	2
Elf5	2.146946	2.611917	6.796827	0.009132	1	1	3	2	0	8	8	5
Ccnb2	2.118334	3.454356	5.684242	0.01711793	1	0	0	0	3	5	4	2
Rmi2	2.107731	1.177401	5.080074	0.024202337	1	0	2	0	1	2	7	2
Gm15506	2.09703	0.510856	4.033235	0.044612314	1	1	2	1	2	2	9	9
Prrt4	2.075981	0.320762	5.661348	0.017342787	1	2	2	1	0	3	9	5
Gm41719	2.070378	1.82756	4.449619	0.034909042	1	0	2	2	0	6	7	1
Pttg1	2.066935	6.221246	8.447995	0.003654461	1	8	194	162	2	384	413	315
Ky	2.012463	-0.03292	4.576543	0.032412543	1	4	3	0	0	13	5	4
Gm30064	2.011718	2.510133	5.168587	0.022998919	1	2	0	0	1	2	3	5
Spint2	2.009359	6.070949	5.821569	0.015830819	1	1	1	0	1	3	2	5
Gm34079	2.003065	1.088274	5.718204	0.016789895	1	0	3	1	4	2	8	14
Dhx58	2.001642	1.634755	4.479004	0.034313717	1	0	4	1	2	0	19	4
Grhl3	1.999211	3.545284	11.02329	0.000899741	1	5	1	1	1	9	9	7
Bmp15	1.99171	1.033807	4.663287	0.030814145	1	2	0	0	1	3	4	3
Gm40372	1.976614	-0.43883	3.951907	0.046818275	1	2	1	0	0	5	4	1
1700124L1	1.974756	0.070478	3.88256	0.048790121	1	1	2	0	0	6	4	0
Ikbke	1.926188	1.840496	4.141463	0.041844986	1	0	1	2	0	0	10	0
Il1rl2	1.918055	3.421452	12.34386	0.00044244	0.705016671	1	7	3	0	8	15	9
Gm30082	1.870344	1.076001	6.546173	0.010511003	1	6	6	3	1	18	18	9
Gm38424	1.856111	2.020384	8.217243	0.004149419	1	8	0	2	0	2	20	7
Gm30164	1.838972	1.605166	4.710998	0.02997024	1	0	2	0	2	3	9	0
AW01173!	1.827642	2.616463	7.669999	0.005614648	1	3	5	0	0	3	11	8
AA467197	1.825606	5.537047	3.937716	0.047214849	1	1	0	2	0	3	5	1
Esrp1	1.815306	7.137428	7.192407	0.007321271	1	1	2	1	1	6	3	5
Gm33350	1.812444	0.813447	4.002599	0.045430162	1	1	1	0	4	0	11	6
Tmod4	1.807164	0.808854	4.022009	0.044910175	1	2	1	0	2	7	4	3
9530034E	1.802572	2.00555	3.975423	0.046168868	1	0	1	1	1	0	9	0

Sigirr	1.777158	1.370781	3.978723	0.046078473	1	0	1	1	2	8	1	2
Gm41832	1.77142	4.247318	5.359296	0.020612095	1	0	3	0	1	10	0	1
Arl1	1.76225	1.276916	6.443152	0.011138086	1	6	5	4	2	15	7	20
Gm33583	1.745773	2.12932	5.209879	0.022458901	1	0	6	1	0	14	2	2
Gm33643	1.741619	1.865953	5.369108	0.020496462	1	0	2	2	1	5	1	7
4933408B1	1.739718	1.583471	6.243165	0.012467351	1	4	1	1	3	9	12	3
Tstd1	1.732968	2.250022	8.176822	0.004242903	1	8	13	1	4	26	19	19
Haus5	1.731701	0.497041	4.498843	0.033917804	1	0	4	2	1	4	8	6
Klhl40	1.727351	0.772517	4.196601	0.040505096	1	1	2	8	1	8	10	12
Gm8273	1.720667	1.21729	4.399855	0.035941994	1	0	6	1	9	17	8	14
Gm33696	1.701069	1.038029	4.017416	0.04503265	1	3	4	2	0	10	10	3
Svep1	1.662855	2.961474	7.07199	0.007829826	1	0	5	2	1	3	14	3
C1rl	1.632693	2.350444	5.977492	0.014489593	1	3	1	3	0	5	7	5
Krt13	1.615403	9.169037	6.435821	0.011184143	1	3	2	2	0	3	12	2
Npy	1.582393	6.939852	4.937277	0.026283419	1	0	0	1	4	2	9	1
F420014N1	1.57632	1.716743	5.947513	0.014738062	1	4	4	1	2	11	7	7
9930014A1	1.550481	2.603883	7.455595	0.006323957	1	5	3	3	1	10	10	7
Kcnj14	1.517935	1.270612	4.451155	0.034877653	1	14	2	0	11	9	31	20
Tead3	1.504125	4.325886	12.82266	0.000342447	0.648414993	9	6	7	1	7	29	14
LOC10264	1.491281	0.652327	5.069291	0.024353342	1	3	3	3	5	11	10	9
Zfp82	1.484021	1.319303	4.946263	0.026147127	1	7	9	4	1	15	16	13
Gm30883	1.480891	0.901629	5.072541	0.024307725	1	8	3	4	2	8	20	9
Slc25a35	1.468261	2.003983	5.141856	0.02335567	1	2	3	4	2	5	15	4
Scn4a	1.461722	2.119918	6.30987	0.012006767	1	2	7	10	1	18	16	8
Dbx2	1.438141	3.638121	6.947638	0.008392996	1	0	7	3	3	13	10	4
LOC10816	1.436671	3.278535	10.32409	0.001313047	1	8	8	2	3	13	17	13
Gatsl3	1.433727	3.120927	9.139917	0.002500924	1	8	3	3	8	21	19	6
LOC10816	1.426769	2.748415	6.832986	0.008948952	1	5	2	3	8	4	25	9
Phxr4	1.412021	3.095021	5.327405	0.020992618	1	1	3	3	5	5	15	5
Zfp119a	1.382314	1.75185	8.279401	0.004009736	1	4	6	5	2	12	16	6
Fdxacb1	1.378981	2.248327	6.747585	0.009387468	1	5	12	1	1	12	14	11
LOC10816	1.371505	3.09737	5.188954	0.022730893	1	6	0	3	9	14	19	4
Tlr5	1.363579	3.515049	4.413876	0.035647754	1	3	0	6	0	16	2	0
C430049B0	1.363127	3.397502	7.594892	0.00585339	1	7	7	3	4	16	8	16
4833438C0	1.351295	2.095172	5.179723	0.022851971	1	2	7	7	4	10	19	10
Pdzk1ip1	1.348526	7.968541	3.969903	0.046320438	1	1	1	1	3	5	6	1
Ak4	1.347622	2.425285	6.986836	0.008211138	1	1	4	6	6	15	11	7
LOC10816	1.343176	2.394477	9.356347	0.002222146	1	10	7	8	6	24	30	7
Gli3	1.29975	2.311581	4.713366	0.029928988	1	3	2	1	3	9	3	5
LOC10816	1.296805	1.038878	3.863239	0.04935497	1	4	5	9	5	5	7	28
LOC10263	1.276632	2.821842	8.343221	0.00387129	1	19	12	3	6	24	17	30
Lhpp	1.253456	1.992924	5.661922	0.017337111	1	3	12	10	2	10	21	17
Tmem202	1.241951	1.221573	5.705746	0.016909469	1	7	6	7	8	20	22	9
Lat2	1.237612	1.633797	4.780049	0.028791309	1	9	5	7	5	9	24	14
Gm32813	1.209997	3.710347	5.808636	0.015947657	1	11	4	12	2	6	32	14
Tmem251	1.202352	2.74667	7.007226	0.008118138	1	9	12	15	13	40	21	23
Gm30294	1.194571	1.9903	3.979234	0.046064495	1	6	5	10	10	25	12	16
Lonrf3	1.19293	2.740211	5.812034	0.015916876	1	2	12	14	7	20	23	17
Prr36	1.188447	1.556214	4.620796	0.03158658	1	11	5	13	13	20	36	18
Zc3h3	1.166072	2.24016	6.446161	0.011119233	1	9	6	3	6	7	21	13
Nccrp1	1.163043	9.048311	5.683053	0.017129536	1	4	1	2	7	5	13	6
LOC10524	1.156406	2.601686	6.316498	0.011961962	1	7	14	6	1	14	14	18
Gm32394	1.133378	2.338869	4.501602	0.033863124	1	1	6	5	7	13	14	5
Aifm2	1.131456	2.109216	3.860149	0.049445946	1	2	6	13	7	12	23	12
Gm39584	1.129834	4.381067	4.689242	0.030352036	1	6	4	3	0	4	16	2
Gm30816	1.127841	2.925828	3.987327	0.04584375	1	15	4	7	6	25	15	13
1700047A1	1.114348	2.615792	4.436121	0.0351861	1	9	3	4	7	10	10	17
Chek1	1.110458	1.845924	3.923022	0.047629199	1	5	10	3	1	9	14	8
Ciart	1.104822	1.920241	4.539512	0.033121029	1	8	17	10	9	16	28	26
Lyrm7	1.095438	2.818406	5.312537	0.021172504	1	18	40	24	14	47	65	41
Cnksr3	1.074532	4.158508	5.885062	0.015269861	1	6	7	3	4	13	13	6
Fam195a	1.056195	1.885082	5.975753	0.014503884	1	9	6	7	10	13	26	12
Gm38597	1.042343	2.899982	6.539213	0.010552204	1	13	9	10	20	19	41	22
9530091C0	1.022779	4.619902	7.763032	0.005332636	1	7	19	18	18	22	39	33
Slc22a8	1.021403	2.237146	4.429386	0.035325206	1	15	28	24	20	45	78	15
LOC10816	1.021243	1.640448	4.071954	0.043600815	1	18	16	7	17	17	48	25

Pxylp1	1.015822	4.107915	7.139851	0.007538943	1	16	13	10	16	25	35	24
Als2cr12	1.000043	3.710905	5.698817	0.016976352	1	3	14	7	5	19	10	14
Prkd2	0.996987	4.235604	4.945189	0.026163383	1	7	8	1	6	11	11	11
LOC10263	0.982329	2.07188	4.059853	0.043914325	1	5	6	12	8	8	20	18
Tmem194	0.974792	2.881314	5.784258	0.016170325	1	5	8	8	13	15	17	18
Sertad3	0.974453	4.573178	9.484671	0.002071958	1	36	48	57	28	94	73	78
Mapk7	0.953694	3.268333	8.444587	0.003661316	1	31	23	27	22	80	56	17
LOC10816	0.942161	1.465298	3.944448	0.047026292	1	14	12	16	12	24	25	28
Scrn2	0.933154	3.14477	3.841779	0.049990442	1	4	7	10	9	13	9	20
Gm5785	0.932421	3.707074	6.308311	0.012017329	1	9	9	7	13	18	33	5
Phykpl	0.914702	2.754176	4.595229	0.032061059	1	12	9	8	14	21	28	13
Zscan20	0.914361	2.561537	3.995026	0.045634753	1	3	14	18	14	23	25	21
Mpv17l	0.912426	2.953134	6.301274	0.012065126	1	28	42	35	35	65	85	49
Gm29975	0.910509	3.4599	4.803825	0.028396628	1	13	23	12	42	30	59	39
Tuft1	0.898198	6.354149	4.117786	0.042434589	1	4	7	5	3	7	18	2
LOC10816	0.884819	3.67557	4.540353	0.033104757	1	10	26	8	23	29	40	24
Psmb9	0.879203	4.161977	6.501249	0.010779875	1	18	12	9	9	24	21	21
Prss48	0.876545	3.586099	4.259539	0.039030523	1	9	7	16	6	17	23	13
Nova2	0.87369	2.540211	5.741963	0.016564272	1	28	34	29	21	52	61	41
Fadd	0.865733	4.125502	7.845726	0.005094099	1	17	10	18	9	24	38	13
Zkscan17	0.860446	3.000774	5.973242	0.014524553	1	27	14	11	16	28	43	23
Neil1	0.860001	2.750229	5.401778	0.020116249	1	16	15	11	5	19	26	19
Efna3	0.856655	3.854415	4.385349	0.036249061	1	7	8	9	3	13	16	8
Gria1	0.856213	3.28212	5.260411	0.021815871	1	36	56	39	27	73	72	66
Gm31262	0.850104	2.682236	4.011495	0.045191057	1	5	6	10	8	10	22	8
Gm16499	0.845623	1.873713	5.4515	0.019551533	1	13	13	13	12	25	26	18
Tnip2	0.839991	3.687827	5.142308	0.023349584	1	16	15	7	9	16	26	21
Tsen2	0.830897	2.840015	6.611908	0.010129907	1	22	32	26	29	70	37	37
1700017B	0.830562	3.249057	4.632432	0.031373071	1	21	17	13	10	32	26	23
Rnf113a2	0.829297	2.764778	4.722801	0.029765228	1	15	14	11	12	21	38	12
Tbrg3	0.818449	4.771236	5.108862	0.023803927	1	14	16	14	12	20	37	18
Pmf1	0.818352	4.673755	9.636726	0.001907249	1	22	43	43	15	71	42	47
Acy1	0.818143	2.60037	4.003143	0.045415504	1	24	18	21	11	22	33	41
Hap1	0.817296	2.347307	4.452894	0.034842159	1	21	30	8	19	50	33	20
Hpgd	0.815941	1.749028	4.386934	0.036215383	1	14	14	12	17	25	26	24
Abcd1	0.815319	3.740933	7.228286	0.00717637	1	35	58	41	45	77	105	56
Slc25a1	0.806006	6.688861	33.58675	6.81554E-09	0.000122598	393	408	368	366	712	718	574
Naprt	0.799075	4.861742	6.066782	0.013774773	1	18	14	10	10	17	20	30
Cdon	0.798281	5.052346	8.503794	0.003544067	1	15	21	16	17	27	26	36
4931414P	0.792917	2.955165	4.111506	0.042592415	1	12	7	17	11	17	29	16
Ngef	0.787357	4.228131	4.426302	0.035389108	1	7	7	9	7	13	15	11
Fastkd1	0.78724	2.246896	4.358867	0.036816784	1	21	16	23	18	35	47	21
Pkp3	0.779974	8.320924	5.67573	0.017201178	1	5	15	12	10	20	18	16
Nradd	0.778937	3.406528	4.49468	0.03400047	1	20	11	14	10	23	27	21
Zfp408	0.775514	2.935888	4.293564	0.0382569	1	20	15	12	11	28	23	23
Arf2	0.770675	5.632975	23.70065	1.12545E-06	0.010122256	84	87	87	82	131	164	138
Becn1	0.768252	3.380975	5.038661	0.024787639	1	27	40	35	27	69	44	49
Nxt1	0.766739	3.228375	3.958094	0.0466465	1	18	21	8	20	27	31	27
Fmnl3	0.762466	2.875507	4.894611	0.026940638	1	9	13	13	19	18	30	21
LOC10816	0.761282	3.821897	4.594966	0.032065987	1	35	61	39	56	71	90	79
Zfp36	0.760423	5.195642	4.177013	0.040975868	1	14	27	5	8	21	27	20
Ublcp1	0.759569	4.575996	7.350611	0.006703998	1	65	152	129	79	167	202	163
Pkn1	0.759305	4.298815	6.830543	0.008961201	1	52	47	33	34	59	95	58
P2rx7	0.749813	3.313621	4.204276	0.040322175	1	35	61	37	45	75	71	74
Dvl1	0.733045	4.151905	7.733598	0.005420264	1	37	63	42	61	83	91	77
Casp2	0.729204	4.107749	4.770462	0.028952054	1	22	17	7	11	20	30	21
Tgif1	0.718004	3.848212	4.23113	0.039689001	1	11	12	10	9	12	24	16
Insig2	0.714522	5.033879	8.213422	0.004158166	1	75	86	91	53	134	147	95
Nfkb2	0.708109	3.567023	4.826674	0.028022639	1	15	19	19	6	32	22	18
Map3k3	0.704376	5.062255	13.14296	0.000288602	0.586926344	54	71	60	32	90	94	79
BC024978	0.701223	3.423199	4.141074	0.041854601	1	10	20	16	13	20	24	27
Ccdc163	0.678906	2.600531	3.921929	0.047660173	1	11	22	19	17	30	26	26
Gramd3	0.673115	5.493425	5.430651	0.019786303	1	24	10	12	9	18	29	19
Bin3	0.670509	3.29124	4.592684	0.032108702	1	23	33	25	17	44	33	38
Tmem106	0.667716	3.024631	4.520824	0.033484691	1	13	24	10	16	37	15	22
Dgat2	0.650047	4.531924	5.374168	0.020437085	1	31	31	24	33	52	44	43

E130311K:	0.646052	3.333579	4.69284	0.030288551	1	29	35	30	20	48	47	38
Fam110a	0.626241	4.847184	5.210684	0.022448507	1	15	14	21	17	23	36	19
Phf13	0.622382	4.207486	6.260983	0.012342573	1	32	39	48	38	52	85	46
Kremen1	0.622231	4.999457	4.761424	0.029104454	1	29	20	24	29	38	45	35
Zfp65	0.619541	3.40181	4.188343	0.040702856	1	19	29	22	23	28	59	22
Zcchc14	0.607889	3.610004	4.788597	0.02864876	1	40	37	30	31	47	54	55
Plekhg2	0.607317	3.516054	4.065037	0.043779733	1	37	36	30	22	47	52	43
2410016O	0.602914	4.718502	6.154163	0.013110314	1	56	64	45	46	77	74	85
Phc1	0.596267	4.680207	8.581517	0.003395923	1	45	36	33	39	43	79	52
Mtfp1	0.579275	3.18887	4.208646	0.040218418	1	45	52	48	51	59	100	62
Ina	0.567109	3.679802	6.511682	0.010716809	1	57	71	49	76	99	117	67
Dctpp1	0.565198	4.24662	5.172045	0.022953182	1	37	50	56	36	72	95	35
Wdr4	0.563884	4.195446	4.964688	0.025869981	1	55	47	45	70	78	76	84
Zfp511	0.563078	3.70275	4.36004	0.036791441	1	44	51	24	34	61	69	40
Ltbp4	0.561272	4.62918	4.375196	0.036465646	1	28	33	29	28	53	42	35
Spry4	0.557554	2.638308	4.148016	0.041683326	1	35	41	35	30	48	66	42
Sdc1	0.556159	8.7174	4.092579	0.043071871	1	23	24	20	11	24	47	16
Stxbp3	0.551632	4.616732	4.82548	0.02804205	1	55	36	21	39	60	47	57
Ccdc9	0.543914	4.337037	5.452515	0.019540176	1	50	65	29	49	63	84	63
Tgfbr1	0.537148	5.441946	6.02934	0.01406999	1	38	45	31	30	48	60	48
Slc52a2	0.536708	4.816414	5.729701	0.016680319	1	139	156	161	128	229	232	173
Polr2i	0.536561	4.367938	4.553695	0.032847778	1	41	53	67	24	73	65	61
Vps52	0.536546	4.602248	5.922746	0.014946642	1	79	84	69	73	101	152	82
Mospd3	0.518432	5.31249	4.439397	0.035118648	1	50	54	99	57	120	88	71
Srf	0.516574	5.046681	4.629361	0.031429271	1	68	82	61	30	99	76	79
Dot1l	0.510361	5.426139	6.401035	0.011405387	1	72	139	76	102	133	148	129
Lsm7	0.502277	4.503689	4.20388	0.040331598	1	83	94	98	84	144	127	108
Dtx2	0.50139	4.553039	3.954409	0.046748725	1	31	44	36	35	60	51	43
Pex16	0.500716	4.473498	5.628156	0.017674218	1	80	92	64	57	101	128	82
Sh3gl1	0.496849	4.558828	4.473132	0.034431819	1	54	57	59	33	76	84	55
Cdyl2	0.489115	3.781238	4.650594	0.031042804	1	63	86	74	61	99	137	66
Map2k3	0.482087	6.409921	7.199936	0.007290618	1	61	53	56	84	91	97	78
Depdc7	0.477126	3.712773	4.110282	0.042623262	1	64	69	51	67	86	81	91
Taf1d	0.475134	5.476843	6.520522	0.010663671	1	100	105	106	92	116	198	110
Crlf2	0.47274	4.684777	4.182831	0.040835428	1	65	55	41	70	69	111	63
Cacnb1	0.471412	4.795604	5.37694	0.020404639	1	139	211	192	163	259	286	188
Pelo	0.466807	4.804686	7.056855	0.00789625	1	74	142	96	117	143	171	128
Gosr2	0.464839	4.7156	5.546859	0.018513885	1	75	72	80	74	111	106	93
Itga3	0.45997	6.607641	5.237623	0.022103449	1	121	138	137	131	207	193	143
Ttll12	0.454506	4.642737	3.937929	0.047208876	1	54	54	51	74	69	116	58
Grn	0.454352	7.472756	4.160103	0.041386886	1	278	303	270	243	454	342	316
Sft2d2	0.450508	6.173066	7.38731	0.006568564	1	91	91	72	72	119	114	99
Pcyox1l	0.448359	5.577312	4.24273	0.039418726	1	255	344	342	309	510	462	309
Sac3d1	0.443592	4.812187	5.036636	0.02481664	1	59	99	96	89	129	128	92
Rnpep	0.441728	6.81211	5.482792	0.019204559	1	52	85	83	63	82	142	67
Il4ra	0.438843	4.271513	4.557658	0.032771859	1	94	114	96	70	107	164	109
Nav3	0.423396	5.269858	4.02501	0.044830358	1	108	168	164	178	225	244	155
Tssc1	0.419328	5.05672	4.966747	0.025839202	1	104	105	118	91	157	162	102
Gap43	0.418973	7.425221	4.741665	0.029440571	1	934	1235	1115	954	1404	1611	1213
Fzr1	0.40637	6.061041	8.805832	0.003002692	1	188	197	181	131	216	269	205
Tada2a	0.404181	5.007299	4.718096	0.029846782	1	114	114	87	94	135	182	93
Pdxk	0.403069	6.876313	7.451907	0.006336928	1	143	150	143	130	201	216	145
Fam214b	0.401665	6.222898	5.368471	0.020503941	1	116	147	109	117	133	208	143
Plrg1	0.388867	5.687754	6.72396	0.009512618	1	136	192	145	135	167	245	182
Cep170b	0.383124	7.104357	8.058923	0.004528009	1	256	270	303	220	328	438	266
Akap5	0.381628	6.473028	4.467914	0.034537126	1	379	456	442	331	540	451	543
Rnf40	0.381453	5.095615	4.859582	0.027492992	1	77	110	82	96	106	141	108
Sumo3	0.371774	8.137887	6.011866	0.014209992	1	640	830	904	647	1003	1058	851
Irf3	0.371458	5.180966	3.87415	0.04903513	1	62	64	52	73	73	102	69
Pacsin3	0.370318	5.612155	4.116969	0.042455088	1	92	131	115	114	158	167	113
Ctdnep1	0.369119	5.522843	3.885972	0.048691066	1	118	99	133	89	135	164	126
Kdm6a	0.365418	4.810481	4.964838	0.02586774	1	76	95	53	55	86	94	87
Vgf	0.362174	5.332646	4.039975	0.044434489	1	254	272	255	208	323	421	220
Tor1aip2	0.361675	6.711552	6.693155	0.009678375	1	139	165	181	157	214	239	166
2510039O	0.360361	6.302971	4.077573	0.043456038	1	120	159	158	158	210	249	121
Myadm	0.360315	5.594049	5.336025	0.020889051	1	282	295	286	246	331	456	286

Snrpd3	0.358325	7.420267	5.392177	0.020227217	1	491	615	496	361	589	662	607
Ccdc50	0.356301	6.265366	5.05092	0.024612863	1	177	254	182	196	234	291	244
Snrnp70	0.355139	7.773808	4.590501	0.032149611	1	524	713	398	608	657	759	703
Bc1	0.354692	8.198465	3.858701	0.049488643	1	1003	1235	1114	1032	1763	1345	1072
Ehd1	0.353881	6.72476	5.972607	0.014529788	1	138	153	153	158	188	226	163
Ccnl2	0.353138	5.92357	4.827063	0.028016313	1	152	156	121	126	199	183	147
Dlgap3	0.352543	4.256886	4.509442	0.033708227	1	123	124	136	117	147	182	148
Rnf112	0.351947	5.38086	5.257999	0.021846128	1	216	289	244	310	361	350	296
Emd	0.351778	5.818106	5.31208	0.021178053	1	135	152	137	157	188	231	140
Yipf4	0.350781	5.351106	5.128846	0.023531365	1	154	165	157	147	209	244	146
Vps36	0.350155	5.583673	4.160437	0.04137873	1	111	122	94	98	111	162	131
Ddhd1	0.34311	5.22648	5.984518	0.014431987	1	198	215	166	143	230	260	194
Dnpep	0.341963	6.173906	7.704011	0.005509824	1	181	186	212	207	256	293	200
Ddr1	0.341377	7.937609	6.465762	0.010997246	1	193	268	212	173	267	320	215
Rarg	0.332151	8.127643	5.000894	0.025334232	1	196	168	179	133	190	242	203
Foxo3	0.32971	6.333536	5.586507	0.018099355	1	183	231	215	165	250	266	226
Ubqln4	0.32747	5.405567	4.10224	0.042826433	1	137	179	148	135	191	200	168
Samd12	0.327291	5.901981	4.586844	0.032218286	1	243	283	225	213	293	372	243
Ngdn	0.324741	5.529973	4.4979	0.033936506	1	129	154	143	114	194	176	135
Tmcc1	0.322166	5.615784	6.438525	0.01116713	1	179	168	159	171	194	275	169
Ppp3r1	0.315657	7.733055	5.356853	0.020640989	1	857	976	951	822	1118	1294	948
Atp6v0b	0.314994	8.012093	3.982297	0.045980817	1	870	969	1172	1048	1287	1566	967
Gng12	0.313765	6.722468	5.326487	0.021003682	1	140	160	164	163	194	225	165
Ets2	0.308613	6.550416	3.842294	0.049975097	1	117	132	141	119	114	199	158
Lcor	0.307707	6.386707	4.065448	0.043769079	1	176	156	138	165	194	213	180
Shisa5	0.303091	6.67784	5.011051	0.025186016	1	214	276	221	226	305	364	203
U2af2	0.302163	6.713925	4.427428	0.035365764	1	287	297	240	269	368	378	265
Maoa	0.299188	5.553135	4.438683	0.035133349	1	244	298	234	224	309	295	304
Ncln	0.294138	5.644259	4.012302	0.045169438	1	154	155	181	163	199	250	155
Lsm6	0.274715	6.324347	4.391644	0.036115474	1	246	249	256	211	279	332	259
Atp6v1f	0.274036	8.331132	5.481597	0.019217689	1	1266	1434	1466	1117	1639	1901	1261
Tmem14c	0.268557	6.320716	3.868038	0.049214011	1	224	306	267	219	342	336	237
Fyn	0.268435	5.919639	5.003538	0.025295562	1	390	434	385	345	393	569	436
Aamp	0.261929	7.821046	4.251455	0.039216722	1	525	732	629	576	819	841	554
Naa35	0.259165	6.648721	4.033861	0.044595773	1	244	271	244	215	301	302	264
Nedd8	0.251465	8.721455	4.447942	0.034943326	1	1417	1723	1655	1310	1894	2070	1476
Akt1	0.249785	7.364651	4.99788	0.025378386	1	656	670	713	559	839	895	590
2310022B(	0.246041	6.762892	4.563041	0.032669021	1	245	275	249	252	300	329	274
Smim15	0.234051	6.452912	3.8505	0.049731171	1	218	285	204	206	269	297	234
Ptges3	0.23306	7.469345	3.901306	0.048248589	1	636	880	770	642	851	946	762
Ldlr	0.230994	8.384465	4.310689	0.037873617	1	524	558	509	510	652	669	522
S100a6	0.210539	12.80554	4.104921	0.04275859	1	4907	5268	5761	4558	6390	6464	4895
**Downregulated genes**												
3110021A:	-5.53401	1.857178	18.28809	1.89891E-05	0.08539381	5	6	3	7	0	0	0
AW04773(	-5.42349	-0.34356	6.411225	0.011340114	1	3	2	0	14	0	0	0
Tfap2c	-5.39454	5.155689	20.22957	6.86831E-06	0.035299197	6	4	0	9	0	0	0
Trim63	-5.38946	-0.33005	10.20924	0.001397387	1	7	6	2	4	0	0	0
Gm35855	-5.31308	0.321223	11.79729	0.000593169	0.790364922	6	6	1	5	0	0	0
Gm40715	-5.16666	-0.43991	5.209513	0.022463632	1	3	2	7	4	0	0	0
Neurog1	-5.0962	-0.48191	4.778054	0.028824671	1	4	1	0	10	0	0	0
A730056A	-4.93406	-0.22403	5.388899	0.02026525	1	2	7	3	2	0	0	0
Skint3	-4.92526	-0.48176	8.070671	0.004498745	1	4	9	0	1	0	0	0
Gm30296	-4.89893	-0.08838	4.261469	0.0389862	1	0	0	4	9	0	0	0
Dyrk4	-4.88989	-0.42216	6.781461	0.009210947	1	2	0	6	5	0	0	0
A430078I0	-4.8731	0.659213	8.654627	0.003262319	1	8	2	2	1	0	0	0
1700028E:	-4.85153	0.021972	6.067017	0.013772938	1	1	5	1	6	0	0	0
Fam205c	-4.8508	-0.7031	5.158426	0.023133856	1	5	5	0	3	0	0	0
Fbxo48	-4.84072	-0.22982	5.000022	0.025346999	1	4	6	0	3	0	0	0
Gm31898	-4.77467	-0.72256	5.710319	0.016865476	1	1	1	3	7	0	0	0
C430042N	-4.77123	0.13429	9.094189	0.002564229	1	4	1	0	7	0	0	0
Adgrg5	-4.76079	-0.7027	8.560006	0.003436283	1	1	2	3	6	0	0	0
Snord68	-4.75794	-0.35784	5.420318	0.019903736	1	4	2	5	1	0	0	0
K230010J2	-4.75109	-0.10453	7.598122	0.005842912	1	4	3	3	2	0	0	0
Tnfrsf11b	-4.75039	1.738532	12.30921	0.000450728	0.705016671	1	3	3	5	0	0	0
Slc15a3	-4.6488	-0.33737	4.806642	0.028350241	1	7	1	3	0	0	0	0
LOC10263!	-4.64534	-0.75648	6.728086	0.009490638	1	5	1	4	1	0	0	0

Rnf222	-4.64163	1.042954	8.364263	0.003826717	1	0	0	11	0	0	0	0
5730522E(	-4.64139	-0.27212	6.157447	0.013085997	1	9	2	0	0	0	0	0
Ccdc170	-4.63038	-0.23221	6.748995	0.009380053	1	2	3	2	4	0	0	0
Clhc1	-4.58307	-0.26954	4.498691	0.033920805	1	3	7	1	0	0	0	0
Gm30335	-4.53551	-0.46768	4.417905	0.035563681	1	0	0	2	8	0	0	0
Gm39526	-4.52556	-0.23312	5.415569	0.019957953	1	0	1	0	9	0	0	0
Gabra6	-4.52489	-0.68622	5.274454	0.021640575	1	5	0	4	1	0	0	0
Prr32	-4.52152	-0.54139	5.883548	0.015282995	1	1	0	6	3	0	0	0
Gm12052	-4.51356	-0.653	3.979852	0.046047622	1	2	2	0	6	0	0	0
Gm10406	-4.49883	-0.65575	4.844719	0.027730918	1	0	3	1	6	0	0	0
Gm2449	-4.49106	-0.65159	4.530826	0.033289538	1	0	3	4	3	0	0	0
Sult6b1	-4.47304	-0.082	7.981503	0.004725768	1	3	6	0	1	0	0	0
Taf7l	-4.47216	-0.60515	3.971668	0.046271902	1	3	5	0	2	0	0	0
Pcdhgc3	-4.46798	-0.4479	5.105872	0.023844994	1	1	5	3	1	0	0	0
Gm38779	-4.376	-0.81123	4.912957	0.026655975	1	4	1	0	4	0	0	0
Fbxl22	-4.35263	0.330523	5.503767	0.018975558	1	3	2	4	0	0	0	0
Gm5868	-4.34079	-0.41226	6.163983	0.013037731	1	0	3	5	1	0	0	0
Cst7	-4.33802	-0.57216	4.353746	0.036927629	1	0	4	0	5	0	0	0
4930599N	-4.32979	-0.36512	5.18411	0.02279435	1	2	4	3	0	0	0	0
Gm12248	-4.22523	-0.35323	4.984722	0.025572091	1	4	0	0	4	0	0	0
Npm2	-4.22475	-0.21999	5.543715	0.018547172	1	1	0	0	7	0	0	0
Gm36443	-4.22285	-0.4817	5.25434	0.021892109	1	2	0	1	5	0	0	0
Gm35200	-4.2159	-0.42872	4.653545	0.030989493	1	3	0	4	1	0	0	0
Vtcn1	-4.21117	0.18758	6.247593	0.012436221	1	5	0	3	0	0	0	0
Abca4	-4.20743	-0.05847	4.223624	0.039864941	1	0	1	3	4	0	0	0
Ankle1	-4.20227	1.312284	7.952449	0.004802234	1	3	0	2	3	0	0	0
Gm34168	-4.20162	-0.70575	4.364127	0.036703292	1	3	1	4	0	0	0	0
Gm38426	-4.19698	2.809487	8.319288	0.003922629	1	4	0	4	0	0	0	0
Ltf	-4.19158	2.145187	7.579022	0.005905148	1	0	2	3	3	0	0	0
Tmem30b	-4.19149	4.111139	8.61124	0.003340948	1	1	2	2	3	0	0	0
Aldoart2	-4.19053	-0.44014	5.526667	0.018728748	1	2	2	3	1	0	0	0
Gm32345	-4.1875	-0.72119	4.564826	0.032634981	1	2	2	4	0	0	0	0
CJ186046F	-4.18297	-0.5573	4.299622	0.038120836	1	0	3	1	4	0	0	0
H2-Q2	-4.18183	-0.45177	4.490039	0.034092901	1	5	3	0	0	0	0	0
Tslp	-4.17841	-0.10916	6.889864	0.008668585	1	1	4	0	3	0	0	0
Gpr119	-4.16972	-0.79469	5.001452	0.025326057	1	0	4	1	3	0	0	0
Gm41753	-4.16789	-0.69096	5.154008	0.023192775	1	3	4	1	0	0	0	0
6230400D	-4.1136	-0.04977	4.654596	0.030970523	1	0	8	0	0	0	0	0
Gm12915	-4.04198	-0.54693	3.890857	0.048549635	1	4	0	1	2	0	0	0
Gm31909	-4.03016	-0.11965	4.745695	0.029371692	1	3	0	4	0	0	0	0
Orm1	-4.02749	-0.73737	4.43388	0.035232324	1	0	1	1	5	0	0	0
Sp5	-4.02706	-0.75685	4.417103	0.035580392	1	4	1	0	2	0	0	0
Gm40180	-4.02698	-0.61744	3.879276	0.048885633	1	2	1	1	3	0	0	0
Zfp92	-4.02278	0.630462	5.695459	0.017008862	1	1	0	6	0	0	0	0
Gm15972	-4.02161	0.417554	5.039201	0.024779913	1	5	1	1	0	0	0	0
Gm33370	-4.01949	-0.44034	6.108683	0.013451935	1	3	1	1	2	0	0	0
Tlr6	-4.01941	0.543299	4.96446	0.025873396	1	1	1	3	2	0	0	0
C5ar1	-4.01925	-0.48367	4.579196	0.0323624	1	2	1	3	1	0	0	0
Gm26847	-4.01924	-0.12418	3.905415	0.048130725	1	3	1	3	0	0	0	0
Slc12a8	-4.01921	-0.36843	3.93404	0.047318143	1	3	1	3	0	0	0	0
Oacyl	-4.01865	0.462377	5.734327	0.016636445	1	3	1	2	1	0	0	0
Gm26698	-4.01703	1.5033	6.983296	0.008227396	1	1	1	0	5	0	0	0
Ak7	-4.0168	0.376842	5.336814	0.020879605	1	0	1	4	2	0	0	0
Dll1	-4.01523	3.264433	7.502021	0.00616298	1	2	0	0	5	0	0	0
Fam71f2	-4.01314	-0.60688	4.291348	0.038306793	1	2	2	0	3	0	0	0
Gm32926	-4.01227	-0.62432	4.170776	0.04112697	1	3	2	0	2	0	0	0
Gm33228	-4.01213	-0.70501	5.720835	0.01676476	1	2	2	0	3	0	0	0
Gm39055	-4.01037	-0.40587	4.498247	0.033929626	1	5	2	0	0	0	0	0
Gm39171	-4.00824	0.182895	5.678368	0.017175335	1	1	2	2	2	0	0	0
Epha8	-3.99657	-0.29299	4.971856	0.025762992	1	1	3	1	2	0	0	0
Gm33098	-3.99336	2.128376	7.273683	0.006997218	1	0	7	0	0	0	0	0
Syt10	-3.98336	-0.5272	4.973542	0.025737894	1	0	4	1	2	0	0	0
4930525G.	-3.97592	0.168928	4.156032	0.041486494	1	0	4	2	1	0	0	0
1700109K.	-3.97464	0.217688	5.249722	0.021950276	1	0	5	0	2	0	0	0
Hbq1a	-3.83347	-0.86807	3.880603	0.048847015	1	1	0	1	4	0	0	0
Gm35235	-3.82726	-0.45801	4.189147	0.040683568	1	2	0	2	2	0	0	0

4933433G	-3.81686	-0.48239	4.220034	0.039949355	1	2	1	0	3	0	0	0
LOC10816	-3.81625	-0.88809	3.956729	0.046684331	1	0	1	1	4	0	0	0
Tmem190	-3.81617	0.480332	5.740403	0.016578988	1	0	0	2	4	0	0	0
Gm36316	-3.81516	-0.68701	3.988165	0.045820948	1	1	1	1	3	0	0	0
Lbx2	-3.81248	-0.39385	4.276447	0.038644044	1	3	1	1	1	0	0	0
Ces5a	-3.81223	-0.15941	4.676392	0.030579904	1	2	1	1	2	0	0	0
Car9	-3.80681	2.711371	6.480608	0.010905768	1	1	0	0	5	0	0	0
Usp43	-3.80416	2.358084	6.468208	0.010982124	1	3	1	0	2	0	0	0
Slc6a14	-3.80388	6.013522	6.513144	0.010708001	1	0	0	6	0	0	0	0
Bnc1	-3.80301	4.331699	6.569594	0.010373562	1	0	1	2	3	0	0	0
Tprg	-3.80246	4.745691	6.662877	0.009844187	1	5	1	0	0	0	0	0
Gm16351	-3.79995	0.20869	5.406435	0.020062645	1	0	2	1	3	0	0	0
Gm39446	-3.79806	-0.42375	4.425709	0.035401405	1	1	2	1	2	0	0	0
Gm35480	-3.79561	-0.63932	5.024127	0.02499653	1	2	2	2	0	0	0	0
AI182371	-3.79057	-0.15446	5.255588	0.021876403	1	0	3	0	3	0	0	0
Gm39819	-3.78055	-0.70216	4.243172	0.039408483	1	2	3	1	0	0	0	0
LOC10263	-3.76973	-0.43977	4.390239	0.036145236	1	1	4	0	1	0	0	0
Gm40011	-3.58126	-0.81059	4.047228	0.044243931	1	3	0	1	1	0	0	0
Gm35887	-3.58022	0.684465	4.587973	0.032197065	1	0	0	0	5	0	0	0
AU040972	-3.57459	0.228368	4.591196	0.032136585	1	1	0	2	2	0	0	0
Lrrc66	-3.57129	0.097865	3.985792	0.045885527	1	0	0	5	0	0	0	0
Siglecg	-3.56961	0.954649	5.075461	0.024266824	1	5	0	0	0	0	0	0
Axdnd1	-3.56747	0.407218	4.692003	0.030303295	1	0	0	5	0	0	0	0
Gm28499	-3.56717	-0.84874	4.086564	0.043225426	1	0	1	1	3	0	0	0
Cdca5	-3.56497	1.405954	4.978436	0.02566517	1	0	1	0	4	0	0	0
Il22ra2	-3.56479	-0.66962	3.915761	0.047835357	1	3	1	1	0	0	0	0
LOC10816	-3.56362	0.567928	4.829284	0.027980244	1	1	1	1	2	0	0	0
Sprr1a	-3.56197	2.89749	4.294006	0.038246957	1	2	1	2	0	0	0	0
Siah3	-3.5619	-0.88894	4.35208	0.036963781	1	2	1	2	0	0	0	0
Gm40218	-3.56097	0.419808	5.154291	0.023188995	1	1	1	2	1	0	0	0
Mfsd4b5	-3.56086	-0.29992	4.112122	0.042576917	1	0	1	3	1	0	0	0
Ltb4r1	-3.56076	3.36368	5.416223	0.019950469	1	4	1	0	0	0	0	0
Angptl6	-3.55993	-0.65306	4.737914	0.029504825	1	1	1	3	0	0	0	0
Oas3	-3.55903	2.306989	5.145891	0.023301451	1	0	1	4	0	0	0	0
Gm5088	-3.55755	-0.5181	3.889731	0.048582218	1	0	1	4	0	0	0	0
Krt7	-3.55213	4.219524	5.229945	0.02220123	1	1	3	1	0	0	0	0
Gm40688	-3.55129	-0.12613	4.0314	0.044660878	1	3	2	0	0	0	0	0
LOC10816	-3.55128	-0.16232	4.027563	0.044762557	1	3	2	0	0	0	0	0
Gm12657	-3.54926	-0.15662	3.863558	0.049345591	1	1	2	1	1	0	0	0
Nek2	-3.5477	1.615557	5.218749	0.022344614	1	0	4	1	0	0	0	0
Rab19	-3.54458	1.998027	5.074791	0.024276204	1	0	4	0	1	0	0	0
Dna2	-3.54002	0.362628	4.313756	0.037805412	1	1	3	0	1	0	0	0
Snord47	-3.53693	-0.3643	4.108857	0.042659194	1	2	3	0	0	0	0	0
Nhlrc4	-3.53315	-0.41691	3.880759	0.048842468	1	0	3	1	1	0	0	0
Cyp4f17	-3.46854	1.250544	7.699613	0.005523266	1	5	6	9	0	0	0	1
Itk	-3.40436	-0.29685	5.581009	0.018156257	1	3	3	4	9	0	0	1
Sugct	-3.32621	0.510417	8.184841	0.004224188	1	3	12	11	6	1	0	1
Gm40331	-3.29829	0.495291	11.17273	0.000830083	0.995436053	10	11	7	15	2	1	0
Slc5a9	-3.27868	1.024795	4.022091	0.044908	1	4	0	0	0	0	0	0
Aurkb	-3.27648	1.354228	4.069575	0.043662253	1	3	0	1	0	0	0	0
Serpine1	-3.27032	0.742833	3.930217	0.047425813	1	2	1	0	1	0	0	0
2810408I1	-3.26953	1.457286	3.997649	0.045563783	1	3	1	0	0	0	0	0
Ly6g6e	-3.26886	3.430459	4.223608	0.039865305	1	3	1	0	0	0	0	0
Tjp3	-3.26882	5.38959	4.435772	0.035193305	1	2	0	1	1	0	0	0
Esrp2	-3.26841	3.864091	4.355318	0.036893576	1	3	1	0	0	0	0	0
Fam186b	-3.26825	-0.57322	3.909393	0.048016942	1	2	1	1	0	0	0	0
C330021F2	-3.25063	0.5688	3.924088	0.047599012	1	0	3	0	1	0	0	0
Dlx5	-3.24057	1.659695	3.908692	0.048036957	1	0	4	0	0	0	0	0
Manba	-3.23481	0.352846	4.920287	0.026543114	1	1	4	3	8	0	1	0
Ggt1	-3.09647	0.168459	4.297171	0.038175825	1	5	4	6	0	1	0	0
Zbtb8b	-3.00282	3.562539	9.005191	0.002692139	1	4	5	4	1	1	0	0
Rps15a-ps	-2.9896	1.076862	7.729705	0.005431963	1	4	5	3	2	0	0	1
Gm17634	-2.89407	3.678103	8.285566	0.003996144	1	0	8	5	0	1	0	0
Acr	-2.82633	0.238746	6.057708	0.013845731	1	3	11	3	6	0	0	2
Amd2	-2.82307	1.39359	9.08137	0.002582266	1	7	5	9	10	3	0	0
Gm14719	-2.81004	1.045048	7.368179	0.006638815	1	13	7	6	13	2	2	0

Ankrd61	-2.77624	0.443468	4.040303	0.044425846	1	2	9	0	1	0	1	0
Exph5	-2.7705	6.814736	12.62796	0.000380019	0.683578819	3	7	8	3	0	0	2
Gm41848	-2.76724	-0.22248	5.101217	0.023909058	1	1	4	3	12	0	2	0
Gm41640	-2.69198	0.17489	3.844162	0.049919467	1	3	1	0	7	1	0	0
Lockd	-2.67683	1.93723	4.856956	0.027534868	1	1	5	1	4	0	1	0
1810044D(	-2.67663	0.645088	4.838061	0.027838172	1	1	2	3	5	1	0	0
6530403H(	-2.66865	-0.04598	4.445786	0.034987477	1	1	6	4	9	1	0	1
Cbln3	-2.6642	2.429944	6.353366	0.011715879	1	2	5	4	0	1	0	0
Ccl7	-2.65532	-0.24872	4.899334	0.026867055	1	5	2	3	1	0	0	1
Gm19522	-2.62512	0.795671	5.020398	0.025050413	1	8	6	1	4	0	1	1
Gm26666	-2.61021	-0.19399	5.52851	0.018709035	1	7	2	5	4	0	2	0
D330045A	-2.58485	0.024358	6.687978	0.009706521	1	6	10	3	8	0	1	2
BC020402	-2.5335	0.613327	4.24379	0.039394142	1	2	4	2	10	1	0	1
Gm5621	-2.52264	-0.3447	3.877304	0.048943087	1	1	5	0	4	1	0	0
Alox5	-2.49914	1.031994	4.544018	0.033033966	1	3	8	4	2	0	2	0
2610316D(	-2.46082	-0.07449	4.571924	0.032500048	1	10	3	1	3	1	0	1
Gm26519	-2.45608	-0.28855	5.087322	0.024101383	1	5	5	4	9	0	3	0
Casc5	-2.39935	3.866924	4.676107	0.030584988	1	3	3	0	3	0	1	0
Mirt1	-2.39895	2.66862	5.773073	0.016273552	1	1	2	6	7	0	1	1
Aldh1a7	-2.39695	2.775294	14.76327	0.000121886	0.398635001	19	14	20	13	3	2	4
Gm30635	-2.39398	2.281977	4.153612	0.041545821	1	9	0	0	0	0	0	1
Mylk3	-2.3929	1.224301	6.148315	0.01315374	1	8	1	3	4	1	0	1
Gm42307	-2.38928	1.646967	4.019953	0.044964977	1	2	1	0	6	0	0	1
Gm33474	-2.34369	0.353908	6.480025	0.010909348	1	7	4	9	8	1	3	0
E230013L2	-2.33354	-0.1395	5.690205	0.01705986	1	1	7	9	5	2	1	0
Lrrd1	-2.32855	0.213479	4.333697	0.037365011	1	9	6	1	6	3	0	0
Nupr1l	-2.28002	1.53297	5.632046	0.017635039	1	2	3	7	3	0	0	2
Comp	-2.24836	-0.06977	5.163162	0.023070864	1	5	9	5	8	3	1	0
Prss27	-2.23285	8.251604	4.281141	0.038537463	1	3	2	2	1	1	0	0
Gm7967	-2.21565	0.435726	6.084581	0.013636667	1	23	5	4	7	3	1	2
Ephx3	-2.20817	5.112202	6.76222	0.009310783	1	3	2	2	7	0	2	0
Gm16630	-2.13793	0.73784	4.515899	0.033581239	1	6	8	9	13	4	2	0
LOC10816	-2.13778	3.317434	4.028387	0.044740697	1	5	3	0	5	0	2	0
Dusp6	-2.132	1.583178	7.156863	0.00746777	1	5	5	11	10	2	1	2
Gm12174	-2.12597	1.050669	5.953985	0.014684058	1	2	4	1	12	1	1	1
Gm16116	-2.11016	0.655398	4.954771	0.026018764	1	5	7	4	3	3	0	0
Pnlip	-2.10884	0.622635	7.63576	0.005722226	1	10	7	17	7	2	4	1
Wdr89	-2.07734	1.29668	7.869552	0.005027409	1	5	8	8	15	3	0	3
Inhbb	-2.04936	1.521087	5.163457	0.023066939	1	4	5	9	0	2	1	0
Meox2	-2.0455	0.472104	4.250219	0.039245261	1	7	10	5	12	2	3	1
Egfl6	-2.03491	2.78056	6.620662	0.010080237	1	3	2	8	5	0	0	3
Gm38859	-2.02556	1.723888	5.089402	0.024072486	1	3	2	13	6	0	0	4
Gm32643	-2.00694	0.956961	4.179134	0.040924617	1	6	7	1	13	1	4	0
Pomc	-2.00662	1.976977	5.334094	0.020912213	1	96	47	18	5	11	13	7
2900092D	-1.98753	2.570726	4.765565	0.029034531	1	6	1	1	14	3	1	0
Gm38462	-1.98256	0.594844	5.696525	0.016998536	1	12	5	10	1	2	1	2
Alox12	-1.96882	4.279872	6.881315	0.008710145	1	9	1	5	2	1	2	0
Bco1	-1.88694	2.700299	5.153923	0.023193916	1	4	2	0	10	1	1	1
Gm42005	-1.88162	2.92866	7.60206	0.005830164	1	8	7	4	7	0	2	3
Il31ra	-1.87333	1.89955	14.48794	0.00014106	0.422897549	22	58	31	34	13	9	7
4732471J(	-1.87259	1.305897	5.111529	0.023767363	1	11	21	18	4	3	0	7
Tmem51os	-1.81517	3.256104	9.324106	0.002261586	1	14	4	10	15	6	2	1
Cdca7l	-1.8077	2.440866	4.49663	0.033961724	1	5	4	4	2	0	3	0
2210404O	-1.77849	1.922205	6.907243	0.008584728	1	5	14	4	14	2	6	0
Zfp493	-1.7313	0.976316	5.562614	0.018348	1	7	7	8	10	0	4	3
Gm31504	-1.71544	1.726195	5.536215	0.018626832	1	5	9	2	16	6	0	1
BC029722	-1.71174	0.72852	4.641528	0.031207212	1	17	10	11	6	5	4	1
Tacstd2	-1.69212	8.937077	4.716005	0.029883085	1	1	4	4	5	1	1	1
Irs3	-1.66949	0.328013	4.770403	0.028953055	1	10	12	6	3	0	4	3
E2f8	-1.66265	2.362584	5.050539	0.02461828	1	4	11	2	5	1	4	0
Bik	-1.65808	3.449757	4.578587	0.032373899	1	1	12	3	2	2	2	0
Gcnt1	-1.63956	0.811083	4.313521	0.037810618	1	11	5	13	13	7	2	1
Anxa1	-1.62766	7.461816	13.15167	0.000287263	0.586926344	9	16	11	19	6	2	5
Rassf6	-1.58614	2.860405	11.80876	0.000589526	0.790364922	4	10	27	16	5	5	4
Glt28d2	-1.58564	3.914447	4.889732	0.027016879	1	10	4	1	2	3	0	1
Sox21	-1.58504	4.707051	3.931282	0.047395814	1	4	2	3	4	0	0	3

Lck	-1.56978	0.581034	4.564789	0.032635702	1	7	12	7	10	5	4	0
4930550C1	-1.49452	3.019424	6.187635	0.012864601	1	15	12	1	3	0	5	3
Pkd1l3	-1.49171	1.21031	4.028857	0.044728244	1	5	10	10	3	2	1	4
Rab10os	-1.48839	2.103433	5.958445	0.01464695	1	5	14	10	16	4	7	1
Hcar1	-1.48287	2.060363	6.007244	0.014247259	1	4	17	7	6	1	8	0
Ifi44	-1.47867	2.580444	5.08964	0.024069192	1	16	21	17	19	11	9	0
Foxm1	-1.46127	2.883943	5.289845	0.021450124	1	6	7	10	4	3	0	4
Il17re	-1.45908	5.902536	13.11041	0.000293659	0.586926344	6	16	20	14	2	7	6
Mettl15	-1.44369	1.637869	4.332623	0.03738859	1	17	8	8	10	2	8	2
Nrg4	-1.44249	1.176759	4.933397	0.026342485	1	5	17	6	9	4	5	1
Serpinb1b	-1.44065	1.021892	6.474925	0.01094069	1	16	9	17	24	9	4	5
LOC10816	-1.40326	1.933142	4.749081	0.029313946	1	11	6	7	15	0	7	4
Myadml2	-1.37749	1.084744	4.946196	0.026148139	1	12	18	27	8	6	4	8
Zkscan7	-1.3769	1.974751	4.765977	0.029027573	1	9	19	7	15	4	5	5
Kcng2	-1.33341	1.986629	7.762503	0.0053342	1	41	28	27	22	15	12	8
Suv39h2	-1.33217	1.307579	4.692155	0.030300623	1	7	20	17	7	2	9	4
Myh8	-1.31467	2.129157	4.332715	0.03738656	1	63	28	37	82	16	23	24
Exo5	-1.3124	2.551651	6.670868	0.009800144	1	23	15	18	14	15	4	2
Gstt2	-1.29991	3.133711	9.256816	0.002346206	1	18	18	24	22	6	13	6
Slc52a3	-1.28499	2.948697	5.182571	0.022814537	1	8	11	2	12	6	4	0
Wfdc2	-1.27653	5.527928	9.239578	0.002368395	1	8	25	11	12	9	5	3
Epha2	-1.27194	3.92415	4.294786	0.038229401	1	6	2	5	10	1	5	1
Nt5dc1	-1.24937	3.667024	6.340626	0.011800323	1	19	9	8	9	7	4	3
Pbld2	-1.23526	1.744739	5.263191	0.02178105	1	12	12	16	17	6	7	5
Gpr83	-1.21036	1.177767	6.77596	0.00923938	1	16	12	11	11	4	7	5
Grm5	-1.20032	1.253489	4.041067	0.044405745	1	9	23	29	19	9	11	6
Emilin2	-1.19878	0.439639	3.933811	0.047324577	1	9	8	13	12	5	9	0
Stxbp2	-1.19202	5.353611	14.10979	0.000172444	0.477219405	29	21	30	22	12	7	14
Cysltr2	-1.15477	1.021614	4.33869	0.037255572	1	25	21	13	24	5	8	14
St6galnac2	-1.14919	4.566285	4.074157	0.043543977	1	19	14	4	14	6	6	5
Bhlhe22	-1.13011	1.232983	4.493676	0.034020446	1	16	24	12	24	8	12	6
Zfp58	-1.12131	2.065138	5.974753	0.014512118	1	21	15	18	27	9	12	7
Sh3bp1	-1.09954	5.003232	4.841959	0.027775322	1	10	11	4	10	2	5	5
Fgfr3	-1.0969	4.611079	6.577473	0.010327739	1	14	17	11	22	3	8	11
Zfp141	-1.08944	2.99654	10.107	0.001477074	1	28	36	29	21	18	15	7
Apoc1	-1.08913	4.494316	4.972464	0.025753936	1	15	11	9	16	4	10	4
Nup98	-1.08401	2.946886	8.048549	0.004554012	1	37	36	21	26	18	21	4
Tmem38b	-1.08105	2.034634	4.644565	0.031152039	1	21	20	20	30	4	15	13
Gm26782	-1.07166	1.659132	5.504025	0.01897276	1	13	34	35	17	11	15	9
Slco1c1	-1.0652	1.650924	4.086291	0.043232412	1	30	23	26	31	20	9	10
Gm16364	-1.05299	2.61806	9.503199	0.00205114	1	48	33	61	43	19	21	26
AW14615	-1.05255	3.321814	6.025104	0.014103803	1	19	19	14	7	6	8	7
Alas2	-1.04328	3.536979	4.37279	0.036517147	1	123	25	63	54	41	34	23
4930412C1	-1.03733	2.372258	4.534179	0.03322439	1	23	40	28	26	12	16	14
Hook1	-1.00839	4.352153	5.07515	0.024271169	1	14	38	13	21	15	6	10
Exosc9	-0.98535	2.415114	4.785293	0.028703765	1	26	32	28	17	10	19	10
Nlrx1	-0.98006	4.241951	13.21278	0.000278046	0.586926344	48	35	54	73	27	30	23
Rft1	-0.98003	4.147262	15.89962	6.67922E-05	0.240291733	76	68	55	63	32	36	31
Chst5	-0.96751	4.764522	5.966872	0.01457712	1	26	17	23	10	7	13	9
Ubl7	-0.96637	3.883667	7.018363	0.008067797	1	56	45	66	107	35	52	21
Fam228b	-0.96012	2.094779	4.004886	0.045368571	1	15	14	20	18	11	11	4
Slc7a6os	-0.95135	4.042812	9.879623	0.001671192	1	87	77	56	59	35	43	30
Postn	-0.94727	3.213799	4.197074	0.040493792	1	38	26	18	40	18	20	10
Prelid2	-0.93873	1.643032	3.988544	0.045810634	1	9	15	15	15	9	8	4
Bbs9	-0.93064	3.162742	8.55224	0.003450973	1	49	64	53	41	32	16	31
Cd59a	-0.92698	7.789429	26.91346	2.12772E-07	0.002551565	911	1754	1902	1722	782	962	726
Chac1	-0.90506	5.309371	4.213875	0.040094638	1	29	15	13	8	9	11	6
Pex2	-0.89802	3.427533	4.018358	0.045007523	1	25	27	23	40	14	11	20
Omp	-0.8788	4.77433	6.456049	0.011057525	1	33	20	22	17	18	7	12
Tmem72	-0.87853	3.713056	3.975331	0.046171369	1	115	129	57	93	50	51	56
Acad10	-0.87827	2.695248	3.953012	0.046787539	1	10	30	9	11	7	10	7
Prokr2	-0.86084	2.783888	8.337973	0.00388249	1	61	50	71	54	40	43	16
Sh3bp4	-0.84995	3.839159	5.570031	0.018270434	1	54	38	50	33	32	25	16
Pprc1	-0.82823	3.133513	5.289594	0.021453217	1	46	38	22	33	25	23	11
Zc3h12b	-0.82699	2.689486	5.375078	0.020426424	1	41	32	48	41	25	28	16
Gabra4	-0.8249	1.879528	4.396158	0.036019983	1	34	30	21	18	15	14	14

Iqcc	-0.81785	2.366249	4.571451	0.032509016	1	47	27	24	23	15	25	12
Snap23	-0.80482	5.044379	8.925604	0.002812004	1	74	48	29	34	24	30	25
Smco4	-0.80325	4.947632	6.82545	0.008986791	1	38	31	20	26	16	17	16
Sh2b3	-0.80113	3.620537	5.14093	0.023368121	1	28	41	17	38	23	10	19
Tor3a	-0.79078	4.785847	6.669296	0.009808791	1	28	23	22	23	12	10	19
2010315B(	-0.79068	3.722347	5.053238	0.02457996	1	55	22	45	49	19	34	22
Xpo4	-0.78353	3.225894	4.107998	0.042680859	1	37	32	36	38	19	17	25
Cx3cl1	-0.77624	4.23381	5.684512	0.017115297	1	26	27	16	21	17	11	11
Alg12	-0.77035	3.05238	5.204892	0.022523408	1	42	32	29	48	30	18	18
F2rl2	-0.74711	3.488762	11.43885	0.000719244	0.923024417	92	102	89	99	50	54	64
Tlr4	-0.74418	4.356785	5.512606	0.018879896	1	34	28	15	19	7	24	12
Rars2	-0.74004	4.278673	5.572374	0.018246005	1	58	58	40	39	36	29	22
Map2k6	-0.72665	4.216207	4.989766	0.025497658	1	112	107	111	108	75	59	62
Cbr3	-0.72397	3.956789	4.587383	0.032208156	1	30	41	22	36	23	19	16
Osmr	-0.71136	4.655322	4.536705	0.033175394	1	33	27	31	27	4	29	21
Atf1	-0.70632	4.437888	8.183971	0.004226214	1	51	37	44	42	21	35	24
Helb	-0.68974	3.692853	5.645698	0.017498248	1	25	42	40	29	23	25	15
Fkbp14	-0.6884	3.661387	8.430791	0.003689198	1	63	94	55	59	44	46	35
Dclre1a	-0.6836	3.780647	4.104238	0.04277586	1	38	29	30	48	23	27	18
Mir6236	-0.68328	4.870337	4.434618	0.035217101	1	124	53	87	122	68	60	53
Atp7a	-0.6769	4.422458	6.579729	0.01031466	1	87	103	107	68	27	91	54
Casp3	-0.67502	4.409096	7.644874	0.005693386	1	51	67	75	64	46	33	40
Vwa9	-0.67269	3.28593	5.217233	0.022364111	1	33	47	32	38	25	25	20
Trim30a	-0.64866	4.566332	3.912012	0.047942175	1	30	33	12	20	14	17	14
Akt1s1	-0.64426	4.485446	3.980441	0.04603152	1	107	78	81	102	61	71	46
Eif2ak2	-0.64298	4.689727	9.55351	0.001995674	1	92	93	79	65	55	56	46
Dhdh	-0.63475	3.122685	4.838708	0.027827735	1	58	68	69	45	37	49	30
Pqlc3	-0.63262	4.62937	6.879276	0.008720088	1	76	119	93	106	70	71	49
Homer1	-0.6299	4.709511	11.04058	0.00089139	1	136	169	142	141	92	117	76
Lurap1l	-0.62504	3.638059	4.010244	0.045224618	1	69	63	76	83	43	58	41
Wtip	-0.62458	3.879157	4.80902	0.028311149	1	41	48	49	65	40	36	23
Edrf1	-0.61344	4.237716	6.458607	0.011041614	1	58	65	57	55	36	47	32
Ankrd29	-0.60777	4.811393	6.889609	0.008669823	1	138	135	135	143	78	89	100
Ints2	-0.60637	3.817984	4.411603	0.035695284	1	34	44	39	66	28	35	27
Redrum	-0.60172	3.004106	4.040193	0.044428741	1	42	93	61	66	45	58	27
Galnt5	-0.59382	3.756675	4.855579	0.027556861	1	99	118	118	112	71	109	46
Alpl	-0.59018	5.654889	4.76163	0.029100984	1	31	62	68	44	37	41	24
Mcc	-0.57965	3.662587	3.97229	0.046254827	1	34	41	35	44	17	34	26
Ccdc80	-0.57865	4.640429	4.21147	0.040151517	1	76	98	105	84	62	81	41
Pim1	-0.5728	7.284081	7.175167	0.007391959	1	63	82	77	90	38	61	57
Utp14a	-0.56581	4.551139	5.684495	0.017115469	1	85	60	61	76	48	54	41
Kansl1l	-0.55481	4.738701	5.692947	0.017033226	1	104	124	83	82	86	57	55
Fbxl19	-0.55146	4.945276	8.305117	0.003953354	1	107	107	103	91	76	67	64
Fn3krp	-0.54879	4.093755	4.175152	0.041020876	1	93	48	79	64	69	43	34
Fam76b	-0.54617	4.784033	5.170765	0.022970095	1	68	64	41	59	34	50	35
Lypd2	-0.53936	11.45805	4.126255	0.042222698	1	18	30	30	35	14	24	20
2310009A(	-0.53417	4.466847	3.938604	0.047189919	1	94	121	57	65	50	60	61
Phactr3	-0.5323	3.959448	5.065808	0.024402327	1	149	146	93	121	88	121	58
Rcor3	-0.53126	5.344886	8.414947	0.003721486	1	117	147	109	162	110	96	71
Afmid	-0.52856	4.262801	3.9525	0.046801791	1	39	49	51	56	28	48	26
Ift122	-0.5225	5.417044	4.243873	0.039392213	1	269	249	138	169	135	188	110
Ccng1	-0.52027	5.886056	12.11272	0.000500791	0.720657938	258	301	256	245	172	205	173
1810026J2	-0.51604	4.317069	4.421813	0.035482311	1	67	95	100	100	69	84	39
Nmi	-0.51009	5.123965	4.613214	0.031726521	1	62	48	45	58	30	54	29
Rpf1	-0.5066	4.299049	4.464047	0.034615385	1	44	62	45	43	30	43	29
Cdh4	-0.50457	4.914584	4.197739	0.04047792	1	126	119	161	139	99	111	79
Lrrn3	-0.49823	3.814055	4.421435	0.035490173	1	111	117	113	90	84	82	62
Lrig2	-0.49537	4.720393	5.953624	0.014687063	1	136	120	159	130	79	131	82
Stat2	-0.49307	4.801339	4.262466	0.038963317	1	129	112	135	172	96	133	68
Mafb	-0.4843	6.214428	5.013273	0.025153702	1	61	43	72	46	45	52	23
Chrac1	-0.48147	4.736376	3.970699	0.046298555	1	89	89	77	59	63	42	60
Tspan9	-0.48092	4.534098	4.8701	0.027325916	1	80	60	76	74	49	54	52
G6pdx	-0.47964	6.07466	6.495944	0.010812086	1	66	76	85	86	61	52	54
Plcxd2	-0.47755	5.416823	6.398083	0.011424364	1	250	277	272	252	170	246	153
Tcea1	-0.47565	4.23889	4.322081	0.037620888	1	65	107	45	62	57	60	33
Ugcg	-0.47322	4.522355	4.023595	0.044867987	1	171	168	196	180	144	127	113

Smc2	-0.47211	5.310422	4.289378	0.038351183	1	84	84	77	79	56	50	66
Zfp503	-0.46985	4.660813	6.281907	0.01219768	1	112	143	124	121	100	93	76
D430041D	-0.46896	4.602004	7.419083	0.00645357	1	237	266	216	264	172	177	177
Rhno1	-0.46722	4.329777	4.01923	0.044984257	1	58	74	77	68	39	51	58
Agps	-0.46539	5.073022	4.045709	0.044283774	1	120	133	126	121	91	98	81
Fam129a	-0.46493	5.486646	5.34297	0.020805993	1	124	112	100	84	61	104	64
Csgalnact1	-0.45934	5.121807	3.891535	0.048530045	1	136	159	142	140	96	136	84
Palm2	-0.45758	5.389155	4.533008	0.033247132	1	253	296	287	356	186	303	170
Lingo2	-0.45669	4.033707	4.954959	0.026015947	1	120	140	151	123	118	110	65
Sema6d	-0.44893	5.033145	6.528507	0.010615904	1	235	240	216	235	156	201	151
Cabin1	-0.44834	5.593712	6.251769	0.012406931	1	197	192	184	199	141	167	117
Anks3	-0.44632	4.32597	3.924893	0.047576209	1	76	72	88	93	76	59	46
Nudt13	-0.44587	4.354406	4.157488	0.041450828	1	57	58	42	60	36	46	37
Slc43a3	-0.4398	4.416045	6.520789	0.010662067	1	163	240	203	187	116	188	133
Nol11	-0.43942	4.792473	5.05363	0.024574407	1	84	96	95	82	81	75	42
Prmt3	-0.43732	4.828506	5.943758	0.014769498	1	142	200	137	125	111	132	90
Prkar2a	-0.43669	6.771158	10.95978	0.00093111	1	621	604	544	585	374	497	425
Ercc4	-0.43387	4.195948	5.440574	0.019674199	1	80	81	74	73	59	68	44
Rnf115	-0.43181	5.124789	5.266746	0.021736611	1	165	215	180	139	123	160	105
Tbc1d10a	-0.43064	5.578638	3.863188	0.049356451	1	87	65	90	68	53	71	49
Gpatch1	-0.42909	5.138371	4.135474	0.041993304	1	104	173	126	114	100	86	96
Ints3	-0.4283	4.561003	4.463591	0.034624638	1	95	107	74	83	68	67	63
Gtf2a2	-0.42766	5.094351	4.277647	0.038616765	1	91	160	126	129	93	94	91
Tnip1	-0.42748	6.209553	5.403518	0.020096209	1	400	314	382	412	241	378	232
Chp2	-0.42568	4.599323	4.322524	0.037611087	1	175	273	176	145	143	131	145
Dkk3	-0.42306	7.596362	4.623082	0.031544511	1	412	434	396	359	334	337	226
Htt	-0.4209	4.778634	4.296867	0.038182661	1	159	138	141	170	125	134	84
Necab1	-0.42085	5.409444	4.42457	0.035425029	1	237	218	228	183	160	183	141
Zfp804a	-0.42068	4.671708	6.142521	0.013196907	1	209	194	213	244	147	216	124
Ccdc71	-0.41232	5.605439	4.35442	0.036913028	1	143	114	132	114	92	112	80
Slitrk5	-0.40763	4.909703	5.431992	0.019771119	1	230	268	241	230	149	252	150
Aacs	-0.40613	5.680819	4.268611	0.038822648	1	171	154	166	194	112	167	111
Bbx	-0.40582	6.785675	6.563401	0.010409724	1	450	428	361	428	314	331	292
Psen1	-0.40111	6.487912	8.262769	0.004046635	1	189	250	202	182	145	170	148
Tecpr2	-0.39871	4.854346	4.936395	0.026296825	1	166	161	162	144	122	150	90
Mbd3	-0.39671	5.302801	4.617587	0.031645728	1	143	177	180	113	109	128	109
Ncoa3	-0.39217	5.617883	4.271589	0.038754677	1	93	106	82	103	76	83	60
Coro7	-0.3899	5.1658	4.309987	0.037889255	1	190	186	175	148	173	129	97
Pcyt1b	-0.38971	5.877852	5.939943	0.014801494	1	531	536	466	382	314	453	327
Ccdc6	-0.38909	5.551216	5.065682	0.024404103	1	132	133	137	164	117	136	74
Sphkap	-0.38797	6.280461	5.776059	0.01624593	1	263	336	276	241	212	257	170
Wbp1l	-0.38742	5.839386	6.113969	0.013411762	1	121	127	146	147	81	152	80
Bnip3l	-0.38209	6.80403	10.47038	0.001213036	1	289	336	339	297	232	258	230
Ptprt	-0.38182	5.136015	8.007259	0.004659019	1	311	312	261	256	237	231	185
Irf2	-0.37894	6.530685	4.995684	0.025410612	1	209	244	163	233	130	188	167
Ar	-0.37582	4.544513	4.62421	0.03152377	1	194	177	150	148	121	135	127
Polr3g	-0.3721	6.627212	8.100443	0.004425444	1	430	472	412	397	305	388	295
Rab27b	-0.37144	6.611819	8.386118	0.003780975	1	191	218	193	210	149	186	135
Scaf4	-0.36938	5.185503	5.398309	0.020156274	1	118	138	106	113	82	120	74
Cttnbp2nl	-0.3582	6.031163	5.310809	0.021193508	1	193	202	195	199	143	182	136
Rims2	-0.35616	5.295816	8.21907	0.004145244	1	288	347	313	313	209	286	239
Abhd6	-0.35331	5.406622	4.042136	0.044377616	1	181	249	215	237	178	188	149
Eml1	-0.34634	5.070892	4.958451	0.025963448	1	252	257	264	258	185	238	184
Dnajb4	-0.34629	5.771974	8.238156	0.004101879	1	288	365	347	282	266	295	195
Ppef1	-0.34385	4.917225	4.092034	0.043085762	1	293	263	248	245	210	247	165
Trappc3l	-0.34354	6.374618	5.039407	0.024776975	1	741	897	746	667	596	687	512
Prkcd	-0.34278	6.371449	3.90378	0.048177608	1	330	375	275	353	234	344	214
Xrn1	-0.33817	6.0592	5.460476	0.019451342	1	191	221	175	193	139	189	134
Mesdc2	-0.33721	5.595568	4.771773	0.028930016	1	256	259	201	210	205	218	129
Hspa12a	-0.33668	7.154547	5.776985	0.016237373	1	1084	1032	1052	1221	822	1145	671
Gatc	-0.33073	4.765705	4.051014	0.044144833	1	137	148	131	127	102	127	94
Serpinb1a	-0.33046	6.150062	5.741887	0.016564985	1	343	360	406	404	325	307	266
Tpcn1	-0.32992	6.422647	5.928154	0.014900847	1	235	219	220	171	162	188	152
Grik1	-0.32861	5.954786	4.221345	0.039918501	1	391	444	485	430	323	465	267
Cyfip2	-0.31601	7.393866	6.456783	0.011052958	1	859	996	865	828	624	879	631
Tmx3	-0.30778	6.709941	7.521918	0.006095275	1	500	527	481	482	384	456	361

Kcnh7	-0.3064	5.4163	3.848512	0.049790149	1	344	447	427	328	273	372	287
Rtn1	-0.30588	9.020087	5.24765	0.021976429	1	3044	4352	3838	2984	2755	3392	2438
Phactr2	-0.30497	7.496238	3.905609	0.048125172	1	962	1208	930	884	758	911	729
Tbrg1	-0.30381	6.772456	5.227456	0.022233022	1	295	229	256	243	177	236	206
Nedd4	-0.30355	8.798239	5.479279	0.019243192	1	2179	2538	1823	1973	1552	1751	1774
Cdkn2aipn	-0.29769	5.609023	4.045632	0.044285801	1	192	163	144	147	141	127	123
Nsg2	-0.29688	5.676339	5.545281	0.018530579	1	399	427	394	347	378	309	263
Lin7a	-0.29616	5.900244	4.194226	0.040561876	1	529	553	577	498	390	583	354
Scn1a	-0.28645	6.699604	7.683769	0.005571966	1	819	959	875	805	687	826	608
Rbm27	-0.28628	6.446566	4.87746	0.027209626	1	236	263	236	261	180	214	212
Slmap	-0.2834	6.744511	3.867035	0.049243435	1	381	403	361	355	264	410	255
Knop1	-0.28182	7.205628	4.292711	0.038276086	1	384	437	415	333	321	401	249
Rassf3	-0.27912	6.463374	4.553145	0.032858347	1	353	358	301	257	235	315	232
Nrip1	-0.27582	7.314109	4.090507	0.043124708	1	334	371	351	325	308	336	214
Cdc5l	-0.27362	6.562898	5.219729	0.022332035	1	356	472	355	358	323	370	260
Homer2	-0.27322	8.514333	4.512046	0.033656957	1	308	330	356	370	271	295	274
Slitrk3	-0.27255	5.995012	5.021066	0.025040742	1	498	513	464	490	370	504	348
Cnst	-0.26841	6.031513	4.088878	0.043166287	1	399	423	388	412	344	413	258
Hecw1	-0.25664	6.85252	4.750034	0.02929772	1	833	1026	851	753	683	883	605
Scaf11	-0.25456	7.46759	4.40993	0.035730299	1	569	698	548	510	496	474	469
Ube2q1	-0.25145	6.347393	3.886152	0.048685841	1	321	370	313	291	283	337	199
Mkrn1	-0.24734	6.711501	4.106075	0.042729424	1	311	354	286	296	270	305	212
Kcna2	-0.24163	8.282401	5.59316	0.018030744	1	2306	2560	2502	2498	1980	2529	1761
Vps35	-0.23798	7.71012	4.662304	0.030831799	1	819	1012	844	787	774	826	595
Ablim1	-0.23638	8.809637	4.784289	0.028720508	1	781	952	842	778	633	823	664
Ppp1r12a	-0.22469	8.3118	4.150518	0.041621793	1	607	688	516	691	498	679	435
Arid1b	-0.21471	6.942678	3.903176	0.048194904	1	350	363	323	315	285	353	236
Ube3a	-0.21452	7.609609	5.098424	0.023947592	1	916	1047	930	883	760	961	713
Ppp1r7	-0.20407	6.485586	3.871516	0.049112138	1	391	454	429	480	356	424	356

Table S5. Differentially expressed genes in the TG of miR-183C MS-CKO vs age- and sex-matched WT control mice.

	logFC	logCPM	LR	Pvalue	FDR	WT1	WT2	WT3	WT4	WT5	MS-CKO1	MS-CKO2	MS-CKO3	MS-CKO4	MS-CKO5
**Upregulated genes**															
Gp9	5.183679	-0.48114	8.710827	0.003163	0.574754	0	0	0	0	0	12	0	3	4	2
LOC10263	4.926109	0.222768	12.35184	0.000441	0.278057	0	0	0	0	0	9	4	4	1	0
Actl9	4.855649	-0.7191	7.578368	0.005907	0.735366	0	0	0	0	0	0	0	3	4	8
Gm40812	4.668406	-0.70467	9.604594	0.001941	0.468633	0	0	0	0	0	6	7	0	1	1
Gpr151	4.598768	-0.67018	9.34274	0.002239	0.502051	0	0	0	0	0	2	7	3	0	2
Fam209	4.562807	-0.7044	7.951564	0.004805	0.698042	0	0	0	0	0	6	5	3	0	0
Gm9903	4.547494	-0.49721	7.209135	0.007253	0.826025	0	0	0	0	0	0	8	6	0	0
Aoah	4.545228	-0.57193	9.324151	0.002262	0.502051	0	0	0	0	0	5	8	0	1	0
Gm32688	4.538073	-0.49753	9.61637	0.001929	0.468633	0	0	0	0	0	6	0	4	1	2
Gm42012	4.511072	-0.28633	10.06352	0.001512	0.415328	0	0	0	0	0	8	0	1	3	1
9530027J0	4.510301	-0.19187	12.45629	0.000417	0.275262	0	0	0	0	0	0	8	0	2	3
Gm8773	4.510089	-0.5086	10.42045	0.001246	0.403928	0	0	0	0	0	1	8	0	1	3
Rpl17-ps8	4.501937	0.019201	13.03571	0.000306	0.26816	0	0	0	0	0	0	7	3	1	2
Treml2	4.500997	-0.62171	5.19223	0.022688	1	0	0	0	0	0	0	13	0	1	0
LOC10524	4.491588	0.25894	10.13638	0.001454	0.411802	0	0	0	0	0	6	0	0	1	5
Tnfsf9	4.487182	-0.73698	5.591473	0.018048	1	0	0	0	0	0	1	0	2	0	8
AW822252	4.409025	-0.75418	4.236014	0.039575	1	0	0	0	0	0	0	0	0	1	9
6430590A	4.398948	-0.56068	4.598792	0.031994	1	0	0	0	0	0	0	0	0	6	5
Ltc4s	4.387966	-0.40411	11.21305	0.000812	0.357189	0	0	0	0	0	2	4	4	1	1
Hpx	4.378418	-0.68541	4.219372	0.039965	1	0	0	0	0	0	0	0	0	2	8
Gm39408	4.368238	-0.13869	7.950539	0.004807	0.698042	0	0	0	0	0	0	2	5	5	0
Tmed1	4.343339	0.215777	11.41771	0.000727	0.348954	0	0	0	0	0	0	9	3	0	0
Gm39668	4.330081	-0.26929	10.77259	0.00103	0.386047	0	0	0	0	0	0	0	3	5	3
Gm41244	4.306155	-0.72114	8.745347	0.003104	0.574754	0	0	0	0	0	7	1	1	0	2
4931403E2	4.30393	-0.3285	7.704399	0.005509	0.725022	0	0	0	0	0	0	3	0	0	7
Gm41072	4.302927	-0.66904	7.542237	0.006027	0.74767	0	0	0	0	0	0	5	0	3	3
Gm30086	4.297359	-0.75893	4.662018	0.030837	1	0	0	0	0	0	0	10	0	2	0
Igfn1	4.289987	0.340781	19.72932	8.92E-06	0.063459	1	0	0	0	0	3	9	5	6	5
Elmo3	4.288509	3.85859	16.61796	4.57E-05	0.149517	0	0	0	0	0	1	5	3	1	1
Gm30440	4.265361	-0.51126	4.399668	0.035946	1	0	0	0	0	0	4	0	7	0	0
Gm26708	4.252867	-0.58881	5.613811	0.017819	1	0	0	0	0	0	9	0	0	2	0
Rag1	4.250937	-0.75737	4.5068	0.03376	1	0	0	0	0	0	9	0	0	2	0
Gm31963	4.248015	-0.37688	6.376788	0.011562	0.988037	0	0	0	0	0	6	0	0	0	4
Chil3	4.247355	-0.52541	5.189767	0.02272	1	0	0	0	0	0	0	9	0	0	2
AW551984	4.243342	-0.61976	8.724196	0.00314	0.574754	0	0	0	0	0	2	2	0	7	0
LOC10816	4.197853	0.286423	11.57271	0.000669	0.348951	0	0	0	0	0	1	3	0	2	4
Gm31279	4.17271	-0.38064	10.0841	0.001496	0.413873	0	0	0	0	0	0	5	0	2	3
Gm39472	4.166817	-0.812	4.135459	0.041994	1	0	0	0	0	0	0	7	0	0	3
Ces1a	4.157253	-0.72058	4.990472	0.025487	1	0	0	0	0	0	9	0	0	0	1
Gm35587	4.152021	-0.06001	10.34381	0.001299	0.406402	0	0	0	0	0	5	1	0	3	1
Gm6410	4.13821	-0.52347	7.329196	0.006784	0.797626	0	0	0	0	0	2	0	6	2	0
Cyp1a1	4.130096	-0.79318	6.398853	0.011419	0.982834	0	0	0	0	0	1	0	4	5	0
LOC10263	4.123312	0.032493	7.248334	0.007097	0.816013	0	0	0	0	0	2	2	0	0	5
Dnali1	4.120758	-0.60132	8.717071	0.003152	0.574754	0	0	0	0	0	0	0	0	4	5
5330432E	4.120662	-0.8123	4.026268	0.044797	1	0	0	0	0	0	8	0	0	2	0
Btg4	4.098026	-0.7937	4.338022	0.03727	1	0	0	0	0	0	7	3	0	0	0
LOC10816	4.080278	-0.82997	6.151656	0.013129	1	0	0	0	0	0	0	0	1	5	3
Gm41452	4.025086	-0.62078	5.149472	0.023253	1	0	0	0	0	0	0	0	5	3	1
Gm41464	4.02487	-0.41534	6.741641	0.009419	0.931074	0	0	0	0	0	0	0	5	3	1
Carm1	3.994448	-0.57517	6.662182	0.009848	0.943727	0	0	0	0	0	2	0	5	2	0
Gm41212	3.991392	-0.79342	4.271799	0.03875	1	0	0	0	0	0	0	7	0	0	2
Zfp352	3.984816	-0.70237	4.828485	0.027993	1	0	0	0	0	0	0	0	4	0	4
COX2	3.980001	-0.82948	4.186788	0.04074	1	0	0	0	0	0	4	0	0	0	4
Gm5089	3.970967	-0.77562	5.608527	0.017873	1	0	0	0	0	0	0	6	2	0	1
Gm39446	3.969126	-0.42375	7.902029	0.004938	0.704155	0	0	0	0	0	6	3	0	0	0
Rhox5	3.963991	0.21668	8.102842	0.00442	0.668064	0	0	0	0	0	0	4	2	3	0
Gm30012	3.953848	-0.59312	6.637443	0.009986	0.943727	0	0	0	0	0	4	4	0	1	0
LOC10524	3.953444	-0.81232	4.205725	0.040288	1	0	0	0	0	0	6	3	0	0	0
Gm31418	3.952346	-0.61954	6.248122	0.012433	0.9915	0	0	0	0	0	0	8	0	0	1
Gm6654	3.926278	0.463521	10.47809	0.001208	0.403928	0	0	0	0	0	0	0	0	3	5
C130080G	3.926254	-0.77489	7.09348	0.007736	0.854323	0	0	0	0	0	2	0	1	2	3
Stpg2	3.915899	-0.22959	6.784988	0.009193	0.924097	0	0	0	0	0	0	9	0	0	0
Rbm3os	3.907344	-0.25896	5.714585	0.016825	1	0	0	0	0	0	0	9	0	0	0
Gm33703	3.901853	-0.02928	7.927035	0.00487	0.698042	0	0	0	0	0	0	0	6	0	2

Glipr1	3.891059	-0.79246	5.245205	0.022007	1	0	0	0	0	0	0	5	0	0	3
Gm38862	3.888097	0.790149	10.01558	0.001552	0.418551	0	0	0	0	0	3	2	0	0	3
Htr2c	3.861512	-0.60262	11.15259	0.000839	0.359393	0	0	0	0	0	1	3	0	2	2
Gm21759	3.860308	-0.75653	6.441725	0.011147	0.980503	0	0	0	0	0	4	0	0	3	1
Gm29874	3.857646	-0.82943	4.094674	0.043019	1	0	0	0	0	0	0	0	0	2	5
Vmn2r1	3.835472	-0.796	4.927335	0.026435	1	0	0	0	0	0	2	0	5	1	0
Gm32701	3.834692	-0.65218	3.922645	0.04764	1	0	0	0	0	0	0	0	6	2	0
LOC10263(	3.832953	-0.79415	5.421421	0.019891	1	0	0	0	0	0	0	4	3	0	1
Corin	3.810361	-0.07323	10.65761	0.001096	0.402413	0	1	0	0	0	0	5	6	4	5
LOC10816(	3.809023	-0.29188	5.238445	0.022093	1	0	0	0	0	0	0	1	0	7	0
Tmem28	3.807502	-0.82953	4.956018	0.026	1	0	0	0	0	0	0	0	0	3	4
Gm39785	3.803197	-0.19381	5.753897	0.016452	1	0	0	0	0	0	0	2	0	6	0
Gm39704	3.79864	-0.66649	5.903384	0.015112	1	0	0	0	0	0	1	0	0	2	4
Krtdap	3.794965	-0.81283	3.845513	0.049879	1	0	0	0	0	0	0	2	0	6	0
Gm38952	3.790733	-0.42196	7.587365	0.005878	0.735366	0	0	0	0	1	2	0	6	5	6
Gm27230	3.788361	-0.84875	3.955447	0.04672	1	0	0	0	0	0	0	3	0	0	4
4921507G(	3.779867	-0.4161	7.487393	0.006213	0.759767	0	0	0	0	0	0	7	0	1	0
LOC10524:	3.768827	-0.83175	4.064302	0.043799	1	0	0	0	0	0	0	5	0	3	0
Gm35219	3.767737	-0.70312	5.392245	0.020226	1	0	0	0	0	0	4	0	0	0	3
Gm41189	3.762667	-0.86816	5.873784	0.015368	1	0	0	0	0	0	0	0	1	3	3
4930596I2	3.762642	-0.55779	5.448539	0.019585	1	0	0	0	0	0	0	0	0	4	3
C130083M	3.759977	-0.15105	12.61745	0.000382	0.274974	1	0	0	0	0	0	6	3	5	5
Gm31906	3.745828	-0.83032	4.215114	0.040065	1	0	0	0	0	0	0	0	5	0	2
Gm34321	3.716375	-0.66844	4.589281	0.032173	1	0	0	0	0	0	0	0	0	0	6
Gm39170	3.708073	1.142134	10.21481	0.001393	0.406676	0	0	0	0	0	0	0	1	2	4
Gm33648	3.701681	-0.7043	5.193443	0.022672	1	0	0	0	0	0	1	0	5	0	1
1700109H(	3.698469	-0.31718	7.47244	0.006265	0.761458	0	0	0	0	0	0	2	0	3	2
Gnrhr	3.688831	-0.5141	6.603462	0.010178	0.943727	0	0	0	0	0	6	0	0	0	1
Ccdc64b	3.685057	4.477891	10.7824	0.001025	0.386047	0	0	0	0	0	0	0	3	0	4
Icos	3.684771	-0.45233	6.342851	0.011786	0.9915	0	0	0	0	0	2	0	0	4	1
Gm39526	3.683649	-0.23311	6.661942	0.009849	0.943727	0	0	0	0	0	1	0	0	5	1
Bnipl	3.682174	5.845432	10.80843	0.00101	0.386047	0	0	0	0	0	0	2	2	0	3
Ap1s3	3.681468	4.470898	10.72423	0.001057	0.392181	0	0	0	0	0	5	0	1	0	1
Klc3	3.672268	0.790104	9.739886	0.001803	0.444309	0	0	0	0	0	5	0	0	2	0
Gm34389	3.671726	0.337047	8.736667	0.003119	0.574754	0	0	0	0	0	3	0	4	0	0
Thpo	3.669365	-0.35921	6.008716	0.014235	1	0	0	0	0	0	3	2	0	1	1
Scnn1b	3.667998	0.476703	9.450208	0.002111	0.486887	0	0	0	0	0	0	0	0	7	0
Gpr18	3.666369	-0.31107	6.191743	0.012835	1	0	0	0	0	0	0	2	0	4	1
Pdzd9	3.665539	-0.23989	4.625012	0.031509	1	0	0	0	0	0	0	0	7	0	0
Gm33148	3.664585	-0.79323	4.109431	0.042645	1	0	0	0	0	0	0	0	7	0	0
Fam166a	3.664053	-0.46727	6.363754	0.011647	0.9915	0	0	0	0	0	1	0	5	1	0
Gm42323	3.661629	-0.52915	5.074668	0.024278	1	0	0	0	0	0	2	0	5	0	0
Gm33851	3.653898	-0.32921	6.850799	0.00886	0.913142	0	0	0	0	0	4	0	0	3	0
Gm30101	3.652665	-0.75709	4.692454	0.030295	1	0	0	0	0	0	0	1	6	0	0
Gm3696	3.651536	-0.62913	3.941431	0.047111	1	0	0	0	0	0	0	4	2	0	1
Bcas3os2	3.65116	-0.70367	4.8109	0.02828	1	0	0	0	0	0	6	0	1	0	0
3300002P1	3.650179	-0.7401	4.49914	0.033912	1	0	0	0	0	0	0	0	3	4	0
Gm36841	3.649506	-0.67041	4.684425	0.030437	1	0	0	0	0	0	2	0	2	3	0
Cct8l1	3.648159	-0.70386	4.244639	0.039374	1	0	0	0	0	0	5	0	1	1	0
Gabra6	3.646437	-0.68622	4.098568	0.04292	1	0	0	0	0	0	7	0	0	0	0
Gm9159	3.645986	0.046621	12.27672	0.000459	0.281521	1	0	0	0	0	8	4	4	1	1
Gm36745	3.644911	-0.65908	4.025319	0.044822	1	0	0	0	0	0	6	0	0	1	0
Gm30604	3.640676	-0.83096	5.367608	0.020514	1	0	0	0	0	0	0	1	2	4	0
Gm13283	3.639415	-0.81305	3.949524	0.046885	1	0	0	0	0	0	2	0	0	5	0
Gm15455	3.625311	-0.53417	6.162116	0.013052	1	0	0	0	0	0	0	3	1	3	0
Recql4	3.613544	-0.49546	5.00288	0.025305	1	0	0	0	0	0	0	3	0	4	0
Gm32838	3.607266	-0.85009	3.964581	0.046467	1	0	0	0	0	0	0	3	0	4	0
Gm35855	3.602626	0.321224	10.81791	0.001005	0.386047	0	0	0	0	1	0	10	3	5	0
Gm17619	3.601631	-0.28739	4.617499	0.031647	1	0	0	0	0	0	0	4	0	3	0
Gm41730	3.601538	-0.83148	4.861311	0.027465	1	0	0	0	0	0	0	5	2	0	0
Gm14164	3.594817	-0.85022	3.869623	0.049168	1	0	0	0	0	0	0	5	2	0	0
Gm41702	3.577331	-0.81124	4.961536	0.025917	1	0	0	0	0	0	2	0	0	1	3
Gm29932	3.573459	-0.25835	10.21109	0.001396	0.406676	0	1	0	0	0	3	0	8	4	2
Efcab11	3.561989	0.984758	13.08765	0.000297	0.267345	1	0	0	0	0	9	4	2	2	0
Gm41148	3.545359	-0.61816	4.9873	0.025534	1	0	0	0	0	0	0	0	4	0	2
Gm39819	3.54241	-0.70216	6.080904	0.013665	1	0	0	0	0	0	0	2	0	1	3
4930443O:	3.538319	-0.60524	5.784547	0.016168	1	0	0	0	0	0	0	0	4	0	2
Irs4	3.532381	-0.77498	4.549141	0.032935	1	0	0	0	0	0	0	0	0	4	2

LOC10816{	3.499782	-0.8495	5.360615	0.020597	1	0	0	0	0	0	0	0	4	1	1
Gm28497	3.489625	2.300815	8.999144	0.002701	0.552121	0	0	0	0	0	0	0	0	3	3
Gm38594	3.485646	-0.723	4.418277	0.035556	1	0	0	0	0	0	0	0	0	5	1
Prr32	3.485623	-0.54139	6.086009	0.013626	1	0	0	0	0	0	0	1	3	1	1
Kcne2	3.470958	-0.52816	6.41094	0.011342	0.980859	0	0	0	0	0	0	2	1	2	1
Gpr39	3.470855	1.85626	8.554106	0.003447	0.590595	0	0	0	0	0	6	0	0	0	0
Fat2	3.467234	1.421589	8.402408	0.003747	0.610501	0	0	0	0	0	0	0	0	6	0
2010016I1	3.46721	1.630972	8.400047	0.003752	0.610501	0	0	0	0	0	0	0	0	6	0
LOC10263:	3.464707	0.475803	10.11022	0.001474	0.412723	0	1	0	0	0	7	2	0	6	1
5730507C(	3.462586	0.318322	7.707201	0.0055	0.725022	0	0	0	0	0	3	0	0	3	0
Nron	3.460634	-0.29638	5.035761	0.024829	1	0	0	0	0	0	0	0	5	1	0
Gm41106	3.459252	-0.84976	4.571465	0.032509	1	0	0	0	0	0	0	0	5	1	0
LOC10816:	3.457353	-0.79368	4.385853	0.036238	1	0	0	0	0	0	2	0	4	0	0
Gm34205	3.455707	-0.67013	4.920622	0.026538	1	0	0	0	0	0	0	3	0	2	1
Gm11837	3.451296	-0.51979	4.749255	0.029311	1	0	0	0	0	0	0	0	0	0	5
A230072E:	3.450046	-0.30558	5.237106	0.02211	1	0	0	0	0	0	6	0	0	0	0
2810407A:	3.448482	-0.7765	4.904986	0.026779	1	0	0	0	0	0	0	0	2	4	0
Slc10a2	3.446267	-0.65274	4.69996	0.030163	1	0	0	0	0	0	5	0	0	1	0
Nrap	3.440964	-0.64008	4.96073	0.025929	1	0	0	0	0	0	0	0	0	6	0
1810073O(	3.440665	-0.79815	4.053202	0.044088	1	0	0	0	0	0	0	0	1	5	0
Gm39551	3.440144	0.061456	5.702201	0.016944	1	0	0	0	0	0	0	5	0	0	1
Gm39210	3.440019	-0.24314	5.680639	0.017153	1	0	0	0	0	0	0	5	0	0	1
Gm30139	3.432338	0.643978	11.50983	0.000692	0.348954	0	0	1	0	0	0	0	8	7	0
2810408B:	3.431946	-0.59887	4.016631	0.045054	1	0	0	0	0	0	0	2	4	0	0
Gm32930	3.430097	-0.83134	6.535471	0.010574	0.954012	0	0	0	0	0	1	3	0	2	0
Otol1	3.413266	-0.24607	5.197271	0.022622	1	0	1	0	0	1	19	0	5	0	2
Gm38861	3.412974	-0.15241	6.41848	0.011294	0.980859	0	0	0	0	0	0	5	0	1	0
A230107N(	3.403903	-0.31532	8.696303	0.003189	0.57644	0	0	0	0	1	2	7	3	0	3
Patl2	3.403331	-0.52822	4.954096	0.026029	1	0	0	1	0	0	6	0	1	5	2
Gm26761	3.400359	0.101157	8.933284	0.0028	0.552875	0	1	0	0	0	1	0	7	5	2
Lmod3	3.398899	-0.5337	5.376019	0.020415	1	0	0	0	0	0	1	5	0	0	0
Plscr5	3.398173	-0.2514	5.982885	0.014445	1	0	0	0	0	0	0	6	0	0	0
Gm31814	3.382241	-0.38607	4.918289	0.026574	1	0	0	0	0	0	0	6	0	0	0
Tead4	3.374191	-0.02813	5.735048	0.01663	1	0	0	0	0	0	0	0	1	0	4
Rxfp2	3.329892	-0.70919	4.740294	0.029464	1	0	0	0	0	0	0	2	0	0	3
Plekhs1	3.327708	6.549279	14.62584	0.000131	0.195	0	0	0	0	1	3	4	3	4	0
Gm12923	3.307631	-0.6532	4.317926	0.037713	1	0	0	0	0	0	0	0	0	3	2
Fam227b	3.301539	-0.86825	5.421419	0.019891	1	0	0	0	0	0	0	0	2	1	2
4933402D:	3.292393	-0.86836	5.986101	0.014419	1	0	0	0	0	0	1	0	2	0	2
Snord15a	3.276649	1.704523	12.71818	0.000362	0.274974	0	0	4	0	1	7	12	0	14	11
Gm30401	3.276317	-0.13737	10.31491	0.00132	0.406676	0	0	2	0	0	1	11	2	2	5
A330009N:	3.263759	-0.81145	5.222703	0.022294	1	0	0	0	0	0	2	0	2	0	1
LOC10816{	3.261027	-0.36549	5.515029	0.018854	1	0	0	0	0	1	4	1	4	5	0
LOC10263:	3.2573	-0.79358	4.878505	0.027193	1	0	0	0	0	0	2	0	0	2	1
Gm34194	3.257249	-0.69017	5.237588	0.022104	1	0	0	0	0	0	3	0	0	1	1
Gm34513	3.246251	0.689058	11.3163	0.000768	0.357189	0	0	1	0	0	3	2	3	3	2
Muc20	3.240337	6.527517	7.705298	0.005506	0.725022	0	0	0	0	0	3	0	0	1	1
Gm36742	3.234525	-0.90837	5.640493	0.01755	1	0	0	0	0	0	0	2	0	2	1
Acox2	3.230157	-0.60312	5.078786	0.02422	1	0	0	0	0	0	0	0	5	0	0
LOC10816(	3.227809	1.453667	7.199852	0.007291	0.826025	0	0	0	0	0	3	2	0	0	0
Prlh	3.225118	0.262269	5.90457	0.015102	1	0	0	0	0	0	4	0	1	0	0
Slc12a1	3.223458	-0.66979	4.86768	0.027364	1	0	0	0	0	0	0	3	0	1	1
Gm34759	3.222515	-0.8312	4.146101	0.041731	1	0	0	0	0	0	0	0	4	1	0
Gm36853	3.216649	-0.43818	4.975288	0.025712	1	0	0	0	0	0	5	0	0	0	0
Cyp26a1	3.216225	1.148087	4.112499	0.042567	1	1	0	0	0	0	0	4	1	4	4
Gm40652	3.215488	-0.61884	5.872184	0.015382	1	0	0	0	0	1	1	1	8	1	2
Mia2	3.213533	-0.42181	4.93499	0.026318	1	0	0	0	0	0	0	4	0	0	1
Foxr1	3.211208	-0.88911	5.347903	0.020747	1	0	0	0	0	0	3	1	1	0	0
Hist1h1b	3.209681	2.68962	6.757024	0.009338	0.928016	0	0	0	0	0	1	4	0	0	0
LOC10816:	3.208778	-0.50548	4.399343	0.035953	1	0	0	0	0	0	2	0	0	3	0
Gm33676	3.206422	-0.6869	4.484182	0.03421	1	0	0	0	0	0	0	0	0	5	0
Gm35394	3.196579	-0.6058	4.598829	0.031994	1	0	0	0	0	0	0	1	0	4	0
Gm41151	3.19514	-0.49865	4.072241	0.043593	1	0	0	0	0	0	2	1	0	2	0
Muc6	3.186773	0.082633	7.792058	0.005248	0.725022	0	0	1	0	0	2	3	2	1	4
Mir484	3.184266	-0.81167	5.133366	0.02347	1	0	0	0	0	0	1	3	0	1	0
Gm39358	3.176316	-0.27874	7.770351	0.005311	0.725022	0	1	0	1	0	4	8	4	4	1
4930448F1	3.158899	-0.92705	3.910856	0.047975	1	0	0	0	0	0	0	0	0	0	4
Gm35172	3.158899	-0.92705	3.910856	0.047975	1	0	0	0	0	0	0	0	0	0	4

Gm38630	3.158899	-0.92705	3.910856	0.047975	1	0	0	0	0	0	0	0	0	0	4
LOC10816	3.158899	-0.92705	3.910856	0.047975	1	0	0	0	0	0	0	0	0	0	4
Vmn1r183	3.158899	-0.92705	3.910856	0.047975	1	0	0	0	0	0	0	0	0	0	4
LOC10524	3.158569	0.478368	10.0354	0.001536	0.418522	0	0	1	1	0	5	0	0	10	4
Gm15545	3.156062	-0.2377	3.981397	0.046005	1	0	0	0	0	0	0	0	0	0	4
Slc22a14	3.144604	-0.7591	3.99987	0.045504	1	0	0	0	0	0	0	5	0	0	0
Tas1r1	3.144603	-0.47858	4.245273	0.03936	1	0	0	0	0	1	3	0	2	3	4
Zc3h12d	3.137836	-0.54167	4.525383	0.033396	1	0	1	0	0	0	2	0	4	6	1
Ern2	3.131419	-0.66797	6.177202	0.012941	1	0	0	0	0	1	3	0	3	3	3
Tfap2c	3.120929	5.155689	12.3912	0.000431	0.277119	0	0	0	0	1	3	3	1	4	1
Gm30618	3.097219	-0.27434	5.895328	0.015181	1	0	0	0	0	2	4	6	5	2	3
Glyat	3.090862	1.092783	11.84371	0.000579	0.322039	0	0	2	0	0	4	2	2	11	0
Gm34050	3.08697	-0.68995	4.253829	0.039162	1	0	0	0	0	0	0	0	0	1	3
Sprr1a	3.07433	2.897486	10.19885	0.001405	0.406676	0	0	15	0	0	0	61	38	0	5
Epha3	3.0724	-0.53998	6.611307	0.010133	0.943727	1	0	1	0	0	6	3	0	3	6
Appbp2os	3.063644	-0.42169	4.807243	0.02834	1	0	0	0	0	0	0	0	0	1	3
Gm38592	3.057345	-0.18743	4.932677	0.026353	1	0	0	0	0	0	0	0	0	1	3
Cxcr6	3.050456	-0.58992	3.873796	0.049045	1	0	0	0	0	0	0	0	2	0	2
Gm39643	3.048793	-0.35008	5.090008	0.024064	1	0	0	0	0	0	0	0	0	1	3
Gm35677	3.0245	-0.70457	4.081514	0.043355	1	0	0	0	0	0	1	1	0	0	2
Mrgprx1	3.011034	-0.54062	5.377351	0.0204	1	1	0	0	0	1	2	12	1	0	4
Gm35102	3.009583	-0.37928	7.313123	0.006845	0.799565	0	0	0	1	0	5	4	0	1	1
Prss8	3.001809	5.90271	11.23971	0.000801	0.357189	0	0	0	0	1	5	5	0	1	0
Gm36287	2.988929	1.193622	9.234853	0.002375	0.514612	1	0	0	0	0	0	0	8	3	0
4930525G	2.984815	0.168928	4.418189	0.035558	1	0	0	0	0	2	0	10	7	0	2
Kif2c	2.980988	2.076713	9.912559	0.001642	0.430212	1	0	0	0	0	3	5	0	3	0
Ptgs2os2	2.980776	-0.62669	4.551146	0.032897	1	0	0	0	0	0	3	0	0	0	1
Gm29875	2.97648	-0.90799	4.831664	0.027942	1	0	0	0	0	0	2	0	0	1	1
Gm31436	2.97449	-0.68827	5.008329	0.025226	1	0	0	0	0	0	2	0	0	1	1
1700056E	2.97155	0.802373	20.62058	5.60E-06	0.063459	2	1	0	2	2	13	20	12	5	3
Gm30908	2.963714	-0.86831	4.943355	0.026191	1	0	0	0	0	0	0	1	0	2	1
Aqp5	2.955929	8.464529	6.184794	0.012885	1	0	0	0	0	0	0	0	0	3	1
Ankk1	2.953727	4.800681	6.108264	0.013455	1	0	0	0	0	0	0	0	0	4	0
Myl7	2.952789	0.542246	5.547823	0.018504	1	0	0	0	0	0	0	0	4	0	0
Krt75	2.952575	2.521894	5.977862	0.014487	1	0	0	0	0	0	4	0	0	0	0
Gm33977	2.947784	0.585588	5.511034	0.018897	1	0	0	0	0	0	2	0	1	1	0
Gm35531	2.947166	-0.50791	4.327489	0.037502	1	0	0	0	0	0	0	0	4	0	0
LOC10816	2.945696	-0.11561	3.985765	0.045886	1	0	0	0	0	0	0	0	4	0	0
Gm42237	2.945214	-0.62258	3.86947	0.049172	1	0	0	0	0	0	0	0	4	0	0
BC025446	2.945071	0.590681	5.888119	0.015243	1	0	0	0	0	0	0	2	0	2	0
Gm32449	2.9429	-0.46589	4.894027	0.02695	1	0	0	0	0	0	0	0	2	2	0
LOC10263	2.94217	-0.7755	4.223873	0.039859	1	0	0	0	0	0	0	0	3	1	0
LOC10263	2.942119	0.491814	5.039485	0.024776	1	0	0	0	0	0	4	0	0	0	0
Gm13582	2.940363	-0.44649	4.173194	0.041068	1	0	0	0	0	0	2	0	2	0	0
Gm30408	2.938161	-0.09607	4.757376	0.029173	1	0	0	0	0	0	3	0	0	1	0
LOC10816	2.936929	-0.6934	4.122967	0.042305	1	0	0	0	0	0	0	0	2	2	0
Gm6109	2.936109	-0.47735	4.546524	0.032986	1	0	0	0	0	0	3	0	0	1	0
Sdr39u1	2.935791	0.061784	4.646615	0.031115	1	0	0	0	0	0	2	0	0	2	0
Gm41177	2.934833	-0.52355	7.344369	0.006727	0.794981	0	1	2	0	0	3	6	2	1	10
Otogl	2.930849	-0.88864	3.946248	0.046976	1	0	0	0	0	0	3	0	0	1	0
Gm2511	2.930446	-0.84964	3.899128	0.048311	1	0	0	0	0	0	0	0	1	3	0
Gm5544	2.930167	-0.83112	3.863872	0.049336	1	0	0	0	0	0	0	0	1	3	0
E2f7	2.928717	3.368234	14.5636	0.000136	0.195	2	0	0	0	0	2	5	3	5	2
LOC10263	2.926977	-0.16225	4.615524	0.031684	1	0	0	0	0	0	3	1	0	0	0
A230056J0	2.924463	-0.51104	4.056059	0.044013	1	0	0	0	0	1	5	0	0	6	0
Gm40194	2.922593	1.898566	8.677938	0.003221	0.576484	0	0	1	0	0	0	0	1	4	5
Fam3b	2.911868	-0.24736	4.380667	0.036349	1	0	0	0	0	0	2	2	0	0	0
Gm38482	2.901516	-0.20807	4.318751	0.037695	1	0	0	0	0	0	0	3	1	0	0
Gm30199	2.892417	-0.30232	4.010489	0.045218	1	0	0	0	0	0	1	3	0	0	0
4930546K	2.875427	-0.30205	3.847293	0.049826	1	0	0	0	0	0	0	4	0	0	0
4930481A	2.855164	1.114048	9.093042	0.002566	0.533576	2	1	0	0	0	7	0	12	1	3
Gm5784	2.850115	0.042292	13.70961	0.000213	0.245312	2	0	0	0	3	8	7	6	5	9
Gm128	2.849888	0.853973	8.187588	0.004218	0.651242	1	0	0	0	0	1	2	3	4	0
2610027K	2.849127	-0.19194	4.611376	0.031761	1	1	0	2	0	0	3	6	0	1	10
Myo5c	2.805741	1.140526	8.914789	0.002829	0.552875	1	0	0	0	1	0	2	0	13	1
Vtcn1	2.800325	0.187578	5.6427	0.017528	1	0	0	1	0	0	0	0	0	6	3
Clec4n	2.789661	-0.62102	4.346262	0.03709	1	0	0	0	1	0	2	0	4	2	1
Ucma	2.77372	-0.50928	5.403428	0.020097	1	0	0	2	0	0	2	2	4	2	4

Ccl27a	2.753491	3.008178	17.41918	3.00E-05	0.120527	4	3	2	1	14	7	2	7	93	38
4930478L0	2.745743	2.028744	7.503134	0.006159	0.759767	1	0	0	0	0	0	0	4	2	3
Grhl2	2.731065	5.435743	8.599805	0.003362	0.584254	0	0	1	0	0	0	4	0	4	1
Lrrc8e	2.718593	2.866002	7.866712	0.005035	0.713191	0	1	0	0	0	3	1	5	0	0
LOC10816	2.714627	0.690383	7.34105	0.00674	0.794981	0	0	0	0	1	0	1	5	3	0
Gm26808	2.70671	0.12572	4.805398	0.028371	1	0	0	1	1	0	4	3	7	0	0
Palb2	2.702992	0.726274	11.47743	0.000704	0.348954	0	0	0	4	1	10	11	5	5	0
Zfp459	2.698766	0.526997	8.820132	0.002979	0.564113	0	2	0	0	0	5	3	4	3	0
Pcdhga9	2.671537	-0.60079	3.992092	0.045714	1	0	0	0	0	0	0	0	1	0	2
Styk1	2.656056	0.410703	5.161725	0.02309	1	0	1	0	0	0	0	1	0	8	0
Pcdhga10	2.655413	0.286716	3.891353	0.048535	1	0	0	0	0	2	0	0	4	6	4
Cyp4f41-ps	2.635202	0.392011	4.387321	0.036207	1	0	0	0	0	0	0	0	1	0	2
Tmem139	2.61684	2.492159	4.595443	0.032057	1	0	0	0	0	0	0	0	0	0	3
Gm35596	2.615006	0.342563	4.396738	0.036008	1	0	0	0	0	0	0	0	2	0	1
LOC73899	2.600542	2.605556	4.532926	0.033249	1	0	0	0	0	0	0	0	3	0	0
Acsm1	2.600191	5.719637	4.612671	0.031737	1	0	0	0	0	0	1	0	0	2	0
Gm13782	2.598267	0.310223	4.129155	0.04215	1	0	0	0	0	0	0	0	3	0	0
LOC10816	2.598012	1.618181	4.518762	0.033525	1	0	0	0	0	0	0	0	0	3	0
Gm40235	2.597715	-0.00879	4.340624	0.037213	1	0	0	0	0	0	0	0	2	1	0
Gm39761	2.597486	0.103551	5.314975	0.021143	1	0	0	3	0	0	4	0	1	8	4
Pym1	2.593334	0.158019	4.567655	0.032581	1	1	0	0	0	0	1	1	2	1	3
Cldn8	2.592697	0.32872	3.851407	0.049704	1	0	0	0	0	0	0	0	2	1	0
Sult6b1	2.592622	-0.082	4.117381	0.042445	1	0	0	0	0	0	3	0	0	0	0
Gm35066	2.592034	0.408004	4.188694	0.040694	1	0	0	0	0	0	1	0	0	2	0
Gm15738	2.59145	-0.58782	4.200386	0.040415	1	0	0	0	0	0	0	0	0	3	0
Gm16041	2.588004	0.371261	4.106471	0.042719	1	0	0	0	0	0	0	1	2	0	0
A330035P1	2.586096	-0.2222	3.908583	0.04804	1	0	0	0	0	0	0	0	0	3	0
LOC10263	2.581882	1.368626	5.900972	0.015133	1	0	0	0	1	0	4	0	4	0	0
Gm12414	2.580832	-0.76012	4.006568	0.045323	1	0	0	0	0	0	1	1	0	1	0
LOC10524	2.576811	0.357553	4.24696	0.039321	1	0	0	0	0	0	0	3	0	0	0
Cep112it	2.576746	-0.11727	6.158449	0.013079	1	0	0	0	0	1	4	0	0	2	2
Gm33834	2.57516	-0.04498	6.278759	0.012219	0.9915	1	0	0	0	2	4	1	4	8	2
Cldn7	2.569738	7.348937	7.197278	0.007301	0.826025	0	0	0	0	1	0	0	0	8	0
Nek5	2.56848	2.407881	6.84606	0.008884	0.913142	0	0	1	0	0	0	2	0	6	0
Sox1	2.565599	-0.35399	9.105573	0.002548	0.533013	1	0	3	0	0	4	4	6	8	1
Cdh3	2.560964	1.349162	11.42342	0.000725	0.348954	0	2	0	0	2	0	8	6	7	3
Epcam	2.554887	5.558254	13.66183	0.000219	0.245312	3	0	0	0	0	1	0	0	17	0
Gm6525	2.548745	1.457579	6.135686	0.013248	1	0	1	0	0	0	4	0	2	2	0
LOC10816	2.542707	1.737654	13.6794	0.000217	0.245312	3	0	1	0	0	9	7	0	4	3
Npy	2.538445	6.939852	19.4031	1.06E-05	0.063459	2	1	0	0	2	0	17	9	2	0
Gm31619	2.515215	-0.22137	6.762379	0.00931	0.927798	0	0	1	1	0	3	0	3	3	3
Gm34868	2.503648	0.49131	4.623795	0.031531	1	0	1	0	0	0	1	5	0	1	1
Slfn9	2.48754	2.891968	16.0592	6.14E-05	0.152284	3	0	0	3	0	9	6	9	0	7
1700015F1	2.483633	-0.49188	4.215627	0.040053	1	0	0	1	0	0	1	0	2	1	3
Fam92b	2.473427	-0.55643	4.462649	0.034644	1	0	2	1	0	0	7	6	2	1	2
Slc44a5	2.472816	-0.23726	4.946619	0.026142	1	0	0	1	1	1	3	7	0	6	1
Atp6v0a4	2.467865	-0.50789	3.911047	0.04797	1	0	0	0	1	0	3	0	0	2	2
Gm38978	2.456496	1.44456	7.628906	0.005744	0.735366	1	1	0	0	0	0	0	0	8	4
Gm33937	2.427567	-0.72125	4.276008	0.038654	1	0	0	1	0	0	2	0	2	2	1
Fcer2a	2.408679	0.590991	7.702996	0.005513	0.725022	1	0	2	0	0	3	3	5	3	2
Pla2g4c	2.402183	0.464463	6.78009	0.009218	0.924097	1	0	2	0	4	11	9	6	9	1
Ankdd1a	2.402147	1.372654	5.616344	0.017794	1	0	0	1	0	0	1	0	2	3	1
Gm15880	2.39976	-0.39142	4.610721	0.031773	1	0	0	0	0	1	1	0	0	4	2
Sox21	2.395448	4.707051	5.960863	0.014627	1	0	0	0	1	0	0	1	0	5	1
Chmp4c	2.394273	5.455672	5.986219	0.014418	1	0	0	1	0	0	2	3	0	1	1
Kcnk7	2.384526	1.792408	5.555548	0.018422	1	0	0	0	0	1	5	0	0	2	0
Ccdc89	2.355584	0.311076	6.323951	0.011912	0.9915	0	0	2	0	2	0	11	0	5	4
1700101I1	2.345154	-0.29604	7.449957	0.006344	0.767853	1	0	2	0	1	1	3	4	4	7
Krt13	2.339584	9.169037	7.92832	0.004867	0.698042	0	0	0	0	2	0	0	0	7	4
Krtcap3	2.338451	3.050796	15.382	8.78E-05	0.164152	0	1	1	0	3	5	3	7	5	4
Fam160a1	2.327964	3.890668	14.57624	0.000135	0.195	1	1	2	0	1	4	6	9	5	0
Ildr1	2.326745	5.066649	10.20807	0.001398	0.406676	0	0	3	0	0	0	5	2	0	8
Gm33454	2.326666	1.598816	6.927968	0.008486	0.893702	2	0	0	0	0	0	4	0	4	3
P3h2	2.298936	1.869445	6.420177	0.011283	0.980859	0	2	0	0	0	1	0	8	2	0
Itgb6	2.289643	2.82925	9.274211	0.002324	0.506723	0	0	0	0	3	4	8	1	0	2
Oacyl	2.282125	0.462377	6.296393	0.012098	0.9915	0	5	0	0	0	8	9	0	1	7
LOC10524	2.274144	-0.21743	4.245738	0.039349	1	0	0	0	0	2	0	7	2	0	2
Ms4a4d	2.270987	-0.06887	4.241018	0.039458	1	0	2	3	0	0	3	11	0	4	5

Rph3al	2.266894	0.587413	5.207622	0.022488	1	0	0	0	2	0	0	2	2	3	3
Gm39667	2.231301	0.057726	4.492621	0.034041	1	0	1	0	0	1	0	6	2	3	0
Gm10560	2.226149	-0.12146	4.232758	0.039651	1	4	0	3	0	0	4	3	8	5	9
Gm30569	2.215805	0.873211	5.385357	0.020306	1	0	0	0	0	4	0	0	8	4	6
Ntf5	2.210054	1.304247	5.691753	0.017045	1	0	0	3	0	0	0	7	0	7	0
Slc2a10	2.206727	1.451906	6.867967	0.008775	0.912443	0	0	2	1	1	3	8	7	0	0
Liph	2.202754	6.060459	7.031622	0.008008	0.858587	2	0	0	0	0	0	7	0	1	2
Ttc36	2.195276	4.198049	4.736364	0.029531	1	0	0	0	1	0	0	2	0	1	3
Wnt7b	2.191198	6.168407	4.774405	0.028886	1	0	1	0	0	0	0	1	2	0	3
Tpbg	2.189887	1.578915	11.59497	0.000661	0.348951	2	1	3	0	2	6	9	0	8	10
1700123J1	2.185657	-0.33876	4.032978	0.044619	1	2	0	0	1	0	9	1	2	1	1
Aurkb	2.175459	1.354228	4.009445	0.045246	1	0	0	0	1	0	0	5	0	1	0
Gm15423	2.154723	0.565566	4.81141	0.028272	1	2	1	0	0	1	6	1	0	7	4
Fermt3	2.081425	2.706777	10.43267	0.001238	0.403928	2	0	0	0	4	5	9	5	0	5
Gm31594	2.076804	-0.13638	4.837224	0.027852	1	2	2	0	2	0	3	0	15	3	3
Sytl1	2.06667	7.389941	5.93812	0.014817	1	0	0	0	0	2	0	1	0	1	7
Ltf	2.063511	2.145187	7.662678	0.005637	0.734833	0	1	3	1	0	4	11	0	5	0
1500002F1	2.04926	0.405233	5.233868	0.022151	1	4	4	0	1	2	2	8	5	9	18
LOC10816{	1.997858	-0.06708	5.751731	0.016472	1	0	4	0	2	1	8	4	5	7	3
Gm10910	1.991955	0.528748	4.646936	0.031109	1	0	1	0	0	4	1	4	10	3	2
LOC10816{	1.981538	0.745508	4.355052	0.036899	1	0	0	0	4	0	0	1	1	1	10
Gm13139	1.969504	2.433597	9.167191	0.002464	0.526744	2	12	1	0	5	11	14	36	11	6
LOC10524:	1.951366	0.173686	5.012092	0.025171	1	0	2	0	2	1	7	4	3	5	0
Gm38639	1.949805	1.752609	12.88696	0.000331	0.274974	8	1	1	6	2	7	17	22	9	7
Polq	1.948171	0.940322	5.010283	0.025197	1	1	0	0	0	7	2	12	6	10	1
Gm26666	1.906759	-0.19399	4.944536	0.026173	1	4	1	0	1	1	1	5	3	5	10
Il20rb	1.904379	5.402057	4.832886	0.027922	1	0	0	0	0	2	0	0	4	1	3
Fam83g	1.896957	6.351785	4.718522	0.029839	1	1	0	0	0	1	3	5	0	0	0
E130012A:	1.890023	5.136739	13.17451	0.000284	0.267345	3	1	0	0	4	1	8	9	4	5
Igfbpl1	1.882489	-0.04025	5.024334	0.024994	1	0	1	4	1	2	4	13	5	3	2
2610305D:	1.881209	0.998523	4.480184	0.03429	1	2	6	0	13	0	13	38	3	10	5
Lpin3	1.871216	3.023096	5.769859	0.016303	1	0	1	2	0	0	2	7	0	2	0
Nhlrc3	1.847769	0.835202	4.421084	0.035497	1	3	0	0	0	0	1	0	2	7	1
Gm33223	1.847069	2.364575	12.83411	0.00034	0.274974	1	0	4	9	0	6	13	16	4	4
Pantr1	1.846479	2.299585	5.110522	0.023781	1	0	3	0	0	0	3	0	4	4	0
Wdfy4	1.839825	0.18862	6.806447	0.009083	0.923346	3	3	0	2	1	3	15	7	4	2
AW11201(	1.836981	0.282153	4.627549	0.031462	1	5	1	6	3	2	10	8	17	18	1
Prr15l	1.819018	1.718841	4.976927	0.025688	1	0	2	0	0	2	0	0	0	12	2
Hyal1	1.796911	3.714712	14.74932	0.000123	0.195	5	6	3	4	3	4	6	11	20	22
Gm5917	1.792484	-0.15573	3.964288	0.046475	1	0	1	0	4	1	0	3	8	1	6
Gm8210	1.787868	0.858376	4.678119	0.030549	1	0	0	2	1	1	0	4	6	1	2
LOC10816:	1.780373	0.895589	5.306358	0.021248	1	2	0	0	4	0	7	4	2	6	0
Pigh	1.772195	0.785121	5.631192	0.017644	1	0	0	2	7	0	11	1	10	4	0
Ctf1	1.757648	1.632849	4.455987	0.034779	1	2	1	1	0	2	8	4	0	8	0
Gm40074	1.753892	1.55541	8.534762	0.003484	0.594076	5	2	15	5	12	42	12	15	23	19
Gm40869	1.7538	1.261022	4.437476	0.035158	1	1	1	0	0	2	3	0	6	0	4
LOC10263	1.744984	0.037234	4.170207	0.041141	1	0	4	4	0	1	3	16	3	5	2
Gm29997	1.7335	2.515636	9.313016	0.002275	0.502051	0	1	3	5	0	3	4	6	5	8
Batf2	1.727828	1.811007	4.209946	0.040188	1	0	0	2	0	1	0	9	0	1	0
Gm31399	1.726734	-0.19526	4.71863	0.029838	1	2	0	0	0	2	2	3	5	2	1
Slc37a2	1.698243	0.961264	4.493131	0.034031	1	0	5	1	1	1	8	4	3	7	3
Lrrc56	1.696857	0.729995	7.081351	0.007789	0.854323	7	4	1	1	1	3	14	12	3	10
LOC10816:	1.693974	1.455701	4.813007	0.028246	1	5	0	0	3	0	2	0	5	17	0
Myb	1.649466	2.1592	4.404996	0.035834	1	0	0	2	0	2	0	5	0	5	2
Gm40671	1.637725	2.290739	7.134991	0.007559	0.846584	2	0	3	2	2	4	2	1	5	12
Gm32856	1.618705	1.605449	9.763704	0.00178	0.444309	4	3	10	2	1	22	9	2	12	8
Dct	1.61657	4.147293	3.889707	0.048583	1	0	1	0	1	2	0	9	1	0	2
2810408A:	1.61123	0.904054	4.214611	0.040077	1	0	0	0	6	2	5	2	5	6	3
Platr14	1.605964	0.44118	6.871268	0.008759	0.912443	1	3	4	2	3	8	16	5	4	3
Zic4	1.585363	1.011842	4.054832	0.044045	1	4	5	2	8	5	20	1	7	9	23
Slc38a5	1.585018	-0.03799	4.161639	0.041349	1	2	0	6	0	3	6	10	3	5	5
2210406O:	1.569141	1.338624	8.171732	0.004255	0.654152	5	10	9	3	3	17	5	7	36	14
Gm16174	1.561967	1.852129	5.480201	0.019233	1	0	0	9	0	3	9	0	8	9	4
Aaed1	1.553936	2.697786	13.50599	0.000238	0.247537	6	6	2	6	2	9	16	10	21	2
2310002F(	1.553355	1.073183	4.995411	0.025415	1	4	2	0	4	2	11	4	1	12	4
Slc35g1	1.541536	1.978038	6.632975	0.010011	0.943727	4	4	0	4	0	9	1	16	6	0
Mfsd7a	1.540224	2.551146	8.913079	0.002831	0.552875	1	8	0	11	6	15	14	27	3	8
2610528A:	1.531752	9.534763	4.281448	0.038531	1	0	0	0	0	4	0	0	0	8	3

Ipp	1.515415	1.963188	10.78711	0.001022	0.386047	5	2	11	5	5	6	26	11	16	10
Fdxacb1	1.503177	2.248325	10.54768	0.001163	0.403928	8	4	11	0	7	4	17	18	17	18
Slurp1	1.473888	9.923438	5.906081	0.015089	1	0	1	1	0	6	0	0	0	13	7
AF357425	1.453611	0.198745	5.054629	0.02456	1	2	2	4	9	0	11	15	7	6	1
LOC10264(	1.448104	1.00211	3.993951	0.045664	1	1	0	0	1	4	1	1	5	4	4
Galnt12	1.447006	3.042206	6.01202	0.014209	1	1	1	0	0	6	0	7	0	10	3
Vrk2	1.444442	2.590029	11.18623	0.000824	0.357189	8	3	6	7	7	16	22	11	15	10
Gm27003	1.440219	1.113818	4.441655	0.035072	1	6	0	1	7	1	7	12	13	3	1
1810044D(	1.43244	0.645088	4.317063	0.037732	1	2	1	6	2	3	3	21	1	7	2
Thap6	1.425841	2.605763	8.68606	0.003207	0.576484	4	6	0	6	4	4	18	19	6	2
Cand2	1.424125	3.343628	17.40823	3.02E-05	0.120527	4	12	30	17	13	30	56	39	27	21
Mirc35hg	1.41472	2.789164	10.6166	0.001121	0.403208	15	4	15	11	12	27	16	45	23	19
Ak4	1.385884	2.425287	9.302314	0.002289	0.502051	9	2	4	2	1	11	12	11	6	2
Krt6b	1.380036	10.92671	6.452401	0.01108	0.977017	0	0	0	0	9	0	0	0	16	5
Ttc22	1.36443	4.254864	3.890769	0.048552	1	2	0	0	1	2	3	6	0	3	0
Pkd2l2	1.350055	0.427449	4.72081	0.0298	1	10	5	4	3	1	5	12	13	14	9
Gm15866	1.337414	1.560613	3.917861	0.047776	1	2	1	1	2	8	12	1	7	1	10
Snord22	1.332353	1.43904	3.874864	0.049014	1	11	0	12	0	6	5	3	31	10	13
Mrpl23	1.307306	0.793407	4.228529	0.03975	1	0	1	5	5	5	4	10	10	6	4
Galk2	1.297067	1.457875	5.040282	0.024764	1	2	2	4	3	6	2	11	2	9	12
LOC10105!	1.293419	3.990287	6.05598	0.013859	1	1	0	0	4	6	3	8	6	5	2
Mia	1.277686	1.941558	5.087907	0.024093	1	8	2	3	7	6	21	12	0	23	1
LOC10816(	1.27409	1.53087	4.015907	0.045073	1	9	1	3	1	5	9	9	3	15	6
Nusap1	1.242871	4.683688	9.514015	0.002039	0.479868	7	6	3	8	1	8	8	10	12	13
Il17rb	1.242707	0.906833	5.004037	0.025288	1	10	1	13	5	11	8	20	30	9	14
LOC10263:	1.235441	0.53817	4.200471	0.040413	1	7	0	0	6	7	9	12	7	8	6
Xndc1	1.228253	2.100022	8.142101	0.004325	0.659293	6	4	7	3	10	9	11	17	9	15
Exoc3l4	1.21892	1.059676	5.825755	0.015793	1	1	8	7	4	7	7	11	12	13	12
4933440N:	1.214504	1.210447	4.21543	0.040058	1	0	5	0	4	5	7	13	3	5	2
Rhpn2	1.204084	2.973708	4.149141	0.041656	1	0	4	0	1	5	2	9	0	5	5
Gpr17	1.203612	0.454463	4.002349	0.045437	1	1	2	8	4	3	6	2	4	11	11
Zfp658	1.184244	1.095255	5.808753	0.015947	1	3	3	8	9	8	13	12	17	16	3
Cd109	1.182752	5.778835	7.010256	0.008104	0.865176	4	2	3	0	6	6	10	11	0	3
Helq	1.181784	1.819251	10.51346	0.001185	0.403928	7	5	5	11	7	13	11	12	17	15
Lrrc1	1.17653	4.273964	15.99548	6.35E-05	0.152284	6	5	12	13	8	9	25	8	26	17
Mns1	1.157014	2.029436	5.169837	0.022982	1	4	2	11	13	10	30	8	14	11	11
Tor2a	1.155548	1.824659	4.754786	0.029217	1	0	0	4	5	5	3	9	3	8	4
Rnls	1.151781	0.990141	3.909223	0.048022	1	3	2	7	3	2	3	10	7	3	9
Gm35290	1.148411	2.144985	6.625757	0.010051	0.943727	12	6	7	3	4	11	19	8	13	12
Naip1	1.148351	4.308702	6.008439	0.014238	1	3	3	4	7	0	8	12	8	5	0
Arhgef19	1.126581	4.300836	10.4852	0.001203	0.403928	8	14	3	26	5	24	38	22	16	7
Syce2	1.121443	2.176993	4.764769	0.029048	1	2	3	8	8	0	5	13	0	10	10
LOC10816(	1.118598	1.318273	7.578804	0.005906	0.735366	6	7	7	10	16	25	12	22	20	9
Nrp	1.113834	2.378702	4.793998	0.028559	1	2	0	3	7	3	0	4	13	9	2
LOC10524(	1.107109	2.919783	5.105079	0.023856	1	6	2	0	8	0	6	4	5	11	4
LOC10263(	1.091381	1.720153	3.861267	0.049413	1	5	10	10	0	4	2	11	25	11	7
Asprv1	1.07184	11.0908	6.095541	0.013552	1	0	0	1	5	11	1	0	2	19	9
Kif20b	1.069419	4.795518	14.2749	0.000158	0.217041	25	8	11	13	5	36	24	27	21	6
Gm16938	1.068567	4.383868	6.244615	0.012457	0.9915	5	10	2	0	10	10	14	14	8	6
Mrap	1.068092	0.847215	4.837669	0.027845	1	7	7	10	3	4	15	7	11	9	14
Morn1	1.065262	2.227368	7.24189	0.007122	0.816013	5	19	26	9	15	21	32	35	16	29
Myrf	1.06168	1.763573	6.738441	0.009436	0.931074	7	12	20	21	24	27	45	34	29	17
D930048N	1.060358	3.062113	3.910307	0.047991	1	0	0	5	1	5	0	3	5	5	7
Ms4a6c	1.056012	1.328803	3.919408	0.047732	1	7	16	1	7	15	18	20	25	20	8
Gm5595	1.055175	1.541782	4.515856	0.033582	1	8	4	7	8	6	15	6	10	12	15
Traf5	1.054545	4.368438	4.218658	0.039982	1	4	1	2	3	2	14	3	4	1	0
Gm26868	1.053972	0.429711	3.854682	0.049607	1	8	5	6	6	2	5	19	12	10	4
Man1a	1.053737	2.225622	5.294431	0.021394	1	6	9	17	12	20	18	32	13	38	15
Zwilch	1.046151	2.134845	3.948684	0.046908	1	4	8	21	2	3	17	16	23	3	8
Fpgs	1.042061	1.928192	7.592952	0.00586	0.735366	4	6	14	8	13	20	16	21	14	9
Esrp1	1.023005	7.137429	5.970212	0.01455	1	5	3	3	0	7	6	0	4	20	2
Bbs12	1.020751	1.750827	6.618672	0.010092	0.943727	14	4	7	8	12	15	15	26	19	6
Ankrd7	1.018541	2.199943	4.738197	0.0295	1	6	7	2	6	10	7	23	6	13	8
Gpr68	1.015613	2.523267	6.651627	0.009907	0.943727	11	5	47	5	12	32	33	29	11	27
Ammecr1	1.01483	3.029431	7.445264	0.00636	0.767853	7	7	17	3	17	6	31	24	13	16
Cpm	1.004152	6.715549	15.74284	7.26E-05	0.159588	19	5	15	19	10	32	20	13	40	12
Exosc8	0.993584	2.636769	6.331226	0.011863	0.9915	11	9	15	7	11	5	13	16	38	19
Apex2	0.988669	2.660835	6.268305	0.012292	0.9915	3	4	7	9	16	7	12	16	14	17

Tpx2	0.981962	4.449456	10.43881	0.001234	0.403928	18	16	12	4	9	28	19	20	19	17
4921531C2	0.976509	2.948553	5.874492	0.015362	1	17	10	18	1	4	15	21	21	24	7
Gdpd2	0.975087	4.412126	17.03261	3.67E-05	0.132188	25	16	23	24	17	27	52	31	48	21
Sap30	0.969425	1.775132	3.969785	0.046324	1	3	4	7	3	6	3	10	7	10	9
Bud31	0.969408	2.539531	5.110419	0.023783	1	16	8	12	7	10	11	8	22	24	24
Dock8	0.950242	3.064174	6.324043	0.011911	0.9915	10	25	9	3	19	7	34	33	29	16
Birc5	0.949991	4.773387	10.18874	0.001413	0.406676	10	11	21	23	12	35	33	31	16	12
Bag2	0.946096	4.134024	10.10346	0.00148	0.412723	12	15	24	14	17	30	33	19	44	12
Gm35558	0.944608	0.969824	4.158573	0.041424	1	4	9	11	15	3	17	16	6	17	12
Rftn1	0.938247	1.446897	5.601	0.01795	1	9	6	19	6	11	15	19	21	17	12
Prob1	0.935756	1.769483	4.690173	0.030336	1	5	11	16	23	17	34	32	21	13	17
Hpgds	0.930485	6.927807	9.37266	0.002202	0.498337	10	12	10	2	11	19	15	15	15	11
Mrvi1	0.90423	1.388283	4.734936	0.029556	1	18	7	9	10	13	13	19	11	35	16
Zfp119a	0.896403	1.75185	5.213769	0.022409	1	1	1	7	11	9	7	12	9	12	6
Zfp53	0.892402	3.340033	10.1599	0.001435	0.409808	18	19	6	14	13	33	27	15	25	15
Gm34583	0.889803	1.344285	6.576954	0.010331	0.951656	16	8	13	11	11	15	36	16	17	12
Plekhg2	0.874554	3.516052	10.90007	0.000962	0.380847	24	14	38	52	19	70	58	27	40	30
Phyhip	0.870295	6.276858	10.34815	0.001296	0.406402	18	14	5	6	18	23	17	28	16	14
Zfp280c	0.868691	3.257178	10.47969	0.001207	0.403928	29	15	38	20	34	30	42	42	65	35
Gal	0.858908	2.935301	7.986112	0.004714	0.695008	43	24	111	48	47	52	139	97	77	51
Gm10051	0.858523	3.191789	5.54487	0.018535	1	9	2	16	16	9	13	15	12	28	11
Sema4g	0.857621	2.67662	5.756153	0.016431	1	38	9	35	64	44	33	61	67	85	42
Col7a1	0.853771	3.962998	3.96045	0.046581	1	5	2	6	2	8	0	5	10	13	8
Ccdc61	0.841471	3.044189	6.414293	0.011321	0.980859	24	13	19	20	11	30	37	18	29	21
Cenpf	0.840453	6.278671	4.676824	0.030572	1	2	9	13	4	3	23	7	11	3	4
Mtm1	0.839866	2.042098	3.856986	0.049539	1	11	13	11	10	18	15	25	15	32	14
Syk	0.837393	3.809797	3.957843	0.046653	1	4	1	6	2	10	6	20	4	1	5
Mybl1	0.833527	2.671057	6.30164	0.012063	0.9915	36	12	19	39	32	36	53	45	33	43
Pptc7	0.829422	2.179206	5.691545	0.017047	1	6	10	20	8	5	8	15	19	19	13
Zfp395	0.804709	3.005912	5.033005	0.024869	1	25	16	6	9	9	24	16	13	37	13
5930430L0	0.794347	3.171385	4.889412	0.027022	1	11	11	34	4	20	22	34	24	23	17
Arhgef37	0.792966	4.515096	8.591185	0.003378	0.584254	20	15	8	20	15	25	21	16	32	23
BC064078	0.789788	5.247732	4.03942	0.044449	1	1	1	9	10	2	9	0	12	9	4
Rfc5	0.783918	3.8536	7.782057	0.005277	0.725022	18	10	22	14	11	30	32	14	18	17
Nsun3	0.782458	2.623054	3.903883	0.048175	1	9	7	12	11	10	18	21	19	6	9
Casp8	0.778966	5.179959	5.235284	0.022133	1	12	8	15	0	12	13	7	17	18	15
Pop4	0.777279	4.831554	6.855288	0.008838	0.913142	68	106	40	57	66	77	84	87	176	101
Pxdn	0.775641	2.299822	4.303789	0.038028	1	28	11	15	49	28	57	39	30	41	24
Il17rc	0.770952	5.271153	7.605404	0.005819	0.735366	12	23	11	33	16	20	33	18	17	48
Hook1	0.765451	4.352157	4.453278	0.034834	1	15	1	30	4	10	17	11	11	29	17
Hspa1a	0.759859	6.964555	13.10185	0.000295	0.267345	35	28	30	30	25	60	56	44	29	29
Zfp408	0.758981	2.93589	4.898698	0.026877	1	7	7	19	16	12	30	11	15	11	19
Myo7a	0.757696	2.75947	6.161716	0.013054	1	20	20	26	35	20	37	25	30	49	32
Tango6	0.743972	2.673748	5.095742	0.023985	1	24	13	37	33	13	36	51	35	30	18
Fgd3	0.728252	4.839148	4.297486	0.038169	1	11	2	11	8	2	14	7	13	5	9
Ccdc51	0.723452	4.577933	7.244159	0.007113	0.816013	3	16	11	19	11	24	13	23	15	10
Agbl4	0.720749	1.731179	6.320398	0.011936	0.9915	22	18	23	23	8	30	23	27	27	25
Gtf2h3	0.719139	3.241003	6.206306	0.01273	1	12	11	32	16	15	29	21	25	29	16
Snx24	0.715787	3.21744	6.272919	0.01226	0.9915	10	36	42	49	29	35	45	61	44	44
Nbeal2	0.704168	6.265568	3.888776	0.04861	1	7	2	4	10	7	9	1	5	22	5
Cfap69	0.70151	2.272465	4.568938	0.032557	1	23	16	11	13	15	26	20	16	23	26
Spon2	0.700277	6.339697	4.604663	0.031885	1	6	5	16	23	6	13	24	8	19	12
Zgrf1	0.691654	2.538466	4.218814	0.039978	1	20	8	10	20	23	25	15	21	25	26
Ttll4	0.689476	3.096306	4.807567	0.028335	1	14	15	10	12	17	15	14	21	29	17
Ankrd33b	0.689243	1.971216	3.989964	0.045772	1	13	26	22	20	20	29	28	15	37	32
Herc6	0.684789	3.982428	4.709073	0.030004	1	15	19	22	27	20	44	28	15	25	29
Pias3	0.672559	3.286609	3.932723	0.047355	1	17	17	22	31	17	42	37	16	33	15
Gm2694	0.668912	4.781649	5.720287	0.01677	1	11	7	22	22	2	25	25	14	13	9
Gk5	0.667053	3.585529	3.955831	0.046709	1	21	10	9	21	5	17	16	7	27	22
Spsb3	0.648027	3.366184	5.105302	0.023853	1	36	17	60	52	41	32	63	65	67	43
Irf6	0.644096	7.435745	6.605153	0.010168	0.943727	13	7	21	12	21	21	34	13	10	21
Ckap2l	0.642156	5.590159	3.954327	0.046751	1	10	8	12	9	4	21	15	11	8	3
Get4	0.641686	2.822491	4.399933	0.03594	1	14	22	35	41	29	26	59	49	38	17
Epb41l4a	0.630282	5.502612	9.116308	0.002533	0.532993	45	27	42	40	23	41	45	46	67	36
Xkr8	0.628735	2.224039	4.209562	0.040197	1	26	17	31	21	23	31	45	39	19	24
Nsun6	0.620387	3.633674	6.012206	0.014207	1	30	28	23	24	32	25	33	38	52	36
Polr3a	0.616015	3.521212	5.144787	0.023316	1	35	28	47	57	40	35	73	43	58	58
Fignl2	0.602837	3.778694	6.339796	0.011806	0.9915	22	29	42	35	50	63	52	54	25	39

Efhd2	0.593804	4.999869	8.460317	0.00363	0.610211	17	39	38	42	25	32	55	54	40	28
Zkscan8	0.593112	4.326801	7.059984	0.007882	0.858587	42	52	66	110	50	67	99	76	92	71
Cxadr	0.574645	6.319607	5.781452	0.016196	1	12	23	34	10	12	17	15	33	31	20
Ankrd52	0.571507	3.198218	4.63995	0.031236	1	38	35	32	57	48	49	44	52	66	56
Med23	0.569858	4.118849	5.831911	0.015738	1	54	28	54	22	24	55	74	40	33	34
Adnp2	0.568066	3.392278	4.466281	0.03457	1	9	18	45	38	21	30	40	24	38	29
Cbr2	0.56701	9.26506	4.996433	0.0254	1	20	5	22	26	32	22	36	28	27	20
Dapl1	0.566872	11.011	7.603211	0.005826	0.735366	25	14	30	42	15	32	34	19	35	37
Vasn	0.566348	4.711099	5.034135	0.024852	1	6	29	26	41	28	37	38	10	34	43
Pfkfb4	0.560552	4.175748	4.949483	0.026098	1	37	20	58	24	36	45	42	31	56	45
Spaca6	0.560028	4.022863	4.180143	0.0409	1	35	29	39	39	20	33	46	43	57	27
Hspa1b	0.552861	7.815268	5.278581	0.021589	1	29	25	41	33	41	40	67	34	42	32
Anln	0.551883	4.610143	4.754936	0.029214	1	64	24	39	60	69	73	99	50	68	40
Copg2	0.549689	4.662089	6.24773	0.012435	0.9915	66	30	61	28	43	57	47	61	67	56
Trp53bp1	0.53484	4.317915	5.597612	0.017985	1	100	53	107	58	75	98	92	75	104	116
Dcaf12l1	0.531352	3.620735	6.574856	0.010343	0.951656	69	72	64	100	56	95	91	89	119	59
Fbll1	0.530206	2.48455	7.084134	0.007777	0.854323	34	40	37	43	34	52	54	42	50	37
Zcchc14	0.528393	3.610003	5.333665	0.020917	1	28	37	41	52	32	45	62	45	59	26
Rnf213	0.523153	4.207412	4.115725	0.042486	1	27	21	79	37	58	39	56	48	72	52
Klhdc8b	0.518817	3.928197	6.023415	0.014117	1	91	61	60	93	93	90	103	107	123	76
Cep162	0.51179	4.908204	9.004219	0.002694	0.552121	98	57	116	106	101	146	110	98	129	98
Gcat	0.50816	5.625052	7.631122	0.005737	0.735366	36	31	68	55	37	45	53	53	63	57
Tep1	0.507955	4.743146	7.828141	0.005144	0.722878	90	69	65	44	49	98	69	80	77	72
Stmn1	0.504501	6.276974	4.213239	0.04011	1	351	445	387	835	313	502	577	785	506	428
Cdv3	0.502886	5.753538	8.659901	0.003253	0.579336	65	72	126	106	79	82	94	90	180	91
Zdhhc5	0.499617	6.163521	6.501661	0.010777	0.95735	49	47	83	104	49	55	106	74	76	80
Tmem62	0.494338	3.938339	4.666486	0.030757	1	28	33	92	67	69	58	75	58	71	75
Onecut1	0.490874	3.09934	5.52373	0.01876	1	74	34	53	71	39	64	73	70	72	48
Racgap1	0.485122	5.010656	5.783055	0.016181	1	39	41	38	35	42	39	61	38	65	36
Myof	0.481878	6.733919	3.92733	0.047507	1	11	20	22	8	15	14	21	21	12	23
Ddx28	0.480921	3.811286	4.455519	0.034789	1	54	40	65	79	43	69	60	51	99	53
Sgsm2	0.479207	3.825933	4.923892	0.026488	1	40	37	91	44	40	56	61	70	51	57
Dagla	0.478684	3.433676	4.086463	0.043228	1	48	40	59	67	49	35	64	60	68	80
Mast4	0.473108	7.918613	12.75263	0.000356	0.274974	105	57	127	134	91	99	108	141	141	112
1300002E1	0.471891	4.431776	5.429759	0.019796	1	43	32	57	81	75	56	67	82	91	45
Pdlim5	0.469814	6.942713	11.49092	0.000699	0.348954	92	63	92	109	69	88	121	94	139	64
Gramd3	0.46558	5.493426	4.211074	0.040161	1	15	19	24	23	11	23	28	20	29	9
Nt5dc3	0.465338	4.86018	10.89673	0.000963	0.380847	166	154	274	230	165	249	243	238	246	183
Mcph1	0.462857	4.042949	4.560992	0.032708	1	67	55	124	84	46	79	104	88	85	78
Rbm4b	0.461431	4.591225	4.625074	0.031508	1	75	72	87	87	85	123	75	98	107	80
Sec22b	0.459119	4.743508	5.943058	0.014775	1	60	81	80	69	64	87	117	94	82	49
Traf6	0.458663	4.062869	4.867661	0.027365	1	39	31	48	40	41	55	46	47	56	32
Asb7	0.457784	4.398315	5.714479	0.016826	1	58	66	80	55	71	73	94	88	81	60
Kdm3b	0.453046	5.150959	6.070969	0.013742	1	59	55	46	86	68	99	85	48	85	56
Uba6	0.452114	5.509668	7.700057	0.005522	0.725022	123	143	234	206	142	186	208	208	270	120
11-Mar	0.446487	3.389366	4.64934	0.031065	1	71	66	117	104	71	115	121	108	84	70
Agpat2	0.434845	4.651243	4.811595	0.028269	1	18	30	43	34	21	49	21	25	48	25
Rps6ka4	0.432776	4.726613	4.361373	0.036763	1	43	40	88	96	43	78	57	55	70	82
Tbl3	0.428948	4.541156	5.603817	0.017921	1	75	52	97	90	77	114	107	77	88	65
Zfp236	0.427389	4.349056	4.282617	0.038504	1	88	70	71	128	69	89	98	121	85	94
Yif1b	0.427313	4.203713	4.123245	0.042298	1	83	57	128	137	92	175	113	97	106	73
Polr2i	0.423483	4.367937	4.356743	0.036863	1	44	43	88	82	49	82	96	68	60	42
Kantr	0.421331	3.633957	4.379449	0.036375	1	49	43	77	71	57	70	77	60	80	52
Kcnk10	0.421194	3.694793	5.326276	0.021006	1	83	76	96	107	98	123	115	128	91	76
Itgav	0.419314	5.700134	6.193486	0.012822	1	132	83	146	136	144	185	142	118	167	124
Pcdh1	0.419106	6.253054	4.339198	0.037244	1	51	26	29	41	33	48	27	38	55	39
Elf2	0.418948	4.988395	6.297995	0.012087	0.9915	48	51	74	98	62	65	81	84	62	82
Camk4	0.41765	3.770278	5.084784	0.024137	1	70	73	104	112	72	92	110	74	131	83
Zbtb22	0.417103	3.797545	3.849596	0.049758	1	38	38	46	71	37	71	49	47	61	34
Ptgfrn	0.413894	5.861908	5.973163	0.014525	1	83	71	95	102	53	123	90	112	72	64
Gid4	0.412398	5.55091	10.52249	0.001179	0.403928	135	139	205	251	227	199	203	243	230	204
Zbtb6	0.41219	4.474426	5.14789	0.023275	1	51	49	69	54	44	63	50	68	77	47
Rad54l2	0.408756	4.511752	4.407852	0.035774	1	52	67	107	47	86	91	107	82	83	54
Cdyl2	0.40683	3.781238	4.893834	0.026953	1	64	63	82	117	92	105	115	118	87	53
Rps6kb2	0.404956	4.185669	4.602323	0.031929	1	42	54	65	82	63	78	56	86	80	47
Srf	0.402772	5.04668	4.13319	0.04205	1	32	78	89	75	68	92	94	80	76	50
Rab27b	0.400417	6.611819	15.3958	8.72E-05	0.164152	165	167	203	246	175	230	244	224	215	169
Trim25	0.399353	6.367866	6.63747	0.009986	0.943727	57	54	71	53	55	73	88	71	63	37

Trim24	0.398302	5.253521	8.130815	0.004352	0.66061	85	84	151	117	113	128	140	124	123	103
Rnf170	0.397554	4.50371	4.181673	0.040863	1	45	50	90	80	49	47	66	75	74	82
Eps8	0.394736	5.373913	4.052245	0.044113	1	32	19	40	64	46	59	62	28	31	43
Ss18	0.394661	5.096285	5.92813	0.014901	1	50	54	75	61	73	83	60	78	77	56
Reep4	0.391474	6.983142	4.243239	0.039407	1	26	37	51	24	37	37	45	38	35	42
Snhg9	0.382183	5.324278	4.989354	0.025504	1	116	92	194	155	67	163	147	151	143	83
Rasgrp1	0.378717	4.959403	3.864507	0.049318	1	166	155	247	242	181	193	233	166	303	198
Ebna1bp2	0.377332	5.514672	7.669035	0.005618	0.734833	126	102	139	165	146	164	160	132	186	116
Mon1b	0.376313	4.252086	3.866163	0.049269	1	44	48	101	76	54	80	64	82	54	70
Cacna2d3	0.370887	4.677597	4.731695	0.029612	1	146	148	194	234	196	214	224	197	222	162
Sertad4	0.370801	4.798574	3.851255	0.049709	1	62	68	97	85	76	118	56	93	94	68
Inpp5b	0.36355	3.916032	5.194236	0.022662	1	61	50	74	87	52	73	78	69	78	57
Iffo2	0.36298	5.876058	4.445977	0.034984	1	47	54	42	82	77	51	87	64	76	58
Ddn	0.361522	4.336503	4.047526	0.044236	1	116	98	145	246	153	150	150	193	170	151
Wbp1l	0.359341	5.839386	7.719196	0.005464	0.725022	100	81	144	147	96	112	145	135	132	95
Llph	0.357112	5.246468	3.879893	0.048868	1	60	51	63	87	101	91	94	91	81	47
Ctdspl	0.355317	6.364332	6.733025	0.009464	0.931074	105	101	135	150	111	174	124	135	144	85
Dpp8	0.353859	5.822675	5.878003	0.015331	1	216	162	282	294	159	188	261	243	224	269
Fam160a2	0.350927	5.220051	5.319093	0.021093	1	137	68	144	98	107	108	118	139	124	115
Nudt7	0.348032	4.365945	5.248417	0.021967	1	47	56	123	120	68	92	111	89	85	65
Rnf4	0.34716	5.167215	4.787523	0.028667	1	123	86	137	187	105	151	160	167	134	81
Zcchc2	0.345077	4.820974	4.440276	0.035101	1	65	52	122	95	69	76	119	70	86	80
Zdhhc22	0.341435	4.504954	3.950564	0.046856	1	130	165	174	234	162	178	139	212	215	186
Stag1	0.334736	5.314926	4.490716	0.034079	1	114	66	105	141	91	138	109	82	133	93
Gstm1	0.333563	5.473224	5.19665	0.02263	1	119	119	125	191	138	159	151	144	174	121
Ephx1	0.323881	6.072705	6.06027	0.013826	1	107	157	250	254	161	205	196	195	230	154
Otud3	0.320686	4.706536	4.975122	0.025714	1	97	66	138	126	105	111	150	114	104	87
Setd1b	0.320159	6.17278	5.881489	0.015301	1	165	149	225	196	232	179	253	235	229	151
Zfp120	0.320134	4.419133	3.849234	0.049769	1	50	39	67	90	74	60	68	74	72	64
1190002N1	0.319874	4.260906	3.986904	0.045855	1	100	80	143	133	131	142	113	138	132	100
Arid5b	0.318608	7.382408	3.869055	0.049184	1	235	216	255	249	350	255	339	291	245	286
Mcm3ap	0.317931	4.67509	4.24768	0.039304	1	87	84	144	111	96	144	82	110	111	104
Parp4	0.315299	5.897495	5.331621	0.020942	1	107	106	124	155	105	105	150	124	158	101
Snapc5	0.314547	5.204268	4.569774	0.032541	1	125	145	167	236	186	193	158	208	195	157
Pnrc2	0.312358	5.473726	3.950498	0.046857	1	81	86	139	115	91	105	102	115	117	100
Pwwp2a	0.309126	5.842525	4.525344	0.033396	1	102	111	110	121	124	119	117	129	143	103
Uvrag	0.30615	4.7947	4.227239	0.03978	1	80	93	94	135	103	105	109	98	105	115
Osgep	0.303499	4.73399	4.276253	0.038648	1	74	71	81	98	75	75	92	100	95	62
Phf20	0.302238	5.481313	6.526133	0.01063	0.954012	235	230	334	354	276	267	308	312	333	277
Rims3	0.301665	5.382683	6.038745	0.013995	1	245	286	430	488	295	339	365	415	334	347
Lrrc8b	0.300895	6.133931	5.558273	0.018394	1	115	114	184	156	167	182	144	149	167	133
Spns1	0.30085	4.47726	4.019555	0.044976	1	91	86	144	114	110	109	147	106	129	86
Pdpn	0.297424	6.842843	3.990971	0.045745	1	102	203	232	215	146	163	208	216	216	145
Fam219a	0.294791	5.911605	5.055905	0.024542	1	443	352	513	521	399	514	443	441	636	324
Cep104	0.293692	4.79796	4.056396	0.044004	1	166	107	167	153	147	180	159	175	159	112
Zdhhc17	0.291377	5.185676	5.679081	0.017168	1	214	203	299	316	247	244	283	287	281	237
Lin7c	0.286996	6.216944	5.620219	0.017754	1	131	137	231	245	153	230	166	191	210	129
Zfp322a	0.283168	5.330954	5.987834	0.014405	1	151	139	207	239	173	155	217	185	209	173
Snrpd3	0.282364	7.420267	4.956616	0.025991	1	489	442	684	642	454	553	577	567	630	479
Pygo2	0.280663	5.979636	5.189519	0.022724	1	201	153	343	287	206	284	233	297	236	170
Ston2	0.278535	5.731797	5.573724	0.018232	1	144	106	170	191	127	157	145	144	181	133
Syne2	0.278218	7.990027	4.039793	0.044439	1	228	118	270	270	247	224	213	212	268	238
Gabbr2	0.275821	5.824978	4.112014	0.04258	1	462	290	516	403	386	425	408	383	499	412
Kdm5c	0.274649	5.698289	5.740224	0.016581	1	131	91	231	188	118	142	188	142	156	142
Nptx1	0.27269	6.035924	6.193574	0.012822	1	406	389	667	820	471	522	662	544	652	419
Slc4a1ap	0.269384	5.436537	4.109334	0.042647	1	145	136	167	235	165	187	153	159	199	169
Eif6	0.262138	6.942821	5.348699	0.020738	1	272	146	350	265	205	255	286	301	254	170
Hif1a	0.257859	6.772225	4.682006	0.03048	1	220	194	301	279	213	209	309	219	286	211
Tmem178b	0.250889	5.064478	4.236688	0.039559	1	218	165	281	311	225	225	237	237	282	225
Jade2	0.249004	5.15291	4.148825	0.041663	1	143	107	167	150	116	164	136	133	157	106
Mcmbp	0.245098	5.574388	3.851397	0.049705	1	113	75	131	112	84	116	120	104	108	75
Sf3a1	0.24212	5.722795	4.58906	0.032177	1	163	165	215	210	141	206	166	191	176	163
Man2a2	0.238785	5.454382	3.937799	0.047213	1	217	219	266	305	231	257	260	256	282	201
Plaa	0.237969	5.34894	4.949507	0.026098	1	216	195	256	241	233	269	232	234	237	189
3830406C1	0.230311	6.17337	3.876411	0.048969	1	258	265	327	335	285	231	375	304	334	246
Pank3	0.222806	7.127521	5.007044	0.025244	1	392	439	688	674	484	500	567	596	590	403
Uso1	0.212678	6.532595	4.66641	0.030758	1	305	219	456	366	318	356	334	296	364	284
Myh9	0.202737	8.412473	4.247227	0.039314	1	675	514	690	669	564	591	629	601	749	524

Lgals8	0.19999	6.540515	4.118967	0.042405	1	396	412	671	613	462	475	608	511	473	425
**Downregulated genes**															
Stk31	-5.07878	-0.5391	8.782283	0.003042	0.571673	0	0	12	10	4	0	0	0	0	0
Gm5432	-5.00477	-0.27179	11.01131	0.000906	0.374472	5	4	11	2	0	0	0	0	0	0
Snx22	-5.00141	-0.33204	11.58094	0.000666	0.348951	10	1	0	7	3	0	0	0	0	0
Gm13056	-4.9695	1.667378	20.10062	7.35E-06	0.063459	0	5	11	0	5	0	0	0	0	0
Fer1l6	-4.76279	-0.52716	8.294291	0.003977	0.63308	11	0	2	5	0	0	0	0	0	0
Gm31989	-4.75588	0.232037	12.53246	0.0004	0.275262	7	0	6	6	0	0	0	0	0	0
LOC10816	-4.69473	-0.53603	6.270346	0.012278	0.9915	3	0	0	17	0	0	0	0	0	0
Gm33947	-4.67659	-0.12622	8.776741	0.003051	0.571673	0	11	4	0	0	0	0	0	0	0
Gm16299	-4.67504	-0.13527	8.905545	0.002843	0.552875	0	0	7	4	7	0	0	0	0	0
Gm20383	-4.67165	4.071774	20.08064	7.42E-06	0.063459	0	0	3	4	10	0	0	0	0	0
Stk32b	-4.63876	-0.6756	7.029265	0.008019	0.858587	2	0	0	8	7	0	0	0	0	0
Rnf43	-4.62526	2.47538	18.34187	1.85E-05	0.094877	0	0	0	13	4	0	0	0	0	0
Gm16068	-4.61174	0.738343	12.17384	0.000485	0.28583	0	5	0	12	0	0	0	0	0	0
Tph1	-4.5823	-0.56429	5.150252	0.023243	1	0	0	15	0	3	0	0	0	0	0
Krt79	-4.57058	-0.37572	12.6486	0.000376	0.274974	3	0	9	4	1	0	0	0	0	0
Mir98	-4.54898	0.620351	11.4238	0.000725	0.348954	5	2	5	0	3	0	0	0	0	0
Gm35315	-4.49888	-0.05644	12.63065	0.000379	0.274974	1	4	4	4	2	0	0	0	0	0
Hsd17b1	-4.46206	-0.39069	5.310125	0.021202	1	0	5	5	5	0	0	0	0	0	0
Gm36090	-4.46051	-0.71999	5.308743	0.021219	1	4	8	0	0	0	0	0	0	0	0
Tgm1	-4.439	1.948849	16.33111	5.32E-05	0.152284	0	0	10	5	0	0	0	0	0	0
Gm39937	-4.42291	-0.37173	7.839435	0.005112	0.721193	8	0	1	0	4	0	0	0	0	0
Aspdh	-4.42193	-0.60711	6.185884	0.012877	1	7	1	6	0	0	0	0	0	0	0
Gm11413	-4.41026	-0.69056	8.602851	0.003356	0.584254	7	1	1	5	0	0	0	0	0	0
0610009E	-4.38261	-0.38831	10.42857	0.001241	0.403928	0	3	3	5	3	0	0	0	0	0
Ccdc108	-4.37582	-0.7415	4.263352	0.038943	1	10	0	0	3	0	0	0	0	0	0
Gm36188	-4.37235	-0.48281	7.04122	0.007965	0.858587	0	3	0	8	3	0	0	0	0	0
Stox1	-4.36565	-0.51007	8.417627	0.003716	0.610501	3	7	2	0	0	0	0	0	0	0
Gm40768	-4.35873	-0.43687	6.939756	0.00843	0.892	0	2	0	0	10	0	0	0	0	0
Tnn	-4.34475	-0.44229	5.258641	0.021838	1	0	2	10	3	0	0	0	0	0	0
Gm36673	-4.33267	-0.44864	5.045263	0.024693	1	0	0	8	8	0	0	0	0	0	0
Gm21283	-4.29989	-0.75875	6.488528	0.010857	0.962073	3	5	4	0	0	0	0	0	0	0
Gm15420	-4.29199	-0.2586	7.731054	0.005428	0.725022	0	3	0	11	0	0	0	0	0	0
LOC10263	-4.28947	-0.63742	9.400487	0.002169	0.493936	4	2	0	2	4	0	0	0	0	0
A1cf	-4.28098	-0.64433	5.4884	0.019143	1	1	0	7	7	0	0	0	0	0	0
Gm11713	-4.25363	0.283078	9.943164	0.001614	0.427077	5	0	8	0	0	0	0	0	0	0
Mir6935	-4.24956	-0.73094	4.274138	0.038697	1	0	0	11	4	0	0	0	0	0	0
Gm31672	-4.24863	-0.56222	5.792699	0.016093	1	6	0	0	7	0	0	0	0	0	0
Tsx	-4.24455	-0.65896	6.426528	0.011243	0.980859	0	2	1	7	3	0	0	0	0	0
Fgr	-4.23818	-0.61008	6.712534	0.009574	0.931074	0	1	4	4	4	0	0	0	0	0
9030624G	-4.22438	-0.73934	5.101471	0.023906	1	5	0	0	0	6	0	0	0	0	0
Gm30635	-4.21222	2.281973	12.7077	0.000364	0.274974	0	3	5	0	4	0	0	0	0	0
Hist1h4n	-4.19461	0.131978	9.850549	0.001698	0.433195	0	0	4	6	3	0	0	0	0	0
Gm40255	-4.18572	-0.60673	10.3781	0.001275	0.406402	3	1	5	1	2	0	0	0	0	0
Klk10	-4.16562	2.99083	13.47727	0.000241	0.247537	0	0	9	0	3	0	0	0	0	0
Tmem239	-4.16426	-0.71263	5.365035	0.020544	1	0	0	9	5	0	0	0	0	0	0
Tmem184	-4.16385	4.69872	14.07326	0.000176	0.218121	0	0	0	12	0	0	0	0	0	0
Oc90	-4.13742	-0.77467	5.08886	0.02408	1	0	5	0	1	4	0	0	0	0	0
Gm31284	-4.10008	-0.72429	6.132267	0.013274	1	5	0	4	0	2	0	0	0	0	0
Rmi2	-4.09544	1.177399	11.95517	0.000545	0.316218	0	0	4	8	0	0	0	0	0	0
Gm31633	-4.08802	0.466103	9.807353	0.001738	0.440365	7	0	0	4	0	0	0	0	0	0
Nr0b2	-4.08619	-0.42303	7.264714	0.007032	0.813479	3	0	0	4	4	0	0	0	0	0
Gm30075	-4.0851	-0.42814	7.174713	0.007394	0.833857	0	2	10	0	0	0	0	0	0	0
Gm36906	-4.08359	0.242228	10.22112	0.001388	0.406676	0	5	0	6	0	0	0	0	0	0
Ugt1a5	-4.07217	0.329609	9.122077	0.002525	0.532993	0	0	6	4	2	0	0	0	0	0
2200002J2	-4.07153	-0.3246	7.624829	0.005757	0.735366	5	2	0	1	2	0	0	0	0	0
Gm34998	-4.06857	-0.76255	4.054787	0.044046	1	3	0	9	0	0	0	0	0	0	0
Gm38797	-4.06784	-0.70834	7.72804	0.005437	0.725022	2	2	6	0	1	0	0	0	0	0
Gm40675	-4.05844	-0.43839	5.510911	0.018898	1	2	0	3	6	1	0	0	0	0	0
Gm40506	-4.05708	-0.74573	5.894569	0.015188	1	3	0	1	8	0	0	0	0	0	0
LOC10816	-4.0408	0.9503	10.46398	0.001217	0.403928	0	2	0	0	8	0	0	0	0	0
4930463O	-4.03801	-0.76563	3.981434	0.046004	1	0	0	3	10	0	0	0	0	0	0
Gm41228	-4.03334	-0.81164	3.88805	0.048631	1	5	4	0	0	0	0	0	0	0	0
LOC10816	-4.01445	0.076658	8.211224	0.004163	0.648379	0	0	7	0	4	0	0	0	0	0
Gm10640	-4.01098	-0.30066	8.831704	0.00296	0.563513	2	3	17	8	2	0	0	0	1	0
LOC10524	-4.00534	-0.51656	6.896413	0.008637	0.905891	4	0	5	2	0	0	0	0	0	0
Snord42a	-4.00271	-0.58699	5.595418	0.018008	1	6	1	0	3	0	0	0	0	0	0

Gm38566	-4.00199	-0.44451	4.68674	0.030396	1	0	0	11	0	1	0	0	0	0	0
LOC10816	-3.97036	-0.57713	7.931563	0.004858	0.698042	1	0	1	7	2	0	0	0	0	0
Krt26	-3.96941	-0.70614	8.376419	0.003801	0.610501	0	4	2	3	1	0	0	0	0	0
Mir684-2	-3.96123	-0.64443	3.944696	0.047019	1	3	0	8	0	0	0	0	0	0	0
Gm42064	-3.94792	-0.77484	4.602135	0.031932	1	3	0	0	0	6	0	0	0	0	0
Defb20	-3.9451	-0.6944	4.939329	0.026252	1	0	0	3	9	0	0	0	0	0	0
Gm35029	-3.94234	-0.04812	6.805917	0.009086	0.923346	0	1	0	0	8	0	0	0	0	0
Arhgap9	-3.91426	-0.65555	7.360365	0.006668	0.791677	1	0	1	4	4	0	0	0	0	0
LOC10816	-3.90365	-0.3026	6.062123	0.013811	1	3	0	1	0	5	0	0	0	0	0
LOC10263	-3.89557	-0.69355	5.644949	0.017506	1	0	1	2	8	0	0	0	0	0	0
4930568G	-3.89517	-0.67564	4.908328	0.026728	1	1	0	10	0	0	0	0	0	0	0
Il5ra	-3.89167	-0.74542	4.564687	0.032638	1	1	0	10	0	0	0	0	0	0	0
Xist	-3.87333	-0.36598	5.594094	0.018021	1	1	0	0	10	0	0	0	0	0	0
Gm40250	-3.87219	-0.70829	5.766308	0.016336	1	3	0	0	6	1	0	0	0	0	0
Gm32024	-3.85875	-0.70679	5.92638	0.014916	1	2	1	2	5	0	0	0	0	0	0
Gm31805	-3.85094	-0.74473	5.938471	0.014814	1	0	0	6	1	3	0	0	0	0	0
Bglap	-3.8459	-0.64539	4.375427	0.036461	1	0	0	9	2	0	0	0	0	0	0
Ifitm5	-3.84137	-0.38923	7.973891	0.004746	0.69686	0	0	5	3	2	0	0	0	0	0
Cacng1	-3.83738	-0.84948	5.468738	0.01936	1	3	3	0	0	2	0	0	0	0	0
Gm36789	-3.8352	-0.83061	5.612225	0.017836	1	3	3	0	0	2	0	0	0	0	0
Gm31560	-3.83518	-0.51708	4.764004	0.029061	1	0	0	4	7	0	0	0	0	0	0
4930505A	-3.83009	-0.75932	7.130757	0.007577	0.846584	1	2	4	0	2	0	0	0	0	0
Gm13025	-3.82952	-0.79841	5.387541	0.020281	1	0	2	1	7	0	0	0	0	0	0
1700097N	-3.82134	-0.68907	7.053347	0.007912	0.858587	3	2	0	4	0	0	0	0	0	0
Gm39664	-3.81696	-0.4205	5.156227	0.023163	1	0	2	0	0	6	0	0	0	0	0
Gm4432	-3.81304	0.20365	8.46756	0.003615	0.610211	1	1	4	0	3	0	0	0	0	0
Scd4	-3.80854	0.751108	9.902768	0.00165	0.430212	0	1	4	0	4	0	0	0	0	0
Gm32647	-3.79852	-0.7932	5.733395	0.016645	1	5	1	0	0	2	0	0	0	0	0
Gm38523	-3.79116	0.136373	7.896904	0.004952	0.704155	0	4	0	0	4	0	0	0	0	0
Gm33305	-3.79099	-0.39081	5.88331	0.015285	1	1	3	1	0	3	0	0	0	0	0
Gm33185	-3.78855	-0.66059	6.810165	0.009064	0.923346	0	0	4	5	1	0	0	0	0	0
4930565N	-3.78426	-0.21061	5.744867	0.016537	1	8	0	0	0	0	0	0	0	0	0
Gm31344	-3.76707	-0.54029	6.506823	0.010746	0.95735	1	2	6	0	0	0	0	0	0	0
Zglp1	-3.75559	-0.17282	5.770936	0.016293	1	0	0	3	2	4	0	0	0	0	0
Ncaph	-3.74674	1.82745	9.876356	0.001674	0.430212	0	0	5	4	0	0	0	0	0	0
Stmnd1	-3.73845	-0.7086	7.730745	0.005429	0.725022	1	0	2	4	2	0	0	0	0	0
Gm29863	-3.72048	-0.83214	5.11761	0.023684	1	0	1	2	0	5	0	0	0	0	0
Gm42306	-3.71898	-0.64141	4.923164	0.026499	1	0	2	6	1	0	0	0	0	0	0
Amelx	-3.71581	-0.64002	3.84629	0.049856	1	0	0	8	2	0	0	0	0	0	0
Gpr63	-3.70875	-0.51657	4.544196	0.033031	1	0	0	3	7	0	0	0	0	0	0
5033426O	-3.70209	-0.77687	5.902706	0.015118	1	3	2	3	0	0	0	0	0	0	0
Gm11735	-3.70134	2.408161	9.206748	0.002411	0.519443	3	4	1	0	0	0	0	0	0	0
Gps1	-3.69834	-0.54661	4.596768	0.032032	1	2	0	0	2	4	0	0	0	0	0
Tssk1	-3.68499	-0.37596	4.865814	0.027394	1	0	6	1	0	0	0	0	0	0	0
Gm40820	-3.67952	-0.79726	4.430504	0.035302	1	1	0	7	0	1	0	0	0	0	0
Muc2	-3.67233	-0.50017	4.557809	0.032769	1	1	3	0	4	0	0	0	0	0	0
Ankrd1	-3.66099	0.39484	8.230617	0.004119	0.644276	5	0	3	0	0	0	0	0	0	0
Gm28874	-3.65186	-0.52679	5.128671	0.023534	1	2	2	0	4	0	0	0	0	0	0
Sntg2	-3.64495	-0.42973	5.455236	0.01951	1	9	0	12	0	2	0	0	0	1	0
Ms4a4c	-3.64302	-0.59032	6.041129	0.013976	1	0	3	5	0	0	0	0	0	0	0
Majin	-3.64237	1.08175	8.939408	0.002791	0.552875	4	0	4	0	0	0	0	0	0	0
Gm2814	-3.63533	1.159087	8.849298	0.002932	0.561798	2	0	0	4	2	0	0	0	0	0
Gm15326	-3.63024	-0.16227	6.553029	0.010471	0.954012	0	3	1	4	0	0	0	0	0	0
Ccdc178	-3.62672	-0.70868	5.863798	0.015455	1	1	0	4	0	3	0	0	0	0	0
Esrp2	-3.62513	3.864093	9.40562	0.002163	0.493936	0	1	0	7	0	0	0	0	0	0
Ly6g	-3.62379	-0.85247	5.631409	0.017641	1	4	0	2	2	0	0	0	0	0	0
Klk13	-3.61969	-0.61698	5.224101	0.022276	1	4	1	0	0	2	0	0	0	0	0
Gm41862	-3.6155	-0.45716	5.262443	0.02179	1	4	0	0	4	0	0	0	0	0	0
Sstr5	-3.60541	-0.60511	5.048256	0.024651	1	0	2	0	5	1	0	0	0	0	0
Dlk2	-3.60384	0.695791	7.49945	0.006172	0.759767	0	5	0	0	2	0	0	0	0	0
Pdilt	-3.60175	-0.05568	5.381826	0.020348	1	7	0	0	0	0	0	0	0	0	0
Gm42123	-3.59932	-0.29347	4.54194	0.033074	1	0	0	0	0	7	0	0	0	0	0
Tmem190	-3.59778	0.480331	8.23448	0.00411	0.644276	1	1	0	6	0	0	0	0	0	0
Gm35678	-3.56812	-0.55595	4.312508	0.037833	1	3	0	1	4	0	0	0	0	0	0
Slc10a5	-3.56551	-0.19063	4.446588	0.034971	1	0	0	0	9	0	0	0	0	0	0
Gm31764	-3.56511	-0.67957	4.871627	0.027302	1	1	0	0	5	2	0	0	0	0	0
Gm10754	-3.56353	-0.722	4.535657	0.033196	1	3	2	2	0	0	0	0	0	0	0
Gm15521	-3.53505	-0.6561	5.641129	0.017544	1	0	0	1	5	2	0	0	0	0	0

6430531B1	-3.51064	-0.79733	4.371921	0.036536	1	3	0	0	2	2	0	0	0	0	0
Zf12	-3.50938	0.581703	6.327456	0.011888	0.9915	4	0	2	0	1	0	0	0	0	0
LOC10263	-3.48628	-0.49572	6.60392	0.010175	0.943727	1	1	3	0	2	0	0	0	0	0
LOC10816	-3.4758	-0.76073	3.984896	0.04591	1	0	0	4	3	1	0	0	0	0	0
LOC10524	-3.47457	-0.83226	5.112803	0.02375	1	3	0	3	0	1	0	0	0	0	0
Ano9	-3.46623	3.633014	8.389291	0.003774	0.610501	3	0	4	0	0	0	0	0	0	0
Smc2os	-3.46228	-0.41325	5.676817	0.017191	1	8	4	5	2	0	0	0	1	0	0
Pcdhga4	-3.45982	-0.16104	4.575546	0.032431	1	0	3	0	0	3	0	0	0	0	0
Cldn3	-3.44651	3.5994	8.230633	0.004119	0.644276	0	0	7	0	0	0	0	0	0	0
Hnf1b	-3.44649	-0.79775	4.293698	0.038254	1	0	1	4	0	2	0	0	0	0	0
Gm41307	-3.42645	-0.59297	5.281092	0.021558	1	0	0	3	1	3	0	0	0	0	0
Gm30150	-3.42406	-0.83345	4.148724	0.041666	1	0	2	5	0	0	0	0	0	0	0
Gm38973	-3.41855	-0.15591	6.829942	0.008964	0.918794	7	0	10	2	1	0	0	0	1	0
Gm41233	-3.41367	-0.75609	4.683186	0.030459	1	2	1	0	0	3	0	0	0	0	0
Gm31125	-3.40395	-0.67477	4.289173	0.038356	1	0	2	0	5	0	0	0	0	0	0
Gm16222	-3.39938	-0.53728	7.142653	0.007527	0.846242	1	1	4	10	5	0	0	0	0	1
LOC10263	-3.38416	-0.43977	5.8133	0.015905	1	0	0	0	5	2	0	0	0	0	0
Gm39155	-3.38337	-0.11409	4.855254	0.027562	1	0	0	0	0	6	0	0	0	0	0
4930563F0	-3.37987	0.999332	6.682562	0.009736	0.941571	1	0	0	6	0	0	0	0	0	0
Gm35476	-3.37503	-0.44286	4.425508	0.035406	1	4	1	1	0	0	0	0	0	0	0
Tmem255b	-3.36993	-0.45934	4.409988	0.035729	1	1	3	2	0	0	0	0	0	0	0
Gm42127	-3.36273	1.371855	11.83317	0.000582	0.322039	8	0	5	0	5	0	0	0	1	0
1700026J1	-3.35208	-0.76043	3.90752	0.04807	1	0	0	0	5	2	0	0	0	0	0
Gm41909	-3.32946	-0.05791	5.797778	0.016046	1	0	0	5	2	0	0	0	0	0	0
Gm39939	-3.32816	-0.52889	5.105376	0.023852	1	8	2	4	5	0	0	0	0	0	1
Gm32576	-3.30912	-0.73992	4.865827	0.027394	1	1	1	0	2	2	0	0	0	0	0
Hist1h2an	-3.30626	0.617779	4.924547	0.026478	1	0	0	7	0	0	0	0	0	0	0
Gm13251	-3.30514	0.685959	11.70132	0.000625	0.340441	1	5	2	1	8	0	0	0	0	1
LOC10263	-3.30143	-0.33667	4.853019	0.027598	1	4	0	2	0	0	0	0	0	0	0
Gm5566	-3.29543	-0.57395	4.390011	0.03615	1	0	0	2	0	4	0	0	0	0	0
1700001J0	-3.29103	-0.44282	3.918693	0.047752	1	0	5	0	0	0	0	0	0	0	0
Ccdc63	-3.29017	-0.48796	4.748825	0.029318	1	8	1	2	4	2	0	0	0	1	0
Als2cr11b	-3.28965	-0.57324	5.558366	0.018393	1	4	0	0	2	0	0	0	0	0	0
LOC10816	-3.28291	0.567928	6.545109	0.010517	0.954012	0	0	2	0	4	0	0	0	0	0
LOC10816	-3.28222	-0.49168	4.056268	0.044008	1	0	0	7	0	0	0	0	0	0	0
Gm41406	-3.2804	-0.16888	4.914789	0.026628	1	0	0	0	7	0	0	0	0	0	0
C030004G	-3.27905	-0.83504	4.253745	0.039164	1	0	0	5	2	0	0	0	0	0	0
Gm35679	-3.25117	-0.50067	5.265551	0.021752	1	0	2	4	0	0	0	0	0	0	0
Lpar2	-3.2464	2.84672	7.090173	0.007751	0.854323	0	0	3	3	0	0	0	0	0	0
Gm40118	-3.23875	-0.85205	4.297388	0.038171	1	2	0	0	3	1	0	0	0	0	0
Spag17	-3.21412	-0.57414	5.14867	0.023264	1	0	0	4	0	2	0	0	0	0	0
Zfp92	-3.21058	0.630461	5.782971	0.016182	1	0	1	5	0	0	0	0	0	0	0
LOC10816	-3.20619	0.458784	9.740975	0.001802	0.444309	2	9	5	12	9	0	1	0	2	0
Sell	-3.20017	-0.63772	5.024407	0.024992	1	2	0	0	4	0	0	0	0	0	0
Ly6d	-3.19966	0.137676	4.99816	0.025374	1	2	0	0	4	0	0	0	0	0	0
Gm32057	-3.198	-0.00317	5.47073	0.019338	1	0	0	1	12	5	0	0	1	0	0
Ngf	-3.18842	-0.2821	4.930782	0.026382	1	0	3	0	0	2	0	0	0	0	0
Gm35470	-3.18644	-0.75665	4.498322	0.033928	1	0	2	0	0	3	0	0	0	0	0
LOC10816	-3.17911	-0.83373	3.88329	0.048769	1	1	0	0	4	1	0	0	0	0	0
Gm29681	-3.16403	-0.77899	4.934717	0.026322	1	0	0	3	2	1	0	0	0	0	0
Gm29771	-3.16233	-0.84997	4.436992	0.035168	1	0	3	1	0	1	0	0	0	0	0
Gm36235	-3.15478	-0.79644	4.24499	0.039366	1	0	1	0	5	0	0	0	0	0	0
LOC10263	-3.15407	-0.70503	3.866017	0.049273	1	1	0	0	0	4	0	0	0	0	0
Gm11532	-3.15051	0.559286	5.453606	0.019528	1	2	3	0	0	0	0	0	0	0	0
Bpifb9a	-3.13827	-0.49793	4.676164	0.030584	1	3	0	0	0	2	0	0	0	0	0
Gm39530	-3.13761	0.010486	5.69193	0.017043	1	2	0	13	0	2	0	0	0	1	0
A930001C0	-3.13726	0.144395	5.077443	0.024239	1	0	1	0	0	4	0	0	0	0	0
Gm30788	-3.12803	-0.27741	4.811301	0.028274	1	0	0	0	0	5	0	0	0	0	0
Platr22	-3.11219	-0.30721	4.573485	0.03247	1	0	0	4	2	0	0	0	0	0	0
Gm42312	-3.10643	-0.50792	4.201201	0.040395	1	0	0	6	0	0	0	0	0	0	0
Gm16596	-3.10137	-0.68898	4.052393	0.044109	1	0	0	6	0	0	0	0	0	0	0
Glis3	-3.10072	1.044378	12.69036	0.000368	0.274974	3	2	5	9	7	0	1	0	0	1
S100z	-3.09783	-0.09531	4.596954	0.032029	1	0	3	0	2	0	0	0	0	0	0
Gm34850	-3.09726	-0.55314	4.91164	0.026676	1	0	3	2	0	0	0	0	0	0	0
Gm34168	-3.09363	-0.70575	4.16592	0.041245	1	0	0	1	0	4	0	0	0	0	0
Gm36167	-3.08709	-0.38939	4.347352	0.037067	1	0	0	0	6	0	0	0	0	0	0
Gm28535	-3.06744	-0.67055	4.171521	0.041109	1	0	1	2	0	2	0	0	0	0	0
Gm39103	-3.04812	-0.79425	4.606648	0.031848	1	3	0	2	0	0	0	0	0	0	0

Fxyd4	-3.04733	2.914059	6.070753	0.013744	1	0	0	0	0	5	0	0	0	0	0	
2010107G	-3.04364	3.340222	6.107163	0.013464	1	5	0	0	0	0	0	0	0	0	0	
Slc39a4	-3.03767	5.019181	6.08165	0.013659	1	0	1	2	0	2	0	0	0	0	0	
Capns2	-3.03682	4.264512	6.029105	0.014072	1	0	0	2	0	3	0	0	0	0	0	
LOC10263	-3.02873	-0.22619	9.8846	0.001667	0.430212	4	5	5	1	8	0	1	0	0	1	
Zscan25	-3.02852	0.488239	6.786988	0.009182	0.924097	12	1	5	0	6	0	0	0	0	2	
Tert	-3.0204	1.517046	9.323223	0.002263	0.502051	9	0	3	0	2	0	0	0	1	0	
Gm35899	-3.01442	-0.14732	11.83911	0.00058	0.322039	4	2	14	5	10	1	0	0	0	2	
Tcap	-3.01442	-0.14316	5.36232	0.020576	1	6	2	4	18	5	3	0	0	0	0	
Gm32798	-3.00654	-0.89008	4.271745	0.038751	1	1	1	0	3	0	0	0	0	0	0	
Gpr141	-3.00239	-0.24079	7.363032	0.006658	0.791677	0	4	4	9	7	0	0	2	0	0	
Gm34232	-2.99896	0.29708	4.61975	0.031606	1	0	4	0	11	0	0	0	0	1	0	
Snord47	-2.99723	-0.3643	4.823602	0.028073	1	2	0	0	3	0	0	0	0	0	0	
Gm21057	-2.9911	-0.77965	3.921709	0.047666	1	0	0	3	0	2	0	0	0	0	0	
Nipal4	-2.9876	0.044837	8.496184	0.003559	0.603942	6	0	7	18	5	1	0	0	1	1	
Fam124b	-2.98411	-0.63848	4.237829	0.039533	1	1	0	0	3	1	0	0	0	0	0	
Exo1	-2.98195	0.880759	5.529809	0.018695	1	0	0	2	3	0	0	0	0	0	0	
Gm40055	-2.9751	-0.62615	6.774463	0.009247	0.924097	0	2	4	3	5	0	0	0	1	0	
Gm26524	-2.97407	-0.46751	4.349744	0.037015	1	0	1	4	0	0	0	0	0	0	0	
5430437J1	-2.96551	-0.89078	4.812873	0.028248	1	1	0	2	2	0	0	0	0	0	0	
Atp7b	-2.94905	-0.3129	4.226955	0.039787	1	3	4	2	0	4	0	0	0	0	1	
A330032B	-2.94433	0.084309	4.297262	0.038174	1	0	0	2	2	1	0	0	0	0	0	
8430419K	-2.94282	0.868306	6.724264	0.009511	0.931074	0	0	7	6	2	1	0	0	0	0	
Galr2	-2.9414	0.05459	5.212471	0.022425	1	2	8	0	2	0	0	0	0	1	0	
Dpcr1	-2.93018	-0.08147	4.169015	0.04117	1	0	4	0	0	0	0	0	0	0	0	
1810062G	-2.90922	-0.25911	6.107535	0.013461	1	0	1	6	14	4	0	1	0	0	1	
Gm40826	-2.9087	0.640064	10.9203	0.000951	0.380847	5	8	6	16	5	4	0	0	0	0	
1700010L	-2.90617	-0.48821	5.156352	0.023161	1	2	3	6	0	2	1	0	0	0	0	
Cbln4	-2.88702	-0.8492	4.09246	0.043075	1	1	2	0	0	1	0	0	0	0	0	
Gm17224	-2.87131	0.061235	5.32436	0.021029	1	2	3	1	6	1	0	0	0	1	0	
Gm15723	-2.86548	-0.37125	6.038799	0.013995	1	2	0	4	7	1	0	0	1	0	0	
D730001G	-2.86012	0.347072	4.60822	0.031819	1	0	4	0	0	0	0	0	0	0	0	
Tnip3	-2.85505	0.052166	3.889778	0.048581	1	4	0	0	0	0	0	0	0	0	0	
4930444A	-2.83522	-0.67199	4.183135	0.040828	1	2	0	0	0	2	0	0	0	0	0	
4930512B	-2.83477	0.442384	4.220134	0.039947	1	3	0	0	0	1	0	0	0	0	0	
Ccdc7a	-2.81847	0.322114	4.456884	0.034761	1	4	0	0	0	0	0	0	0	0	0	
Gm39334	-2.81318	-0.29567	4.645002	0.031144	1	0	2	1	1	8	0	0	0	0	1	
Mir6398	-2.81199	1.03892	7.400333	0.006521	0.78202	8	1	3	0	0	0	0	0	1	0	
Gm33997	-2.80807	0.368135	4.722882	0.029764	1	2	2	0	0	0	0	0	0	0	0	
Ntn4	-2.80676	0.773834	4.669187	0.030708	1	3	1	0	0	0	0	0	0	0	0	
Gm35551	-2.79891	1.360379	4.828494	0.027993	1	0	4	0	0	0	0	0	0	0	0	
Crb2	-2.79378	2.185433	4.842725	0.027763	1	0	4	0	0	0	0	0	0	0	0	
Gm7173	-2.79163	-0.16016	4.008747	0.045265	1	3	0	0	1	0	0	0	0	0	0	
Gm34656	-2.78956	2.007532	4.697593	0.030205	1	0	0	0	0	4	0	0	0	0	0	
Acap1	-2.78806	-0.38546	4.195986	0.04052	1	1	0	1	0	2	0	0	0	0	0	
Tnf	-2.78306	1.982892	4.751916	0.029266	1	0	0	0	0	4	0	0	0	0	0	
A830092H	-2.77555	-0.14263	10.22411	0.001386	0.406676	7	9	2	9	7	0	1	0	3	0	
Ost4	-2.76518	0.043592	5.239282	0.022082	1	0	0	4	6	3	0	0	1	0	0	
Ap1m2	-2.7603	5.179818	4.919292	0.026558	1	2	2	0	0	0	0	0	0	0	0	
Sapcd2	-2.75818	1.316722	6.950162	0.008381	0.889442	0	5	0	0	6	0	0	0	1	0	
Lamb3	-2.75734	4.229762	4.88093	0.027155	1	0	1	2	0	1	0	0	0	0	0	
Lvrn	-2.74691	0.903069	4.33884	0.037252	1	0	0	1	1	2	0	0	0	0	0	
Cyp2u1	-2.74002	-0.08276	7.621831	0.005767	0.735366	6	0	7	2	5	1	0	0	0	1	
Smim17	-2.73101	-0.17029	8.071009	0.004498	0.677057	3	0	13	7	7	0	0	0	1	2	
Gm38851	-2.7262	2.030239	7.323302	0.006807	0.797639	0	4	0	0	7	0	0	1	0	0	
4930579H	-2.72565	-0.26614	3.952503	0.046802	1	10	4	0	9	9	0	2	0	2	0	
Gm39299	-2.72531	1.102693	6.886884	0.008683	0.908085	4	3	0	0	4	0	0	0	0	1	
Kif27	-2.72254	-0.06708	7.700458	0.005521	0.725022	5	1	6	12	12	0	0	0	3	1	
Psg17	-2.71864	0.111366	4.247086	0.039318	1	0	0	3	0	1	0	0	0	0	0	
Pigr	-2.71133	1.887215	4.526044	0.033383	1	0	0	0	4	0	0	0	0	0	0	
Gm15753	-2.70601	0.078622	4.156647	0.041471	1	1	0	0	3	0	0	0	0	0	0	
Gm31728	-2.69491	-0.07454	6.382734	0.011524	0.987077	1	9	6	5	5	1	0	0	1	1	
LOC10816	-2.67935	0.006465	4.202134	0.040373	1	0	0	0	4	0	0	0	0	0	0	
Gm9861	-2.65564	0.232017	3.936794	0.047241	1	0	0	0	4	0	0	0	0	0	0	
LOC10816	-2.61321	-0.09169	4.156107	0.041485	1	0	0	3	22	3	0	3	0	0	0	
Mybl2	-2.61245	1.300121	6.275786	0.01224	0.9915	4	1	0	6	0	0	0	0	0	1	
LOC10816	-2.60126	0.498688	7.030636	0.008013	0.858587	9	3	0	0	4	0	1	1	0	0	
Btbd16	-2.56199	-0.22862	7.197949	0.007299	0.826025	1	7	3	10	3	0	1	2	0	0	

Il15	-2.553	1.067525	10.27293	0.00135	0.406676	4	9	6	2	2	0	0	1	2	0
Gm35051	-2.52188	-0.27816	5.350609	0.020715	1	0	0	5	8	5	0	0	1	1	0
Gm30339	-2.50323	-0.1851	6.055363	0.013864	1	3	0	6	7	2	0	0	1	0	1
Gm39463	-2.49734	-0.29109	4.279471	0.038575	1	0	12	14	0	2	0	1	1	2	0
Gm38484	-2.48132	-0.72508	4.345405	0.037109	1	0	1	1	5	3	0	0	0	1	0
Gm39497	-2.46825	-0.1503	3.984709	0.045915	1	0	2	0	3	10	0	0	0	2	0
Slc5a1	-2.46141	6.350938	9.949871	0.001609	0.427077	5	1	0	5	5	0	0	0	2	0
Gm35585	-2.45893	0.234085	3.98935	0.045789	1	1	2	2	0	4	1	0	0	0	0
B830017H(	-2.43864	-0.0851	6.712162	0.009576	0.931074	5	13	4	6	4	0	1	3	1	0
A430110L2	-2.42593	1.081789	8.84695	0.002936	0.561798	3	4	8	3	4	1	1	0	0	1
Krt9	-2.38885	-0.2997	4.620967	0.031583	1	3	7	7	0	3	0	0	2	1	0
Efcab6	-2.3886	4.528462	23.6673	1.15E-06	0.041197	9	4	9	12	12	5	0	0	0	2
LOC10816	-2.37735	1.496341	5.730682	0.016671	1	2	0	4	0	3	0	0	0	1	0
Spata18	-2.37168	-0.42149	4.009189	0.045253	1	4	4	0	4	3	0	0	0	0	2
Llgl2	-2.36731	5.034575	5.992139	0.01437	1	1	0	3	5	0	0	0	1	0	0
Gm41293	-2.36034	0.375627	4.966334	0.025845	1	3	0	0	5	1	0	1	0	0	0
Akr1b7	-2.35652	6.637722	5.845757	0.015615	1	0	0	2	7	0	0	0	0	1	0
Egf	-2.33889	0.826266	4.636638	0.031296	1	0	2	7	0	0	0	0	0	1	0
Cd40	-2.33106	0.328025	5.398986	0.020148	1	3	2	10	0	0	0	0	1	1	0
Tmlhe	-2.30351	0.321714	6.215414	0.012664	1	3	2	0	16	11	0	0	1	4	0
1700061G	-2.29511	-0.18249	4.927341	0.026435	1	0	7	10	4	4	0	3	0	1	0
Azgp1	-2.29397	0.121049	4.49365	0.034021	1	3	3	7	0	1	0	0	0	2	0
Gm32311	-2.28923	0.278742	6.05283	0.013884	1	1	2	11	3	4	2	0	0	0	1
Slfn10-ps	-2.27002	2.327726	4.833985	0.027904	1	2	3	0	0	3	0	0	0	1	0
Gm40142	-2.21314	0.579779	6.549326	0.010492	0.954012	5	1	0	11	3	0	0	0	0	3
Ccdc69	-2.20612	1.060621	4.470145	0.034492	1	3	0	4	1	0	0	1	0	0	0
Gm42244	-2.20129	0.612909	4.583596	0.032279	1	3	0	12	3	2	0	0	1	2	0
Hrc	-2.19403	0.371369	4.809237	0.028308	1	7	1	0	4	1	0	1	0	0	1
Gm8430	-2.19337	0.976852	8.377647	0.003799	0.610501	15	3	4	8	5	0	1	0	1	4
Gm34872	-2.16918	1.413702	6.302685	0.012056	0.9915	3	3	7	4	12	0	0	3	0	2
Murc	-2.14821	1.476253	9.65808	0.001885	0.461375	4	3	14	3	11	1	0	0	0	5
LOC10264(	-2.14312	0.167092	5.052843	0.024586	1	7	0	4	13	1	3	0	0	0	1
Gpx2-ps1	-2.11478	-0.10381	4.809149	0.028309	1	2	5	2	17	3	2	2	0	0	1
Gm30064	-2.09625	2.510129	10.00757	0.001559	0.418551	15	0	4	5	3	1	1	0	2	1
Gm39603	-2.09022	0.605588	4.869881	0.027329	1	4	1	5	1	6	0	1	0	1	1
Gm36283	-2.08159	1.075031	5.441541	0.019663	1	9	6	1	0	0	1	0	0	0	2
Gm41831	-2.06443	1.928287	4.014484	0.045111	1	5	0	0	0	2	0	0	0	1	0
Gm34079	-2.06406	1.088274	6.150078	0.013141	1	1	1	13	8	1	0	1	2	0	1
Cldn23	-2.05185	5.5181	4.127157	0.0422	1	0	5	0	0	2	0	0	1	0	0
Dhx58	-2.02989	1.634751	6.085581	0.013629	1	11	0	18	10	0	3	1	1	0	2
Hk1os	-2.02814	0.444392	4.503377	0.033828	1	0	0	4	8	14	1	1	0	3	0
Zbtb49	-2.00275	1.174917	10.98168	0.00092	0.376182	11	11	11	9	1	1	6	1	1	0
Gm30694	-1.99924	0.809768	5.31259	0.021172	1	9	0	4	9	5	0	0	1	1	3
Gbp8	-1.99383	0.853443	5.803302	0.015996	1	12	2	0	3	7	0	2	0	1	2
Lrp2	-1.94738	0.156916	4.979567	0.025648	1	3	2	4	10	11	0	0	1	3	2
LOC10263:	-1.93442	0.911137	5.202274	0.022557	1	3	0	11	3	4	3	0	0	0	1
Akr1c14	-1.9342	-0.03413	4.116484	0.042467	1	6	1	8	10	5	1	0	5	0	0
Prss22	-1.93284	7.360883	5.24411	0.022021	1	1	0	0	8	2	0	0	0	2	0
E130008D(	-1.90012	2.219985	6.0186	0.014156	1	3	3	0	7	2	1	0	1	1	0
Gck	-1.89829	0.306343	4.573732	0.032466	1	7	6	13	8	1	1	1	1	0	4
AI661453	-1.89737	9.20547	15.19362	9.70E-05	0.166227	4	1	12	11	9	0	3	0	4	1
Nuf2	-1.88225	1.894024	5.442937	0.019648	1	10	1	2	0	9	0	0	0	4	1
Pyhin1	-1.87607	3.796421	8.85725	0.002919	0.561798	1	3	8	7	9	0	3	0	1	2
Gtpbp10	-1.87434	0.657135	5.364004	0.020557	1	5	2	4	14	4	0	1	4	0	1
Lyz2	-1.86549	1.165715	13.24765	0.000273	0.265369	9	12	33	39	22	3	3	4	9	6
Adrb3	-1.83654	1.489573	4.479508	0.034304	1	0	1	0	20	0	0	0	0	3	1
Acta1	-1.82587	0.826475	5.911847	0.015039	1	15	30	12	24	4	1	3	2	12	3
Stag3	-1.82373	2.05986	5.058453	0.024506	1	3	2	3	2	4	0	0	0	3	0
Ltb4r1	-1.81214	3.36368	4.30019	0.038108	1	0	0	4	1	5	0	0	0	2	0
Crhbp	-1.8024	1.012967	5.783148	0.016181	1	8	3	6	14	16	2	4	1	1	3
Slc2a5	-1.79486	0.543065	4.318221	0.037706	1	28	3	11	22	15	3	14	0	2	1
Gm17059	-1.77146	1.385467	5.705418	0.016913	1	8	0	4	4	2	0	0	0	1	3
LOC10816	-1.76946	0.960611	4.037996	0.044487	1	1	1	1	9	6	0	0	0	4	0
Gramd2	-1.76385	1.577619	6.256761	0.012372	0.9915	2	7	8	3	5	0	3	1	1	1
5430416O(	-1.75936	0.094957	4.778219	0.028822	1	8	8	5	11	2	4	0	1	0	3
6330407A(	-1.75761	2.004543	7.597464	0.005845	0.735366	0	14	8	5	5	0	1	4	3	0
Gm16230	-1.74556	0.64547	4.156619	0.041472	1	2	4	0	13	0	0	0	0	0	4
Sp110	-1.74551	0.685936	5.519567	0.018805	1	8	4	5	16	6	1	1	1	2	4

Gm34342	-1.73894	-0.51366	4.016771	0.04505	1	2	5	3	5	2	0	0	3	1	0
Grap2	-1.71752	0.892091	4.201892	0.040379	1	0	2	0	15	1	0	3	0	1	0
Vsig10l	-1.68377	4.419813	16.13582	5.90E-05	0.152284	17	10	22	11	13	9	1	0	5	4
Gm30164	-1.65643	1.605166	4.635979	0.031308	1	5	0	4	0	7	0	0	2	1	1
Slc2a12	-1.6537	1.3173	6.271405	0.01227	0.9915	9	2	2	14	1	0	3	0	2	2
Gm42245	-1.64925	0.843785	4.025434	0.044819	1	8	1	9	0	5	4	0	0	2	0
Gm12174	-1.61714	1.050667	5.587704	0.018087	1	1	0	6	10	7	2	0	1	2	1
Gm34538	-1.61312	0.633975	5.576287	0.018205	1	2	5	12	12	14	1	2	5	3	1
Gm4890	-1.60563	0.926525	5.433574	0.019753	1	2	5	2	11	3	2	3	0	0	1
Fcrlb	-1.60242	0.071912	3.960345	0.046584	1	7	8	15	5	5	0	3	5	2	1
Serpinb5	-1.5974	7.299767	4.325265	0.037551	1	3	1	3	0	5	3	0	0	0	0
Gm32497	-1.59294	1.726494	6.192928	0.012826	1	11	7	5	7	15	0	7	2	1	3
D830035M	-1.57158	0.729253	4.772475	0.028918	1	5	11	15	11	4	4	4	3	2	0
Gm40181	-1.57011	1.175769	4.295509	0.038213	1	6	2	3	11	11	3	0	0	4	2
9930014A1	-1.55012	2.603883	8.980893	0.002728	0.552875	6	8	0	18	7	3	3	1	2	2
Rps15a-ps(	-1.54655	1.787419	6.061593	0.013815	1	5	1	3	12	5	3	0	0	2	2
Guca1b	-1.5337	1.801386	4.803985	0.028394	1	1	2	6	10	0	0	1	0	4	0
Gm35566	-1.51169	1.852335	6.665265	0.009831	0.943727	4	2	4	9	12	3	3	0	3	0
Cdrt4	-1.50957	-0.35631	4.161075	0.041363	1	4	4	3	6	1	0	1	2	1	1
Igsf6	-1.49943	1.00146	5.593303	0.018029	1	16	8	6	18	14	4	0	2	5	7
Peak1os	-1.47581	0.916548	8.445343	0.00366	0.610501	10	7	7	12	13	4	2	7	2	0
Glt28d2	-1.47156	3.914446	6.713125	0.009571	0.931074	9	3	11	0	1	0	0	2	3	2
Plac8	-1.44992	6.686629	3.953397	0.046777	1	5	1	5	0	3	1	0	1	0	2
1700124L1	-1.44471	0.070478	4.272817	0.038727	1	8	5	9	5	6	1	3	2	1	3
Gm31504	-1.434	1.726192	6.647528	0.009929	0.943727	4	5	14	26	3	4	4	3	2	2
Gm11696	-1.42478	0.885454	4.6597	0.030879	1	8	7	9	22	8	4	0	3	5	4
Gdap10	-1.42015	1.515614	6.535855	0.010572	0.954012	4	13	18	4	11	5	2	6	2	1
Slc16a3	-1.41523	2.383755	9.159411	0.002474	0.526744	13	14	23	9	13	1	3	7	9	3
Chek2	-1.41453	3.494383	8.434825	0.003681	0.610501	2	4	11	9	13	0	2	3	4	3
Cd84	-1.40927	0.396386	5.576558	0.018202	1	9	5	11	15	6	1	0	6	5	2
Doc2g	-1.3959	1.611337	5.709534	0.016873	1	6	3	8	12	5	0	0	1	1	8
Zfp607	-1.37906	1.988881	4.919482	0.026555	1	6	0	9	5	3	0	0	1	4	2
Npas2	-1.36683	2.257403	5.792428	0.016095	1	12	4	9	8	5	1	0	1	5	5
Suv39h2	-1.35277	1.30758	5.450828	0.019559	1	10	5	8	1	12	3	1	2	2	4
Gm41581	-1.32699	1.302428	4.699038	0.03018	1	15	5	16	1	8	0	6	2	1	6
Sik1	-1.31821	4.46327	15.3095	9.13E-05	0.164152	11	30	23	12	16	6	9	0	12	5
Hmgn2	-1.30337	2.612205	7.935514	0.004847	0.698042	1	7	24	11	11	2	5	5	6	0
Akr1b3	-1.30264	1.860663	4.504568	0.033804	1	4	5	1	6	23	6	0	2	5	1
Poc1a	-1.30064	1.723934	4.684302	0.030439	1	6	10	16	14	5	2	0	0	13	2
LOC10816(	-1.29754	1.93314	4.22388	0.039859	1	6	7	10	0	3	0	3	5	1	0
Mpeg1	-1.29235	1.534438	5.732055	0.016658	1	33	17	20	49	18	8	4	15	9	10
Gm41526	-1.27053	2.54414	4.636504	0.031299	1	7	6	5	15	29	2	0	4	4	11
Qpct	-1.26952	2.140713	6.657663	0.009873	0.943727	6	5	22	5	12	3	2	6	2	4
Il1rl2	-1.26822	3.421453	5.901621	0.015127	1	19	2	0	9	4	8	2	1	0	1
1700008J0	-1.26537	1.879957	4.134606	0.042015	1	1	2	2	23	9	0	6	0	2	4
Rgs14	-1.26291	0.954875	8.390211	0.003772	0.610501	10	13	9	24	25	8	8	4	5	4
Galns	-1.2474	2.18942	7.62085	0.00577	0.735366	7	9	16	20	27	6	6	7	4	5
Shroom4	-1.24125	3.032747	9.779569	0.001765	0.443943	7	7	9	30	25	2	1	5	9	10
Rab42	-1.23843	2.027519	8.15078	0.004304	0.658937	11	12	22	20	10	0	5	9	4	8
Frk	-1.23837	5.449467	5.010815	0.025189	1	10	1	5	1	6	3	0	0	1	4
Shq1	-1.23828	1.814038	6.925856	0.008496	0.893702	18	2	12	16	15	3	3	6	3	7
Crlf1	-1.23739	5.629386	15.66999	7.54E-05	0.159588	11	19	21	26	7	6	9	5	8	2
LOC10524	-1.23242	0.430041	5.371239	0.020471	1	3	6	13	13	9	3	2	1	3	6
LOC10816(	-1.23215	1.760419	4.991847	0.025467	1	11	0	18	24	9	1	4	1	13	2
Zkscan7	-1.22189	1.974751	5.235317	0.022133	1	6	10	4	39	5	0	7	2	9	4
Zfp114	-1.21947	1.064192	4.591726	0.032127	1	12	14	9	6	4	4	2	2	7	2
Spint2	-1.21776	6.07095	4.102186	0.042828	1	2	3	7	3	5	0	3	1	2	1
4930451G(	-1.21649	1.134012	6.524978	0.010637	0.954012	18	16	17	14	16	8	3	6	7	6
B230209E1	-1.2145	0.884829	5.553161	0.018447	1	14	12	8	24	25	3	9	11	4	4
Ccna2	-1.19919	3.641712	5.377476	0.020398	1	0	12	7	27	3	6	0	5	2	4
LOC10816(	-1.17213	1.565389	5.023045	0.025012	1	8	33	27	19	8	8	9	12	5	3
C230037L1	-1.16242	1.425487	4.838072	0.027838	1	31	21	19	27	17	18	6	14	3	4
Zfp970	-1.15511	1.233089	3.905194	0.048137	1	2	9	21	14	21	0	3	7	4	10
Ifi44	-1.15285	2.580441	5.281123	0.021558	1	22	26	41	24	19	11	19	8	5	8
Ube2cbp	-1.13657	1.17595	4.046604	0.04426	1	8	8	17	11	4	6	3	6	1	2
1700086L1	-1.12696	1.761141	10.24345	0.001372	0.406676	17	28	36	40	34	6	15	5	21	13
Zfp879	-1.11887	1.158359	4.166267	0.041237	1	9	10	11	14	11	0	8	8	6	0
Gm11802	-1.11675	1.905136	5.109422	0.023796	1	7	2	20	20	13	8	7	2	2	4

Ucp3	-1.09293	1.354878	4.480531	0.034283	1	1	6	16	9	19	0	4	6	7	3
Gm40466	-1.08777	2.081532	3.969618	0.046328	1	19	4	7	15	11	0	3	11	2	6
Mdfi	-1.0837	4.449688	5.086552	0.024112	1	14	2	15	2	5	5	2	6	0	2
Hmgb2	-1.07719	5.203583	11.3018	0.000774	0.357189	14	11	30	34	8	13	10	3	8	4
Gm4604	-1.07568	1.388454	4.575913	0.032424	1	10	2	4	14	8	3	4	5	1	2
Foxq1	-1.07336	6.556928	10.35526	0.001291	0.406402	23	9	9	11	17	9	0	9	6	4
Rbm12b1	-1.06884	1.486739	4.044887	0.044305	1	21	7	8	7	25	2	5	9	9	4
Aass	-1.06593	1.638363	4.179968	0.040904	1	10	7	8	20	10	5	7	5	2	3
Tst	-1.0655	2.181935	5.119243	0.023662	1	10	5	11	19	6	6	6	0	4	4
Itgam	-1.0423	6.288008	8.751705	0.003093	0.574754	10	7	12	13	19	3	10	0	4	8
Fbxo5	-1.03775	2.669881	3.911832	0.047947	1	15	3	18	2	12	8	6	6	0	1
Klrg2	-1.03709	5.343846	11.19913	0.000818	0.357189	11	13	14	32	25	13	0	4	16	6
1700030J2	-1.03324	2.043224	5.320637	0.021074	1	33	31	41	34	17	10	27	8	17	5
Lmx1b	-1.02373	3.992667	4.718571	0.029839	1	5	6	12	2	9	0	1	4	5	4
Spc25	-1.02215	3.630913	4.967523	0.025828	1	19	15	8	13	5	2	4	13	6	1
Efcab12	-1.01961	1.406274	5.06264	0.024447	1	26	16	15	12	14	7	13	5	3	8
Slc25a42	-1.01172	2.072447	6.343732	0.01178	0.9915	16	19	39	18	16	7	17	7	12	3
Tmem194b	-1.00813	2.449044	6.785623	0.009189	0.924097	15	12	10	21	28	4	8	5	14	6
Card14	-1.0072	3.3965	5.80128	0.016015	1	25	12	12	5	18	5	8	6	9	4
Gm35113	-1.00422	1.839731	4.327311	0.037505	1	21	16	22	37	34	17	21	13	5	1
Fkbp10	-0.99513	3.050188	11.01722	0.000903	0.374472	23	33	56	62	43	24	22	11	17	17
Serpinc1	-0.99321	2.170832	5.094257	0.024005	1	17	17	21	12	20	5	4	9	6	13
Cbx2	-0.99247	2.539035	7.422747	0.00644	0.774921	22	11	43	40	15	14	13	10	11	6
Nfatc4	-0.98833	2.469069	6.35922	0.011677	0.9915	12	10	22	14	7	0	4	5	8	10
Tbc1d2	-0.97436	4.201587	4.100906	0.04286	1	3	1	17	4	6	3	0	0	10	0
Layn	-0.97208	2.262425	4.878767	0.027189	1	30	19	34	18	31	22	1	8	11	15
Upp1	-0.9699	4.707061	4.428126	0.035351	1	6	12	15	13	5	12	4	2	3	1
Gm13840	-0.96691	2.462062	3.918055	0.04777	1	27	5	27	30	11	12	7	15	3	5
Polm	-0.95322	2.552648	4.735238	0.029551	1	18	9	6	7	13	6	4	2	8	4
4930522L1	-0.95245	2.94478	5.813594	0.015903	1	7	11	19	23	19	3	10	5	8	8
Kcng3	-0.94634	1.92344	4.252108	0.039202	1	3	7	7	20	14	4	2	7	6	3
Kcnn2	-0.94367	1.367222	5.427071	0.019827	1	16	29	12	31	20	4	15	7	13	10
Gm31105	-0.94236	1.679691	4.808626	0.028318	1	7	5	35	18	14	5	11	6	3	8
Qtrtd1	-0.92391	2.373979	5.488044	0.019147	1	8	39	24	34	23	12	12	11	17	7
Calcrl	-0.92037	2.382749	4.084194	0.043286	1	52	27	37	63	20	25	22	5	28	10
Mettl25	-0.91799	2.261467	6.626588	0.010047	0.943727	28	16	33	36	28	10	7	10	19	16
Zfp780b	-0.91751	2.846673	8.557969	0.00344	0.590595	36	28	40	43	35	14	19	17	23	10
Fam83h	-0.91169	6.331429	12.48168	0.000411	0.275262	25	15	39	22	23	4	14	15	17	6
Zfp526	-0.90782	3.504184	12.4374	0.000421	0.275262	31	48	54	78	33	30	13	18	28	20
Adamts10	-0.90333	3.31064	6.214803	0.012669	1	68	28	34	76	54	24	27	22	30	17
St3gal1	-0.89095	2.912395	6.258648	0.012359	0.9915	32	42	40	55	23	22	13	17	14	21
Hyal2	-0.88992	3.975811	11.23338	0.000803	0.357189	17	37	39	47	46	16	19	16	31	5
Ctso	-0.8842	3.64109	6.542488	0.010533	0.954012	16	22	44	23	19	8	6	14	16	12
C330018D2	-0.87461	3.702795	6.258456	0.01236	0.9915	30	22	47	16	31	10	13	3	26	16
Slc34a2	-0.86829	6.161563	5.596521	0.017996	1	8	6	22	5	11	4	6	2	9	3
LOC10816	-0.86594	4.137221	5.430425	0.019789	1	13	1	20	15	8	5	0	7	13	1
Dtwd1	-0.86582	2.718265	7.100577	0.007706	0.854323	15	16	29	50	14	12	10	20	8	6
Phf11d	-0.86298	4.216299	4.300429	0.038103	1	3	12	15	19	2	2	9	2	2	8
Tpcn2	-0.85831	3.876267	6.052193	0.013889	1	22	12	34	44	6	19	13	9	13	0
Papss2	-0.85764	5.380007	7.295254	0.006914	0.802346	18	8	24	23	9	5	11	7	11	4
Cdk5rap1	-0.85212	3.012158	5.302283	0.021297	1	35	36	55	23	25	13	18	18	18	16
Sh3rf2	-0.84982	3.721669	4.054454	0.044055	1	4	7	16	10	2	10	0	3	4	1
Lfng	-0.84753	4.213977	11.18962	0.000823	0.357189	53	51	75	41	33	42	23	29	16	12
Fcor	-0.84093	1.148237	4.186815	0.04074	1	13	18	18	12	15	6	8	7	11	5
2610306M	-0.83841	2.160691	4.152341	0.041577	1	34	41	41	29	22	20	13	20	12	16
Gnb1l	-0.82249	2.493923	4.811696	0.028267	1	17	12	21	31	11	6	12	1	13	11
Psmb9	-0.81819	4.161975	8.014903	0.004639	0.689698	16	21	30	39	12	11	11	9	16	9
Ttc8	-0.81598	2.910489	5.127678	0.023547	1	15	7	37	41	41	20	12	11	12	11
Gpt	-0.81493	5.070622	8.247711	0.00408	0.644276	16	14	14	30	25	8	18	8	7	7
Fgd2	-0.80673	2.775318	4.784929	0.02871	1	19	4	21	14	18	4	12	6	11	4
Zfp213	-0.80628	2.018394	5.165976	0.023034	1	12	10	14	21	15	10	8	3	11	3
Shb	-0.80071	4.595778	4.967727	0.025825	1	17	9	17	2	10	6	3	3	11	4
Tfap4	-0.80036	2.961479	6.093142	0.013571	1	35	22	31	49	19	16	17	15	11	16
Prpsap2	-0.79897	3.761612	8.911742	0.002833	0.552875	31	78	111	73	85	21	49	33	67	19
Zfp839	-0.7959	4.03681	8.18987	0.004212	0.651242	28	31	38	56	37	12	35	11	25	11
Il18	-0.79016	6.324953	14.07671	0.000176	0.218121	47	32	41	43	56	28	10	31	22	18
Lypd6b	-0.78512	3.162364	5.527916	0.018715	1	25	18	60	68	38	5	33	13	35	14
Ttc12	-0.77707	2.661642	6.101587	0.013506	1	50	41	54	79	22	13	38	21	22	26

B230311B(	-0.77351	1.359375	4.235227	0.039593	1	13	21	28	33	18	9	15	10	15	7
Mgmt	-0.76339	2.866177	4.234794	0.039603	1	14	25	23	27	11	4	11	4	11	19
Irf5	-0.76286	3.213576	4.024023	0.044857	1	12	17	7	22	16	0	13	13	8	4
Mapkapk3	-0.76139	5.805963	7.302727	0.006885	0.801601	16	5	31	28	11	11	16	10	5	3
Tmem200c	-0.75072	1.254296	4.239098	0.039503	1	14	15	16	33	20	10	10	14	6	9
Slc13a3	-0.74556	3.157902	3.902516	0.048214	1	67	59	68	134	72	40	38	26	52	43
Pard6b	-0.72917	4.98795	6.534668	0.010579	0.954012	29	15	21	19	22	14	2	13	19	7
Ercc6	-0.72101	3.343364	6.725346	0.009505	0.931074	34	48	40	64	39	17	12	25	37	25
Fgd6	-0.72032	4.18127	4.795515	0.028534	1	11	8	29	12	15	8	1	19	2	8
6030419C1	-0.71597	4.406746	4.781324	0.02877	1	84	272	289	210	191	94	236	84	88	65
Fzd7	-0.71428	3.947266	9.497232	0.002058	0.479868	89	66	112	82	56	41	62	44	32	33
Serac1	-0.70957	3.085538	7.709963	0.005492	0.725022	49	21	44	58	44	25	18	25	27	17
Myd88	-0.70697	4.72838	11.11825	0.000855	0.361801	37	22	77	36	59	26	19	22	35	18
A430046D	-0.69771	2.031202	5.315246	0.02114	1	37	17	33	33	30	13	27	12	19	9
Casp6	-0.69365	3.719113	6.31332	0.011983	0.9915	14	18	45	31	24	19	12	11	12	14
Pln	-0.68723	2.22915	4.193953	0.040568	1	26	36	64	83	29	11	41	23	27	20
Fcer1g	-0.68576	2.351124	4.387758	0.036198	1	33	38	42	78	43	20	32	29	28	15
Fkbp14	-0.68124	3.661386	14.21703	0.000163	0.217041	92	78	111	111	109	60	69	47	55	39
C1ql4	-0.67532	2.097224	4.497815	0.033938	1	37	43	31	49	39	26	17	32	26	9
Fastkd1	-0.67459	2.246896	4.799628	0.028466	1	26	40	46	31	36	21	23	15	26	13
Them5	-0.67432	2.671466	4.744473	0.029393	1	55	35	92	114	36	33	52	49	20	18
Ramp3	-0.67272	2.781909	4.278989	0.038586	1	71	63	71	89	26	32	42	48	31	20
Fgf7	-0.66797	1.952963	4.392119	0.036105	1	34	18	32	49	29	25	15	15	18	13
Exd2	-0.66141	2.622812	4.172487	0.041085	1	48	29	48	20	41	9	27	27	21	19
Slc46a1	-0.66067	3.308203	6.148945	0.013149	1	41	45	124	96	57	27	53	59	31	21
Nupl2	-0.65907	2.824836	4.519029	0.03352	1	27	28	28	30	33	21	20	18	13	9
Ly6a	-0.64151	7.221271	8.046777	0.004558	0.683314	89	61	55	74	54	25	53	38	40	31
Supv3l1	-0.63954	3.306218	4.495179	0.033991	1	46	37	71	108	24	38	39	24	35	16
Plppr2	-0.63662	3.66307	9.511866	0.002041	0.479868	90	94	123	96	102	66	60	66	67	26
Qsox2	-0.63514	3.618463	6.528526	0.010616	0.954012	20	36	54	59	43	9	38	30	24	15
Tuft1	-0.63511	6.354149	5.126788	0.023559	1	13	15	10	35	15	19	3	13	9	4
Tpd52l1	-0.63375	3.242343	4.742093	0.029433	1	29	28	63	69	18	16	22	27	25	19
Golga7	-0.63184	3.784102	5.659209	0.017364	1	18	20	57	52	64	28	24	22	27	14
Idh2	-0.62852	4.746269	9.586283	0.00196	0.470174	127	60	160	163	59	59	57	78	56	54
Mir6236	-0.62629	4.870336	5.180945	0.022836	1	166	111	158	165	66	125	37	92	63	51
Ntmt1	-0.625	3.602929	4.988733	0.025513	1	77	74	134	92	76	48	44	48	55	52
Zfp1	-0.62096	3.573561	8.037288	0.004582	0.684053	67	55	68	99	56	42	51	35	36	28
Wnt6	-0.6201	4.057341	8.632589	0.003302	0.581129	68	69	118	183	87	59	64	48	69	44
Stx3	-0.61624	5.012492	9.488643	0.002067	0.479868	69	47	75	141	53	37	44	51	49	29
Fam45a	-0.60918	4.326465	10.62002	0.001119	0.403208	111	115	172	136	98	87	73	72	70	53
Uckl1	-0.6089	4.49046	6.521093	0.01066	0.954012	55	49	87	89	47	41	46	19	47	28
Card10	-0.60387	5.294429	5.445957	0.019614	1	173	106	190	177	134	126	95	104	37	75
Thbs1	-0.60121	4.739543	4.920666	0.026537	1	34	41	29	54	29	31	25	21	17	13
Amn1	-0.59945	3.460758	5.702702	0.016939	1	51	39	90	64	42	31	28	33	38	28
Cxcl16	-0.5949	3.997766	5.377965	0.020393	1	45	37	60	51	32	35	28	18	26	20
Kpna6	-0.59286	4.650398	12.233	0.00047	0.281521	73	103	169	166	106	64	64	57	102	57
Szt2	-0.59104	3.135575	4.590996	0.03214	1	28	22	56	55	45	27	21	22	31	14
Amt	-0.58938	3.97767	4.038368	0.044477	1	29	22	59	21	31	18	13	26	26	9
Cyp39a1	-0.5879	4.920224	8.647402	0.003275	0.580451	82	70	86	92	45	47	27	47	49	42
Fastkd5	-0.58578	2.512131	4.039281	0.044453	1	29	14	25	35	35	14	10	16	22	16
Adrb1	-0.5833	5.044413	5.139487	0.023388	1	46	35	42	55	14	12	16	28	38	15
Asun	-0.57885	4.614888	5.747495	0.016512	1	66	24	85	83	51	34	46	29	30	33
Slc7a6os	-0.57825	4.042811	5.855958	0.015524	1	61	69	81	122	58	49	39	41	61	32
Gss	-0.57365	4.448606	7.613378	0.005794	0.735366	72	39	79	73	63	25	29	58	42	32
Zfp182	-0.57268	3.08216	4.058782	0.043942	1	30	26	40	34	15	9	14	20	20	19
Gprin1	-0.57242	2.467186	4.176447	0.04099	1	71	49	52	46	54	36	45	30	26	25
Wnt4	-0.56945	7.848365	6.107697	0.013459	1	37	27	55	23	17	28	16	9	19	19
Rad52	-0.56746	3.237496	5.6145	0.017812	1	35	22	71	90	58	37	21	33	35	27
Ctbp2	-0.56335	5.561838	13.42947	0.000248	0.247537	91	57	141	122	92	80	51	35	84	38
Rfwd3	-0.56058	4.954923	12.23596	0.000469	0.281521	96	74	115	150	92	76	76	49	59	44
Dvl2	-0.55923	4.235044	6.29199	0.012128	0.9915	39	49	58	73	66	52	31	44	24	16
Gorab	-0.55832	3.613367	5.310238	0.0212	1	49	27	95	48	34	29	33	36	27	19
Cep89	-0.55681	3.761766	5.659972	0.017356	1	25	26	35	44	37	25	14	26	9	22
Ccdc163	-0.55237	2.600531	4.116539	0.042466	1	41	19	43	48	29	17	12	40	17	17
Cbx8	-0.55014	3.410001	4.954611	0.026021	1	21	32	70	43	47	27	17	28	27	23
Plk2	-0.54062	5.148643	5.658133	0.017375	1	43	44	41	49	25	29	23	18	24	25
Ccdc120	-0.53817	4.536996	4.786731	0.02868	1	20	34	29	23	20	14	15	23	15	8
Dmpk	-0.53486	3.93136	5.98142	0.014457	1	79	60	70	89	59	30	32	52	66	32

Dnajc17	-0.53151	4.147079	7.093844	0.007735	0.854323	51	38	70	126	64	32	47	42	45	35
Dnajc19	-0.53059	3.883609	5.347244	0.020755	1	52	61	81	78	71	30	43	55	38	37
Ncapd3	-0.52725	4.129635	6.683951	0.009728	0.941571	59	47	94	113	53	39	38	65	40	30
Sox9	-0.52666	6.660992	8.627414	0.003311	0.581129	207	172	221	217	165	123	117	129	110	107
Epb41l4ao	-0.52467	3.590946	4.996561	0.025398	1	69	32	79	99	68	41	50	43	37	32
Mterf4	-0.52304	3.588643	5.20644	0.022503	1	75	40	114	103	39	51	53	37	38	35
Smpd4	-0.52301	3.815314	4.212132	0.040136	1	50	26	91	60	54	21	31	37	45	29
Smpd2	-0.51669	3.829367	4.801476	0.028435	1	17	39	61	71	34	25	18	15	39	31
Mkrn2	-0.51609	4.041334	5.453979	0.019524	1	116	57	118	103	68	59	73	75	34	36
Asl	-0.50914	3.538609	3.918838	0.047748	1	40	19	70	45	37	36	15	28	28	17
Dhrsx	-0.50747	4.345669	4.83756	0.027846	1	74	55	102	84	77	52	66	46	40	33
Lrrc61	-0.50565	4.398593	5.786412	0.016151	1	60	71	112	152	84	44	63	87	36	51
Gm10516	-0.50232	3.740162	6.42341	0.011263	0.980859	38	56	65	73	60	27	42	43	40	25
Chic2	-0.49305	5.437928	6.575772	0.010338	0.951656	59	76	64	110	71	38	39	52	67	36
Nmd3	-0.49229	4.225998	4.505748	0.033781	1	85	60	124	88	74	50	50	72	34	52
Slc25a15	-0.49159	3.749874	4.284771	0.038455	1	34	64	72	92	45	32	53	28	39	33
Fxyd5	-0.48797	3.222952	4.133769	0.042036	1	113	69	89	100	75	66	61	59	42	47
Iffo1	-0.48709	2.695993	4.286157	0.038424	1	60	49	71	82	57	30	42	43	40	38
Dck	-0.48559	3.516315	3.933968	0.04732	1	33	46	59	43	64	24	49	34	25	21
Ift80	-0.48052	3.917653	4.174823	0.041029	1	65	79	107	86	106	50	62	31	67	62
Parn	-0.48022	4.678136	9.530203	0.002021	0.479868	76	84	127	127	101	60	65	65	71	53
Mpzl2	-0.47648	7.765993	4.030212	0.044692	1	28	29	31	60	21	10	31	12	12	36
Pycrl	-0.4738	4.210852	7.722694	0.005453	0.725022	78	90	141	162	74	78	72	65	58	56
Kcnmb2	-0.47362	2.600111	3.947467	0.046942	1	61	48	90	101	40	45	54	28	51	28
D130009I1	-0.47267	3.078456	4.519563	0.033509	1	71	70	120	115	61	60	34	73	49	47
Bcas2	-0.46609	5.417775	9.02761	0.002659	0.549837	145	171	197	165	159	107	121	103	111	85
Cops7a	-0.4608	5.506749	4.200892	0.040403	1	90	206	273	242	260	89	192	114	158	116
Orai1	-0.45597	3.93964	5.668329	0.017274	1	56	47	83	104	75	44	44	55	57	26
Ankrd34c	-0.45392	4.154921	4.095315	0.043002	1	68	50	66	76	67	32	24	46	42	57
Ccdc191	-0.45158	5.074403	4.420123	0.035517	1	97	67	138	59	51	73	50	38	55	42
Sh3gl1	-0.45131	4.558828	5.608144	0.017877	1	46	58	127	126	79	51	45	73	63	34
Ltbp4	-0.44984	4.629179	4.418702	0.035547	1	66	57	54	53	40	39	33	22	47	31
Mknk1	-0.44481	3.805907	5.033932	0.024855	1	71	60	126	143	95	82	71	50	55	47
Nsmce1	-0.44258	4.381577	4.568988	0.032556	1	104	64	136	171	61	109	79	52	43	46
Rnf2	-0.44028	4.866555	5.938566	0.014813	1	41	75	89	135	85	57	54	39	50	62
B2m	-0.44004	8.136567	5.762054	0.016376	1	1092	1194	1857	3271	1710	1129	1788	1024	894	798
Slc25a33	-0.43966	4.747529	12.45943	0.000416	0.275262	236	238	346	368	203	186	202	185	178	123
Prmt9	-0.43844	4.229428	3.871051	0.049126	1	108	87	123	127	119	81	77	65	73	62
Palld	-0.43587	7.542297	13.60971	0.000225	0.245312	143	122	223	215	199	147	114	99	130	79
Mafk	-0.43552	5.220205	3.967459	0.046388	1	35	38	53	37	50	31	33	30	20	22
Gpank1	-0.43374	3.771309	4.024482	0.044844	1	76	61	77	120	50	52	48	40	51	48
Stat1	-0.4305	4.549727	3.923933	0.047603	1	71	112	148	123	127	94	79	81	63	55
Ctsh	-0.43006	6.29707	5.70912	0.016877	1	75	31	91	60	54	40	45	53	36	23
Chsy1	-0.42804	4.668771	6.405786	0.011375	0.981351	94	65	125	122	63	60	59	61	66	48
Ldlrap1	-0.42689	6.48072	4.169651	0.041154	1	33	23	69	46	46	29	39	24	40	6
Ephb3	-0.42587	7.237128	5.115699	0.02371	1	51	63	73	93	96	59	29	56	42	52
Mical3	-0.4237	5.479113	6.453259	0.011075	0.977017	163	128	320	256	192	124	157	123	144	112
Lsr	-0.42016	6.045309	4.226976	0.039786	1	46	54	64	63	43	50	37	25	32	29
Fzd2	-0.41919	4.403527	4.788195	0.028655	1	86	93	118	152	78	82	82	74	64	36
Car5b	-0.41421	6.201812	4.895966	0.02692	1	40	40	51	65	60	41	35	21	47	21
Ezh2	-0.41257	5.359906	4.894072	0.026949	1	108	67	126	118	107	67	85	67	63	56
F11r	-0.41238	8.490776	5.115639	0.023711	1	57	36	90	92	77	46	31	44	70	32
Smg9	-0.41091	4.219144	4.54837	0.03295	1	105	107	160	165	91	113	76	65	88	59
Plxnb2	-0.41032	7.839856	7.807403	0.005203	0.725022	111	103	149	185	73	69	102	93	77	55
Rnaseh2a	-0.40917	4.176532	4.666322	0.03076	1	100	59	108	120	62	62	52	75	54	43
Acp1	-0.40731	5.671035	6.418024	0.011297	0.980859	224	225	310	289	298	145	234	244	173	93
Ccdc117	-0.40614	3.922212	4.399647	0.035946	1	41	41	103	88	66	36	55	46	49	29
Katna1	-0.40526	5.350541	5.771266	0.01629	1	80	53	68	117	52	49	52	51	46	39
Senp8	-0.40413	4.492666	5.084683	0.024138	1	129	94	171	169	99	50	117	108	93	58
Gnai3	-0.40368	5.516362	4.021486	0.044924	1	77	66	115	122	92	44	60	45	95	57
Mthfd1	-0.40312	4.795534	4.932757	0.026352	1	92	72	142	164	131	90	85	54	81	72
Rps4l	-0.40221	6.382407	7.493263	0.006193	0.759767	149	184	296	365	247	172	194	139	142	141
Rchy1	-0.40209	5.605883	8.941804	0.002787	0.552875	125	117	209	189	119	113	84	83	102	100
Bloc1s5	-0.4016	4.841614	7.373513	0.006619	0.791132	168	134	197	232	142	122	106	141	99	93
Ier5	-0.40089	7.256974	5.452738	0.019538	1	62	62	95	116	95	67	55	60	45	49
Pdcl3	-0.40012	4.706792	4.796448	0.028518	1	82	111	107	132	75	65	110	53	61	45
Ccnt1	-0.39966	3.944341	4.668955	0.030713	1	71	62	56	79	49	36	48	50	43	31
Maz	-0.39928	5.373017	4.23942	0.039496	1	231	196	330	315	236	170	236	146	174	124

Naa30	-0.39907	4.996786	5.190718	0.022708	1	140	129	200	238	186	101	114	132	175	60
Slc35b2	-0.39692	4.321913	4.549802	0.032923	1	63	69	103	146	67	50	63	73	60	40
Gm10419	-0.3962	4.614145	7.040544	0.007968	0.858587	218	184	269	367	205	179	178	161	170	114
Bcl7c	-0.39256	3.691903	4.251408	0.039218	1	76	48	62	76	46	46	34	33	54	34
Gas1	-0.39087	7.210612	4.342168	0.03718	1	40	23	83	50	46	26	44	30	35	21
Oscp1	-0.38379	4.3723	4.179263	0.040921	1	155	118	226	180	139	109	130	94	116	85
Lig1	-0.38246	6.618005	4.370728	0.036561	1	294	248	370	513	219	267	250	194	190	161
Mrpl57	-0.38168	5.317352	8.351547	0.003854	0.616163	218	172	313	359	164	172	142	166	155	148
Mrpl9	-0.37884	4.481942	4.565157	0.032629	1	61	72	92	101	54	48	52	63	58	29
Aasdhppt	-0.37378	4.27038	4.55019	0.032915	1	90	80	132	173	69	75	90	78	68	43
Kdm6a	-0.3733	4.810481	7.777749	0.005289	0.725022	94	79	134	130	79	76	71	73	74	45
Tmem97	-0.37237	4.165034	4.185383	0.040774	1	53	45	103	80	65	30	54	41	57	43
Mmab	-0.37221	4.648632	6.955179	0.008358	0.889442	108	95	128	207	111	92	88	90	95	60
Galnt7	-0.37198	7.472614	4.335372	0.037328	1	85	59	78	78	36	61	39	42	32	47
Rnf111	-0.37081	5.115051	6.777318	0.009232	0.924097	108	96	148	155	108	82	68	75	111	68
Coq5	-0.37067	5.306082	5.364354	0.020552	1	274	207	248	318	131	175	125	172	192	116
Pxk	-0.36921	5.23902	4.29384	0.038251	1	158	135	167	170	129	145	88	76	103	93
Stx16	-0.36814	5.140285	4.100123	0.04288	1	164	105	176	197	154	101	118	111	124	75
Sdf2	-0.36534	6.373994	8.939644	0.00279	0.552875	247	280	393	322	334	243	238	211	231	139
Ccdc97	-0.36531	5.399484	4.651411	0.031028	1	244	155	298	381	161	227	184	147	131	117
Txndc12	-0.36319	5.128982	4.402406	0.035888	1	64	63	99	136	87	72	64	42	56	59
1110059E2	-0.36268	5.184016	4.481009	0.034273	1	109	105	169	171	93	98	90	107	56	73
Tspan14	-0.36179	5.734331	6.851169	0.008858	0.913142	121	115	241	174	180	87	149	132	107	77
Slc15a4	-0.36139	5.103253	6.385985	0.011502	0.987077	131	137	210	238	144	140	150	105	112	65
Cdk5rap2	-0.36041	5.410097	4.961502	0.025918	1	277	177	361	319	208	192	167	188	194	142
Fhdc1	-0.36037	5.795806	7.044243	0.007952	0.858587	94	88	180	178	114	68	102	79	113	67
Zfp639	-0.35751	4.654989	3.957174	0.046672	1	152	146	285	286	130	141	153	136	124	97
Zfp618	-0.35624	5.194805	4.175247	0.041019	1	51	61	65	92	78	51	38	53	51	39
5031439G(	-0.35313	5.244076	8.381469	0.003791	0.610501	209	146	303	258	224	144	155	148	156	150
Shroom2	-0.3494	4.994007	5.562268	0.018352	1	182	117	192	288	171	157	113	119	121	116
Drg2	-0.34819	5.241682	3.893757	0.048466	1	247	227	361	246	169	182	193	220	140	112
Mtmr3	-0.33759	5.118001	5.194631	0.022657	1	148	142	169	209	163	102	115	97	143	106
Ctss	-0.33535	4.506339	3.975784	0.046159	1	123	72	168	151	110	69	120	79	84	67
Nrep	-0.33453	4.669332	4.549181	0.032934	1	142	139	217	234	201	111	140	127	134	116
Secisbp2	-0.33446	4.793957	3.868092	0.049212	1	102	65	114	124	73	58	69	73	72	50
Nabp2	-0.32895	5.186375	5.332095	0.020936	1	151	127	249	190	183	131	148	141	121	74
Kdm5a	-0.3273	7.032047	7.482538	0.00623	0.759767	394	398	538	622	351	357	296	352	311	244
Nxt2	-0.32567	4.620419	4.493772	0.034019	1	104	139	179	223	148	98	106	123	117	92
Gtf3c3	-0.32548	4.988906	4.456222	0.034774	1	185	173	215	261	138	155	98	145	166	98
Tmem80	-0.32344	4.470836	4.237717	0.039535	1	104	103	159	207	106	114	103	77	84	78
Zcchc10	-0.32007	4.780256	5.761817	0.016378	1	172	126	221	234	133	149	106	100	135	108
Cpsf3	-0.31972	5.417239	5.19623	0.022636	1	127	135	241	227	164	103	89	176	133	101
Tnfrsf1a	-0.31886	6.024267	4.523445	0.033433	1	103	109	153	193	124	70	106	105	108	75
Zfp330	-0.31712	4.558038	3.862178	0.049386	1	89	85	131	115	104	71	66	69	97	57
Rgcc	-0.31105	4.299362	4.199675	0.040432	1	137	172	268	237	166	170	152	137	124	91
Chd1	-0.30748	6.571161	5.017998	0.025085	1	267	149	249	283	159	139	159	148	180	134
Bdh1	-0.30487	4.878044	3.920537	0.0477	1	158	150	184	267	112	150	179	94	94	85
Bmpr1a	-0.30398	6.17452	6.052562	0.013886	1	315	294	388	348	209	238	262	196	197	188
Mlst8	-0.30169	4.760339	4.189481	0.040676	1	141	129	147	283	173	147	118	118	128	91
Osbpl5	-0.30056	6.219503	4.379661	0.03637	1	113	82	172	181	163	93	99	83	121	91
Rit1	-0.29828	6.775997	7.991919	0.004699	0.695008	550	528	642	669	456	441	444	430	369	313
Tecpr2	-0.29756	4.854345	4.482163	0.03425	1	198	146	220	313	194	157	141	152	170	118
AU022252	-0.2971	4.923209	4.878427	0.027194	1	205	168	233	314	229	182	178	177	163	103
Rhog	-0.29262	5.549142	3.912444	0.04793	1	96	93	183	147	120	59	113	90	106	74
Creg1	-0.28764	5.79039	6.255934	0.012378	0.9915	320	291	363	362	215	228	259	246	222	145
Trmt6	-0.28625	5.674018	3.859798	0.049456	1	245	140	235	270	152	128	155	134	131	169
Inpp5k	-0.28503	5.074753	4.64701	0.031108	1	186	178	286	279	204	148	191	204	127	122
Ifnar2	-0.28275	5.031999	6.34054	0.011801	0.9915	191	192	275	252	169	157	175	147	140	137
Rpl6	-0.28215	7.242734	5.934981	0.014843	1	354	289	505	430	288	316	247	244	286	210
Hsd17b10	-0.28115	6.081772	5.380829	0.020359	1	278	231	431	361	299	218	244	278	206	174
Rcc2	-0.27951	6.658067	5.355719	0.020654	1	435	347	712	652	472	357	486	322	378	282
Smarcd2	-0.27789	6.908431	5.108372	0.023811	1	165	173	231	241	161	175	127	119	168	97
Grb14	-0.27528	5.342203	5.127638	0.023548	1	268	326	486	438	365	222	315	274	277	237
B3gat3	-0.27461	5.810743	5.149033	0.023259	1	350	430	480	604	361	353	343	331	289	258
Paqr5	-0.27418	7.691805	5.103134	0.023883	1	163	99	195	205	117	114	109	137	101	84
Cttnbp2nl	-0.27252	6.031162	4.651588	0.031025	1	199	170	295	249	150	157	131	179	157	121
2310022B(	-0.26702	6.762892	7.771724	0.005307	0.725022	244	280	415	433	295	220	273	220	263	200
Med28	-0.25887	6.377433	6.291609	0.012131	0.9915	402	375	537	562	393	358	367	341	331	230

Acbd6	-0.25612	5.63852	4.814893	0.028215	1	218	189	354	315	222	166	191	226	182	153
Cox14	-0.25171	6.606255	4.990478	0.025487	1	442	477	605	781	526	464	409	344	444	359
Uba52	-0.24745	6.943722	4.060396	0.0439	1	279	225	489	538	236	274	230	249	267	209
Gnptab	-0.24744	5.728811	4.301115	0.038087	1	142	110	168	178	169	106	107	134	123	84
Timm17b	-0.24672	5.847968	4.327052	0.037511	1	202	145	236	274	197	182	163	156	146	111
Imp3	-0.24565	5.893009	3.845813	0.04987	1	283	203	337	431	231	243	214	213	233	156
Dnttip2	-0.24409	5.969424	4.891723	0.026986	1	241	209	302	336	244	223	207	187	211	136
Crim1	-0.23751	7.946938	4.674338	0.030617	1	143	141	218	243	144	114	148	132	130	114
Dffa	-0.23345	6.229015	4.46039	0.03469	1	551	447	689	759	547	525	452	371	438	378
Etfb	-0.2331	7.314694	4.513545	0.033627	1	641	653	928	1188	604	631	674	621	593	383
Arl4c	-0.23052	5.206006	3.912769	0.047921	1	249	199	276	354	241	192	235	187	173	171
Knop1	-0.21606	7.205628	3.886703	0.04867	1	464	382	604	679	369	350	413	471	329	263
Dad1	-0.2147	7.426766	5.752612	0.016464	1	843	696	1053	1156	716	694	720	616	687	552
Nicn1	-0.21367	6.454046	5.100619	0.023917	1	682	511	940	965	536	580	538	548	529	440
Mllt3	-0.20992	6.292735	4.090417	0.043127	1	461	330	433	538	389	298	364	296	356	282
Ndufa11	-0.2089	8.126606	6.503064	0.010769	0.95735	1584	1469	2320	2110	1414	1350	1526	1259	1331	1089
Arhgap12	-0.20606	6.28923	4.553365	0.032854	1	302	316	464	517	362	330	289	279	299	245
Hip1r	-0.20271	6.413314	3.866511	0.049259	1	249	239	330	393	290	231	225	244	237	176
Chmp3	-0.1999	7.944237	5.52783	0.018716	1	1115	1053	1439	1603	1110	1060	1103	972	913	681
Tmcc3	-0.19481	6.775217	4.109768	0.042636	1	367	334	524	508	375	313	361	285	355	257
Abhd17b	-0.19249	6.796327	4.207342	0.040249	1	597	491	770	744	476	554	516	407	464	359

Table S6. Differentially expressed genes in the TG of miR-183C KO vs age- and sex-matched WT control mice.

	log2FC	logCPM	LR	PValue	FDR	WT1	WT2	WT3	KO1	KO2	KO3
**Upregulated genes**											
Gbp1	7.869934	2.002584	16.04506	6.18527E-05	0.014309931	0	0	0	0	74	30
Olfml2a	6.787865	-0.76982	9.407956	0.002160459	0.167813834	0	0	0	3	12	24
Rnf180	6.639192	-0.96268	6.705907	0.009609406	0.389370954	0	0	0	8	14	10
Col22a1	6.414903	-1.12469	5.712681	0.0168428	0.504721015	0	0	0	1	15	17
1700007K13Rik	6.293373	0.666839	15.7236	7.3304E-05	0.015931404	0	0	0	4	28	0
Slc15a3	6.158839	-0.98298	4.060977	0.043885099	0.757810431	0	0	0	0	33	2
Alox12b	6.157591	-0.48096	9.972233	0.001589187	0.143066707	0	0	0	1	12	14
Kcng3	6.09034	-0.61679	10.05746	0.001517321	0.13833339	0	0	0	3	17	6
Ctcfl	5.983533	-1.47484	5.052458	0.024591037	0.599028227	0	0	0	5	5	9
Cacna1f	5.893107	-1.04329	4.508432	0.033728149	0.67948957	0	0	0	0	0	20
Grem1	5.884219	-1.51615	4.842448	0.027767457	0.62298708	0	0	0	4	7	8
Fam83a	5.746201	7.100875	26.89558	2.1475E-07	0.00015933	0	0	0	0	20	0
9230116N13Rik	5.740471	-0.1862	14.4685	0.000142523	0.025939835	0	0	0	2	9	8
Fxyd4	5.655839	2.86854	21.46584	3.60189E-06	0.0018902	0	0	0	3	7	7
Gm5148	5.606444	0.280327	11.14723	0.000841573	0.094418351	0	0	0	0	10	9
Rd3	5.5858	-0.3944	8.522267	0.003508272	0.225999933	0	0	0	0	10	9
Lrrc50	5.569751	-1.56481	4.193852	0.04057082	0.730176079	0	0	0	5	0	7
4933430I17Rik	5.510571	-1.38852	5.228944	0.022214014	0.574915158	0	0	0	5	8	1
Gm10474	5.50804	-1.18063	4.638119	0.031269255	0.660464347	0	0	0	0	18	3
Oas2	5.407161	1.374948	13.40368	0.000251131	0.039154624	0	0	0	1	14	2
Gdf5	5.394629	-0.68259	6.285903	0.012170204	0.429066966	0	0	0	1	12	4
5830416P10Rik	5.247544	2.309126	16.48403	4.90616E-05	0.012720219	0	0	0	2	12	0
Peg12	5.244477	-0.54079	8.942745	0.002785737	0.193396506	0	0	0	2	13	0
Eppk1	5.24131	5.81139	19.92733	8.04421E-06	0.003393711	0	0	0	0	7	6
Psapl1	5.227975	6.324118	20.08282	7.416E-06	0.00332422	0	0	0	0	12	1
Mia2	5.139752	-0.95323	5.04375	0.02471494	0.599616062	0	0	0	0	17	0
Trp73	5.101105	0.434255	11.00374	0.000909281	0.097477719	0	0	0	0	15	0
Pde6h	5.097348	-1.2257	5.018128	0.025083268	0.602257311	0	0	0	6	0	1
Cldn8	5.096831	-0.57166	4.660882	0.030857338	0.657352962	0	0	0	1	14	0
Grap2	5.060055	0.711122	12.31177	0.00045011	0.060176308	0	0	0	1	7	4
Siglece	5.059273	-1.10611	5.512074	0.018885642	0.532733953	0	0	0	0	13	2
Alox15	5.059089	-0.95485	5.014211	0.025140091	0.602257311	0	0	0	0	16	0
Ltf	5.049794	2.587274	14.65891	0.000128824	0.02431392	0	0	0	4	0	6
Mapk15	5.017104	0.30398	9.884317	0.001666934	0.146990757	0	0	0	1	0	9
5730559C18Rik	4.992312	4.208334	15.96325	6.45841E-05	0.014627289	0	0	0	8	0	2
Leprel1	4.980818	1.942102	14.45606	0.000143467	0.025939835	0	0	0	0	5	6
Hrc	4.934634	-0.28968	5.844201	0.01562847	0.486272706	0	0	0	0	0	10
Gm4013	4.930064	-1.26824	4.970342	0.025785555	0.60700438	0	0	0	0	0	10
Rax	4.923359	-0.87053	4.737164	0.029517686	0.644774134	0	0	0	1	12	0
Snx31	4.916144	3.163254	14.31373	0.000154732	0.027066842	0	0	0	4	6	0
Ccdc88c	4.897153	0.612969	11.72676	0.000616079	0.074890147	0	0	0	2	5	3
Catsper3	4.883525	-1.231	4.418381	0.035553758	0.694145031	0	0	0	0	1	9
Gjb3	4.850484	5.383095	15.27385	9.29952E-05	0.01869986	0	0	0	0	10	0
Fgfr4	4.840589	-0.49446	7.235482	0.007147662	0.337332033	0	0	0	0	13	0
Mxd3	4.819973	1.00324	9.846312	0.001701726	0.148838744	0	0	0	0	12	0
Irx2	4.812347	0.27136	7.730967	0.005428166	0.289802085	0	0	0	6	0	0
S100a7a	4.773562	1.624529	11.22522	0.000806931	0.091862058	0	0	0	0	11	0
Pdilt	4.765891	-0.88889	3.910059	0.047997914	0.789126837	0	0	0	0	13	0
4930420K17Rik	4.720241	-1.29249	5.672537	0.017232515	0.511506439	0	0	0	1	7	2
Slc25a43	4.612011	0.963137	9.44526	0.002116955	0.167681269	0	0	0	0	10	0
Rab25	4.610079	7.428767	12.90581	0.000327563	0.04762064	0	0	0	8	0	0
Mms22l	4.550233	1.841769	9.333376	0.002250174	0.170474424	0	0	0	6	0	0
Kynu	4.53302	0.680336	8.734398	0.003122624	0.205053712	0	0	0	2	6	0
4833423E24Rik	4.510946	-0.3455	6.019666	0.014147321	0.458424345	0	0	0	1	8	0
4833403I15Rik	4.499622	-0.88453	5.574384	0.01822507	0.524940575	0	0	0	4	0	1
Rab44	4.477346	0.4093	7.099326	0.007711295	0.347310694	0	0	0	5	0	0
Gm94	4.470742	-0.72371	5.394588	0.020199298	0.549441328	0	0	0	0	10	0
Stx19	4.45424	1.937262	9.527158	0.002024534	0.164999521	0	0	0	0	0	7
Cldn7	4.429101	7.77474	11.29261	0.000778162	0.089534456	0	0	0	7	0	0
AU023871	4.354059	-0.51462	5.877334	0.015337025	0.481739326	0	0	0	1	0	5
Nphs1	4.318107	0.469835	7.867322	0.005033614	0.282590488	0	0	0	0	8	0

1700123I01Rik	4.310131	0.411622	7.697457	0.005529869	0.289825134	0	0	0	0	8	0
BC016579	4.248399	0.192587	6.299599	0.012076525	0.429066966	0	0	0	0	0	6
Dnajb13	4.241507	-0.49125	5.115528	0.023712649	0.587113195	0	0	0	0	0	6
Hist1h1d	4.217035	-0.17075	5.436502	0.019720123	0.543546322	0	0	0	4	0	0
Vipr1	4.149509	-0.91368	3.934796	0.047296889	0.787260109	0	0	0	0	8	0
Wnt11	4.077359	2.321083	17.62186	2.69473E-05	0.00852643	0	1	0	4	4	10
Dmrta2	4.032369	9.165108	22.53574	2.06271E-06	0.001167925	0	1	0	0	18	1
Msx2	4.020093	-0.4826	4.610319	0.031780119	0.665804326	0	0	0	0	7	0
Ces2e	3.989374	1.481009	7.090615	0.007748867	0.348068118	0	0	0	0	0	5
Cldn4	3.963418	7.532212	8.066055	0.004510222	0.260166043	0	0	0	5	0	0
Ccdc89	3.9524	-0.43387	4.933279	0.026344277	0.611068844	0	1	0	7	0	5
Cd5	3.937935	-0.6402	5.428758	0.019807759	0.544992009	0	0	0	0	1	4
Ap1m2	3.918852	5.007197	7.863139	0.005045274	0.282590488	0	0	0	0	5	0
Ccdc64b	3.918303	4.897028	7.857111	0.005062121	0.282590488	0	0	0	0	5	0
Cdsn	3.850083	3.314134	6.399717	0.011413853	0.421959559	0	0	0	4	0	0
Gdf15	3.824698	-0.2093	5.272981	0.021658891	0.567615952	0	0	2	1	30	4
Npm2	3.814393	-0.40951	4.827114	0.028015492	0.625058265	2	0	0	3	14	13
Sox15	3.750168	3.21234	6.443956	0.011133047	0.41872495	0	0	0	4	0	0
Dpep2	3.698686	-0.91778	3.920462	0.047701779	0.789126837	0	0	0	0	0	4
Gpha2	3.650807	4.490986	31.03839	2.52975E-08	2.86474E-05	0	3	0	5	20	10
Depdc1b	3.642944	-0.74992	4.412259	0.035681558	0.694145031	0	0	0	0	5	0
Pak6	3.631876	4.306533	6.287931	0.012156285	0.429066966	0	0	0	1	3	0
Slc6a3	3.631575	-0.41421	4.742756	0.029421895	0.643587562	0	0	0	0	1	3
Tprg	3.615994	4.514326	6.252635	0.012400871	0.429657248	0	0	0	0	4	0
Pyhin1	3.502774	3.381942	10.53801	0.001169441	0.116489268	0	0	1	5	0	6
Cxcl2	3.46922	0.476634	4.851218	0.02762662	0.621772341	0	0	0	0	4	0
D730005E14Rik	3.467917	-0.24806	4.127551	0.042190356	0.746732391	0	0	0	0	3	1
Aknad1	3.459566	1.507698	4.805807	0.028363981	0.62785949	0	0	0	3	0	0
Rbpjl	3.387994	0.356311	7.283505	0.006959061	0.330532334	1	0	0	2	2	7
Six3	3.384735	-0.63436	4.6967	0.030220586	0.655209118	0	0	1	2	0	8
2310002L13Rik	3.317111	0.938009	4.11775	0.042435476	0.748631171	0	0	0	0	0	3
Mylk3	3.303359	-0.34767	4.585166	0.032249857	0.671071484	0	0	2	0	16	7
Rspo4	3.270167	0.069861	7.742838	0.005392599	0.289603968	7	4	0	14	50	31
Pttg1	3.261209	5.281364	13.48718	0.000240199	0.038282441	25	35	30	14	974	9
Fgd1	3.258146	0.315937	6.731228	0.009473938	0.387530896	0	1	8	16	33	18
Pde6a	3.216481	-0.62002	4.027705	0.044758783	0.764309503	0	0	2	5	11	1
1700034H15Rik	3.206482	-0.27579	3.931118	0.047400411	0.787455204	0	2	4	10	39	1
Bnipl	3.165585	6.266878	10.42218	0.001245109	0.122327719	0	0	1	4	6	0
Fosb	3.093725	1.927958	8.490513	0.003570032	0.227837959	1	2	1	6	26	1
Glra1	3.063131	0.302619	6.30883	0.012013811	0.429066966	1	2	14	39	26	24
Sectm1b	2.934582	-0.44825	4.123857	0.042282567	0.747536325	0	2	1	0	17	8
Gh	2.916025	4.225626	35.93532	2.03977E-09	3.83129E-06	132	76	98	219	415	1187
Mlf1ip	2.815949	1.198662	8.023622	0.004617115	0.263506244	1	0	5	4	30	7
Manba	2.730368	-0.25872	4.362008	0.036748967	0.700860741	3	1	2	6	21	9
Gprc5a	2.639792	7.381306	9.757433	0.001785991	0.151577456	0	0	2	0	8	4
Il1r2	2.620417	0.188422	4.369062	0.036597134	0.700170206	0	0	2	1	13	0
A630055G03Rik	2.605345	1.405272	11.87758	0.000568136	0.071485521	3	0	5	9	12	17
Sh3bp2	2.535543	3.445032	27.12662	1.90556E-07	0.000151852	5	15	2	11	86	21
A430110N23Rik	2.534471	0.074413	5.049998	0.02462596	0.599028227	6	0	7	13	28	20
1700029I15Rik	2.505629	0.256491	7.389469	0.006560685	0.31882955	4	4	7	22	37	5
Pomc	2.46446	2.760494	35.84477	2.13681E-09	3.83129E-06	39	64	40	90	272	279
Ovol2	2.455364	3.458312	12.31104	0.000450287	0.060176308	0	4	0	5	9	5
Fetub	2.375815	2.061402	4.252123	0.039201296	0.718445555	0	0	1	0	6	0
Mrgpra3	2.370023	2.367528	25.39332	4.67535E-07	0.000314359	37	28	50	69	288	154
Bspry	2.366256	5.848952	9.47851	0.002078928	0.166903784	0	0	3	0	6	8
Chek2	2.352683	3.280069	17.46105	2.93255E-05	0.009013831	8	0	1	6	28	7
Gsta3	2.323216	5.651377	20.55924	5.78142E-06	0.00276429	0	0	9	15	15	6
Rhbg	2.318832	3.794285	6.354809	0.011706354	0.427470411	0	2	0	0	10	0
Areg	2.268666	2.016043	6.61554	0.01010927	0.400833293	0	3	0	4	9	0
Tmprss2	2.267145	5.349268	6.212597	0.012684443	0.433666095	2	0	0	0	0	9
Gm12216	2.262465	1.375637	10.63665	0.001108684	0.111992737	0	12	7	13	29	29
Gdnf	2.237524	1.354751	6.364049	0.011645552	0.426858101	0	4	0	2	14	2
Krt13	2.237026	9.540774	6.236823	0.012512075	0.431425957	1	1	0	0	9	0
Sytl5	2.194201	1.222454	6.980174	0.008241758	0.360306545	0	0	6	4	15	5

Trp63	2.134504	5.399082	7.323745	0.006804945	0.326567584	0	3	0	0	9	3
Chaf1b	2.134297	1.837015	9.875349	0.001675078	0.147106033	3	2	6	2	14	25
Sox7	2.128715	2.282923	4.662757	0.030823665	0.657286399	0	0	2	0	9	0
Ska1	2.115163	2.158094	4.90674	0.026752086	0.614298696	0	0	4	2	15	0
Grhl2	2.025769	5.037748	20.13768	7.20629E-06	0.003298949	0	12	3	0	42	13
Thap6	2.006841	1.676778	8.379014	0.003795784	0.236040708	6	2	6	7	14	24
Ppl	2.003816	9.514417	20.584	5.70712E-06	0.00276429	0	10	2	9	25	6
Pax6	2.002671	7.376403	15.96391	6.45615E-05	0.014627289	0	7	2	4	11	15
Glis3	1.951815	0.916201	5.122874	0.023612486	0.586182792	4	4	6	19	10	5
4930594C11Rik	1.937642	1.37232	5.014722	0.025132659	0.602257311	0	7	0	3	12	8
N4bp2	1.934124	2.563831	10.30335	0.001327891	0.12641995	3	26	1	10	55	34
Aspm	1.887861	4.460865	9.399465	0.002170487	0.167900698	7	2	0	4	27	0
Prr15l	1.878842	1.854081	6.21414	0.012673388	0.433666095	2	11	7	1	18	43
D2Ertd750e	1.832044	2.569697	4.877959	0.027201768	0.620371191	0	0	4	0	8	5
Arsj	1.794172	1.814089	7.651246	0.005673313	0.294405785	4	5	5	4	18	19
Egfl6	1.785481	2.272764	8.28047	0.004007375	0.2431336	16	8	2	14	31	28
Tgm1	1.767923	2.181209	4.769112	0.028974765	0.637444832	2	2	2	5	0	10
Mcm10	1.752039	2.108349	4.491675	0.034060293	0.683773541	7	0	1	9	11	1
Nell1	1.745886	0.160218	3.910431	0.047987297	0.789126837	12	4	14	17	31	30
Cd40	1.745401	2.121769	6.105317	0.013477574	0.446287344	0	9	0	3	15	8
Dgkk	1.740272	2.509731	17.34675	3.1143E-05	0.00943765	61	39	80	79	245	171
Stap2	1.732491	5.298796	8.07953	0.004476806	0.259630595	5	0	8	2	41	0
Ascc2	1.723974	1.982671	4.816898	0.028182023	0.625442919	0	18	2	6	33	18
Dlk1	1.723633	1.704297	9.298501	0.002293414	0.172057298	22	21	35	46	114	45
Marveld2	1.713564	4.177134	10.63922	0.001107142	0.111992737	0	8	29	15	74	16
Tstd1	1.702091	2.313583	13.24988	0.000272597	0.041597214	29	42	41	54	132	102
Sh3tc1	1.686007	1.24804	5.237207	0.022108747	0.574915158	5	0	4	6	13	5
Tspan1	1.673633	3.978733	4.647761	0.031094084	0.658484554	0	0	4	4	7	0
Gldc	1.625157	3.610627	5.012281	0.025168127	0.602257311	2	1	10	14	13	1
Tm9sf1	1.615774	2.074356	12.8576	0.000336111	0.047961082	17	29	16	29	87	40
Upp1	1.612974	5.135118	21.98574	2.74684E-06	0.001477527	15	12	20	28	28	51
Ascl2	1.600268	2.527787	7.388427	0.006564486	0.31882955	3	2	7	2	20	10
Slc1a1	1.588348	5.941951	47.25489	6.23303E-12	2.6822E-08	68	54	71	57	321	134
Rasgrf1	1.57694	5.639648	71.75226	2.43984E-17	2.62478E-13	128	151	128	223	483	244
Serpinb5	1.576303	7.483989	11.82311	0.000585002	0.072756617	0	7	6	2	14	16
Sh3rf2	1.567639	4.438681	16.82314	4.103E-05	0.011615802	7	9	11	8	49	12
Gng4	1.563528	3.297082	26.28244	2.94958E-07	0.00020472	96	142	114	185	378	236
Wnt10a	1.561609	4.269115	8.950655	0.002773701	0.193396506	13	0	0	6	15	11
2610018G03Rik	1.559135	2.354186	4.909268	0.026712955	0.61417846	0	10	6	3	34	7
D3Bwg0562e	1.541863	2.647607	17.65493	2.64827E-05	0.00852643	56	83	84	88	270	171
Fanci	1.540336	1.294534	4.742081	0.029433452	0.643587562	8	8	8	17	8	22
Shq1	1.522387	1.839002	6.867598	0.008777255	0.375450142	12	6	22	21	58	15
Syce2	1.517985	1.964102	6.80728	0.009078693	0.380033386	0	11	8	8	19	16
Chmp4c	1.487582	5.342557	5.835472	0.015706193	0.486937254	0	2	5	0	17	0
Zfp750	1.482275	6.484439	7.208031	0.007257808	0.338321803	1	8	0	4	13	4
Slc7a3	1.481856	2.072926	6.154802	0.013105581	0.440389035	5	11	39	31	63	22
C1qtnf2	1.475483	2.69654	9.742238	0.001800815	0.151946448	12	16	40	23	109	32
Zfp296	1.466733	3.21702	5.915654	0.015006929	0.476941025	0	4	9	7	21	2
Zmynd17	1.459425	0.58266	4.545012	0.03301479	0.67459281	3	8	9	12	14	14
Fam83b	1.434617	4.250718	3.857627	0.049520324	0.798973908	0	5	0	0	12	0
Cpz	1.415583	2.513507	5.063814	0.024430413	0.597057701	9	0	15	16	12	17
Isg20	1.409713	1.464862	4.930661	0.026384224	0.611068844	18	24	14	5	64	65
Trpv4	1.403617	3.424873	9.888882	0.001662803	0.146990757	0	13	14	11	28	18
Slc25a1	1.396849	7.409823	77.1076	1.61893E-18	3.48328E-14	1371	1550	1387	1637	4463	3049
St6galnac1	1.393744	7.789976	9.93149	0.00162475	0.145054412	41	22	26	51	19	88
Cbln1	1.391773	5.396292	15.74309	7.25525E-05	0.015931404	58	18	31	61	60	83
Zdbf2	1.388932	2.411139	6.463384	0.011011972	0.418402432	80	45	74	90	146	158
Idi1	1.386108	1.815518	5.049058	0.024639337	0.599028227	8	41	34	29	105	45
Nox4	1.379541	1.504443	5.842953	0.015639557	0.486272706	8	8	4	3	22	20
B4galnt1	1.373513	5.016262	44.8545	2.12232E-11	7.61065E-08	271	375	360	383	1042	665
1190003J15Rik	1.372947	7.373791	7.196271	0.007305524	0.339493834	0	11	0	0	18	6
Lgals7	1.365402	5.303457	24.65655	6.85115E-07	0.000446695	24	32	22	23	98	48
Lpcat1	1.357983	1.107855	4.106354	0.042722376	0.750131423	16	32	19	46	51	20
Arhgef5	1.354759	6.267621	22.72148	1.87263E-06	0.001088961	13	16	28	30	72	16

Plk1	1.348508	4.543195	7.845042	0.005096027	0.283323327	7	8	14	11	47	6
A830093I24Rik	1.33414	1.721194	5.532509	0.018666324	0.530565321	23	24	11	37	42	25
Grhl3	1.333447	3.2017	9.201203	0.002418562	0.1771011	25	10	6	19	27	34
F2rl1	1.331704	4.405547	7.524109	0.006087865	0.306759938	8	19	8	8	67	7
Grb7	1.32687	7.158763	6.435505	0.011186137	0.41872495	6	5	0	3	13	7
Arhgef15	1.319182	1.787736	7.164983	0.007434041	0.340386324	48	48	33	49	105	99
Gp1bb	1.315928	3.521383	13.86172	0.000196766	0.033335593	24	54	77	46	192	87
Klhl36	1.313121	2.121166	4.7094	0.029998126	0.652776571	9	30	4	16	47	24
Nckap5	1.300734	1.259075	5.588325	0.018080581	0.522878739	14	14	23	19	57	27
Prdm9	1.297951	2.513529	6.829656	0.008965654	0.378796703	6	5	20	9	29	24
1810010H24Rik	1.290596	1.880685	9.380823	0.002192671	0.168124366	18	41	28	35	65	62
BC060267	1.286002	1.039005	4.127069	0.042202388	0.746732391	14	16	30	18	85	25
Slc16a12	1.268432	4.06834	14.36534	0.000150548	0.026770179	14	32	20	37	43	37
Tcfap2a	1.266121	5.69682	19.77117	8.72894E-06	0.003611766	12	17	21	9	63	29
Spag1	1.262068	1.527354	5.486976	0.019158657	0.533960698	14	12	17	23	41	15
Smpdl3b	1.261178	3.125864	4.209738	0.04019255	0.729159283	12	15	2	1	55	14
Nova1	1.243484	2.026995	6.163845	0.013038748	0.440389035	45	59	61	75	158	68
Serpinc1	1.240495	1.607424	4.513364	0.033631041	0.67948957	3	25	18	13	54	24
Rrm2	1.238654	3.364081	4.041442	0.044395864	0.761739563	6	2	1	2	10	6
Hook2	1.22974	4.446116	8.967739	0.002747884	0.193396506	8	25	16	12	21	55
Ybey	1.205687	2.229137	6.464705	0.011003794	0.418402432	24	31	23	27	95	30
Ms4a6c	1.197252	2.029484	3.877347	0.048941826	0.793588094	8	54	55	38	75	88
Sv2c	1.18594	5.891348	6.077751	0.013689492	0.44968413	393	1789	436	686	3201	1249
Ccdc14	1.176649	1.829349	4.653245	0.030994893	0.658325037	24	15	19	12	33	61
A030009H04Rik	1.163646	3.402616	6.762101	0.009311409	0.384537952	79	217	110	120	318	281
Sh3gl3	1.161143	2.76216	6.872555	0.008752941	0.375155914	89	106	86	114	284	103
Zfp51	1.154742	2.365867	8.095654	0.004437153	0.258638786	20	25	18	22	64	29
2210020M01Rik	1.15337	2.853937	8.295692	0.003973923	0.242906061	17	24	52	37	83	41
Nova2	1.153359	2.975271	12.66628	0.000372309	0.052016864	82	108	147	102	346	172
Nebl	1.151326	2.819688	9.003891	0.002694053	0.19130447	23	52	41	27	74	103
Gm13157	1.148568	2.315489	6.217495	0.012649397	0.433666095	15	8	19	11	45	23
Nup43	1.132675	2.139256	4.416247	0.035598248	0.694145031	21	10	32	9	91	30
D19Ertd652e	1.130802	3.063817	6.406683	0.011369158	0.421756554	21	12	42	14	61	62
Ckap2l	1.128555	5.431048	6.276083	0.01223783	0.429066966	16	2	9	13	32	4
Mttp	1.121834	2.420429	4.55966	0.032733575	0.671717756	12	17	36	14	36	61
Prim2	1.115945	1.472761	4.071247	0.043619063	0.755034398	8	10	14	6	31	22
Ercc8	1.106331	2.132201	5.969105	0.014558667	0.468227611	26	26	33	14	100	50
Rnf165	1.106058	2.151821	5.730296	0.016674676	0.501779481	38	80	76	69	156	98
Sdcbp2	1.097761	4.920172	5.184899	0.022784001	0.579064072	1	14	6	7	5	22
Cmtm7	1.090236	2.182267	5.771737	0.016285933	0.494228684	25	38	25	20	112	35
Gpt	1.069439	4.954014	9.547567	0.002002147	0.164420552	39	22	7	28	51	34
1500011B03Rik	1.066919	4.188761	3.896216	0.048394999	0.790161592	91	437	81	248	270	362
Mthfd2l	1.064609	1.732477	4.890276	0.027008365	0.61820423	15	36	25	24	58	43
Cacna2d2	1.062124	6.378794	17.71396	2.56735E-05	0.008498322	1202	678	1554	876	3798	1580
Rgs19	1.058324	2.286571	4.519147	0.033517546	0.67948957	25	60	61	37	147	71
Scin	1.057164	4.890441	6.191424	0.012837088	0.43565108	8	7	10	15	14	11
Airn	1.048757	2.02078	5.383814	0.020324399	0.552146168	15	16	36	24	52	32
Klhl8	1.048649	3.874682	10.76905	0.001032117	0.105246569	142	125	112	122	358	166
Arhgef11	1.037635	2.153445	4.974168	0.025728572	0.606991182	15	55	59	44	69	81
BC030867	1.032949	1.736238	4.774109	0.028890799	0.636248137	18	26	16	28	40	23
Prdm8	1.028252	2.045593	6.772587	0.009256857	0.384537952	64	57	63	57	148	97
Cog1	1.018708	2.406807	3.929026	0.04745942	0.787455204	22	47	93	37	149	87
Lama3	1.017547	4.954276	6.748782	0.009381172	0.386676811	10	5	15	5	17	27
Setd4	1.012559	1.966719	4.310691	0.037873569	0.707367806	17	16	39	25	61	29
Papss2	1.01203	5.34925	15.52747	8.1315E-05	0.016833925	18	27	47	20	73	58
Dak	1.00915	2.128999	5.251935	0.021922386	0.573125212	14	22	45	23	50	53
Fam33a	1.00602	2.416106	5.554499	0.018433248	0.528108883	21	33	30	25	42	61
Mpv17l	1.004846	2.306796	5.782517	0.016186342	0.493649021	55	72	67	76	94	110
Pcca	0.990759	3.254677	11.57628	0.000667984	0.078537405	114	91	79	68	275	142
Pxmp2	0.990127	2.552442	4.081994	0.043342476	0.752617461	32	76	89	61	134	105
Rtp4	0.983428	3.728346	5.692732	0.017035317	0.509072051	42	38	90	34	161	93
Nck2	0.972307	4.607676	14.63405	0.000130535	0.024422481	51	36	57	35	118	80
Hnrnpul1	0.96636	2.773245	4.292946	0.038270805	0.710000942	7	38	50	19	66	64
Plcg2	0.956826	3.435931	7.223225	0.007196629	0.338084448	37	17	35	19	58	64

Rpl29	0.955265	5.285653	4.256455	0.039101439	0.717226397	75	152	89	68	88	300
Tmem154	0.953468	5.94366	11.74883	0.000608815	0.074427622	7	36	36	18	53	51
Mki67	0.950079	5.467182	6.262396	0.012332729	0.429066966	8	8	17	10	29	13
Gbp3	0.947176	2.577935	4.99534	0.025415667	0.603252529	44	68	90	39	188	107
Chst15	0.942045	5.487533	28.35296	1.01092E-07	8.36578E-05	284	291	276	183	752	456
Irx3	0.924005	4.748533	5.041095	0.024752836	0.599616062	18	7	17	18	42	5
Col4a5	0.921798	3.437077	6.387807	0.011490693	0.424071615	10	14	26	8	42	29
Cx3cl1	0.920696	4.615334	11.76339	0.000604072	0.074269844	55	56	95	46	220	72
Trim14	0.916737	3.877594	5.487065	0.019157679	0.533960698	32	9	44	20	92	28
Cd109	0.915454	5.554388	5.181967	0.022822473	0.579064072	7	3	14	7	21	9
AW555464	0.915274	7.422286	60.49041	7.39378E-15	5.30282E-11	1015	990	1207	853	2588	1522
Mccc2	0.91336	2.411331	4.080746	0.043374496	0.752617461	37	35	28	22	43	82
A930013F10Rik	0.910628	4.489716	9.45483	0.002105939	0.167681269	87	70	145	91	253	115
Lrch2	0.909468	2.984243	7.36243	0.006660071	0.32129615	85	41	76	62	144	97
Rad18	0.901194	2.206533	3.893401	0.048476171	0.790161592	26	36	35	31	63	46
Arhgef16	0.897089	4.448852	7.418933	0.006454107	0.317046935	19	26	15	13	66	17
Pld2	0.885757	3.044488	6.977135	0.008255769	0.360306545	40	34	32	27	82	52
Slc22a21	0.879361	3.077283	5.022639	0.02501801	0.602257311	25	45	16	22	87	26
Rfc5	0.872001	4.242399	9.516959	0.002035816	0.165292923	26	51	56	41	66	77
Miip	0.869587	2.33577	4.47623	0.034369456	0.686567536	31	42	30	32	95	28
Tmem220	0.857757	2.495274	4.797228	0.028505571	0.630345175	20	51	55	31	88	63
Myadm	0.852915	5.721288	20.00361	7.72959E-06	0.003393711	578	569	837	524	1740	716
Ppm1f	0.852244	4.533228	10.8321	0.000997554	0.102886204	312	323	343	242	553	593
Ints2	0.846277	3.894488	8.266691	0.004037903	0.2431336	57	88	123	68	199	121
Tspo	0.84573	6.903923	37.29543	1.01522E-09	2.18436E-06	205	214	206	165	460	285
Rpl10	0.845204	6.381129	18.18691	2.00251E-05	0.006732173	207	404	287	204	587	503
Samd12	0.842632	2.607414	4.996243	0.025402401	0.603252529	40	88	52	44	128	88
Glrx	0.842335	4.489313	9.009717	0.00268548	0.19130447	185	340	287	214	538	395
Lrrc3	0.840543	3.09877	5.915694	0.015006591	0.476941025	169	90	106	112	206	189
Chst10	0.839672	3.943192	7.32095	0.006815538	0.326567584	178	120	190	105	382	249
Rgs8	0.837429	4.081861	13.73185	0.000210849	0.034552777	240	294	327	189	627	450
Cartpt	0.834007	3.634704	5.496893	0.01905029	0.533051164	187	251	185	117	680	210
Pde1c	0.831986	5.804368	29.21946	6.46267E-08	6.04569E-05	796	1028	1012	621	1983	1530
Bmp3	0.830546	5.605117	10.0925	0.00148874	0.13833339	63	35	40	40	112	50
Bcl11a	0.82422	2.906164	5.404027	0.020090343	0.549185963	37	41	46	24	134	37
Rbmx2	0.82334	3.207649	5.141052	0.023366479	0.58316509	45	127	120	100	167	112
Mvd	0.817871	6.461316	16.21399	5.65749E-05	0.013525172	809	713	636	645	1802	667
Slc4a3	0.81632	4.861476	6.801911	0.009106037	0.380437865	366	450	551	335	850	721
Lama5	0.808471	4.361399	4.420516	0.035509297	0.694145031	4	26	40	14	65	24
Spon2	0.805934	5.542064	4.262309	0.038966915	0.71597962	14	6	19	4	17	34
Mto1	0.795836	3.081852	4.320322	0.037659788	0.706159831	58	118	78	65	179	109
Parp12	0.790932	4.650769	11.78175	0.000598142	0.073963368	69	70	63	44	166	83
1700017B05Rik	0.78884	3.702103	6.283706	0.012185301	0.429066966	34	69	87	39	149	84
Gng12	0.788021	6.892978	33.3683	7.62569E-09	1.17196E-05	454	394	443	361	920	510
Reep6	0.785876	3.387856	5.680441	0.017155056	0.511230184	42	21	37	21	90	37
Tcf19	0.78316	3.047722	5.019163	0.025068288	0.602257311	22	35	65	27	79	62
BC064078	0.778716	5.35466	5.095249	0.023991467	0.59129486	20	6	14	9	27	20
Npnt	0.776712	6.769446	9.772884	0.001771044	0.151577456	31	46	35	33	56	60
Vat1	0.775364	6.271668	28.95221	7.41861E-08	6.65078E-05	536	685	627	550	1216	700
Oasl1	0.773476	6.303554	5.495951	0.019060556	0.533051164	14	21	19	8	32	35
Gm14446	0.771924	8.330843	4.954104	0.026028816	0.608734787	0	27	13	12	23	19
Itprip	0.770743	2.873143	3.983176	0.045956845	0.778588573	18	20	28	24	27	32
Zfyve26	0.76864	2.920812	4.43563	0.035196232	0.691683889	40	38	74	36	144	41
N4bp2l1	0.764521	4.551424	6.279657	0.012213175	0.429066966	92	122	150	131	229	101
Antxr2	0.764308	3.131643	4.394584	0.036053248	0.698343917	89	126	197	83	417	116
Apobec1	0.753386	2.73689	4.301913	0.038069521	0.709286246	51	54	37	22	129	61
2610017I09Rik	0.750372	3.779177	3.851545	0.049700181	0.799812338	11	24	8	10	31	18
Rbm3	0.75015	3.947261	9.573459	0.001974104	0.162738806	64	92	90	59	190	91
Piga	0.748173	5.92343	8.907142	0.002840575	0.19402479	36	34	39	43	72	28
Celf6	0.745519	4.249156	10.54927	0.001162343	0.116320796	268	344	393	198	757	459
Nt5dc2	0.739714	3.066934	4.202431	0.040366076	0.730176079	29	29	26	33	41	30
Gap43	0.739435	8.27836	16.68942	4.40259E-05	0.011990654	5378	6328	4584	4525	10675	6268
Gdpd3	0.735224	4.932943	10.90118	0.000961029	0.100789978	91	131	101	66	268	121
D730040F13Rik	0.734284	3.22494	4.622915	0.031547583	0.662729393	51	69	97	28	167	117

Nptx2	0.731622	4.058258	9.488509	0.002067628	0.16661832	279	290	313	174	634	419
Wnt4	0.72951	7.880735	19.53526	9.87597E-06	0.00386348	104	97	120	80	247	110
Psen2	0.722344	6.510016	18.99416	1.31119E-05	0.004864062	1145	1457	1469	1037	2431	1799
Irak4	0.722046	5.307032	7.996266	0.004687392	0.266809336	84	60	76	33	218	75
Trp53bp2	0.721871	4.439118	4.783917	0.028726703	0.634582891	37	102	163	54	193	157
Impact	0.72043	5.46848	20.28968	6.65587E-06	0.00311321	663	599	616	399	1293	873
Acta2	0.714596	3.492481	4.338067	0.037269208	0.704024823	122	124	179	68	182	305
Uhrf1bp1	0.713073	3.391685	6.199673	0.01277739	0.43499734	81	69	98	52	159	121
Islr2	0.712547	3.953895	6.072701	0.013728686	0.450284156	284	273	268	228	620	249
Mobkl2a	0.707911	4.186513	7.098264	0.007715863	0.347310694	215	185	221	154	443	232
Rbp1	0.707285	6.960831	12.02597	0.000524644	0.067594282	283	356	326	298	642	283
Ldlr	0.706982	8.620816	30.05182	4.20654E-08	4.30991E-05	1957	1891	2028	1420	4224	2223
Star	0.706285	2.840877	4.373925	0.036492847	0.700170206	68	63	98	44	176	96
Nradd	0.705719	3.668164	6.375391	0.011571358	0.42523009	64	84	76	68	151	68
Stard4	0.700763	6.083544	13.40585	0.000250841	0.039154624	517	674	467	423	1211	561
Fcer1g	0.699439	2.853699	4.508918	0.033718571	0.67948957	48	82	111	51	157	108
Capn5	0.698931	7.599065	29.93501	4.46772E-08	4.36943E-05	971	1148	942	752	1863	1337
Itga3	0.697675	6.558744	26.93471	2.10446E-07	0.00015933	322	438	396	254	751	515
Ceacam1	0.696774	7.023366	5.868879	0.015410862	0.482847354	60	82	72	48	112	113
Lgals3	0.693414	9.399402	11.12504	0.0008517	0.094418351	67	137	134	59	191	190
Rasgef1b	0.692856	5.456988	12.03617	0.00052178	0.067594282	158	172	129	111	258	219
Emid1	0.692436	3.425706	4.171303	0.04111417	0.735713998	205	167	198	123	492	181
Hmgb2	0.69156	5.878642	5.090474	0.024057621	0.591685293	32	66	105	28	185	75
A830080D01Rik	0.689692	3.48076	6.539787	0.0105488	0.407482923	98	95	157	94	219	131
Rpl15	0.687561	4.682677	7.231139	0.007164975	0.337333936	138	249	225	130	451	234
Abhd13	0.683757	4.625025	8.705659	0.003172237	0.206829872	97	163	178	110	300	153
Ina	0.680777	4.323007	9.428021	0.002136948	0.167681269	328	343	418	269	666	450
Lxn	0.677944	7.210063	14.9119	0.00011265	0.021640841	1875	1871	1872	1034	3473	2948
Hunk	0.675766	3.907793	5.482286	0.019210125	0.534240733	51	50	71	29	123	79
Adcy6	0.673935	4.075444	4.324876	0.037559152	0.706159831	90	148	201	89	374	134
Zfp236	0.669827	3.729347	5.304953	0.021264868	0.562082181	102	173	108	80	258	162
Dennd4c	0.668021	3.323911	4.41444	0.035635965	0.694145031	37	26	62	27	83	52
Tor3a	0.658175	4.833395	5.497886	0.019039472	0.533051164	33	50	75	46	74	68
Hipk2	0.654776	3.418246	4.934193	0.026330359	0.611068844	43	65	72	27	145	72
Tap1	0.654601	5.115469	5.33251	0.02093122	0.558476062	80	105	140	38	192	200
Arf2	0.653501	5.591621	13.72179	0.00021198	0.034552777	275	277	304	187	484	406
Ptplad2	0.652726	5.126143	8.920435	0.002819974	0.19402479	51	52	61	43	92	69
Iah1	0.646335	3.102245	3.918194	0.047766168	0.789126837	59	63	41	31	110	72
C530028O21Rik	0.64604	3.554894	6.438925	0.011164622	0.41872495	164	185	221	130	387	210
Nrn1l	0.643825	3.582528	4.995228	0.025417311	0.603252529	153	259	245	185	389	213
Heca	0.641217	4.87073	5.442737	0.019649861	0.542873892	58	168	128	68	268	125
Dgat2	0.640896	4.643502	7.703504	0.005511372	0.289825134	65	94	83	55	134	110
Disp2	0.637441	4.98243	6.839254	0.008917607	0.378444265	568	626	557	304	955	972
Ifitm1	0.630945	8.806115	6.928335	0.008484063	0.368030452	48	64	60	44	113	57
Ptpn9	0.629268	4.129905	6.060572	0.013823292	0.452009043	118	210	234	115	347	239
Cebpa	0.627827	4.667934	6.126747	0.013315162	0.442112709	81	87	43	36	145	94
Coq10b	0.625451	4.390667	3.988085	0.045823129	0.777547661	59	88	102	78	139	72
Insig2	0.624959	4.72272	8.05487	0.00453815	0.261077128	168	203	142	104	336	213
Lrig3	0.624849	5.135306	6.666812	0.009822476	0.395028775	75	50	79	53	150	55
Eif2ak2	0.619441	4.310079	5.549179	0.018489354	0.529011879	115	133	113	62	168	214
Smpd4	0.618491	3.535677	4.867638	0.027364928	0.62042549	58	90	102	47	124	133
Haus4	0.617161	3.100456	4.231214	0.039687046	0.723036818	82	100	55	55	154	87
Mreg	0.61391	7.050158	13.26778	0.000270006	0.041496083	100	80	104	70	181	100
Grik5	0.612043	3.564292	4.566054	0.032611598	0.671455635	111	162	197	98	255	216
Rgs4	0.605578	9.120435	16.48628	4.90034E-05	0.012720219	8862	11112	11663	7292	17034	13369
Mt2	0.601791	9.724791	8.264475	0.004042836	0.2431336	1714	1906	1852	1557	1833	2535
Fnbp1l	0.600289	5.599456	8.341358	0.003875263	0.239881444	132	277	315	144	448	294
Etaa1	0.598555	3.752855	6.143284	0.013191212	0.441083167	91	77	77	55	168	83
AU019823	0.598002	4.842278	5.358783	0.020618163	0.553142638	119	147	158	148	201	114
Braf	0.592707	6.992482	19.95016	7.94874E-06	0.003393711	957	1021	1165	612	1891	1374
Tbc1d2b	0.591366	4.604367	6.442642	0.011141283	0.41872495	128	116	134	83	177	186
Prdm12	0.590831	3.9743	5.40441	0.020085934	0.549185963	240	332	316	183	480	402
Prrx1	0.588297	5.123936	7.291611	0.006927732	0.330503497	67	81	53	35	146	73
Ifi35	0.587894	5.162609	8.922505	0.002816779	0.19402479	185	198	164	136	252	245

Lypd2	0.587386	11.53472	4.869422	0.027336655	0.62042549	70	20	33	26	86	43
Slc44a2	0.586228	6.632697	17.63151	2.68109E-05	0.00852643	719	921	884	480	1461	1142
BC013529	0.584147	5.151577	6.195098	0.01281046	0.435434221	175	276	180	160	416	181
Dcaf12l1	0.583513	4.187528	6.433658	0.011197774	0.41872495	174	226	300	125	457	289
Aldh16a1	0.582408	3.640759	3.944867	0.047014571	0.785443766	72	72	66	40	164	65
Nsdhl	0.582304	6.304792	14.95598	0.000110049	0.021331642	463	746	633	411	1138	665
Acat2	0.581488	7.038832	12.13464	0.000494938	0.065331798	1635	1520	1231	933	2807	1676
Thbs4	0.581111	5.984791	10.21255	0.001394882	0.131463717	98	123	104	87	161	129
Ndn	0.580508	6.311546	13.55741	0.000231377	0.037151488	1042	1187	1186	711	1808	1547
Zfp276	0.576468	3.488038	4.714152	0.029915313	0.652135632	137	120	117	75	220	161
Zfp593	0.573013	5.548697	12.8303	0.000341051	0.047961082	201	208	206	107	378	272
Slc19a1	0.571985	4.183882	4.482898	0.034235626	0.685222067	291	244	337	250	453	278
Zfand2b	0.568225	4.725754	6.765968	0.009291251	0.384537952	241	243	254	167	363	326
Wfdc2	0.566714	6.407327	7.574446	0.005920156	0.300294309	74	66	76	66	112	70
Snx30	0.565772	4.493714	5.308735	0.021218756	0.5615532	198	214	149	157	265	206
Prkce	0.563963	8.399661	15.08192	0.000102945	0.02013601	5837	6247	6501	4095	10769	7156
Atp2b4	0.563277	5.475423	8.464495	0.003621459	0.229174424	560	597	589	418	967	646
Fam83f	0.562072	4.505266	5.205079	0.02252099	0.578926676	54	94	101	56	155	84
A3galt2	0.560257	4.257566	7.768299	0.005317109	0.287444516	328	390	370	217	683	418
Rgs2	0.559	5.670061	8.894757	0.002859907	0.194727056	689	709	616	522	1051	718
Tctex1d2	0.553494	3.736238	4.311346	0.037859005	0.707367806	96	75	104	42	155	138
Nkap	0.549996	5.061553	7.924061	0.004878165	0.27505833	243	339	297	226	437	321
Dlg3	0.549049	6.098737	12.4077	0.000427566	0.058224773	191	204	179	118	290	261
Plxna3	0.542116	3.799569	3.983805	0.045939687	0.778588573	107	127	117	70	244	113
Fam160a2	0.541779	5.270316	10.57514	0.001146185	0.115239833	300	309	380	193	641	357
Plk2	0.541218	5.159019	6.630384	0.010025363	0.400833293	79	130	134	61	240	116
Ppp3r1	0.53954	8.305003	15.3765	8.80768E-05	0.017877937	4522	4217	4458	3095	8443	4007
Ehd2	0.53769	6.218568	8.029754	0.004601508	0.263313933	344	286	415	201	684	379
Aim1	0.535639	7.221704	4.391213	0.036124595	0.698343917	10	37	31	13	51	28
Ppil1	0.534115	4.971721	4.510382	0.033689711	0.67948957	186	180	125	110	369	121
Ttc7	0.533823	5.589312	5.317461	0.021112747	0.560125598	36	43	61	17	87	64
Sdad1	0.533688	5.099492	5.031834	0.024885532	0.600939511	316	305	384	205	751	277
Rapgef5	0.53361	4.202876	4.523303	0.033436217	0.67948957	46	79	51	29	108	73
Tmem97	0.533497	4.153354	4.572706	0.032485219	0.671233175	160	114	144	85	222	181
Sertad4	0.533204	4.921386	7.317127	0.006830053	0.326567584	197	209	268	133	440	233
Morf4l2	0.531502	4.720721	7.514057	0.006121933	0.30740795	206	339	261	161	475	307
Ccdc50	0.529358	6.416571	16.15781	5.82778E-05	0.013779183	547	679	733	369	1300	685
Cep250	0.529241	4.483382	5.541766	0.018567833	0.529766703	198	248	363	142	467	345
Tmem18	0.527791	4.106239	4.392319	0.036101177	0.698343917	190	189	265	127	361	262
Parp14	0.524994	5.361473	4.867854	0.0273615	0.62042549	202	191	199	104	430	197
Rgnef	0.524551	4.849792	7.215276	0.00722857	0.338321803	185	269	259	127	485	248
Syngr3	0.523221	5.67054	8.856519	0.002920441	0.196784986	1025	902	1033	565	1778	1171
Smpd2	0.522508	3.786111	4.14679	0.041713526	0.745438726	97	89	136	56	224	111
Plekhm1	0.522327	4.67719	4.332695	0.037387005	0.704394754	83	110	113	43	233	106
Cdv3	0.519501	5.583848	6.25823	0.012361763	0.429066966	175	306	233	136	299	360
Eif2ak4	0.518236	4.86156	4.447983	0.034942494	0.689745603	104	146	185	64	323	151
Garnl3	0.517338	4.859442	6.207901	0.012718131	0.433666095	425	625	551	343	884	594
Fem1b	0.516904	6.293372	16.52762	4.79465E-05	0.012720219	801	1027	944	624	1566	962
Kctd3	0.51593	4.010315	4.966705	0.025839827	0.607605717	181	210	193	148	252	231
Tubb2c	0.515814	5.143055	5.277378	0.021604253	0.5675616	330	451	345	228	417	584
Hsd17b7	0.515746	5.947553	6.735342	0.009452109	0.387374414	460	800	724	467	839	823
6330403K07Rik	0.513637	4.517303	4.933496	0.02634097	0.611068844	344	460	541	303	668	517
Synpr	0.507939	5.616	8.115899	0.004387871	0.256547383	813	1093	985	512	1592	1251
Ppm1l	0.505502	7.504712	14.38567	0.000148931	0.026703391	2564	2840	2969	1654	4645	3309
Tmem164	0.504872	4.7382	4.672968	0.030640932	0.657286399	190	246	165	118	344	232
Wee1	0.50299	4.783695	4.41718	0.035578785	0.694145031	106	129	150	82	184	161
Zbtb41	0.502707	4.540929	6.555101	0.01045839	0.406178199	244	230	238	115	461	279
Arid3a	0.502472	4.987872	6.548365	0.010498058	0.406861212	129	121	159	62	284	148
Gabra2	0.500746	4.623898	4.342561	0.037170962	0.704024823	364	635	458	318	638	621
Rfesd	0.500631	4.154509	4.631728	0.031385931	0.661410083	231	244	186	116	467	218
Fam89a	0.500551	6.104269	5.563898	0.018334545	0.526683661	1097	1231	784	548	1924	1229
Ankrd27	0.499666	5.271027	8.091358	0.004447683	0.258638786	546	470	557	367	882	519
Tmem110	0.499446	4.611354	5.180367	0.022843503	0.579064072	227	265	326	160	469	308
Cacnb1	0.498243	5.494458	6.839445	0.008916654	0.378444265	853	928	794	546	1465	922

Alkbh6	0.497635	3.902784	4.379958	0.036363892	0.699939273	187	192	185	114	353	189
Fdft1	0.496559	6.071619	11.20765	0.000814608	0.092247969	716	708	610	429	1065	799
Mvk	0.495345	6.096584	10.81688	0.001005791	0.103050476	496	494	419	322	676	554
Nbl1	0.4945	6.771288	7.012153	0.008095828	0.357857709	1718	2136	2177	1209	3376	2261
Pgap1	0.492993	5.427387	5.632449	0.017630982	0.51579438	433	365	402	249	548	531
Rasgrp1	0.492982	4.672594	5.145816	0.023302451	0.58316509	304	470	545	219	843	491
Sidt2	0.489695	6.073105	6.319368	0.011942617	0.429066966	433	448	554	305	835	484
Ttl	0.487297	7.719209	9.818738	0.001727428	0.149889878	4113	3822	3757	2171	7417	4167
Adrm1	0.48679	4.888631	5.529437	0.018699123	0.530778802	311	393	353	191	457	520
Ttyh3	0.485176	5.524366	5.189647	0.022721836	0.579064072	398	684	777	403	1160	530
Arid5b	0.483206	6.443287	5.193056	0.022677312	0.579064072	258	497	427	191	501	612
Lcmt1	0.482848	5.412539	10.2025	0.001402505	0.131463717	555	531	548	305	979	604
Mal2	0.479958	9.481706	13.74928	0.000208901	0.034552777	747	1041	995	482	1649	1075
Mpp7	0.478952	5.290964	4.339795	0.037231411	0.704024823	144	97	146	71	256	127
Zfp451	0.477854	4.446581	6.151083	0.013133162	0.440389035	215	251	293	164	421	258
2510006D16Rik	0.477632	6.150663	9.108827	0.00254379	0.183665054	791	629	674	387	1081	904
Epb4.1l4a	0.475928	5.763259	6.617633	0.010097395	0.400833293	93	111	139	62	187	137
Peg3	0.475482	6.020865	12.05353	0.000516943	0.067409359	854	1139	1342	669	1867	1187
Fdps	0.474409	7.579094	13.38935	0.000253057	0.039171013	1133	996	1068	679	1832	1085
Shc1	0.472262	5.380559	5.868157	0.015417184	0.482847354	430	518	642	304	1108	443
Scamp4	0.469643	5.902107	9.913868	0.00164038	0.145844692	434	561	595	325	808	610
Mtx1	0.468598	4.486698	4.571475	0.03250856	0.671233175	207	253	205	123	469	189
Tppp	0.46753	5.447694	6.960352	0.008333563	0.362893783	274	281	258	192	373	304
Tmem115	0.466758	4.707742	6.458	0.011045389	0.418402432	297	278	231	175	434	284
Col8a1	0.465228	7.010475	9.169713	0.002460532	0.178854064	200	242	251	108	415	273
Fasn	0.465098	7.750111	10.82867	0.000999406	0.102886204	2150	2016	2557	1315	4043	2272
Tmsb10	0.46203	5.851843	8.766721	0.003067765	0.203524147	771	1039	848	482	1310	1144
Orai1	0.460343	4.429466	4.11026	0.042623802	0.750131423	147	218	265	104	409	214
Map3k3	0.456532	5.695212	6.088479	0.013606614	0.448330643	315	435	359	259	569	358
Trpm3	0.455897	5.801185	6.131744	0.013277584	0.441546373	154	156	166	83	247	200
Ebf3	0.451548	5.696989	5.684973	0.017110807	0.510618748	812	1110	1195	553	1879	1094
Perp	0.449423	9.652162	4.315301	0.037771084	0.706680557	36	31	59	15	72	54
Sema3c	0.449379	5.962112	7.922103	0.004883449	0.27505833	157	166	210	115	314	159
Dhcr24	0.44876	8.933464	16.24191	5.57472E-05	0.013477045	2279	2272	2454	1439	4056	2270
Mll1	0.447205	7.935543	9.297189	0.002295057	0.172057298	1947	1914	2790	1273	4086	2123
Fscn1	0.44662	6.501193	8.726915	0.003135465	0.205053712	290	390	397	226	576	366
Inpp5a	0.445516	5.800637	7.367059	0.00664295	0.321190376	426	489	387	237	845	408
Nme3	0.445457	5.309873	7.64143	0.005704268	0.294405785	366	351	416	206	688	386
Wtap	0.444869	4.741797	5.182656	0.02281343	0.579064072	171	182	248	108	287	258
Gprasp2	0.442502	6.016149	9.793294	0.001751494	0.150740568	1238	1210	1339	788	1869	1412
Stim1	0.442345	5.932416	9.149352	0.002488061	0.180246226	417	555	555	295	828	548
Tmem161b	0.440905	4.659381	5.229479	0.022207181	0.574915158	210	236	208	127	390	212
Spns2	0.438989	6.050798	3.914972	0.047857826	0.789126837	89	66	144	56	177	99
Scfd1	0.437737	5.807091	5.039545	0.02477499	0.599616062	358	602	654	274	987	552
Fxyd2	0.43711	8.071162	12.0921	0.00050636	0.066431919	5002	6029	5275	3115	8831	5938
Klf5	0.434737	9.529157	13.43608	0.00024683	0.039050036	1112	1236	1301	729	2128	1160
Nfkbia	0.434343	7.944246	5.885175	0.015268883	0.480505892	1723	2356	1917	1159	2883	2375
P2ry2	0.432828	6.130418	8.047707	0.004556129	0.261412435	216	203	240	136	339	236
Mrrf	0.431103	4.362397	4.2886	0.03836875	0.710449255	186	217	293	149	372	223
Snx17	0.430124	5.787653	6.671142	0.00979864	0.395028775	591	639	534	332	888	694
Mgp	0.430067	7.500802	5.337991	0.020865501	0.55838573	1264	1013	1279	704	1457	1570
Rcn2	0.429542	4.581786	4.874996	0.027248503	0.620400836	221	245	241	133	414	235
Zfp217	0.429164	5.794972	3.902348	0.04821866	0.789653935	69	72	64	25	150	63
Esyt2	0.427901	4.976156	3.900856	0.048261523	0.789653935	206	223	312	157	422	221
Mvp	0.425183	5.395294	5.246163	0.021995227	0.573635513	202	175	197	87	371	199
Malat1	0.424892	13.01219	5.603173	0.017927984	0.520564769	40069	59401	73990	27448	104372	63098
Pnpla8	0.423161	5.366795	5.790627	0.016111843	0.493649021	401	514	547	267	715	584
Trim24	0.422922	5.036733	5.570989	0.018260443	0.52525626	229	292	298	136	399	351
Gna11	0.420551	7.181071	14.11893	0.000171609	0.029538632	1512	1840	1632	1032	2480	1760
Idh1	0.420446	6.645084	7.808026	0.005201468	0.285981922	793	1084	1151	620	1732	910
Nmb	0.418033	6.641125	8.388781	0.003775439	0.236040708	2144	2052	1819	1100	3465	2095
Ccdc41	0.41688	5.082138	4.566756	0.032598233	0.671455635	325	536	440	224	732	468
Fam49b	0.415743	6.776803	10.89356	0.00096499	0.100789978	1190	1159	1202	726	2008	1108
Itpripl2	0.413557	5.295232	3.971974	0.046263504	0.779758933	314	399	516	208	805	368

Mdm4	0.411475	5.826873	9.200149	0.002419954	0.1771011	455	419	513	261	702	522
Pcyt2	0.410218	5.256386	7.430712	0.006411997	0.316223999	494	452	448	262	760	491
1300010F03Rik	0.409681	4.901455	5.761349	0.016382488	0.495595262	321	372	378	198	630	343
Prkab1	0.409667	5.326665	4.329169	0.037464533	0.705237873	78	75	110	44	156	88
Tspyl2	0.409084	4.206448	4.370602	0.036564062	0.700170206	274	298	359	168	454	369
Sumo1	0.408595	4.965709	5.0571	0.024525244	0.598282495	216	225	227	99	391	255
Dnpep	0.408164	6.210771	8.304263	0.003955212	0.242906061	627	760	677	349	1188	735
Rps11	0.4062	6.44835	5.627498	0.017680853	0.516175359	450	566	453	250	683	631
Bcl11b	0.405549	5.242268	4.960222	0.025936878	0.608079186	492	467	477	289	860	414
Upf3a	0.405546	5.370459	5.888865	0.015236915	0.480505892	397	471	395	254	560	496
Gga2	0.405224	6.172321	6.720529	0.009530933	0.38801464	887	899	943	522	1562	877
Camk2a	0.402855	6.664108	7.769907	0.005312377	0.287444516	2091	2195	1908	1128	3687	2001
Slc35a1	0.402776	6.195692	5.210815	0.022446816	0.578055343	183	273	208	143	340	211
Wdr82	0.402073	4.693388	4.664914	0.030784957	0.657286399	273	282	306	140	454	341
Acpp	0.40082	6.744303	8.299548	0.003965495	0.242906061	1057	1269	1388	601	1968	1461
Zfhx2	0.400755	4.895059	5.0261	0.024968061	0.602256495	375	522	409	210	778	456
Lass4	0.398405	5.936004	5.906349	0.015086408	0.477484096	953	1213	1404	571	1982	1342
Qdpr	0.397739	6.950877	9.32066	0.002265843	0.171059249	1783	1826	1582	1141	2560	1671
Wipi1	0.397385	5.330862	4.318498	0.037700189	0.706159831	244	238	296	169	303	310
Ltbp4	0.397221	4.658557	4.3725	0.03652336	0.700170206	127	156	152	92	225	139
Pdlim2	0.393284	6.902944	4.501313	0.03386883	0.681685453	1099	1047	653	610	1294	979
Rnf2	0.392717	4.81023	4.368103	0.036617737	0.700170206	152	181	223	116	288	176
Ngfrap1	0.392285	6.142655	8.485866	0.003579161	0.227837959	1057	1368	1160	601	1800	1425
Klf7	0.390481	6.316103	7.709835	0.005492077	0.289825134	1210	1645	1543	720	2197	1770
Efna1	0.389646	4.7211	4.257535	0.039076587	0.717226397	147	193	215	110	303	172
Prom1	0.388702	6.36666	4.456115	0.034776507	0.689632552	102	174	131	74	188	161
Tubb2a	0.388405	7.929253	10.05971	0.001515471	0.13833339	4322	4663	4505	2477	6735	5019
Atl3	0.388122	5.067191	5.624915	0.017706924	0.51623602	261	280	315	163	406	322
Aldh1a1	0.387215	5.593699	4.24571	0.039349609	0.719937241	130	180	188	118	255	136
Jdp2	0.385683	7.773613	7.608367	0.005809809	0.297475712	334	301	384	214	535	316
Nup50	0.380866	6.447746	8.61032	0.003342636	0.216627007	643	773	694	417	930	805
Pnn	0.377134	6.620801	6.619373	0.010087534	0.400833293	462	655	707	318	913	675
Nme2	0.376016	7.289085	7.742691	0.005393036	0.289603968	1168	971	1027	638	1625	1040
Plekha3	0.373721	4.766637	4.108664	0.042664062	0.750131423	419	376	381	211	611	422
Klhl26	0.370985	5.321454	6.595093	0.010226021	0.402023377	477	499	567	315	735	524
Nptx1	0.3708	6.418344	7.377981	0.006602724	0.319964458	1621	1669	2029	1015	2640	1841
Ralgds	0.36972	6.052	7.38858	0.006563927	0.31882955	774	749	887	445	1355	755
Sc4mol	0.369278	7.934455	11.22734	0.000806012	0.091862058	2091	2366	2122	1245	3502	2143
Maoa	0.367699	5.79891	5.509213	0.018916562	0.532733953	843	955	1010	517	1325	1045
Coro2a	0.367369	6.127927	7.628864	0.005744148	0.295507569	554	706	711	379	931	702
Rasgef1a	0.365492	6.019029	5.505434	0.018957479	0.533051164	1243	1424	1362	754	2302	1205
2700094K13Rik	0.363296	4.91303	4.569903	0.032538399	0.671233175	225	324	265	137	428	289
Aprt	0.358328	6.449095	8.123587	0.004369302	0.256256489	803	884	808	441	1311	859
Zc3h7a	0.357832	5.665733	4.435378	0.035201425	0.691683889	209	258	230	149	300	244
Pofut1	0.354536	5.347404	4.911161	0.026683696	0.61417846	309	265	302	162	485	272
Dhcr7	0.353874	5.345207	4.669902	0.030695683	0.657286399	504	503	439	260	713	523
Dtd1	0.351991	5.284141	4.128368	0.042170005	0.746732391	534	660	454	324	769	572
Ppap2c	0.349615	7.04669	4.95973	0.02594426	0.608079186	264	260	204	135	412	217
Tpm3	0.349511	7.888458	10.45454	0.001223483	0.120754397	852	920	1005	499	1380	974
Acsl4	0.348461	5.492746	4.692401	0.030296287	0.655789647	475	710	529	278	936	588
Osbpl5	0.34805	6.498765	5.90586	0.015090592	0.477484096	436	374	370	207	595	423
Msn	0.343098	7.810328	4.884034	0.027106195	0.619784154	3876	4710	4630	2501	7350	3805
Tmem50b	0.340845	6.813193	8.382242	0.003789046	0.236040708	935	1032	1049	515	1444	1125
Tmem123	0.339557	7.868257	4.176231	0.040994774	0.734911734	324	525	509	291	629	409
Slmo2	0.337721	5.572119	5.851835	0.015560814	0.485226759	399	501	503	235	753	466
Ipo9	0.334672	4.991104	4.695652	0.030239015	0.655209118	354	404	425	197	607	414
Rnh1	0.334425	7.434222	5.956616	0.014662156	0.470852158	608	602	525	313	783	653
Zeb1	0.333922	6.30223	5.144402	0.023321445	0.58316509	1423	1320	1443	818	2156	1274
Kpnb1	0.333184	6.463935	7.783143	0.005273597	0.286532113	896	995	1049	514	1580	940
Eea1	0.333126	7.924761	8.421763	0.003707561	0.233935158	2954	2987	3526	1647	4514	3446
Por	0.333106	7.406	8.909825	0.002836404	0.19402479	1006	1011	1082	569	1636	972
Ldha	0.331388	7.704241	6.715239	0.00955925	0.38806947	1514	1684	1713	788	2256	1955
Slc4a11	0.33133	7.393136	4.555307	0.032816872	0.671824758	180	181	169	116	274	143
Atox1	0.330741	7.212764	6.352461	0.01172186	0.427470411	1033	1078	995	549	1583	1049

Ids	0.330327	7.492118	4.60514	0.031876252	0.666204354	1972	2204	3022	1357	3375	2418
Baz1b	0.329218	7.441917	7.164405	0.007436435	0.340386324	1138	1277	1370	653	1757	1404
Lats2	0.328527	5.877307	5.675596	0.017202498	0.511506439	662	695	816	407	1041	729
Ank3	0.327225	8.44714	3.945527	0.046996136	0.785443766	5504	5977	8201	3161	11214	6255
Prkrir	0.322709	6.452957	3.901046	0.048256052	0.789653935	586	742	920	403	973	834
Ppp2ca	0.321611	7.951161	5.808019	0.015953256	0.49237727	2159	2585	1930	1151	3259	2357
Bod1l	0.320937	7.560445	5.592484	0.018037703	0.522878739	1811	2421	2642	1131	3034	2682
Chd2	0.31914	6.865916	5.127431	0.02355056	0.58579636	735	948	1020	448	1458	867
Ccdc55	0.318734	6.332639	4.908922	0.026718304	0.61417846	432	572	617	271	739	607
Slc25a44	0.317568	5.623101	4.292451	0.038281939	0.710000942	499	564	670	302	877	571
Rassf3	0.317044	6.346587	4.577211	0.032399905	0.671233175	734	881	815	395	1407	733
Zdhhc20	0.316186	6.530315	4.778891	0.028810663	0.635720257	443	765	650	324	868	650
Stambp	0.313791	5.608781	4.1032	0.042802132	0.750131423	436	467	502	264	704	436
Erbb2ip	0.312482	6.741608	3.873627	0.049050418	0.79470542	340	347	353	150	667	295
Psmb10	0.30982	5.342534	3.961953	0.046539673	0.781569653	330	437	517	217	648	424
Syne2	0.306547	7.703519	5.081759	0.024178834	0.592568192	443	549	568	226	916	488
Hspb1	0.306438	9.011851	7.078767	0.007800269	0.348920119	1966	2169	2291	1093	3044	2275
Syt1	0.306144	7.822994	6.085255	0.013631467	0.448462765	4123	5235	5128	2372	7061	5123
Sun2	0.305206	6.49821	5.922558	0.014948238	0.476483392	595	652	766	378	884	697
Coro7	0.304077	5.338742	4.334763	0.037341612	0.704156118	499	632	614	308	842	581
Igsf8	0.303906	6.669161	4.059097	0.043933997	0.757971112	1275	1222	1446	767	2166	1025
Nedd4l	0.303662	7.018708	4.09589	0.042987599	0.751185748	1869	2392	2831	1168	3568	2387
Hook3	0.303036	7.741428	7.8146	0.005182581	0.285981922	1318	1514	1554	781	2146	1432
Aebp1	0.301305	6.574608	4.490257	0.034088558	0.683773541	615	582	757	382	1027	532
Shisa5	0.298041	7.082784	4.016772	0.04504987	0.767932125	1078	1099	937	560	1503	1029
Rasa1	0.295957	7.559866	6.159298	0.013072307	0.440389035	2207	2075	1960	1158	3004	2002
Usp45	0.294072	5.597373	4.297183	0.038175557	0.709313724	374	451	519	226	628	470
Eef1g	0.293031	7.420041	4.894522	0.026942036	0.617682769	1397	1598	1320	697	1949	1629
Med1	0.291794	6.551671	4.949256	0.026101902	0.609781232	682	986	999	478	1368	788
A930001N09Rik	0.291544	7.785255	6.153829	0.013112794	0.440389035	2742	3387	3198	1689	4408	3020
Mycbp2	0.290177	8.554704	6.289141	0.012147993	0.429066966	2977	3831	4210	1785	5383	3780
Azi2	0.287498	5.781469	4.103162	0.042803096	0.750131423	621	735	641	341	1006	639
Mtss1	0.286413	6.618115	5.717211	0.016799393	0.504122385	903	984	1169	555	1468	962
Tm9sf4	0.285409	6.35129	4.879116	0.027183534	0.620371191	703	693	827	358	1203	687
Rps5	0.285156	10.06763	4.821149	0.028112601	0.625442919	3918	4140	3391	2021	5413	3823
Dgkz	0.280357	7.66515	6.30294	0.012053788	0.429066966	3250	3706	3987	1791	5852	3347
Rabgap1l	0.279552	6.329739	4.085185	0.043260721	0.752617461	1127	991	1256	551	1641	1155
Sqle	0.279466	7.615036	5.631172	0.017643831	0.51579438	1404	1467	1504	700	2124	1519
Nktr	0.277147	6.886695	4.239205	0.039500652	0.721473715	835	794	959	424	1433	755
Tmx1	0.275901	6.923893	4.298718	0.038141114	0.709286269	1002	989	1196	619	1588	879
Clptm1l	0.274337	6.154851	4.20501	0.040304739	0.730176079	582	598	732	321	967	599
Osbpl3	0.2738	6.909211	4.558842	0.032749206	0.671717756	1956	2068	2002	1087	3172	1692
Syncrip	0.272792	6.660751	4.385062	0.036255181	0.699611189	693	775	833	407	1003	795
Raph1	0.272749	6.58075	4.924683	0.026475664	0.612059673	1224	1283	1472	704	1977	1209
Tlk2	0.269023	6.396003	4.476112	0.034371829	0.686567536	916	954	965	506	1222	981
Fam126b	0.265742	7.239384	6.304948	0.012040142	0.429066966	1958	2269	2178	1071	3217	2002
Scaper	0.261662	6.90861	4.13388	0.042032877	0.746732391	1143	1298	1490	632	1810	1366
Luc7l2	0.257701	7.428465	4.828982	0.027985158	0.625058265	1064	1361	1545	701	1939	1174
Sgk1	0.251916	6.732169	4.405478	0.035823694	0.696280579	648	692	669	369	847	671
Tmx4	0.251695	7.071111	4.990637	0.025484827	0.603252529	2286	2694	2717	1393	3674	2270
Celf1	0.251387	6.906991	3.96158	0.04654999	0.781569653	1006	1044	1386	634	1613	1009
Rpl31	0.251032	9.143012	3.859037	0.049478732	0.798973908	2580	2723	2335	1398	3660	2251
Cd47	0.25076	6.562907	4.135509	0.041992432	0.746732391	1193	1446	1390	629	2008	1303
Prkar2b	0.249004	7.867234	3.93619	0.047257697	0.787260109	4038	4523	4357	2358	6614	3472
Rab12	0.248322	6.919959	4.27487	0.038679917	0.71294618	960	1157	1104	582	1583	916
Rbm25	0.243703	8.72808	3.91128	0.047963062	0.789126837	2532	3194	3411	1516	4030	3092
Sp1	0.241439	7.549953	3.945478	0.046997487	0.785443766	804	1052	1086	546	1421	808
Stmn2	0.241221	9.576853	3.885028	0.048718451	0.791637017	15051	18468	16536	8109	22620	17013
Mef2a	0.240004	6.951418	3.971388	0.046279613	0.779758933	1312	1604	1560	661	2241	1474
Anapc5	0.238236	7.689294	4.574484	0.032451522	0.671233175	1931	1893	2134	1036	2969	1714
Lrrc58	0.228445	7.276887	3.854001	0.049627463	0.799812338	1692	1998	2280	1050	2742	1788
Ap3d1	0.227206	7.949509	3.88626	0.048682711	0.791637017	3148	3370	3489	1716	4454	3200
Ahnak	0.224779	10.68296	4.336562	0.037302165	0.704029287	6395	7161	7321	3582	10393	5878
Evi5	0.22438	7.682579	4.032652	0.044627724	0.763283076	1961	2464	2461	1094	3229	2208

Downregulated genes

Clec18a	-7.2325	-0.51637	10.83816	0.000994295	0.102886204	25	12	27	0	0	0
Tomt	-7.02269	-0.66756	9.25967	0.002342552	0.173801188	23	19	13	0	0	0
Ckap2	-6.83462	2.47138	32.8811	9.7971E-09	1.4053E-05	14	28	7	0	0	0
Cntnap5a	-6.72368	-0.84691	7.447097	0.006353883	0.315727846	8	33	5	0	0	0
4933437F05Rik	-6.53282	-0.75322	10.97729	0.000922351	0.09824412	17	10	12	0	0	0
Fbxo40	-6.2541	-1.15599	5.360172	0.02060174	0.553142638	14	14	4	0	0	0
Phox2b	-6.18374	-1.18498	5.323532	0.021039321	0.559557518	10	6	15	0	0	0
Pcdhga10	-6.14382	-0.39449	11.6273	0.000649907	0.077256336	11	3	16	0	0	0
4931417G12Rik	-6.09799	-0.13629	11.00403	0.000909139	0.097477719	10	19	0	0	0	0
Tnfrsf9	-5.99465	-0.61585	5.733293	0.016646244	0.501644289	10	1	16	0	0	0
Snora23	-5.96189	-1.09238	6.027107	0.014087807	0.457184402	12	9	5	0	0	0
Hist1h1b	-5.89243	2.736771	19.44047	1.03784E-05	0.003987546	11	5	9	0	0	0
Gmfg	-5.84792	-1.02107	6.008534	0.014236847	0.460631573	11	9	4	0	0	0
Slc4a9	-5.81263	-0.00942	11.11632	0.000855716	0.094418351	7	0	17	0	0	0
Bmp15	-5.78485	-0.19665	9.93808	0.001618943	0.145054412	11	0	12	0	0	0
Rab19	-5.77242	1.762345	16.6978	4.38318E-05	0.011990654	9	14	0	0	0	0
1700026J04Rik	-5.7644	-0.96606	6.782597	0.009205086	0.383830697	8	4	11	0	0	0
Zar1	-5.73021	-1.43584	4.268024	0.038836062	0.714796166	11	3	8	0	0	0
Nos3	-5.68839	-1.11884	4.351805	0.036969734	0.70268622	6	6	10	0	0	0
Fut1	-5.68435	0.635808	15.3826	8.77931E-05	0.017877937	5	7	10	0	0	0
Robo3	-5.67412	-1.44124	4.708983	0.030005393	0.652776571	4	12	6	0	0	0
Apol11b	-5.67396	-1.44124	4.132389	0.042069921	0.746732391	4	12	6	0	0	0
Lrmp	-5.59368	-1.11254	3.974447	0.04619563	0.779758933	17	0	2	0	0	0
Gm2518	-5.43852	-0.64222	6.210013	0.012702972	0.433666095	8	8	2	0	0	0
Tnfsf8	-5.41246	-1.26945	4.199966	0.040424792	0.730176079	13	0	4	0	0	0
Slc5a9	-5.39074	0.293346	11.001	0.000910626	0.097477719	12	5	0	0	0	0
Efhb	-5.3746	-1.53426	3.904321	0.048162097	0.789653935	2	6	10	0	0	0
Ppp1r14d	-5.27628	-0.79064	4.943675	0.026186308	0.610427523	0	9	8	0	0	0
4632434I11Rik	-5.27494	0.337996	7.739025	0.005403996	0.289603968	0	0	17	0	0	0
D630029K05Rik	-5.26937	-0.97927	4.537347	0.033162951	0.67576525	0	0	17	0	0	0
S100a14	-5.24302	8.893802	18.48298	1.71428E-05	0.006046643	0	8	8	0	0	0
5031410I06Rik	-5.18167	-1.15476	4.365457	0.036674652	0.700170206	7	0	8	0	0	0
Prss32	-5.16652	5.61485	17.29039	3.20805E-05	0.009586715	15	0	0	0	0	0
Clca5	-5.07761	1.978302	13.10931	0.000293832	0.044145081	7	7	0	0	0	0
Slc10a1	-5.05608	-0.96041	6.597559	0.01021187	0.402023377	4	5	5	0	0	0
Pthlh	-4.96823	3.255271	41.97606	9.23977E-11	2.84004E-07	8	41	45	0	0	2
Arg1	-4.95287	6.957004	15.08308	0.000102881	0.02013601	0	8	5	0	0	0
Tlr1	-4.94429	-0.83886	7.404071	0.006507644	0.318292152	3	5	5	0	0	0
Saa1	-4.90932	-0.39399	5.501255	0.019002834	0.533051164	0	13	0	0	0	0
Ucn2	-4.89822	-0.03446	5.522446	0.018773988	0.530803048	0	0	13	0	0	0
Rassf6	-4.87983	1.650325	30.85517	2.78021E-08	2.99095E-05	14	18	18	1	0	0
Scnn1a	-4.82787	3.880018	12.61956	0.000381731	0.052989209	0	0	12	0	0	0
Krt17	-4.79593	0.834517	7.694299	0.005539552	0.289825134	0	0	12	0	0	0
Hes7	-4.78796	-1.46705	4.471615	0.034462397	0.686567536	0	3	9	0	0	0
4930512B01Rik	-4.78743	-0.00466	5.338518	0.020859205	0.55838573	0	0	12	0	0	0
Rasl2-9-ps	-4.78054	-1.341	3.996443	0.0455964	0.774311083	7	3	1	0	0	0
Hist1h2ab	-4.70053	-0.1463	4.964849	0.025867579	0.607605717	10	0	0	0	0	0
Uchl4	-4.68553	0.348539	8.339211	0.003879845	0.239881444	0	5	6	0	0	0
Otx1	-4.59031	7.186088	11.64178	0.000644867	0.077083157	2	8	0	0	0	0
Krt23	-4.58592	4.246525	11.11761	0.000855119	0.094418351	1	8	1	0	0	0
Lrrc26	-4.58213	3.974541	10.96551	0.000928232	0.0983834	0	10	0	0	0	0
Lamb3	-4.57945	3.764271	10.92249	0.000950036	0.100200869	0	0	10	0	0	0
Pdyn	-4.56542	3.702886	8.854391	0.002923846	0.196784986	0	10	0	0	0	0
Dcaf12l2	-4.55423	0.734242	6.815243	0.0090383	0.379813343	0	10	0	0	0	0
Slfn3	-4.54852	-0.36641	5.367361	0.020516995	0.553142638	0	10	0	0	0	0
Cryba1	-4.54469	-0.33584	4.08318	0.043312069	0.752617461	0	10	0	0	0	0
1110059M19Rik	-4.47024	0.571256	7.736722	0.005410894	0.289603968	5	0	4	0	0	0
Cldn23	-4.44176	5.280222	10.33438	0.001305749	0.124864452	0	8	1	0	0	0
Erp27	-4.43409	0.614209	7.570955	0.005931636	0.300294309	2	0	7	0	0	0
Trim72	-4.41421	1.236462	7.081059	0.007790296	0.348920119	0	9	0	0	0	0
Gsta1	-4.41265	1.058571	6.818117	0.009023767	0.379813343	0	9	0	0	0	0
AB099516	-4.40338	-1.01831	3.980218	0.046037614	0.779343278	6	8	23	0	0	1
Rdh18-ps	-4.32095	2.404325	8.495631	0.003560004	0.227837959	8	0	0	0	0	0

Cngb3	-4.24619	0.034584	5.219635	0.022333244	0.576857229	0	8	0	0	0	0
Gm10639	-4.24426	-0.12984	4.836075	0.02787026	0.624609624	0	8	0	0	0	0
Lpin3	-4.18807	2.882001	18.81752	1.43839E-05	0.005245509	13	1	16	0	0	1
Aim1l	-4.10514	6.581407	8.215658	0.004153046	0.247526169	7	0	0	0	0	0
Gsta2	-4.09784	5.606193	8.073528	0.004491658	0.259791709	0	0	7	0	0	0
Psg16	-4.05738	-0.22132	4.687803	0.030377469	0.656886043	0	1	6	0	0	0
Notum	-4.05662	-0.75297	3.938291	0.047198713	0.78723063	0	4	3	0	0	0
Slc39a4	-4.03417	5.402814	23.12844	1.51533E-06	0.000931536	8	15	4	0	0	1
Ces1d	-3.89927	4.821815	6.614382	0.010115843	0.400833293	3	0	3	0	0	0
Tmprss11e	-3.89799	6.062929	7.02037	0.008058755	0.357509653	6	0	0	0	0	0
Krt14	-3.89012	8.382095	6.811792	0.009055784	0.379813343	1	0	5	0	0	0
Cep55	-3.86881	1.544963	5.79116	0.016106959	0.493649021	0	0	6	0	0	0
Tmem102	-3.86876	1.444383	5.787778	0.016137973	0.493649021	0	0	6	0	0	0
Rasal1	-3.854	0.078094	4.214198	0.040087018	0.728202461	0	3	3	0	0	0
Lipc	-3.84838	-0.74471	4.377562	0.036415049	0.700170206	20	13	15	0	3	0
Hpn	-3.84398	-0.85576	4.057911	0.043964861	0.757971112	21	16	5	0	0	2
Qrfp	-3.82926	0.102839	8.122889	0.004370986	0.256256489	12	1	24	0	2	0
Cpne7	-3.77601	3.969578	16.40949	5.1029E-05	0.012916603	14	0	8	0	1	0
Krt6a	-3.64862	12.23232	5.805822	0.015973198	0.49237727	1	2	2	0	0	0
Lass3	-3.6468	4.715577	5.811199	0.015924431	0.492284556	0	0	5	0	0	0
Il1a	-3.63806	2.693285	5.329034	0.020973006	0.558484148	0	5	0	0	0	0
Tmem139	-3.63362	1.796525	5.254501	0.021890072	0.572976638	0	0	5	0	0	0
Gli2	-3.62692	0.049831	4.995167	0.025418196	0.603252529	0	11	14	1	0	0
C8g	-3.61248	0.669942	18.3488	1.83934E-05	0.006282338	25	19	31	0	4	1
Atg9b	-3.58911	0.680598	10.0272	0.001542452	0.13985517	4	7	10	1	0	0
Lpar2	-3.50921	2.741631	8.852601	0.002926715	0.196784986	17	0	0	0	1	0
Oas3	-3.40305	1.998645	4.0623	0.043850743	0.757810431	4	0	0	0	0	0
Fermt1	-3.35503	6.918741	4.699933	0.030163787	0.654898134	0	0	4	0	0	0
Ankk1	-3.35359	4.731514	4.656667	0.03093318	0.65831681	0	0	4	0	0	0
Capns2	-3.35268	4.029696	4.602624	0.031923066	0.666204354	0	4	0	0	0	0
Ptgs2	-3.33768	0.851107	4.104533	0.04276839	0.750131423	0	0	4	0	0	0
Spna1	-3.25307	0.460763	5.632382	0.017631657	0.51579438	58	25	19	4	3	0
Pde4c	-3.23918	-0.01231	4.32142	0.03763551	0.706159831	1	58	37	1	1	5
Itgal	-3.16692	-0.33098	4.667456	0.030739432	0.657286399	24	22	15	0	0	5
Mybl2	-3.02456	0.81083	4.445487	0.03499361	0.690121455	0	0	14	0	0	1
Xlr4c	-3.00651	4.842235	9.214033	0.002401671	0.176966956	6	3	4	0	0	1
Polh	-2.9443	0.247035	6.666981	0.00982154	0.395028775	16	13	3	0	0	3
Tmc8	-2.83486	-0.19973	4.39544	0.036035164	0.698343917	0	6	13	0	2	0
Phldb3	-2.82038	5.63657	21.12664	4.29909E-06	0.002151144	0	16	23	4	0	0
Calb2	-2.81125	4.339358	54.17817	1.8311E-13	9.84946E-10	597	890	1004	23	129	147
Nnmt	-2.748	1.078204	4.232828	0.039649328	0.723036818	7	0	3	0	1	0
Ect2	-2.73481	1.989406	7.649036	0.005680268	0.294405785	10	0	9	0	0	2
Zfp57	-2.71455	-0.67477	4.467915	0.034537106	0.686784085	17	14	7	2	2	0
Myo5b	-2.71136	4.403476	11.13913	0.000845256	0.094418351	1	6	12	2	0	0
Il12rb2	-2.70289	0.031835	4.338481	0.037260161	0.704024823	14	31	4	0	2	4
Snai2	-2.6927	4.301351	26.70093	2.37507E-07	0.00017034	24	37	12	1	6	2
Crygs	-2.68665	0.669065	8.404027	0.003743906	0.235537692	49	22	22	2	11	0
Tmprss13	-2.62681	5.179094	6.342239	0.011789599	0.427765628	0	10	0	1	0	0
Wnt7b	-2.60309	6.582811	14.51798	0.000138828	0.025530122	5	7	13	3	0	0
Mir351	-2.58405	0.090784	4.067979	0.043703526	0.75588831	0	12	4	0	2	0
Lrfn1	-2.57542	0.384308	8.231533	0.004116875	0.24672577	44	13	32	1	8	4
1300002K09Rik	-2.51474	0.23075	4.273882	0.038702414	0.71294618	11	13	6	0	1	3
Slc18a1	-2.46424	0.862367	4.045413	0.044291539	0.761163536	13	35	0	4	1	0
Ly6g6e	-2.43747	1.988292	4.359248	0.036808544	0.700860741	2	0	7	1	0	0
Neb	-2.4365	2.020723	11.03663	0.000893292	0.097477719	118	117	170	5	34	26
E130306D19Rik	-2.42231	1.870156	6.564966	0.010400574	0.405396304	0	9	7	1	0	1
Clec4a3	-2.39746	0.598046	7.133816	0.007564357	0.343364338	36	51	8	4	4	5
Grxcr2	-2.38978	1.542459	15.60071	7.82253E-05	0.016664322	84	116	109	7	13	25
Rbp2	-2.30647	5.941502	7.448177	0.006350075	0.315727846	0	7	7	0	1	1
Chia	-2.27416	0.031699	4.924145	0.026483898	0.612059673	40	24	25	2	3	9
Cth	-2.23048	1.951751	6.592641	0.010240114	0.402023377	11	6	3	2	0	1
Tnfaip2	-2.22064	3.655094	16.29552	5.4192E-05	0.013249948	13	21	26	8	0	1
Prom2	-2.20821	4.663022	6.402917	0.011393301	0.421924741	8	4	1	1	1	0
Rpgrip1	-2.19903	0.86124	6.259924	0.012349948	0.429066966	36	32	8	0	5	9

Gxylt2	-2.19892	0.714789	5.177914	0.022875775	0.579064072	12	9	9	4	0	0
Plekhg6	-2.17571	5.108442	8.287825	0.003991177	0.2431336	12	5	1	0	3	0
Enpep	-2.16653	3.039849	15.52622	8.13687E-05	0.016833925	37	91	116	2	12	29
Zbp1	-2.1534	1.299594	6.376359	0.011565045	0.42523009	13	13	7	3	0	2
Htr1a	-2.13983	0.02779	4.51116	0.0336744	0.67948957	28	36	25	3	8	5
Atp2c2	-2.12771	2.567668	5.010638	0.025192024	0.602257311	2	0	11	2	0	0
Susd1	-2.12608	0.20637	4.416693	0.03558895	0.694145031	15	6	6	0	2	3
E2f8	-2.11661	2.38558	8.263303	0.004045447	0.2431336	4	8	18	1	0	4
Frrs1	-2.09809	3.531027	11.85327	0.000575601	0.072003678	8	10	17	5	1	0
Sox21	-2.09472	5.072397	15.54171	8.07046E-05	0.016833925	20	26	3	1	3	5
Irx5	-2.09124	4.081863	13.09902	0.00029545	0.044145081	15	22	8	1	0	7
Esrp1	-2.08994	7.076086	5.782118	0.016190024	0.493649021	0	9	3	0	0	2
Dnd1	-2.04812	1.579134	5.116539	0.023698832	0.587113195	30	57	41	12	1	4
Chodl	-2.04748	2.272293	18.53183	1.67091E-05	0.005991867	218	149	139	15	50	37
Hpse	-2.03765	2.903807	19.65394	9.28116E-06	0.003767802	63	62	55	12	20	2
Cdh3	-2.00744	0.827262	6.294416	0.012111891	0.429066966	13	12	15	2	0	5
Melk	-1.99837	3.191857	4.130417	0.042118967	0.746732391	11	0	0	0	0	2
Dyrk2	-1.99583	1.230121	3.889817	0.048579722	0.790651519	0	8	10	2	1	0
B3gnt7	-1.9836	3.629823	7.235081	0.007149257	0.337332033	10	12	0	4	0	0
Dhx58	-1.97978	2.011766	7.061022	0.007877905	0.351661839	37	9	20	3	0	9
Ccdc69	-1.97014	0.595232	4.860814	0.027473364	0.621574019	7	15	10	0	0	6
Unc5a	-1.94557	3.388159	13.79171	0.000204236	0.034330756	18	28	67	1	17	7
Pcdhb19	-1.94257	0.777468	6.269194	0.012285505	0.429066966	47	12	29	6	3	7
Hrasls	-1.92675	0.994163	6.726099	0.009501217	0.387909277	49	68	74	3	25	16
9530008L14Rik	-1.91861	6.500104	16.96499	3.80755E-05	0.011070699	5	33	16	0	0	11
Grin2c	-1.82749	0.159266	4.03624	0.044532946	0.763283076	25	37	30	6	9	4
Xpnpep2	-1.8223	0.804583	4.30166	0.038075182	0.709286246	20	3	18	4	2	2
Fkbp11	-1.80911	0.60613	5.660794	0.017348267	0.51243781	39	20	24	2	10	8
AI467606	-1.79243	1.334813	8.684415	0.003209426	0.208622386	17	20	16	2	5	5
Rerg	-1.78993	1.83936	4.621602	0.031571743	0.662729393	4	12	2	0	2	2
Ccbe1	-1.78911	2.046754	6.482281	0.010895505	0.417874669	14	20	19	6	0	4
2200002D01Rik	-1.789	7.97249	31.23615	2.28472E-08	2.731E-05	49	65	26	3	7	22
Slc5a1	-1.77695	6.597886	7.584937	0.005885803	0.300092245	7	5	10	0	5	0
Hal	-1.7706	2.246818	12.52824	0.000400847	0.05493388	28	51	32	3	6	16
Spaca1	-1.71106	1.85765	4.225113	0.039829959	0.725026572	22	16	0	0	5	5
Nxph4	-1.69815	3.286926	9.089185	0.002571255	0.185027139	18	8	18	7	3	0
Fam83g	-1.67398	6.543396	4.452855	0.034842941	0.689745603	0	11	2	3	0	0
Fbp2	-1.67392	1.029023	6.241976	0.012475719	0.430862859	56	80	28	7	18	16
Gsto2	-1.65525	1.693538	6.343894	0.011778605	0.427765628	29	41	52	12	5	8
Naip1	-1.64585	3.848699	6.827053	0.008978728	0.378796703	4	34	26	3	14	0
Dbx2	-1.60682	3.474678	6.097652	0.013536163	0.446693367	17	35	7	10	0	2
BC048355	-1.60225	2.563056	6.360049	0.01167183	0.42709371	31	26	4	0	7	10
Fzd5	-1.60182	3.864229	9.452815	0.002108253	0.167681269	34	36	16	7	0	13
Cgn	-1.58275	5.741932	16.05164	6.16381E-05	0.014309931	60	53	15	9	22	4
Dennd2c	-1.5663	4.259179	5.400289	0.020133412	0.549185963	9	0	10	1	4	0
Tnfrsf22	-1.5338	1.565694	4.077331	0.043462259	0.75353261	48	25	11	5	2	14
Glb1l2	-1.51506	3.616826	13.76351	0.000207324	0.034552777	146	179	225	38	40	59
Akr1b3	-1.5031	1.7201	4.651559	0.031025357	0.658325037	8	22	20	4	0	8
Ppp2r5d	-1.49579	1.783211	7.160825	0.00745129	0.340386324	34	95	53	12	12	22
Aldh1a7	-1.48672	2.409979	8.507245	0.003537353	0.227193065	31	18	39	8	6	9
Sncaip	-1.46821	1.4737	4.556862	0.032787098	0.671824758	80	48	27	6	14	25
D14Ertd668e	-1.46417	4.415692	11.00544	0.000908449	0.097477719	24	20	37	8	6	8
Ptchd1	-1.45687	2.613554	16.38737	5.1628E-05	0.012916603	116	80	107	15	44	31
Kctd21	-1.45681	1.977421	5.722944	0.016744634	0.503180932	35	77	64	6	9	33
Haus7	-1.45383	2.194809	6.299807	0.01207511	0.429066966	53	23	25	10	9	8
Gm525	-1.44934	0.986357	5.021631	0.025032583	0.602257311	23	66	24	6	21	7
Cited4	-1.43319	4.750014	9.812768	0.001733045	0.149889878	16	7	26	5	3	6
Myh8	-1.4262	1.769448	5.909211	0.015061915	0.477484096	89	82	147	12	76	19
Ibsp	-1.4245	2.590231	10.35438	0.001291676	0.124864452	115	282	200	30	98	53
Adamts4	-1.36713	1.318039	5.481536	0.019218364	0.534240733	85	76	63	12	42	19
Efcab6	-1.36696	4.288002	8.571668	0.003414342	0.22060957	11	19	15	0	7	7
Nubpl	-1.35585	1.641525	4.573526	0.032469672	0.671233175	10	23	41	3	11	9
Cux2	-1.35395	2.035977	9.024129	0.002664388	0.190455085	67	83	132	14	58	21
Tlr5	-1.32247	3.113751	7.152018	0.00748797	0.340724832	22	22	34	4	23	0

Adcy7	-1.31974	2.633515	9.715257	0.001827445	0.151985985	76	83	43	9	21	34
Cdkl4	-1.30561	2.272876	4.015719	0.045078001	0.767932125	126	158	169	4	99	69
Agphd1	-1.30318	1.19967	4.680551	0.030505967	0.657228793	43	50	37	6	20	17
Lmtk2	-1.30043	1.889707	5.27527	0.021630438	0.5675616	53	45	56	7	23	21
Hs3st3b1	-1.3	3.539048	7.039227	0.007974343	0.3544958	12	45	18	8	5	9
Btc	-1.2954	4.908545	12.59993	0.000385762	0.053205433	35	15	49	12	12	7
Hcrtr2	-1.29489	0.964773	4.096916	0.042961522	0.751185748	55	50	65	12	29	14
Exph5	-1.29473	6.416335	11.69575	0.000626432	0.075720799	16	27	16	3	4	12
Tctn1	-1.29104	2.610459	8.468948	0.003612603	0.229174424	74	123	69	21	31	29
Akr1c13	-1.28828	4.114736	3.9412	0.04711716	0.786480075	5	8	6	0	0	6
Angptl1	-1.2705	4.421279	8.838564	0.00294931	0.197686487	75	53	17	10	33	8
Gjc2	-1.2643	2.914318	8.166508	0.004267097	0.252922487	258	171	108	42	87	48
Mgmt	-1.23734	2.857838	5.936	0.014834647	0.473564206	96	56	26	16	31	13
Det1	-1.22715	2.767176	6.001918	0.014290332	0.461667848	21	42	47	13	6	13
Srpx	-1.22323	3.932594	13.15197	0.000287218	0.043519553	85	81	69	16	36	28
Cdt1	-1.20413	3.286857	4.183127	0.040828297	0.732898399	9	48	21	6	4	14
Chst9	-1.20352	4.429379	5.772853	0.016275594	0.494228684	2	25	19	3	2	10
Kcnip1	-1.2009	3.367551	16.46102	4.96606E-05	0.012720219	369	315	299	60	174	116
Smagp	-1.19227	6.724717	21.35656	3.81314E-06	0.001953419	76	77	38	10	33	25
Car3	-1.17957	8.156719	17.46533	2.92595E-05	0.009013831	28	34	58	7	15	20
Esrrg	-1.17829	3.391285	11.40397	0.000732873	0.084776846	325	293	356	57	137	147
Lingo3	-1.17615	1.17422	4.448821	0.034925346	0.689745603	66	72	57	13	33	23
Arc	-1.16871	1.685539	4.375661	0.036455684	0.700170206	33	40	29	10	6	16
Pla2g5	-1.15331	2.916435	7.32599	0.006796453	0.326567584	55	42	30	6	17	23
A730017L22Rik	-1.15075	1.788011	3.928709	0.047468368	0.787455204	37	57	77	8	27	27
Dpyd	-1.1419	1.878397	6.46957	0.010973705	0.418402432	74	78	76	18	35	27
Smad9	-1.14173	3.735261	16.46396	4.95837E-05	0.012720219	330	476	466	65	228	181
Zfp599	-1.1378	1.34816	4.651756	0.031021794	0.658325037	29	43	30	10	11	13
Nfe2l3	-1.13425	4.618723	9.499609	0.002055157	0.166235956	19	70	50	17	15	14
Tmigd1	-1.13237	2.953915	6.835651	0.008935614	0.378461956	32	17	28	3	15	11
Obfc2a	-1.12686	4.418751	10.40626	0.001255885	0.122825572	26	68	53	8	27	19
2810417H13Rik	-1.12266	5.344493	7.245215	0.007109018	0.336911098	26	12	30	1	15	10
Rassf9	-1.11284	3.132484	4.847813	0.027681208	0.622349921	10	7	28	1	10	6
Osgin1	-1.11273	4.015033	13.56703	0.000230193	0.037151488	53	46	64	5	30	27
Supv3l1	-1.11225	3.287592	14.34712	0.000152012	0.026808903	125	125	180	38	70	45
Plk4	-1.11101	2.34158	4.321261	0.037639023	0.706159831	28	98	33	6	37	20
Calb1	-1.09459	4.570829	23.36775	1.33803E-06	0.000846735	747	737	785	179	358	284
Col13a1	-1.08988	3.963179	8.177731	0.004240776	0.25205675	28	52	28	14	8	15
Mcm5	-1.08073	4.031514	4.194596	0.040553019	0.730176079	9	25	60	14	5	10
Aldh1a3	-1.07182	5.12137	9.532665	0.002018468	0.164999521	27	48	54	13	13	20
Sema7a	-1.06462	4.893539	31.63552	1.85997E-08	2.35406E-05	836	1085	919	186	487	411
Cdr1	-1.05387	7.008792	32.33755	1.29585E-08	1.74259E-05	3189	4694	4589	797	1851	2044
Tlr4	-1.05219	4.303831	14.53201	0.000137798	0.025530122	85	111	73	18	57	31
Cxcr4	-1.0513	2.455475	4.199089	0.04044569	0.730176079	147	161	186	50	112	24
Cep152	-1.04842	2.348155	5.45396	0.01952402	0.542037179	44	51	63	19	24	13
Zfp384	-1.04831	2.430445	6.278796	0.012219106	0.429066966	64	56	34	10	34	18
Folh1	-1.04625	2.156953	5.902441	0.015119916	0.477709429	103	156	142	29	81	46
Hapln1	-1.0422	2.538252	7.690987	0.005549728	0.289825134	154	178	191	28	98	83
Olfm3	-1.04057	2.256241	6.909066	0.00857598	0.370523669	119	142	168	32	89	47
Rem2	-1.03155	3.874109	16.9326	3.87307E-05	0.011111063	455	420	492	118	248	155
Gemin4	-1.02991	2.406003	4.547986	0.032957488	0.674062088	49	60	22	14	26	10
Fgd2	-1.02877	2.471956	4.084782	0.043271031	0.752617461	10	23	43	3	19	9
Ccdc111	-1.02013	2.178823	4.81669	0.02818542	0.625442919	41	75	63	9	56	13
Gal	-1.0114	2.198182	4.071409	0.043614888	0.755034398	132	135	137	14	95	68
9130011E15Rik	-1.00344	2.328128	6.304174	0.012045403	0.429066966	65	61	44	18	24	22
Gstm2	-0.9851	5.033719	19.34638	1.09026E-05	0.004115431	332	263	234	46	220	98
Isg20l2	-0.97851	2.80925	4.682322	0.030474529	0.657228793	77	93	97	17	80	21
Aldh3a1	-0.96917	13.67583	4.284705	0.03845675	0.711111914	4	18	10	2	7	4
C3	-0.96264	2.709076	4.982245	0.025608733	0.605491755	32	37	22	8	17	12
C1qtnf7	-0.95747	3.372223	5.665418	0.017302589	0.51243781	29	45	49	2	16	33
D2hgdh	-0.94663	2.619748	7.691839	0.005547108	0.289825134	63	74	66	15	38	31
Zcwpw1	-0.94269	3.804217	12.85078	0.000337339	0.047961082	114	217	179	50	94	59
Nos1	-0.94138	4.411648	15.14261	9.96876E-05	0.019859985	619	558	746	122	501	234
Angptl7	-0.93306	8.451049	6.586297	0.010276671	0.402023377	106	161	188	7	168	54

Rab11fip1	-0.93305	6.155877	7.492857	0.006194418	0.309951412	14	21	31	4	20	4
Slc25a36	-0.93298	2.286823	4.497391	0.033946611	0.682612404	81	85	81	31	22	36
Nmral1	-0.92957	2.66916	5.175696	0.022904999	0.579111594	51	98	50	16	33	32
Bambi	-0.91801	3.383094	6.858201	0.008823536	0.376191245	133	221	192	39	142	60
Mmp23	-0.91507	2.239825	4.608611	0.03181179	0.665819534	33	37	30	3	20	21
Ednra	-0.91	2.16162	4.213606	0.040101014	0.728202461	72	72	93	19	64	22
Cd302	-0.9097	2.840179	5.088155	0.024089808	0.591685293	64	60	73	28	21	24
D430042O09Rik	-0.90744	3.122157	7.286681	0.006946771	0.330532334	113	82	152	22	96	40
Fam84b	-0.90471	5.337327	9.726482	0.001816318	0.151985985	49	68	76	1	53	35
Slc17a7	-0.90341	5.470233	33.88027	5.86099E-09	9.7004E-06	1232	1506	1355	319	904	549
3110009E18Rik	-0.89954	2.222764	4.197485	0.040483979	0.730176079	27	57	62	6	33	26
Lypd6b	-0.88668	3.13278	9.421927	0.002144061	0.167681269	69	83	86	12	52	42
Serpinb1a	-0.88612	6.334673	28.53535	9.20034E-08	7.91818E-05	1282	1264	1206	307	882	469
Hs3st2	-0.88341	4.147418	16.79286	4.16899E-05	0.011649344	501	552	553	131	330	233
Zfp952	-0.87961	4.206373	14.20423	0.000164001	0.028456855	221	305	256	58	149	131
Ccbl2	-0.8785	2.573837	4.539559	0.033120123	0.67576525	73	76	103	30	28	39
Pdzk1ip1	-0.87788	8.336936	7.62556	0.005754679	0.295507569	44	30	14	4	28	8
Ndp	-0.87715	2.934401	4.990558	0.025485989	0.603252529	218	221	239	66	89	116
Ung	-0.87546	3.417844	5.403144	0.020100506	0.549185963	44	35	39	7	22	23
Gabra5	-0.85963	2.843091	5.994089	0.01435389	0.463025932	210	177	243	58	127	87
Zbtb24	-0.8536	2.281663	4.678295	0.030546049	0.657228793	43	51	71	15	35	22
Sirt6	-0.84482	3.649072	9.438891	0.002124319	0.167681269	193	221	165	31	154	92
Klhdc5	-0.83318	3.07465	6.718859	0.009539865	0.38801464	106	152	110	25	83	61
Ncstn	-0.83298	3.785418	6.602733	0.010182235	0.401983424	79	119	132	21	55	70
Rbl1	-0.83278	3.73942	4.642995	0.031180541	0.659666203	21	53	22	12	16	13
Pvalb	-0.83228	6.006608	22.89643	1.70969E-06	0.001021826	1898	2125	1810	562	1178	801
Chrna6	-0.83038	2.881965	4.517962	0.033540756	0.67948957	170	242	231	37	116	139
Ppm1h	-0.83027	4.15118	9.713152	0.001829539	0.151985985	265	353	418	51	285	169
Usp38	-0.82841	3.52529	8.272247	0.004025565	0.2431336	126	141	165	34	103	61
Vav3	-0.82534	4.197631	9.416037	0.002150959	0.167681269	270	391	265	85	153	160
March9	-0.82379	3.091306	6.418571	0.011293301	0.420038738	111	96	124	18	75	63
1700030C10Rik	-0.82081	2.581892	4.117216	0.042448877	0.748631171	73	145	123	27	89	42
Cenpl	-0.81618	2.971552	5.179343	0.022856963	0.579064072	76	83	75	13	82	23
Tmem138	-0.80366	3.113015	4.666563	0.030755413	0.657286399	119	124	95	11	115	52
Dgkb	-0.79791	2.536337	3.883918	0.048750653	0.791637017	135	209	157	47	90	83
Lace1	-0.79512	2.953902	5.857408	0.015511622	0.484394866	123	139	98	43	69	45
P2rx6	-0.79419	3.226166	4.575991	0.032422995	0.671233175	368	232	191	60	202	124
Gdpd2	-0.7917	4.637227	7.640928	0.005705857	0.294405785	90	121	53	31	57	31
Pigv	-0.78641	3.60386	6.557635	0.010443505	0.406178199	53	107	80	20	67	27
Atad5	-0.78489	3.366869	4.339591	0.037235872	0.704024823	75	86	137	21	49	65
Tmem40	-0.78393	4.663187	6.134281	0.013258546	0.441546373	108	79	42	18	63	31
Cd274	-0.78193	5.338081	6.345502	0.011767933	0.427765628	66	33	46	10	48	14
Zfp652	-0.78053	3.521295	6.217151	0.012651851	0.433666095	65	72	91	28	44	29
Pde5a	-0.77813	4.612298	6.438799	0.011165411	0.41872495	62	59	109	11	83	24
Irf6	-0.7712	7.158437	5.399817	0.020138861	0.549185963	64	73	19	10	25	38
Rbm47	-0.77063	6.733602	7.834	0.005127252	0.28432464	30	47	47	11	27	20
Lrtm2	-0.76496	3.932681	4.488106	0.034131473	0.683773541	20	48	32	3	33	14
2010300C02Rik	-0.76127	3.176907	4.669096	0.030710081	0.657286399	36	50	33	10	20	25
Spp1	-0.75942	3.891684	6.141818	0.013202155	0.441083167	373	396	492	97	371	163
Epn3	-0.75918	7.568545	38.06382	6.8468E-10	1.63684E-06	977	1095	1121	303	641	527
Yes1	-0.75847	3.749078	4.426135	0.035392565	0.694145031	128	213	169	47	60	114
Gm765	-0.75825	4.301888	6.769042	0.009275259	0.384537952	627	470	620	169	355	268
Hapln4	-0.74132	4.705497	12.94739	0.000320368	0.046891447	725	791	783	210	486	387
Fnip2	-0.73915	3.943156	5.435632	0.019729953	0.543546322	107	74	62	21	62	37
Hps5	-0.7368	3.951464	7.680206	0.005582978	0.290855605	223	197	312	88	152	92
Pla2g4b	-0.73526	3.367335	5.494492	0.019076473	0.533051164	264	288	267	66	267	90
B3gat1	-0.73404	3.114001	4.023972	0.044857962	0.765395644	103	218	115	49	79	67
Naa40	-0.73368	3.936953	8.913765	0.002830292	0.19402479	113	130	153	42	102	46
Gda	-0.73061	8.16427	38.60256	5.19508E-10	1.39722E-06	1194	1605	1607	407	957	723
Baiap2l1	-0.72703	4.705751	13.06225	0.000301308	0.044403681	306	269	371	81	265	127
Car2	-0.72019	5.064846	10.33824	0.001303025	0.124864452	831	1157	954	283	529	547
Chchd5	-0.71992	3.702736	5.314134	0.0211531	0.560505061	199	204	118	49	138	72
Clec2l	-0.71635	5.551773	15.82744	6.9389E-05	0.015551802	1254	1453	1324	306	832	832
Ppp2r1b	-0.71461	4.627408	4.430862	0.035294688	0.692883681	19	41	80	8	39	23

2410131K14Rik	-0.71135	3.764718	7.403685	0.006509042	0.318292152	245	292	306	58	259	124
Cpt2	-0.70941	4.028405	5.88442	0.015275424	0.480505892	348	256	301	75	146	210
A2m	-0.70694	8.856523	8.141756	0.004325738	0.255371077	238	163	103	31	139	95
Ptpn18	-0.70332	3.616733	3.999063	0.045525556	0.773960303	110	120	49	17	69	58
Wfs1	-0.6987	6.053688	16.30706	5.3863E-05	0.013249948	317	397	437	93	225	241
Fndc5	-0.69672	5.453532	14.08588	0.00017465	0.029823541	1061	1251	1378	317	887	630
Lhfp	-0.68832	3.833689	6.328793	0.011879317	0.429066966	157	336	354	61	205	160
Heatr2	-0.68638	3.351604	3.909014	0.048027765	0.789126837	76	156	107	27	118	35
Zfp101	-0.68411	2.979773	4.761299	0.02910658	0.639273059	109	125	169	38	106	58
Ccdc61	-0.68306	3.024656	4.379682	0.036369776	0.699939273	106	69	94	28	69	38
Sel1l3	-0.68179	4.15808	9.372559	0.002202578	0.168124366	356	405	497	116	254	241
Slc25a37	-0.68041	4.898957	6.589025	0.010260934	0.402023377	417	365	430	166	292	112
Celsr1	-0.67675	4.733599	5.65683	0.017387525	0.51247945	56	67	93	11	79	27
Ppp1r13l	-0.67596	5.858998	5.659376	0.017362296	0.51243781	37	36	33	10	22	21
St3gal5	-0.67416	6.281409	18.34864	1.8395E-05	0.006282338	1340	1051	1182	362	949	501
Kcns1	-0.66804	4.098281	6.533948	0.010583478	0.408089822	368	441	381	115	232	232
D17H6S53E	-0.66739	2.861429	4.360067	0.036790857	0.700860741	75	95	112	22	81	44
Ahcyl2	-0.66453	4.486229	7.605003	0.005820658	0.297475712	449	669	444	137	325	314
Hyou1	-0.66356	3.956475	9.393817	0.002177184	0.167900698	203	287	240	58	201	121
Gyg	-0.66023	8.284297	17.02137	3.69614E-05	0.010893995	1403	1195	1451	372	1039	670
Prps2	-0.65319	4.219853	7.854402	0.005069712	0.282590488	206	247	311	52	195	154
Ntng1	-0.65282	6.370323	22.26392	2.3763E-06	0.001310988	2041	2084	2603	639	1496	1232
Hhatl	-0.64943	3.996539	6.873048	0.008750528	0.375155914	445	391	524	137	305	238
2610015P09Rik	-0.64933	4.762472	6.572268	0.010357989	0.404469144	212	189	250	43	147	150
Plekhn1	-0.64689	6.587936	4.760665	0.029117289	0.639273059	30	32	44	11	17	25
Abca1	-0.64562	4.430037	7.71396	0.005479543	0.289825134	380	397	649	125	371	242
St5	-0.64539	4.25842	6.067476	0.013769362	0.450930897	199	280	217	75	145	122
C2cd3	-0.64486	3.712878	4.946156	0.026148752	0.610213171	153	142	126	46	98	68
Rnf144b	-0.64118	5.028001	4.723663	0.029750307	0.649196356	89	63	85	18	32	68
Ttc37	-0.64012	3.156465	4.088322	0.043180483	0.752617461	73	93	115	22	66	57
Mrps31	-0.63438	3.761437	3.860546	0.049434236	0.798973908	189	362	217	67	241	105
Naf1	-0.63358	4.009279	5.44286	0.019648475	0.542873892	141	205	225	63	102	111
Mab21l2	-0.62526	3.812741	7.432074	0.006407144	0.316223999	332	422	412	106	327	185
Gprc5c	-0.62401	5.502815	11.59705	0.000660567	0.078092049	958	1246	1163	293	886	600
Mrpl22	-0.62307	3.395299	4.855128	0.027564072	0.621772341	184	133	203	46	106	115
Plagl2	-0.62083	3.499136	4.121028	0.042353327	0.748172569	43	63	101	20	53	34
Ncan	-0.6186	3.823312	5.237173	0.022109178	0.574915158	301	505	397	138	296	171
Cdk8	-0.61733	3.77077	4.625462	0.031500769	0.662532303	89	117	182	28	135	53
Nhlrc1	-0.61483	3.119676	4.282573	0.038505025	0.711136587	210	197	155	46	157	103
Gabrg1	-0.61397	4.134488	5.395569	0.020187944	0.549441328	320	631	554	122	400	279
Slc10a7	-0.61192	3.867414	4.18258	0.040841475	0.732898399	127	120	123	45	80	61
Esrra	-0.61064	4.1872	5.156347	0.023161561	0.58316509	161	264	181	52	186	92
Plch2	-0.60991	5.171288	5.360285	0.020600413	0.553142638	49	51	46	18	42	17
Lrrc20	-0.6078	4.926939	10.4987	0.001194588	0.11844583	532	540	452	154	359	280
Chst2	-0.60481	5.591271	9.42502	0.002140447	0.167681269	1243	1160	1341	344	854	762
Tysnd1	-0.60386	4.345828	6.461027	0.011026592	0.418402432	264	207	264	84	148	139
1110038D17Rik	-0.60327	3.648119	6.027249	0.014086673	0.457184402	128	158	140	36	115	78
Rfxap	-0.60253	3.790566	5.209776	0.022460228	0.578055343	235	257	252	51	208	153
Arhgap22	-0.60072	4.231118	5.32079	0.021072454	0.559746819	255	194	230	79	154	115
D430041D05Rik	-0.59902	4.551742	4.777301	0.028837283	0.635720257	569	568	854	141	499	446
Tmem56	-0.59462	4.440873	6.984435	0.008222162	0.360301504	469	713	634	164	434	359
Bai1	-0.59456	3.915373	6.227293	0.012579589	0.433059906	423	465	369	119	348	213
Rnf128	-0.59024	5.139289	5.365662	0.020536995	0.553142638	991	876	976	292	853	404
Pcdh7	-0.58947	6.880277	19.54598	9.82072E-06	0.00386348	1653	2303	2238	549	1617	1164
1700106N22Rik	-0.58806	3.338735	4.509452	0.033708046	0.67948957	152	213	242	53	162	112
Galm	-0.58683	3.964034	4.853863	0.027584294	0.621772341	153	167	127	57	84	83
Cwc22	-0.58603	3.787488	4.519199	0.033516522	0.67948957	94	124	94	30	108	36
Bckdk	-0.58054	5.300544	13.07016	0.000300038	0.044403681	331	359	361	112	238	201
D8Ertd82e	-0.58031	2.802229	3.858405	0.049497362	0.798973908	105	138	127	36	117	51
9030425E11Rik	-0.578	6.010994	6.672983	0.009788524	0.395028775	1487	889	1500	336	894	869
Uba7	-0.57779	4.609596	4.872848	0.027282442	0.62042549	98	77	78	16	90	40
Zdhhc5	-0.57699	6.164018	9.266462	0.002333881	0.173757006	209	266	253	85	184	114
8430410A17Rik	-0.57656	4.122466	6.623938	0.01006171	0.400833293	230	222	227	69	161	131
Nprl2	-0.57412	4.418016	6.765229	0.009295099	0.384537952	409	339	301	100	270	202

Dclk2	-0.57332	5.364602	6.257925	0.012363893	0.429066966	1069	1205	1192	356	878	614
Eif2s3y	-0.57159	4.316655	4.38095	0.036342742	0.699939273	113	141	115	25	74	100
Krt222	-0.57154	3.879331	6.431784	0.011209592	0.41872495	388	386	398	111	285	235
Tuft1	-0.56918	6.423021	5.122278	0.023620584	0.586182792	33	77	47	11	52	24
Stxbp2	-0.56382	5.566527	3.973032	0.046234441	0.779758933	75	95	81	47	40	34
Steap3	-0.56235	5.085903	5.196227	0.022635963	0.579064072	325	340	385	83	238	252
1700020I14Rik	-0.56167	4.832564	9.033727	0.002650436	0.190089255	377	350	342	114	296	174
Cidea	-0.55973	4.253781	4.386851	0.036217142	0.699504517	447	528	520	175	422	205
Arl5b	-0.55863	4.858772	8.969585	0.00274511	0.193396506	195	196	208	63	163	101
Rrm1	-0.55854	5.418382	6.131796	0.01327719	0.441546373	152	227	250	63	202	85
Pcyox1l	-0.55411	6.544386	7.480475	0.006237163	0.311366123	2238	2835	2735	822	2554	1002
Cdk5rap2	-0.55388	5.42262	8.757272	0.003083699	0.203524147	696	719	759	226	619	352
2810432D09Rik	-0.55273	4.435483	7.01126	0.008099865	0.357857709	293	246	302	86	198	170
Gsta4	-0.54799	11.22333	11.6776	0.000632571	0.076035719	183	187	227	45	177	115
Amigo2	-0.54759	4.576499	4.128582	0.042164678	0.746732391	621	583	726	284	408	266
Srr	-0.547	5.238017	12.84102	0.000339103	0.047961082	784	786	819	259	660	391
Rab22a	-0.54493	4.717506	8.953839	0.002768871	0.193396506	307	321	330	107	237	173
St3gal1	-0.54451	3.338384	4.343222	0.037156555	0.704024823	95	146	169	36	119	74
Fgf9	-0.53934	4.025453	4.825971	0.028034078	0.625058265	344	486	379	103	379	214
Stk24	-0.53915	7.19001	15.60793	7.7927E-05	0.016664322	579	682	582	146	516	386
Traf3	-0.53831	4.716219	7.726526	0.005441534	0.289802085	324	403	434	99	323	232
Nexn	-0.53559	5.239818	6.258587	0.012359277	0.429066966	570	914	761	167	602	509
Pdk3	-0.53518	4.780704	5.539716	0.018589601	0.529766703	483	555	619	183	479	253
Plekhg1	-0.53342	5.669586	5.442272	0.019655083	0.542873892	178	216	322	81	244	79
Scx	-0.53311	3.884689	4.102776	0.042812855	0.750131423	289	382	351	84	364	158
Wnk4	-0.53201	4.548959	4.354157	0.036918717	0.702336979	52	70	97	21	72	31
5930434B04Rik	-0.52996	5.40403	11.97852	0.000538173	0.068924538	504	596	580	179	407	329
Galnt10	-0.52838	4.236812	5.283096	0.021533424	0.56709077	279	246	256	82	178	166
Dcun1d2	-0.52699	3.598628	5.1444	0.023321466	0.58316509	253	272	272	81	217	147
Slit2	-0.52535	5.140411	9.756527	0.001786872	0.151577456	470	442	511	154	360	269
H2-Ab1	-0.52329	5.548913	5.098279	0.02394959	0.590939657	1146	1051	943	302	1073	477
Mrpl50	-0.52265	4.216287	5.820956	0.015836337	0.490265651	251	341	354	81	255	199
Ggh	-0.52192	4.051077	4.462103	0.034654798	0.687852983	153	215	232	41	187	124
Zfp36l2	-0.5198	4.651468	5.177881	0.022876207	0.579064072	139	197	190	35	188	92
Gpr64	-0.51878	4.688271	5.631251	0.017643032	0.51579438	616	611	816	223	538	366
2310016C08Rik	-0.51792	3.92452	5.3674	0.020516539	0.553142638	265	294	255	91	205	152
Sphkap	-0.51547	4.99345	5.141533	0.023360018	0.58316509	243	396	428	104	219	252
Abcg1	-0.51512	5.313978	7.551722	0.005995276	0.302803654	396	577	592	140	435	314
Nrarp	-0.51243	5.585225	5.331218	0.020946746	0.558476062	248	396	311	65	354	164
Lrrc4	-0.51203	4.548596	4.098426	0.042923144	0.751185748	150	192	284	59	176	119
Kcnj10	-0.5089	4.442453	6.030592	0.014060021	0.457184402	547	625	625	180	564	295
Accn2	-0.50593	5.143399	7.804959	0.005210305	0.285981922	869	914	1064	337	662	540
Col23a1	-0.50474	5.295223	6.506635	0.010747269	0.412925444	692	677	859	207	635	442
Plin3	-0.50062	6.642181	7.930163	0.004861741	0.27505833	913	1160	896	400	804	409
Abhd6	-0.50045	5.28639	8.387321	0.003778474	0.236040708	794	657	691	204	597	438
Bet3l	-0.50041	5.667491	9.289298	0.002304964	0.17220005	1362	1476	1378	414	1082	892
Iqsec2	-0.49772	4.961038	3.891326	0.048536078	0.790539183	495	475	475	176	437	205
Rgs12	-0.49514	6.571433	11.92802	0.00055296	0.070399334	296	298	328	82	263	189
A2ld1	-0.49483	5.117557	9.75393	0.001789397	0.151577456	643	702	687	191	662	349
Mospd3	-0.49433	5.44945	9.725918	0.001816876	0.151985985	317	362	364	106	267	217
Slc1a3	-0.49201	5.230872	5.556631	0.018410809	0.528108883	711	652	690	272	540	314
D19Ertd737e	-0.49191	5.392344	9.235008	0.002374314	0.175552373	563	551	732	183	525	355
Fn1	-0.49144	6.356955	6.912489	0.008559575	0.370523669	301	319	371	133	300	123
Itga4	-0.48956	3.731045	4.033333	0.044609738	0.763283076	172	268	271	67	205	138
Sv2b	-0.48882	6.571759	14.70052	0.000126011	0.023993469	2349	2914	2644	819	2141	1543
Lrrk1	-0.48843	5.792087	5.791075	0.016107738	0.493649021	165	191	183	74	120	99
Cgnl1	-0.48799	7.087687	15.7257	7.32227E-05	0.015931404	1344	1286	1351	428	1101	748
Vstm2l	-0.4869	6.193843	10.38577	0.0012699	0.123634216	2046	1888	2120	646	1552	1229
Pddc1	-0.4846	5.107609	5.29203	0.021423232	0.564880208	423	438	489	155	449	184
Llph	-0.48399	4.967913	4.649244	0.031067232	0.658484554	208	338	249	65	187	203
Sema3f	-0.48368	4.995584	4.956364	0.025994804	0.608600884	232	154	187	64	132	127
Uba3	-0.48229	5.120152	7.187663	0.007340654	0.340386324	495	560	781	179	566	330
4732418C07Rik	-0.48092	6.26473	8.960917	0.002758165	0.193396506	930	1161	948	334	653	689
Aifm1	-0.48068	5.250957	6.643826	0.009950005	0.399411018	405	653	441	182	389	263

Fam46a	-0.48067	6.689032	6.507523	0.010741906	0.412925444	215	219	283	53	184	182
Galt	-0.47954	4.068482	4.828507	0.027992864	0.625058265	218	206	234	55	235	112
Fam125a	-0.47886	5.270762	6.736017	0.009448536	0.387374414	341	457	352	126	353	189
Dnajb12	-0.47652	4.722505	4.056106	0.044011881	0.75817424	405	329	288	95	347	181
Fam160b2	-0.47536	5.516381	6.996375	0.008167496	0.359369807	433	518	638	132	574	269
4933424B01Rik	-0.47508	4.668234	4.032707	0.044626287	0.763283076	106	214	218	43	146	124
Thoc4	-0.47485	4.773391	5.157081	0.023151776	0.58316509	257	269	349	85	215	200
Pon2	-0.47185	6.538841	11.46117	0.000710654	0.082651033	364	437	353	137	327	199
Dvl2	-0.46864	4.72354	4.463386	0.034628787	0.687852983	190	262	158	79	166	100
Setd7	-0.4684	6.94456	11.55581	0.000675379	0.078975327	1056	1692	1579	458	1094	899
Zfp385b	-0.46691	4.705478	4.674216	0.03061868	0.657286399	586	724	837	214	701	366
Ccbl1	-0.46685	4.562567	4.193136	0.040587962	0.730176079	143	167	163	57	94	110
Efnb2	-0.46587	5.730832	8.817386	0.002983738	0.199373024	409	507	462	125	370	312
Chgb	-0.46361	6.284589	8.730448	0.003129396	0.205053712	1845	2261	2333	677	1534	1438
Fam59a	-0.46281	4.507124	4.448956	0.034922581	0.689745603	239	280	295	59	258	181
Furin	-0.46199	6.519567	9.355657	0.002222983	0.169009514	888	829	875	317	769	414
C330019G07Rik	-0.46144	5.12032	7.169172	0.007416701	0.340386324	444	458	506	134	377	314
Slc24a2	-0.46073	6.966406	4.816002	0.028196674	0.625442919	2774	3522	4021	938	2667	2423
Poldip2	-0.45824	5.375054	10.05791	0.001516955	0.13833339	593	664	601	183	532	383
Slc25a13	-0.45797	5.627121	4.748274	0.029327702	0.642621622	180	150	204	72	167	71
Ppp2r5a	-0.45783	5.144555	5.54464	0.018537368	0.529681287	142	145	168	34	159	86
Senp5	-0.45483	6.097896	10.33517	0.001305194	0.124864452	1214	1399	1344	397	1140	803
Gba	-0.45482	5.458102	6.5457	0.010513796	0.406861212	430	344	418	138	290	254
Dner	-0.45181	6.756299	12.3696	0.000436381	0.059051405	2779	3017	3086	1009	2594	1587
Lin7a	-0.45136	5.426241	5.523615	0.018761451	0.530803048	1072	1369	1082	332	921	822
Sec61a1	-0.45107	7.595994	9.37177	0.002203526	0.168124366	1703	1359	1715	514	1254	1017
Stard10	-0.44506	8.00602	11.88718	0.000565215	0.071485521	2847	2809	2498	905	2441	1500
Uxs1	-0.44366	4.798653	4.939191	0.026254324	0.611068844	463	600	424	166	356	331
Hebp2	-0.44331	7.477163	12.2259	0.000471307	0.062596542	1313	1465	1275	398	1306	765
Pak1ip1	-0.43929	6.379271	12.86291	0.000335159	0.047961082	1464	1611	1464	475	1318	914
Banp	-0.43703	5.349255	4.586083	0.032232601	0.671071484	659	622	448	228	462	305
Ypel2	-0.43582	5.617461	4.851929	0.027615231	0.621772341	227	301	278	90	269	127
Rprm	-0.43555	5.69593	6.328431	0.011881739	0.429066966	1485	1344	1375	493	1285	726
Mrpl34	-0.43433	5.884755	6.29265	0.012123966	0.429066966	927	832	870	331	824	403
Hip1r	-0.43409	6.616377	6.989144	0.008200554	0.360088016	1193	966	1208	391	891	685
Tmem8	-0.43294	4.609333	5.579588	0.018170997	0.524084682	546	696	720	211	581	376
Atp11a	-0.43028	6.140924	5.532451	0.018666943	0.530565321	1544	1728	2008	460	1312	1384
Cd34	-0.43026	4.769977	4.997226	0.025387982	0.603252529	411	491	479	147	420	263
Fbxo18	-0.42972	4.160104	3.968413	0.046361434	0.780526308	229	289	234	80	241	135
1110007C09Rik	-0.42923	6.230562	10.19881	0.00140531	0.131463717	862	1078	1050	321	993	506
BC048546	-0.42841	6.903502	11.07702	0.000874043	0.09594856	3112	3471	3190	1005	2931	1978
Tmem229b	-0.42806	6.653288	10.06953	0.001507415	0.13833339	2630	2600	2742	872	2084	1736
Iglon5	-0.42802	4.963434	5.974143	0.014517132	0.467590721	777	850	768	266	693	469
Mfsd1	-0.42801	6.720076	10.02176	0.001547013	0.13985517	1118	1039	1043	339	1053	572
Wipf3	-0.42796	6.986785	7.787558	0.005260726	0.286532113	3035	3241	4019	1067	3178	1989
Rhobtb2	-0.42594	4.804691	5.375292	0.020423917	0.553142638	615	595	591	217	511	335
Mif4gd	-0.42466	5.295264	3.859817	0.049455732	0.798973908	117	138	219	43	119	119
Ift122	-0.42269	5.768149	8.761056	0.003077307	0.203524147	954	1341	1188	368	1090	652
Gyk	-0.4223	5.751856	5.332651	0.020929531	0.558476062	275	358	286	83	260	215
Plbd2	-0.42136	6.175019	7.929805	0.004862704	0.27505833	1552	1475	1649	562	1233	937
Glt25d2	-0.41993	5.135312	5.362704	0.020571852	0.553142638	360	416	493	106	422	267
Pick1	-0.41795	4.886029	4.213396	0.040105964	0.728202461	428	575	575	178	563	231
Cops3	-0.41791	5.806749	7.813874	0.005184665	0.285981922	703	801	955	245	768	492
Pfdn2	-0.41694	6.004306	4.748166	0.029329542	0.642621622	841	1420	1411	387	1288	591
2310035K24Rik	-0.41676	4.492014	4.489606	0.034101536	0.683773541	263	375	326	96	342	163
Hopx	-0.41652	7.826445	4.843994	0.027742567	0.62298708	1097	1862	1359	420	1383	857
Phyhipl	-0.41565	4.685862	4.170438	0.041135152	0.735713998	644	621	618	184	652	350
Zyx	-0.41503	5.519334	6.243815	0.012462776	0.430862859	619	695	633	217	560	392
Tmem132c	-0.41403	5.059834	4.537169	0.033166393	0.67576525	138	255	243	71	185	123
Ust	-0.41368	4.559284	4.392378	0.036099923	0.698343917	505	615	652	193	593	303
Fam98a	-0.41357	4.147836	4.136472	0.04196854	0.746732391	293	301	278	102	228	186
Jag1	-0.4124	6.287081	4.86214	0.02745227	0.621574019	271	275	451	91	370	171
Lrp11	-0.41211	5.357404	5.759465	0.016400066	0.495595262	1073	1035	1177	314	938	761
Slc6a17	-0.41065	4.836993	4.041938	0.044382831	0.761739563	634	840	834	202	721	517

Polr2b	-0.40661	5.601688	7.432656	0.006405073	0.316223999	624	609	590	190	574	363
Ccz1	-0.40578	6.662536	10.07003	0.001507004	0.13833339	1103	1419	1349	453	1227	669
9030409G11Rik	-0.40507	6.514325	6.604264	0.010173483	0.401983424	665	711	635	252	610	347
Hipk3	-0.40262	6.034363	5.233423	0.022156891	0.574915158	402	535	520	156	396	323
Nqo2	-0.4009	5.145228	5.247806	0.021974466	0.573635513	511	589	628	224	442	341
Tbx3	-0.40062	5.230213	4.628091	0.031452543	0.662165283	774	1137	1136	289	865	711
Rhobtb3	-0.39933	5.144569	4.424227	0.035432163	0.694145031	644	927	1097	275	833	531
Htra1	-0.39717	6.427287	5.66181	0.017338226	0.51243781	1483	1382	1624	472	1153	1082
Dnajc11	-0.39617	5.62893	5.444924	0.019625264	0.542873892	514	595	574	153	556	357
Gmpr	-0.39599	6.765665	8.783436	0.003039782	0.202488976	1182	1095	1049	411	932	654
Pelo	-0.39525	5.233614	5.293766	0.021401893	0.564880208	580	633	607	210	521	371
Dnajb4	-0.39482	5.431708	6.957087	0.008348783	0.362893783	635	858	658	238	641	435
Lrrn1	-0.39468	5.016726	4.450606	0.034888872	0.689745603	682	976	956	309	790	479
Mink1	-0.3942	5.123471	3.877253	0.048944572	0.793588094	309	359	409	137	277	219
Lgi3	-0.39306	6.020917	5.672218	0.017235646	0.511506439	1715	1513	1993	577	1383	1188
Rdbp	-0.39145	5.631583	5.863878	0.015454709	0.483319069	452	561	472	144	492	301
Stam2	-0.38982	5.392178	4.971156	0.02577341	0.60700438	321	350	324	102	356	175
Osbpl9	-0.38913	6.305513	7.179236	0.007375211	0.340386324	895	867	806	269	747	573
Edil3	-0.38848	5.849651	6.034532	0.014028673	0.457184402	1227	1701	1738	506	1321	1007
Gas6	-0.38797	7.053401	4.705827	0.030060541	0.653315766	1292	1896	2106	518	1991	902
Gtf3c6	-0.38751	6.085371	6.104688	0.013482375	0.446287344	580	699	596	262	522	323
Atp2b2	-0.38641	6.810701	5.403206	0.020099801	0.549185963	2868	2918	3238	926	2790	1930
Atf6b	-0.38569	6.076923	4.857396	0.02752786	0.621772341	1120	1094	1120	431	1162	473
Abcg2	-0.38408	5.450239	5.780591	0.016204092	0.493649021	736	824	749	280	715	421
Ahsa1	-0.38374	5.428184	5.421878	0.019885954	0.546444679	685	841	740	253	825	362
Kctd11	-0.38368	5.586181	4.083622	0.043300747	0.752617461	208	202	233	76	182	134
S100b	-0.38363	10.6812	6.636584	0.009990534	0.400291104	47979	42546	41299	16699	39255	24091
Spnb1	-0.38353	6.019232	4.366178	0.036659124	0.700170206	1358	1576	1986	590	1494	907
Ednrb	-0.38353	6.471864	7.151442	0.007490372	0.340724832	1022	1144	1267	436	850	733
Triap1	-0.38303	6.661905	6.856983	0.008829549	0.376191245	1351	1242	1059	435	1200	647
Gdap2	-0.38157	4.864327	3.916811	0.047805489	0.789126837	491	480	478	122	481	335
Slc25a33	-0.3815	4.310894	4.175107	0.041021983	0.734911734	424	535	501	153	446	309
Vamp1	-0.3797	7.747926	8.887213	0.002871747	0.194916462	5567	5738	5877	1774	4917	3980
Mtap7d2	-0.37944	8.705989	8.226569	0.00412815	0.24672577	9655	12425	11747	3473	9740	7733
Med27	-0.37936	5.08929	4.678475	0.030542856	0.657228793	563	625	783	257	530	383
Nefh	-0.37915	10.64622	7.580606	0.00589996	0.300102906	40287	45083	44059	13690	34490	31002
Ccdc92	-0.37804	7.73946	7.786618	0.005263462	0.286532113	5197	5782	6000	1771	5262	3617
Adarb1	-0.3776	5.348323	3.895975	0.048401929	0.790161592	742	748	946	256	711	544
Ak2	-0.37719	6.326938	5.100023	0.023925528	0.590939657	530	521	494	169	563	260
Abhd12	-0.37562	7.624714	10.23173	0.001380454	0.130845159	3478	3481	3661	1104	3172	2376
Tac1	-0.37537	7.010231	8.31921	0.003922797	0.2418421	3037	3847	3507	1153	2888	2314
Itm2c	-0.37438	8.252254	7.206357	0.007264579	0.338321803	4913	6546	5376	1830	6020	2924
Dnm2	-0.37366	6.414357	6.297859	0.012088389	0.429066966	457	413	527	188	368	280
Ermp1	-0.37327	6.760791	7.786467	0.005263904	0.286532113	995	1047	999	348	779	718
Wdr61	-0.37119	6.221713	5.617976	0.017777169	0.517582641	656	758	649	262	494	469
Satb1	-0.36975	6.646716	9.811073	0.001734643	0.149889878	1116	1335	1528	451	1222	797
Foxj2	-0.36945	5.060643	5.227588	0.022231335	0.574915158	397	504	510	174	443	254
Map3k5	-0.36871	5.873733	6.175476	0.012953304	0.438902831	416	428	558	159	449	270
Mrpl4	-0.36812	5.512222	5.10776	0.023819048	0.589069699	673	709	639	181	629	489
Scn1b	-0.36793	7.615279	7.162416	0.007444684	0.340386324	5325	4937	5414	1934	4917	2870
Fam108c	-0.36792	7.094615	5.58851	0.018078669	0.522878739	649	665	782	293	677	311
Zfp365	-0.3675	5.158557	4.195712	0.040526329	0.730176079	817	1089	918	295	775	677
Fbln2	-0.36731	5.353293	4.02979	0.044703514	0.763972048	719	827	1126	275	870	549
Lias	-0.36718	6.176016	6.464096	0.011007561	0.418402432	1168	1252	1108	365	1135	749
Elovl5	-0.36691	6.892491	9.178914	0.002448192	0.17856036	2051	2436	2462	768	2191	1403
Fem1a	-0.36628	5.399362	4.277038	0.038630606	0.712844004	506	568	624	151	589	367
Usp10	-0.36557	5.914596	4.403446	0.035866392	0.696481318	522	486	410	118	518	308
9530068E07Rik	-0.36551	6.262039	4.473822	0.034417916	0.686567536	703	986	1098	334	909	489
Ikbkb	-0.36531	5.274238	4.000209	0.045494617	0.773960303	341	325	348	93	282	265
Dapk1	-0.36511	6.952786	6.10047	0.013514594	0.446666658	986	1215	1402	339	1282	725
Trib1	-0.36393	7.715944	7.005785	0.008124676	0.358218299	436	494	571	170	439	322
Mkrn1	-0.36143	7.110772	6.47035	0.010968891	0.418402432	1035	1097	1300	451	1012	628
Cds1	-0.36131	6.367619	6.88584	0.008688124	0.374616583	984	1106	1218	350	933	778
Ablim1	-0.36131	8.789028	9.58412	0.001962675	0.162418922	1880	2230	2752	732	2179	1424

Ogfrl1	-0.35998	7.52704	6.049103	0.013913366	0.454263999	4064	4693	4461	1381	3902	3046
Tmod3	-0.35952	7.224406	6.268573	0.012289806	0.429066966	498	487	500	195	408	303
Cerk	-0.35886	6.275889	4.80994	0.028296024	0.627000257	1031	1405	1530	358	1352	874
Hsdl1	-0.35795	5.682591	5.23498	0.022137061	0.574915158	858	972	979	298	813	651
Rapgef1	-0.35701	6.604899	5.137253	0.023417672	0.58316509	1183	992	1123	396	1175	549
Rela	-0.35622	5.940889	5.638311	0.017572126	0.51579438	591	495	532	207	501	303
Cpne6	-0.35563	7.787723	6.150103	0.013140442	0.440389035	6092	5723	5798	2059	4820	4006
Fbxo21	-0.35532	5.444392	5.580093	0.018165754	0.524084682	788	1061	1003	300	914	601
Ythdf3	-0.35494	6.531229	5.835969	0.015701762	0.486937254	1011	1053	1224	434	1084	530
Arl4c	-0.35286	5.520069	5.948536	0.014729514	0.470906708	824	869	863	293	873	472
Apbb3	-0.35134	5.185567	4.560649	0.032714683	0.671717756	847	885	970	260	977	547
Dexi	-0.351	5.897642	5.767931	0.016321241	0.494602561	1269	1112	1193	356	1219	766
Stag1	-0.35087	5.226783	4.991251	0.025475789	0.603252529	363	436	383	149	357	230
Mrps23	-0.35083	6.182256	6.87335	0.00874905	0.375155914	1063	958	976	358	919	612
Tnks1bp1	-0.35006	6.591871	6.296795	0.012095644	0.429066966	744	837	982	306	830	481
Lrrc49	-0.34844	7.376393	4.572365	0.032491672	0.671233175	4010	4472	4436	1560	4030	2518
Alad	-0.34834	5.718604	4.299281	0.038128492	0.709286269	419	385	377	114	432	236
Mef2c	-0.3454	6.128493	4.247005	0.039319626	0.719937241	1561	1705	2093	621	1512	1198
Yipf3	-0.34502	5.817459	5.950976	0.014709143	0.470906708	611	815	627	236	670	403
Hadha	-0.34318	7.45382	7.122367	0.007612812	0.344315852	2734	2406	2686	892	2357	1721
Dennd1b	-0.34253	5.584482	3.934321	0.047310249	0.787260109	347	354	372	95	360	251
Eif2s1	-0.34211	5.570055	3.93272	0.047355294	0.787400694	481	441	492	194	345	314
Ganab	-0.33975	6.395233	5.19791	0.022614065	0.579064072	791	890	1036	349	909	460
Ptprd	-0.33972	6.214805	4.110043	0.042629285	0.750131423	606	656	594	242	614	319
Tars	-0.33895	6.147227	5.149466	0.02325352	0.58316509	664	726	705	224	598	507
Cend1	-0.3367	7.371482	6.740351	0.009425607	0.387374414	4022	4485	4673	1572	4139	2648
Prkag1	-0.33551	6.647243	7.162851	0.007442881	0.340386324	1206	1253	1077	420	1103	733
Cdh1	-0.33551	9.220823	8.139072	0.004332146	0.255371077	4151	4676	4979	1476	4901	2700
Bcas3	-0.33455	5.925267	4.338277	0.03726461	0.704024823	1030	917	1243	393	989	636
Cabp1	-0.33405	7.014053	5.377672	0.020396077	0.553142638	3479	3481	3259	1203	3228	2112
Etl4	-0.33301	6.753517	4.318036	0.037710432	0.706159831	773	974	1298	402	816	634
Tusc2	-0.32957	6.400867	6.158427	0.013078745	0.440389035	1735	1909	1652	557	1668	1219
Chmp1b	-0.32912	6.487465	5.170784	0.022969849	0.580069556	540	672	689	243	602	357
Jak1	-0.32882	9.128729	7.693939	0.005540656	0.289825134	7572	8552	7932	2713	6900	5658
Fgf1	-0.32807	7.842607	4.284091	0.038470639	0.711111914	5549	5423	5787	1992	5458	3347
Maf	-0.32745	7.026052	4.636425	0.031300142	0.660464347	2373	3408	3634	901	3379	2001
Vasp	-0.32702	7.482216	7.511891	0.0061293	0.30740795	1258	1403	1557	555	1208	858
Spock2	-0.32317	7.299468	3.998537	0.045539772	0.773960303	3393	4467	4149	1222	3216	3214
Pptc7	-0.32243	7.525937	4.83437	0.027897836	0.624609624	2242	1984	2724	892	2194	1329
Def8	-0.3223	5.797097	4.472517	0.03444422	0.686567536	715	640	613	218	699	389
Ntrk2	-0.32162	7.468204	7.214015	0.00723365	0.338321803	2488	2832	3386	983	2662	1939
Nkiras1	-0.32067	5.476573	4.932285	0.026359435	0.611068844	948	1069	1143	335	1042	694
Tra2a	-0.32041	5.599692	3.893432	0.048475259	0.790161592	465	471	506	172	416	329
Laptm4b	-0.32035	7.100176	7.427723	0.006422657	0.316223999	2151	2485	2201	809	2047	1510
Timp2	-0.31913	11.3274	5.179337	0.022857044	0.579064072	55428	61307	57176	22127	51011	36326
Purb	-0.31855	8.898474	6.420019	0.011284093	0.420038738	3807	4419	5475	1673	4700	2490
Pik3r1	-0.31808	7.799679	8.942288	0.002786434	0.193396506	3469	4092	4313	1374	3812	2489
Pdrg1	-0.31718	7.238998	4.242204	0.039430945	0.720812417	2484	2443	2038	827	2148	1524
Arl3	-0.31716	7.044732	5.071918	0.024316468	0.595214034	2708	2456	2903	958	2691	1611
Cwc25	-0.31682	5.789951	4.136387	0.041970666	0.746732391	623	676	679	278	661	320
Rexo2	-0.31641	7.740989	5.182451	0.022816122	0.579064072	3234	3986	3515	1384	3608	1905
Fstl1	-0.31513	10.49699	7.121305	0.007617324	0.344315852	33552	37484	38162	12368	34026	24355
R3hdm2	-0.31468	6.955593	5.612758	0.017830174	0.518424343	949	1199	1436	400	1167	762
Wdtc1	-0.3139	5.784642	5.523697	0.018760567	0.530803048	561	586	656	198	595	389
Pgd	-0.31315	7.008613	7.045032	0.007948539	0.354080252	838	816	865	290	801	549
Sfrp5	-0.31136	7.15662	4.683181	0.030459304	0.657228793	3063	3747	4517	1360	3918	2107
Ddx47	-0.31129	6.270706	5.702719	0.016938655	0.506887471	820	981	935	333	975	489
Usf2	-0.31115	8.053986	4.291137	0.03831154	0.710000942	3190	2722	2957	1130	3220	1500
Crbn	-0.31108	6.145904	6.416995	0.011303329	0.420038738	1456	1797	1561	600	1457	1016
Slc25a11	-0.31031	6.238116	3.920629	0.047697043	0.789126837	1032	951	962	326	814	757
Gmcl1	-0.31006	5.856453	3.910968	0.047971972	0.789126837	646	840	953	284	879	445
H2afj	-0.3087	5.801781	3.901485	0.048243459	0.789653935	433	507	558	173	487	316
Psmc4	-0.30866	6.627284	4.302029	0.038066931	0.709286246	1452	1383	1388	392	1573	941
Sacm1l	-0.30654	6.451479	5.952191	0.014699007	0.470906708	1318	1568	1656	507	1438	1020

Tyro3	-0.30643	6.356616	4.051744	0.044125742	0.759527579	1035	1041	1004	415	966	588
Lancl1	-0.30625	6.793614	4.619079	0.031618212	0.663057945	1343	1376	1274	468	1322	835
Pbx1	-0.30299	7.806229	4.442604	0.035052737	0.690654475	1559	2023	2290	660	2194	1081
Golim4	-0.30275	6.651566	4.978264	0.02566773	0.6062205	1017	1321	1500	395	1214	918
Usp4	-0.30251	6.15387	4.99601	0.025405829	0.603252529	453	618	563	213	513	323
Arap2	-0.29616	7.583157	4.46825	0.034530338	0.686784085	1783	1683	1736	655	1416	1243
Sgms1	-0.29595	5.932444	4.134782	0.042010473	0.746732391	475	626	600	205	517	379
Sod2	-0.29591	6.606777	5.0334	0.024863047	0.600939511	896	951	1052	335	1065	547
Lman1	-0.29563	7.098669	5.042876	0.024727401	0.599616062	1149	1008	1163	403	1186	628
Sh3gl2	-0.29547	7.67439	6.441534	0.011148234	0.41872495	2627	2864	3069	1101	2664	1753
Extl3	-0.29444	6.048178	3.959291	0.046613321	0.781708664	1028	982	1285	403	958	761
Enah	-0.29388	7.903459	5.359345	0.020611511	0.553142638	4843	5583	6530	1937	4840	4175
Crnkl1	-0.29358	5.978761	3.851609	0.049698307	0.799812338	516	511	416	183	494	278
Cbara1	-0.29349	6.915406	4.917915	0.026579576	0.612954074	1376	1518	1554	454	1528	1023
Snx8	-0.29219	6.717619	4.820636	0.028120962	0.625442919	416	480	445	151	442	296
Tex261	-0.29161	6.754387	5.153825	0.02319523	0.58316509	924	1086	1244	376	1139	653
Nceh1	-0.29076	5.858417	4.514919	0.033600486	0.67948957	1264	1485	1495	483	1317	988
Cnih4	-0.29074	6.632013	3.951241	0.046836809	0.784844843	946	849	852	355	900	484
Ppp2r2b	-0.29021	6.996683	5.081615	0.024180836	0.592568192	2948	3239	3690	1257	3342	1878
1110008P14Rik	-0.28885	6.924911	3.944724	0.047018571	0.785443766	2704	2293	2011	763	2450	1567
Atp1a1	-0.28835	11.10697	5.280156	0.021569811	0.567354599	48756	41955	47021	16815	46651	28207
Ubl5	-0.28816	8.110408	5.733227	0.016646868	0.501644289	2724	2847	2973	958	3002	1802
Rcc2	-0.28671	7.04893	4.663636	0.030807892	0.657286399	1318	1854	1576	545	1742	915
Bod1	-0.28483	6.257737	4.413663	0.035652203	0.694145031	718	958	828	324	707	572
Ubac1	-0.2836	5.960715	3.857073	0.049536679	0.798973908	1152	1072	1156	461	983	724
Ndel1	-0.28227	6.683121	4.439278	0.035121103	0.6913684	1468	2058	1581	551	1778	1126
Zcchc6	-0.28197	6.702254	4.047361	0.044240453	0.760893348	722	680	837	269	724	490
Psmd2	-0.28142	8.439741	5.646857	0.017486688	0.514697105	5303	5855	5996	1988	5257	4064
Gabra1	-0.27964	6.649995	4.094904	0.043012681	0.751185748	2572	2589	2653	812	2697	1833
Sdha	-0.27942	7.263249	5.778764	0.016220945	0.493649021	2243	2535	2503	852	2243	1709
Tbcel	-0.27874	6.466864	3.978854	0.046074891	0.779361121	1593	1643	1795	555	1621	1196
Kcnb1	-0.27858	6.467244	4.91833	0.026573187	0.612954074	2020	2234	2568	773	2196	1576
Stx12	-0.27804	6.972732	6.373855	0.011581374	0.42523009	1957	2107	2129	728	2077	1340
Thsd7a	-0.27748	6.670733	4.563408	0.032662019	0.671717756	2521	2560	2849	1011	2422	1741
Zdhhc3	-0.27743	6.193234	5.212337	0.022427175	0.578055343	1084	1132	1052	359	1078	766
Smg7	-0.27635	7.764302	3.963663	0.046492433	0.781569653	3143	2964	2878	1155	3027	1794
Eif1b	-0.27556	8.169394	4.085874	0.043243089	0.752617461	6881	7075	6784	2535	7123	4259
Ndufa10	-0.27452	8.011833	4.183577	0.040817466	0.732898399	4889	4298	4677	1559	4438	3339
Ubl4	-0.27431	6.203808	3.921382	0.047675684	0.789126837	832	917	767	338	728	562
Epb4.1	-0.27267	6.98935	4.580337	0.032340858	0.671233175	715	788	693	250	675	538
Rab3gap1	-0.26656	7.096749	5.51131	0.018893891	0.532733953	1261	1313	1420	517	1356	793
Sh3bp5l	-0.26575	7.031526	5.786335	0.016151226	0.493649021	1591	1586	1636	601	1608	1018
Efr3a	-0.26504	6.795481	5.088688	0.024082408	0.591685293	1305	1543	1692	570	1402	1019
Nfatc3	-0.26443	7.324117	4.203694	0.040336024	0.730176079	768	1044	856	323	952	532
Eps15	-0.26299	6.543807	4.893571	0.026956875	0.617682769	1452	1756	1873	649	1672	1054
Tom1l2	-0.2616	7.394133	5.062618	0.024447287	0.597057701	3229	3407	3493	1223	3239	2321
Idh3a	-0.26058	6.836121	4.603913	0.031899065	0.666204354	2047	2417	2255	701	2475	1506
Onecut2	-0.25928	7.752759	3.975071	0.046178495	0.779758933	4874	5432	6490	1938	5378	3978
Cdc37l1	-0.25806	7.027351	4.876906	0.027218368	0.620371191	2098	2287	2210	769	2280	1453
Rora	-0.25623	7.852727	5.141315	0.023362948	0.58316509	917	938	1022	338	981	639
Oxr1	-0.25603	8.358467	3.908379	0.048045927	0.789126837	1834	2248	2239	652	2029	1639
Cdk16	-0.25236	7.192495	4.232564	0.039655489	0.723036818	2277	2863	3008	923	2558	2013
Nefl	-0.25202	11.41623	4.13119	0.042099727	0.746732391	65451	72785	74106	24274	65708	53341
Mtus1	-0.25096	7.205547	4.598984	0.031990904	0.666973143	1080	1307	1329	480	1206	797
Psmd3	-0.25064	8.360333	4.635865	0.031310357	0.660464347	3926	4890	4560	1693	4823	2596
Ube3b	-0.24663	6.711615	3.960903	0.046568707	0.781569653	1585	1811	2089	665	1775	1261
Tmem65	-0.24459	6.598744	4.517696	0.033545979	0.67948957	1183	1353	1542	504	1310	933
Rab3d	-0.23731	8.030933	3.89361	0.048470145	0.790161592	1341	1270	1416	486	1489	834
Sec31a	-0.23189	7.013891	3.843126	0.049950314	0.803236887	1358	1575	1606	528	1424	1148
Nfia	-0.22951	8.646096	5.138978	0.023394411	0.58316509	6382	7421	7673	2564	7701	4689
Ttc1	-0.22918	7.035023	3.916047	0.047827204	0.789126837	2042	2459	2432	852	2273	1614
Dnajc8	-0.21995	8.712465	4.265695	0.038889349	0.715165148	3727	4036	3817	1537	3971	2454

Table S7. miR-183C target genes in the TG of SNS-CKO strain.

	log2FC	logCPM	LR	PValue	FDR	WT1	WT2	WT3	WT4	SNS-CKO1	SNS-CKO2	SNS-CKO3
Alk	4.763251	0.997144	12.31975	0.00044819	0.705017	0	0	0	0	1	4	4
Wnt2	3.27212	3.713093	5.037166	0.024809044	1	0	0	0	0	3	0	0
Grhl2	3.10056	5.435743	21.30568	3.9157E-06	0.028174	1	1	0	1	3	16	3
Pyroxd2	2.339075	0.637894	6.08841	0.013607148	1	1	1	0	0	1	5	3
Ky	2.012463	-0.03292	4.576543	0.032412543	1	4	3	0	0	13	5	4
Kcnj14	1.517935	1.270612	4.451155	0.034877653	1	14	2	0	11	9	31	20
Mpv17l	0.912426	2.953134	6.301274	0.012065126	1	28	42	35	35	65	85	49
Nova2	0.87369	2.540211	5.741963	0.016564272	1	28	34	29	21	52	61	41
Zkscan17	0.860446	3.000774	5.973242	0.014524553	1	27	14	11	16	28	43	23
Gria1	0.856213	3.28212	5.260411	0.021815871	1	36	56	39	27	73	72	66
Abcd1	0.815319	3.740933	7.228286	0.00717637	1	35	58	41	45	77	105	56
Slc25a1	0.806006	6.688861	33.58675	6.81554E-09	0.000123	393	408	368	366	712	718	574
Zfp36	0.760423	5.195642	4.177013	0.040975868	1	14	27	5	8	21	27	20
Casp2	0.729204	4.107749	4.770462	0.028952054	1	22	17	7	11	20	30	21
Insig2	0.714522	5.033879	8.213422	0.004158166	1	75	86	91	53	134	147	95
Map3k3	0.704376	5.062255	13.14296	0.000288602	0.586926	54	71	60	32	90	94	79
Phf13	0.622382	4.207486	6.260983	0.012342573	1	32	39	48	38	52	85	46
Zcchc14	0.607889	3.610004	4.788597	0.02864876	1	40	37	30	31	47	54	55
2410016O06Rik	0.602914	4.718502	6.154163	0.013110314	1	56	64	45	46	77	74	85
Spry4	0.557554	2.638308	4.148016	0.041683326	1	35	41	35	30	48	66	42
Tgfbr1	0.537148	5.441946	6.02934	0.01406999	1	38	45	31	30	48	60	48
Cdyl2	0.489115	3.781238	4.650594	0.031042804	1	63	86	74	61	99	137	66
Map2k3	0.482087	6.409921	7.199936	0.007290618	1	61	53	56	84	91	97	78
Cacnb1	0.471412	4.795604	5.37694	0.020404639	1	139	211	192	163	259	286	188
Itga3	0.45997	6.607641	5.237623	0.022103449	1	121	138	137	131	207	193	143
Gap43	0.418973	7.425221	4.741665	0.029440571	1	934	1235	1115	954	1404	1611	1213
Cep170b	0.383124	7.104357	8.058923	0.004528009	1	256	270	303	220	328	438	266
Kdm6a	0.365418	4.810481	4.964838	0.02586774	1	76	95	53	55	86	94	87
Myadm	0.360315	5.594049	5.336025	0.020889051	1	282	295	286	246	331	456	286
Ccdc50	0.356301	6.265366	5.05092	0.024612863	1	177	254	182	196	234	291	244
Ehd1	0.353881	6.72476	5.972607	0.014529788	1	138	153	153	158	188	226	163
Yipf4	0.350781	5.351106	5.128846	0.023531365	1	154	165	157	147	209	244	146
Vps36	0.350155	5.583673	4.160437	0.04137873	1	111	122	94	98	111	162	131
Ddhd1	0.34311	5.22648	5.984518	0.014431987	1	198	215	166	143	230	260	194
Rarg	0.332151	8.127643	5.000894	0.025334232	1	196	168	179	133	190	242	203
Foxo3	0.32971	6.333536	5.586507	0.018099355	1	183	231	215	165	250	266	226
Samd12	0.327291	5.901981	4.586844	0.032218286	1	243	283	225	213	293	372	243
Ppp3r1	0.315657	7.733055	5.356853	0.020640989	1	857	976	951	822	1118	1294	948
Gng12	0.313765	6.722468	5.326487	0.021003682	1	140	160	164	163	194	225	165
Lcor	0.307707	6.386707	4.065448	0.043769079	1	176	156	138	165	194	213	180
Fyn	0.268435	5.919639	5.003538	0.025295562	1	390	434	385	345	393	569	436
2310022B05Rik	0.246041	6.762892	4.563041	0.032669021	1	245	275	249	252	300	329	274
Ldlr	0.230994	8.384465	4.310689	0.037873617	1	524	558	509	510	652	669	522

Table S8. miR-183C target genes in the TG of MS-CKO strain

	logFC	fc	logCPM	LR	Pvalue	FDR	WT1	WT2	WT3	WT4	WT5	MS-CKO1	MS-CKO2	MS-CKO3	MS-CKO4	MS-CKO5
Ankrd52	0.571507	**1.486075**	3.198218	4.63995	0.031235905	1	38	35	32	57	48	49	44	52	66	56
Ap1s3	3.681468	**12.83017**	4.470898	10.72423	0.001057414	0.392181	0	0	0	0	0	5	0	1	0	1
Arhgef37	0.792966	**1.732633**	4.515096	8.591185	0.003377942	0.584254	20	15	8	20	15	25	21	16	32	23
Arid5b	0.318608	**1.247126**	7.382408	3.869055	0.049184215	1	235	216	255	249	350	255	339	291	245	286
Cdv3	0.502886	**1.417045**	5.753538	8.659901	0.003252889	0.579336	65	72	126	106	79	82	94	90	180	91
Cdyl2	0.40683	**1.32577**	3.781238	4.893834	0.026952765	1	64	63	82	117	92	105	115	118	87	53
Cpm	1.004152	**2.005764**	6.715549	15.74284	7.25619E-05	0.159588	19	5	15	19	10	32	20	13	40	12
Ctdspl	0.355317	**1.279266**	6.364332	6.733025	0.009464395	0.931074	105	101	135	150	111	174	124	135	144	85
Cxadr	0.574645	**1.489311**	6.319607	5.781452	0.01619616	1	12	23	34	10	12	17	15	33	31	20
Dagla	0.478684	**1.393472**	3.433676	4.086463	0.043228003	1	48	40	59	67	49	35	64	60	68	80
Dpp8	0.353859	**1.277974**	5.822675	5.878003	0.015331204	1	216	162	282	294	159	188	261	243	224	269
Epha3	3.0724	**8.411717**	-0.53998	6.611307	0.010133329	0.943727	1	0	1	0	0	6	3	0	3	6
Fam160a2	0.350927	**1.27538**	5.220051	5.319093	0.021092992	1	137	68	144	98	107	108	118	139	124	115
Gid4	0.412398	**1.330896**	5.55091	10.52249	0.001179304	0.403928	135	139	205	251	227	199	203	243	230	204
Gpr39	3.470855	**11.08744**	1.85626	8.554106	0.003447439	0.590595	0	0	0	0	0	6	0	0	0	0
Grhl2	2.731065	**6.639456**	5.435743	8.599805	0.00336199	0.584254	0	0	1	0	0	0	4	0	4	1
Irf6	0.644096	**1.562759**	7.435745	6.605153	0.010168408	0.943727	13	7	21	12	21	21	34	13	10	21
Jade2	0.249004	**1.188386**	5.15291	4.148825	0.041663422	1	143	107	167	150	116	164	136	133	157	106
Kcnk10	0.421194	**1.339035**	3.694793	5.326276	0.021006221	1	83	76	96	107	98	123	115	128	91	76
Mast4	0.473108	**1.388097**	7.918613	12.75263	0.000355509	0.274974	105	57	127	134	91	99	108	141	141	112
Mcmbp	0.245098	**1.185173**	5.574388	3.851397	0.049704582	1	113	75	131	112	84	116	120	104	108	75
Mrvi1	0.90423	**1.871545**	1.388283	4.734936	0.029555941	1	18	7	9	10	13	13	19	11	35	16
Myb	1.649466	**3.137175**	2.1592	4.404996	0.035833818	1	0	0	2	0	2	0	5	0	5	2
Nhlrc3	1.847769	**3.599431**	0.835202	4.421084	0.035497469	1	3	0	0	0	0	1	0	2	7	1
Nptx1	0.27269	**1.208058**	6.035924	6.193574	0.012821501	1	406	389	667	820	471	522	662	544	652	419
Nt5dc3	0.465338	**1.380641**	4.86018	10.89673	0.00096334	0.380847	166	154	274	230	165	249	243	238	246	183
Onecut1	0.490874	**1.405296**	3.09934	5.52373	0.018760214	1	74	34	53	71	39	64	73	70	72	48
Prr15l	1.819018	**3.52841**	1.718841	4.976927	0.025687579	1	0	2	0	0	2	0	0	0	12	2
Pygo2	0.280663	**1.214753**	5.979636	5.189519	0.022723508	1	201	153	343	287	206	284	233	297	236	170
Rftn1	0.938247	**1.916199**	1.446897	5.601	0.017950229	1	9	6	19	6	11	15	19	21	17	12
Rhpn2	1.204084	**2.303909**	2.973708	4.149141	0.041655642	1	0	4	0	1	5	2	9	0	5	5
Rims3	0.301665	**1.232566**	5.382683	6.038745	0.013995229	1	245	286	430	488	295	339	365	415	334	347
Ss18	0.394661	**1.314634**	5.096285	5.92813	0.014901049	1	50	54	75	61	73	83	60	78	77	56
Stag1	0.334736	**1.261147**	5.314926	4.490716	0.034079398	1	114	66	105	141	91	138	109	82	133	93
Syne2	0.278218	**1.212696**	7.990027	4.039793	0.044439277	1	228	118	270	270	247	224	213	212	268	238
Tmem178b	0.250889	**1.189941**	5.064478	4.236688	0.039559264	1	218	165	281	311	225	225	237	237	282	225
Zcchc14	0.528393	**1.442322**	3.610003	5.333665	0.020917357	1	28	37	41	52	32	45	62	45	59	26
Zdhhc17	0.291377	**1.223808**	5.185676	5.679081	0.017168356	1	214	203	299	316	247	244	283	287	281	237
Zdhhc5	0.499617	**1.413838**	6.163521	6.501661	0.010777377	0.95735	49	47	83	104	49	55	106	74	76	80
Zfp322a	0.283168	**1.216864**	5.330954	5.987834	0.014404879	1	151	139	207	239	173	155	217	185	209	173

Table S9. miR-183C target genes in the TG of miR-183C KO strain.

	log2FC	logCPM	LR	PValue	FDR	WT1	WT2	WT3	KO1	KO2	KO3
Grem1	5.884219	-1.516145	4.842448	0.027767457	0.62298708	0	0	0	4	7	8
Rd3	5.5858	-0.394397	8.522267	0.003508272	0.225999933	0	0	0	0	10	9
5730559C18Rik	4.992312	4.208334	15.96325	6.45841E-05	0.014627289	0	0	0	8	0	2
Ccdc88c	4.897153	0.612969	11.72676	0.000616079	0.074890147	0	0	0	2	5	3
Irx2	4.812347	0.27136	7.730967	0.005428166	0.289802085	0	0	0	6	0	0
Cdsn	3.850083	3.314134	6.399717	0.011413853	0.421959559	0	0	0	4	0	0
Gdnf	2.237524	1.354751	6.364049	0.011645552	0.426858101	0	4	0	2	14	2
Grhl2	2.025769	5.037748	20.13768	7.20629E-06	0.003298949	0	12	3	0	42	13
Pax6	2.002671	7.376403	15.96391	6.45615E-05	0.014627289	0	7	2	4	11	15
Prr15l	1.878842	1.854081	6.21414	0.012673388	0.433666095	2	11	7	1	18	43
Arsj	1.794172	1.814089	7.651246	0.005673313	0.294405785	4	5	5	4	18	19
Dgkk	1.740272	2.509731	17.34675	3.1143E-05	0.00943765	61	39	80	79	245	171
Slc1a1	1.588348	5.941951	47.25489	6.23303E-12	2.6822E-08	68	54	71	57	321	134
2610018G03Rik	1.559135	2.354186	4.909268	0.026712955	0.61417846	0	10	6	3	34	7
D3Bwg0562e	1.541863	2.647607	17.65493	2.64827E-05	0.00852643	56	83	84	88	270	171
Slc25a1	1.396849	7.409823	77.1076	1.61893E-18	3.48328E-14	1371	1550	1387	1637	4463	3049
Nox4	1.379541	1.504443	5.842953	0.015639557	0.486272706	8	8	4	3	22	20
B4galnt1	1.373513	5.016262	44.8545	2.12232E-11	7.61065E-08	271	375	360	383	1042	665
Nova1	1.243484	2.026995	6.163845	0.013038748	0.440389035	45	59	61	75	158	68
Sv2c	1.18594	5.891348	6.077751	0.013689492	0.44968413	393	1789	436	686	3201	1249
Ccdc14	1.176649	1.829349	4.653245	0.030994893	0.658325037	24	15	19	12	33	61
Nova2	1.153359	2.975271	12.66628	0.000372309	0.052016864	82	108	147	102	346	172
Cacna2d2	1.062124	6.378794	17.71396	2.56735E-05	0.008498322	1202	678	1554	876	3798	1580
Klhl8	1.048649	3.874682	10.76905	0.001032117	0.105246569	142	125	112	122	358	166
Mpv17l	1.004846	2.306796	5.782517	0.016186342	0.493649021	55	72	67	76	94	110
Nck2	0.972307	4.607676	14.63405	0.000130535	0.024422481	51	36	57	35	118	80
Cx3cl1	0.920696	4.615334	11.76339	0.000604072	0.074269844	55	56	95	46	220	72
Lrch2	0.909468	2.984243	7.36243	0.006660071	0.32129615	85	41	76	62	144	97
Slc22a21	0.879361	3.077283	5.022639	0.02501801	0.602257311	25	45	16	22	87	26
Myadm	0.852915	5.721288	20.00361	7.72959E-06	0.003393711	578	569	837	524	1740	716
Ppm1f	0.852244	4.533228	10.8321	0.000997554	0.102886204	312	323	343	242	553	593
Samd12	0.842632	2.607414	4.996243	0.025402401	0.603252529	40	88	52	44	128	88
Lrrc3	0.840543	3.09877	5.915694	0.015006591	0.476941025	169	90	106	112	206	189
Chst10	0.839672	3.943192	7.32095	0.006815538	0.326567584	178	120	190	105	382	249
Gng12	0.788021	6.892978	33.3683	7.62569E-09	1.17196E-05	454	394	443	361	920	510
Npnt	0.776712	6.769446	9.772884	0.001771044	0.151577456	31	46	35	33	56	60
Vat1	0.775364	6.271668	28.95221	7.41861E-08	6.65078E-05	536	685	627	550	1216	700
Zfyve26	0.76864	2.920812	4.43563	0.035196232	0.691683889	40	38	74	36	144	41
Antxr2	0.764308	3.131643	4.394584	0.036053248	0.698343917	89	126	197	83	417	116
Celf6	0.745519	4.249156	10.54927	0.001162343	0.116320796	268	344	393	198	757	459
Gap43	0.739435	8.27836	16.68942	4.40259E-05	0.011990654	5378	6328	4584	4525	10675	6268
Nptx2	0.731622	4.058258	9.488509	0.002067628	0.16661832	279	290	313	174	634	419
Wnt4	0.72951	7.880735	19.53526	9.87597E-06	0.00386348	104	97	120	80	247	110
Psen2	0.722344	6.510016	18.99416	1.31119E-05	0.004864062	1145	1457	1469	1037	2431	1799
Uhrf1bp1	0.713073	3.391685	6.199673	0.01277739	0.43499734	81	69	98	52	159	121
Ldlr	0.706982	8.620816	30.05182	4.20654E-08	4.30991E-05	1957	1891	2028	1420	4224	2223
Itga3	0.697675	6.558744	26.93471	2.10446E-07	0.00015933	322	438	396	254	751	515
Abhd13	0.683757	4.625025	8.705659	0.003172237	0.206829872	97	163	178	110	300	153
Adcy6	0.673935	4.075444	4.324876	0.037559152	0.706159831	90	148	201	89	374	134
Ptpn9	0.629268	4.129905	6.060572	0.013823292	0.452009043	118	210	234	115	347	239
Cebpa	0.627827	4.667934	6.126747	0.013315162	0.442112709	81	87	43	36	145	94
Insig2	0.624959	4.72272	8.05487	0.00453815	0.261077128	168	203	142	104	336	213
Fnbp1l	0.600289	5.599456	8.341358	0.003875263	0.239881444	132	277	315	144	448	294
Braf	0.592707	6.992482	19.95016	7.94874E-06	0.003393711	957	1021	1165	612	1891	1374
Prrx1	0.588297	5.123936	7.291611	0.006927732	0.330503497	67	81	53	35	146	73
Slc44a2	0.586228	6.632697	17.63151	2.68109E-05	0.00852643	719	921	884	480	1461	1142
Snx30	0.565772	4.493714	5.308735	0.021218756	0.5615532	198	214	149	157	265	206
Prkce	0.563963	8.399661	15.08192	0.000102945	0.02013601	5837	6247	6501	4095	10769	7156
Atp2b4	0.563277	5.475423	8.464495	0.003621459	0.229174424	560	597	589	418	967	646
Rgs2	0.559	5.670061	8.894757	0.002859907	0.194727056	689	709	616	522	1051	718
Nkap	0.549996	5.061553	7.924061	0.004878165	0.27505833	243	339	297	226	437	321
Fam160a2	0.541779	5.270316	10.57514	0.001146185	0.115239833	300	309	380	193	641	357
Ppp3r1	0.53954	8.305003	15.3765	8.80768E-05	0.017877937	4522	4217	4458	3095	8443	4007
Ppil1	0.534115	4.971721	4.510382	0.033689711	0.67948957	186	180	125	110	369	121
Rapgef5	0.53361	4.202876	4.523303	0.033436217	0.67948957	46	79	51	29	108	73
Morf4l2	0.531502	4.720721	7.514057	0.006121933	0.30740795	206	339	261	161	475	307

Ccdc50	0.529358	6.416571	16.15781	5.82778E-05	0.013779183	547	679	733	369	1300	685
Cep250	0.529241	4.483382	5.541766	0.018567833	0.529766703	198	248	363	142	467	345
Plekhm1	0.522327	4.67719	4.332695	0.037387005	0.704394754	83	110	113	43	233	106
Cdv3	0.519501	5.583848	6.25823	0.012361763	0.429066966	175	306	233	136	299	360
Fem1b	0.516904	6.293372	16.52762	4.79465E-05	0.012720219	801	1027	944	624	1566	962
Ppm1l	0.505502	7.504712	14.38567	0.000148931	0.026703391	2564	2840	2969	1654	4645	3309
Zbtb41	0.502707	4.540929	6.555101	0.01045839	0.406178199	244	230	238	115	461	279
Arid3a	0.502472	4.987872	6.548365	0.010498058	0.406861212	129	121	159	62	284	148
Ankrd27	0.499666	5.271027	8.091358	0.004447683	0.258638786	546	470	557	367	882	519
Cacnb1	0.498243	5.494458	6.839445	0.008916654	0.378444265	853	928	794	546	1465	922
Pgap1	0.492993	5.427387	5.632449	0.017630982	0.51579438	433	365	402	249	548	531
Sidt2	0.489695	6.073105	6.319368	0.011942617	0.429066966	433	448	554	305	835	484
Ttyh3	0.485176	5.524366	5.189647	0.022721836	0.579064072	398	684	777	403	1160	530
Arid5b	0.483206	6.443287	5.193056	0.022677312	0.579064072	258	497	427	191	501	612
Mal2	0.479958	9.481706	13.74928	0.000208901	0.034552777	747	1041	995	482	1649	1075
Zfp451	0.477854	4.446581	6.151083	0.013133162	0.440389035	215	251	293	164	421	258
Shc1	0.472262	5.380559	5.868157	0.015417184	0.482847354	430	518	642	304	1108	443
Tmem115	0.466758	4.707742	6.458	0.011045389	0.418402432	297	278	231	175	434	284
Map3k3	0.456532	5.695212	6.088479	0.013606614	0.448330643	315	435	359	259	569	358
Trpm3	0.455897	5.801185	6.131744	0.013277584	0.441546373	154	156	166	83	247	200
Ebf3	0.451548	5.696989	5.684973	0.017110807	0.510618748	812	1110	1195	553	1879	1094
Dhcr24	0.44876	8.933464	16.24191	5.57472E-05	0.013477045	2279	2272	2454	1439	4056	2270
Inpp5a	0.445516	5.800637	7.367059	0.00664295	0.321190376	426	489	387	237	845	408
Gprasp2	0.442502	6.016149	9.793294	0.001751494	0.150740568	1238	1210	1339	788	1869	1412
Rcn2	0.429542	4.581786	4.874996	0.027248503	0.620400836	221	245	241	133	414	235
Pnpla8	0.423161	5.366795	5.790627	0.016111843	0.493649021	401	514	547	267	715	584
Gna11	0.420551	7.181071	14.11893	0.000171609	0.029538632	1512	1840	1632	1032	2480	1760
Fam49b	0.415743	6.776803	10.89356	0.00096499	0.100789978	1190	1159	1202	726	2008	1108
Mdm4	0.411475	5.826873	9.200149	0.002419954	0.1771011	455	419	513	261	702	522
Bcl11b	0.405549	5.242268	4.960222	0.025936878	0.608079186	492	467	477	289	860	414
Gga2	0.405224	6.172321	6.720529	0.009530933	0.38801464	887	899	943	522	1562	877
Slc35a1	0.402776	6.195692	5.210815	0.022446816	0.578055343	183	273	208	143	340	211
Wdr82	0.402073	4.693388	4.664914	0.030784957	0.657286399	273	282	306	140	454	341
Klf7	0.390481	6.316103	7.709835	0.005492077	0.289825134	1210	1645	1543	720	2197	1770
Nup50	0.380866	6.447746	8.61032	0.003342636	0.216627007	643	773	694	417	930	805
Plekha3	0.373721	4.766637	4.108664	0.042664062	0.750131423	419	376	381	211	611	422
Nptx1	0.3708	6.418344	7.377981	0.006602724	0.319964458	1621	1669	2029	1015	2640	1841
Msn	0.343098	7.810328	4.884034	0.027106195	0.619784154	3876	4710	4630	2501	7350	3805
Tmem50b	0.340845	6.813193	8.382242	0.003789046	0.236040708	935	1032	1049	515	1444	1125
Slmo2	0.337721	5.572119	5.851835	0.015560814	0.485226759	399	501	503	235	753	466
Ipo9	0.334672	4.991104	4.695652	0.030239015	0.655209118	354	404	425	197	607	414
Zeb1	0.333922	6.30223	5.144402	0.023321445	0.58316509	1423	1320	1443	818	2156	1274
Kpnb1	0.333184	6.463935	7.783143	0.005273597	0.286532113	896	995	1049	514	1580	940
Eea1	0.333126	7.924761	8.421763	0.003707561	0.233935158	2954	2987	3526	1647	4514	3446
Ids	0.330327	7.492118	4.60514	0.031876252	0.666204354	1972	2204	3022	1357	3375	2418
Baz1b	0.329218	7.441917	7.164405	0.007436435	0.340386324	1138	1277	1370	653	1757	1404
Ank3	0.327225	8.44714	3.945527	0.046996136	0.785443766	5504	5977	8201	3161	11214	6255
Ppp2ca	0.321611	7.951161	5.808019	0.015953256	0.49237727	2159	2585	1930	1151	3259	2357
Bod1l	0.320937	7.560445	5.592484	0.018037703	0.522878739	1811	2421	2642	1131	3034	2682
Chd2	0.31914	6.865916	5.127431	0.02355056	0.58579636	735	948	1020	448	1458	867
Zdhhc20	0.316186	6.530315	4.778891	0.028810663	0.635720257	443	765	650	324	868	650
Erbb2ip	0.312482	6.741608	3.873627	0.049050418	0.79470542	340	347	353	150	667	295
Syne2	0.306547	7.703519	5.081759	0.024178834	0.592568192	443	549	568	226	916	488
Syt1	0.306144	7.822994	6.085255	0.013631467	0.448462765	4123	5235	5128	2372	7061	5123
Sun2	0.305206	6.49821	5.922558	0.014948238	0.476483392	595	652	766	378	884	697
Nedd4l	0.303662	7.018708	4.09589	0.042987599	0.751185748	1869	2392	2831	1168	3568	2387
Hook3	0.303036	7.741428	7.8146	0.005182581	0.285981922	1318	1514	1554	781	2146	1432
Rasa1	0.295957	7.559866	6.159298	0.013072307	0.440389035	2207	2075	1960	1158	3004	2002
Usp45	0.294072	5.597373	4.297183	0.038175557	0.709313724	374	451	519	226	628	470
Med1	0.291794	6.551671	4.949256	0.026101902	0.609781232	682	986	999	478	1368	788
Mycbp2	0.290177	8.554704	6.289141	0.012147993	0.429066966	2977	3831	4210	1785	5383	3780
Mtss1	0.286413	6.618115	5.717211	0.016799393	0.504122385	903	984	1169	555	1468	962
Tm9sf4	0.285409	6.35129	4.879116	0.027183534	0.620371191	703	693	827	358	1203	687
Tmx1	0.275901	6.923893	4.298718	0.038141114	0.709286269	1002	989	1196	619	1588	879
Clptm1l	0.274337	6.154851	4.20501	0.040304739	0.730176079	582	598	732	321	967	599
Syncrip	0.272792	6.660751	4.385062	0.036255181	0.699611189	693	775	833	407	1003	795
Fam126b	0.265742	7.239384	6.304948	0.012040142	0.429066966	1958	2269	2178	1071	3217	2002
Tmx4	0.251695	7.071111	4.990637	0.025484827	0.603252529	2286	2694	2717	1393	3674	2270

Celf1	0.251387	6.906991	3.96158	0.04654999	0.781569653	1006	1044	1386	634	1613	1009
Cd47	0.25076	6.562907	4.135509	0.041992432	0.746732391	1193	1446	1390	629	2008	1303
Rbm25	0.243703	8.72808	3.91128	0.047963062	0.789126837	2532	3194	3411	1516	4030	3092
Stmn2	0.241221	9.576853	3.885028	0.048718451	0.791637017	15051	18468	16536	8109	22620	17013
Mef2a	0.240004	6.951418	3.971388	0.046279613	0.779758933	1312	1604	1560	661	2241	1474
Lrrc58	0.228445	7.276887	3.854001	0.049627463	0.799812338	1692	1998	2280	1050	2742	1788
Evi5	0.22438	7.682579	4.032652	0.044627724	0.763283076	1961	2464	2461	1094	3229	2208

Table S10. miR-183C target genes in the cornea of SNS-CKO strain.

	log2FC	logCPM	LR	PValue	FDR	WT1	WT2	WT3	WT4	SNS-CKO1	SNS-CKO2	SNS-CKO3
Kcnh5	4.10648	1.675	7.513	0.0061	1	0	0	0	0	4	0	1
Stmnd1	3.51499	-0.7086	4.34	0.0372	1	0	0	0	0	2	1	0
Mgat4c	3.44474	2.0635	4.86	0.0275	1	0	0	0	0	1	2	0
Ikzf1	3.44472	1.4863	4.738	0.0295	1	0	0	0	0	3	0	0
B3galt1	3.3887	5.0622	4.987	0.0255	1	0	0	0	0	1	1	1
Slc7a14	3.38755	5.4721	5.005	0.0253	1	0	0	0	0	1	1	1
P2rx6	2.30325	4.0272	4.016	0.0451	1	0	1	0	0	1	2	2
Ebf1	1.75531	7.4255	13	0.0003	0.509334	4	5	2	0	10	8	11
Fat3	1.62907	4.8821	4.328	0.0375	1	2	0	0	2	6	2	2
9330151L19Rik	1.5256	1.828	4.826	0.028	1	2	6	0	1	5	5	10
Dusp18	1.48233	3.47	6.891	0.0087	1	6	1	3	1	9	3	13
Rab40b	1.31046	1.6305	4.196	0.0405	1	3	8	1	3	7	8	13
Klhl13	1.29623	4.0357	11.5	0.0007	0.632525	6	11	4	5	19	9	21
Hs3st1	1.24613	2.602	6.685	0.0097	1	4	11	3	2	10	14	11
St3gal6	1.21675	4.2565	5.914	0.015	1	4	2	0	7	4	2	18
Lancl3	1.20682	3.2078	4.436	0.0352	1	4	2	4	1	7	5	8
Tmem86a	0.95836	3.4718	4.621	0.0316	1	9	10	7	5	8	19	19
Fam149b	0.92849	2.8366	5.315	0.0211	1	15	19	17	22	24	16	78
Rimbp2	0.89603	4.6176	3.996	0.0456	1	7	2	2	8	17	3	7
Sh3pxd2b	0.8226	4.5583	8.475	0.0036	1	9	20	14	30	18	28	54
Cygb	0.77888	6.3264	5.863	0.0155	1	5	25	4	10	21	11	25
Reep1	0.75553	6.7262	5.984	0.0144	1	13	8	4	21	16	12	32
Tmem170b	0.73125	3.2092	5.547	0.0185	1	24	32	15	33	54	29	44
Ptprg	0.67125	5.3489	15.2	1E-04	0.302172	74	139	81	113	113	112	289
Yod1	0.60473	3.9501	4.362	0.0367	1	33	65	37	69	36	45	177
Sec14l1	0.5895	5.0192	8.531	0.0035	1	40	80	45	86	83	66	140
Gnai3	0.56281	5.5164	7.065	0.0079	1	184	330	149	243	227	231	602
Hes1	0.55294	7.7531	8.201	0.0042	1	1023	1560	703	1337	1326	1097	2916
Pdcd6	0.53291	5.8925	8.928	0.0028	1	176	314	159	275	231	225	606
Dgcr2	0.5158	5.4434	7.565	0.006	1	114	172	93	200	149	139	365
Gabarapl2	0.50713	6.0681	4.385	0.0363	1	69	111	60	119	84	50	295
Cx3cl1	0.50474	4.2338	5.456	0.0195	1	73	81	47	106	79	79	184
Rac1	0.49846	8.1171	9.922	0.0016	0.88998	761	1225	644	1106	1075	751	2414
Anxa3	0.49798	4.0464	4.19	0.0407	1	66	113	73	85	124	64	188
Kdelr2	0.4893	5.6744	3.962	0.0465	1	172	248	110	141	166	142	460
Sowahb	0.47809	4.2127	6.447	0.0111	1	91	127	81	133	134	107	225
Arf4	0.46774	6.1874	5.797	0.0161	1	235	351	204	363	292	240	760
Clptm1l	0.4624	5.8949	5.11	0.0238	1	199	251	150	206	204	136	594
Tsc1	0.4493	4.8939	4.558	0.0328	1	51	82	32	68	63	59	121
Mta1	0.4442	5.4058	5.59	0.0181	1	142	194	90	126	159	126	303
Elavl1	0.44121	5.1819	5.319	0.0211	1	140	203	89	163	160	145	320
Jade1	0.43617	5.3747	5.687	0.0171	1	130	164	121	189	159	149	340
Pcx	0.43427	4.7257	4.274	0.0387	1	75	109	50	83	66	77	197
Dazap2	0.42938	7.3884	8.501	0.0035	1	593	939	479	768	706	566	1743
Hs2st1	0.42564	4.4909	3.908	0.0481	1	41	54	34	41	42	62	68
Golph3l	0.4255	4.5232	3.915	0.0479	1	78	102	61	60	70	84	165
Tapt1	0.41756	4.876	3.937	0.0472	1	72	103	56	84	82	78	168
Map2k1	0.37726	5.7389	4.748	0.0293	1	180	246	151	222	213	211	376
Sgms2	0.3687	5.504	4.514	0.0336	1	153	236	133	205	180	151	417
Cd164	0.35143	7.7324	5.046	0.0247	1	936	1397	658	1241	1049	920	2260
Rapgef5	0.33118	6.1301	4.404	0.0358	1	246	279	172	225	230	226	457
Azin1	0.31842	6.5145	5.806	0.016	1	180	260	136	266	197	180	445
Map1lc3b	0.3089	8.2543	5.563	0.0183	1	358	520	314	582	539	383	750
Ctsb	0.28995	8.7305	5.075	0.0243	1	582	920	434	723	694	523	1321
Igsf3	0.28067	7.371	3.974	0.0462	1	482	875	443	637	568	525	1227
Sypl	0.275	7.4836	4.231	0.0397	1	822	1131	670	1133	883	764	1976
Ube3c	0.27283	6.0321	3.897	0.0484	1	114	247	111	201	182	146	286
Foxo1	0.27017	6.3978	4.083	0.0433	1	317	438	223	401	333	287	680
Prr13	0.26168	7.6459	4.845	0.0277	1	559	844	464	777	671	525	1288
Klf7	0.25981	8.7611	4.197	0.0405	1	207	308	162	290	241	233	406
Gmfb	0.25915	7.5787	4.201	0.0404	1	318	422	263	361	313	292	692
Paqr8	0.2523	6.3331	4.255	0.0391	1	308	413	264	330	325	308	587
Mbnl1	0.24722	7.8511	4.05	0.0442	1	413	691	357	605	456	408	1075

2310022B05Rik	0.20967	6.7629	3.855	0.0496	1	375	640	324	518	464	402	772

Table S11. miR-183C target genes in the cornea of MS-CKO strain.

	log2FC	logCPM	LR	PValue	FDR	WT1	WT2	WT3	WT4	WT5	MS-CKO1	MS-CKO2	MS-CKO3	MS-CKO4	MS-CKO5
H2-Bl	4.1254	-0.632	4.1755	0.041	0.835	0	0	0	0	0	9	0	0	1	0
Grid1	4.1057	3.0743	15.677	8E-05	0.055	0	0	0	0	0	5	0	4	0	2
Slc2a13	3.4947	1.6849	9.1534	0.0025	0.317	0	0	0	0	0	0	0	0	0	7
Kcnj3	3.2952	4.4333	8.762	0.0031	0.338	0	0	0	0	0	1	0	0	0	5
Astn1	3.2754	3.3011	8.5822	0.0034	0.353	0	0	0	0	0	0	6	0	0	0
Pirt	3.0632	8.0307	7.341	0.0067	0.464	0	0	0	0	0	0	0	0	2	3
Grik3	3.059	1.9567	7.1626	0.0074	0.483	0	0	0	0	0	0	0	5	0	0
Slc4a1	2.6742	0.2104	6.912	0.0086	0.505	0	2	0	0	0	0	9	3	3	0
Gm6878	2.6324	-0.128	3.9299	0.0474	0.868	0	0	0	0	0	0	3	0	0	1
Nanos1	2.5682	1.3964	6.0271	0.0141	0.604	0	0	0	1	0	0	0	8	0	1
Plcxd3	2.4443	4.3518	4.4024	0.0359	0.811	0	0	0	0	0	0	0	0	2	1
Elmo1	2.4368	3.9687	4.3923	0.0361	0.811	0	0	0	0	0	3	0	0	0	0
Slain1	2.4346	1.314	4.0742	0.0435	0.85	0	0	0	0	0	0	0	0	0	3
Tmem198	2.4325	2.9818	6.2986	0.0121	0.567	1	0	0	0	0	0	0	6	0	2
L3mbtl1	1.9249	1.029	7.7597	0.0053	0.427	2	2	3	0	0	3	9	9	3	2
Trim46	1.6053	3.0118	5.5025	0.019	0.665	4	0	1	0	0	1	0	2	4	8
Apoo	1.5443	1.5823	6.7117	0.0096	0.525	3	1	0	6	2	8	24	1	2	3
D630045J12Rik	1.3068	3.4204	7.196	0.0073	0.48	6	0	3	1	3	3	15	8	2	4
Lancl3	1.2291	3.2078	6.5498	0.0105	0.545	1	3	8	1	1	7	12	8	3	1
Fgf14	1.2241	2.7455	7.2074	0.0073	0.478	6	4	13	13	7	5	42	33	9	13
Filip1	1.1504	4.8424	4.6581	0.0309	0.773	5	3	1	1	0	0	21	0	1	0
Rab6b	1.0795	8.8355	8.9566	0.0028	0.324	7	7	5	1	5	16	0	18	4	11
Gpr155	1.078	4.1895	8.0819	0.0045	0.394	8	4	8	3	1	9	15	14	1	9
Trib3	1.0022	1.7691	7.9628	0.0048	0.402	15	12	11	13	10	14	43	20	19	20
Kcnc3	0.9888	6.7981	9.4519	0.0021	0.287	16	5	8	2	2	3	35	10	8	6
Ahr	0.9038	3.0744	7.6081	0.0058	0.437	46	26	35	19	19	56	27	51	33	78
Arhgap31	0.8911	4.4979	5.3001	0.0213	0.696	25	5	8	2	13	12	25	25	15	20
Ttc21b	0.87	2.5131	5.42	0.0199	0.677	11	10	16	5	3	8	16	10	20	16
Gprasp2	0.8026	5.8317	8.1322	0.0043	0.391	9	10	15	9	10	21	33	19	9	5
Nrbf2	0.7557	2.884	4.3046	0.038	0.813	18	23	17	23	4	19	23	18	24	38
Sh3pxd2b	0.7193	4.5583	11.342	0.0008	0.18	34	48	21	31	9	49	64	48	19	37
Gab2	0.6819	4.5415	4.5445	0.033	0.785	13	22	7	13	8	7	26	14	28	13
Lmnb2	0.6644	3.7546	11.542	0.0007	0.171	69	97	88	59	40	96	126	114	43	140
Ostm1	0.6482	4.2969	9.2944	0.0023	0.307	42	66	59	63	22	41	119	64	39	103
Gnaz	0.6384	6.2114	4.8519	0.0276	0.747	18	15	5	12	1	5	42	8	8	12
Kctd7	0.6102	3.5088	6.6527	0.0099	0.536	33	16	32	18	13	24	52	40	21	23
Col5a1	0.6023	4.4851	4.1656	0.0413	0.838	54	123	244	102	32	88	266	261	47	108

Cdyl2	0.5743	3.7812	4.7695	0.029	0.756	15	23	21	16	7	20	31	37	5	21
Bnip3	0.5223	5.8122	5.4152	0.02	0.677	33	40	21	21	15	61	32	22	23	30
Zfp592	0.5103	4.3184	7.5343	0.0061	0.446	54	90	116	94	40	93	159	107	43	122
Tmem170b	0.4956	3.2092	3.883	0.0488	0.869	26	45	52	44	9	36	66	55	26	39
Mras	0.4777	6.8429	5.7123	0.0168	0.634	40	100	137	37	32	116	112	114	25	67
Slc7a8	0.4428	4.4525	4.3835	0.0363	0.811	97	169	141	167	77	118	192	182	116	187
Fam73b	0.4392	4.5216	4.3401	0.0372	0.813	32	64	45	61	27	43	86	31	48	72
Zfp532	0.4318	4.8884	6.2565	0.0124	0.575	111	159	173	120	50	109	195	206	86	146
Tpm2	0.4215	6.6972	5.4141	0.02	0.677	575	1137	940	740	374	834	848	714	793	953
Ptch1	0.4126	4.7563	4.2684	0.0388	0.82	54	77	67	54	34	41	77	108	55	60
Mapk8ip1	0.3975	7.1831	4.0062	0.0453	0.858	22	30	35	33	9	30	24	45	27	27
Nampt	0.3757	5.1803	8.8338	0.003	0.332	150	201	194	196	73	199	293	190	95	199
Slc39a1	0.3712	5.113	4.9413	0.0262	0.736	193	252	223	192	139	205	375	186	164	267
Dcaf12	0.3402	6.2115	11.82	0.0006	0.157	406	497	491	345	175	453	621	483	211	455
Xbp1	0.3351	5.8269	5.085	0.0241	0.718	287	349	422	251	173	240	467	333	190	493
Tox4	0.3182	5.0803	4.2385	0.0395	0.823	139	229	196	126	56	162	211	175	82	200
Dazap2	0.273	7.3884	5.0252	0.025	0.722	1031	1328	1300	914	548	897	1404	1234	557	1607
Rap2c	0.2644	5.3315	5.0238	0.025	0.722	129	159	179	138	92	159	246	157	70	157
Tmsb4x	0.2642	12.325	4.6248	0.0315	0.774	36652	43220	44653	36924	22121	35277	49614	33989	24003	59386
Mdp1	0.2536	5.9027	5.0504	0.0246	0.721	312	405	358	310	164	348	557	400	154	276
Pcmt1	0.2325	6.9294	4.6564	0.0309	0.773	190	287	283	177	125	219	299	241	114	273

Table S12. miR-183C target genes in the cornea of miR-183C KO train.

	log2FC	logCPM	LR	PValue	FDR	WT1	WT2	WT3	KO1	KO2	KO3
Mak	5.995725	-0.768916	6.099519	0.013521871	0.376390618	0	0	0	6	0	7
Myrip	5.382911	1.506843	15.27673	9.28533E-05	0.015564362	0	0	0	0	11	2
Fbxl2	5.269457	3.691976	18.7582	1.48384E-05	0.004041313	0	0	0	1	10	0
Tagln3	5.081802	6.733713	16.91874	3.90145E-05	0.008229759	0	0	0	0	0	9
Ubxn10	5.037601	-1.108022	4.09973	0.042890058	0.684586422	0	0	0	0	13	0
Nrn1	4.882861	7.773578	14.87916	0.000114622	0.018133953	0	0	0	2	6	0
Fibcd1	4.741054	-0.151272	7.37113	0.006627926	0.254200464	0	0	0	6	0	0
Myt1l	4.504685	6.609825	11.28124	0.000782943	0.068758403	0	0	0	6	0	0
Elavl4	4.501532	8.809614	11.29743	0.000776144	0.06844061	0	0	0	6	0	0
Cacna1e	4.447176	0.660783	7.43869	0.006383633	0.250156494	0	0	0	5	0	0
Dgkk	4.398428	2.509731	9.978912	0.001583432	0.105304624	0	0	0	0	6	0
Tub	4.351791	-0.281715	6.31507	0.011971602	0.348553444	0	0	0	0	2	3
Prss12	3.910236	4.114798	7.291381	0.006928619	0.25933058	0	0	0	0	4	0
Hoxd8	3.790268	1.441202	10.36403	0.001284947	0.090943797	1	0	0	1	14	0
Kcnh5	3.740489	0.867971	5.526014	0.018735736	0.454473608	0	0	0	0	4	0
Gpr22	3.732446	2.81456	13.90779	0.000192001	0.025927263	1	0	0	0	12	1
Unc5d	3.648659	1.117715	5.354173	0.020672732	0.481091376	0	0	0	3	0	0
Rundc3b	3.107325	3.224422	8.606602	0.003349467	0.172079943	1	0	0	0	8	0
Fut9	2.884179	1.445702	19.60133	9.54028E-06	0.002932408	14	4	7	11	78	34
Avil	2.606653	7.959145	8.521967	0.00350885	0.1763935	2	0	0	0	0	9
Sox2	2.538395	4.445089	7.857341	0.005061477	0.220450876	0	0	2	0	9	0
Scn2a1	2.483773	3.06872	8.643283	0.003282693	0.169784668	0	1	2	4	0	7
Nsg1	2.45456	4.048026	13.558	0.000231304	0.029103686	0	5	0	4	16	0
Clcn5	2.40528	3.111664	9.667104	0.001875968	0.119761824	5	0	0	4	0	11
Nova2	2.33268	2.975271	7.701866	0.005516377	0.230020078	0	0	3	3	7	1
Elmo1	2.215123	3.755488	16.44463	5.00917E-05	0.009729398	4	2	4	5	14	11
Celsr3	2.086823	3.733945	4.189912	0.040665209	0.666376723	2	0	0	6	0	0
Coro2b	2.047226	6.100321	23.07888	1.55489E-06	0.00079655	7	6	2	4	27	10
Pcdh17	1.940878	5.932583	18.00892	2.19872E-05	0.005378547	1	13	0	12	15	8
Grip1	1.923959	4.430855	35.59714	2.42643E-09	4.45078E-06	57	37	18	44	128	89
Cdsn	1.865646	3.314134	10.9085	0.000957242	0.075754905	167	72	86	67	331	294
Trim46	1.842892	3.554254	10.67175	0.001087841	0.082126241	0	7	5	2	23	6
D630045J12Rik	1.841322	3.437815	18.41235	1.77901E-05	0.00461171	10	15	9	16	28	30
Esrrg	1.77696	3.391285	8.107857	0.004407381	0.201764262	0	9	0	5	15	2
Slc39a14	1.771217	1.67146	6.199553	0.012778261	0.361759281	1	6	7	15	5	8
Sv2c	1.741765	5.891348	5.602654	0.017933287	0.446594836	0	8	0	4	11	4
Plcxd3	1.702915	4.770777	7.295622	0.006912285	0.25933058	0	7	0	0	16	0
9330159F19Rik	1.65587	5.489106	7.677997	0.005589818	0.231735126	0	0	8	2	13	2
Robo2	1.610326	5.490906	16.88318	3.97524E-05	0.008304003	22	8	4	3	41	23
Gria3	1.602819	3.88974	15.24158	9.45974E-05	0.015656601	15	8	7	16	23	17
Lphn3	1.530869	2.42231	6.455275	0.01106234	0.333966347	20	4	8	6	57	6
Prkd1	1.516851	2.537231	5.959152	0.014641081	0.396468879	9	0	8	2	23	8
Tox3	1.46694	4.208456	8.384764	0.003783794	0.184231678	9	6	0	7	15	5
Bnip3	1.404131	5.968689	25.51819	4.38231E-07	0.000286867	103	78	104	53	309	143
Sox5	1.402133	2.565192	6.970578	0.008286071	0.28711827	10	13	7	8	47	5
Sncb	1.300131	7.117983	4.261948	0.0389752	0.653107798	0	8	0	7	6	0
Rnf157	1.220213	6.404902	8.321772	0.00391727	0.18940219	9	6	7	8	24	2
Lcp1	1.191605	5.703614	7.599571	0.00583822	0.238358892	71	22	38	10	81	82
Mmd	1.161359	2.957801	5.854945	0.015533342	0.412611601	16	15	56	29	42	44
Cntn1	1.156595	8.946245	30.42967	3.46192E-08	4.13814E-05	66	35	64	42	158	46
Chst1	1.014639	4.234149	5.100103	0.023924428	0.516306912	4	20	7	8	23	11
Mef2c	0.993958	6.128493	17.65589	2.64694E-05	0.006399049	33	39	60	49	69	46
Wisp1	0.985652	3.239455	4.379971	0.036363621	0.631986814	63	90	67	21	197	88
Rnf24	0.980306	3.758715	7.070479	0.007836428	0.279616235	32	13	21	20	32	26
Aatk	0.867533	8.915216	16.0801	6.07188E-05	0.011166034	72	58	63	50	98	70
Pdzd4	0.866418	4.354298	5.372774	0.020453421	0.479255481	30	25	13	34	45	3
Gng5	0.865576	3.24461	5.665791	0.017298916	0.438868758	186	70	156	132	253	91
Ier5l	0.784008	2.956932	4.040863	0.044411113	0.693935742	72	30	79	68	84	38
Fyn	0.76893	6.334412	6.360166	0.011671064	0.344294107	23	27	11	20	21	23
Anxa3	0.75456	4.035785	7.287791	0.006942481	0.25933058	211	159	216	112	336	184
B4galnt1	0.751776	5.016262	11.637	0.000646527	0.060219387	198	81	116	85	245	97
Bnc2	0.730588	3.890631	4.551911	0.032882025	0.603145484	22	39	15	22	38	21
Dnajb9	0.728872	3.107109	4.209925	0.040188104	0.662225126	57	55	49	41	112	30
Slc16a13	0.660032	3.187751	3.884645	0.048729559	0.719255682	199	123	144	92	335	85
Ankrd27	0.619722	5.271027	11.07438	0.000875288	0.073565219	285	210	180	163	376	138
Cacna2d1	0.617079	7.040251	7.499491	0.006171644	0.246570902	58	55	34	57	66	20

Zeb2	0.609941	6.816022	8.463185	0.003624066	0.179666854	119	129	89	82	136	99
Slc35d1	0.575186	3.401966	3.888424	0.048620023	0.719107082	129	108	155	90	181	98
Dusp1	0.548482	4.995334	6.796925	0.0091315	0.302732425	376	497	412	289	610	318
Slc35e4	0.547299	4.212664	4.514072	0.033617119	0.60795906	128	98	106	70	119	101
Dip2a	0.526233	4.371574	5.288841	0.021462503	0.489181367	142	107	147	105	198	68
Rnf208	0.508854	7.110025	8.439918	0.003670728	0.180513584	1036	566	642	377	1087	552
Dtna	0.492458	6.087402	4.124039	0.042278032	0.679353346	54	82	87	36	111	59
Sidt2	0.48589	6.073105	6.4088	0.011355612	0.338872879	1057	684	878	419	1139	715
Magi1	0.485358	6.487522	7.343513	0.006730519	0.25630768	119	113	154	76	204	77
Parm1	0.484455	8.280592	6.753734	0.00935517	0.305441321	1047	1077	904	499	1433	798
Tns1	0.480867	7.16856	12.03257	0.00052279	0.051400206	236	191	240	144	303	147
Tpm2	0.46765	7.326003	5.50409	0.018972049	0.458654616	2770	3001	3187	1755	5056	1708
Prr13	0.466843	8.074541	7.780133	0.00528239	0.223292522	3723	2377	2876	1413	4713	2015
Slc39a5	0.465075	5.290979	6.653096	0.009898366	0.315049168	197	146	194	97	261	121
Slc25a1	0.456443	7.409823	8.300272	0.003963915	0.190374082	1313	1097	1249	625	1623	932
Tgfbi	0.437545	8.837142	7.695231	0.005536691	0.230420604	10348	8009	8573	5912	11476	5650
Rab34	0.43091	4.728428	3.96257	0.046522637	0.704916231	313	243	243	165	314	188
Pde4d	0.404579	5.255696	4.194906	0.040545594	0.665938162	195	114	128	96	166	94
Msn	0.388616	7.810328	5.19707	0.022624988	0.502142899	324	322	276	165	366	228
Prkce	0.380874	8.399661	5.588775	0.018075929	0.447036419	453	258	249	174	368	224
Slc39a1	0.375948	5.571921	5.45384	0.019525358	0.466713643	952	516	726	454	951	402
Sobp	0.375638	5.579599	4.02103	0.044936273	0.699601188	148	137	140	90	217	62
Prkca	0.338533	7.179291	4.029655	0.044707072	0.698053233	353	298	337	210	492	139
Gyk	0.334953	5.751856	3.888755	0.048610432	0.719107082	1146	674	938	699	1026	437
Zfc3h1	0.334712	5.85405	4.730167	0.029638004	0.573463395	945	632	675	575	844	355
Dab2ip	0.312138	6.578239	5.046749	0.024672183	0.525070906	1053	837	843	555	950	581
Vps36	0.297004	5.63755	4.926747	0.026444046	0.541054773	924	614	729	490	879	389
Ssh2	0.292401	6.356147	4.431376	0.035284043	0.625347173	1198	670	841	612	942	477
Atp6v1c1	0.285636	6.457112	4.652201	0.031013757	0.583807525	554	417	471	263	584	276
Rtn4	0.239339	9.160383	3.930177	0.047426944	0.711106713	1705	1011	1337	715	1636	707

Table S13. GO_BP analysis of miR-183C targets in SNS-CKO TG up genes

Category	Term			Count	%	PValue	Genes	List Total	Pop Hits	Pop Total	ld Enrichm	Bonferroni	Benjamini	FDR
GOTERM_BP_[	GO	7507	heart development	5	11.90476	0.002284	MAP2K3, PPP3R1, ITGA3, TGFBR1, KDM6A	38	306	20240	8.70313	0.715635	0.728945	0.728945
**GOTERM_BP_[**	**GO**	**1904646**	**cellular response to beta-amyloid**	**3**	**7.142857**	**0.002801**	**CACNB1, FYN, FOXO3**	**38**	**43**	**20240**	**37.16034**	**0.786186**	**0.728945**	**0.728945**
GOTERM_BP_[	GO	10628	positive regulation of gene expression	6	14.28571	0.004869	GRIA1, RARG, ITGA3, LDLR, TGFBR1, KDM6A	38	612	20240	5.221878	0.931727	0.728945	0.728945
GOTERM_BP_[	GO	46777	protein autophosphorylation	4	9.52381	0.005301	ALK, MAP3K3, FYN, TGFBR1	38	194	20240	10.98209	0.946257	0.728945	0.728945
GOTERM_BP_[	GO	10730	negative regulation of hydrogen peroxide biosynthetic process	2	4.761905	0.009108	MPV17L, FYN	38	5	20240	213.0526	0.993476	0.8406	0.8406
**GOTERM_BP_[**	**GO**	**71560**	**cellular response to transforming growth factor beta stimulus**	**3**	**7.142857**	**0.00917**	**FYN, WNT2, TGFBR1**	**38**	**79**	**20240**	**20.22652**	**0.993698**	**0.8406**	**0.8406**
**GOTERM_BP_[**	**GO**	**1843**	**neural tube closure**	**3**	**7.142857**	**0.014934**	**RARG, GRHL2, KDM6A**	**38**	**102**	**20240**	**15.66563**	**0.999745**	**0.983687**	**0.983687**
**GOTERM_BP_[**	**GO**	**60070**	**canonical Wnt signaling pathway**	**3**	**7.142857**	**0.017817**	**FOXO3, WNT2, KDM6A**	**38**	**112**	**20240**	**14.26692**	**0.999949**	**0.983687**	**0.983687**
GOTERM_BP_[	GO	34383	low-density lipoprotein particle clearance	2	4.761905	0.019931	EHD1, LDLR	38	11	20240	96.84211	0.999984	0.983687	0.983687
GOTERM_BP_[	GO	7169	transmembrane receptor protein tyrosine kinase signaling pathway	3	7.142857	0.020597	ALK, FYN, SAMD12	38	121	20240	13.20574	0.999989	0.983687	0.983687
GOTERM_BP_[	GO	38066	p38MAPK cascade	2	4.761905	0.021723	MAP2K3, ZFP36	38	12	20240	88.77193	0.999994	0.983687	0.983687
GOTERM_BP_[	GO	48333	mesodermal cell differentiation	2	4.761905	0.021723	ITGA3, KDM6A	38	12	20240	88.77193	0.999994	0.983687	0.983687
GOTERM_BP_[	GO	60317	cardiac epithelial to mesenchymal transition	2	4.761905	0.025299	WNT2, TGFBR1	38	14	20240	76.09023	0.999999	0.983687	0.983687
GOTERM_BP_[	GO	16310	phosphorylation	5	11.90476	0.026707	ALK, MAP2K3, MAP3K3, FYN, TGFBR1	38	626	20240	4.254246	1	0.983687	0.983687
GOTERM_BP_[	GO	60487	lung epithelial cell differentiation	2	4.761905	0.028862	PPP3R1, GRHL2	38	16	20240	66.57895	1	0.983687	0.983687
GOTERM_BP_[	GO	35264	multicellular organism growth	3	7.142857	0.029929	RARG, GRHL2, KDM6A	38	148	20240	10.79659	1	0.983687	0.983687
GOTERM_BP_[	GO	6468	protein phosphorylation	5	11.90476	0.030405	ALK, MAP2K3, MAP3K3, FYN, TGFBR1	38	652	20240	4.084598	1	0.983687	0.983687
GOTERM_BP_[	GO	43065	positive regulation of apoptotic process	4	9.52381	0.036725	RARG, CASP2, FOXO3, TGFBR1	38	402	20240	5.299817	1	1	1
GOTERM_BP_[	GO	60324	face development	2	4.761905	0.039475	RARG, GRHL2	38	22	20240	48.42105	1	1	1
GOTERM_BP_[	GO	70542	response to fatty acid	2	4.761905	0.042987	INSIG2, FOXO3	38	24	20240	44.38596	1	1	1
GOTERM_BP_[	GO	32331	negative regulation of chondrocyte differentiation	2	4.761905	0.042987	RARG, TGFBR1	38	24	20240	44.38596	1	1	1
GOTERM_BP_[	GO	45893	positive regulation of transcription, DNA-templated	5	11.90476	0.044363	MAP2K3, ZKSCAN17, FOXO3, GRHL2, TGFBR1	38	736	20240	3.618421	1	1	1
**GOTERM_BP_[**	**GO**	**10976**	**positive regulation of neuron projection development**	**3**	**7.142857**	**0.044906**	**EHD1, ITGA3, FYN**	**38**	**185**	**20240**	**8.637269**	**1**	**1**	**1**
**GOTERM_BP_[**	**GO**	**7399**	**nervous system development**	**4**	**9.52381**	**0.047277**	**ALK, GAP43, NOVA2, TGFBR1**	**38**	**445**	**20240**	**4.7877**	**1**	**1**	**1**
GOTERM_BP_[	GO	7275	multicellular organism development	6	14.28571	0.048098	ALK, GAP43, ZFP36, SPRY4, FYN, WNT2	38	1098	20240	2.910555	1	1	1

Table S14. KEGG pathway analysis of miR-183C targets in the TG of SNS-CKO strain.

Category	Term		Count	%	PValue	Genes	List Total	Pop Hits	Pop Total	ld Enrichm	Bonferroni	Benjamini	FDR
**KEGG_PATHWAY**	**mmu04010**	**MAPK signaling pathway**	**6**	**14.28571**	**3.79E-04**	**CACNB1, MAP2K3, MAP3K3, PPP3R1, GNG12, TGFBR1**	**21**	**302**	**9212**	**8.715232**	**0.04514**	**0.046182**	**0.045804**
KEGG_PATHWAY	mmu05235	PD-L1 expression and PD-1 checkpoint pathway in cancer	4	9.52381	8.54E-04	ALK, MAP2K3, MAP3K3, PPP3R1	21	88	9212	19.93939	0.098962	0.052082	0.051655
KEGG_PATHWAY	mmu04218	Cellular senescence	4	9.52381	0.006958	MAP2K3, PPP3R1, FOXO3, TGFBR1	21	184	9212	9.536232	0.573388	0.282971	0.280651
KEGG_PATHWAY	mmu05166	Human T-cell leukemia virus 1 infection	4	9.52381	0.016001	MAP3K3, PPP3R1, ZFP36, TGFBR1	21	250	9212	7.018667	0.860246	0.414795	0.411395
KEGG_PATHWAY	mmu04144	Endocytosis	4	9.52381	0.02001	EHD1, VPS36, LDLR, TGFBR1	21	272	9212	6.45098	0.915078	0.414795	0.411395
**KEGG_PATHWAY**	**mmu04725**	**Cholinergic synapse**	**3**	**7.142857**	**0.024134**	**KCNJ14, FYN, GNG12**	**21**	**112**	**9212**	**11.75**	**0.949234**	**0.414795**	**0.411395**
**KEGG_PATHWAY**	**mmu04724**	**Glutamatergic synapse**	**3**	**7.142857**	**0.024944**	**GRIA1, PPP3R1, GNG12**	**21**	**114**	**9212**	**11.54386**	**0.954119**	**0.414795**	**0.411395**
KEGG_PATHWAY	mmu05200	Pathways in cancer	5	11.90476	0.0272	ALK, ITGA3, GNG12, WNT2, TGFBR1	21	543	9212	4.039288	0.965415	0.414795	0.411395
KEGG_PATHWAY	mmu04380	Osteoclast differentiation	3	7.142857	0.030913	PPP3R1, FYN, TGFBR1	21	128	9212	10.28125	0.978311	0.419045	0.415611
KEGG_PATHWAY	mmu04921	Oxytocin signaling pathway	3	7.142857	0.042823	CACNB1, PPP3R1, KCNJ14	21	153	9212	8.601307	0.995202	0.522446	0.518164

Table S15. Biological processes GO analysis of miR-183C targets in the cornea of SNS-CKO strain.

Category	Term		Count	%	PValue	Genes	List Total	Pop Hits	Pop Total	Fold Enrichment	Bonferroni	Benjamini	FDR
GOTERM_	GO:0016236	macroautophagy	3	4.6875	0.006833	GABARAPL2, MAP1LC3B, YOD1	58	44	20240	23.7931	0.988942	1	1
**GOTERM_**	**GO:0006890**	**retrograde vesicle-mediated transport, Golgi to ER**	**3**	**4.6875**	**0.006833**	**ARF4, GOLPH3L, KDELR2**	**58**	**44**	**20240**	**23.7931**	**0.988942**	**1**	**1**
GOTERM_	GO:0048538	thymus development	3	4.6875	0.011262	MAP2K1, HES1, IKZF1	58	57	20240	18.36661	0.999413	1	1
GOTERM_	GO:0051496	positive regulation of stress fiber assembly	3	4.6875	0.011643	SH3PXD2B, TSC1, RAC1	58	58	20240	18.04994	0.999545	1	1
GOTERM_	GO:0010595	positive regulation of endothelial cell migration	3	4.6875	0.013633	ANXA3, PDCD6, RAC1	58	63	20240	16.61741	0.999879	1	1
GOTERM_	GO:0035437	maintenance of protein localization in endoplasmic reticulum	2	3.125	0.014003	KDELR2, TAPT1	58	5	20240	139.5862	0.999905	1	1
GOTERM_	GO:0045893	positive regulation of transcription, DNA-templated	7	10.9375	0.017049	KLF7, MAP2K1, MTA1, EBF1, HES1, IKZF1, FOXO1	58	736	20240	3.318966	0.999988	1	1
**GOTERM_**	**GO:0010977**	**negative regulation of neuron projection development**	**3**	**4.6875**	**0.026095**	**TSC1, HES1, PTPRG**	**58**	**89**	**20240**	**11.76288**	**1**	**1**	**1**
GOTERM_	GO:0006995	cellular response to nitrogen starvation	2	3.125	0.027814	GABARAPL2, MAP1LC3B	58	10	20240	69.7931	1	1	1
GOTERM_	GO:0007155	cell adhesion	6	9.375	0.028481	CD164, HES1, FAT3, RAC1, CX3CL1, DGCR2	58	611	20240	3.42683	1	1	1
GOTERM_	GO:0010764	negative regulation of fibroblast migration	2	3.125	0.030553	RAC1, CYGB	58	11	20240	63.44828	1	1	1
**GOTERM_**	**GO:1904936**	**interneuron migration**	**2**	**3.125**	**0.033285**	**FAT3, RAC1**	**58**	**12**	**20240**	**58.16092**	**1**	**1**	**1**
GOTERM_	GO:0046425	regulation of JAK-STAT cascade	2	3.125	0.036009	HES1, RAC1	58	13	20240	53.687	1	1	1
GOTERM_	GO:0035881	amacrine cell differentiation	2	3.125	0.036009	HES1, IKZF1	58	13	20240	53.687	1	1	1
GOTERM_	GO:0051492	regulation of stress fiber assembly	2	3.125	0.044136	TSC1, RAC1	58	16	20240	43.62069	1	1	1

Table S16. KEGG pathway analysis of miR-183C targets in the cornea of SNS-CKO strain.

Category	Term		Count	%	PValue	Genes	List Total	Pop Hits	Pop Total	Fold Enrichment	Bonferroni	Benjamini	FDR
**KEGG_PATHWAY**	**mmu04140**	**Autophagy - animal**	**5**	**7.8125**	**6.21E-04**	**GABARAPL2, MAP1LC3B, MAP2K1, TSC1, CTSB**	**27**	**142**	**9212**	**12.01356**	**0.094119**	**0.098816**	**0.098816**
**KEGG_PATHWAY**	**mmu04071**	**Sphingolipid signaling pathway**	**4**	**6.25**	**0.005162**	**MAP2K1, GNAI3, RAC1, SGMS2**	**27**	**126**	**9212**	**10.83128**	**0.560869**	**0.285085**	**0.285085**
KEGG_PATHWAY	mmu05163	Human cytomegalovirus infection	5	7.8125	0.005379	MAP2K1, GNAI3, TSC1, RAC1, CX3CL1	27	256	9212	6.663773	0.575806	0.285085	0.285085
**KEGG_PATHWAY**	**mmu04062**	**Chemokine signaling pathway**	**4**	**6.25**	**0.016289**	**MAP2K1, GNAI3, RAC1, CX3CL1**	**27**	**192**	**9212**	**7.108025**	**0.926554**	**0.647471**	**0.647471**
KEGG_PATHWAY	mmu04015	Rap1 signaling pathway	4	6.25	0.021686	MAP2K1, GNAI3, RAC1, RAPGEF5	27	214	9212	6.377293	0.969378	0.689617	0.689617
KEGG_PATHWAY	mmu05231	Choline metabolism in cancer	3	4.6875	0.030847	MAP2K1, TSC1, RAC1	27	98	9212	10.44444	0.993139	0.803113	0.803113
KEGG_PATHWAY	mmu04152	AMPK signaling pathway	3	4.6875	0.049413	TSC1, ELAVL1, FOXO1	27	127	9212	8.059493	0.999683	0.803113	0.803113
**KEGG_PATHWAY**	**mmu04726**	**Serotonergic synapse**	**3**	**4.6875**	**0.052229**	**MAP2K1, DUSP1, GNAI3**	**27**	**131**	**9212**	**7.813401**	**0.999802**	**0.803113**	**0.803113**
KEGG_PATHWAY	mmu04068	FoxO signaling pathway	3	4.6875	0.052229	GABARAPL2, MAP2K1, FOXO1	27	131	9212	7.813401	0.999802	0.803113	0.803113

Table S17. Biological processes GO analysis of miR-183C targets in the TG of miR-183C KO strain.

Category	Term		Count	%	PValue	Genes	List Total	Pop Hits	Pop Total	Fold Enrichı	Bonferroni	Benjamini	FDR
GOTERM_BP_I	GO:0045944	positive regulation of transcription from RNA polymerase II promoter	20	15.15152	2.30E-04	MEF2A, MED1, CEBPA, BCL11B, PRRX1, PAX6, ARID3A, EBF3, NPNT, BA	125	1257	20240	2.5762928	0.261159546	0.2905658	0.290566
GOTERM_BP_I	GO:0051496	positive regulation of stress fiber assembly	5	3.787879	4.41E-04	NOX4, BRAF, CD47, PPM1F, WNT4	125	58	20240	13.958621	0.440806468	0.2905658	0.290566
GOTERM_BP_I	GO:0000381	regulation of alternative mRNA splicing, via spliceosome	5	3.787879	7.63E-04	RBM25, CELF1, NOVA1, NOVA2, CELF6	125	67	20240	12.083582	0.634568288	0.3354305	0.335431
GOTERM_BP_I	GO:0035556	intracellular signal transduction	10	7.575758	0.002151	MAP3K3, SHC1, PNPLA8, PRKCE, PLEKHM1, PSEN2, BRAF, RAPGEF5, DC	125	461	20240	3.5123644	0.941474291	0.7088088	0.708809
GOTERM_BP_I	GO:0010811	positive regulation of cell-substrate adhesion	4	3.030303	0.002811	ITGA3, PRKCE, NPNT, PPM1F	125	46	20240	14.08	0.97553943	0.7102606	0.710261
GOTERM_BP_I	GO:0033077	T cell differentiation in thymus	4	3.030303	0.003367	BCL11B, NKAP, BRAF, WNT4	125	49	20240	13.217959	0.988266494	0.7102606	0.710261
GOTERM_BP_I	GO:0045184	establishment of protein localization	4	3.030303	0.003772	SLC1A1, ANK3, NPNT, KPNB1	125	51	20240	12.699608	0.993134354	0.7102606	0.710261
GOTERM_BP_I	GO:0010628	positive regulation of gene expression	11	8.333333	0.004434	MEF2A, MED1, CEBPA, ITGA3, CELF1, MSN, BRAF, PAX6, ANK3, LDLR, F	125	612	20240	2.9103268	0.997139317	0.7304627	0.730463
GOTERM_BP_I	GO:0006606	protein import into nucleus	5	3.787879	0.005843	MED1, PPP3R1, IPO9, NUP50, KPNB1	125	117	20240	6.9196581	0.999557544	0.8556206	0.855621
GOTERM_BP_I	GO:0043066	negative regulation of apoptotic process	11	8.333333	0.008294	GREM1, MED1, BCL11B, SHC1, PRKCE, RASA1, PSEN2, MDM4, DHCR24	125	671	20240	2.6544262	0.999982915	1	1
GOTERM_BP_I	GO:0071539	protein localization to centrosome	3	2.272727	0.009409	CCDC14, CEP250, HOOK3	125	24	20240	20.24	0.999996121	1	1
GOTERM_BP_I	GO:0000122	negative regulation of transcription from RNA polymerase II promoter	14	10.60606	0.01061	MEF2A, MED1, IRX2, CEBPA, PRRX1, NKAP, PSEN2, ARID5B, PAX6, ZBTE	125	1031	20240	2.1987197	0.999999216	1	1
GOTERM_BP_I	GO:0031022	nuclear migration along microfilament	2	1.515152	0.012216	SUN2, SYNE2	125	2	20240	161.92	0.999999908	1	1
GOTERM_BP_I	GO:0006470	protein dephosphorylation	5	3.787879	0.013696	PPP2CA, PPP3R1, PPM1L, PTPN9, PPM1F	125	150	20240	5.3973333	0.999999987	1	1
GOTERM_BP_I	GO:0045893	positive regulation of transcription, DNA-templated	11	8.333333	0.015115	GREM1, MEF2A, MED1, KLF7, CEBPA, SHC1, MORF4L2, EBF3, PAX6, GF	125	736	20240	2.42	0.999999998	1	1
GOTERM_BP_I	GO:0050680	negative regulation of epithelial cell proliferation	4	3.030303	0.017916	ZEB1, CELF1, PAX6, MTSS1	125	90	20240	7.1964444	1	1	1
GOTERM_BP_I	GO:0015031	protein transport	10	7.575758	0.017967	RD3, GGA2, IPO9, PGAP1, ANKRD27, PLEKHM1, PSEN2, HOOK3, SNX30	125	646	20240	2.5065015	1	1	1
GOTERM_BP_I	GO:0043368	positive T cell selection	2	1.515152	0.018268	BCL11B, BRAF	125	3	20240	107.94667	1	1	1
GOTERM_BP_I	GO:0021817	nucleokinesis involved in cell motility in cerebral cortex radial glia guided migration	2	1.515152	0.018268	SUN2, SYNE2	125	3	20240	107.94667	1	1	1
GOTERM_BP_I	GO:0072660	maintenance of protein location in plasma membrane	2	1.515152	0.018268	ANK3, ADCY6	125	3	20240	107.94667	1	1	1
**GOTERM_BP_I**	**GO:0030182**	**neuron differentiation**	**6**	**4.545455**	0.019871	**IRX2, GDNF, SHC1, NOVA2, PAX6, WNT4**	**125**	**253**	**20240**	**3.84**	**1**	**1**	**1**
**GOTERM_BP_I**	**GO:0050729**	**positive regulation of inflammatory response**	**4**	**3.030303**	0.022996	**CEBPA, CD47, LDLR, CX3CL1**	**125**	**99**	**20240**	**6.5422222**	**1**	**1**	**1**
GOTERM_BP_I	GO:2001234	negative regulation of apoptotic signaling pathway	3	2.272727	0.024954	PSEN2, CX3CL1, WNT4	125	40	20240	12.144	1	1	1
GOTERM_BP_I	GO:0006397	mRNA processing	7	5.30303	0.025904	SYNCRIP, PPIL1, RBM25, CELF1, NOVA1, NOVA2, CELF6	125	368	20240	3.08	1	1	1
GOTERM_BP_I	GO:0001933	negative regulation of protein phosphorylation	4	3.030303	0.026772	PPP2CA, MYADM, PSEN2, PAX6	125	105	20240	6.168381	1	1	1
**GOTERM_BP_I**	**GO:0010976**	**positive regulation of neuron projection development**	**5**	**3.787879**	0.027169	**RGS2, ANKRD27, ITGA3, STMN2, CX3CL1**	**125**	**185**	**20240**	**4.3762162**	**1**	**1**	**1**
GOTERM_BP_I	GO:0001656	metanephros development	3	2.272727	0.027329	IRX2, GDNF, WNT4	125	42	20240	11.565714	1	1	1
**GOTERM_BP_I**	**GO:0010634**	**positive regulation of epithelial cell migration**	**3**	**2.272727**	0.027329	**ITGA3, PRKCE, PPM1F**	**125**	**42**	**20240**	**11.565714**	**1**	**1**	**1**
**GOTERM_BP_I**	**GO:0007528**	**neuromuscular junction development**	**3**	**2.272727**	0.029791	**CACNB1, CACNA2D2, ANK3**	**125**	**44**	**20240**	**11.04**	**1**	**1**	**1**
GOTERM_BP_I	GO:0008380	RNA splicing	6	4.545455	0.029907	SYNCRIP, PPIL1, RBM25, CELF1, NOVA1, NOVA2	125	282	20240	3.4451064	1	1	1
**GOTERM_BP_I**	**GO:0007411**	**axon guidance**	**5**	**3.787879**	0.03006	**KLF7, GAP43, MYCBP2, PAX6, ANK3**	**125**	**191**	**20240**	**4.2387435**	**1**	**1**	**1**
GOTERM_BP_I	GO:0060676	ureteric bud formation	2	1.515152	0.030262	GREM1, GDNF	125	5	20240	64.768	1	1	1
GOTERM_BP_I	GO:0043547	positive regulation of GTPase activity	5	3.787879	0.030559	RASA1, RAPGEF5, EVI5, CX3CL1, WNT4	125	192	20240	4.2166667	1	1	1
GOTERM_BP_I	GO:0000165	MAPK cascade	4	3.030303	0.032291	MEF2A, PPM1L, PRKCE, BRAF	125	113	20240	5.7316814	1	1	1
**GOTERM_BP_I**	**GO:0007420**	**brain development**	**6**	**4.545455**	0.033569	**MED1, RGS2, SYT1, SLC1A1, PAX6, GRHL2**	**125**	**291**	**20240**	**3.3385567**	**1**	**1**	**1**
GOTERM_BP_I	GO:0000902	cell morphogenesis	4	3.030303	0.033757	GREM1, MED1, NOX4, SIDT2	125	115	20240	5.632	1	1	1
**GOTERM_BP_I**	**GO:1902667**	**regulation of axon guidance**	**2**	**1.515152**	0.036205	**NOVA2, MYCBP2**	**125**	**6**	**20240**	**53.973333**	**1**	**1**	**1**
GOTERM_BP_I	GO:0008203	cholesterol metabolic process	4	3.030303	0.036791	CEBPA, INSIG2, DHCR24, LDLR	125	119	20240	5.4426891	1	1	1
GOTERM_BP_I	GO:0001658	branching involved in ureteric bud morphogenesis	3	2.272727	0.037675	GDNF, NPNT, WNT4	125	50	20240	9.7152	1	1	1
**GOTERM_BP_I**	**GO:0007409**	**axonogenesis**	**4**	**3.030303**	0.038359	**KLF7, BCL11B, PAX6, ANK3**	**125**	**121**	**20240**	**5.3527273**	**1**	**1**	**1**
GOTERM_BP_I	GO:0006357	regulation of transcription from RNA polymerase II promoter	17	12.87879	0.040129	MEF2A, MED1, IRX2, CEBPA, BCL11B, PRRX1, ATP2B4, ARID5B, PAX6, A	125	1627	20240	1.69185	1	1	1
GOTERM_BP_I	GO:0007507	heart development	6	4.545455	0.040271	MEF2A, MED1, PPP3R1, ITGA3, SHC1, GNA11	125	306	20240	3.174902	1	1	1
GOTERM_BP_I	GO:0010894	negative regulation of steroid biosynthetic process	2	1.515152	0.042111	INSIG2, WNT4	125	7	20240	46.262857	1	1	1
**GOTERM_BP_I**	**GO:0021902**	**commitment of neuronal cell to specific neuron type in forebrain**	**2**	**1.515152**	0.042111	**BCL11B, PAX6**	**125**	**7**	**20240**	**46.262857**	**1**	**1**	**1**
GOTERM_BP_I	GO:0003337	mesenchymal to epithelial transition involved in metanephros morphogenesis	2	1.515152	0.042111	GREM1, GDNF	125	7	20240	46.262857	1	1	1
GOTERM_BP_I	GO:1903078	positive regulation of protein localization to plasma membrane	3	2.272727	0.047754	ITGA3, ATP2B4, PTPN9	125	57	20240	8.5221053	1	1	1
GOTERM_BP_I	GO:0048538	thymus development	3	2.272727	0.047754	BCL11B, PSEN2, BRAF	125	57	20240	8.5221053	1	1	1
**GOTERM_BP_I**	**GO:1903979**	**negative regulation of microglial cell activation**	**2**	**1.515152**	0.047982	**LDLR, CX3CL1**	**125**	**8**	**20240**	**40.48**	**1**	**1**	**1**

Table S18. KEGG pathway analysis of miR-183C targets in the TG of miR-183C KO strain.

Category	Term		Count	%	PValue	Genes	List Total	Pop Hits	Pop Total	Fold Enricl	Bonferron	Benjamini	FDR
KEGG_PATHWAY	mmu04022	cGMP-PKG signaling pathway	7	5.30303	8.70E-04	MEF2A, PPP3R1, RGS2, GNA11, PRKCE, ATP2B4, ADCY6	61	173	9212	6.11049	0.144202	0.155653	0.155653
KEGG_PATHWAY	mmu04010	MAPK signaling pathway	8	6.060606	0.003306	CACNB1, MAP3K3, PPP3R1, GDNF, RASA1, CACNA2D2, BRAF, GNG12	61	302	9212	4.000434	0.447202	0.256397	0.256397
**KEGG_PATHWAY**	**mmu04925**	**Aldosterone synthesis and secretion**	**5**	**3.787879**	**0.004297**	**GNA11, PRKCE, ATP2B4, LDLR, ADCY6**	**61**	**102**	**9212**	**7.402764**	**0.537379**	**0.256397**	**0.256397**
**KEGG_PATHWAY**	**mmu04921**	**Oxytocin signaling pathway**	**5**	**3.787879**	**0.017377**	**CACNB1, PPP3R1, RGS2, CACNA2D2, ADCY6**	**61**	**153**	**9212**	**4.935176**	**0.956619**	**0.460731**	**0.460731**
KEGG_PATHWAY	mmu04261	Adrenergic signaling in cardiomyocytes	5	3.787879	0.018527	CACNB1, PPP2CA, ATP2B4, CACNA2D2, ADCY6	61	156	9212	4.840269	0.964825	0.460731	0.460731
**KEGG_PATHWAY**	**mmu04512**	**ECM-receptor interaction**	**4**	**3.030303**	**0.020078**	**SV2C, ITGA3, CD47, NPNT**	**61**	**89**	**9212**	**6.787254**	**0.973497**	**0.460731**	**0.460731**
KEGG_PATHWAY	mmu04934	Cushing syndrome	5	3.787879	0.020968	GNA11, BRAF, LDLR, ADCY6, WNT4	61	162	9212	4.661	0.977476	0.460731	0.460731
KEGG_PATHWAY	mmu01522	Endocrine resistance	4	3.030303	0.022528	MED1, SHC1, BRAF, ADCY6	61	93	9212	6.495329	0.98307	0.460731	0.460731
KEGG_PATHWAY	mmu05414	Dilated cardiomyopathy	4	3.030303	0.023165	CACNB1, ITGA3, CACNA2D2, ADCY6	61	94	9212	6.42623	0.984935	0.460731	0.460731
KEGG_PATHWAY	mmu04928	Parathyroid hormone synthesis, secretion and action	4	3.030303	0.033109	MEF2A, GNA11, BRAF, ADCY6	61	108	9212	5.5932	0.997587	0.566109	0.566109
**KEGG_PATHWAY**	**mmu04062**	**Chemokine signaling pathway**	**5**	**3.787879**	**0.036078**	**SHC1, BRAF, GNG12, CX3CL1, ADCY6**	**61**	**192**	**9212**	**3.932719**	**0.998608**	**0.566109**	**0.566109**
**KEGG_PATHWAY**	**mmu04724**	**Glutamatergic synapse**	**4**	**3.030303**	**0.037951**	**PPP3R1, SLC1A1, GNG12, ADCY6**	**61**	**114**	**9212**	**5.298821**	**0.999018**	**0.566109**	**0.566109**
**KEGG_PATHWAY**	**mmu04722**	**Neurotrophin signaling pathway**	**4**	**3.030303**	**0.044035**	**MAP3K3, SHC1, PSEN2, BRAF**	**61**	**121**	**9212**	**4.992277**	**0.999684**	**0.606329**	**0.606329**

Table S19. Biological processes GO analysis of miR-183C targets in the cornea of miR-183C KO strain.

Category	Term		Count	%	PValue	Genes	List Total	Pop Hit	Pop Total	Fold Enrich	Bonferroni	Benjamini	FDR
GOTERM_	GO:0007399	nervous system development	11	11.95652	1.71E-05	ROBO2, GRIP1, MEF2C, NRN1, FUT9, MYT1L, NOVA2, AVIL, DIP2A, PRKD1, RTN4	86	445	20240	5.817612	0.014931	0.015043	0.014958
GOTERM_	GO:0010976	positive regulation of neuron projection development	7	7.608696	1.29E-04	ANKRD27, FUT9, AVIL, DAB2IP, CNTN1, FYN, PRKD1	86	185	20240	8.905091	0.107293	0.044649	0.044395
GOTERM_	GO:0090649	response to oxygen-glucose deprivation	3	3.26087	1.73E-04	SOX2, ZEB2, BNIP3	86	5	20240	141.2093	0.140838	0.044649	0.044395
GOTERM_	GO:0002062	chondrocyte differentiation	5	5.434783	2.03E-04	MEF2C, PRKCA, TGFBI, SLC39A14, SOX5	86	69	20240	17.05426	0.163572	0.044649	0.044395
GOTERM_	GO:2000300	regulation of synaptic vesicle exocytosis	5	5.434783	4.53E-04	NRN1, SV2C, PRKCE, PRKCA, CACNA1E	86	85	20240	13.84405	0.328074	0.079503	0.079051
GOTERM_	GO:0055085	transmembrane transport	9	9.782609	6.41E-04	SLC25A1, CLCN5, SV2C, KCNH5, SLC39A5, CACNA1E, SLC39A1, SLC39A14, SLC16A13	86	454	20240	4.665506	0.430461	0.086473	0.085981
GOTERM_	GO:0030182	neuron differentiation	7	7.608696	6.89E-04	SOX2, MEF2C, FUT9, MYT1L, NOVA2, ELAVL4, RTN4	86	253	20240	6.511628	0.45421	0.086473	0.085981
GOTERM_	GO:0006915	apoptotic process	10	10.86957	0.001902	TOX3, MEF2C, BNIP3, DAB2IP, ELMO1, AATK, PRKCA, UNC5D, NSG1, PRKD1	86	666	20240	3.533766	0.812051	0.208749	0.207561
GOTERM_	GO:0035556	intracellular signal transduction	8	8.695652	0.003256	DUSP1, MAK, PRKCE, FYN, PRKCA, PRKD1, DGKK, TNS1	86	461	20240	4.084145	0.942911	0.304299	0.302566
GOTERM_	GO:0006811	ion transport	9	9.782609	0.003812	CLCN5, KCNH5, CACNA2D1, SLC39A5, CACNA1E, SLC39A1, SLC39A14, GRIA3, ATP6V1C1	86	603	20240	3.512669	0.965018	0.304299	0.302566
GOTERM_	GO:0071577	zinc II ion transmembrane transport	3	3.26087	0.003812	SLC39A5, SLC39A1, SLC39A14	86	22	20240	32.09302	0.965045	0.304299	0.302566
GOTERM_	GO:0006829	zinc II ion transport	3	3.26087	0.004911	SLC39A5, SLC39A1, SLC39A14	86	25	20240	28.24186	0.986732	0.358342	0.356301
GOTERM_	GO:0061178	regulation of insulin secretion involved in cellular response to glucose stimulus	3	3.26087	0.005306	PRKCE, CACNA1E, SIDT2	86	26	20240	27.15564	0.990636	0.358342	0.356301
GOTERM_	GO:0030001	metal ion transport	3	3.26087	0.005715	SLC39A5, SLC39A1, SLC39A14	86	27	20240	26.14987	0.993473	0.35839	0.356349
GOTERM_	GO:0022409	positive regulation of cell-cell adhesion	3	3.26087	0.006138	SOX2, MAGI1, PDE4D	86	28	20240	25.21595	0.995508	0.359251	0.357205
GOTERM_	GO:0030517	negative regulation of axon extension	3	3.26087	0.006574	TRIM46, AATK, RTN4	86	29	20240	24.34643	0.996946	0.360764	0.35871
GOTERM_	GO:0050730	regulation of peptidyl-tyrosine phosphorylation	3	3.26087	0.007489	PRKCE, FYN, PRKCA	86	31	20240	22.77569	0.99864	0.38678	0.384577
GOTERM_	GO:1904520	regulation of myofibroblast cell apoptotic process	2	2.173913	0.008382	SOX2, ZEB2	86	2	20240	235.3488	0.999383	0.408845	0.406516
GOTERM_	GO:0007626	locomotory behavior	4	4.347826	0.014426	ELAVL4, CNTN1, SOBP, CACNA1E	86	121	20240	7.780127	0.999997	0.666646	0.66285
GOTERM_	GO:0001764	neuron migration	4	4.347826	0.028019	MEF2C, TRIM46, FYN, CELSR3	86	156	20240	6.034586	1	1	0.995439
GOTERM_	GO:0010595	positive regulation of endothelial cell migration	3	3.26087	0.02885	ANXA3, PRKCA, PRKD1	86	63	20240	11.20709	1	1	0.995439
GOTERM_	GO:0045766	positive regulation of angiogenesis	4	4.347826	0.030848	ANXA3, PRKCA, PRKD1, RTN4	86	162	20240	5.811082	1	1	0.995439
GOTERM_	GO:0007015	actin filament organization	4	4.347826	0.032317	TPM2, AVIL, ELMO1, TAGLN3	86	165	20240	5.705426	1	1	0.995439
GOTERM_	GO:0071578	zinc II ion transmembrane import	2	2.173913	0.033113	SLC39A5, SLC39A14	86	8	20240	58.83721	1	1	0.995439
GOTERM_	GO:0006688	glycosphingolipid biosynthetic process	2	2.173913	0.037175	FUT9, B4GALNT1	86	9	20240	52.29974	1	1	0.995439
GOTERM_	GO:0007275	multicellular organism development	10	10.86957	0.040593	SOX2, ROBO2, MEF2C, MYT1L, DAB2IP, DIP2A, FYN, UNC5D, HOXD8, CELSR3	86	1098	20240	2.143432	1	1	0.995439
GOTERM_	GO:0045822	negative regulation of heart contraction	2	2.173913	0.04122	PDE4D, PRKCA	86	10	20240	47.06977	1	1	0.995439
GOTERM_	GO:0098887	neurotransmitter receptor transport, endosome to postsynaptic membrane	2	2.173913	0.04122	GRIP1, NSG1	86	10	20240	47.06977	1	1	0.995439
GOTERM_	GO:0007155	cell adhesion	7	7.608696	0.04357	PRKCE, FIBCD1, CNTN1, PRKCA, TGFBI, CELSR3, PCDH17	86	611	20240	2.696304	1	1	0.995439
GOTERM_	GO:0071560	cellular response to transforming growth factor beta stimulus	3	3.26087	0.043603	MEF2C, FYN, SOX5	86	79	20240	8.937298	1	1	0.995439
GOTERM_	GO:0046777	protein autophosphorylation	4	4.347826	0.048356	MAK, FYN, PRKCA, PRKD1	86	194	20240	4.852553	1	1	0.995439
GOTERM_	GO:0043524	negative regulation of neuron apoptotic process	4	4.347826	0.049583	TOX3, MEF2C, FYN, SNCB	86	196	20240	4.803037	1	1	0.995439
GOTERM_	GO:0045165	cell fate commitment	3	3.26087	0.049721	SOX2, MEF2C, SOX5	86	85	20240	8.30643	1	1	0.995439

Table S20. KEGG pathway analysis of miR-183C targets in the cornea of miR-183C KO strain.

Category	Term	Count	%	PValue	Genes	List Total	Pop Hits	Pop Total	Fold Enrich	Bonferroni	Benjamini	FDR
**KEGG_PAT**	**mmu04360:Axon guidance**	**5**	**5.434783**	0.004219	**ROBO2, FYN, PRKCA, UNC5D, SSH2**	**35**	**181**	**9212**	**7.270718**	**0.4301**	**0.561107**	**0.561107**
**KEGG_PAT**	**mmu04010:MAPK signaling pathway**	**5**	**5.434783**	0.024221	**MEF2C, DUSP1, CACNA2D1, PRKCA, CACNA1E**	**35**	**302**	**9212**	**4.357616**	**0.961651**	**0.880408**	**0.880408**
KEGG_PAT	mmu05032:Morphine addiction	3	3.26087	0.044137	GNG5, PDE4D, PRKCA	35	91	9212	8.676923	0.99753	0.880408	0.880408
**KEGG_PAT**	**mmu04713:Circadian entrainment**	**3**	**3.26087**	0.050421	**GNG5, PRKCA, GRIA3**	**35**	**98**	**9212**	**8.057143**	**0.998973**	**0.880408**	**0.880408**
KEGG_PAT	mmu04925:Aldosterone synthesis and secretion	3	3.26087	0.054149	PRKCE, PRKCA, PRKD1	35	102	9212	7.741176	0.999391	0.880408	0.880408
KEGG_PAT	mmu04928:Parathyroid hormone synthesis, secretion and action	3	3.26087	0.05992	MEF2C, PDE4D, PRKCA	35	108	9212	7.311111	0.99973	0.880408	0.880408
**KEGG_PAT**	**mmu04725:Cholinergic synapse**	**3**	**3.26087**	0.063881	**GNG5, FYN, PRKCA**	**35**	**112**	**9212**	**7.05**	**0.999846**	**0.880408**	**0.880408**
**KEGG_PAT**	**mmu04724:Glutamatergic synapse**	**3**	**3.26087**	0.065895	**GNG5, PRKCA, GRIA3**	**35**	**114**	**9212**	**6.926316**	**0.999884**	**0.880408**	**0.880408**
KEGG_PAT	mmu05012:Parkinson disease	4	4.347826	0.072604	DUSP1, SLC39A5, SLC39A1, SLC39A14	35	264	9212	3.987879	0.999956	0.880408	0.880408
**KEGG_PAT**	**mmu04071:Sphingolipid signaling pathway**	**3**	**3.26087**	0.078411	**PRKCE, FYN, PRKCA**	**35**	**126**	**9212**	**6.266667**	**0.999981**	**0.880408**	**0.880408**
**KEGG_PAT**	**mmu04726:Serotonergic synapse**	**3**	**3.26087**	0.083833	**GNG5, DUSP1, PRKCA**	**35**	**131**	**9212**	**6.027481**	**0.999991**	**0.880408**	**0.880408**
**KEGG_PAT**	**mmu04728:Dopaminergic synapse**	**3**	**3.26087**	0.088253	**GNG5, PRKCA, GRIA3**	**35**	**135**	**9212**	**5.848889**	**0.999995**	**0.880408**	**0.880408**
**KEGG_PAT**	**mmu04371:Apelin signaling pathway**	**3**	**3.26087**	0.090489	**MEF2C, GNG5, PRKCE**	**35**	**137**	**9212**	**5.763504**	**0.999997**	**0.880408**	**0.880408**

Table S21. Biological processes GO analysis of miR-183C targets in the TG of both SNS-CKO and miR-183C KO strains.

Category	Term	Count	%	PValue	Genes	List Total	Pop Hits	Pop Total	Fold Enrichment	Bonferroni	Benjamini	FDR
GOTERM_	GO:0045944~positive regulation of transcription from RNA polymerase II promoter	24	15.09433962	3.79E-05	MEF2A, MED1, CEBPA, RARG, BCL11B, PRRX1, PAX6, ARID3A, EBF3, FOXO3, NPNT, BAZ1B, GRHL2, CX3CL1, GREM1, KLF7, PPP3R1, ZEB1, GDNF, MORF4L2, NCK2, WNT2, ZFP451, KDM6A	148	1257	20240	2.611107528	0.056897194	0.049077	0.048982
GOTERM_	GO:0051496~positive regulation of stress fiber assembly	6	3.773584906	6.34E-05	NOX4, BRAF, CD47, TGFBR1, PPM1F, WNT4	148	58	20240	14.1472507	0.093494124	0.049077	0.048982
GOTERM_	GO:0035556~intracellular signal transduction	13	8.176100629	1.41E-04	MAP3K3, SHC1, PNPLA8, PRKCE, PSEN2, BRAF, TGFBR1, ADCY6, ZFP36, PLEKHM1, FYN, DGKK, RAPGEF5	148	461	20240	3.85648121	0.196263587	0.059039	0.058924
GOTERM_	GO:0010628~positive regulation of gene expression	15	9.433962264	1.53E-04	MEF2A, GRIA1, MED1, CEBPA, RARG, ITGA3, CELF1, MSN, PAX6, BRAF, ANK3, TGFBR1, PPM1F, LDLR, KDM6A	148	612	20240	3.351881293	0.210355323	0.059039	0.058924
**GOTERM_**	**GO:0071560~cellular response to transforming growth factor beta stimulus**	**6**	**3.773584906**	**2.76E-04**	**ZEB1, NOX4, FYN, WNT2, TGFBR1, WNT4**	**148**	**79**	**20240**	**10.38658912**	**0.347755938**	**0.085455**	**0.08529**
GOTERM_	GO:0045893~positive regulation of transcription, DNA-templated	15	9.433962264	9.61E-04	MEF2A, MAP2K3, MED1, CEBPA, SHC1, PAX6, EBF3, FOXO3, GRHL2, TGFBR1, GREM1, KLF7, MORF4L2, ZKSCAN17, WNT4	148	736	20240	2.787162162	0.774207277	0.246773	0.246294
GOTERM_	GO:0000122~negative regulation of transcription from RNA polymerase II promoter	18	11.32075472	0.001344064	MEF2A, MED1, IRX2, CEBPA, RARG, PRRX1, NKAP, PSEN2, ARID5B, PAX6, FOXO3, ZBTB41, PPP2CA, KLF7, ZFP36, ZEB1, LCOR, MDM4	148	1031	20240	2.387605841	0.875152922	0.246773	0.246294
GOTERM_	GO:0000165~MAPK cascade	6	3.773584906	0.001412669	MEF2A, MAP2K3, ZFP36, PPM1L, PRKCE, BRAF	148	113	20240	7.261420713	0.887740703	0.246773	0.246294
GOTERM_	GO:0000381~regulation of alternative mRNA splicing, via spliceosome	5	3.144654088	0.001435653	RBM25, CELF1, NOVA1, NOVA2, CELF6	148	67	20240	10.20572812	0.891667617	0.246773	0.246294
GOTERM_	GO:0007507~heart development	9	5.660377358	0.001809947	MEF2A, MAP2K3, MED1, PPP3R1, ITGA3, SHC1, GNA11, TGFBR1, KDM6A	148	306	20240	4.022257552	0.939343281	0.279999	0.279456
**GOTERM_**	**GO:0010976~positive regulation of neuron projection development**	**7**	**4.402515723**	**0.002345408**	**EHD1, RGS2, ANKRD27, ITGA3, STMN2, FYN, CX3CL1**	**148**	**185**	**20240**	**5.174579985**	**0.973552891**	**0.32985**	**0.32921**
GOTERM_	GO:0043066~negative regulation of apoptotic process	13	8.176100629	0.003657853	MED1, BCL11B, SHC1, PRKCE, PSEN2, DHCR24, BRAF, TGFBR1, CX3CL1, GREM1, RASA1, CASP2, MDM4	148	671	20240	2.649534781	0.996548939	0.471558	0.470644
**GOTERM_**	**GO:0007528~neuromuscular junction development**	**4**	**2.51572327**	**0.003997876**	**CACNB1, CACNA2D2, ANK3, KY**	**148**	**44**	**20240**	**12.43243243**	**0.997964696**	**0.475747**	**0.474825**
GOTERM_	GO:0010811~positive regulation of cell-substrate adhesion	4	2.51572327	0.004534167	ITGA3, PRKCE, NPNT, PPM1F	148	46	20240	11.89189189	0.99911534	0.501025	0.500054
GOTERM_	GO:0033077~T cell differentiation in thymus	4	2.51572327	0.005416753	BCL11B, NKAP, BRAF, WNT4	148	49	20240	11.16381688	0.999775693	0.526913	0.525891
**GOTERM_**	**GO:0007420~brain development**	**8**	**5.031446541**	**0.00544965**	**ALK, MED1, RGS2, SYT1, SLC1A1, CASP2, PAX6, GRHL2**	**148**	**291**	**20240**	**3.759635925**	**0.999786882**	**0.526913**	**0.525891**
GOTERM_	GO:0045184~establishment of protein localization	4	2.51572327	0.00605848	SLC1A1, ANK3, NPNT, KPNB1	148	51	20240	10.72602014	0.999917357	0.551322	0.550253
**GOTERM_**	**GO:0003407~neural retina development**	**3**	**1.886792453**	**0.0066335**	**USP45, ATP2B4, CASP2**	**148**	**17**	**20240**	**24.13354531**	**0.999966239**	**0.570113**	**0.569007**
GOTERM_	GO:0015031~protein transport	12	7.547169811	0.007602606	RD3, GGA2, EHD1, IPO9, PGAP1, ANKRD27, PLEKHM1, PSEN2, HOOK3, SNX30, VPS36, KPNB1	148	646	20240	2.540373191	0.999992542	0.619012	0.617812
GOTERM_	GO:0048538~thymus development	4	2.51572327	0.008247888	BCL11B, PSEN2, BRAF, TGFBR1	148	57	20240	9.596965386	0.999997273	0.637974	0.636737
GOTERM_	GO:0010468~regulation of gene expression	10	6.289308176	0.008961644	RARG, BCL11B, GDNF, NOVA1, NKAP, PAX6, GRHL2, TGFBR1, KDM6A, ZFP451	148	484	20240	2.825552826	0.999999105	0.660174	0.658894
GOTERM_	GO:0006915~apoptotic process	12	7.547169811	0.009431792	GREM1, MEF2A, RARG, RBM25, BCL11B, FEM1B, CASP2, CD47, CLPTM1L, FOXO3, TGFBR1, PPM1F	148	666	20240	2.464085707	0.99999957	0.663226	0.66194
GOTERM_	GO:0006606~protein import into nucleus	5	3.144654088	0.01051371	MED1, PPP3R1, IPO9, NUP50, KPNB1	148	117	20240	5.844305844	0.999999921	0.678922	0.677606
**GOTERM_**	**GO:0030182~neuron differentiation**	**7**	**4.402515723**	**0.010532731**	**IRX2, GDNF, SHC1, NOVA2, PAX6, WNT2, WNT4**	**148**	**253**	**20240**	**3.783783784**	**0.999999923**	**0.678922**	**0.677606**

GOTERM_	GO:0060324~face development	3	1.886792453	0.011003217	RARG, BRAF, GRHL2	148	22	20240	18.64864865	0.999999963	0.680879	0.679559
GOTERM_	GO:0007169~transmembrane receptor protein tyrosine kinase signaling pathway	5	3.144654088	0.011784197	ALK, GDNF, SHC1, FYN, SAMD12	148	121	20240	5.651105651	0.999999989	0.70116	0.6998
GOTERM_	GO:0071539~protein localization to centrosome	3	1.886792453	0.013022869	CCDC14, CEP250, HOOK3	148	24	20240	17.09459459	0.999999998	0.719514	0.718118
GOTERM_	GO:0032331~negative regulation of chondrocyte differentiation	3	1.886792453	0.013022869	GREM1, RARG, TGFBR1	148	24	20240	17.09459459	0.999999998	0.719514	0.718118
GOTERM_	GO:0006357~regulation of transcription from RNA polymerase II promoter	21	13.20754717	0.014081574	MEF2A, MED1, IRX2, CEBPA, BCL11B, PRRX1, ATP2B4, ARID5B, PAX6, ARID3A, EBF3, FOXO3, CHD2, GRHL2, ZBTB41, KLF7, ZFP36, ZEB1, LCOR, MDM4, ZKSCAN17	148	1627	20240	1.7651456	1	0.734372	0.732948
GOTERM_	GO:0031022~nuclear migration along microfilament	2	1.257861635	0.014473299	SUN2, SYNE2	148	2	20240	136.7567568	1	0.734372	0.732948
GOTERM_	GO:0043410~positive regulation of MAPK cascade	6	3.773584906	0.015189801	ALK, MAP2K3, SHC1, PRKCE, TGFBR1, CX3CL1	148	199	20240	4.123319299	1	0.734372	0.732948
**GOTERM_**	**GO:0036120~cellular response to platelet-derived growth factor stimulus**	**3**	**1.886792453**	**0.015190627**	**PRKCE, RASA1, FYN**	**148**	**26**	**20240**	**15.77962578**	**1**	**0.734372**	**0.732948**
**GOTERM_**	**GO:0007399~nervous system development**	**9**	**5.660377358**	**0.016167941**	**ALK, MEF2A, GAP43, ZEB1, GDNF, NOVA1, NOVA2, TGFBR1, MTSS1**	**148**	**445**	**20240**	**2.765866991**	**1**	**0.742951**	**0.74151**
**GOTERM_**	**GO:0097150~neuronal stem cell population maintenance**	**3**	**1.886792453**	**0.016328587**	**PRRX1, HOOK3, FOXO3**	**148**	**27**	**20240**	**15.1951952**	**1**	**0.742951**	**0.74151**
GOTERM_	GO:0048844~artery morphogenesis	3	1.886792453	0.018709815	PRRX1, LDLR, TGFBR1	148	29	20240	14.1472507	1	0.826974	0.82537
GOTERM_	GO:0060218~hematopoietic stem cell differentiation	3	1.886792453	0.021227713	MED1, ZFP36, CHD2	148	31	20240	13.23452485	1	0.84708	0.845437
GOTERM_	GO:0043368~positive T cell selection	2	1.257861635	0.021631735	BCL11B, BRAF	148	3	20240	91.17117117	1	0.84708	0.845437
GOTERM_	GO:0072660~maintenance of protein location in plasma membrane	2	1.257861635	0.021631735	ANK3, ADCY6	148	3	20240	91.17117117	1	0.84708	0.845437
**GOTERM_**	**GO:0021817~nucleokinesis involved in cell motility in cerebral cortex radial glia guided migration**	**2**	**1.257861635**	**0.021631735**	**SUN2, SYNE2**	**148**	**3**	**20240**	**91.17117117**	**1**	**0.84708**	**0.845437**
GOTERM_	GO:0060021~palate development	4	2.51572327	0.021902513	PRRX1, INSIG2, ARID5B, TGFBR1	148	82	20240	6.671061305	1	0.84708	0.845437
GOTERM_	GO:2001240~negative regulation of extrinsic apoptotic signaling pathway in absence of ligand	3	1.886792453	0.022536536	GDNF, FYN, CX3CL1	148	32	20240	12.82094595	1	0.850342	0.848693
GOTERM_	GO:0006470~protein dephosphorylation	5	3.144654088	0.023937213	PPP2CA, PPP3R1, PPM1L, PTPN9, PPM1F	148	150	20240	4.558558559	1	0.865208	0.86353
GOTERM_	GO:0045165~cell fate commitment	4	2.51572327	0.024049083	GAP43, PAX6, WNT2, WNT4	148	85	20240	6.435612083	1	0.865208	0.86353
GOTERM_	GO:0071333~cellular response to glucose stimulus	4	2.51572327	0.025541189	PPP2CA, NOX4, NPTX1, FOXO3	148	87	20240	6.287666977	1	0.898005	0.896264
**GOTERM_**	**GO:0050680~negative regulation of epithelial cell proliferation**	**4**	**2.51572327**	**0.027870794**	**ZEB1, CELF1, PAX6, MTSS1**	**148**	**90**	**20240**	**6.078078078**	**1**	**0.958136**	**0.956278**
**GOTERM_**	**GO:0030900~forebrain development**	**4**	**2.51572327**	**0.03199643**	**ZEB1, PSEN2, FYN, PAX6**	**148**	**95**	**20240**	**5.758179232**	**1**	**0.984806**	**0.982897**
GOTERM_	GO:0042593~glucose homeostasis	5	3.144654088	0.033533354	KLF7, CEBPA, PAX6, FOXO3, SIDT2	148	167	20240	4.094513675	1	0.984806	0.982897
GOTERM_	GO:2001234~negative regulation of apoptotic signaling pathway	3	1.886792453	0.034133877	PSEN2, CX3CL1, WNT4	148	40	20240	10.25675676	1	0.984806	0.982897
**GOTERM_**	**GO:0050729~positive regulation of inflammatory response**	**4**	**2.51572327**	**0.035514176**	**CEBPA, CD47, LDLR, CX3CL1**	**148**	**99**	**20240**	**5.525525526**	**1**	**0.984806**	**0.982897**
GOTERM_	GO:0048701~embryonic cranial skeleton morphogenesis	3	1.886792453	0.035716678	PRRX1, GRHL2, TGFBR1	148	41	20240	10.00659196	1	0.984806	0.982897
GOTERM_	GO:0010730~negative regulation of hydrogen peroxide biosynthetic process	2	1.257861635	0.035794043	MPV17L, FYN	148	5	20240	54.7027027	1	0.984806	0.982897
GOTERM_	GO:0060676~ureteric bud formation	2	1.257861635	0.035794043	GREM1, GDNF	148	5	20240	54.7027027	1	0.984806	0.982897
GOTERM_	GO:0001656~metanephros development	3	1.886792453	0.037327402	IRX2, GDNF, WNT4	148	42	20240	9.768339768	1	0.984806	0.982897
**GOTERM_**	**GO:0010634~positive regulation of epithelial cell migration**	**3**	**1.886792453**	**0.037327402**	**ITGA3, PRKCE, PPM1F**	**148**	**42**	**20240**	**9.768339768**	**1**	**0.984806**	**0.982897**
GOTERM_	GO:1904646~cellular response to beta-amyloid	3	1.886792453	0.038965567	CACNB1, FYN, FOXO3	148	43	20240	9.541169076	1	0.984806	0.982897
GOTERM_	GO:0008285~negative regulation of cell proliferation	8	5.031446541	0.038977699	CEBPA, RARG, ZEB1, BCL11B, CELF1, NOX4, DHCR24, PAX6	148	433	20240	2.526683728	1	0.984806	0.982897

**GOTERM_**	**GO:0045664~regulation of neuron differentiation**	**3**	**1.886792453**	**0.040630695**	**ALK, BCL11B, PAX6**	**148**	**44**	**20240**	**9.324324324**	**1**	**0.984806**	**0.982897**
GOTERM_	GO:0001933~negative regulation of protein phosphorylation	4	2.51572327	0.041148746	PPP2CA, MYADM, PSEN2, PAX6	148	105	20240	5.20978121	1	0.984806	0.982897
**GOTERM_**	**GO:0007616~long-term memory**	**3**	**1.886792453**	**0.042322314**	**GRIA1, CHST10, LDLR**	**148**	**45**	**20240**	**9.117117117**	**1**	**0.984806**	**0.982897**
**GOTERM_**	**GO:1902667~regulation of axon guidance**	**2**	**1.257861635**	**0.042798653**	**NOVA2, MYCBP2**	**148**	**6**	**20240**	**45.58558559**	**1**	**0.984806**	**0.982897**
GOTERM_	GO:0071242~cellular response to ammonium ion	2	1.257861635	0.042798653	GRIA1, SLC1A1	148	6	20240	45.58558559	1	0.984806	0.982897
**GOTERM_**	**GO:0031175~neuron projection development**	**5**	**3.144654088**	**0.04313866**	**EHD1, GDNF, SHC1, STMN2, FYN**	**148**	**181**	**20240**	**3.77781096**	**1**	**0.984806**	**0.982897**
**GOTERM_**	**GO:1990090~cellular response to nerve growth factor stimulus**	**3**	**1.886792453**	**0.044039957**	**EHD1, STMN2, FOXO3**	**148**	**46**	**20240**	**8.918918919**	**1**	**0.984806**	**0.982897**
GOTERM_	GO:0008284~positive regulation of cell proliferation	10	6.289308176	0.045233171	GREM1, MED1, RARG, GDNF, SHC1, MDM4, CD47, WNT2, TGFBR1, CX3CL1	148	643	20240	2.126854693	1	0.984806	0.982897
GOTERM_	GO:0097192~extrinsic apoptotic signaling pathway in absence of ligand	3	1.886792453	0.045783163	CASP2, FOXO3, CX3CL1	148	47	20240	8.729154687	1	0.984806	0.982897
GOTERM_	GO:0001837~epithelial to mesenchymal transition	3	1.886792453	0.045783163	PPP3R1, TGFBR1, WNT4	148	47	20240	8.729154687	1	0.984806	0.982897
**GOTERM_**	**GO:0002088~lens development in camera-type eye**	**3**	**1.886792453**	**0.045783163**	**MED1, PAX6, TGFBR1**	**148**	**47**	**20240**	**8.729154687**	**1**	**0.984806**	**0.982897**
GOTERM_	GO:0030335~positive regulation of cell migration	6	3.773584906	0.04614881	SUN2, MYADM, TGFBR1, CX3CL1, SYNE2, PPM1F	148	268	20240	3.061718435	1	0.984806	0.982897
GOTERM_	GO:0060070~canonical Wnt signaling pathway	4	2.51572327	0.048256095	FOXO3, WNT2, KDM6A, WNT4	148	112	20240	4.884169884	1	0.984806	0.982897
GOTERM_	GO:0071364~cellular response to epidermal growth factor stimulus	3	1.886792453	0.049344442	MED1, ZFP36, RASA1	148	49	20240	8.372862659	1	0.984806	0.982897
**GOTERM_**	**GO:0021902~commitment of neuronal cell to specific neuron type in forebrain**	**2**	**1.257861635**	**0.04975272**	**BCL11B, PAX6**	**148**	**7**	**20240**	**39.07335907**	**1**	**0.984806**	**0.982897**
GOTERM_	GO:0010894~negative regulation of steroid biosynthetic process	2	1.257861635	0.04975272	INSIG2, WNT4	148	7	20240	39.07335907	1	0.984806	0.982897
GOTERM_	GO:0003337~mesenchymal to epithelial transition involved in metanephros morphogenesis	2	1.257861635	0.04975272	GREM1, GDNF	148	7	20240	39.07335907	1	0.984806	0.982897
**GOTERM_**	**GO:0007411~axon guidance**	**5**	**3.144654088**	**0.050791008**	**KLF7, GAP43, MYCBP2, PAX6, ANK3**	**148**	**191**	**20240**	**3.58001981**	**1**	**0.984806**	**0.982897**

Table S22. KEGG pathway analysis of miR-183C targets in the TG of both SNS-CKO and miR-183C KO strains.

Category	Term	Count	%	PValue	Genes	.ist Tota	Pop Hits	Pop Total	d Enrichm	Bonferroni	Benjamini	FDR
KEGG_PAT	mmu04010:MAPK signaling pathway	10	6.289308	6.60E-04	CACNB1, MAP2K3, MAP3K3, PPP3R1, GDNF, RASA1, CACNA2D2, BRAF, GNG12, TGFBR1	75	302	9212	4.067108	0.11957	0.127303	0.121366
KEGG_PAT	mmu04022:cGMP-PKG signaling pathway	7	4.402516	0.002593	MEF2A, PPP3R1, RGS2, GNA11, PRKCE, ATP2B4, ADCY6	75	173	9212	4.969865	0.394151	0.250237	0.238568
KEGG_PAT	mmu04921:Oxytocin signaling pathway	6	3.773585	0.007602	CACNB1, PPP3R1, RGS2, KCNJ14, CACNA2D2, ADCY6	75	153	9212	4.816732	0.770694	0.317871	0.303048
KEGG_PAT	mmu04925:Aldosterone synthesis and secretion	5	3.144654	0.009015	GNA11, PRKCE, ATP2B4, LDLR, ADCY6	75	102	9212	6.020915	0.825856	0.317871	0.303048
KEGG_PAT	mmu04934:Cushing syndrome	6	3.773585	0.009606	GNA11, BRAF, LDLR, WNT2, ADCY6, WNT4	75	162	9212	4.549136	0.844788	0.317871	0.303048
KEGG_PAT	mmu04730:Long-term depression	4	2.515723	0.012273	PPP2CA, GRIA1, GNA11, BRAF	75	60	9212	8.188444	0.907759	0.317871	0.303048
**KEGG_PAT**	**mmu04725:Cholinergic synapse**	**5**	**3.144654**	**0.012411**	**GNA11, KCNJ14, FYN, GNG12, ADCY6**	**75**	**112**	**9212**	**5.483333**	**0.910214**	**0.317871**	**0.303048**
**KEGG_PAT**	**mmu04724:Glutamatergic synapse**	**5**	**3.144654**	**0.013176**	**GRIA1, PPP3R1, SLC1A1, GNG12, ADCY6**	**75**	**114**	**9212**	**5.387135**	**0.922685**	**0.317871**	**0.303048**
**KEGG_PAT**	**mmu04722:Neurotrophin signaling pathway**	**5**	**3.144654**	**0.016088**	**MAP3K3, SHC1, PSEN2, BRAF, FOXO3**	**75**	**121**	**9212**	**5.075482**	**0.956295**	**0.345004**	**0.328916**
**KEGG_PAT**	**mmu04062:Chemokine signaling pathway**	**6**	**3.773585**	**0.018868**	**SHC1, BRAF, FOXO3, GNG12, CX3CL1, ADCY6**	**75**	**192**	**9212**	**3.838333**	**0.974684**	**0.364151**	**0.34717**
KEGG_PAT	mmu04371:Apelin signaling pathway	5	3.144654	0.024179	MEF2A, PRKCE, GNG12, TGFBR1, ADCY6	75	137	9212	4.482725	0.99112	0.402853	0.384067
KEGG_PAT	mmu05200:Pathways in cancer	10	6.289308	0.029161	ALK, CEBPA, ITGA3, GNA11, BRAF, GNG12, WNT2, TGFBR1, ADCY6, WNT4	75	543	9212	2.262001	0.996693	0.402853	0.384067
KEGG_PAT	mmu05226:Gastric cancer	5	3.144654	0.032293	SHC1, BRAF, WNT2, TGFBR1, WNT4	75	150	9212	4.094222	0.998227	0.402853	0.384067
KEGG_PAT	mmu05235:PD-L1 expression and PD-1 checkpoint pathway in cancer	4	2.515723	0.033583	ALK, MAP2K3, MAP3K3, PPP3R1	75	88	9212	5.58303	0.99863	0.402853	0.384067
KEGG_PAT	mmu04512:ECM-receptor interaction	4	2.515723	0.034559	SV2C, ITGA3, CD47, NPNT	75	89	9212	5.5203	0.998873	0.402853	0.384067
KEGG_PAT	mmu04912:GnRH signaling pathway	4	2.515723	0.03555	MAP2K3, MAP3K3, GNA11, ADCY6	75	90	9212	5.458963	0.999075	0.402853	0.384067
KEGG_PAT	mmu04261:Adrenergic signaling in cardiomyocytes	5	3.144654	0.036517	CACNB1, PPP2CA, ATP2B4, CACNA2D2, ADCY6	75	156	9212	3.936752	0.999238	0.402853	0.384067
KEGG_PAT	mmu01522:Endocrine resistance	4	2.515723	0.03861	MED1, SHC1, BRAF, ADCY6	75	93	9212	5.282867	0.999499	0.402853	0.384067
KEGG_PAT	mmu05414:Dilated cardiomyopathy	4	2.515723	0.039659	CACNB1, ITGA3, CACNA2D2, ADCY6	75	94	9212	5.226667	0.999594	0.402853	0.384067
KEGG_PAT	mmu05225:Hepatocellular carcinoma	5	3.144654	0.051027	SHC1, BRAF, WNT2, TGFBR1, WNT4	75	174	9212	3.529502	0.999959	0.492415	0.469453
KEGG_PAT	mmu04928:Parathyroid hormone synthesis, secretion and action	4	2.515723	0.05584	MEF2A, GNA11, BRAF, ADCY6	75	108	9212	4.549136	0.999985	0.503682	0.480194
**KEGG_PAT**	**mmu04360:Axon guidance**	**5**	**3.144654**	**0.057415**	**PPP3R1, RASA1, NCK2, FYN, WNT4**	**75**	**181**	**9212**	**3.393002**	**0.999989**	**0.503682**	**0.480194**

Table S23. Biological processes GO analysis of miR-183C targets in the cornea of both SNS-CKO and miR-183C KO strains.

Category	Term	Count	%	PValue	Genes	List Total	Pop Hits	Pop Total	Fold Enrichment	Bonferroni	Benjamini	FDR
GOTERM_	GO:0007399~nervous system development	13	8.496732	6.79E-05	ROBO2, MEF2C, MBNL1, NRN1, MYT1L, AVIL, TSC1, RTN4, GRIP1, FUT9, NOVA2, DIP2A, PRKD1	142	445	20240	4.16395	0.0852317	0.0890814	0.08881
GOTERM_	GO:0010976~positive regulation of neuron projection development	8	5.228758	3.21E-04	ANKRD27, FUT9, AVIL, DAB2IP, CNTN1, FYN, PRKD1, CX3CL1	142	185	20240	6.163685	0.3437748	0.1558997	0.155424
GOTERM_	GO:0030182~neuron differentiation	9	5.882353	4.05E-04	SOX2, MAP2K1, MEF2C, FUT9, MYT1L, NOVA2, ELAVL4, HES1, RTN4	142	253	20240	5.070423	0.4118971	0.1558997	0.155424
GOTERM_	GO:0090649~response to oxygen-glucose deprivation	3	1.960784	4.75E-04	SOX2, ZEB2, BNIP3	142	5	20240	85.52113	0.4640675	0.1558997	0.155424
GOTERM_	GO:0006915~apoptotic process	14	9.150327	7.88E-04	MEF2C, PDCD6, BNIP3, DAB2IP, JADE1, AATK, PRKCA, UNC5D, NSG1, FOXO1, TOX3, ELMO1, PRKD1, CLPTM1L	142	666	20240	2.996236	0.644364	0.206688	0.206058
GOTERM_	GO:0010595~positive regulation of endothelial cell migration	5	3.267974	9.78E-04	ANXA3, PDCD6, PRKCA, PRKD1, RAC1	142	63	20240	11.31232	0.7228524	0.213763	0.213111
GOTERM_	GO:0007155~cell adhesion	13	8.496732	0.001191	CD164, PRKCE, FIBCD1, PRKCA, CX3CL1, PCDH17, CELSR3, CNTN1, HES1, FAT3, TGFBI, RAC1, DGCR2	142	611	20240	3.032664	0.7906501	0.2232595	0.222579
GOTERM_	GO:0002062~chondrocyte differentiation	5	3.267974	0.001374	MEF2C, PRKCA, TGFBI, SLC39A14, SOX5	142	69	20240	10.32864	0.8353118	0.2253078	0.224621
GOTERM_	GO:0010764~negative regulation of fibroblast migration	3	1.960784	0.002543	ZEB2, RAC1, CYGB	142	11	20240	38.87324	0.9646042	0.370768	0.369638
GOTERM_	GO:2000300~regulation of synaptic vesicle exocytosis	5	3.267974	0.002954	NRN1, SV2C, PRKCE, PRKCA, CACNA1E	142	85	20240	8.384424	0.9793664	0.3875102	0.386329
GOTERM_	GO:0055085~transmembrane transport	10	6.535948	0.004592	SLC25A1, CLCN5, SV2C, KCNH5, SLC7A14, SLC39A5, CACNA1E, SLC39A1, SLC39A14, SLC16A13	142	454	20240	3.139542	0.9976152	0.5059155	0.504373
GOTERM_	GO:0001764~neuron migration	6	3.921569	0.004744	MEF2C, TRIM46, FYN, FAT3, RAC1, CELSR3	142	156	20240	5.482124	0.9980483	0.5059155	0.504373
GOTERM_	GO:0035556~intracellular signal transduction	10	6.535948	0.005066	RAB40B, DUSP1, MAK, PRKCE, FYN, PRKCA, PRKD1, RAPGEF5, DGKK, TNS1	142	461	20240	3.09187	0.9987239	0.5059155	0.504373
GOTERM_	GO:0048813~dendrite morphogenesis	4	2.614379	0.005398	KLF7, ELAVL4, FYN, RAC1	142	51	20240	11.17923	0.9991765	0.5059155	0.504373
GOTERM_	GO:0071577~zinc II ion transmembrane transport	3	1.960784	0.01016	SLC39A5, SLC39A1, SLC39A14	142	22	20240	19.43662	0.9999985	0.8389759	0.836418
GOTERM_	GO:0007626~locomotory behavior	5	3.267974	0.010231	GMFB, ELAVL4, CNTN1, SOBP, CACNA1E	142	121	20240	5.889885	0.9999986	0.8389759	0.836418
GOTERM_	GO:0006829~zinc II ion transport	3	1.960784	0.013017	SLC39A5, SLC39A1, SLC39A14	142	25	20240	17.10423	1	0.8609098	0.858285
GOTERM_	GO:1904520~regulation of myofibroblast cell apoptotic process	2	1.30719	0.013885	SOX2, ZEB2	142	2	20240	142.5352	1	0.8609098	0.858285
GOTERM_	GO:0061178~regulation of insulin secretion involved in cellular response to glucose stimulus	3	1.960784	0.014038	PRKCE, CACNA1E, SIDT2	142	26	20240	16.44637	1	0.8609098	0.858285
GOTERM_	GO:0030030~cell projection organization	6	3.921569	0.014212	UBXN10, RAB34, GPR22, TSC1, FAM149B, TAPT1	142	204	20240	4.192212	1	0.8609098	0.858285
GOTERM_	GO:0090090~negative regulation of canonical Wnt signaling pathway	5	3.267974	0.0148	SOX2, DAB2IP, JADE1, TMEM170B, FOXO1	142	135	20240	5.279082	1	0.8609098	0.858285
GOTERM_	GO:0097150~neuronal stem cell population maintenance	3	1.960784	0.015092	SOX2, HES1, FOXO1	142	27	20240	15.83725	1	0.8609098	0.858285
GOTERM_	GO:0030001~metal ion transport	3	1.960784	0.015092	SLC39A5, SLC39A1, SLC39A14	142	27	20240	15.83725	1	0.8609098	0.858285
GOTERM_	GO:0022409~positive regulation of cell-cell adhesion	3	1.960784	0.01618	SOX2, MAGI1, PDE4D	142	28	20240	15.27163	1	0.8844902	0.881794
GOTERM_	GO:0030517~negative regulation of axon extension	3	1.960784	0.0173	TRIM46, AATK, RTN4	142	29	20240	14.74502	1	0.907891	0.905123

GOTERM_	GO:0050730~regulation of peptidyl-tyrosine phosphorylation	3	1.960784	0.019635	PRKCE, FYN, PRKCA	142	31	20240	13.79373	1	0.973244	0.970277
GOTERM_	GO:0016239~positive regulation of macroautophagy	3	1.960784	0.02085	BNIP3, GNAI3, TSC1	142	32	20240	13.36268	1	0.973244	0.970277
GOTERM_	GO:0007275~multicellular organism development	15	9.803922	0.021099	ROBO2, PAQR8, MEF2C, MYT1L, DAB2IP, EBF1, UNC5D, IKZF1, CELSR3, SOX2, DIP2A, FAT3, FYN, HOXD8, TAPT1	142	1098	20240	1.947202	1	0.973244	0.970277
GOTERM_	GO:0045165~cell fate commitment	4	2.614379	0.021585	SOX2, MEF2C, HES1, SOX5	142	85	20240	6.707539	1	0.973244	0.970277
**GOTERM_**	**GO:0048812~neuron projection morphogenesis**	**4**	**2.614379**	**0.022254**	**MAP2K1, ANKRD27, DAB2IP, RAC1**	**142**	**86**	**20240**	**6.629545**	**1**	**0.973244**	**0.970277**
**GOTERM_**	**GO:0010977~negative regulation of neuron projection development**	**4**	**2.614379**	**0.024328**	**TSC1, HES1, RTN4, PTPRG**	**142**	**89**	**20240**	**6.406077**	**1**	**0.9896694**	**0.986652**
GOTERM_	GO:0006811~ion transport	10	6.535948	0.025461	P2RX6, CLCN5, KCNH5, CACNA2D1, SLC39A5, CACNA1E, SLC39A1, SLC39A14, GRIA3, ATP6V1C1	142	603	20240	2.363768	1	0.9896694	0.986652
GOTERM_	GO:0045766~positive regulation of angiogenesis	5	3.267974	0.02682	ANXA3, PDCD6, PRKCA, PRKD1, RTN4	142	162	20240	4.399235	1	0.9896694	0.986652
GOTERM_	GO:0061512~protein localization to cilium	3	1.960784	0.027372	TUB, ARF4, FAM149B	142	37	20240	11.55691	1	0.9896694	0.986652
**GOTERM_**	**GO:0060164~regulation of timing of neuron differentiation**	**2**	**1.30719**	**0.027578**	**HES1, SOX5**	**142**	**4**	**20240**	**71.26761**	**1**	**0.9896694**	**0.986652**
GOTERM_	GO:0007015~actin filament organization	5	3.267974	0.02842	TPM2, AVIL, ELMO1, TAGLN3, RAC1	142	165	20240	4.319249	1	0.9896694	0.986652
**GOTERM_**	**GO:0030900~forebrain development**	**4**	**2.614379**	**0.028779**	**SOX2, FYN, RAC1, IKZF1**	**142**	**95**	**20240**	**6.001483**	**1**	**0.9896694**	**0.986652**
GOTERM_	GO:0045944~positive regulation of transcription from RNA polymerase II promoter	16	10.45752	0.029042	ARF4, MEF2C, MYT1L, DAB2IP, EBF1, ESRRG, IKZF1, FOXO1, CX3CL1, SOX2, TOX3, KLF7, ZEB2, HES1, PRKD1, HOXD8	142	1257	20240	1.814291	1	0.9896694	0.986652
GOTERM_	GO:0042593~glucose homeostasis	5	3.267974	0.029419	KLF7, MAP2K1, CACNA1E, FOXO1, SIDT2	142	167	20240	4.267521	1	0.9896694	0.986652
**GOTERM_**	**GO:0048870~cell motility**	**3**	**1.960784**	**0.030182**	**MAP2K1, ELMO1, RAC1**	**142**	**39**	**20240**	**10.96425**	**1**	**0.9899633**	**0.986945**
**GOTERM_**	**GO:0050772~positive regulation of axonogenesis**	**3**	**1.960784**	**0.033101**	**ROBO2, ZEB2, MAP2K1**	**142**	**41**	**20240**	**10.42941**	**1**	**1**	**0.997712**
GOTERM_	GO:0045893~positive regulation of transcription, DNA-templated	11	7.189542	0.03333	SOX2, TOX3, KLF7, MAP2K1, MEF2C, MTA1, EBF1, HES1, ESRRG, IKZF1, FOXO1	142	736	20240	2.130282	1	1	0.997712
GOTERM_	GO:0035437~maintenance of protein localization in endoplasmic reticulum	2	1.30719	0.034353	KDELR2, TAPT1	142	5	20240	57.01408	1	1	0.997712
GOTERM_	GO:0016236~macroautophagy	3	1.960784	0.037676	GABARAPL2, MAP1LC3B, YOD1	142	44	20240	9.71831	1	1	0.997712
GOTERM_	GO:0006890~retrograde vesicle-mediated transport, Golgi to ER	3	1.960784	0.037676	ARF4, GOLPH3L, KDELR2	142	44	20240	9.71831	1	1	0.997712
GOTERM_	GO:0036324~vascular endothelial growth factor receptor-2 signaling pathway	2	1.30719	0.041082	PDCD6, DAB2IP	142	6	20240	47.51174	1	1	0.997712
GOTERM_	GO:1901998~toxin transport	3	1.960784	0.044127	RAB40B, BNIP3, PTPRG	142	48	20240	8.908451	1	1	0.997712
GOTERM_	GO:0071902~positive regulation of protein serine/threonine kinase activity	3	1.960784	0.044127	DAZAP2, MAP2K1, DAB2IP	142	48	20240	8.908451	1	1	0.997712
**GOTERM_**	**GO:0007411~axon guidance**	**5**	**3.267974**	**0.044784**	**ROBO2, KLF7, CNTN1, UNC5D, RAC1**	**142**	**191**	**20240**	**3.731288**	**1**	**1**	**0.997712**
GOTERM_	GO:0042177~negative regulation of protein catabolic process	3	1.960784	0.047495	DAB2IP, FYN, AZIN1	142	50	20240	8.552113	1	1	0.997712
GOTERM_	GO:0045933~positive regulation of muscle contraction	2	1.30719	0.047764	MAP2K1, CACNA2D1	142	7	20240	40.72435	1	1	0.997712
**GOTERM_**	**GO:0051402~neuron apoptotic process**	**4**	**2.614379**	**0.048499**	**BNIP3, AATK, SNCB, CTSB**	**142**	**117**	**20240**	**4.872999**	**1**	**1**	**0.997712**

Table S24. KEGG pathway analysis of miR-183C targets in the cornea of both SNS-CKO and miR-183C KO strains.

Category	Term	Count	%	PValue	Genes	List Total	Pop Hits	Pop Total	Fold Enrichment	Bonferroni	Benjamini	FDR
KEGG_PAT	mmu04071:Sphingolipid signaling pathway	7	4.575163	1.59E-04	MAP2K1, PRKCE, GNAI3, FYN, PRKCA, RAC1, SGMS2	61	126	9212	8.3898	0.030688	0.031166	0.028781
**KEGG_PAT**	**mmu04360:Axon guidance**	**7**	**4.575163**	**0.0011**	**ROBO2, GNAI3, FYN, PRKCA, UNC5D, RAC1, SSH2**	**61**	**181**	**9212**	**5.840413**	**0.194023**	**0.101113**	**0.093374**
KEGG_PAT	mmu04371:Apelin signaling pathway	6	3.921569	0.001917	MAP1LC3B, MAP2K1, MEF2C, GNG5, PRKCE, GNAI3	61	137	9212	6.613857	0.313435	0.101113	0.093374
KEGG_PAT	mmu04140:Autophagy - animal	6	3.921569	0.002243	GABARAPL2, MAP1LC3B, MAP2K1, BNIP3, TSC1, CTSB	61	142	9212	6.380974	0.356031	0.101113	0.093374
KEGG_PAT	mmu04015:Rap1 signaling pathway	7	4.575163	0.002579	MAGI1, MAP2K1, GNAI3, PRKCA, PRKD1, RAC1, RAPGEF5	61	214	9212	4.939789	0.397228	0.101113	0.093374
KEGG_PAT	mmu05231:Choline metabolism in cancer	5	3.267974	0.003724	MAP2K1, TSC1, PRKCA, RAC1, DGKK	61	98	9212	7.704918	0.518682	0.121644	0.112335
KEGG_PAT	mmu04928:Parathyroid hormone synthesis, secretion and action	5	3.267974	0.005265	MAP2K1, MEF2C, PDE4D, GNAI3, PRKCA	61	108	9212	6.9915	0.644641	0.134959	0.12463
**KEGG_PAT**	**mmu04725:Cholinergic synapse**	**5**	**3.267974**	**0.005985**	**MAP2K1, GNG5, GNAI3, FYN, PRKCA**	**61**	**112**	**9212**	**6.741803**	**0.691686**	**0.134959**	**0.12463**
KEGG_PAT	mmu05163:Human cytomegalovirus infection	7	4.575163	0.006197	MAP2K1, GNG5, GNAI3, TSC1, PRKCA, RAC1, CX3CL1	61	256	9212	4.129355	0.704299	0.134959	0.12463
**KEGG_PAT**	**mmu04730:Long-term depression**	**4**	**2.614379**	**0.006908**	**MAP2K1, GNAI3, PRKCA, GRIA3**	**61**	**60**	**9212**	**10.06776**	**0.743001**	**0.135399**	**0.125036**
**KEGG_PAT**	**mmu04062:Chemokine signaling pathway**	**6**	**3.921569**	**0.008069**	**MAP2K1, GNG5, ELMO1, GNAI3, RAC1, CX3CL1**	**61**	**192**	**9212**	**4.719262**	**0.795637**	**0.143768**	**0.132765**
KEGG_PAT	mmu04664:Fc epsilon RI signaling pathway	4	2.614379	0.008987	MAP2K1, FYN, PRKCA, RAC1	61	66	9212	9.152509	0.829564	0.146786	0.135553
**KEGG_PAT**	**mmu04726:Serotonergic synapse**	**5**	**3.267974**	**0.010306**	**MAP2K1, GNG5, DUSP1, GNAI3, PRKCA**	**61**	**131**	**9212**	**5.763984**	**0.868737**	**0.15539**	**0.143498**
KEGG_PAT	mmu00601:Glycosphingolipid biosynthesis - lacto and neolacto series	3	1.960784	0.012263	FUT9, B3GALT1, ST3GAL6	61	26	9212	17.42497	0.910939	0.171684	0.158545
KEGG_PAT	mmu04010:MAPK signaling pathway	7	4.575163	0.013393	MAP2K1, MEF2C, DUSP1, CACNA2D1, PRKCA, RAC1, CACNA1E	61	302	9212	3.50038	0.928835	0.175002	0.161609
KEGG_PAT	mmu04072:Phospholipase D signaling pathway	5	3.267974	0.015914	MAP2K1, TSC1, FYN, PRKCA, DGKK	61	149	9212	5.067664	0.956901	0.194947	0.180028
**KEGG_PAT**	**mmu04921:Oxytocin signaling pathway**	**5**	**3.267974**	**0.017377**	**MAP2K1, MEF2C, CACNA2D1, GNAI3, PRKCA**	**61**	**153**	**9212**	**4.935176**	**0.967798**	**0.200341**	**0.185009**
**KEGG_PAT**	**mmu04727:GABAergic synapse**	**4**	**2.614379**	**0.020078**	**GABARAPL2, GNG5, GNAI3, PRKCA**	**61**	**89**	**9212**	**6.787254**	**0.981226**	**0.218625**	**0.201894**
KEGG_PAT	mmu05032:Morphine addiction	4	2.614379	0.021283	GNG5, PDE4D, GNAI3, PRKCA	61	91	9212	6.638083	0.985251	0.219556	0.202753
**KEGG_PAT**	**mmu04666:Fc gamma R-mediated phagocytosis**	**4**	**2.614379**	**0.022528**	**MAP2K1, PRKCE, PRKCA, RAC1**	**61**	**93**	**9212**	**6.495329**	**0.988507**	**0.220777**	**0.20388**
**KEGG_PAT**	**mmu04713:Circadian entrainment**	**4**	**2.614379**	**0.025811**	**GNG5, GNAI3, PRKCA, GRIA3**	**61**	**98**	**9212**	**6.163934**	**0.994056**	**0.240904**	**0.222468**
KEGG_PAT	mmu04933:AGE-RAGE signaling pathway in diabetic complications	4	2.614379	0.027898	PRKCE, PRKCA, RAC1, FOXO1	61	101	9212	5.980847	0.996096	0.248548	0.229526
KEGG_PAT	mmu04724:Glutamatergic synapse	4	2.614379	0.037951	GNG5, GNAI3, PRKCA, GRIA3	61	114	9212	5.298821	0.999491	0.323412	0.298661
KEGG_PAT	mmu04650:Natural killer cell mediated cytotoxicity	4	2.614379	0.039642	MAP2K1, FYN, PRKCA, RAC1	61	116	9212	5.207462	0.99964	0.323745	0.298969
**KEGG_PAT**	**mmu04670:Leukocyte transendothelial migration**	**4**	**2.614379**	**0.041371**	**GNAI3, MSN, PRKCA, RAC1**	**61**	**118**	**9212**	**5.1192**	**0.999747**	**0.324349**	**0.299527**

Table S25. miR-183C target genes identified in the CRMCs in miR-183C conventional KO mice

	logFC	logCPM	PValue	WT	KO
2310022B05Rik	7.231048112	3.736955525	1.5823E-18	1	242
AI314180	6.573937181	2.372838111	0.00143701	0	14
Abca1	10.97607693	4.028589272	1.68103E-21	0	299
Abl1	13.03577642	6.337087454	8.9812E-36	0	1247
Ablim1	12.2435095	7.384718763	4.19122E-30	0	720
Actr2	5.383979384	6.719408347	2.55726E-24	28	1717
Ahr	11.90908098	5.625730749	9.03997E-28	0	571
Amotl2	10.92202762	3.635958559	3.71851E-21	0	288
Anxa3	13.19962853	6.229897996	5.81888E-37	0	1397
Aph1a	11.87068524	4.307042048	1.6338E-27	0	556
Appl1	9.560625516	5.772024489	4.12739E-13	0	112
Arcn1	11.16137982	4.013834788	1.03204E-22	0	340
Arhgdia	11.05787647	8.38729483	2.00064E-44	1	3436
Arhgef12	8.70608081	5.419677652	2.53585E-28	1	673
Arhgef18	12.61422634	7.051975209	9.61495E-33	0	931
Arhgef3	13.13306242	7.040913601	1.76808E-36	0	1334
Arid4a	8.559579656	6.61415195	2.57093E-27	1	608
Arl10	12.16318443	5.140615095	1.52377E-29	0	681
Arpc1b	1.588441032	11.18900024	0.000163053	1979	8709
Arrdc3	11.46267656	5.977067833	1.03963E-24	0	419
Asap1	10.74655227	6.451868412	4.73969E-20	0	255
Ash2l	12.22536592	4.994081091	5.52736E-30	0	711
Atp13a3	12.27717036	4.66607867	2.43167E-30	0	737
Atxn7	12.15255521	4.334259075	1.79916E-29	0	676
Azin1	4.905470962	6.511323752	1.85188E-20	23	1013
Basp1	5.070947327	7.040008099	1.45621E-22	33	1628
Baz1b	12.1842108	6.795630623	1.09657E-29	0	691
Bicd2	13.15772175	5.172511581	1.17124E-36	0	1357
Braf	8.777019161	5.828788074	2.40073E-09	0	65
Bre	12.92272147	5.698353812	5.93757E-35	0	1153
Btg1	9.79737522	6.941988362	2.04048E-14	0	132
Bzw1	9.03950534	6.053790526	1.5162E-10	0	78
C2cd2	13.21911571	5.178426976	4.17392E-37	0	1416
Cacna1b	9.338551471	1.225034255	5.39633E-12	0	96
Cald1	12.09598436	7.219200341	4.57838E-29	0	650
Camk2n1	12.49476024	7.011712067	6.79243E-32	0	857
Camkk2	10.8020157	6.465726399	2.1694E-20	0	265
Cand1	12.88212054	5.7837639	1.15188E-34	0	1121
Capns1	13.22115177	8.956843947	4.08873E-37	0	1418
Casd1	12.20904448	5.521423344	7.31206E-30	0	703
Ccdc50	11.31034778	5.95232094	1.03633E-23	0	377
Ccng2	12.33847724	5.455729531	8.78956E-31	0	769
Cd2ap	6.468186381	6.039041642	0.002434933	0	13
Cd47	11.26764398	7.674874057	1.97681E-23	0	366
Cdc37	14.74328756	8.030815771	2.48405E-48	0	4073
Cdc42bpg	11.96360842	3.97433951	3.81663E-28	0	593
Cdc42se1	13.15239676	7.532341836	1.27661E-36	0	1352
Cdsn	8.798995637	0.754843559	1.8437E-09	0	66
Celf1	12.98994095	7.432178092	1.916E-35	0	1208
Celf2	13.08132167	8.76881608	4.20845E-36	0	1287
Cers5	13.93260339	6.984314278	2.48826E-42	0	2322
Chd2	13.38630466	6.131117756	2.52081E-38	0	1590

Chmp1a	12.21722828	7.493118362	6.2292E-30	0	707
Cited2	6.202980071	5.962195396	1.06754E-25	9	981
Cnot6	12.6372817	6.509792385	6.65973E-33	0	946
Coro1c	13.50720493	7.802664761	3.29356E-39	0	1729
Cpeb4	10.01014639	4.218213945	1.39302E-15	0	153
Creb3l1	11.28721163	5.248782832	1.48053E-23	0	371
Creb3l2	11.0047266	5.661305968	1.09307E-21	0	305
Crebrf	11.9853344	5.549943486	2.62603E-28	0	602
Crk	12.219267	6.088721576	6.2292E-30	0	708
Csad	12.06684474	5.517859594	7.08314E-29	0	637
Csnk1d	13.11565607	7.517966579	2.35286E-36	0	1318
Csnk2a1	11.29496498	5.830137297	1.37802E-23	0	373
Ctdsp1	12.46242095	6.15390255	1.16461E-31	0	838
Ctnnd1	12.42404675	5.662602949	2.16664E-31	0	816
Cttn	6.724236137	5.044156079	2.21057E-29	9	1408
Cux1	13.57005967	7.00005907	1.15432E-39	0	1806
Cxadr	12.53787111	4.321927056	3.40538E-32	0	883
Cyb5b	14.00056034	6.980702988	7.90847E-43	0	2434
D17Wsu92e	12.79458922	7.216015769	4.97253E-34	0	1055
Dagla	13.10135712	5.180461169	3.00413E-36	0	1305
Dap	1.002746871	5.618689546	0.019281544	253	742
Dazap2	13.62414801	8.895253196	4.63643E-40	0	1875
Dcaf12	10.76341633	7.281188364	3.88953E-20	0	258
Ddah1	12.38825262	4.823042842	3.95106E-31	0	796
Ddx6	6.468186381	7.531516453	0.002434933	0	13
Denr	13.2533438	7.002236092	2.35804E-37	0	1450
Dgcr2	12.20699126	5.198901298	7.61223E-30	0	702
Dhcr24	11.51340369	4.341726136	4.55073E-25	0	434
Dixdc1	12.1114345	3.986749434	3.53648E-29	0	657
Dusp1	0.873979545	8.30121228	0.037944936	519	1392
Dyrk2	13.42832835	6.229237038	1.24068E-38	0	1637
Edem1	13.18092097	6.855632816	7.97776E-37	0	1379
Egr1	0.86459479	9.833102797	0.036797413	3821	10181
Ehd1	12.75859433	7.239758055	8.89709E-34	0	1029
Eif3j1	12.20904448	5.634452955	7.31206E-30	0	703
Eif4a2	12.84695172	6.135344993	2.04661E-34	0	1094
Eif4e2	10.13634555	6.542804072	2.45694E-16	0	167
Ell	5.777895307	4.590456779	0.022676824	0	8
Emc7	13.13630285	6.291953284	1.65602E-36	0	1337
Eny2	9.229497657	5.153704014	1.94396E-11	0	89
Ep300	12.15041993	6.123786088	1.87579E-29	0	675
Epas1	12.70867644	5.801706612	2.03298E-33	0	994
Ephb1	7.010519164	4.303697398	0.000193974	0	19
Erlin1	12.80819774	4.820786773	3.89103E-34	0	1065
Exoc4	7.712450153	4.029604379	2.40377E-06	0	31
Fam134b	9.683851328	5.118408069	9.584E-14	0	122
Fam171a1	10.019536	1.853564145	1.19985E-15	0	154
Fam49b	11.76858071	7.833439629	8.51021E-27	0	518
Fbxw11	3.375530668	5.179620715	3.45558E-12	46	700
Flrt3	12.78910966	4.570684643	5.39902E-34	0	1051
Fnbp1	11.4899528	8.41068537	6.64061E-25	0	427
Fnta	11.55273908	5.907587186	2.45432E-25	0	446
Foxp1	13.89100518	8.715216753	5.06729E-42	0	2256
Foxq1	12.93020856	4.890350251	5.23753E-35	0	1159

Fyn	3.74158897	5.14017635	2.72474E-10	9	178
Gap43	7.010519164	4.634385849	0.000193974	0	19
Gbp7	11.99963873	5.522373081	2.08464E-28	0	608
Gcnt1	12.65696927	6.299826233	4.77845E-33	0	959
Gmfb	11.65267383	6.699625582	5.27521E-26	0	478
Gnai3	7.403759936	5.223953566	1.95811E-05	0	25
Gorasp1	13.34302006	5.122682432	5.22887E-38	0	1543
Grb2	5.07481805	7.963590904	1.32599E-19	13	646
Grhl2	11.22769401	3.812753858	3.83594E-23	0	356
Hdac7	12.33472094	7.514909447	9.46441E-31	0	767
Hecw2	6.573937181	3.245760123	0.00143701	0	14
Hilpda	6.672462944	3.810977769	0.00085757	0	15
Hn1	11.72045325	6.228820411	1.81566E-26	0	501
Hnrnpa1	5.832448629	5.90236687	1.23268E-21	7	592
Hsph1	14.53605444	6.857325164	8.56513E-47	0	3528
Idh2	12.35340537	6.279294166	7.04944E-31	0	777
Ids	13.85539963	7.296981552	9.20874E-42	0	2201
Igsf3	12.24551145	4.555119048	4.02977E-30	0	721
Ikzf1	8.279571054	8.006039381	2.50562E-07	0	46
Insig1	5.777895307	4.469976659	0.022676824	0	8
Insig2	11.29882608	4.896497484	1.28287E-23	0	374
Irf2bpl	12.99946256	6.875642823	1.65933E-35	0	1216
Irf6	13.58040647	5.931192292	9.65478E-40	0	1819
Itgb1	12.70285998	7.229850592	2.21792E-33	0	990
Itsn2	13.10246209	7.066026385	2.93784E-36	0	1306
Jam2	12.97794989	4.81849104	2.32404E-35	0	1198
Jmjd1c	12.28302983	6.002666944	2.16722E-30	0	740
Kat7	11.86808882	5.466648402	1.71749E-27	0	555
Kctd15	11.04206207	2.872043148	6.05682E-22	0	313
Kdm5a	11.85765617	6.24976358	1.99639E-27	0	551
Kif13a	11.52992396	5.953380845	3.54834E-25	0	439
Klhl7	8.613029204	4.697007045	1.24346E-08	0	58
Kmt2a	8.004913702	7.05113311	2.40483E-07	0	38
Kpnb1	10.89677399	6.736246364	5.33577E-21	0	283
Kras	9.912727715	5.997696034	4.7536E-15	0	143
Ktn1	13.41860217	6.468552971	1.48517E-38	0	1626
L1cam	9.695614235	5.687713749	8.0319E-14	0	123
Lamc1	12.23949727	6.043757902	4.3594E-30	0	718
Larp4	9.623553668	4.038083351	1.96785E-13	0	117
Lpp	8.637628806	5.118567783	9.38231E-09	0	59
Lrrc8c	12.40088183	5.45156528	3.19083E-31	0	803
Lrrk1	9.323468035	4.57274873	6.65137E-12	0	95
Lsm14a	12.90256383	6.298823246	8.25191E-35	0	1137
Ltn1	11.9972645	5.593941681	2.18278E-28	0	607
Magi1	10.22017439	2.044123359	8.23326E-17	0	177
Mal2	14.60433637	6.728297246	2.67384E-47	0	3699
Map1lc3b	11.81784773	8.454956168	3.8732E-27	0	536
Map2k1	11.85503617	6.629708021	2.09951E-27	0	550
Map2k3	2.360892363	6.977097897	2.77729E-07	82	617
Map4k5	11.79888774	5.150470484	5.29089E-27	0	529
Mapre1	12.88725858	8.48556611	1.06613E-34	0	1125
Marcks	7.460015749	8.650910541	1.26863E-05	0	26
Mark2	12.3004668	6.644915586	1.66004E-30	0	749
Mast4	6.573937181	5.648314625	0.00143701	0	14

Mbnl1	3.503634621	8.306539018	4.10083E-13	51	848
Mdp1	12.76698091	6.189826562	7.73665E-34	0	1035
Med1	6.573937181	4.502672988	0.00143701	0	14
Meis2	6.094555017	1.255360712	0.007243414	0	10
Mfsd5	14.04376592	6.873615686	3.75876E-43	0	2508
Mgat2	13.27310505	5.910642619	1.71137E-37	0	1470
Morc3	10.82361749	4.763764852	1.62835E-20	0	269
Mospd2	7.616709141	3.436800498	3.60565E-06	0	29
Mpp1	11.53647947	5.773611227	3.13616E-25	0	441
Mta1	10.01014639	6.270729068	1.39302E-15	0	153
Mtch2	12.23547385	6.23198788	4.71717E-30	0	716
Mtdh	13.03808815	7.839708296	8.57226E-36	0	1249
Mtmr12	13.15239676	6.901141325	1.27661E-36	0	1352
Muc15	10.11897901	1.94776503	3.25398E-16	0	165
Myadm	12.40626069	6.667184415	2.97265E-31	0	806
Myo1c	12.26537953	7.266876157	2.94955E-30	0	731
Naa50	12.06457866	6.519694982	7.4022E-29	0	636
Nampt	11.64055406	5.580078161	6.26929E-26	0	474
Nap1l1	13.40702241	7.976205397	1.78015E-38	0	1613
Ndrg1	6.851370265	5.11161698	0.000315208	0	17
Nfic	12.27325078	6.629548586	2.5271E-30	0	735
Nop9	11.97330483	4.935098788	3.16363E-28	0	597
Npc2	14.11118995	8.962349112	1.19769E-43	0	2628
Npm1	9.753031766	8.346732033	4.01056E-14	0	128
Nr4a3	10.5788509	5.878465851	5.79007E-19	0	227
Nsd1	12.2574651	7.253611915	3.31421E-30	0	727
Nudt4	13.61952435	7.103936915	5.01603E-40	0	1869
Nus1	11.17824632	5.511616319	8.18434E-23	0	344
Ogt	7.5787818	6.052881362	9.6529E-21	1	308
Pafah1b1	11.93907839	7.077485528	5.57861E-28	0	583
Paip2	12.91267786	6.385082877	6.90796E-35	0	1145
Parm1	12.02083275	4.846992786	1.51423E-28	0	617
Pax6	12.27128699	6.649848343	2.62644E-30	0	734
Pcnx	8.182597729	4.186665988	6.64018E-07	0	43
Pfn1	8.904120519	5.698565414	6.58751E-10	0	71
Phf20l1	6.933138139	5.381016816	0.000315208	0	18
Phf21a	13.91006227	7.491721562	3.65835E-42	0	2286
Phip	11.11395372	6.194267735	2.09679E-22	0	329
Pik3ap1	6.573937181	4.539831758	0.00143701	0	14
Pkp4	12.88854024	5.373955087	1.03904E-34	0	1126
Plekha3	7.403759936	4.577685328	1.95811E-05	0	25
Plet1	15.50125939	8.31311048	5.54898E-54	0	6888
Pmepa1	10.97607693	6.969468189	1.68103E-21	0	299
Pnisr	11.59103038	7.273859694	1.34353E-25	0	458
Pou2f2	6.230137985	7.782813683	0.007243414	0	11
Ppm1l	5.589001617	4.487756612	0.040964585	0	7
Ppp1r12c	13.2533438	6.745092238	2.35804E-37	0	1450
Ppp1r14b	14.29010483	6.394212891	5.6872E-45	0	2975
Ppp2ca	12.56056188	6.934255246	2.31453E-32	0	897
Ppp2cb	6.573937181	1.779889373	0.00143701	0	14
Ppp2r5c	9.534665043	6.171206367	6.02775E-13	0	110
Ppp3ca	9.85095401	7.625329808	1.05675E-14	0	137
Prdm1	11.29109351	4.664396657	1.37802E-23	0	372
Prkacb	8.661815978	6.401437227	7.10124E-09	0	60

Prkar1a	13.25731776	7.754130689	2.2198E-37	0	1454
Prkcz	9.623553668	1.867352691	1.96785E-13	0	117
Prrx1	10.12768841	6.531554734	2.8264E-16	0	166
Ptdss1	13.13089807	6.654472553	1.84715E-36	0	1332
Ptpn1	12.23345792	8.78812142	4.90738E-30	0	715
Pura	13.43973902	9.17701804	1.03777E-38	0	1650
Pygo2	14.03915694	6.986782705	4.08357E-43	0	2500
Qk	10.24440178	6.518345018	6.31015E-17	0	180
Rab21	12.10041559	5.783275188	4.19955E-29	0	652
Rab2a	13.29453428	7.455819986	1.17576E-37	0	1492
Rab35	12.14828149	5.262244456	1.95581E-29	0	674
Rabgap1	13.53365972	6.168001241	2.12534E-39	0	1761
Rac1	6.372885246	8.546555054	5.76764E-29	14	1710
Rad23b	13.07907823	7.315573005	4.3048E-36	0	1285
Rala	13.19134399	7.092141099	6.59856E-37	0	1389
Rap2b	11.1313805	5.121078685	1.65182E-22	0	333
Rap2c	11.77967525	6.012241765	6.88204E-27	0	522
Rbms1	13.55076071	8.291566101	1.60258E-39	0	1782
Rbmx	6.468186381	4.546033125	0.002434933	0	13
Rcn2	8.078633437	2.336425103	1.29886E-06	0	40
Rcor1	8.114127181	5.947225854	9.26662E-07	0	41
Reps2	12.20904448	4.119141231	7.31206E-30	0	703
Rere	9.79737522	7.825657538	2.04048E-14	0	132
Rfxap	5.944895804	4.186174973	0.012730848	0	9
Rhob	14.00056034	7.397227733	7.90847E-43	0	2434
Rimkla	7.802231021	0.398672071	1.08934E-06	0	33
Rnf130	6.933138139	4.321871218	0.000315208	0	18
Rnf208	8.310499164	1.517102093	1.82531E-07	0	47
Rora	12.91393714	6.574981237	6.73543E-35	0	1146
Rps12	1.203394176	11.29236823	0.003911722	2558	8620
Rsu1	12.25349154	6.236063587	3.58311E-30	0	725
Rtn4	13.99343047	8.077876388	8.82665E-43	0	2422
Rufy1	8.732035672	5.905687107	4.10498E-09	0	63
Sar1a	5.944895804	6.476902366	0.012730848	0	9
Sbds	13.27015806	6.129651953	1.78093E-37	0	1467
Scaf11	11.82857117	5.730833112	3.3187E-27	0	540
Scyl2	7.802231021	5.56667275	1.08934E-06	0	33
Sdc2	12.17163179	5.591360995	1.34622E-29	0	685
Sec11a	12.57017829	6.900421587	1.97399E-32	0	903
Secisbp2l	12.60333976	5.450829061	1.15766E-32	0	924
Serp1	14.69905004	9.190370523	5.26991E-48	0	3950
Sgk3	12.94506718	5.97401295	4.08297E-35	0	1171
Sgms1	13.12655962	5.686848428	1.97271E-36	0	1328
Sidt2	8.114127181	6.688521104	9.26662E-07	0	41
Skil	9.730338228	5.252126069	4.76242E-14	0	126
Slc10a3	12.67787504	4.761338071	3.34321E-33	0	973
Slc22a23	12.89110014	5.580886289	9.86989E-35	0	1128
Slc25a1	10.72375577	6.733482424	7.0716E-20	0	251
Slc25a51	11.21549006	5.897342247	4.45554E-23	0	353
Slc35d1	11.91160472	4.53623369	8.61033E-28	0	572
Slco3a1	7.460015749	4.905567905	1.26863E-05	0	26
Slmo2	11.69723723	5.061620792	2.53038E-26	0	493
Snx16	8.661815978	3.802947791	7.10124E-09	0	60
Socs6	8.215651735	4.400209755	4.77852E-07	0	44

Socs7	8.114127181	5.374363317	9.26662E-07	0	41
Soga1	11.07845566	3.973092462	3.69137E-22	0	321
Sowahc	11.48318194	4.594478648	7.54177E-25	0	425
Spcs2	9.338551471	6.45703588	5.39633E-12	0	96
Spen	12.78223082	6.589495331	6.02767E-34	0	1046
Sppl2a	9.19676297	6.232815798	3.02494E-11	0	87
Srsf2	13.58119931	7.252422507	9.49986E-40	0	1820
St8sia3	8.732035672	0.698669956	4.10498E-09	0	63
Strbp	9.671991724	8.089517281	9.584E-14	0	121
Sypl	13.17042209	6.296544593	9.45547E-37	0	1369
Taok1	11.71757159	6.334419154	1.91834E-26	0	500
Tapt1	13.10907416	6.145876462	2.62848E-36	0	1312
Tbl1x	7.616709141	5.097311046	3.60565E-06	0	29
Tcf12	6.573937181	6.033958982	0.00143701	0	14
Tcf4	6.666173212	8.52693618	1.0259E-25	5	758
Tes	7.460015749	4.394898581	1.26863E-05	0	26
Tgfbr1	11.99963873	6.099834531	2.08464E-28	0	608
Tle4	13.14598073	6.406576085	1.42233E-36	0	1346
Tmbim6	6.838268483	8.648322304	3.78581E-34	23	3868
Tmcc3	13.24235862	5.885658446	2.82909E-37	0	1439
Tmed7	11.67661215	4.871798646	3.54224E-26	0	486
Tmem126a	11.87327699	4.965647624	1.55434E-27	0	557
Tmem203	12.74733585	5.061916404	1.08342E-33	0	1021
Tmem57	12.2294176	5.739201958	5.10558E-30	0	713
Tmpo	11.76300128	6.980801372	8.97675E-27	0	516
Tmx1	10.47347312	6.819072421	2.51342E-18	0	211
Tnk2	11.37395438	5.858732494	3.91851E-24	0	394
Tnrc6a	14.13622274	8.252118639	7.82653E-44	0	2674
Tnrc6b	12.19874901	7.06552533	8.59205E-30	0	698
Tpd52	1.495104539	7.886730309	0.000626543	158	652
Tram1	3.947182838	7.450669638	8.17449E-14	19	431
Trim2	14.00883422	6.130441811	6.83616E-43	0	2448
Trp53inp2	13.05530911	5.252760689	6.49304E-36	0	1264
Tsc1	12.26537953	4.961532801	2.94955E-30	0	731
Tsc22d3	11.58471866	7.525351939	1.51384E-25	0	456
Tsnax	14.26219645	6.711028119	9.13869E-45	0	2918
Tspan14	10.47347312	5.557725763	2.51342E-18	0	211
Ttyh3	1.497702365	7.123496373	0.000673244	134	554
Ube2k	12.5653781	6.478187806	2.17151E-32	0	900
Ube2r2	13.63945405	8.46437149	3.55452E-40	0	1895
Ubn2	5.101365421	6.926851224	8.8188E-20	13	658
Uck2	12.14828149	5.567819856	1.95581E-29	0	674
Usp2	8.661815978	2.072739783	7.10124E-09	0	60
Usp36	8.383160344	6.269136517	4.26124E-26	1	538
Usp47	12.25944778	6.148040192	3.18771E-30	0	728
Vat1	11.72619934	4.65406378	1.62707E-26	0	503
Vmac	13.01009979	5.576631636	1.37152E-35	0	1225
Wasf2	13.0183193	7.426882244	1.19005E-35	0	1232
Wdr26	8.712496139	8.337551213	2.23808E-28	1	676
Wdr5	7.403759936	5.325269881	1.95811E-05	0	25
Wdr76	2.421287185	2.533014666	0.002142368	4	32
Whsc1l1	9.89242896	6.098196097	6.52344E-15	0	141
Wisp1	13.26819005	5.267367076	1.85344E-37	0	1465
Wnt4	8.39949382	4.582075431	9.79951E-08	0	50

Ywhae	14.88101169	8.871541142	2.33579E-49	0	4481
Ywhag	14.15604729	7.664205495	5.6219E-44	0	2711
Zcchc14	12.9189633	4.999641543	6.24422E-35	0	1150
Zdhhc20	11.27941639	5.329102882	1.71005E-23	0	369
Zeb2	12.30431323	5.925662123	1.53909E-30	0	751
Zfp292	6.764688096	5.405020324	0.000517265	0	16
Zfp322a	12.08484635	4.949967352	5.44654E-29	0	645

Table S26. miR-183C target genes identified in the CRMCs in miR-183C MS-CKO mice

	logFC	logCPM	PValue	WT	MS-CKO
0610007P14Rik	9.784783276	5.716488848	2.41191E-14	0	125
1810011O10Rik	12.69409719	3.795705157	2.1544E-33	0	940
2310022B05Rik	11.85941	3.736955525	1.6338E-27	0	527
2810403A07Rik	4.442439223	5.622321719	4.88917E-17	21	602
4931406P16Rik	11.51482316	3.728926072	3.77519E-25	0	415
Abca1	10.14056927	4.028589272	2.13744E-16	0	160
Ablim1	13.84605727	7.384718763	8.84527E-42	0	2089
Actr2	10.84051733	6.719408347	1.11639E-20	0	260
Acvr1	13.23325412	4.553462932	2.71657E-37	0	1366
Adam10	12.52841318	5.686544826	3.29679E-32	0	838
Adcy6	12.43233341	3.618978778	1.58543E-31	0	784
Adra2c	11.64753502	2.845008756	4.70479E-26	0	455
Aebp2	13.05713034	5.794828702	5.16324E-36	0	1209
Aff4	2.029786224	5.819154059	2.51278E-06	477	2556
Agap3	4.348094439	4.336392674	6.66251E-16	17	457
Ago3	8.510313517	4.67165944	1.81013E-26	1	530
Akap7	4.8231367	2.944239656	7.67243E-16	8	301
Aktip	11.07069886	3.942250942	3.40217E-22	0	305
Amd1	13.35956433	4.905813384	3.26759E-38	0	1491
Ammecr1l	6.916815087	5.048044162	0.000315208	0	17
Ank3	13.17507052	4.250226348	7.17808E-37	0	1312
Ankrd13c	11.65701313	3.137551241	4.19824E-26	0	458
Appl1	11.38775665	5.772024489	2.79363E-24	0	380
Arcn1	6.169895158	4.013834788	2.2085E-16	2	199
Arel1	9.560741089	4.441970521	4.12739E-13	0	107
Arf4	5.913009883	7.537454534	4.56491E-30	82	6491
Arhgap17	12.88364263	6.115195632	9.13944E-35	0	1072
Arhgdia	14.75919579	8.38729483	1.52678E-48	0	3934
Arhgef12	13.17836525	5.419677652	6.73872E-37	0	1315
Arhgef18	11.79790734	7.051975209	4.52468E-27	0	505
Arhgef3	8.949566504	7.040913601	2.69457E-11	0	70
Arid4a	13.64957364	6.61415195	2.45426E-40	0	1823
Arid4b	1.520701673	6.314558789	0.000403572	273	1028
Arid5b	12.77176514	5.830361347	5.86369E-34	0	992
Armc1	12.17961732	6.023548844	9.70371E-30	0	658
Arpp19	12.37414036	6.89915064	4.09505E-31	0	753
Atad2b	13.50398724	6.218799229	2.87591E-39	0	1648
Atf2	9.895693432	4.357427697	5.56586E-15	0	135
Atf7ip	12.36453059	6.370556462	4.72787E-31	0	748
Atg7	10.08547751	4.404813397	4.32335E-16	0	154
Atl2	11.88920527	4.082610459	1.04678E-27	0	538
Atp13a3	9.664631256	4.66607867	9.584E-14	0	115
Atp2b4	5.718732351	4.184690819	7.44324E-21	7	491
Atrn	12.91821126	4.246872642	5.23753E-35	0	1098
Atxn1	7.286067663	4.377692178	3.04594E-05	0	22

Atxn1l	12.59073563	4.843868091	1.15766E-32	0	875
Azin1	13.88690753	6.511323752	4.38827E-42	0	2149
B4galt6	11.85117607	5.743677786	1.89854E-27	0	524
Basp1	0.851126078	7.040008099	0.040922529	1153	2729
Baz1b	13.42667229	6.795630623	1.05641E-38	0	1562
Bcat2	12.76885397	5.224858749	6.19642E-34	0	990
Bcl11b	1.495562499	3.436496663	0.000689284	134	496
Bcl2	13.40433538	6.559396035	1.53981E-38	0	1538
Bcl2l11	1.315025361	6.185781686	0.002323644	211	689
Bcl2l13	5.139184529	5.275487135	1.17752E-23	47	2178
Bcl6	13.37974031	6.906510453	2.2992E-38	0	1512
Bhlhe41	10.1224381	2.185904865	2.8264E-16	0	158
Bmi1	9.123731766	3.994685864	5.96009E-11	0	79
Bnc2	8.727660335	0.442569242	2.96087E-10	0	60
Bnip3	12.46687357	4.441530822	8.8812E-32	0	803
Bnip3l	11.64436177	5.784114615	4.98152E-26	0	454
Bod1l	11.92886826	5.59342072	5.57861E-28	0	553
Braf	12.78190826	5.828788074	4.97253E-34	0	999
Bre	5.80245348	5.698353812	3.11306E-10	1	81
Brpf3	11.52865736	4.191002355	3.13616E-25	0	419
Btg1	12.76885397	6.941988362	6.19642E-34	0	990
Cacnb4	12.33531111	4.379799616	7.58575E-31	0	733
Camk2n1	1.884709973	7.011712067	9.53605E-06	1222	5921
Camkk2	0.996927805	6.465726399	0.030255908	79	207
Capns1	1.308968277	8.956843947	0.001729153	3501	11381
Cask	11.5926168	3.143118644	1.12449E-25	0	438
Cav1	9.664631256	2.844755125	9.584E-14	0	115
Cbx5	13.83357319	6.053163818	1.08253E-41	0	2071
Ccbe1	10.96274382	2.244535092	1.83379E-21	0	283
Ccdc88a	5.071948629	5.228057389	1.47147E-20	18	799
Ccdc88c	11.59590581	7.403523787	1.06002E-25	0	439
Ccnd2	0.868485222	8.118379211	0.036858262	1292	3095
Ccny	12.22067684	5.482809687	4.90738E-30	0	677
Cd164	13.59391224	7.836589261	6.26082E-40	0	1754
Cd47	12.26060006	7.674874057	2.62644E-30	0	696
Cdk12	10.23641147	5.425381122	5.53007E-17	0	171
Cdk5r1	11.47250634	4.921630183	7.54177E-25	0	403
Cdo1	4.637872829	3.950541081	2.93931E-19	31	1016
Cdv3	13.78252767	5.823014274	2.57539E-41	0	1999
Celf2	1.726920764	8.76881608	5.44095E-05	480	2085
Celsr2	11.6726729	2.867389844	3.16491E-26	0	463
Cenpm	6.533461152	2.083124078	0.00143701	0	13
Cers5	14.14667781	6.984314278	5.32254E-44	0	2573
Chd2	4.86748075	6.131117756	3.20291E-17	10	387
Chl1	13.18493221	4.253925278	6.06753E-37	0	1321
Chmp1a	13.30232824	7.493118362	8.4431E-38	0	1433
Chmp2b	13.68475083	5.949808902	1.33969E-40	0	1868

Chst10	10.08547751	3.974911751	4.32335E-16	0	154
Chst11	11.34148055	4.04542608	5.52193E-24	0	368
Cited2	7.16757829	5.962195396	6.40509E-30	6	1152
Clcn3	7.731059524	6.384930632	1.61305E-06	0	30
Clock	13.11214622	4.793155387	2.06132E-36	0	1256
Clptm1l	12.36645767	5.848298795	4.56068E-31	0	749
Cnn3	11.14442949	6.064238134	1.11551E-22	0	321
Cnnm3	12.59238321	5.268926682	1.15766E-32	0	876
Cnot6l	13.87747874	7.602789868	5.13422E-42	0	2135
Cntn1	9.90632954	1.356866819	4.7536E-15	0	136
Coa4	11.81776222	4.03308628	3.15278E-27	0	512
Cobl	11.87844169	3.051461768	1.21317E-27	0	534
Cog3	11.12179083	3.759565546	1.65182E-22	0	316
Coro1c	14.27635444	7.802664761	5.86045E-45	0	2815
Cpeb2	13.36920714	4.435095177	2.76472E-38	0	1501
Cpsf6	11.78066864	6.239317656	5.87571E-27	0	499
Creb3l2	1.701325448	5.661305968	6.83784E-05	490	2091
Crebrf	11.28961417	5.549943486	1.28287E-23	0	355
Crtc1	12.58908616	4.100747212	1.1942E-32	0	874
Csnk1d	13.7636415	7.517966579	3.56105E-41	0	1973
Csnk2a1	11.84289488	5.830137297	2.20817E-27	0	521
Ctdsp1	6.14318155	6.15390255	9.97775E-24	7	659
Ctdspl	11.40660868	3.614144408	2.00068E-24	0	385
Ctnnd1	13.95508496	5.662602949	1.37636E-42	0	2253
Ctsb	0.98863262	9.618976049	0.01721179	3609	9396
Cttn	8.842875745	5.044156079	7.59943E-11	0	65
Cux1	12.37605466	7.00005907	3.95106E-31	0	754
Cygb	1.23620058	7.133111645	0.003149561	1275	3941
Cyld	12.23129053	7.062942433	4.19122E-30	0	682
D17Wsu92e	1.270605252	7.216015769	0.002468364	966	3058
Daam1	11.75735853	2.94295918	8.51021E-27	0	491
Dagla	11.74259611	5.180461169	1.05427E-26	0	486
Dap	8.864854445	5.618689546	7.59943E-11	0	66
Dazap2	3.469514577	8.895253196	3.65271E-14	256	3722
Dchs1	12.83018518	3.922018765	2.27533E-34	0	1033
Dcun1d3	9.761540091	2.240062787	2.85416E-14	0	123
Dcx	7.778146178	1.069523187	1.08934E-06	0	31
Ddah1	12.32939553	4.823042842	8.4711E-31	0	730
Dgkg	11.53552503	3.489461009	2.77354E-25	0	421
Dixdc1	8.864854445	3.986749434	7.59943E-11	0	66
Dmxl1	9.262656685	4.203505671	1.25887E-11	0	87
Dnajb9	11.53552503	4.785700399	2.77354E-25	0	421
Dnmt3a	11.46892327	4.636557995	8.03917E-25	0	402
Dpp8	8.14439843	6.605725329	5.79163E-08	0	40
Dpy19l3	11.30577257	3.232473317	9.65553E-24	0	359
Dyrk2	5.689054884	6.229237038	1.1883E-20	7	481
Ednrb	12.51457662	3.62998025	4.13998E-32	0	830

Ehd1	13.25941636	7.239758055	1.74579E-37	0	1391
Eif3j1	5.015453072	5.634452955	1.06817E-19	15	641
Eif3j2	11.91576805	3.722192202	6.75923E-28	0	548
Eif4e2	12.48295048	6.542804072	6.79243E-32	0	812
Eif4ebp2	1.110714763	7.005041056	0.011575783	139	394
Emc7	6.671047416	6.291953284	1.30134E-29	11	1485
Eme2	11.53894664	3.296809495	2.60886E-25	0	422
Eny2	11.91049448	5.153704014	7.4443E-28	0	546
Ep300	1.307379429	6.123786088	0.002778046	161	523
Epas1	14.05585198	5.801706612	2.49365E-43	0	2416
Epdr1	11.67888952	2.872928302	2.99217E-26	0	465
Ephb1	3.903996188	4.303697398	6.63467E-16	64	1259
Erbb2ip	13.52398102	5.592673462	2.02158E-39	0	1671
Erich5	12.80482817	4.546471462	3.3989E-34	0	1015
Erp44	13.57820043	5.958750301	8.21658E-40	0	1735
Etf1	14.12008247	5.914282523	8.36636E-44	0	2526
Etl4	8.727660335	0.442569242	2.96087E-10	0	60
Etv3	10.95251704	5.644780139	2.00104E-21	0	281
Ezr	1.62758259	8.979785081	0.000120569	919	3726
Fam110b	11.48320248	3.485439119	6.23276E-25	0	406
Fam122a	11.09414702	5.237230619	2.46134E-22	0	310
Fam160a2	12.93778428	5.291779131	3.79088E-35	0	1113
Fam178a	2.026165546	5.276943249	8.84329E-06	83	444
Fam53c	11.84289488	4.973892637	2.20817E-27	0	521
Fbxw7	12.42310476	5.585715371	1.82063E-31	0	779
Fcho2	12.45061547	5.128333436	1.16461E-31	0	794
Fchsd2	10.47658689	4.450716315	1.99391E-18	0	202
Fdx1	12.88229638	4.095787052	9.37646E-35	0	1071
Fgf14	12.5808104	3.691002746	1.39577E-32	0	869
Flot1	0.965715634	7.806481517	0.020227878	1776	4551
Fnta	4.098736128	5.907587186	1.27159E-13	14	317
Foxn2	11.96238234	3.906646899	3.16363E-28	0	566
Foxo4	12.20998449	5.003550329	5.98549E-30	0	672
Foxp1	1.672309651	8.715216753	8.95032E-05	477	1995
Foxp2	11.8036081	2.984340674	4.07877E-27	0	507
Fut9	6.295281956	-0.153292309	0.004174171	0	11
Fxr1	1.653872269	5.491389413	0.000115726	363	1499
Fyttd1	11.88920527	4.865199391	1.04678E-27	0	538
Gab2	8.521732932	4.140914705	2.27371E-09	0	52
Galnt2	12.15083516	4.644603524	1.52377E-29	0	645
Gatsl2	9.159737163	2.202122439	3.78455E-11	0	81
Gdnf	10.74301706	2.062793036	4.29278E-20	0	243
Git2	9.418837245	6.903279191	7.10076E-33	1	995
Gmps	7.952481566	4.397088286	3.47842E-07	0	35
Gna11	11.50085501	5.035535401	4.84467E-25	0	411
Gnai3	12.67711761	5.223953566	2.80173E-33	0	929
Gng12	14.05046798	6.984890527	2.73723E-43	0	2407

Gng5	11.62838993	4.434172368	6.26929E-26	0	449
Golph3l	8.214544939	3.786326275	4.10802E-08	0	42
Gphn	11.83734756	4.696454371	2.3227E-27	0	519
Gpm6b	2.132021279	5.986504713	8.14387E-07	516	2968
Gpr155	11.63159848	3.959050089	6.26929E-26	0	450
Grb2	11.25258217	7.963590904	2.12609E-23	0	346
Grhl2	11.4796459	3.812753858	6.64061E-25	0	405
Gria1	5.752615128	4.444745864	6.48551E-26	21	1493
Gxylt1	8.388277018	4.681660439	1.21318E-25	1	487
Hdac7	1.896025087	7.514909447	9.99711E-06	534	2608
Hes1	1.430523244	7.324362512	0.000707093	706	2497
Hn1	11.55255252	6.228820411	2.17314E-25	0	426
Hnrnpa1	10.88421522	5.90236687	5.84468E-21	0	268
Hspa13	11.76029297	3.843613116	8.06884E-27	0	492
Hspa2	11.14442949	3.910448683	1.11551E-22	0	321
Igsf3	11.79790734	4.555119048	4.52468E-27	0	505
Insig1	12.57748676	4.469976659	1.44017E-32	0	867
Irf2bpl	6.078325575	6.875642823	7.97032E-30	36	3201
Itga3	13.616759	4.662699363	4.28662E-40	0	1782
Itgb1	4.241538009	7.229850592	4.00305E-17	36	896
Jade1	11.33362147	5.232078313	6.34243E-24	0	366
Jade2	12.76447615	5.624343408	6.73279E-34	0	987
Jam2	9.141846785	4.81849104	4.74447E-11	0	80
Jmjd6	13.55303859	5.511704784	1.25249E-39	0	1705
Kcmf1	11.54576562	6.383827868	2.30929E-25	0	424
Kcnh5	11.92364244	3.09210176	5.85206E-28	0	551
Kdelr1	14.63360296	8.160517015	1.30709E-47	0	3606
Kdelr2	2.373747791	5.718780552	7.94762E-08	213	1449
Kif2a	12.5058609	5.471482538	4.7198E-32	0	825
Klf13	1.617179942	8.099925265	0.000152141	429	1727
Klf7	3.887175197	7.185961266	2.55351E-16	109	2118
Klhl2	1.68221674	5.310224922	0.000213809	84	354
Klhl9	13.07137763	4.954446748	4.02242E-36	0	1221
Kpna3	9.203469042	4.662580715	2.42676E-31	1	857
Kpnb1	13.5715334	6.736246364	9.19771E-40	0	1727
Kras	13.14057705	5.997696034	1.27661E-36	0	1281
Ktn1	12.02716158	6.468552971	1.15501E-28	0	592
Lcor	11.368655	5.904285456	3.66076E-24	0	375
Ldb1	4.444584706	5.733848793	1.55616E-18	37	1060
Ldlr	15.14747883	8.25831905	1.95327E-51	0	5149
Lekr1	9.968550294	1.407654454	2.19182E-15	0	142
Lhfpl2	9.08680473	0.710164198	9.46504E-11	0	77
Lnx2	5.653664318	4.79226097	0.040964585	0	7
Lpp	12.54382231	5.118567783	2.54769E-32	0	847
Lrch2	6.009851765	-1.061396486	0.012730848	0	9
Lrig1	3.929424803	0.131195511	1.25995E-05	2	42
Lrrc58	6.95262524	4.976770368	3.65816E-27	5	830

Lrrk1	11.53552503	4.57274873	2.77354E-25	0	421
Lsm11	12.88094888	4.163764523	9.61989E-35	0	1070
Lsm14a	12.41381669	6.298823246	2.16664E-31	0	774
Ltn1	12.85510421	5.593941681	1.49336E-34	0	1051
Maf	4.320546923	3.894390992	4.55209E-16	20	527
Mal2	13.51619033	6.728297246	2.31076E-39	0	1662
Map2k1	1.579454116	6.629708021	0.000216634	409	1604
Map2k3	7.464315052	6.977097897	2.73829E-36	13	3037
Map3k3	7.219393581	6.001567533	4.77659E-05	0	21
Map4k5	11.46173031	5.150470484	8.57079E-25	0	400
Mapk1	12.16197328	6.167218971	1.29194E-29	0	650
Mapk9	11.13088897	4.278951577	1.41073E-22	0	318
Mapkapk2	6.113031666	8.082208402	6.18997E-29	25	2280
Mapre1	1.53754859	8.48556611	0.00025655	2261	8612
Mark2	12.97867315	6.644915586	1.916E-35	0	1145
Mast4	13.66453098	5.648314625	1.8941E-40	0	1842
Mdp1	10.02819816	6.189826562	1.03437E-15	0	148
Med13	4.144332938	5.44858443	6.51087E-16	28	652
Mef2a	0.969332188	5.937586739	0.021040319	608	1562
Mef2c	11.74851922	8.182203607	9.46994E-27	0	488
Meis2	9.626543741	1.255360712	1.64072E-13	0	112
Mfap3	8.313756789	6.838133928	1.50714E-08	0	45
Mfsd5	12.45967036	6.873615686	1.01664E-31	0	799
Mfsd6	11.76029297	4.538684967	8.06884E-27	0	492
Mgat2	10.77818621	5.910642619	2.6315E-20	0	249
Mitf	11.67578456	2.870161878	2.99217E-26	0	464
Mme	1.068715305	7.025786351	0.010452725	1263	3476
Mon2	6.9969563	5.999970599	1.91268E-32	12	2029
Morf4l2	1.974276897	4.134852966	7.19644E-06	184	949
Mospd2	11.69738026	3.436800498	2.14225E-26	0	471
Mpp1	9.784783276	5.773611227	2.41191E-14	0	125
Mta1	12.31948199	6.270729068	9.82183E-31	0	725
Mtch2	11.87031597	6.23198788	1.40724E-27	0	531
Mtss1	5.842722641	5.96312264	0.022676824	0	8
Myadm	8.315389218	6.667184415	3.82318E-25	1	463
Mycbp2	0.928458324	7.247902826	0.02567832	1422	3551
Myo1b	9.549055015	3.994514899	8.2306E-34	1	1089
Nap1l1	13.07019571	7.976205397	4.11434E-36	0	1220
Ndnl2	12.62816206	4.109078447	6.2676E-33	0	898
Ndrg1	12.35096844	5.11161698	5.87425E-31	0	741
Nedd4l	0.97875125	6.284512733	0.019670323	722	1867
Nf1	12.69409719	4.277902733	2.1544E-33	0	940
Nfat5	3.800404047	5.745338637	7.67804E-16	122	2232
Nlgn2	13.03428037	4.262523492	7.45768E-36	0	1190
Nnmt	12.34707001	3.476299909	6.31777E-31	0	739
Npc2	1.657093611	8.962349112	8.63427E-05	2065	8545
Nrp2	0.875376248	4.10861563	0.045324914	157	378

Nt5dc3	12.76008502	5.016408863	7.11702E-34	0	984
Ntng1	9.830175532	1.294995859	1.24369E-14	0	129
Nuak1	11.54236016	2.891985302	2.45432E-25	0	423
Nudt4	14.31428712	7.103936915	3.05339E-45	0	2890
Obfc1	11.34929705	4.886529031	4.81125E-24	0	370
Osbpl8	11.22735532	6.170191633	3.30544E-23	0	340
Pacs1	8.376299594	5.633468656	7.89453E-09	0	47
Paip2	5.398511841	6.385082877	8.89011E-23	18	1002
Pak1	11.7216716	6.902191097	1.45876E-26	0	479
Palld	13.48637322	4.88783147	3.83974E-39	0	1628
Pam	5.156854096	4.837069141	1.59866E-23	41	1924
Pan3	11.65386067	5.544775445	4.19824E-26	0	457
Pard3b	5.847433309	3.867976421	8.19745E-24	11	839
Parm1	10.99299625	4.846992786	1.09307E-21	0	289
Pcmt1	1.579830825	5.914858222	0.000269293	209	820
Pcnx	2.851462416	4.186665988	3.83029E-09	45	427
Pdcd4	11.94185059	4.069802426	4.39748E-28	0	558
Pde7a	7.083169244	5.887912569	3.78706E-17	1	197
Pdzd2	8.886503329	0.559533944	5.8403E-11	0	67
Pdzd8	10.65745981	4.575222677	1.60555E-19	0	229
Pex5	10.80685279	4.03154862	1.7911E-20	0	254
Pfn1	13.41647745	5.698565414	1.24068E-38	0	1551
Pfn2	10.79545442	2.100656499	2.1694E-20	0	252
Pgap1	11.61223929	5.552943075	8.38195E-26	0	444
Pgrmc2	13.82798984	6.940698854	1.19021E-41	0	2063
Phactr4	11.61872126	5.938032459	7.45965E-26	0	446
Phf13	10.80685279	4.535761693	1.7911E-20	0	254
Phf20l1	11.09879126	5.381016816	2.27147E-22	0	311
Phf21a	11.52865736	7.491721562	3.13616E-25	0	419
Phf3	1.092620196	6.486104359	0.010006644	387	1083
Phip	13.23957646	6.194267735	2.45515E-37	0	1372
Phldb1	9.477599065	1.489495175	1.07555E-12	0	101
Pi4k2a	2.063665056	5.190729717	3.82276E-06	129	708
Pigx	9.048907631	5.131881042	1.5162E-10	0	75
Pik3c2a	1.863491201	4.757562432	2.5384E-05	139	664
Pink1	1.27114319	6.22143774	0.002625859	545	1726
Pkp4	11.67578456	5.373955087	2.99217E-26	0	464
Plcxd3	11.52521122	2.73643418	3.33564E-25	0	418
Plekha1	11.24002389	5.301690743	2.64839E-23	0	343
Plekha5	12.48117298	5.611137347	7.02284E-32	0	811
Plekha7	11.28146659	2.894295433	1.37802E-23	0	353
Polr3g	8.406582789	2.817044188	5.74928E-09	0	48
Pou2f1	12.33334194	5.384179538	7.86965E-31	0	732
Pou3f3	1.507131623	5.857440871	0.000359067	836	3118
Ppfibp1	12.56579335	3.706594349	1.79508E-32	0	860
Ppm1l	11.82898638	4.487756612	2.70483E-27	0	516
Ppp1r11	13.86662698	7.363103638	6.17396E-42	0	2119

Ppp1r12c	12.2832184	6.745092238	1.79091E-30	0	707
Ppp1r14b	12.65993581	6.394212891	3.76379E-33	0	918
Ppp2ca	13.08431519	6.934255246	3.28524E-36	0	1232
Ppp2r5c	8.506860418	6.171206367	4.78407E-32	2	1006
Ppp3ca	1.269690389	7.625329808	0.002516714	831	2629
Ppp4r2	11.16671838	6.20659957	8.18434E-23	0	326
Prdm1	10.52570063	4.664396657	1.00809E-18	0	209
Prkacb	12.82738965	6.401437227	2.33656E-34	0	1031
Prkce	12.32147015	5.955931686	9.82183E-31	0	726
Prkci	11.93407522	4.044496849	5.07083E-28	0	555
Prkd1	11.28146659	2.521765583	1.37802E-23	0	353
Prosc	11.68507947	4.255665334	2.67546E-26	0	467
Prpf19	11.56603128	6.675089642	1.70673E-25	0	430
Prr13	13.57820043	8.508362525	8.21658E-40	0	1735
Ptdss1	12.71995149	6.654472553	1.39889E-33	0	957
Ptpn4	11.05644483	3.288101773	4.34912E-22	0	302
Ptpn9	10.77818621	2.888061448	2.6315E-20	0	249
Ptrf	1.783414102	7.285985663	2.66443E-05	1130	5104
Qser1	7.778146178	2.125741179	1.08934E-06	0	31
Rab10	1.507574774	8.015809926	0.000386063	539	2011
Rab21	11.8675972	5.783275188	1.47889E-27	0	530
Rab23	5.17322348	1.487902699	4.34345E-13	3	147
Rab27a	11.8757382	4.744692161	1.27456E-27	0	533
Rab34	11.37632604	2.605040728	3.19677E-24	0	377
Rab35	11.70957706	5.262244456	1.81566E-26	0	475
Rabgap1	8.136536214	6.168001241	6.14733E-24	1	409
Rac1	11.40109231	8.546555054	2.29482E-53	2	7480
Rala	1.35335157	7.092141099	0.001406783	516	1730
Ralgapb	10.59308387	3.932183529	3.74804E-19	0	219
Ralgps2	5.653664318	7.353756988	0.040964585	0	7
Rap2b	9.460278705	5.121078685	3.51351E-33	1	1024
Rapgef4	9.105386392	3.743636107	7.50284E-11	0	78
Rapgef5	11.96492838	5.823806711	3.01931E-28	0	567
Rarg	6.62851447	6.986555798	3.4279E-32	21	2740
Rasa1	12.30347726	5.385767797	1.27514E-30	0	717
Rassf8	10.67622547	2.31896099	1.17683E-19	0	232
Rbfox1	8.50486055	3.03984525	1.9021E-26	1	528
Rbm25	1.657474632	8.547248383	8.91981E-05	1178	4876
Rbmx	6.998613759	4.546033125	0.000193974	0	18
Rcc2	8.629840735	7.138037635	2.54243E-38	4	2135
Rcor1	12.07036345	5.947225854	5.68925E-29	0	610
Rell1	13.6551023	5.713955717	2.23911E-40	0	1830
Rhob	14.59672973	7.397227733	2.46693E-47	0	3515
Rictor	0.945562731	5.670290511	0.024180672	702	1774
Rnf130	9.784783276	4.321871218	2.41191E-14	0	125
Rnf145	12.53528201	6.328125631	2.89717E-32	0	842
Rnf44	13.62402642	6.622205284	3.7829E-40	0	1791

Rpia	11.56938139	6.28435024	1.60728E-25	0	431
Rprd1b	11.7336656	4.053053954	1.23948E-26	0	483
Rreb1	12.63776788	5.918975903	5.38879E-33	0	904
Rsf1	12.96601942	5.217830377	2.32404E-35	0	1135
Rtn4	2.429775691	8.077876388	2.51642E-08	597	4221
Rufy1	7.219393581	5.905687107	4.77659E-05	0	21
Runx1t1	11.71261021	2.902995198	1.71867E-26	0	476
Rwdd4a	13.21732597	5.599388025	3.54086E-37	0	1351
Ryk	3.855259448	5.601670651	2.00621E-16	166	3154
Safb	12.52324001	5.839392808	3.51769E-32	0	835
Sar1a	12.82598985	6.476902366	2.39951E-34	0	1030
Sav1	12.54722432	4.531029213	2.38968E-32	0	849
Sbds	13.33517152	6.129651953	4.84707E-38	0	1466
Sc5d	13.1461966	4.885534043	1.17124E-36	0	1286
Scaf11	12.29944818	5.730833112	1.37457E-30	0	715
Scyl2	12.49003867	5.56667275	6.1472E-32	0	816
Sec14l1	14.19302207	6.645736375	2.4264E-44	0	2657
Sec62	1.073462956	8.431577189	0.009967789	1851	5111
Sema3a	12.07979068	3.233054649	4.78096E-29	0	614
Sgk3	5.842722641	5.97401295	0.022676824	0	8
Sh3bp4	10.82378325	2.362361301	1.34732E-20	0	257
Sh3kbp1	7.529822351	6.781034923	3.41543E-25	2	511
Sh3pxd2b	10.81816181	3.794372944	1.48092E-20	0	256
Shc1	7.867945609	4.031247474	7.40189E-07	0	33
Sik1	9.477599065	3.366052662	1.07555E-12	0	101
Skil	11.94443309	5.252126069	4.19428E-28	0	559
Slc10a3	5.653664318	4.761338071	0.040964585	0	7
Slc12a6	10.41833	3.481362038	4.52701E-18	0	194
Slc1a1	12.22280583	3.36290364	4.71717E-30	0	678
Slc23a2	12.87148094	4.361690374	1.12253E-34	0	1063
Slc25a20	10.46943214	4.460216648	2.23805E-18	0	201
Slc25a51	12.72446631	5.897342247	1.28429E-33	0	960
Slc2a13	6.098207328	2.873277169	2.63007E-21	5	459
Slc30a9	9.547212579	1.626555983	4.12739E-13	0	106
Slc33a1	2.319751388	5.14248454	1.91127E-07	148	970
Slc35a1	12.59402892	4.193132538	1.12227E-32	0	877
Slc39a1	13.68552288	5.298675694	1.33969E-40	0	1869
Slc39a10	11.12179083	3.095938914	1.65182E-22	0	316
Slc39a13	12.59402892	4.369220745	1.12227E-32	0	877
Slc39a9	12.47939328	4.9792465	7.26139E-32	0	810
Slc43a2	11.36094296	5.977568132	4.1952E-24	0	373
Slc5a3	9.229159389	3.045212626	1.94396E-11	0	85
Slco3a1	1.404538534	4.905567905	0.001150898	234	813
Slmo2	12.34120254	5.061620792	7.04944E-31	0	736
Smad1	10.70087258	2.455085489	8.65875E-20	0	236
Smad7	6.639263481	-0.811006747	0.00085757	0	14
Smc6	13.24691768	6.461961837	2.13234E-37	0	1379

Smco4	13.59801864	5.518635234	5.87541E-40	0	1759
Smim14	1.1539106	8.046161112	0.006790843	325	949
Smpd3	13.03912105	4.14757598	6.958E-36	0	1194
Snap23	12.82178227	5.613391	2.59916E-34	0	1027
Snf8	12.14411078	5.979341091	1.72579E-29	0	642
Snx1	8.414687743	5.489117758	7.94113E-26	1	496
Snx16	11.37632604	3.802947791	3.19677E-24	0	377
Snx18	11.56938139	4.792088208	1.60728E-25	0	431
Snx29	12.8426985	4.136642654	1.8417E-34	0	1042
Socs7	12.20783648	5.374363317	6.2292E-30	0	671
Sort1	13.60293095	6.846672824	5.34293E-40	0	1765
Sowahc	4.692474703	4.594478648	2.15287E-14	7	241
Spats2l	11.78931374	2.971542581	5.02151E-27	0	502
Spcs2	12.07508476	6.45703588	5.21458E-29	0	612
Spen	12.66933306	6.589495331	3.24591E-33	0	924
Spin1	12.82458868	4.8535959	2.46422E-34	0	1029
Spopl	11.73664858	4.222472315	1.17425E-26	0	484
Sppl2a	1.058228378	6.232815798	0.012059099	512	1399
Spry4	9.90632954	1.356866819	4.7536E-15	0	136
Spsb1	10.9161414	3.22686239	3.40027E-21	0	274
Srpk1	12.94553974	5.432887538	3.27004E-35	0	1119
Srsf2	2.763746913	7.252422507	6.07814E-09	54	482
St7	9.574143917	2.797314798	3.42262E-13	0	108
Stag1	11.78643779	5.271915525	5.29089E-27	0	501
Stk35	11.46533127	6.136275495	8.03917E-25	0	401
Strbp	12.17082227	8.089517281	1.09657E-29	0	654
Stx5a	8.628367727	5.068170307	6.94986E-10	0	56
Surf2	12.42680331	3.933842806	1.75862E-31	0	781
Syncrip	1.322266689	7.26663281	0.001651844	950	3117
Sypl	7.469382554	6.296544593	1.26863E-05	0	25
Taok1	11.6347999	6.334419154	5.91791E-26	0	451
Tbc1d22b	11.45812034	4.334074893	9.13906E-25	0	399
Tbc1d24	11.70349155	3.19106636	2.02709E-26	0	473
Tbl1x	3.792550246	5.097311046	1.39486E-15	95	1729
Tceanc2	12.89436762	6.496455257	7.64539E-35	0	1080
Tcf12	8.12168864	6.033958982	7.86484E-37	6	2232
Tead1	12.69409719	3.795705157	2.1544E-33	0	940
Tex2	12.70632166	4.273202509	1.75956E-33	0	948
Tfdp2	11.20167948	3.653590933	4.80355E-23	0	334
Tgfbi	3.399725644	9.20156158	4.92112E-14	938	12990
Tgfbr1	13.29019739	6.099834531	1.04554E-37	0	1421
Tiam1	8.406582789	4.308699767	5.74928E-09	0	48
Tle4	14.14891877	6.406576085	5.09501E-44	0	2577
Tm9sf4	8.267890554	4.898988171	7.99638E-25	1	448
Tmbim6	0.953167402	8.648322304	0.021966798	1575	4001
Tmed7	11.00294104	4.871798646	1.0038E-21	0	291
Tmem115	11.1755384	2.602527831	7.02034E-23	0	328

Tmem127	6.533461152	6.153785675	0.00143701	0	13
Tmem50b	12.74683084	5.848353662	8.89709E-34	0	975
Tmem57	13.11902089	5.739201958	1.84715E-36	0	1262
Tmpo	4.021499275	6.980801372	1.54444E-16	58	1238
Tmsb4x	1.718613323	13.32353642	4.59611E-05	21377	92309
Tmx1	11.60898736	6.819072421	8.88684E-26	0	443
Tnfaip8	13.9570046	8.416162281	1.34253E-42	0	2256
Tnk2	11.3839565	5.858732494	2.98814E-24	0	379
Tnpo1	13.90889171	6.983200937	3.01487E-42	0	2182
Tnrc6b	2.132673732	7.06552533	1.62294E-06	144	829
Tns1	11.20167948	4.374320605	4.80355E-23	0	334
Tox4	12.01000517	5.941368634	1.51423E-28	0	585
Tpd52	12.25228649	7.886730309	2.94955E-30	0	692
Trabd2b	13.02454999	4.103409283	8.77425E-36	0	1182
Traf4	11.00294104	4.120958822	1.0038E-21	0	291
Tram1	1.042902503	7.450669638	0.012484359	1153	3117
Trim2	12.4193967	6.130441811	1.95158E-31	0	777
Trio	11.7336656	3.938875006	1.23948E-26	0	483
Trp53inp1	10.36535108	5.870034988	9.34593E-18	0	187
Trpm3	6.533461152	-0.857454566	0.00143701	0	13
Tsc1	11.77777539	4.961532801	6.19295E-27	0	498
Tsnax	10.53943141	6.711028119	8.06364E-19	0	211
Ttc14	11.37249562	4.134860528	3.42059E-24	0	376
Ttc19	7.525652942	2.477223187	8.28134E-06	0	26
Ttyh3	1.996784918	7.123496373	4.72007E-06	252	1320
Ube2f	10.87341421	6.23995073	6.40425E-21	0	266
Ube2g1	2.967484452	5.108043364	9.12542E-09	23	237
Ube2h	14.0275114	6.694883721	4.03544E-43	0	2369
Ube2k	7.514300283	6.478187806	2.04061E-32	6	1465
Ube2l3	13.69552219	6.894913727	1.12036E-40	0	1882
Ube2r2	10.69970503	8.46437149	3.91321E-48	2	4600
Uchl3	13.32826697	5.494044101	5.53566E-38	0	1459
Uck2	13.06189508	5.567819856	4.71374E-36	0	1213
Usp2	8.928850233	2.072739783	3.47788E-11	0	69
Usp24	12.93259072	5.681345825	4.08297E-35	0	1109
Usp36	14.21350803	6.269136517	1.69689E-44	0	2695
Usp47	12.73792659	6.148040192	1.02396E-33	0	969
Usp5	8.751451286	5.341823406	2.24222E-10	0	61
Vamp3	11.52865736	6.058041086	3.13616E-25	0	419
Vps26a	11.35707143	5.102036338	4.49225E-24	0	372
Vps26b	11.1489149	5.10356757	1.03204E-22	0	322
Vps36	11.1034206	4.710255909	2.09679E-22	0	312
Wasl	12.452431	3.970602541	1.12563E-31	0	795
Wdr82	12.721458	6.673011239	1.35955E-33	0	958
Whamm	10.95763949	3.736462291	1.83379E-21	0	282
Whsc1	7.410828025	4.520696277	1.95811E-05	0	24
Wipi2	11.62838993	5.212861315	6.26929E-26	0	449

Wisp1	11.42521755	5.267367076	1.53661E-24	0	390
Wnt2	5.842722641	-1.117656276	0.022676824	0	8
Wnt4	13.46224795	4.582075431	5.80797E-39	0	1601
Xiap	11.84566056	5.354172979	2.09951E-27	0	522
Yaf2	11.81494249	4.935102046	3.3187E-27	0	511
Yod1	11.76322146	3.485884574	7.65122E-27	0	493
Ywhae	4.828908664	8.871541142	3.91442E-23	211	7872
Zadh2	12.20783648	3.984666573	6.2292E-30	0	671
Zbtb41	11.34929705	3.71373828	4.81125E-24	0	370
Zbtb44	11.99513551	3.664831897	1.90187E-28	0	579
Zc3h15	12.5197809	6.042025875	3.75404E-32	0	833
Zcchc14	11.55930764	4.999641543	1.9253E-25	0	428
Zdhhc17	10.32628131	3.141191878	1.73828E-17	0	182
Zdhhc20	6.120315383	5.329102882	9.53559E-12	1	101
Zdhhc6	10.73707128	4.644778811	4.73969E-20	0	242
Zeb1	13.41368449	5.676165361	1.30929E-38	0	1548
Zeb2	11.97759164	5.925662123	2.5071E-28	0	572
Zfand5	14.04265545	6.901246808	3.11325E-43	0	2394
Zfp202	11.69431488	4.21433247	2.26424E-26	0	470
Zfp207	3.33452987	7.778078096	7.52292E-13	105	1391
Zfp292	7.778146178	5.405020324	1.08934E-06	0	31
Zfp36	2.550586279	8.047416075	5.67321E-09	612	4705
Zfp36l1	2.170061183	8.11548788	4.96814E-07	687	4057
Zfp609	11.12634707	3.450125098	1.52633E-22	0	317
Zfp704	13.77020703	5.195081408	3.17994E-41	0	1982
Zfp827	6.522412521	5.848972	1.85236E-28	10	1219
Zmym2	10.84051733	4.735220576	1.11639E-20	0	260
Znrf1	7.956500722	4.580507058	9.84312E-23	1	361
Zzef1	1.859199722	5.821636369	1.85144E-05	260	1238

Table S27. Immune-related KEGG pathways enriched in target genes of CRMCs of KO and MS-CKO mice.

Category	Term code	Term	Count	%	PValue	Genes	List Total	Pop Hits	Pop Total	Fold Enrichment	Bonferroni	Benjamini	FDR
KEGG_PAT	mmu05100	Bacterial invasion of epithelial cells	11	1.627219	2.98E-04	ITGB1, ACTR2, CTTN, SHC1, CAV1, ARPC1B, RAC1, WASL, CRK, WASF2, CD2AP	328	76	9330	4.117057	0.076422647	0.0036131	0.00272
KEGG_PAT	mmu04666	Fc gamma R-mediated phagocytosis	12	1.775148	3.87E-04	ACTR2, MAP2K1, MARCKS, PAK1, PRKCE, ARPC1B, MAPK1, ASAP1, GAB2, RAC1, CRK, WASF2	328	93	9330	3.670338	0.098215328	0.0043826	0.003299
KEGG_PAT	mmu05220	Chronic myeloid leukemia	10	1.47929	0.001287	MAP2K1, SHC1, ABL1, MAPK1, GRB2, BRAF, KRAS, GAB2, CRK, TGFBR1	328	76	9330	3.742779	0.291021406	0.0101091	0.00761
KEGG_PAT	mmu04664	Fc epsilon RI signaling pathway	9	1.331361	0.002041	MAP2K3, MAPK9, MAP2K1, MAPK1, GRB2, FYN, KRAS, GAB2, RAC1	328	66	9330	3.87888	0.420482794	0.0147298	0.011089
KEGG_PAT	mmu05135	Yersinia infection	13	1.923077	0.002728	ITGB1, MAP2K3, ACTR2, GIT2, MAP2K1, ARHGEF12, ARPC1B, WASL, MAPK9, MAPK1, RAC1, CRK, WASF2	328	135	9330	2.73916	0.517785579	0.0186762	0.01406
KEGG_PAT	mmu04062	Chemokine signaling pathway	16	2.366864	0.003045	MAP2K1, SHC1, GNAI3, BRAF, GNG12, PRKCZ, ADCY6, TIAM1, PAK1, GNG5, MAPK1, GRB2, KRAS, RAC1, CRK, PRKACB	328	192	9330	2.370427	0.557063383	0.0193592	0.014574
KEGG_PAT	mmu05166	Human T-cell leukemia virus 1 infection	18	2.662722	0.006333	ATF2, MAP3K3, EGR1, MAP2K1, CRTC1, XIAP, TGFBR1, ADCY6, PPP3CA, MAPK9, ZFP36, CCND2, CREB3L1, CREB3L2, MAPK1, EP300, KRAS, PRKACB	328	248	9330	2.064565	0.816640616	0.0338186	0.025459
KEGG_PAT	mmu05132	Salmonella infection	18	2.662722	0.007681	MAP2K3, ACTR2, RALA, MAP2K1, ARPC1B, WASL, PIK3C2A, RHOB, MAPK9, SNX18, PAK1, BCL2, EXOC4, MAPK1, RAC1, PFN1, KPNA3, PFN2	328	253	9330	2.023764	0.872386293	0.0391141	0.029445
KEGG_PAT	mmu05163	Human cytomegalovirus infection	18	2.662722	0.007979	ATF2, MAP2K1, ARHGEF12, GNAI3, TSC1, GNG12, ADCY6, PPP3CA, GNG5, GNA11, CREB3L1, CREB3L2, MAPK1, GRB2, KRAS, RAC1, CRK, PRKACB	328	254	9330	2.015796	0.882211304	0.0394503	0.029699
KEGG_PAT	mmu04660	T cell receptor signaling pathway	11	1.627219	0.010453	PPP2CA, MAPK9, PPP2CB, PPP3CA, MAP2K1, PAK1, MAPK1, GRB2, FYN, KRAS, PPP2R5C	328	122	9330	2.564724	0.939528797	0.048119	0.036224
KEGG_PAT	mmu05165	Human papillomavirus infection	22	3.254438	0.015215	MAGI1, ITGB1, PRKCI, MAP2K1, ITGA3, TSC1, PPP2R5C, LAMC1, PRKCZ, PPP2CA, PPP2CB, CCND2, CREB3L1, CREB3L2, MAPK1, EP300, GRB2, HES1, KRAS, WNT2, PRKACB, WNT4	328	360	9330	1.738313	0.983320047	0.0644814	0.048542
KEGG_PAT	mmu04650	Natural killer cell mediated cytotoxicity	10	1.47929	0.020466	PPP3CA, MAP2K1, PAK1, SHC1, MAPK1, GRB2, FYN, BRAF, KRAS, RAC1	328	116	9330	2.452166	0.995998949	0.0815598	0.061399
KEGG_PAT	mmu05145	Toxoplasmosis	9	1.331361	0.037313	ITGB1, MAP2K3, MAPK9, GNAI3, BCL2, XIAP, MAPK1, LAMC1, LDLR	328	109	9330	2.34868	0.999961053	0.1328357	0.1
KEGG_PAT	mmu04350	TGF-beta signaling pathway	9	1.331361	0.039069	ACVR1, PPP2CA, SMAD1, PPP2CB, EP300, MAPK1, SKIL, TGFBR1, SMAD7	328	110	9330	2.327328	0.999976074	0.1368526	0.103024
KEGG_PAT	mmu05170	Human immunodeficiency virus 1 infection	15	2.218935	0.040389	MAP2K3, MAP2K1, FBXW11, GNAI3, GNG12, PPP3CA, MAPK9, PAK1, GNG5, GNA11, BCL2, MAPK1, KRAS, RAC1, CRK	328	238	9330	1.79276	0.999983426	0.1368526	0.103024

KEGG_PAT	mmu04668	TNF signaling pathway	9	1.331361	0.048672	MAP2K3, CYLD, MAPK9, ATF2, MAP2K1, CREB3L1, CREB3L2, XIAP, MAPK1	328	115	9330	2.22614	0.999998362	0.1584792	0.119305

Table S28. Neuronal function related KEGG pathways enriched in the predicted targets of upregulated genes of CRMCs of MS-CKO and KO mice

Category	Term code	Term	Count	%	PValue	Genes	List Total	Pop Hits	Pop Total	Fold Enrichment	Bonferroni	Benjamini	FDR
KEGG_PAT	mmu04722	Neurotrophin signaling pathway	16	2.366864	2.00E-05	YWHAE, MAP3K3, MAP2K1, SHC1, SORT1, BRAF, MAPK9, ARHGDIA, MAPKAPK2, BCL2, ABL1, MAPK1, GRB2, KRAS, RAC1, CRK	328	121	9330	3.761338	0.0053197	0.00127	9.56E-04
KEGG_PAT	mmu04360	Axon guidance	19	2.810651	6.26E-05	NTNG1, ITGB1, ARHGEF12, RYK, SEMA3A, GNAI3, L1CAM, PRKCZ, PPP3CA, ABLIM1, PAK1, RASA1, ABL1, MAPK1, KRAS, FYN, RAC1, EPHB1, WNT4	328	181	9330	2.985952	0.0165846	0.0020904	0.001574
KEGG_PAT	mmu04725	Cholinergic synapse	14	2.071006	1.41E-04	MAP2K1, GNAI3, CACNA1B, GNG12, ADCY6, GNG5, GNA11, CREB3L1, CREB3L2, BCL2, MAPK1, KRAS, FYN, PRKACB	328	112	9330	3.55564	0.0369912	0.0028494	0.002145
KEGG_PAT	mmu04810	Regulation of actin cytoskeleton	21	3.106509	1.72E-04	ITGB1, ACTR2, MAP2K1, ARHGEF12, ITGA3, ARPC1B, BRAF, WASL, GNG12, TIAM1, PAK1, TMSB4X, MAPK1, KRAS, RAC1, EZR, PFN1, CRK, PPP1R12C, WASF2, PFN2	328	231	9330	2.58592	0.0449924	0.0028494	0.002145
KEGG_PAT	mmu04728	Dopaminergic synapse	15	2.218935	2.64E-04	GRIA1, ATF2, GNAI3, CACNA1B, PPP2R5C, GNG12, PPP2CA, PPP2CB, PPP3CA, MAPK9, GNG5, CREB3L1, CREB3L2, CLOCK, PRKACB	328	135	9330	3.160569	0.0680108	0.0033536	0.002525
KEGG_PAT	mmu04730	Long-term depression	9	1.331361	0.00109	PPP2CA, GRIA1, PPP2CB, MAP2K1, GNA11, GNAI3, MAPK1, BRAF, KRAS	328	60	9330	4.266768	0.2527134	0.0090984	0.006849
KEGG_PAT	mmu04720	Long-term potentiation	8	1.183432	0.008733	GRIA1, PPP3CA, MAP2K1, EP300, MAPK1, BRAF, KRAS, PRKACB	328	67	9330	3.396432	0.9038649	0.0423956	0.031916
KEGG_PAT	mmu04726	Serotonergic synapse	10	1.47929	0.040492	MAP2K1, GNG5, DUSP1, GNAI3, CACNA1B, MAPK1, BRAF, KRAS, GNG12, PRKACB	328	131	9330	2.171383	0.9999839	0.1368526	0.103024
KEGG_PAT	mmu04724	Glutamatergic synapse	9	1.331361	0.046639	GRIA1, PPP3CA, GNG5, SLC1A1, GNAI3, MAPK1, GNG12, PRKACB, ADCY6	328	114	9330	2.245668	0.9999971	0.1537362	0.115734

Table S29. Cell-cell interaction relted KEGG pathways enriched in the predicted targets of upregulated genes of CRMCs of MS-CKO and KO mice

Category	Term code	Term	Count	%	PValue	Genes	List Total	Pop Hits	Pop Total	Fold Enrichment	Bonferroni	Benjamini	FDR
KEGG_PAT	mmu04530	Tight junction	22	3.254438	3.81E-07	MAGI1, ITGB1, ACTR2, PRKCI, PRKCE, ARPC1B, NEDD4L, ARHGEF18, ARHGAP17, PRKCZ, RAP2C, PPP2CA, PPP2CB, TIAM1, MAPK9, CTTN, AMOTL2, RAC1, WHAMM, EZR, PRKACB, JAM2	328	168	9330	3.724956	1.02E-04	1.02E-04	7.65E-05
KEGG_PAT	mmu04810	Regulation of actin cytoskeleton	21	3.106509	1.72E-04	ITGB1, ACTR2, MAP2K1, ARHGEF12, ITGA3, ARPC1B, BRAF, WASL, GNG12, TIAM1, PAK1, TMSB4X, MAPK1, KRAS, RAC1, EZR, PFN1, CRK, PPP1R12C, WASF2, PFN2	328	231	9330	2.58592	0.0449924	0.0028494	0.002145
KEGG_PAT	mmu04510	Focal adhesion	18	2.662722	7.60E-04	ITGB1, MAP2K1, SHC1, ITGA3, CAV1, XIAP, BRAF, LAMC1, MAPK9, PAK1, CCND2, BCL2, MAPK1, GRB2, FYN, RAC1, CRK, PPP1R12C	328	203	9330	2.522228	0.1836956	0.0069962	0.005267
KEGG_PAT	mmu04520	Adherens junction	10	1.47929	0.004819	PTPN1, CSNK2A1, CTNND1, EP300, MAPK1, FYN, RAC1, WASL, WASF2, TGFBR1	328	92	9330	3.091861	0.7246776	0.0268063	0.02018
KEGG_PAT	mmu04540	Gap junction	9	1.331361	0.01033	MAP2K1, GNA11, GNAI3, MAPK1, GRB2, KRAS, CSNK1D, PRKACB, ADCY6	328	86	9330	2.976815	0.9374973	0.048119	0.036224

Table S30. Dysregulated genes at protein levels detected by proteomics in TG of miR-183C KO vs WT control mice

Accession	Description	WT TG1	WT TG2	WT TG3	KO TG1	KO TG2	KO TG3	Fold Changes	pVal	q-value
**Upregulated genes (215)**										
Q80U49	Centrosomal protein of 170 kDa protein B OS=Mus musculus OX=10090 GN=Cep170b PE=1 SV=2	2.54E+04	3.74E+04	3.52E+04	56596.93	5.17E+04	5.14E+04	1.628698525	0.007069009	0.3110762
P01867	Ig gamma-2B chain C region OS=Mus musculus OX=10090 GN=Igh-3 PE=1 SV=3	8.69E+05	9.63E+05	8.40E+05	1453535.62	1.51E+06	1.36E+06	1.617397335	0.000628816	0.158849
Q8VCM7	Fibrinogen gamma chain OS=Mus musculus OX=10090 GN=Fgg PE=1 SV=1	1.25E+06	1.51E+06	1.38E+06	1888356.49	2.12E+06	2.35E+06	1.53960219	0.008442719	0.3224334
Q8JZU2	Tricarboxylate transport protein, mitochondrial OS=Mus musculus OX=10090 GN=Slc25a1 PE=1 SV=1	2.49E+05	3.05E+05	2.94E+05	477748.59	3.99E+05	4.21E+05	1.529619182	0.006898208	0.3110762
P06327	Ig heavy chain V region VH558 A1/A4 OS=Mus musculus OX=10090 GN=Gm5629 PE=2 SV=1	2.05E+05	2.18E+05	1.41E+05	287412.68	3.25E+05	2.47E+05	1.523517666	0.04013253	0.4270203
P01646	Ig kappa chain V-V region HP 123E6 OS=Mus musculus OX=10090 PE=1 SV=1	4.07E+05	4.98E+05	3.72E+05	614609.05	6.90E+05	6.24E+05	1.511526292	0.00805165	0.3180017
Q8BWL5	RNA-binding motif, single-stranded-interacting protein 3 OS=Mus musculus OX=10090 GN=Rbms3 PE=2 SV=2	5.29E+04	4.95E+04	6.31E+04	79444.29	8.05E+04	8.70E+04	1.492232926	0.004464293	0.2962781
P01865	Ig gamma-2A chain C region, membrane-bound form OS=Mus musculus OX=10090 GN=Igh-1a PE=1 SV=3	3.79E+05	4.92E+05	4.29E+05	538864.47	7.03E+05	6.42E+05	1.449082358	0.028174364	0.3937240
Q5SVR0	TBC1 domain family member 9B OS=Mus musculus OX=10090 GN=Tbc1d9b PE=1 SV=1	9.82E+04	9.62E+04	1.21E+05	134899.03	1.40E+05	1.69E+05	1.407166549	0.031702479	0.3997531
Q06770	Corticosteroid-binding globulin OS=Mus musculus OX=10090 GN=Serpina6 PE=1 SV=1	2.07E+05	1.90E+05	1.97E+05	290266.28	2.38E+05	2.77E+05	1.35798436	0.012523627	0.3480419
Q64362	AKT-interacting protein OS=Mus musculus OX=10090 GN=Aktip PE=1 SV=1	1.57E+05	1.92E+05	2.13E+05	258618.12	2.46E+05	2.57E+05	1.354110588	0.016846124	0.3489800
Q6PHS9	Voltage-dependent calcium channel subunit alpha-2/delta-2 OS=Mus musculus OX=10090 GN=Cacna2d2 PE=1 SV=1	4.13E+05	4.10E+05	4.35E+05	599300.69	5.72E+05	5.31E+05	1.35326451	0.002224419	0.2962781
P01869	Ig gamma-1 chain C region, membrane-bound form OS=Mus musculus OX=10090 GN=Ighg1 PE=1 SV=2	6.75E+05	7.07E+05	6.39E+05	868163.82	8.44E+05	9.56E+05	1.320190356	0.005408804	0.3029392
Q71KT5	Delta(14)-sterol reductase TM7SF2 OS=Mus musculus OX=10090 GN=Tm7sf2 PE=1 SV=2	3.31E+05	4.36E+05	4.26E+05	544135.97	4.77E+05	5.42E+05	1.310917224	0.037171606	0.4206427
Q99KF1	Transmembrane emp24 domain-containing protein 9 OS=Mus musculus OX=10090 GN=Tmed9 PE=1 SV=2	2.63E+03	2.60E+03	2.60E+03	3268.69	3.07E+03	3.73E+03	1.286140542	0.019508506	0.3521754
Q03059	Choline O-acetyltransferase OS=Mus musculus OX=10090 GN=Chat PE=2 SV=2	2.61E+05	3.01E+05	2.63E+05	378876.28	3.19E+05	3.63E+05	1.28547252	0.024245819	0.3689823
P97825	Jupiter microtubule associated homolog 1 OS=Mus musculus OX=10090 GN=Jpt1 PE=1 SV=3	5.20E+04	4.92E+04	5.50E+04	61404.66	6.64E+04	7.25E+04	1.282361438	0.015305275	0.3489800
Q9QUH0	Glutaredoxin-1 OS=Mus musculus OX=10090 GN=Glrx PE=1 SV=3	6.42E+05	6.83E+05	7.48E+05	836329.33	8.22E+05	9.71E+05	1.268189133	0.030741239	0.3966517
Q64338	Calcium/calmodulin-dependent 3',5'-cyclic nucleotide phosphodiesterase 1C OS=Mus musculus OX=10090 GN=Pde1c PE=1 SV=2	1.37E+05	1.33E+05	1.32E+05	176894.13	1.60E+05	1.72E+05	1.267691515	0.002621513	0.2962781
P58771	Tropomyosin alpha-1 chain OS=Mus musculus OX=10090 GN=Tpm1 PE=1 SV=1	2.19E+05	2.12E+05	2.38E+05	266455.45	2.79E+05	2.99E+05	1.25999327	0.00856	0.3224334
Q9CY45	EEF1A lysine methyltransferase 1 OS=Mus musculus OX=10090 GN=Eef1akmt1 PE=1 SV=1	3.20E+04	3.37E+04	3.62E+04	45449.07	3.72E+04	4.55E+04	1.257845767	0.044332018	0.4281674
Q8CGP7	Histone H2A type 1-K OS=Mus musculus OX=10090 GN=H2ac15 PE=1 SV=3	9.04E+06	9.26E+06	9.02E+06	11914181.25	1.09E+07	1.15E+07	1.253902325	0.001870463	0.2962781
Q9JK42	[Pyruvate dehydrogenase (acetyl-transferring)] kinase isozyme 2, mitochondrial OS=Mus musculus OX=10090 GN=Pdk2 PE=1 SV=2	4.43E+04	4.94E+04	5.14E+04	62816.77	6.00E+04	5.87E+04	1.250667892	0.007658882	0.3168862
Q3TCH7	Cullin-4A OS=Mus musculus OX=10090 GN=Cul4a PE=1 SV=1	4.88E+03	5.05E+03	5.33E+03	6118.05	6.13E+03	6.78E+03	1.246471293	0.007924882	0.3180017
O55091	Protein IMPACT OS=Mus musculus OX=10090 GN=Impact PE=1 SV=2	1.07E+05	1.14E+05	1.06E+05	143860.69	1.30E+05	1.30E+05	1.237317912	0.008058108	0.3180017
Q9CZJ1	Probable U3 small nucleolar RNA-associated protein 11 OS=Mus musculus OX=10090 GN=Utp11 PE=2 SV=1	2.62E+04	2.45E+04	2.56E+04	29157.29	3.48E+04	3.02E+04	1.234135349	0.030876297	0.396678
Q99K95	Replication termination factor 2 OS=Mus musculus OX=10090 GN=Rtf2 PE=1 SV=1	4.77E+05	4.14E+05	4.93E+05	578071.99	5.69E+05	5.60E+05	1.233579866	0.012380641	0.3480419
Q9Z2G0	Protein fem-1 homolog B OS=Mus musculus OX=10090 GN=Fem1b PE=1 SV=1	5.63E+03	7.24E+03	5.96E+03	7964.31	7.53E+03	7.71E+03	1.232444731	0.04486794	0.4294348
Q9D7B6	Isobutyryl-CoA dehydrogenase, mitochondrial OS=Mus musculus OX=10090 GN=Acad8 PE=1 SV=2	1.54E+04	1.66E+04	1.51E+04	16947.20	2.02E+04	2.06E+04	1.22618172	0.045536649	0.4306465
P53798	Squalene synthase OS=Mus musculus OX=10090 GN=Fdft1 PE=1 SV=2	1.06E+05	1.17E+05	1.22E+05	148519.65	1.36E+05	1.35E+05	1.218809972	0.017360721	0.3489800
Q91YR7	Pre-mRNA-processing factor 6 OS=Mus musculus OX=10090 GN=Prpf6 PE=1 SV=1	9.25E+04	9.84E+04	8.68E+04	122391.09	1.08E+05	1.08E+05	1.218008636	0.026321685	0.3876421
Q8BIW1	Exopolyphosphatase PRUNE1 OS=Mus musculus OX=10090 GN=Prune1 PE=1 SV=1	8.39E+04	9.92E+04	8.87E+04	112273.92	1.13E+05	1.06E+05	1.216816683	0.017660966	0.350746
P11627	Neural cell adhesion molecule L1 OS=Mus musculus OX=10090 GN=L1cam PE=1 SV=1	1.88E+05	1.95E+05	2.01E+05	240024.04	2.27E+05	2.43E+05	1.216197906	0.002532348	0.2962781
Q62351	Transferrin receptor protein 1 OS=Mus musculus OX=10090 GN=Tfrc PE=1 SV=1	6.17E+04	6.27E+04	6.52E+04	75508.89	8.14E+04	7.31E+04	1.213522588	0.007126873	0.3110762
Q99KK2	N-acylneuraminate cytidylyltransferase OS=Mus musculus OX=10090 GN=Cmas PE=1 SV=2	4.85E+05	4.62E+05	5.22E+05	576228.98	5.77E+05	6.28E+05	1.213229564	0.012699605	0.3480419
P47757	F-actin-capping protein subunit beta OS=Mus musculus OX=10090 GN=Capzb PE=1 SV=3	2.35E+05	2.57E+05	2.48E+05	295402.43	3.06E+05	2.95E+05	1.211517211	0.002150375	0.2962781
O89020	Afamin OS=Mus musculus OX=10090 GN=Afm PE=1 SV=2	9.56E+04	1.08E+05	1.08E+05	128606.68	1.19E+05	1.31E+05	1.211018992	0.018216604	0.3521754
Q91W53	Golgin subfamily A member 7 OS=Mus musculus OX=10090 GN=Golga7 PE=1 SV=1	2.05E+05	1.88E+05	2.08E+05	254134.78	2.29E+05	2.45E+05	1.21076702	0.012360902	0.3480419
P70202	Latexin OS=Mus musculus OX=10090 GN=Lxn PE=1 SV=2	1.72E+05	1.65E+05	2.06E+05	220749.75	2.22E+05	2.11E+05	1.20442583	0.047371616	0.4329233
Q8BUK6	Protein Hook homolog 3 OS=Mus musculus OX=10090 GN=Hook3 PE=1 SV=2	5.54E+05	6.32E+05	6.20E+05	755284.23	7.35E+05	6.84E+05	1.203866195	0.018777058	0.3521754
P06728	Apolipoprotein A-IV OS=Mus musculus OX=10090 GN=Apoa4 PE=1 SV=3	1.04E+06	1.03E+06	1.21E+06	1282976.42	1.34E+06	1.32E+06	1.203040027	0.020139458	0.3521754
P16054	Protein kinase C epsilon type OS=Mus musculus OX=10090 GN=Prkce PE=1 SV=1	2.92E+05	3.43E+05	3.33E+05	418908.07	3.86E+05	3.59E+05	1.202859663	0.048802634	0.4387728
P48678	Prelamin-A/C OS=Mus musculus OX=10090 GN=Lmna PE=1 SV=2	2.97E+06	2.73E+06	2.86E+06	3398229.28	3.35E+06	3.55E+06	1.202791546	0.003331783	0.2962781
Q8BJ90	Zinc finger protein 771 OS=Mus musculus OX=10090 GN=Znf771 PE=2 SV=1	6.99E+04	6.54E+04	6.74E+04	77588.87	8.20E+04	8.38E+04	1.201072574	0.003955559	0.2962781
Q62009	Periostin OS=Mus musculus OX=10090 GN=Postn PE=1 SV=2	3.72E+05	4.00E+05	4.21E+05	496148.21	4.43E+05	4.93E+05	1.200654612	0.02296913	0.3606062
Q61703	Inter-alpha-trypsin inhibitor heavy chain H2 OS=Mus musculus OX=10090 GN=Itih2 PE=1 SV=1	1.48E+05	1.76E+05	1.64E+05	192814.88	1.95E+05	1.97E+05	1.200488788	0.018119212	0.3521754
P62806	Histone H4 OS=Mus musculus OX=10090 GN=H4c1 PE=1 SV=2	3.08E+06	3.29E+06	3.15E+06	3741058.72	3.86E+06	3.82E+06	1.199488345	0.000883401	0.1949371
Q80WQ6	Inactive rhomboid protein 2 OS=Mus musculus OX=10090 GN=Rhbdf2 PE=1 SV=1	4.61E+04	4.33E+04	4.60E+04	56704.84	5.42E+04	5.14E+04	1.198969735	0.007345989	0.3137448
Q3UW53	Protein Niban 1 OS=Mus musculus OX=10090 GN=Niban1 PE=1 SV=2	4.30E+04	4.67E+04	4.39E+04	51646.76	5.34E+04	5.50E+04	1.197161171	0.003897221	0.2962781
O08688	Calpain-5 OS=Mus musculus OX=10090 GN=Capn5 PE=1 SV=1	2.00E+05	2.31E+05	2.29E+05	269535.84	2.61E+05	2.58E+05	1.193649796	0.015371714	0.3489800
Q60780	Growth arrest-specific protein 7 OS=Mus musculus OX=10090 GN=Gas7 PE=1 SV=1	2.80E+05	2.64E+05	2.76E+05	319074.69	3.02E+05	3.52E+05	1.188041391	0.029170532	0.3944086
Q9JKS5	Intracellular hyaluronan-binding protein 4 OS=Mus musculus OX=10090 GN=Habp4 PE=1 SV=2	7.63E+05	7.05E+05	7.60E+05	810549.69	9.06E+05	9.28E+05	1.187448859	0.026860438	0.3876421
Q2UY11	Collagen alpha-1(XXVIII) chain OS=Mus musculus OX=10090 GN=Col28a1 PE=2 SV=1	8.63E+05	7.79E+05	7.83E+05	947873.41	1.01E+06	9.12E+05	1.183388679	0.020091157	0.3521754
Q3TES0	IQ motif and SEC7 domain-containing protein 3 OS=Mus musculus OX=10090 GN=Iqsec3 PE=1 SV=1	2.91E+04	3.57E+04	3.33E+04	40198.42	3.73E+04	3.85E+04	1.181675366	0.047273762	0.4329233
Q3TMX7	Sulfhydryl oxidase 2 OS=Mus musculus OX=10090 GN=Qsox2 PE=1 SV=1	5.98E+04	6.11E+04	6.30E+04	68684.26	7.14E+04	7.73E+04	1.181383748	0.014542768	0.3489800
P41234	ATP-binding cassette sub-family A member 2 OS=Mus musculus OX=10090 GN=Abca2 PE=1 SV=4	1.96E+05	2.13E+05	2.11E+05	262208.05	2.40E+05	2.30E+05	1.181128148	0.026935862	0.3876421
P62849	40S ribosomal protein S24 OS=Mus musculus OX=10090 GN=Rps24 PE=1 SV=1	1.72E+06	1.72E+06	1.72E+06	2067331.21	1.89E+06	2.12E+06	1.177063034	0.012114127	0.3480419
Q924K8	Metastasis-associated protein MTA3 OS=Mus musculus OX=10090 GN=Mta3 PE=1 SV=1	3.66E+04	3.32E+04	3.32E+04	43988.30	3.93E+04	3.79E+04	1.176290247	0.048187616	0.4350027
P97863	Nuclear factor 1 B-type OS=Mus musculus OX=10090 GN=Nfib PE=1 SV=2	1.74E+05	1.46E+05	1.72E+05	200577.93	1.84E+05	1.94E+05	1.175503137	0.048874638	0.4387728
Q9Z1M0	P2X purinoceptor 7 OS=Mus musculus OX=10090 GN=P2rx7 PE=1 SV=2	4.57E+05	4.37E+05	4.14E+05	540545.90	5.12E+05	4.83E+05	1.17413988	0.021269123	0.3559661
P27661	Histone H2AX OS=Mus musculus OX=10090 GN=H2ax PE=1 SV=2	2.38E+06	2.42E+06	2.34E+06	2806331.48	2.76E+06	2.80E+06	1.171951521	0.000142301	0.100290
Q91W39	Nuclear receptor coactivator 5 OS=Mus musculus OX=10090 GN=Ncoa5 PE=1 SV=1	1.10E+05	1.15E+05	1.08E+05	128173.34	1.30E+05	1.32E+05	1.171639684	0.001541344	0.2782826
P23198	Chromobox protein homolog 3 OS=Mus musculus OX=10090 GN=Cbx3 PE=1 SV=2	2.94E+05	2.94E+05	2.98E+05	339125.44	3.45E+05	3.52E+05	1.170145053	0.000213828	0.1213319
A2A8L5	Receptor-type tyrosine-protein phosphatase F OS=Mus musculus OX=10090 GN=Ptprf PE=1 SV=1	4.66E+04	4.08E+04	4.41E+04	53599.49	5.08E+04	4.92E+04	1.168026309	0.026192396	0.3876421
Q8C0L0	Thioredoxin-related transmembrane protein 4 OS=Mus musculus OX=10090 GN=Tmx4 PE=1 SV=2	2.29E+05	2.76E+05	2.58E+05	303942.17	2.92E+05	2.95E+05	1.167199576	0.038271881	0.4247452
P18608	Non-histone chromosomal protein HMG-14 OS=Mus musculus OX=10090 GN=Hmgn1 PE=1 SV=2	5.95E+05	6.28E+05	6.11E+05	721478.21	7.03E+05	7.17E+05	1.16714374	0.000807125	0.1885824
P57722	Poly(rC)-binding protein 3 OS=Mus musculus OX=10090 GN=Pcbp3 PE=1 SV=3	4.29E+05	4.59E+05	4.88E+05	547945.65	4.98E+05	5.59E+05	1.165694046	0.038814119	0.4256777
Q9Z1J3	Cysteine desulfurase, mitochondrial OS=Mus musculus OX=10090 GN=Nfs1 PE=1 SV=3	4.47E+05	5.05E+05	4.92E+05	562291.59	5.49E+05	5.71E+05	1.165583531	0.013318825	0.3480419

B2RY56	RNA-binding protein 25 OS=Mus musculus OX=10090 GN=Rbm25 PE=1 SV=2	3.69E+05	4.16E+05	3.50E+05	454136.68	4.29E+05	4.36E+05	1.161992519	0.043860833	0.4270203
Q80X50	Ubiquitin-associated protein 2-like OS=Mus musculus OX=10090 GN=Ubap2l PE=1 SV=1	3.31E+05	3.10E+05	3.66E+05	387509.94	4.01E+05	3.81E+05	1.161333307	0.034648447	0.4129479
Q91XI1	tRNA-dihydrouridine(47) synthase [NAD(P)(+)]-like OS=Mus musculus OX=10090 GN=Dus3l PE=1 SV=1	7.10E+04	6.87E+04	7.41E+04	85893.41	8.40E+04	7.80E+04	1.159865165	0.015968646	0.3489800
O35143	ATPase inhibitor, mitochondrial OS=Mus musculus OX=10090 GN=Atp5if1 PE=1 SV=2	9.60E+04	8.75E+04	9.44E+04	103302.11	1.07E+05	1.12E+05	1.159832519	0.015406479	0.3489800
Q3THW5	Histone H2A.V OS=Mus musculus OX=10090 GN=H2az2 PE=1 SV=3	2.42E+06	2.33E+06	2.44E+06	2792188.37	2.75E+06	2.80E+06	1.159198575	0.000542127	0.158849
Q8C052	Microtubule-associated protein 1S OS=Mus musculus OX=10090 GN=Map1s PE=1 SV=2	7.66E+04	7.32E+04	8.04E+04	87207.41	8.58E+04	9.36E+04	1.158275461	0.018584766	0.3521754
Q8BL66	Early endosome antigen 1 OS=Mus musculus OX=10090 GN=Eea1 PE=1 SV=2	2.45E+05	2.77E+05	2.53E+05	309132.93	2.84E+05	3.04E+05	1.157683247	0.030376097	0.3966517
Q9CWZ3	RNA-binding protein 8A OS=Mus musculus OX=10090 GN=Rbm8a PE=1 SV=4	1.26E+05	1.35E+05	1.41E+05	161686.57	1.48E+05	1.55E+05	1.156665013	0.025385364	0.3804930
Q9CZX8	40S ribosomal protein S19 OS=Mus musculus OX=10090 GN=Rps19 PE=1 SV=3	9.31E+05	8.27E+05	9.07E+05	1026925.74	9.75E+05	1.07E+06	1.15416247	0.032312332	0.4017909
Q64331	Unconventional myosin-VI OS=Mus musculus OX=10090 GN=Myo6 PE=1 SV=1	3.49E+05	3.40E+05	3.59E+05	427184.61	3.76E+05	4.06E+05	1.15407496	0.027458225	0.3909106
P08752	Guanine nucleotide-binding protein G(i) subunit alpha-2 OS=Mus musculus OX=10090 GN=Gnai2 PE=1 SV=5	4.76E+05	4.71E+05	4.12E+05	532113.93	5.39E+05	4.97E+05	1.153822902	0.045294257	0.4304038
Q01147	Cyclic AMP-responsive element-binding protein 1 OS=Mus musculus OX=10090 GN=Creb1 PE=1 SV=2	2.54E+05	2.54E+05	2.80E+05	306168.84	3.17E+05	2.86E+05	1.15318948	0.032369866	0.4017909
Q9CZU3	Exosome RNA helicase MTR4 OS=Mus musculus OX=10090 GN=Mtrex PE=1 SV=1	4.75E+05	4.96E+05	4.90E+05	565420.72	5.45E+05	5.73E+05	1.152073581	0.002181415	0.2962781
Q8BXC6	COMM domain-containing protein 2 OS=Mus musculus OX=10090 GN=Commd2 PE=1 SV=1	3.67E+05	3.32E+05	3.65E+05	425832.43	4.09E+05	3.91E+05	1.151298344	0.024374507	0.3695249
O54901	OX-2 membrane glycoprotein OS=Mus musculus OX=10090 GN=Cd200 PE=1 SV=1	1.33E+04	1.51E+04	1.43E+04	16639.20	1.57E+04	1.67E+04	1.149240712	0.027255827	0.3894249
Q6PB93	Polypeptide N-acetylgalactosaminyltransferase 2 OS=Mus musculus OX=10090 GN=Galnt2 PE=1 SV=1	2.57E+05	2.84E+05	2.88E+05	322358.47	3.14E+05	3.16E+05	1.148795867	0.014880609	0.3489800
P31938	Dual specificity mitogen-activated protein kinase kinase 1 OS=Mus musculus OX=10090 GN=Map2k1 PE=1 SV=2	2.94E+05	2.99E+05	2.98E+05	349624.84	3.35E+05	3.39E+05	1.148746253	0.000639878	0.158849
Q5SSZ5	Tensin-3 OS=Mus musculus OX=10090 GN=Tns3 PE=1 SV=1	1.10E+05	1.12E+05	1.20E+05	125156.81	1.30E+05	1.37E+05	1.14836619	0.01992556	0.3521754
Q68ED7	CREB-regulated transcription coactivator 1 OS=Mus musculus OX=10090 GN=Crtc1 PE=1 SV=1	5.00E+04	5.26E+04	5.22E+04	61645.37	5.97E+04	5.63E+04	1.147745165	0.01192098	0.3480419
P97351	40S ribosomal protein S3a OS=Mus musculus OX=10090 GN=Rps3a PE=1 SV=3	6.02E+05	6.53E+05	6.23E+05	703257.02	6.98E+05	7.53E+05	1.147176998	0.015718684	0.3489800
Q8VEA4	Mitochondrial intermembrane space import and assembly protein 40 OS=Mus musculus OX=10090 GN=Chchd4 PE=1 SV=1	8.24E+04	8.11E+04	8.52E+04	90585.88	9.39E+04	1.01E+05	1.146813021	0.019435176	0.3521754
Q6PEE2	CBP80/20-dependent translation initiation factor OS=Mus musculus OX=10090 GN=Ctif PE=1 SV=2	1.90E+05	1.94E+05	2.10E+05	230169.50	2.17E+05	2.34E+05	1.146372262	0.02122577	0.3559661
P70377	Fibroblast growth factor 13 OS=Mus musculus OX=10090 GN=Fgf13 PE=1 SV=2	4.03E+05	4.05E+05	4.02E+05	484042.78	4.64E+05	4.37E+05	1.145488894	0.012817797	0.3480419
Q8BHD7	Polypyrimidine tract-binding protein 3 OS=Mus musculus OX=10090 GN=Ptbp3 PE=1 SV=1	4.20E+05	4.50E+05	4.29E+05	524001.54	4.85E+05	4.79E+05	1.145249685	0.019611152	0.3521754
Q3UYC0	Protein phosphatase 1H OS=Mus musculus OX=10090 GN=Ppm1h PE=1 SV=1	7.20E+04	7.43E+04	7.31E+04	86576.98	8.02E+04	8.41E+04	1.144077215	0.005764365	0.3029392
P10605	Cathepsin B OS=Mus musculus OX=10090 GN=Ctsb PE=1 SV=2	4.50E+05	4.30E+05	4.46E+05	470940.16	5.04E+05	5.40E+05	1.142780952	0.039617045	0.4270203
P62748	Hippocalcin-like protein 1 OS=Mus musculus OX=10090 GN=Hpcal1 PE=1 SV=2	4.52E+05	4.57E+05	4.54E+05	525022.77	5.16E+05	5.16E+05	1.142751186	3.96378E-05	0.0936477
Q921F2	TAR DNA-binding protein 43 OS=Mus musculus OX=10090 GN=Tardbp PE=1 SV=1	7.46E+05	7.39E+05	7.78E+05	819889.94	8.46E+05	9.19E+05	1.142388426	0.028490196	0.3937279
Q9CQF0	39S ribosomal protein L11, mitochondrial OS=Mus musculus OX=10090 GN=Mrpl11 PE=1 SV=1	2.64E+05	2.73E+05	2.95E+05	321656.62	3.31E+05	2.98E+05	1.141743838	0.043901553	0.4270203
Q9WTX2	Interferon-inducible double-stranded RNA-dependent protein kinase activator A OS=Mus musculus OX=10090 GN=Prkra PE=1 SV=1	3.04E+05	3.49E+05	3.28E+05	386988.93	3.56E+05	3.77E+05	1.141210155	0.043089348	0.4270203
Q60520	Paired amphipathic helix protein Sin3a OS=Mus musculus OX=10090 GN=Sin3a PE=1 SV=3	2.44E+05	2.53E+05	2.56E+05	298848.82	2.84E+05	2.76E+05	1.140819085	0.009557688	0.3280756
Q8VBT0	Thioredoxin-related transmembrane protein 1 OS=Mus musculus OX=10090 GN=Tmx1 PE=1 SV=1	5.56E+04	5.50E+04	5.42E+04	63523.79	5.99E+04	6.41E+04	1.137828761	0.005646781	0.3029392
Q3TQB2	FAD-dependent oxidoreductase domain-containing protein 1 OS=Mus musculus OX=10090 GN=Foxred1 PE=1 SV=1	1.00E+05	1.09E+05	1.02E+05	122093.28	1.13E+05	1.19E+05	1.13758199	0.01709961	0.3489800
Q7TNC4	Putative RNA-binding protein Luc7-like 2 OS=Mus musculus OX=10090 GN=Luc7l2 PE=1 SV=1	6.78E+04	6.73E+04	6.35E+04	76175.06	7.56E+04	7.41E+04	1.137387929	0.003766758	0.2962781
P55288	Cadherin-11 OS=Mus musculus OX=10090 GN=Cdh11 PE=1 SV=1	2.96E+05	3.21E+05	3.30E+05	360380.08	3.60E+05	3.56E+05	1.136437388	0.013813481	0.3489800
Q69ZQ2	Pre-mRNA-splicing factor ISY1 homolog OS=Mus musculus OX=10090 GN=Isy1 PE=1 SV=2	5.51E+04	6.04E+04	6.26E+04	68282.81	7.00E+04	6.41E+04	1.135708485	0.046053017	0.4324410
Q9Z204	Heterogeneous nuclear ribonucleoproteins C1/C2 OS=Mus musculus OX=10090 GN=Hnrnpc PE=1 SV=1	3.58E+05	3.68E+05	3.50E+05	401534.02	4.01E+05	4.18E+05	1.133647331	0.003477905	0.2962781
Q8CJG0	Protein argonaute-2 OS=Mus musculus OX=10090 GN=Ago2 PE=1 SV=3	8.11E+04	8.50E+04	8.88E+04	97876.04	9.99E+04	9.06E+04	1.131175045	0.036099384	0.4144125
Q9DCS2	Methyltransferase-like 26 OS=Mus musculus OX=10090 GN=Mettl26 PE=1 SV=1	9.65E+04	1.02E+05	1.00E+05	106115.11	1.11E+05	1.20E+05	1.130813886	0.038603482	0.4247452
Q9D1J3	SAP domain-containing ribonucleoprotein OS=Mus musculus OX=10090 GN=Sarnp PE=1 SV=3	4.53E+05	4.44E+05	4.31E+05	530815.51	4.87E+05	4.81E+05	1.128313299	0.029118416	0.3944086
Q9QZE7	Translin-associated protein X OS=Mus musculus OX=10090 GN=Tsnax PE=1 SV=1	2.95E+05	3.00E+05	2.72E+05	341258.36	3.21E+05	3.17E+05	1.127961897	0.032503176	0.4020170
P80314	T-complex protein 1 subunit beta OS=Mus musculus OX=10090 GN=Cct2 PE=1 SV=4	1.21E+06	1.17E+06	1.23E+06	1338773.42	1.35E+06	1.38E+06	1.127910177	0.002388478	0.2962781
Q8JZK9	Hydroxymethylglutaryl-CoA synthase, cytoplasmic OS=Mus musculus OX=10090 GN=Hmgcs1 PE=1 SV=1	7.44E+05	7.31E+05	7.00E+05	826826.06	8.49E+05	7.77E+05	1.127552676	0.020228263	0.3521754
P62307	Small nuclear ribonucleoprotein F OS=Mus musculus OX=10090 GN=Snrpf PE=1 SV=1	6.64E+04	6.78E+04	6.48E+04	77574.01	7.33E+04	7.30E+04	1.124973145	0.008246269	0.3180017
Q9CWU9	Nucleoporin Nup37 OS=Mus musculus OX=10090 GN=Nup37 PE=1 SV=2	7.11E+04	7.50E+04	7.12E+04	85239.51	7.90E+04	8.03E+04	1.12468413	0.017146486	0.3489800
Q7TQG1	Pleckstrin homology domain-containing family A member 6 OS=Mus musculus OX=10090 GN=Plekha6 PE=1 SV=1	1.09E+05	1.12E+05	1.11E+05	117203.70	1.32E+05	1.23E+05	1.124383547	0.035390298	0.4129479
Q9D5R3	Centrosomal protein of 83 kDa OS=Mus musculus OX=10090 GN=Cep83 PE=1 SV=2	8.85E+03	1.01E+04	9.92E+03	10736.87	1.05E+04	1.11E+04	1.12298303	0.047288305	0.4329233
Q9D8E6	60S ribosomal protein L4 OS=Mus musculus OX=10090 GN=Rpl4 PE=1 SV=3	1.08E+06	1.19E+06	1.18E+06	1231187.10	1.29E+06	1.34E+06	1.121809756	0.03960201	0.4270203
Q9QYA2	Mitochondrial import receptor subunit TOM40 homolog OS=Mus musculus OX=10090 GN=Tomm40 PE=1 SV=3	1.30E+05	1.37E+05	1.31E+05	153492.85	1.46E+05	1.47E+05	1.121417092	0.005796421	0.3029392
Q99JI4	26S proteasome non-ATPase regulatory subunit 6 OS=Mus musculus OX=10090 GN=Psmd6 PE=1 SV=1	8.68E+04	8.29E+04	8.90E+04	94789.62	9.54E+04	9.97E+04	1.120407011	0.011456175	0.3480419
Q9QWY8	Arf-GAP with SH3 domain, ANK repeat and PH domain-containing protein 1 OS=Mus musculus OX=10090 GN=Asap1 PE=1 SV=2	1.81E+05	1.71E+05	1.87E+05	206028.50	1.90E+05	2.08E+05	1.118878626	0.044070062	0.4270203
Q80UZ2	Protein SDA1 homolog OS=Mus musculus OX=10090 GN=Sdad1 PE=1 SV=1	4.42E+05	4.06E+05	4.43E+05	486338.51	4.85E+05	4.73E+05	1.118873667	0.016631835	0.3489800
Q8BUY8	G-protein coupled receptor-associated sorting protein 2 OS=Mus musculus OX=10090 GN=Gprasp2 PE=1 SV=2	1.13E+05	1.09E+05	1.13E+05	128756.72	1.24E+05	1.22E+05	1.118420995	0.004579497	0.2962781
O35887	Calumenin OS=Mus musculus OX=10090 GN=Calu PE=1 SV=1	2.04E+05	2.09E+05	1.99E+05	227352.32	2.23E+05	2.32E+05	1.117347334	0.003360716	0.2962781
P63280	SUMO-conjugating enzyme UBC9 OS=Mus musculus OX=10090 GN=Ube2i PE=1 SV=1	5.05E+05	4.83E+05	4.91E+05	569207.74	5.25E+05	5.58E+05	1.11711275	0.017396286	0.3489800
Q9R0P4	Small acidic protein OS=Mus musculus OX=10090 GN=Smap PE=1 SV=1	9.02E+04	9.33E+04	8.93E+04	103045.91	9.50E+04	1.07E+05	1.11659014	0.044015518	0.4270203
Q91W45	Polyadenylate-binding protein-interacting protein 2B OS=Mus musculus OX=10090 GN=Paip2b PE=1 SV=2	2.21E+04	2.22E+04	2.14E+04	24890.58	2.38E+04	2.47E+04	1.116036982	0.003467027	0.2962781
Q8CFI0	E3 ubiquitin-protein ligase NEDD4-like OS=Mus musculus OX=10090 GN=Nedd4l PE=1 SV=2	1.89E+05	1.90E+05	1.87E+05	211900.96	2.02E+05	2.18E+05	1.115250738	0.009581262	0.3280756
Q6PGL7	WASH complex subunit 2 OS=Mus musculus OX=10090 GN=Washc2 PE=1 SV=1	1.50E+05	1.48E+05	1.53E+05	166778.54	1.75E+05	1.61E+05	1.114893578	0.014255471	0.3489800
Q8VBT9	Tether containing UBX domain for GLUT4 OS=Mus musculus OX=10090 GN=Aspscr1 PE=1 SV=1	1.42E+05	1.47E+05	1.55E+05	170528.96	1.60E+05	1.63E+05	1.112445968	0.024962743	0.3770038
Q99LP6	GrpE protein homolog 1, mitochondrial OS=Mus musculus OX=10090 GN=Grpel1 PE=1 SV=1	2.35E+05	2.44E+05	2.24E+05	261332.59	2.64E+05	2.57E+05	1.112222544	0.011591774	0.3480419
Q91ZW3	SWI/SNF-related matrix-associated actin-dependent regulator of chromatin subfamily A member 5 OS=Mus musculus OX=10090 GN=Smarca5 PE=1 SV=1	3.48E+05	3.79E+05	3.64E+05	403974.41	3.96E+05	4.13E+05	1.111779521	0.016501737	0.3489800
Q8BJL0	SWI/SNF-related matrix-associated actin-dependent regulator of chromatin subfamily A-like protein 1 OS=Mus musculus OX=10090 GN=Smarcal1 PE=1 SV=1	4.10E+05	4.19E+05	4.25E+05	472616.93	4.76E+05	4.45E+05	1.111602911	0.012958782	0.3480419
Q9D6Z1	Nucleolar protein 56 OS=Mus musculus OX=10090 GN=Nop56 PE=1 SV=2	6.83E+05	6.90E+05	7.23E+05	768198.71	7.78E+05	7.82E+05	1.110980899	0.004014942	0.2962781
Q3UHB1	5'-nucleotidase domain-containing protein 3 OS=Mus musculus OX=10090 GN=Nt5dc3 PE=1 SV=1	8.59E+05	9.15E+05	9.02E+05	1024292.41	1.01E+06	9.38E+05	1.110484816	0.035258531	0.4129479
Q9Z1D1	Eukaryotic translation initiation factor 3 subunit G OS=Mus musculus OX=10090 GN=Eif3g PE=1 SV=2	5.85E+05	5.61E+05	5.84E+05	653572.29	6.43E+05	6.25E+05	1.110079069	0.005264418	0.3029392
A2ASZ8	Calcium-binding mitochondrial carrier protein SCaMC-2 OS=Mus musculus OX=10090 GN=Slc25a25 PE=1 SV=1	2.04E+05	1.98E+05	1.97E+05	225439.28	2.27E+05	2.12E+05	1.109065546	0.016202248	0.3489800
P26645	Myristoylated alanine-rich C-kinase substrate OS=Mus musculus OX=10090 GN=Marcks PE=1 SV=2	3.98E+05	3.92E+05	4.05E+05	456667.32	4.17E+05	4.49E+05	1.107224095	0.026818485	0.3876421
P12023	Amyloid-beta A4 protein OS=Mus musculus OX=10090 GN=App PE=1 SV=3	3.03E+05	2.95E+05	2.94E+05	331000.57	3.16E+05	3.41E+05	1.106780519	0.01545732	0.3489800
B9EJ86	Oxysterol-binding protein-related protein 8 OS=Mus musculus OX=10090 GN=Osbpl8 PE=1 SV=1	1.11E+05	1.02E+05	1.08E+05	121984.94	1.15E+05	1.18E+05	1.105566267	0.034618755	0.4129479
Q9CZU6	Citrate synthase, mitochondrial OS=Mus musculus OX=10090 GN=Cs PE=1 SV=1	1.60E+06	1.54E+06	1.64E+06	1823769.06	1.73E+06	1.73E+06	1.105358934	0.016762012	0.3489800
Q8K215	LYR motif-containing protein 4 OS=Mus musculus OX=10090 GN=Lyrm4 PE=1 SV=1	6.00E+05	5.97E+05	5.48E+05	634533.31	6.45E+05	6.47E+05	1.104290829	0.024089971	0.3680206

Q922P9	Putative oxidoreductase GLYR1 OS=Mus musculus OX=10090 GN=Glyr1 PE=1 SV=1	3.40E+05	3.25E+05	3.43E+05	369043.38	3.58E+05	3.83E+05	1.10170727	0.020333792	0.3521754
P05132	cAMP-dependent protein kinase catalytic subunit alpha OS=Mus musculus OX=10090 GN=Prkaca PE=1 SV=3	3.57E+05	3.48E+05	3.37E+05	383238.58	3.84E+05	3.81E+05	1.101522908	0.003538159	0.2962781
Q99PV0	Pre-mRNA-processing-splicing factor 8 OS=Mus musculus OX=10090 GN=Prpf8 PE=1 SV=2	3.99E+05	4.16E+05	4.03E+05	460825.22	4.41E+05	4.39E+05	1.101047358	0.00884825	0.3224334
Q8VH51	RNA-binding protein 39 OS=Mus musculus OX=10090 GN=Rbm39 PE=1 SV=2	8.00E+05	7.79E+05	7.69E+05	861921.90	8.31E+05	8.86E+05	1.09876709	0.013212906	0.3480419
Q99N96	39S ribosomal protein L1, mitochondrial OS=Mus musculus OX=10090 GN=Mrpl1 PE=1 SV=2	5.33E+04	5.63E+04	5.30E+04	60639.14	5.83E+04	5.98E+04	1.09857744	0.013554564	0.3489800
P63328	Serine/threonine-protein phosphatase 2B catalytic subunit alpha isoform OS=Mus musculus OX=10090 GN=Ppp3ca PE=1 SV=1	1.98E+05	2.02E+05	2.10E+05	232498.06	2.21E+05	2.17E+05	1.098358595	0.026898677	0.3876421
P84244	Histone H3.3 OS=Mus musculus OX=10090 GN=H3-3a PE=1 SV=2	4.01E+05	3.93E+05	3.75E+05	428596.31	4.43E+05	4.12E+05	1.097915842	0.032792929	0.4020170
P51410	60S ribosomal protein L9 OS=Mus musculus OX=10090 GN=Rpl9 PE=1 SV=2	3.52E+05	3.75E+05	3.76E+05	394655.39	3.97E+05	4.19E+05	1.097810293	0.029914368	0.396241
P28658	Ataxin-10 OS=Mus musculus OX=10090 GN=Atxn10 PE=1 SV=2	4.88E+05	5.02E+05	5.13E+05	575736.85	5.45E+05	5.29E+05	1.097539208	0.035436454	0.4129479
Q9CQW0	ER membrane protein complex subunit 6 OS=Mus musculus OX=10090 GN=Emc6 PE=1 SV=1	8.64E+04	8.92E+04	9.42E+04	98351.47	9.82E+04	9.91E+04	1.09603933	0.019323428	0.3521754
Q9Z2A5	Arginyl-tRNA--protein transferase 1 OS=Mus musculus OX=10090 GN=Ate1 PE=1 SV=2	3.19E+04	3.31E+04	3.45E+04	35985.28	3.78E+04	3.51E+04	1.094213551	0.046954595	0.4329233
Q91VM5	RNA binding motif protein, X-linked-like-1 OS=Mus musculus OX=10090 GN=Rbmxl1 PE=2 SV=1	1.29E+05	1.29E+05	1.27E+05	144826.49	1.37E+05	1.39E+05	1.093914626	0.009468072	0.3280756
P55302	Alpha-2-macroglobulin receptor-associated protein OS=Mus musculus OX=10090 GN=Lrpap1 PE=1 SV=1	3.74E+05	4.10E+05	3.75E+05	412326.85	4.27E+05	4.28E+05	1.093785067	0.046924643	0.4329233
Q9EP89	Serine beta-lactamase-like protein LACTB, mitochondrial OS=Mus musculus OX=10090 GN=Lactb PE=1 SV=1	2.08E+05	2.22E+05	2.16E+05	246835.17	2.32E+05	2.27E+05	1.093633552	0.049448936	0.4390325
Q8BTV2	Cleavage and polyadenylation specificity factor subunit 7 OS=Mus musculus OX=10090 GN=Cpsf7 PE=1 SV=2	1.74E+05	1.74E+05	1.61E+05	187392.54	1.83E+05	1.87E+05	1.093532257	0.023351407	0.3637325
O35841	Apoptosis inhibitor 5 OS=Mus musculus OX=10090 GN=Api5 PE=1 SV=2	7.25E+05	7.58E+05	7.96E+05	840749.02	8.39E+05	8.10E+05	1.093079244	0.036373663	0.4158599
D3Z7P3	Glutaminase kidney isoform, mitochondrial OS=Mus musculus OX=10090 GN=Gls PE=1 SV=1	7.96E+05	8.37E+05	7.99E+05	909579.95	8.63E+05	8.86E+05	1.092963654	0.015974515	0.3489800
Q8R2Z5	von Willebrand factor A domain-containing protein 1 OS=Mus musculus OX=10090 GN=Vwa1 PE=1 SV=1	8.12E+05	8.86E+05	8.46E+05	906157.45	9.49E+05	9.25E+05	1.092526796	0.032783211	0.4020170
Q9WU40	Inner nuclear membrane protein Man1 OS=Mus musculus OX=10090 GN=Lemd3 PE=1 SV=2	2.88E+05	2.92E+05	2.84E+05	321441.14	3.20E+05	3.02E+05	1.092394077	0.015847485	0.3489800
Q9D4H1	Exocyst complex component 2 OS=Mus musculus OX=10090 GN=Exoc2 PE=1 SV=1	1.75E+05	1.79E+05	1.85E+05	193716.17	1.94E+05	2.02E+05	1.092221706	0.015805025	0.3489800
P15379	CD44 antigen OS=Mus musculus OX=10090 GN=Cd44 PE=1 SV=3	5.54E+03	5.17E+03	5.61E+03	6118.72	5.87E+03	5.81E+03	1.091182853	0.039927644	0.4270203
Q8K440	ABC-type organic anion transporter ABCA8B OS=Mus musculus OX=10090 GN=Abca8b PE=1 SV=2	2.56E+05	2.56E+05	2.53E+05	282159.68	2.71E+05	2.81E+05	1.090965426	0.002861395	0.2962781
Q9R0P6	Signal peptidase complex catalytic subunit SEC11A OS=Mus musculus OX=10090 GN=Sec11a PE=1 SV=1	3.76E+04	3.77E+04	3.60E+04	41177.93	3.91E+04	4.09E+04	1.089250436	0.017320244	0.3489800
P08775	DNA-directed RNA polymerase II subunit RPB1 OS=Mus musculus OX=10090 GN=Polr2a PE=1 SV=3	1.06E+05	1.07E+05	1.04E+05	118416.65	1.18E+05	1.09E+05	1.088735255	0.042401941	0.4270203
Q8BTV1	Tumor suppressor candidate 3 OS=Mus musculus OX=10090 GN=Tusc3 PE=1 SV=1	4.35E+05	4.24E+05	4.49E+05	488977.21	4.53E+05	4.80E+05	1.08738041	0.042163114	0.4270203
P41216	Long-chain-fatty-acid--CoA ligase 1 OS=Mus musculus OX=10090 GN=Acsl1 PE=1 SV=2	2.24E+05	2.37E+05	2.33E+05	243179.59	2.59E+05	2.52E+05	1.086711777	0.025228169	0.3795692
Q9Z1M8	Protein Red OS=Mus musculus OX=10090 GN=Ik PE=1 SV=2	1.52E+05	1.58E+05	1.55E+05	172385.40	1.62E+05	1.71E+05	1.086489603	0.022057143	0.3561421
Q99MR6	Serrate RNA effector molecule homolog OS=Mus musculus OX=10090 GN=Srrt PE=1 SV=1	3.85E+05	4.15E+05	4.08E+05	447901.26	4.30E+05	4.34E+05	1.08616049	0.030427402	0.3966517
P54818	Galactocerebrosidase OS=Mus musculus OX=10090 GN=Galc PE=1 SV=2	2.57E+05	2.44E+05	2.44E+05	270363.55	2.69E+05	2.69E+05	1.085423672	0.007652264	0.3168862
Q8C3F2	Constitutive coactivator of PPAR-gamma-like protein 2 OS=Mus musculus OX=10090 GN=Fam120c PE=1 SV=3	3.88E+05	4.11E+05	4.27E+05	445836.75	4.49E+05	4.35E+05	1.084398399	0.046883707	0.4329233
P15331	Peripherin OS=Mus musculus OX=10090 GN=Prph PE=1 SV=2	9.39E+06	9.54E+06	9.74E+06	10762923.89	1.02E+07	1.01E+07	1.083395151	0.026733095	0.3876421
Q9D0W5	Peptidyl-prolyl cis-trans isomerase-like 1 OS=Mus musculus OX=10090 GN=Ppil1 PE=1 SV=1	2.58E+05	2.63E+05	2.48E+05	283588.89	2.81E+05	2.69E+05	1.082538868	0.028706356	0.3937279
P61358	60S ribosomal protein L27 OS=Mus musculus OX=10090 GN=Rpl27 PE=1 SV=2	9.11E+05	9.20E+05	9.02E+05	1006503.83	9.87E+05	9.65E+05	1.082281117	0.004703608	0.2962781
Q3U3F9	Probable G-protein coupled receptor 160 OS=Mus musculus OX=10090 GN=Gpr160 PE=2 SV=1	1.39E+05	1.38E+05	1.40E+05	151940.49	1.51E+05	1.49E+05	1.082175993	0.000365378	0.158849
Q9JIX8	Apoptotic chromatin condensation inducer in the nucleus OS=Mus musculus OX=10090 GN=Acin1 PE=1 SV=3	1.76E+05	1.88E+05	1.88E+05	196744.39	2.00E+05	2.00E+05	1.082123418	0.020900106	0.3559661
P54731	FAS-associated factor 1 OS=Mus musculus OX=10090 GN=Faf1 PE=1 SV=2	1.38E+05	1.45E+05	1.46E+05	151305.70	1.58E+05	1.55E+05	1.081851133	0.017884269	0.351664
Q9ESN6	Tripartite motif-containing protein 2 OS=Mus musculus OX=10090 GN=Trim2 PE=1 SV=1	3.96E+05	3.75E+05	4.12E+05	430306.41	4.25E+05	4.24E+05	1.081726776	0.042508685	0.4270203
P40124	Adenylyl cyclase-associated protein 1 OS=Mus musculus OX=10090 GN=Cap1 PE=1 SV=4	1.12E+06	1.14E+06	1.16E+06	1260730.51	1.24E+06	1.20E+06	1.08158546	0.014218322	0.3489800
Q9CQR2	40S ribosomal protein S21 OS=Mus musculus OX=10090 GN=Rps21 PE=1 SV=1	2.07E+05	2.00E+05	2.18E+05	224601.59	2.23E+05	2.28E+05	1.081517586	0.030653062	0.3966517
Q3UHD6	Sorting nexin-27 OS=Mus musculus OX=10090 GN=Snx27 PE=1 SV=2	3.03E+05	3.05E+05	3.11E+05	331304.48	3.24E+05	3.36E+05	1.080336598	0.004164583	0.2962781
Q9WTL7	Acyl-protein thioesterase 2 OS=Mus musculus OX=10090 GN=Lypla2 PE=1 SV=1	1.47E+05	1.40E+05	1.37E+05	151419.04	1.50E+05	1.58E+05	1.079251137	0.042487149	0.4270203
Q8CI51	PDZ and LIM domain protein 5 OS=Mus musculus OX=10090 GN=Pdlim5 PE=1 SV=4	2.01E+05	2.00E+05	1.95E+05	218801.61	2.08E+05	2.15E+05	1.078426964	0.012580556	0.3480419
P49312	Heterogeneous nuclear ribonucleoprotein A1 OS=Mus musculus OX=10090 GN=Hnrnpa1 PE=1 SV=2	1.18E+06	1.19E+06	1.21E+06	1283934.70	1.30E+06	1.27E+06	1.07679245	0.001335502	0.2585545
Q9D0J8	Parathymosin OS=Mus musculus OX=10090 GN=Ptms PE=1 SV=3	1.79E+06	1.73E+06	1.84E+06	1932175.05	1.86E+06	1.98E+06	1.076601621	0.042444245	0.4270203
Q62077	1-phosphatidylinositol 4,5-bisphosphate phosphodiesterase gamma-1 OS=Mus musculus OX=10090 GN=Plcg1 PE=1 SV=2	1.71E+05	1.69E+05	1.61E+05	177116.17	1.82E+05	1.80E+05	1.07565733	0.021532826	0.3559661
Q6P5F9	Exportin-1 OS=Mus musculus OX=10090 GN=Xpo1 PE=1 SV=1	1.97E+05	1.95E+05	1.88E+05	209917.58	2.09E+05	2.04E+05	1.074436281	0.008817877	0.3224334
Q3TWW8	Serine/arginine-rich splicing factor 6 OS=Mus musculus OX=10090 GN=Srsf6 PE=1 SV=1	1.94E+05	2.07E+05	2.09E+05	216503.69	2.19E+05	2.19E+05	1.074262042	0.035675434	0.4129681
Q62376	U1 small nuclear ribonucleoprotein 70 kDa OS=Mus musculus OX=10090 GN=Snrnp70 PE=1 SV=2	1.68E+05	1.79E+05	1.78E+05	189612.34	1.91E+05	1.82E+05	1.073119438	0.04944747	0.4390325
Q91WG7	Diacylglycerol kinase gamma OS=Mus musculus OX=10090 GN=Dgkg PE=1 SV=1	1.77E+05	1.80E+05	1.81E+05	188229.90	1.91E+05	1.97E+05	1.071517496	0.010047027	0.340681
P70232	Neural cell adhesion molecule L1-like protein OS=Mus musculus OX=10090 GN=Chl1 PE=1 SV=2	1.08E+05	1.15E+05	1.07E+05	118411.28	1.17E+05	1.18E+05	1.07082456	0.047095384	0.4329233
P97822	Acidic leucine-rich nuclear phosphoprotein 32 family member E OS=Mus musculus OX=10090 GN=Anp32e PE=1 SV=2	3.96E+05	3.84E+05	3.81E+05	406793.86	4.12E+05	4.26E+05	1.070657646	0.02041034	0.3521754
A3KMP2	Tetratricopeptide repeat protein 38 OS=Mus musculus OX=10090 GN=Ttc38 PE=1 SV=2	1.15E+05	1.07E+05	1.11E+05	119994.44	1.17E+05	1.19E+05	1.066902506	0.038561895	0.4247452
O88958	Glucosamine-6-phosphate isomerase 1 OS=Mus musculus OX=10090 GN=Gnpda1 PE=1 SV=3	1.41E+05	1.34E+05	1.41E+05	145026.90	1.47E+05	1.52E+05	1.066611817	0.037496954	0.4225000
Q8C167	Prolyl endopeptidase-like OS=Mus musculus OX=10090 GN=Prepl PE=1 SV=1	8.10E+05	8.14E+05	8.21E+05	878910.13	8.81E+05	8.41E+05	1.063581471	0.018734405	0.3521754
P70372	ELAV-like protein 1 OS=Mus musculus OX=10090 GN=Elavl1 PE=1 SV=2	4.80E+05	4.85E+05	5.00E+05	509564.83	5.18E+05	5.31E+05	1.063451859	0.02368511	0.3646405
Q9D554	Splicing factor 3A subunit 3 OS=Mus musculus OX=10090 GN=Sf3a3 PE=1 SV=2	4.13E+05	4.13E+05	4.20E+05	452669.33	4.25E+05	4.45E+05	1.062151838	0.041578441	0.4270203
Q3TYX3	SET and MYND domain-containing protein 5 OS=Mus musculus OX=10090 GN=Smyd5 PE=1 SV=2	9.86E+03	1.01E+04	1.02E+04	10937.28	1.05E+04	1.05E+04	1.059091475	0.02916863	0.3944086
Q8BZZ3	NEDD4-like E3 ubiquitin-protein ligase WWP1 OS=Mus musculus OX=10090 GN=Wwp1 PE=1 SV=2	1.55E+05	1.55E+05	1.46E+05	161792.87	1.60E+05	1.59E+05	1.057469784	0.049741542	0.4400298
Q8C7E7	Starch-binding domain-containing protein 1 OS=Mus musculus OX=10090 GN=Stbd1 PE=1 SV=1	1.08E+05	1.09E+05	1.11E+05	112868.78	1.17E+05	1.17E+05	1.0567535	0.020298094	0.3521754
Q91W90	Thioredoxin domain-containing protein 5 OS=Mus musculus OX=10090 GN=Txndc5 PE=1 SV=2	3.36E+05	3.41E+05	3.44E+05	366314.91	3.56E+05	3.56E+05	1.056488978	0.01045893	0.3480419
Q99L88	Beta-1-syntrophin OS=Mus musculus OX=10090 GN=Sntb1 PE=1 SV=4	8.06E+05	8.04E+05	8.41E+05	870607.51	8.57E+05	8.60E+05	1.055585722	0.023944957	0.3672176
O08579	Emerin OS=Mus musculus OX=10090 GN=Emd PE=1 SV=1	1.10E+05	1.14E+05	1.12E+05	120465.57	1.16E+05	1.19E+05	1.055427918	0.028086844	0.3937240
P62908	40S ribosomal protein S3 OS=Mus musculus OX=10090 GN=Rps3 PE=1 SV=1	7.65E+05	7.99E+05	7.78E+05	832700.56	8.08E+05	8.29E+05	1.054950868	0.025611072	0.3824330
O35855	Branched-chain-amino-acid aminotransferase, mitochondrial OS=Mus musculus OX=10090 GN=Bcat2 PE=1 SV=2	7.21E+05	7.14E+05	7.24E+05	757514.88	7.69E+05	7.52E+05	1.05468855	0.002389608	0.2962781
O55131	Septin-7 OS=Mus musculus OX=10090 GN=Septin7 PE=1 SV=1	7.24E+05	7.31E+05	7.02E+05	762269.58	7.53E+05	7.60E+05	1.054454338	0.01246686	0.3480419
Q6ZWV7	60S ribosomal protein L35 OS=Mus musculus OX=10090 GN=Rpl35 PE=1 SV=1	2.66E+06	2.74E+06	2.73E+06	2862575.70	2.79E+06	2.91E+06	1.05250492	0.036569085	0.4161960
Q62093	Serine/arginine-rich splicing factor 2 OS=Mus musculus OX=10090 GN=Srsf2 PE=1 SV=4	6.68E+04	6.62E+04	6.93E+04	71803.18	7.03E+04	7.07E+04	1.051860323	0.027798396	0.3937240
Q6P2K6	Serine/threonine-protein phosphatase 4 regulatory subunit 3A OS=Mus musculus OX=10090 GN=Ppp4r3a PE=1 SV=1	8.36E+04	8.27E+04	8.39E+04	87481.30	8.88E+04	8.58E+04	1.047659418	0.01283066	0.3480419
P29758	Ornithine aminotransferase, mitochondrial OS=Mus musculus OX=10090 GN=Oat PE=1 SV=1	8.23E+05	8.31E+05	8.19E+05	879905.90	8.50E+05	8.61E+05	1.047503829	0.01451249	0.3489800
P47811	Mitogen-activated protein kinase 14 OS=Mus musculus OX=10090 GN=Mapk14 PE=1 SV=3	2.42E+05	2.48E+05	2.38E+05	252104.73	2.53E+05	2.56E+05	1.045018373	0.021547831	0.3559661
Q3UHJ0	AP2-associated protein kinase 1 OS=Mus musculus OX=10090 GN=Aak1 PE=1 SV=2	5.81E+05	5.76E+05	5.82E+05	610429.39	5.92E+05	6.11E+05	1.043093	0.016566425	0.3489800
Q8K183	Pyridoxal kinase OS=Mus musculus OX=10090 GN=Pdxk PE=1 SV=1	2.14E+05	2.16E+05	2.24E+05	227873.10	2.27E+05	2.27E+05	1.043028054	0.049035712	0.4387728

Q9D8M4	60S ribosomal protein L7-like 1 OS=Mus musculus OX=10090 GN=Rpl7l1 PE=1 SV=1	1.88E+05	1.90E+05	1.94E+05	194627.16	1.99E+05	1.99E+05	1.037793451	0.034994804	0.4129479
P63276	40S ribosomal protein S17 OS=Mus musculus OX=10090 GN=Rps17 PE=1 SV=2	2.41E+06	2.44E+06	2.43E+06	2496285.52	2.45E+06	2.47E+06	1.01834807	0.041443992	0.4270203
Q9WUM4	Coronin-1C OS=Mus musculus OX=10090 GN=Coro1c PE=1 SV=2	1.25E+05	1.25E+05	1.24E+05	125541.74	1.27E+05	1.28E+05	1.016433186	0.035765624	0.4129681
**Downregulated genes (235)**										
Q61543	Golgi apparatus protein 1 OS=Mus musculus OX=10090 GN=Glg1 PE=1 SV=1	5.72E+05	5.72E+05	5.59E+05	551514.81	5.50E+05	5.31E+05	0.958125805	0.042133669	0.4270203
Q99MN9	Propionyl-CoA carboxylase beta chain, mitochondrial OS=Mus musculus OX=10090 GN=Pccb PE=1 SV=2	5.65E+05	5.61E+05	5.53E+05	534959.48	5.22E+05	5.49E+05	0.956148886	0.045861613	0.4324410
Q62384	Zinc finger protein ZPR1 OS=Mus musculus OX=10090 GN=Zpr1 PE=1 SV=1	1.43E+05	1.47E+05	1.47E+05	142395.80	1.40E+05	1.35E+05	0.954135413	0.049518277	0.4390325
P54797	Transport and Golgi organization 2 homolog OS=Mus musculus OX=10090 GN=Tango2 PE=1 SV=1	1.13E+05	1.15E+05	1.13E+05	109647.38	1.10E+05	1.05E+05	0.952982059	0.03306492	0.4041041
O54916	RalBP1-associated Eps domain-containing protein 1 OS=Mus musculus OX=10090 GN=Reps1 PE=1 SV=2	8.94E+04	8.50E+04	8.54E+04	83170.73	8.23E+04	8.16E+04	0.950587166	0.044412132	0.4281674
Q8BHS6	Armadillo repeat-containing X-linked protein 3 OS=Mus musculus OX=10090 GN=Armcx3 PE=1 SV=1	1.40E+05	1.42E+05	1.44E+05	138738.34	1.34E+05	1.32E+05	0.948819088	0.034565564	0.4129479
O88544	COP9 signalosome complex subunit 4 OS=Mus musculus OX=10090 GN=Cops4 PE=1 SV=1	5.50E+05	5.72E+05	5.68E+05	532775.17	5.44E+05	5.25E+05	0.948027398	0.026539629	0.3876421
P83877	Thioredoxin-like protein 4A OS=Mus musculus OX=10090 GN=Txnl4a PE=1 SV=1	2.53E+05	2.54E+05	2.46E+05	241785.67	2.31E+05	2.41E+05	0.947256021	0.036754506	0.4171111
Q6ZPU9	KIF-binding protein OS=Mus musculus OX=10090 GN=Kifbp PE=1 SV=2	1.42E+05	1.46E+05	1.48E+05	141018.24	1.34E+05	1.38E+05	0.945848087	0.03779828	0.4229148
Q9D4J1	EF-hand domain-containing protein D1 OS=Mus musculus OX=10090 GN=Efhd1 PE=1 SV=1	8.78E+04	8.65E+04	8.49E+04	81791.24	8.03E+04	8.29E+04	0.945337617	0.01393535	0.3489800
P17183	Gamma-enolase OS=Mus musculus OX=10090 GN=Eno2 PE=1 SV=2	3.73E+06	3.64E+06	3.70E+06	3556512.12	3.51E+06	3.36E+06	0.94276032	0.031445744	0.3995656
Q61035	Histidine--tRNA ligase, cytoplasmic OS=Mus musculus OX=10090 GN=Hars1 PE=1 SV=2	4.98E+05	4.83E+05	4.92E+05	449509.91	4.71E+05	4.67E+05	0.941898985	0.022630557	0.3595542
Q9JHU4	Cytoplasmic dynein 1 heavy chain 1 OS=Mus musculus OX=10090 GN=Dync1h1 PE=1 SV=2	6.33E+06	6.19E+06	6.10E+06	5981396.39	5.80E+06	5.74E+06	0.941165603	0.020481502	0.3521754
Q8R0X7	Sphingosine-1-phosphate lyase 1 OS=Mus musculus OX=10090 GN=Sgpl1 PE=1 SV=1	7.90E+05	8.25E+05	7.75E+05	757571.26	7.55E+05	7.35E+05	0.940006488	0.04461425	0.4285182
P51125	Calpastatin OS=Mus musculus OX=10090 GN=Cast PE=1 SV=2	1.33E+06	1.30E+06	1.36E+06	1217857.47	1.26E+06	1.27E+06	0.939548194	0.031251314	0.3991325
Q9CPQ3	Mitochondrial import receptor subunit TOM22 homolog OS=Mus musculus OX=10090 GN=Tomm22 PE=1 SV=3	3.17E+05	3.13E+05	3.05E+05	284742.43	2.90E+05	3.04E+05	0.938662886	0.047236941	0.4329233
Q60870	Receptor expression-enhancing protein 5 OS=Mus musculus OX=10090 GN=Reep5 PE=1 SV=1	8.07E+05	7.75E+05	7.81E+05	713243.05	7.60E+05	7.45E+05	0.938602849	0.04707446	0.4329233
P47738	Aldehyde dehydrogenase, mitochondrial OS=Mus musculus OX=10090 GN=Aldh2 PE=1 SV=1	1.04E+06	1.10E+06	1.06E+06	983435.75	9.98E+05	1.02E+06	0.937752174	0.042725996	0.4270203
Q6PDI6	Ubiquitin carboxyl-terminal hydrolase MINDY-2 OS=Mus musculus OX=10090 GN=Mindy2 PE=1 SV=1	3.65E+05	3.70E+05	3.68E+05	350628.46	3.30E+05	3.53E+05	0.936977563	0.033417232	0.4059120
Q8K2I1	Protein farnesyltransferase subunit beta OS=Mus musculus OX=10090 GN=Fntb PE=1 SV=1	6.32E+05	6.46E+05	6.43E+05	612047.78	5.92E+05	5.92E+05	0.934949839	0.005891924	0.3039314
P47867	Secretogranin-3 OS=Mus musculus OX=10090 GN=Scg3 PE=1 SV=1	6.10E+04	5.75E+04	5.94E+04	55402.55	5.38E+04	5.70E+04	0.933655745	0.047138179	0.4329233
P48758	Carbonyl reductase [NADPH] 1 OS=Mus musculus OX=10090 GN=Cbr1 PE=1 SV=3	3.22E+05	3.17E+05	3.37E+05	312350.26	3.00E+05	2.98E+05	0.93350169	0.042539246	0.4270203
Q8C0L6	Peroxisomal N(1)-acetyl-spermine/spermidine oxidase OS=Mus musculus OX=10090 GN=Paox PE=1 SV=3	6.43E+04	6.26E+04	6.30E+04	61233.37	5.73E+04	5.88E+04	0.933366747	0.029466746	0.3944086
Q8R3T5	Syntaxin-binding protein 6 OS=Mus musculus OX=10090 GN=Stxbp6 PE=1 SV=1	5.67E+05	5.39E+05	5.71E+05	516640.20	5.24E+05	5.24E+05	0.933222986	0.022041542	0.3561421
Q8BYA0	Tubulin-specific chaperone D OS=Mus musculus OX=10090 GN=Tbcd PE=1 SV=1	3.50E+04	3.57E+04	3.56E+04	33885.60	3.37E+04	3.15E+04	0.931746708	0.038568159	0.4247452
Q8K0T4	Katanin p60 ATPase-containing subunit A-like 1 OS=Mus musculus OX=10090 GN=Katnal1 PE=1 SV=1	1.05E+05	1.02E+05	1.07E+05	99343.42	9.73E+04	9.64E+04	0.93149115	0.014218561	0.3489800
Q91YH5	Atlastin-3 OS=Mus musculus OX=10090 GN=Atl3 PE=1 SV=1	2.39E+05	2.53E+05	2.50E+05	231965.19	2.22E+05	2.36E+05	0.930473309	0.043855637	0.4270203
Q02053	Ubiquitin-like modifier-activating enzyme 1 OS=Mus musculus OX=10090 GN=Uba1 PE=1 SV=1	1.40E+06	1.43E+06	1.43E+06	1345405.32	1.30E+06	1.32E+06	0.928754715	0.004978382	0.2996080
P28230	Gap junction beta-1 protein OS=Mus musculus OX=10090 GN=Gjb1 PE=1 SV=3	6.74E+05	6.28E+05	6.46E+05	601660.84	5.94E+05	6.13E+05	0.928724278	0.032764716	0.4020170
Q9DCT2	NADH dehydrogenase [ubiquinone] iron-sulfur protein 3, mitochondrial OS=Mus musculus OX=10090 GN=Ndufs3 PE=1 SV=2	6.64E+05	6.45E+05	6.25E+05	597008.85	6.07E+05	5.93E+05	0.928708687	0.018572986	0.3521754
Q91YM2	Rho GTPase-activating protein 35 OS=Mus musculus OX=10090 GN=Arhgap35 PE=1 SV=3	1.35E+05	1.32E+05	1.34E+05	128237.50	1.25E+05	1.18E+05	0.927031559	0.033874144	0.4089607
Q91YN5	UDP-N-acetylhexosamine pyrophosphorylase OS=Mus musculus OX=10090 GN=Uap1 PE=1 SV=1	8.14E+04	8.54E+04	8.32E+04	74147.20	7.75E+04	7.99E+04	0.926560324	0.039314253	0.4270203
Q9DCW4	Electron transfer flavoprotein subunit beta OS=Mus musculus OX=10090 GN=Etfb PE=1 SV=3	2.37E+05	2.42E+05	2.31E+05	219720.75	2.20E+05	2.17E+05	0.926353134	0.0052172	0.3029392
P62983	Ubiquitin-40S ribosomal protein S27a OS=Mus musculus OX=10090 GN=Rps27a PE=1 SV=2	4.52E+06	4.40E+06	4.28E+06	4181712.51	4.03E+06	4.01E+06	0.925776978	0.019319408	0.3521754
Q9EQX4	Allograft inflammatory factor 1-like OS=Mus musculus OX=10090 GN=Aif1l PE=1 SV=1	4.62E+05	4.58E+05	4.54E+05	439743.69	4.31E+05	4.01E+05	0.924527947	0.045012565	0.4297834
Q8QZS1	3-hydroxyisobutyryl-CoA hydrolase, mitochondrial OS=Mus musculus OX=10090 GN=Hibch PE=1 SV=1	5.51E+05	5.44E+05	5.14E+05	509321.97	4.98E+05	4.78E+05	0.923192333	0.046558729	0.4329233
Q9Z1N5	Spliceosome RNA helicase Ddx39b OS=Mus musculus OX=10090 GN=Ddx39b PE=1 SV=1	3.34E+05	3.31E+05	3.10E+05	306994.57	2.92E+05	3.00E+05	0.921640837	0.042964644	0.4270203
O35350	Calpain-1 catalytic subunit OS=Mus musculus OX=10090 GN=Capn1 PE=1 SV=1	4.62E+05	4.65E+05	4.59E+05	438774.50	4.32E+05	4.06E+05	0.921022386	0.023535529	0.3645916
Q9CZ69	CKLF-like MARVEL transmembrane domain-containing protein 6 OS=Mus musculus OX=10090 GN=Cmtm6 PE=1 SV=1	3.04E+06	3.00E+06	2.79E+06	2705742.15	2.75E+06	2.67E+06	0.920269177	0.04527989	0.4304038
Q8BH48	Ubiquitin-associated protein 1 OS=Mus musculus OX=10090 GN=Ubap1 PE=1 SV=1	1.52E+05	1.54E+05	1.52E+05	146203.21	1.43E+05	1.32E+05	0.920030603	0.04788053	0.434574
O70311	Glycylpeptide N-tetradecanoyltransferase 2 OS=Mus musculus OX=10090 GN=Nmt2 PE=1 SV=1	3.97E+05	4.16E+05	4.22E+05	389646.59	3.73E+05	3.71E+05	0.918334264	0.023590145	0.3645916
Q9DCH6	AN1-type zinc finger protein 6 OS=Mus musculus OX=10090 GN=Zfand6 PE=1 SV=1	5.53E+04	5.85E+04	6.01E+04	53180.91	5.42E+04	5.21E+04	0.917707803	0.035470042	0.4129479
Q60714	Long-chain fatty acid transport protein 1 OS=Mus musculus OX=10090 GN=Slc27a1 PE=1 SV=1	2.60E+05	2.79E+05	2.61E+05	248300.46	2.46E+05	2.40E+05	0.917339573	0.031988695	0.3998493
Q921J2	GTP-binding protein Rheb OS=Mus musculus OX=10090 GN=Rheb PE=1 SV=1	3.02E+05	3.09E+05	3.01E+05	289164.95	2.73E+05	2.75E+05	0.91716277	0.011801778	0.3480419
Q8CD92	Tetratricopeptide repeat protein 27 OS=Mus musculus OX=10090 GN=Ttc27 PE=1 SV=2	9.09E+04	8.72E+04	8.85E+04	83392.40	8.14E+04	7.96E+04	0.91686483	0.00902371	0.3258379
Q8VHL1	Histone-lysine N-methyltransferase SETD7 OS=Mus musculus OX=10090 GN=Setd7 PE=1 SV=2	4.92E+05	4.98E+05	4.84E+05	442499.05	4.41E+05	4.65E+05	0.915457091	0.00951875	0.3280756
Q9EQ20	Methylmalonate-semialdehyde dehydrogenase [acylating], mitochondrial OS=Mus musculus OX=10090 GN=Aldh6a1 PE=1 SV=1	5.62E+05	5.89E+05	5.74E+05	524243.73	5.43E+05	5.11E+05	0.914989302	0.01626918	0.3489800
Q8CHC4	Synaptojanin-1 OS=Mus musculus OX=10090 GN=Synj1 PE=1 SV=3	5.23E+05	4.98E+05	5.06E+05	462523.99	4.81E+05	4.53E+05	0.91453041	0.016939504	0.3489800
P21447	ATP-dependent translocase ABCB1 OS=Mus musculus OX=10090 GN=Abcb1a PE=1 SV=3	5.57E+05	5.51E+05	5.26E+05	502762.50	5.14E+05	4.79E+05	0.914300267	0.029290007	0.3944086
Q9JLC8	Sacsin OS=Mus musculus OX=10090 GN=Sacs PE=1 SV=2	2.36E+05	2.39E+05	2.34E+05	227755.89	2.09E+05	2.11E+05	0.914149109	0.029927641	0.396241
O35639	Annexin A3 OS=Mus musculus OX=10090 GN=Anxa3 PE=1 SV=4	3.18E+05	3.20E+05	3.32E+05	310346.57	2.94E+05	2.83E+05	0.91374073	0.038124591	0.4247452
Q9ERD7	Tubulin beta-3 chain OS=Mus musculus OX=10090 GN=Tubb3 PE=1 SV=1	1.15E+06	1.06E+06	1.06E+06	996809.24	9.94E+05	9.90E+05	0.913564655	0.037577196	0.4225000
Q9WV98	Mitochondrial import inner membrane translocase subunit Tim9 OS=Mus musculus OX=10090 GN=Timm9 PE=1 SV=1	1.83E+05	1.88E+05	1.86E+05	173542.57	1.62E+05	1.73E+05	0.913432636	0.017586662	0.350746
Q5EBH1	Ras association domain-containing protein 5 OS=Mus musculus OX=10090 GN=Rassf5 PE=1 SV=1	1.58E+05	1.52E+05	1.44E+05	141546.03	1.36E+05	1.38E+05	0.912869308	0.044664292	0.4285182
Q61503	5'-nucleotidase OS=Mus musculus OX=10090 GN=Nt5e PE=1 SV=2	1.59E+05	1.57E+05	1.52E+05	143609.40	1.39E+05	1.44E+05	0.912017694	0.004655488	0.2962781
Q9D7X8	Gamma-glutamylcyclotransferase OS=Mus musculus OX=10090 GN=Ggct PE=1 SV=1	3.29E+05	3.22E+05	3.31E+05	296617.91	3.01E+05	2.98E+05	0.911787458	0.000558436	0.158849
Q6NZK8	Protein tyrosine phosphatase domain-containing protein 1 OS=Mus musculus OX=10090 GN=Ptpdc1 PE=1 SV=1	1.46E+05	1.52E+05	1.52E+05	134796.30	1.37E+05	1.39E+05	0.911725116	0.005513639	0.3029392
Q8R1Q8	Cytoplasmic dynein 1 light intermediate chain 1 OS=Mus musculus OX=10090 GN=Dync1li1 PE=1 SV=1	3.47E+05	3.32E+05	3.26E+05	314524.62	3.00E+05	3.01E+05	0.911492843	0.016911519	0.3489800
P53810	Phosphatidylinositol transfer protein alpha isoform OS=Mus musculus OX=10090 GN=Pitpna PE=1 SV=2	1.23E+06	1.21E+06	1.24E+06	1128505.52	1.16E+06	1.07E+06	0.911051038	0.018040123	0.3521754
Q9CX00	IST1 homolog OS=Mus musculus OX=10090 GN=Ist1 PE=1 SV=1	2.18E+05	2.02E+05	1.97E+05	185211.27	1.87E+05	1.90E+05	0.910962162	0.045964819	0.4324410
Q9CR67	Transmembrane protein 33 OS=Mus musculus OX=10090 GN=Tmem33 PE=1 SV=1	2.77E+05	2.77E+05	2.74E+05	265885.66	2.42E+05	2.46E+05	0.910361742	0.029760232	0.396241
Q0P678	Zinc finger CCCH domain-containing protein 18 OS=Mus musculus OX=10090 GN=Zc3h18 PE=1 SV=1	1.32E+05	1.20E+05	1.28E+05	119210.64	1.15E+05	1.12E+05	0.909332165	0.049157578	0.4387728
A6H630	Damage-control phosphatase ARMT1 OS=Mus musculus OX=10090 GN=Armt1 PE=1 SV=1	2.02E+05	1.85E+05	1.92E+05	173043.63	1.78E+05	1.75E+05	0.908517054	0.028608458	0.3937279
A2AG50	MAP7 domain-containing protein 2 OS=Mus musculus OX=10090 GN=Map7d2 PE=1 SV=1	7.86E+05	7.27E+05	7.45E+05	665624.73	7.06E+05	6.78E+05	0.907907742	0.030757484	0.3966517
Q0KL02	Triple functional domain protein OS=Mus musculus OX=10090 GN=Trio PE=1 SV=3	8.60E+04	9.25E+04	8.71E+04	79165.11	8.21E+04	7.97E+04	0.907619203	0.020267667	0.3521754
Q80UK0	SEC14 domain and spectrin repeat-containing protein 1 OS=Mus musculus OX=10090 GN=Sestd1 PE=1 SV=1	2.36E+05	2.21E+05	2.34E+05	212553.48	2.14E+05	2.01E+05	0.907543303	0.026612459	0.3876421
P49443	Protein phosphatase 1A OS=Mus musculus OX=10090 GN=Ppm1a PE=1 SV=1	2.53E+05	2.53E+05	2.44E+05	240233.01	2.23E+05	2.17E+05	0.907217625	0.038297232	0.4247452
Q9D892	Inosine triphosphate pyrophosphatase OS=Mus musculus OX=10090 GN=Itpa PE=1 SV=2	5.92E+05	6.02E+05	5.56E+05	539878.98	5.24E+05	5.21E+05	0.905026193	0.021989434	0.3561421

Q3UPL5	Uncharacterized protein C11orf96 homolog OS=Mus musculus OX=10090 GN=Ag2 PE=1 SV=2	2.21E+05	2.44E+05	2.32E+05	207597.54	2.16E+05	2.07E+05	0.904710097	0.040668024	0.4270203
Q8R146	Acylamino-acid-releasing enzyme OS=Mus musculus OX=10090 GN=Apeh PE=1 SV=3	2.80E+05	2.81E+05	2.79E+05	241639.32	2.55E+05	2.63E+05	0.904040268	0.013311385	0.3480419
P20444	Protein kinase C alpha type OS=Mus musculus OX=10090 GN=Prkca PE=1 SV=3	5.38E+05	5.20E+05	5.23E+05	465356.26	4.84E+05	4.78E+05	0.902792468	0.003104566	0.2962781
Q91VZ6	Stromal membrane-associated protein 1 OS=Mus musculus OX=10090 GN=Smap1 PE=1 SV=1	9.24E+04	9.28E+04	8.64E+04	85340.65	8.22E+04	7.75E+04	0.902491997	0.045496312	0.4306465
P48722	Heat shock 70 kDa protein 4L OS=Mus musculus OX=10090 GN=Hspa4l PE=1 SV=2	5.37E+05	5.57E+05	5.16E+05	487720.37	4.75E+05	4.90E+05	0.90219612	0.014547606	0.3489800
Q62000	Mimecan OS=Mus musculus OX=10090 GN=Ogn PE=1 SV=1	1.27E+06	1.26E+06	1.25E+06	1078118.58	1.10E+06	1.22E+06	0.90143974	0.047412299	0.4329233
Q8R3Q6	Coiled-coil domain-containing protein 58 OS=Mus musculus OX=10090 GN=Ccdc58 PE=1 SV=1	1.43E+05	1.45E+05	1.49E+05	128213.61	1.35E+05	1.30E+05	0.900844818	0.00564064	0.3029392
Q9R0P3	S-formylglutathione hydrolase OS=Mus musculus OX=10090 GN=Esd PE=1 SV=1	3.23E+05	3.44E+05	3.17E+05	293283.32	2.89E+05	3.01E+05	0.898672213	0.021056114	0.3559661
Q9R0U0	Serine/arginine-rich splicing factor 10 OS=Mus musculus OX=10090 GN=Srsf10 PE=1 SV=2	3.96E+04	4.04E+04	4.09E+04	37541.93	3.72E+04	3.39E+04	0.898184424	0.028411047	0.3937279
A2A6T1	Cerebellar degeneration-related protein 2-like OS=Mus musculus OX=10090 GN=Cdr2l PE=1 SV=1	3.64E+05	3.45E+05	3.46E+05	315570.65	3.12E+05	3.18E+05	0.896991783	0.004848459	0.2962781
Q9WTX5	S-phase kinase-associated protein 1 OS=Mus musculus OX=10090 GN=Skp1 PE=1 SV=3	4.22E+05	4.06E+05	4.26E+05	373617.68	3.82E+05	3.67E+05	0.895306391	0.00392587	0.2962781
Q8BYN5	FSD1-like protein OS=Mus musculus OX=10090 GN=Fsd1l PE=1 SV=2	1.44E+05	1.47E+05	1.41E+05	122770.78	1.25E+05	1.39E+05	0.894915786	0.044078129	0.4270203
E9Q394	A-kinase anchor protein 13 OS=Mus musculus OX=10090 GN=Akap13 PE=1 SV=1	9.88E+04	9.97E+04	1.03E+05	93081.04	8.96E+04	8.66E+04	0.894619317	0.008189398	0.3180017
Q8VIM9	Immunity-related GTPase family Q protein OS=Mus musculus OX=10090 GN=Irgq PE=1 SV=1	3.00E+05	2.97E+05	2.89E+05	278533.42	2.60E+05	2.54E+05	0.894481269	0.018642494	0.3521754
P16125	L-lactate dehydrogenase B chain OS=Mus musculus OX=10090 GN=Ldhb PE=1 SV=2	8.49E+05	7.86E+05	7.88E+05	712732.49	7.18E+05	7.35E+05	0.894205524	0.016800646	0.3489800
Q99P30	Peroxisomal coenzyme A diphosphatase NUDT7 OS=Mus musculus OX=10090 GN=Nudt7 PE=1 SV=2	2.03E+04	2.13E+04	1.94E+04	18989.44	1.75E+04	1.80E+04	0.893535215	0.037654839	0.4225000
Q91YD9	Neural Wiskott-Aldrich syndrome protein OS=Mus musculus OX=10090 GN=Wasl PE=1 SV=1	1.49E+05	1.47E+05	1.39E+05	131842.74	1.29E+05	1.28E+05	0.893161271	0.008149729	0.3180017
Q9DBJ1	Phosphoglycerate mutase 1 OS=Mus musculus OX=10090 GN=Pgam1 PE=1 SV=3	1.94E+06	1.86E+06	1.81E+06	1715408.50	1.69E+06	1.61E+06	0.893023703	0.017021407	0.3489800
O70362	Phosphatidylinositol-glycan-specific phospholipase D OS=Mus musculus OX=10090 GN=Gpld1 PE=1 SV=1	2.72E+05	2.84E+05	2.66E+05	233571.19	2.43E+05	2.58E+05	0.892967457	0.02936706	0.3944086
Q8K389	CDK5 regulatory subunit-associated protein 2 OS=Mus musculus OX=10090 GN=Cdk5rap2 PE=1 SV=3	5.51E+04	6.04E+04	6.16E+04	53800.67	5.12E+04	5.32E+04	0.892946653	0.042761961	0.4270203
Q99LC3	NADH dehydrogenase [ubiquinone] 1 alpha subcomplex subunit 10, mitochondrial OS=Mus musculus OX=10090 GN=Ndufa10 PE=1 SV=1	1.25E+05	1.36E+05	1.23E+05	113057.43	1.12E+05	1.18E+05	0.892226695	0.040965668	0.4270203
Q61165	Sodium/hydrogen exchanger 1 OS=Mus musculus OX=10090 GN=Slc9a1 PE=1 SV=1	4.56E+04	4.53E+04	4.58E+04	40061.95	4.11E+04	4.07E+04	0.89149626	0.000119132	0.100290
Q8CIV8	Tubulin-specific chaperone E OS=Mus musculus OX=10090 GN=Tbce PE=1 SV=1	1.93E+05	1.88E+05	1.72E+05	169140.60	1.63E+05	1.60E+05	0.89064883	0.039444546	0.4270203
Q64520	Guanylate kinase OS=Mus musculus OX=10090 GN=Guk1 PE=1 SV=2	2.48E+05	2.52E+05	2.48E+05	230627.82	2.27E+05	2.08E+05	0.890411593	0.019856101	0.3521754
P62960	Y-box-binding protein 1 OS=Mus musculus OX=10090 GN=Ybx1 PE=1 SV=3	6.57E+05	7.00E+05	6.48E+05	618725.24	5.77E+05	5.87E+05	0.889267319	0.021905263	0.3561421
P61294	Ras-related protein Rab-6B OS=Mus musculus OX=10090 GN=Rab6b PE=1 SV=1	4.58E+04	4.68E+04	4.73E+04	42458.41	4.31E+04	3.88E+04	0.88918302	0.022385991	0.3578990
Q9Z0W3	Nuclear pore complex protein Nup160 OS=Mus musculus OX=10090 GN=Nup160 PE=1 SV=2	2.69E+04	2.97E+04	2.66E+04	25263.07	2.46E+04	2.40E+04	0.887599772	0.041403088	0.4270203
Q9DCZ4	MICOS complex subunit Mic26 OS=Mus musculus OX=10090 GN=Apoo PE=1 SV=2	4.53E+05	4.63E+05	4.46E+05	402179.69	3.90E+05	4.16E+05	0.886981775	0.004686333	0.2962781
A2AKQ0	UDP-glucuronic acid/UDP-N-acetylgalactosamine transporter OS=Mus musculus OX=10090 GN=Slc35d1 PE=2 SV=2	8.32E+04	8.42E+04	7.95E+04	73693.52	7.00E+04	7.52E+04	0.886847322	0.011812301	0.3480419
Q8C7R4	Ubiquitin-like modifier-activating enzyme 6 OS=Mus musculus OX=10090 GN=Uba6 PE=1 SV=1	1.15E+05	1.07E+05	1.12E+05	97367.23	9.33E+04	1.05E+05	0.885829745	0.042058791	0.4270203
O70152	Dolichol-phosphate mannosyltransferase subunit 1 OS=Mus musculus OX=10090 GN=Dpm1 PE=1 SV=1	9.60E+04	9.52E+04	9.74E+04	83687.95	7.96E+04	9.22E+04	0.885355358	0.042549966	0.4270203
P05063	Fructose-bisphosphate aldolase C OS=Mus musculus OX=10090 GN=Aldoc PE=1 SV=4	5.57E+05	4.91E+05	5.06E+05	454421.15	4.53E+05	4.65E+05	0.883138816	0.040186386	0.4270203
Q7TSH2	Phosphorylase b kinase regulatory subunit beta OS=Mus musculus OX=10090 GN=Phkb PE=1 SV=1	9.28E+04	9.06E+04	8.99E+04	82426.58	7.96E+04	7.92E+04	0.882919866	0.00136698	0.2585545
Q9ER58	Testican-2 OS=Mus musculus OX=10090 GN=Spock2 PE=1 SV=1	1.12E+05	1.13E+05	1.11E+05	107309.34	9.38E+04	9.57E+04	0.881543422	0.035189848	0.4129479
Q8VD75	Huntingtin-interacting protein 1 OS=Mus musculus OX=10090 GN=Hip1 PE=1 SV=2	2.02E+05	2.13E+05	2.18E+05	194482.68	1.93E+05	1.71E+05	0.881051573	0.047921407	0.434574
Q62418	Drebrin-like protein OS=Mus musculus OX=10090 GN=Dbnl PE=1 SV=2	2.38E+05	2.48E+05	2.33E+05	218281.45	2.13E+05	2.02E+05	0.880607989	0.012015243	0.3480419
P61759	Prefoldin subunit 3 OS=Mus musculus OX=10090 GN=Vbp1 PE=1 SV=2	1.11E+05	1.15E+05	1.04E+05	98841.43	9.31E+04	9.81E+04	0.87983922	0.019858632	0.3521754
Q9Z2C9	Myotubularin-related protein 7 OS=Mus musculus OX=10090 GN=Mtmr7 PE=1 SV=2	1.57E+05	1.59E+05	1.56E+05	140553.75	1.37E+05	1.37E+05	0.879663062	0.000132261	0.100290
P98191	Phosphatidate cytidylyltransferase 1 OS=Mus musculus OX=10090 GN=Cds1 PE=1 SV=2	4.69E+04	4.65E+04	4.56E+04	39373.02	4.18E+04	4.10E+04	0.878899057	0.002219977	0.2962781
Q60676	Serine/threonine-protein phosphatase 5 OS=Mus musculus OX=10090 GN=Ppp5c PE=1 SV=3	8.57E+04	8.24E+04	8.54E+04	77723.70	7.53E+04	6.96E+04	0.878071817	0.017286059	0.3489800
Q80WT5	Aftiphilin OS=Mus musculus OX=10090 GN=Aftph PE=1 SV=2	5.71E+04	5.89E+04	6.33E+04	54134.20	5.19E+04	5.14E+04	0.87748032	0.021977404	0.3561421
P24526	Myelin P2 protein OS=Mus musculus OX=10090 GN=Pmp2 PE=2 SV=2	5.08E+05	5.57E+05	5.57E+05	470504.29	4.55E+05	4.96E+05	0.8763888	0.029491278	0.3944086
Q6P1B1	Xaa-Pro aminopeptidase 1 OS=Mus musculus OX=10090 GN=Xpnpep1 PE=1 SV=1	1.11E+05	1.15E+05	1.16E+05	95317.99	1.06E+05	9.89E+04	0.875607189	0.016385051	0.3489800
Q3UVK0	Endoplasmic reticulum metallopeptidase 1 OS=Mus musculus OX=10090 GN=Ermp1 PE=1 SV=2	2.11E+05	2.06E+05	2.12E+05	185364.88	1.85E+05	1.80E+05	0.874641563	0.000518313	0.158849
Q61249	Immunoglobulin-binding protein 1 OS=Mus musculus OX=10090 GN=Igbp1 PE=1 SV=1	4.27E+05	4.03E+05	3.91E+05	379078.16	3.56E+05	3.32E+05	0.87426019	0.042439963	0.4270203
P97328	Ketohexokinase OS=Mus musculus OX=10090 GN=Khk PE=1 SV=1	1.58E+05	1.48E+05	1.48E+05	131819.97	1.33E+05	1.30E+05	0.869882758	0.004578935	0.2962781
Q9Z2I9	Succinate--CoA ligase [ADP-forming] subunit beta, mitochondrial OS=Mus musculus OX=10090 GN=Sucla2 PE=1 SV=2	1.23E+06	1.28E+06	1.17E+06	1104657.34	1.07E+06	1.02E+06	0.869689478	0.016706712	0.3489800
Q8BKZ9	Pyruvate dehydrogenase protein X component, mitochondrial OS=Mus musculus OX=10090 GN=Pdhx PE=1 SV=1	3.31E+05	3.10E+05	3.02E+05	276574.14	2.63E+05	2.78E+05	0.868423064	0.01407128	0.3489800
Q8K2I3	Dimethylaniline monooxygenase [N-oxide-forming] 2 OS=Mus musculus OX=10090 GN=Fmo2 PE=1 SV=3	4.96E+04	5.68E+04	4.96E+04	45611.59	4.47E+04	4.51E+04	0.868349901	0.047759287	0.434574
P19536	Cytochrome c oxidase subunit 5B, mitochondrial OS=Mus musculus OX=10090 GN=Cox5b PE=1 SV=1	2.77E+05	2.83E+05	2.57E+05	256236.66	2.28E+05	2.24E+05	0.867236864	0.049106039	0.4387728
Q923D3	Prostate androgen-regulated mucin-like protein 1 homolog OS=Mus musculus OX=10090 GN=Parm1 PE=1 SV=1	1.55E+05	1.56E+05	1.60E+05	146643.60	1.36E+05	1.26E+05	0.867221244	0.028250591	0.3937240
P51642	Ciliary neurotrophic factor OS=Mus musculus OX=10090 GN=Cntf PE=2 SV=1	4.59E+05	4.65E+05	4.75E+05	379828.83	4.26E+05	4.08E+05	0.866958992	0.011775038	0.3480419
O35551	Rab GTPase-binding effector protein 1 OS=Mus musculus OX=10090 GN=Rabep1 PE=1 SV=2	1.85E+05	1.87E+05	1.98E+05	155143.61	1.74E+05	1.65E+05	0.866560245	0.020085095	0.3521754
O08539	Myc box-dependent-interacting protein 1 OS=Mus musculus OX=10090 GN=Bin1 PE=1 SV=1	2.56E+05	2.56E+05	2.59E+05	238170.58	2.13E+05	2.17E+05	0.866340336	0.011951338	0.3480419
P61222	ATP-binding cassette sub-family E member 1 OS=Mus musculus OX=10090 GN=Abce1 PE=1 SV=1	2.04E+05	1.81E+05	1.91E+05	169397.00	1.61E+05	1.68E+05	0.865724908	0.021553683	0.3559661
Q9QXN3	Activating signal cointegrator 1 OS=Mus musculus OX=10090 GN=Trip4 PE=1 SV=2	1.99E+04	1.93E+04	1.91E+04	17826.04	1.67E+04	1.59E+04	0.865432388	0.01297377	0.3480419
Q9JI75	Ribosyldihydronicotinamide dehydrogenase [quinone] OS=Mus musculus OX=10090 GN=Nqo2 PE=1 SV=3	1.48E+05	1.37E+05	1.45E+05	125733.74	1.30E+05	1.16E+05	0.864434609	0.023186305	0.3625826
P63024	Vesicle-associated membrane protein 3 OS=Mus musculus OX=10090 GN=Vamp3 PE=1 SV=1	2.50E+05	2.34E+05	2.33E+05	209260.05	2.21E+05	1.88E+05	0.86242773	0.038902577	0.4256777
O88456	Calpain small subunit 1 OS=Mus musculus OX=10090 GN=Capns1 PE=1 SV=1	5.71E+05	5.28E+05	5.34E+05	462987.52	4.76E+05	4.68E+05	0.862098216	0.005991312	0.3050960
E9Q401	Ryanodine receptor 2 OS=Mus musculus OX=10090 GN=Ryr2 PE=1 SV=1	6.38E+04	7.06E+04	6.17E+04	57753.00	5.80E+04	5.31E+04	0.861055297	0.043352733	0.4270203
Q9D394	Protein RUFY3 OS=Mus musculus OX=10090 GN=Rufy3 PE=1 SV=1	6.77E+05	6.09E+05	6.69E+05	598298.28	5.22E+05	5.61E+05	0.860312099	0.041954837	0.4270203
P18654	Ribosomal protein S6 kinase alpha-3 OS=Mus musculus OX=10090 GN=Rps6ka3 PE=1 SV=2	4.70E+05	4.65E+05	4.47E+05	386690.86	4.14E+05	3.87E+05	0.860085926	0.004827156	0.2962781
O35295	Transcriptional activator protein Pur-beta OS=Mus musculus OX=10090 GN=Purb PE=1 SV=3	3.45E+05	3.38E+05	3.32E+05	306953.46	2.61E+05	3.03E+05	0.859210841	0.034247398	0.4122141
Q80TZ3	Putative tyrosine-protein phosphatase auxilin OS=Mus musculus OX=10090 GN=Dnajc6 PE=1 SV=2	1.82E+05	1.57E+05	1.66E+05	145053.75	1.49E+05	1.41E+05	0.859158202	0.036434863	0.4158599
Q9QUR8	Semaphorin-7A OS=Mus musculus OX=10090 GN=Sema7a PE=1 SV=1	1.30E+05	1.12E+05	1.25E+05	100096.74	1.11E+05	1.04E+05	0.858815889	0.049912594	0.4405618
Q9Z0S1	3'(2'),5'-bisphosphate nucleotidase 1 OS=Mus musculus OX=10090 GN=Bpnt1 PE=1 SV=2	2.08E+05	2.21E+05	2.09E+05	198140.30	1.82E+05	1.66E+05	0.857771647	0.03946656	0.4270203
Q9D6G9	CKLF-like MARVEL transmembrane domain-containing protein 5 OS=Mus musculus OX=10090 GN=Cmtm5 PE=1 SV=1	1.57E+05	1.46E+05	1.40E+05	131146.63	1.26E+05	1.23E+05	0.857708003	0.019382774	0.3521754
Q9D2N4	Dystrobrevin alpha OS=Mus musculus OX=10090 GN=Dtna PE=1 SV=2	6.53E+04	6.29E+04	6.70E+04	57866.27	5.51E+04	5.44E+04	0.857336665	0.00421758	0.2962781
P70460	Vasodilator-stimulated phosphoprotein OS=Mus musculus OX=10090 GN=Vasp PE=1 SV=4	4.29E+05	4.75E+05	4.44E+05	391608.60	3.94E+05	3.69E+05	0.857061995	0.015830207	0.3489800
P97494	Glutamate--cysteine ligase catalytic subunit OS=Mus musculus OX=10090 GN=Gclc PE=1 SV=4	5.64E+05	5.44E+05	5.52E+05	455665.10	4.93E+05	4.72E+05	0.856286559	0.00294427	0.2962781
O89112	Glutathione S-transferase LANCL1 OS=Mus musculus OX=10090 GN=Lancl1 PE=1 SV=1	9.43E+04	9.22E+04	1.00E+05	83209.45	7.67E+04	8.56E+04	0.855629267	0.019057828	0.3521754
Q64512	Tyrosine-protein phosphatase non-receptor type 13 OS=Mus musculus OX=10090 GN=Ptpn13 PE=1 SV=2	3.90E+04	3.92E+04	3.62E+04	33461.41	2.99E+04	3.45E+04	0.855549009	0.031486419	0.3995656

Q8K019	Bcl-2-associated transcription factor 1 OS=Mus musculus OX=10090 GN=Bclaf1 PE=1 SV=2	1.46E+05	1.53E+05	1.48E+05	140319.77	1.20E+05	1.21E+05	0.855257363	0.035399927	0.4129479
P19246	Neurofilament heavy polypeptide OS=Mus musculus OX=10090 GN=Nefh PE=1 SV=3	2.03E+07	1.88E+07	1.86E+07	17334230.79	1.61E+07	1.58E+07	0.85294378	0.0155268	0.3489800
P63040	Complexin-1 OS=Mus musculus OX=10090 GN=Cplx1 PE=1 SV=1	6.75E+05	7.10E+05	7.04E+05	625244.61	5.88E+05	5.69E+05	0.852758395	0.006646806	0.3110762
Q99LR1	Lysophosphatidylserine lipase ABHD12 OS=Mus musculus OX=10090 GN=Abhd12 PE=1 SV=2	5.31E+05	4.78E+05	4.96E+05	411005.11	4.67E+05	4.06E+05	0.852693477	0.042798551	0.4270203
Q8VDC0	Probable leucine--tRNA ligase, mitochondrial OS=Mus musculus OX=10090 GN=Lars2 PE=1 SV=1	6.07E+04	5.94E+04	5.51E+04	52611.02	5.09E+04	4.58E+04	0.851848499	0.031879386	0.3998493
Q64378	Peptidyl-prolyl cis-trans isomerase FKBP5 OS=Mus musculus OX=10090 GN=Fkbp5 PE=1 SV=1	2.69E+05	2.83E+05	2.64E+05	216894.72	2.49E+05	2.28E+05	0.85083647	0.021598147	0.3559661
Q80U28	MAP kinase-activating death domain protein OS=Mus musculus OX=10090 GN=Madd PE=1 SV=2	9.91E+04	9.53E+04	9.97E+04	86836.18	8.30E+04	7.94E+04	0.847794115	0.00422202	0.2962781
Q3UMR5	Calcium uniporter protein, mitochondrial OS=Mus musculus OX=10090 GN=Mcu PE=1 SV=2	9.41E+04	1.01E+05	1.01E+05	83321.68	8.52E+04	8.31E+04	0.847668585	0.003796953	0.2962781
P10852	4F2 cell-surface antigen heavy chain OS=Mus musculus OX=10090 GN=Slc3a2 PE=1 SV=1	2.32E+05	2.42E+05	2.25E+05	197797.64	2.07E+05	1.87E+05	0.846345416	0.008035227	0.3180017
P70404	Isocitrate dehydrogenase [NAD] subunit gamma 1, mitochondrial OS=Mus musculus OX=10090 GN=Idh3g PE=1 SV=1	4.00E+05	3.84E+05	3.45E+05	325583.03	3.00E+05	3.29E+05	0.845902997	0.036073664	0.4144125
O88329	Unconventional myosin-Ia OS=Mus musculus OX=10090 GN=Myo1a PE=2 SV=2	3.84E+05	3.77E+05	4.01E+05	345233.60	3.08E+05	3.28E+05	0.84426508	0.009350102	0.3280756
Q8VIJ8	GATOR complex protein NPRL3 OS=Mus musculus OX=10090 GN=Nprl3 PE=1 SV=1	3.28E+04	3.68E+04	3.21E+04	28287.65	3.05E+04	2.69E+04	0.84288938	0.040202474	0.4270203
Q9DAZ9	Abscission/NoCut checkpoint regulator OS=Mus musculus OX=10090 GN=Zfyve19 PE=1 SV=2	1.37E+05	1.23E+05	1.38E+05	115262.54	1.06E+05	1.13E+05	0.842279062	0.018785186	0.3521754
P42703	Leukemia inhibitory factor receptor OS=Mus musculus OX=10090 GN=Lifr PE=1 SV=1	2.05E+05	1.93E+05	1.98E+05	163120.59	1.54E+05	1.85E+05	0.840512229	0.030959302	0.396678
P56695	Wolframin OS=Mus musculus OX=10090 GN=Wfs1 PE=1 SV=1	9.30E+04	9.79E+04	1.02E+05	84601.57	8.05E+04	8.10E+04	0.84033708	0.00574469	0.3029392
Q6PGN3	Serine/threonine-protein kinase DCLK2 OS=Mus musculus OX=10090 GN=Dclk2 PE=1 SV=1	2.44E+05	2.35E+05	2.31E+05	206179.85	1.94E+05	1.96E+05	0.839987568	0.002288321	0.2962781
P57716	Nicastrin OS=Mus musculus OX=10090 GN=Ncstn PE=1 SV=3	2.38E+05	2.38E+05	2.32E+05	222559.26	1.82E+05	1.89E+05	0.839788199	0.039986251	0.4270203
P08551	Neurofilament light polypeptide OS=Mus musculus OX=10090 GN=Nefl PE=1 SV=5	1.59E+07	1.52E+07	1.43E+07	12668783.57	1.31E+07	1.23E+07	0.838511298	0.010603197	0.3480419
Q61730	Interleukin-1 receptor accessory protein OS=Mus musculus OX=10090 GN=Il1rap PE=1 SV=1	7.50E+04	7.85E+04	7.54E+04	59342.70	6.66E+04	6.59E+04	0.838275114	0.008636329	0.3224334
P05622	Platelet-derived growth factor receptor beta OS=Mus musculus OX=10090 GN=Pdgfrb PE=1 SV=2	1.82E+05	2.00E+05	1.81E+05	153946.42	1.65E+05	1.52E+05	0.838059041	0.016083567	0.3489800
Q3TIV5	Zinc finger CCCH domain-containing protein 15 OS=Mus musculus OX=10090 GN=Zc3h15 PE=1 SV=2	7.29E+04	7.49E+04	6.96E+04	66862.51	6.04E+04	5.50E+04	0.837981971	0.035555942	0.4129479
Q8VEK0	Cell cycle control protein 50A OS=Mus musculus OX=10090 GN=Tmem30a PE=1 SV=1	1.35E+06	1.22E+06	1.14E+06	1066891.47	1.02E+06	1.02E+06	0.837624848	0.031601422	0.3997479
P62774	Myotrophin OS=Mus musculus OX=10090 GN=Mtpn PE=1 SV=2	1.90E+06	1.88E+06	2.04E+06	1558768.03	1.69E+06	1.63E+06	0.837252455	0.006622193	0.3110762
Q9D832	DnaJ homolog subfamily B member 4 OS=Mus musculus OX=10090 GN=Dnajb4 PE=1 SV=1	2.41E+05	2.40E+05	2.20E+05	203592.36	1.95E+05	1.88E+05	0.837038809	0.009524632	0.3280756
Q9EPX2	Papilin OS=Mus musculus OX=10090 GN=Papln PE=2 SV=2	2.18E+05	2.15E+05	2.01E+05	177929.23	1.78E+05	1.73E+05	0.83498244	0.002954126	0.2962781
Q99N28	Cell adhesion molecule 3 OS=Mus musculus OX=10090 GN=Cadm3 PE=1 SV=1	7.61E+05	7.45E+05	7.20E+05	686332.58	5.96E+05	5.76E+05	0.834350015	0.02706415	0.3880823
Q80TJ1	Calcium-dependent secretion activator 1 OS=Mus musculus OX=10090 GN=Cadps PE=1 SV=3	2.07E+05	1.90E+05	2.07E+05	173421.92	1.63E+05	1.66E+05	0.832333431	0.00644983	0.3110762
P48428	Tubulin-specific chaperone A OS=Mus musculus OX=10090 GN=Tbca PE=1 SV=3	6.33E+05	5.72E+05	6.40E+05	494583.55	5.09E+05	5.30E+05	0.830889529	0.012169953	0.3480419
Q9JMA1	Ubiquitin carboxyl-terminal hydrolase 14 OS=Mus musculus OX=10090 GN=Usp14 PE=1 SV=3	1.77E+05	1.75E+05	1.69E+05	147989.47	1.42E+05	1.43E+05	0.83077013	0.000615899	0.158849
Q9D8B1	Androgen-induced gene 1 protein OS=Mus musculus OX=10090 GN=Aig1 PE=2 SV=2	1.23E+04	1.36E+04	1.23E+04	11410.75	1.07E+04	9.70E+03	0.830709102	0.03068848	0.3966517
Q99L04	Dehydrogenase/reductase SDR family member 1 OS=Mus musculus OX=10090 GN=Dhrs1 PE=1 SV=1	3.92E+05	3.63E+05	3.43E+05	284983.95	3.20E+05	3.05E+05	0.828574363	0.02293743	0.3606062
O70439	Syntaxin-7 OS=Mus musculus OX=10090 GN=Stx7 PE=1 SV=3	1.48E+05	1.45E+05	1.39E+05	119871.75	1.26E+05	1.13E+05	0.828509264	0.006425495	0.3110762
Q69ZW3	EH domain-binding protein 1 OS=Mus musculus OX=10090 GN=Ehbp1 PE=1 SV=3	1.93E+04	2.02E+04	1.75E+04	17125.10	1.45E+04	1.55E+04	0.827327173	0.040991612	0.4270203
Q91VJ5	Polyglutamine-binding protein 1 OS=Mus musculus OX=10090 GN=Pqbp1 PE=1 SV=1	1.07E+05	1.16E+05	1.06E+05	100385.06	8.71E+04	8.36E+04	0.825664165	0.033400147	0.4059120
P56389	Cytidine deaminase OS=Mus musculus OX=10090 GN=Cda PE=1 SV=2	2.17E+06	1.91E+06	2.15E+06	1806396.32	1.63E+06	1.71E+06	0.825655748	0.020141887	0.3521754
Q3UY34	Protein CUSTOS OS=Mus musculus OX=10090 GN=Custos PE=2 SV=1	3.43E+04	3.71E+04	3.35E+04	29604.69	3.10E+04	2.60E+04	0.824945396	0.0303397	0.3966517
Q80W21	Glutathione S-transferase Mu 7 OS=Mus musculus OX=10090 GN=Gstm7 PE=1 SV=1	2.39E+05	2.42E+05	2.25E+05	194429.75	1.90E+05	1.98E+05	0.824829327	0.001783681	0.2951991
Q9R1Q8	Transgelin-3 OS=Mus musculus OX=10090 GN=Tagln3 PE=1 SV=1	6.53E+05	5.83E+05	6.31E+05	545902.50	5.23E+05	4.69E+05	0.823742201	0.022776911	0.3604378
Q91XM9	Disks large homolog 2 OS=Mus musculus OX=10090 GN=Dlg2 PE=1 SV=2	3.62E+05	3.27E+05	3.18E+05	286711.55	2.76E+05	2.67E+05	0.82280716	0.014957508	0.3489800
O88343	Electrogenic sodium bicarbonate cotransporter 1 OS=Mus musculus OX=10090 GN=Slc4a4 PE=1 SV=2	2.10E+05	1.98E+05	1.87E+05	170948.17	1.65E+05	1.53E+05	0.820668103	0.013446187	0.3489800
Q6WVG3	BTB/POZ domain-containing protein KCTD12 OS=Mus musculus OX=10090 GN=Kctd12 PE=1 SV=1	7.53E+04	7.51E+04	6.97E+04	62316.50	5.65E+04	6.14E+04	0.818734966	0.006833248	0.3110762
O70172	Phosphatidylinositol 5-phosphate 4-kinase type-2 alpha OS=Mus musculus OX=10090 GN=Pip4k2a PE=1 SV=1	7.03E+04	7.40E+04	7.29E+04	60699.47	5.81E+04	5.89E+04	0.817496443	0.000598942	0.158849
Q8CI75	DIS3-like exonuclease 2 OS=Mus musculus OX=10090 GN=Dis3l2 PE=1 SV=1	5.73E+04	6.11E+04	5.45E+04	50058.60	5.04E+04	4.04E+04	0.81473906	0.048137751	0.4350027
Q9CXN7	Phenazine biosynthesis-like domain-containing protein 2 OS=Mus musculus OX=10090 GN=Pbld2 PE=1 SV=1	2.32E+04	2.34E+04	2.23E+04	17788.80	2.01E+04	1.83E+04	0.81464308	0.005262973	0.3029392
P57080	Ubiquitin carboxyl-terminal hydrolase 25 OS=Mus musculus OX=10090 GN=Usp25 PE=1 SV=2	2.30E+04	2.42E+04	2.51E+04	20624.44	1.98E+04	1.84E+04	0.813791115	0.007315058	0.3137448
Q3TCN2	Putative phospholipase B-like 2 OS=Mus musculus OX=10090 GN=Plbd2 PE=1 SV=2	3.09E+05	2.98E+05	2.80E+05	257746.06	2.28E+05	2.36E+05	0.813013199	0.010594197	0.3480419
P63054	Calmodulin regulator protein PCP4 OS=Mus musculus OX=10090 GN=Pcp4 PE=3 SV=2	8.44E+04	7.42E+04	7.68E+04	62966.03	6.59E+04	6.18E+04	0.810082212	0.010880922	0.3480419
Q8R016	Bleomycin hydrolase OS=Mus musculus OX=10090 GN=Blmh PE=1 SV=1	1.21E+06	1.41E+06	1.50E+06	1116466.90	1.09E+06	1.12E+06	0.806117908	0.035534799	0.4129479
P08207	Protein S100-A10 OS=Mus musculus OX=10090 GN=S100a10 PE=1 SV=2	2.57E+05	2.82E+05	2.50E+05	233017.16	2.08E+05	1.91E+05	0.801319773	0.027984608	0.3937240
Q8BMS9	Ras association domain-containing protein 2 OS=Mus musculus OX=10090 GN=Rassf2 PE=1 SV=1	6.08E+04	6.86E+04	6.48E+04	57331.57	4.87E+04	4.94E+04	0.800531008	0.022436272	0.3578990
P61793	Lysophosphatidic acid receptor 1 OS=Mus musculus OX=10090 GN=Lpar1 PE=1 SV=1	1.67E+04	1.75E+04	1.92E+04	13648.96	1.32E+04	1.58E+04	0.799573432	0.030457269	0.3966517
Q9WVH9	Fibulin-5 OS=Mus musculus OX=10090 GN=Fbln5 PE=1 SV=1	2.34E+05	2.28E+05	2.25E+05	199374.80	1.85E+05	1.64E+05	0.798642399	0.011597991	0.3480419
Q7TPD0	Integrator complex subunit 3 OS=Mus musculus OX=10090 GN=Ints3 PE=1 SV=2	1.67E+05	2.08E+05	1.84E+05	148689.12	1.37E+05	1.57E+05	0.793980115	0.04308604	0.4270203
Q8BGS2	BolA-like protein 2 OS=Mus musculus OX=10090 GN=Bola2 PE=1 SV=1	7.02E+04	7.54E+04	7.50E+04	57779.53	5.98E+04	5.74E+04	0.793367225	0.001184983	0.2477238
P58802	TBC1 domain family member 10A OS=Mus musculus OX=10090 GN=Tbc1d10a PE=1 SV=1	4.57E+04	4.99E+04	4.99E+04	42449.00	3.50E+04	3.80E+04	0.792999871	0.017823207	0.351664
Q8CIB6	Transmembrane protein 230 OS=Mus musculus OX=10090 GN=Tmem230 PE=1 SV=1	1.19E+05	1.02E+05	1.08E+05	89678.41	8.25E+04	8.91E+04	0.792698749	0.014317122	0.3489800
Q03173	Protein enabled homolog OS=Mus musculus OX=10090 GN=Enah PE=1 SV=2	2.49E+04	2.27E+04	2.49E+04	19574.34	2.09E+04	1.69E+04	0.791284296	0.022327598	0.3578990
Q7TPW1	Nexilin OS=Mus musculus OX=10090 GN=Nexn PE=1 SV=3	1.70E+05	1.83E+05	1.71E+05	146932.05	1.30E+05	1.36E+05	0.789176925	0.004426519	0.2962781
Q9Z2Y3	Homer protein homolog 1 OS=Mus musculus OX=10090 GN=Homer1 PE=1 SV=2	1.07E+05	9.36E+04	9.31E+04	77708.51	7.27E+04	8.04E+04	0.787277197	0.014016593	0.3489800
Q8CEE7	Retinol dehydrogenase 13 OS=Mus musculus OX=10090 GN=Rdh13 PE=1 SV=1	9.98E+03	8.55E+03	7.98E+03	6757.80	6.96E+03	7.06E+03	0.783624559	0.033679793	0.4078540
Q8R151	NFX1-type zinc finger-containing protein 1 OS=Mus musculus OX=10090 GN=Znfx1 PE=1 SV=3	2.91E+04	2.61E+04	3.08E+04	21484.43	2.33E+04	2.22E+04	0.779322457	0.013162914	0.3480419
Q8VEK2	Rhomboid domain-containing protein 2 OS=Mus musculus OX=10090 GN=Rhbdd2 PE=1 SV=1	8.19E+03	7.27E+03	7.88E+03	6808.77	5.56E+03	5.73E+03	0.775244525	0.021358578	0.3559661
Q9CX80	Cytoglobin OS=Mus musculus OX=10090 GN=Cygb PE=1 SV=1	9.17E+04	9.55E+04	1.04E+05	78983.26	7.49E+04	7.22E+04	0.775026209	0.006821465	0.3110762
Q6PIU9	Uncharacterized protein FLJ45252 homolog OS=Mus musculus OX=10090 PE=1 SV=2	2.81E+04	2.77E+04	2.89E+04	21704.88	2.17E+04	2.17E+04	0.768693632	4.7154E-05	0.0936477
P98200	Phospholipid-transporting ATPase IB OS=Mus musculus OX=10090 GN=Atp8a2 PE=1 SV=1	9.06E+04	8.24E+04	8.27E+04	66005.61	6.80E+04	6.25E+04	0.768623873	0.003266063	0.2962781
P98197	Probable phospholipid-transporting ATPase IH OS=Mus musculus OX=10090 GN=Atp11a PE=1 SV=1	7.30E+04	8.03E+04	7.27E+04	60676.68	5.43E+04	5.74E+04	0.762881163	0.004455748	0.2962781
P97770	THUMP domain-containing protein 3 OS=Mus musculus OX=10090 GN=Thumpd3 PE=1 SV=1	6.66E+04	7.60E+04	6.75E+04	60309.27	5.57E+04	4.42E+04	0.762400781	0.04186418	0.4270203
Q91YL3	Uridine-cytidine kinase-like 1 OS=Mus musculus OX=10090 GN=Uckl1 PE=1 SV=1	5.46E+03	5.06E+03	5.78E+03	4118.33	4.00E+03	4.30E+03	0.761971003	0.004533616	0.2962781
Q9R111	Guanine deaminase OS=Mus musculus OX=10090 GN=Gda PE=1 SV=1	1.44E+05	1.33E+05	1.32E+05	93429.06	1.13E+05	1.04E+05	0.758589092	0.00881984	0.3224334
Q4KMM3	Oxidation resistance protein 1 OS=Mus musculus OX=10090 GN=Oxr1 PE=1 SV=3	7.20E+05	6.49E+05	6.01E+05	518141.90	4.87E+05	4.82E+05	0.754934611	0.011344432	0.3480419
Q9D6J5	NADH dehydrogenase [ubiquinone] 1 beta subcomplex subunit 8, mitochondrial OS=Mus musculus OX=10090 GN=Ndufb8 PE=1 SV=1	1.57E+06	1.38E+06	1.23E+06	999462.60	1.11E+06	1.05E+06	0.754510168	0.028746503	0.3937279
P31428	Dipeptidase 1 OS=Mus musculus OX=10090 GN=Dpep1 PE=1 SV=2	1.22E+05	1.46E+05	1.44E+05	104995.97	1.01E+05	1.05E+05	0.752790919	0.01160568	0.3480419

Q8VCG4	Complement component C8 gamma chain OS=Mus musculus OX=10090 GN=C8g PE=1 SV=1	7.47E+04	6.89E+04	8.44E+04	48113.64	6.20E+04	6.09E+04	0.750246903	0.04037705	0.4270203
Q9D154	Leukocyte elastase inhibitor A OS=Mus musculus OX=10090 GN=Serpinb1a PE=1 SV=1	8.49E+05	6.90E+05	6.91E+05	580403.33	5.44E+05	5.43E+05	0.747968653	0.026138178	0.3876421
P70695	Fructose-1,6-bisphosphatase isozyme 2 OS=Mus musculus OX=10090 GN=Fbp2 PE=1 SV=2	3.02E+04	2.81E+04	2.89E+04	21461.96	1.91E+04	2.45E+04	0.746541962	0.01168346	0.3480419
Q61233	Plastin-2 OS=Mus musculus OX=10090 GN=Lcp1 PE=1 SV=4	2.95E+05	2.83E+05	2.34E+05	202237.25	2.03E+05	1.97E+05	0.743357051	0.020875515	0.3559661
B2RUR8	OTU domain-containing protein 7B OS=Mus musculus OX=10090 GN=Otud7b PE=1 SV=1	1.99E+05	1.91E+05	2.16E+05	155456.58	1.59E+05	1.35E+05	0.741858176	0.007598796	0.3168862
Q91WL8	WW domain-containing oxidoreductase OS=Mus musculus OX=10090 GN=Wwox PE=1 SV=1	2.28E+03	2.52E+03	2.15E+03	1966.84	1.81E+03	1.38E+03	0.740262539	0.043007278	0.4270203
P14220	Glycophorin-A OS=Mus musculus OX=10090 GN=Gypa PE=1 SV=2	4.52E+05	5.28E+05	4.02E+05	299810.58	3.66E+05	3.51E+05	0.735570079	0.043682911	0.4270203
Q00897	Alpha-1-antitrypsin 1-4 OS=Mus musculus OX=10090 GN=Serpina1d PE=1 SV=1	2.78E+05	3.34E+05	3.60E+05	208411.77	2.71E+05	2.27E+05	0.727049192	0.043631825	0.4270203
Q09M02	Cytosolic carboxypeptidase-like protein 5 OS=Mus musculus OX=10090 GN=Agbl5 PE=1 SV=2	1.46E+05	1.58E+05	1.38E+05	113299.09	1.10E+05	9.56E+04	0.72169751	0.006841864	0.3110762
Q5XPI3	E3 ubiquitin-protein ligase RNF123 OS=Mus musculus OX=10090 GN=Rnf123 PE=1 SV=1	1.22E+05	1.27E+05	1.16E+05	80282.42	1.09E+05	6.97E+04	0.708419662	0.043975445	0.4270203
Q08331	Calretinin OS=Mus musculus OX=10090 GN=Calb2 PE=1 SV=3	2.22E+05	1.96E+05	2.12E+05	147288.10	1.54E+05	1.43E+05	0.706363875	0.00169626	0.2929367
P17809	Solute carrier family 2, facilitated glucose transporter member 1 OS=Mus musculus OX=10090 GN=Slc2a1 PE=1 SV=4	2.92E+05	3.36E+05	3.02E+05	240738.08	2.11E+05	2.00E+05	0.700382113	0.007010904	0.3110762
Q01279	Epidermal growth factor receptor OS=Mus musculus OX=10090 GN=Egfr PE=1 SV=1	2.46E+05	2.47E+05	2.39E+05	126737.96	1.78E+05	2.05E+05	0.695736769	0.032012111	0.3998493
Q8C1A5	Thimet oligopeptidase OS=Mus musculus OX=10090 GN=Thop1 PE=1 SV=1	1.34E+06	1.40E+06	1.38E+06	962469.56	9.66E+05	8.56E+05	0.676686024	0.000397161	0.158849
B5X0G2	Major urinary protein 17 OS=Mus musculus OX=10090 GN=Mup17 PE=2 SV=2	2.44E+05	2.68E+05	2.91E+05	179204.10	1.80E+05	1.79E+05	0.671538044	0.002736734	0.2962781
P50114	Protein S100-B OS=Mus musculus OX=10090 GN=S100b PE=1 SV=2	1.95E+05	2.55E+05	2.35E+05	177929.80	1.50E+05	1.31E+05	0.670253137	0.028217111	0.3937240
Q9D1X0	Nucleolar protein 3 OS=Mus musculus OX=10090 GN=Nol3 PE=1 SV=1	1.83E+05	1.72E+05	1.87E+05	114302.59	1.06E+05	1.08E+05	0.60462554	0.000151497	0.100290
P12658	Calbindin OS=Mus musculus OX=10090 GN=Calb1 PE=1 SV=2	7.08E+04	4.74E+04	4.79E+04	32459.01	3.40E+04	3.17E+04	0.590972729	0.043214824	0.4270203
Q8VEL9	GTP-binding protein REM 2 OS=Mus musculus OX=10090 GN=Rem2 PE=1 SV=2	3.34E+05	2.52E+05	2.64E+05	164520.35	1.69E+05	1.61E+05	0.581548242	0.010120939	0.340681
Q8K182	Complement component C8 alpha chain OS=Mus musculus OX=10090 GN=C8a PE=1 SV=1	1.94E+04	1.98E+04	2.27E+04	8729.53	1.28E+04	1.32E+04	0.560234077	0.006847886	0.3110762
Q8BH35	Complement component C8 beta chain OS=Mus musculus OX=10090 GN=C8b PE=1 SV=1	3.00E+05	3.28E+05	3.12E+05	105803.76	2.04E+05	1.98E+05	0.539409138	0.011519568	0.3480419
Q00898	Alpha-1-antitrypsin 1-5 OS=Mus musculus OX=10090 GN=Serpina1e PE=1 SV=1	6.74E+06	7.31E+06	8.01E+06	2779717.18	3.60E+06	4.86E+06	0.50957497	0.007033367	0.3110762
P06683	Complement component C9 OS=Mus musculus OX=10090 GN=C9 PE=1 SV=2	1.62E+04	1.66E+04	1.94E+04	5047.90	1.02E+04	1.07E+04	0.496833335	0.013232151	0.3480419

Table S31. miR-183C target genes among upregulated protein in TG of miR-183C KO vs WT control mice.

Accession	Gene Name	Description	WT TG1	WT TG2	WT TG3	KO TG1	KO TG2	KO TG3	Fold Changes	pVal	q-value
Q3UHJ0	**Aak1**	AP2-associated protein kinase 1 OS=Mus musculus OX=10090 GN=Aak1 PE=1 SV=2	5.81E+05	5.76E+05	5.82E+05	6.10E+05	5.92E+05	6.11E+05	1.04	**1.66E-02**	0.3490
P41234	**Abca2**	ATP-binding cassette sub-family A member 2 OS=Mus musculus OX=10090 GN=Abca2 PE=1 SV=4	1.96E+05	2.13E+05	2.11E+05	2.62E+05	2.40E+05	2.30E+05	1.18	**2.69E-02**	0.3876
Q8CJG0	**Ago2**	Protein argonaute-2 OS=Mus musculus OX=10090 GN=Ago2 PE=1 SV=3	8.11E+04	8.50E+04	8.88E+04	9.79E+04	9.99E+04	9.06E+04	1.13	**3.61E-02**	0.4144
Q64362	**Aktip**	AKT-interacting protein OS=Mus musculus OX=10090 GN=Aktip PE=1 SV=1	1.57E+05	1.92E+05	2.13E+05	2.59E+05	2.46E+05	2.57E+05	1.35	**1.68E-02**	0.3490
Q9QWY8	**Asap1**	Arf-GAP with SH3 domain, ANK repeat and PH domain-containing protein 1 OS=Mus musculus OX=10090 GN=Asap1 PE=1 SV=2	1.81E+05	1.71E+05	1.87E+05	2.06E+05	1.90E+05	2.08E+05	1.12	**4.41E-02**	0.4270
O35855	**Bcat2**	Branched-chain-amino-acid aminotransferase, mitochondrial OS=Mus musculus OX=10090 GN=Bcat2 PE=1 SV=2	7.21E+05	7.14E+05	7.24E+05	7.58E+05	7.69E+05	7.52E+05	1.05	**2.39E-03**	0.2963
Q6PHS9	**Cacna2d2**	Voltage-dependent calcium channel subunit alpha-2/delta-2 OS=Mus musculus OX=10090 GN=Cacna2d2 PE=1 SV=1	4.13E+05	4.10E+05	4.35E+05	5.99E+05	5.72E+05	5.31E+05	1.35	**2.22E-03**	0.2963
Q80U49	**Cep170b**	Centrosomal protein of 170 kDa protein B OS=Mus musculus OX=10090 GN=Cep170b PE=1 SV=2	2.54E+04	3.74E+04	3.52E+04	5.66E+04	5.17E+04	5.14E+04	1.63	**7.07E-03**	0.3111
Q9D5R3	**Cep83**	Centrosomal protein of 83 kDa OS=Mus musculus OX=10090 GN=Cep83 PE=1 SV=2	8.85E+03	1.01E+04	9.92E+03	1.07E+04	1.05E+04	1.11E+04	1.12	**4.73E-02**	0.4329
P70232	**Chl1**	Neural cell adhesion molecule L1-like protein OS=Mus musculus OX=10090 GN=Chl1 PE=1 SV=2	1.08E+05	1.15E+05	1.07E+05	1.18E+05	1.17E+05	1.18E+05	1.07	**4.71E-02**	0.4329
Q9WUM4	**Coro1c**	Coronin-1C OS=Mus musculus OX=10090 GN=Coro1c PE=1 SV=2	1.25E+05	1.25E+05	1.24E+05	1.26E+05	1.27E+05	1.28E+05	1.02	**3.58E-02**	0.4130
Q8BTV2	**Cpsf7**	Cleavage and polyadenylation specificity factor subunit 7 OS=Mus musculus OX=10090 GN=Cpsf7 PE=1 SV=2	1.74E+05	1.74E+05	1.61E+05	1.87E+05	1.83E+05	1.87E+05	1.09	**2.34E-02**	0.3637
Q01147	**Creb1**	Cyclic AMP-responsive element-binding protein 1 OS=Mus musculus OX=10090 GN=Creb1 PE=1 SV=2	2.54E+05	2.54E+05	2.80E+05	3.06E+05	3.17E+05	2.86E+05	1.15	**3.24E-02**	0.4018
Q68ED7	**Crtc1**	CREB-regulated transcription coactivator 1 OS=Mus musculus OX=10090 GN=Crtc1 PE=1 SV=1	5.00E+04	5.26E+04	5.22E+04	6.16E+04	5.97E+04	5.63E+04	1.15	**1.19E-02**	0.3480
P10605	**Ctsb**	Cathepsin B OS=Mus musculus OX=10090 GN=Ctsb PE=1 SV=2	4.50E+05	4.30E+05	4.46E+05	4.71E+05	5.04E+05	5.40E+05	1.14	**3.96E-02**	0.4270
Q91WG7	**Dgkg**	Diacylglycerol kinase gamma OS=Mus musculus OX=10090 GN=Dgkg PE=1 SV=1	1.77E+05	1.80E+05	1.81E+05	1.88E+05	1.91E+05	1.97E+05	1.07	**1.00E-02**	0.3407
Q8BL66	**Eea1**	Early endosome antigen 1 OS=Mus musculus OX=10090 GN=Eea1 PE=1 SV=2	2.45E+05	2.77E+05	2.53E+05	3.09E+05	2.84E+05	3.04E+05	1.16	**3.04E-02**	0.3967
P70372	**Elavl1**	ELAV-like protein 1 OS=Mus musculus OX=10090 GN=Elavl1 PE=1 SV=2	4.80E+05	4.85E+05	5.00E+05	5.10E+05	5.18E+05	5.31E+05	1.06	**2.37E-02**	0.3646
Q8C3F2	**Fam120c**	Constitutive coactivator of PPAR-gamma-like protein 2 OS=Mus musculus OX=10090 GN=Fam120c PE=1 SV=3	3.88E+05	4.11E+05	4.27E+05	4.46E+05	4.49E+05	4.35E+05	1.08	**4.69E-02**	0.4329
Q9Z2G0	**Fem1b**	Protein fem-1 homolog B OS=Mus musculus OX=10090 GN=Fem1b PE=1 SV=1	5.63E+03	7.24E+03	5.96E+03	7.96E+03	7.53E+03	7.71E+03	1.23	**4.49E-02**	0.4294
Q6PB93	**Galnt2**	Polypeptide N-acetylgalactosaminyltransferase 2 OS=Mus musculus OX=10090 GN=Galnt2 PE=1 SV=1	2.57E+05	2.84E+05	2.88E+05	3.22E+05	3.14E+05	3.16E+05	1.15	**1.49E-02**	0.3490
Q8BUY8	**Gprasp2**	G-protein coupled receptor-associated sorting protein 2 OS=Mus musculus OX=10090 GN=Gprasp2 PE=1 SV=2	1.13E+05	1.09E+05	1.13E+05	1.29E+05	1.24E+05	1.22E+05	1.12	**4.58E-03**	0.2963
P97825	**Hn1**	Jupiter microtubule associated homolog 1 OS=Mus musculus OX=10090 GN=Jpt1 PE=1 SV=3	5.20E+04	4.92E+04	5.50E+04	6.14E+04	6.64E+04	7.25E+04	1.28	**1.53E-02**	0.3490
P49312	**Hnrnpa1**	Heterogeneous nuclear ribonucleoprotein A1 OS=Mus musculus OX=10090 GN=Hnrnpa1 PE=1 SV=2	1.18E+06	1.19E+06	1.21E+06	1.28E+06	1.30E+06	1.27E+06	1.08	**1.34E-03**	0.2586
Q8BUK6	**Hook3**	Protein Hook homolog 3 OS=Mus musculus OX=10090 GN=Hook3 PE=1 SV=2	5.54E+05	6.32E+05	6.20E+05	7.55E+05	7.35E+05	6.84E+05	1.20	**1.88E-02**	0.3522
P11627	**L1cam**	Neural cell adhesion molecule L1 OS=Mus musculus OX=10090 GN=L1cam PE=1 SV=1	1.88E+05	1.95E+05	2.01E+05	2.40E+05	2.27E+05	2.43E+05	1.22	**2.53E-03**	0.2963
P31938	**Map2k1**	Dual specificity mitogen-activated protein kinase kinase 1 OS=Mus musculus OX=10090 GN=Map2k1 PE=1 SV=2	2.94E+05	2.99E+05	2.98E+05	3.50E+05	3.35E+05	3.39E+05	1.15	**6.40E-04**	0.1588
P26645	**Marcks**	Myristoylated alanine-rich C-kinase substrate OS=Mus musculus OX=10090 GN=Marcks PE=1 SV=2	3.98E+05	3.92E+05	4.05E+05	4.57E+05	4.17E+05	4.49E+05	1.11	**2.68E-02**	0.3876
Q8CFI0	**Nedd4l**	E3 ubiquitin-protein ligase NEDD4-like OS=Mus musculus OX=10090 GN=Nedd4l PE=1 SV=2	1.89E+05	1.90E+05	1.87E+05	2.12E+05	2.02E+05	2.18E+05	1.12	**9.58E-03**	0.3281
Q3UHB1	**Nt5dc3**	5'-nucleotidase domain-containing protein 3 OS=Mus musculus OX=10090 GN=Nt5dc3 PE=1 SV=1	8.59E+05	9.15E+05	9.02E+05	1.02E+06	1.01E+06	9.38E+05	1.11	**3.53E-02**	0.4129
B9EJ86	**Osbpl8**	Oxysterol-binding protein-related protein 8 OS=Mus musculus OX=10090 GN=Osbpl8 PE=1 SV=1	1.11E+05	1.02E+05	1.08E+05	1.22E+05	1.15E+05	1.18E+05	1.11	**3.46E-02**	0.4129
Q9JK42	**Pdk2**	[Pyruvate dehydrogenase (acetyl-transferring)] kinase isozyme 2, mitochondrial OS=Mus musculus OX=10090 GN=Pdk2 PE=1 SV=2	4.43E+04	4.94E+04	5.14E+04	6.28E+04	6.00E+04	5.87E+04	1.25	**7.66E-03**	0.3169
Q9D0W5	**Ppil1**	Peptidyl-prolyl cis-trans isomerase-like 1 OS=Mus musculus OX=10090 GN=Ppil1 PE=1 SV=1	2.58E+05	2.63E+05	2.48E+05	2.84E+05	2.81E+05	2.69E+05	1.08	**2.87E-02**	0.3937
P63328	**Ppp3ca**	Serine/threonine-protein phosphatase 2B catalytic subunit alpha isoform OS=Mus musculus OX=10090 GN=Ppp3ca PE=1 SV=1	1.98E+05	2.02E+05	2.10E+05	2.32E+05	2.21E+05	2.17E+05	1.10	**2.69E-02**	0.3876
Q8C167	**Prepl**	Prolyl endopeptidase-like OS=Mus musculus OX=10090 GN=Prepl PE=1 SV=1	8.10E+05	8.14E+05	8.21E+05	8.79E+05	8.81E+05	8.41E+05	1.06	**1.87E-02**	0.3522
P16054	**Prkce**	Protein kinase C epsilon type OS=Mus musculus OX=10090 GN=Prkce PE=1 SV=1	2.92E+05	3.43E+05	3.33E+05	4.19E+05	3.86E+05	3.59E+05	1.20	**4.88E-02**	0.4388
B2RY56	**Rbm25**	RNA-binding protein 25 OS=Mus musculus OX=10090 GN=Rbm25 PE=1 SV=2	3.69E+05	4.16E+05	3.50E+05	4.54E+05	4.29E+05	4.36E+05	1.16	**4.39E-02**	0.4270
Q9R0P6	**Sec11a**	Signal peptidase complex catalytic subunit SEC11A OS=Mus musculus OX=10090 GN=Sec11a PE=1 SV=1	3.76E+04	3.77E+04	3.60E+04	4.12E+04	3.91E+04	4.09E+04	1.09	**1.73E-02**	0.3490
Q60520	**Sin3a**	Paired amphipathic helix protein Sin3a OS=Mus musculus OX=10090 GN=Sin3a PE=1 SV=3	2.44E+05	2.53E+05	2.56E+05	2.99E+05	2.84E+05	2.76E+05	1.14	**9.56E-03**	0.3281
Q8JZU2	**Slc25a1**	Tricarboxylate transport protein, mitochondrial OS=Mus musculus OX=10090 GN=Slc25a1 PE=1 SV=1	2.49E+05	3.05E+05	2.94E+05	4.78E+05	3.99E+05	4.21E+05	1.53	**6.90E-03**	0.3111
A2ASZ8	**Slc25a25**	Calcium-binding mitochondrial carrier protein SCaMC-2 OS=Mus musculus OX=10090 GN=Slc25a25 PE=1 SV=1	2.04E+05	1.98E+05	1.97E+05	2.25E+05	2.27E+05	2.12E+05	1.11	**1.62E-02**	0.3490
Q62093	**Srsf2**	Serine/arginine-rich splicing factor 2 OS=Mus musculus OX=10090 GN=Srsf2 PE=1 SV=4	6.68E+04	6.62E+04	6.93E+04	7.18E+04	7.03E+04	7.07E+04	1.05	**2.78E-02**	0.3937
Q8VBT0	**Tmx1**	Thioredoxin-related transmembrane protein 1 OS=Mus musculus OX=10090 GN=Tmx1 PE=1 SV=1	5.56E+04	5.50E+04	5.42E+04	6.35E+04	5.99E+04	6.41E+04	1.14	**5.65E-03**	0.3029
Q8C0L0	**Tmx4**	Thioredoxin-related transmembrane protein 4 OS=Mus musculus OX=10090 GN=Tmx4 PE=1 SV=2	2.29E+05	2.76E+05	2.58E+05	3.04E+05	2.92E+05	2.95E+05	1.17	**3.83E-02**	0.4247
Q5SSZ5	**Tns3**	Tensin-3 OS=Mus musculus OX=10090 GN=Tns3 PE=1 SV=1	1.10E+05	1.12E+05	1.20E+05	1.25E+05	1.30E+05	1.37E+05	1.15	**1.99E-02**	0.3522
Q9ESN6	**Trim2**	Tripartite motif-containing protein 2 OS=Mus musculus OX=10090 GN=Trim2 PE=1 SV=1	3.96E+05	3.75E+05	4.12E+05	4.30E+05	4.25E+05	4.24E+05	1.08	**4.25E-02**	0.4270
Q9QZE7	**Tsnax**	Translin-associated protein X OS=Mus musculus OX=10090 GN=Tsnax PE=1 SV=1	2.95E+05	3.00E+05	2.72E+05	3.41E+05	3.21E+05	3.17E+05	1.13	**3.25E-02**	0.4020

Table S32. Dysregulated genes at protein levels in the cornea of miR-183C KO vs WT control mice.

Accession	Description	WT Cornea1	WT Cornea2	WT Cornea3	KO Cornea1	KO Cornea2	KO Cornea3	Fold Changes	pVal	q-value
**Upregulated (48)**										
P01867	Ig gamma-2B chain C region OS=Mus musculus OX=10090 GN=Igh-3 PE=1 SV=3	2.71E+05	3.13E+05	2.37E+05	4.89E+05	5.57E+05	3.85E+05	1.74	**0.0203**	0.855
Q05816	Fatty acid-binding protein 5 OS=Mus musculus OX=10090 GN=Fabp5 PE=1 SV=3	1.77E+05	2.21E+05	1.46E+05	2.76E+05	3.01E+05	2.94E+05	1.60	**0.0090**	0.708
P01837	Immunoglobulin kappa constant OS=Mus musculus OX=10090 GN=Igkc PE=1 SV=2	1.39E+05	1.72E+05	1.20E+05	2.18E+05	2.78E+05	1.91E+05	1.59	**0.0458**	0.922
P01723	Ig lambda-1 chain V region OS=Mus musculus OX=10090 PE=1 SV=2	1.00E+05	1.32E+05	1.23E+05	1.48E+05	2.14E+05	1.77E+05	1.52	**0.0441**	0.922
P01633	Ig kappa chain V19-17 OS=Mus musculus OX=10090 GN=Igk-V19-17 PE=1 SV=1	9.28E+04	1.28E+05	9.64E+04	1.67E+05	1.79E+05	1.32E+05	1.51	**0.0401**	0.922
Q9CWM4	Prefoldin subunit 1 OS=Mus musculus OX=10090 GN=Pfdn1 PE=1 SV=1	8.61E+04	9.20E+04	8.43E+04	1.32E+05	9.81E+04	1.24E+05	1.35	**0.0428**	0.922
Q8C838	Trafficking regulator of GLUT4 1 OS=Mus musculus OX=10090 GN=Trarg1 PE=1 SV=1	3.52E+04	4.40E+04	4.54E+04	5.62E+04	5.32E+04	5.57E+04	1.33	**0.0153**	0.806
O55112	AF4/FMR2 family member 2 OS=Mus musculus OX=10090 GN=Aff2 PE=2 SV=2	3.32E+05	2.92E+05	3.92E+05	4.58E+05	4.12E+05	4.69E+05	1.32	**0.0337**	0.881
O89053	Coronin-1A OS=Mus musculus OX=10090 GN=Coro1a PE=1 SV=5	6.01E+04	7.12E+04	8.01E+04	9.15E+04	9.28E+04	9.05E+04	1.30	**0.0219**	0.879
Q9CWS0	N(G),N(G)-dimethylarginine dimethylaminohydrolase 1 OS=Mus musculus OX=10090 GN=Ddah1 PE=1 SV=3	1.27E+05	1.13E+05	1.22E+05	1.63E+05	1.46E+05	1.55E+05	1.28	**0.0052**	0.555
Q8K2Y9	Cerebral cavernous malformations protein 2 homolog OS=Mus musculus OX=10090 GN=Ccm2 PE=1 SV=1	3.27E+06	2.79E+06	2.91E+06	3.55E+06	4.09E+06	3.73E+06	1.27	**0.0201**	0.855
Q8BUV8	Protein GPR107 OS=Mus musculus OX=10090 GN=Gpr107 PE=1 SV=2	1.57E+05	1.51E+05	1.59E+05	1.84E+05	2.03E+05	2.01E+05	1.26	**0.0031**	0.483
Q64471	Glutathione S-transferase theta-1 OS=Mus musculus OX=10090 GN=Gstt1 PE=1 SV=4	2.33E+05	2.27E+05	2.14E+05	2.76E+05	2.75E+05	2.91E+05	1.25	**0.0017**	0.483
Q8BIQ5	Cleavage stimulation factor subunit 2 OS=Mus musculus OX=10090 GN=Cstf2 PE=1 SV=2	8.31E+04	8.02E+04	1.03E+05	1.13E+05	1.11E+05	1.08E+05	1.25	**0.0389**	0.922
Q9DBC7	cAMP-dependent protein kinase type I-alpha regulatory subunit OS=Mus musculus OX=10090 GN=Prkar1a PE=1 SV=3	2.05E+05	1.79E+05	2.21E+05	2.55E+05	2.35E+05	2.51E+05	1.22	**0.0286**	0.879
P01869	Ig gamma-1 chain C region, membrane-bound form OS=Mus musculus OX=10090 GN=Ighg1 PE=1 SV=2	1.38E+05	1.57E+05	1.36E+05	1.83E+05	1.63E+05	1.77E+05	1.21	**0.0280**	0.879
Q60952	Centrosome-associated protein CEP250 OS=Mus musculus OX=10090 GN=Cep250 PE=1 SV=4	1.04E+04	1.01E+04	1.04E+04	1.27E+04	1.19E+04	1.26E+04	1.20	**0.0018**	0.483
Q9Z0F7	Gamma-synuclein OS=Mus musculus OX=10090 GN=Sncg PE=1 SV=1	1.32E+05	1.40E+05	1.59E+05	1.74E+05	1.77E+05	1.69E+05	1.20	**0.0230**	0.879
Q60715	Prolyl 4-hydroxylase subunit alpha-1 OS=Mus musculus OX=10090 GN=P4ha1 PE=1 SV=2	1.15E+05	1.16E+05	1.30E+05	1.31E+05	1.45E+05	1.46E+05	1.17	**0.0445**	0.922
P19137	Laminin subunit alpha-1 OS=Mus musculus OX=10090 GN=Lama1 PE=1 SV=2	3.51E+05	3.32E+05	3.37E+05	3.76E+05	3.85E+05	4.17E+05	1.15	**0.0192**	0.855
Q9CQ65	S-methyl-5'-thioadenosine phosphorylase OS=Mus musculus OX=10090 GN=Mtap PE=1 SV=1	3.43E+05	3.14E+05	2.94E+05	3.83E+05	3.54E+05	3.62E+05	1.15	**0.0418**	0.922
O70378	ER membrane protein complex subunit 8 OS=Mus musculus OX=10090 GN=Emc8 PE=1 SV=1	7.32E+04	8.46E+04	7.23E+04	8.95E+04	8.96E+04	8.63E+04	1.15	**0.0454**	0.922
P13020	Gelsolin OS=Mus musculus OX=10090 GN=Gsn PE=1 SV=3	5.37E+05	5.11E+05	5.34E+05	5.59E+05	5.99E+05	6.42E+05	1.14	**0.0454**	0.922
Q8BFW7	Lipoma-preferred partner homolog OS=Mus musculus OX=10090 GN=Lpp PE=1 SV=1	1.51E+05	1.40E+05	1.42E+05	1.66E+05	1.57E+05	1.64E+05	1.13	**0.0114**	0.778
O35638	Cohesin subunit SA-2 OS=Mus musculus OX=10090 GN=Stag2 PE=1 SV=3	2.56E+04	2.54E+04	2.53E+04	2.78E+04	2.80E+04	2.99E+04	1.12	**0.0104**	0.778
Q4KMM3	Oxidation resistance protein 1 OS=Mus musculus OX=10090 GN=Oxr1 PE=1 SV=3	4.71E+05	4.51E+05	4.69E+05	5.49E+05	5.03E+05	5.04E+05	1.12	**0.0290**	0.879
Q80WR5	UPF0688 protein C1orf174 homolog OS=Mus musculus OX=10090 PE=1 SV=1	8.68E+04	7.76E+04	8.32E+04	8.97E+04	9.40E+04	9.32E+04	1.12	**0.0304**	0.879
Q06180	Tyrosine-protein phosphatase non-receptor type 2 OS=Mus musculus OX=10090 GN=Ptpn2 PE=1 SV=2	2.58E+04	2.51E+04	2.74E+04	2.91E+04	2.96E+04	2.86E+04	1.12	**0.0150**	0.806
Q6X893	Choline transporter-like protein 1 OS=Mus musculus OX=10090 GN=Slc44a1 PE=1 SV=3	4.29E+04	4.19E+04	4.57E+04	4.73E+04	4.74E+04	5.06E+04	1.11	**0.0338**	0.881
Q921Z5	Tumor necrosis factor alpha-induced protein 8 OS=Mus musculus OX=10090 GN=Tnfaip8 PE=1 SV=1	4.99E+04	5.30E+04	5.10E+04	5.62E+04	5.63E+04	5.86E+04	1.11	**0.0084**	0.678
P20152	Vimentin OS=Mus musculus OX=10090 GN=Vim PE=1 SV=3	6.42E+05	6.25E+05	6.20E+05	6.96E+05	6.89E+05	7.10E+05	1.11	**0.0016**	0.483
Q9DB34	Charged multivesicular body protein 2a OS=Mus musculus OX=10090 GN=Chmp2a PE=1 SV=1	2.02E+05	2.00E+05	2.09E+05	2.20E+05	2.19E+05	2.38E+05	1.11	**0.0298**	0.879
O88696	ATP-dependent Clp protease proteolytic subunit, mitochondrial OS=Mus musculus OX=10090 GN=Clpp PE=1 SV=1	1.18E+05	1.19E+05	1.25E+05	1.31E+05	1.29E+05	1.39E+05	1.10	**0.0305**	0.879
P19783	Cytochrome c oxidase subunit 4 isoform 1, mitochondrial OS=Mus musculus OX=10090 GN=Cox4i1 PE=1 SV=2	1.42E+05	1.53E+05	1.41E+05	1.58E+05	1.57E+05	1.63E+05	1.09	**0.0341**	0.881
Q8C460	ERI1 exoribonuclease 3 OS=Mus musculus OX=10090 GN=Eri3 PE=1 SV=1	1.69E+05	1.72E+05	1.73E+05	1.80E+05	1.91E+05	1.90E+05	1.09	**0.0116**	0.778
Q9DB29	Isoamyl acetate-hydrolyzing esterase 1 homolog OS=Mus musculus OX=10090 GN=Iah1 PE=1 SV=1	4.31E+05	4.00E+05	4.22E+05	4.56E+05	4.51E+05	4.63E+05	1.09	**0.0175**	0.833
O35309	N-myc-interactor OS=Mus musculus OX=10090 GN=Nmi PE=1 SV=1	2.10E+05	2.09E+05	2.10E+05	2.23E+05	2.32E+05	2.26E+05	1.08	**0.0030**	0.483
Q01768	Nucleoside diphosphate kinase B OS=Mus musculus OX=10090 GN=Nme2 PE=1 SV=1	8.88E+05	8.56E+05	8.04E+05	9.13E+05	9.21E+05	9.27E+05	1.08	**0.0466**	0.925
Q9CZW5	Mitochondrial import receptor subunit TOM70 OS=Mus musculus OX=10090 GN=Tomm70 PE=1 SV=2	2.61E+05	2.59E+05	2.76E+05	2.78E+05	2.97E+05	2.86E+05	1.08	**0.0485**	0.952
P62996	Transformer-2 protein homolog beta OS=Mus musculus OX=10090 GN=Tra2b PE=1 SV=1	2.70E+05	2.65E+05	2.68E+05	2.90E+05	2.89E+05	2.88E+05	1.08	**0.0002**	0.483
Q8BH97	Reticulocalbin-3 OS=Mus musculus OX=10090 GN=Rcn3 PE=1 SV=1	8.02E+04	7.89E+04	7.97E+04	8.22E+04	8.67E+04	8.85E+04	1.08	**0.0309**	0.881
Q60634	Flotillin-2 OS=Mus musculus OX=10090 GN=Flot2 PE=1 SV=2	4.58E+04	4.31E+04	4.54E+04	4.77E+04	4.75E+04	4.87E+04	1.07	**0.0263**	0.879
Q80T21	ADAMTS-like protein 4 OS=Mus musculus OX=10090 GN=Adamtsl4 PE=2 SV=1	4.32E+04	4.20E+04	4.34E+04	4.51E+04	4.64E+04	4.53E+04	1.06	**0.0111**	0.778
Q6PDY2	2-aminoethanethiol dioxygenase OS=Mus musculus OX=10090 GN=Ado PE=1 SV=2	3.54E+05	3.34E+05	3.49E+05	3.70E+05	3.64E+05	3.65E+05	1.06	**0.0303**	0.879
Q9QYS9	Protein quaking OS=Mus musculus OX=10090 GN=Qki PE=1 SV=1	4.51E+05	4.45E+05	4.33E+05	4.58E+05	4.76E+05	4.68E+05	1.05	**0.0342**	0.881
Q9CRB2	H/ACA ribonucleoprotein complex subunit 2 OS=Mus musculus OX=10090 GN=Nhp2 PE=1 SV=1	6.17E+04	6.24E+04	6.04E+04	6.51E+04	6.27E+04	6.55E+04	1.05	**0.0491**	0.957
Q9R069	Basal cell adhesion molecule OS=Mus musculus OX=10090 GN=Bcam PE=1 SV=1	2.64E+05	2.59E+05	2.56E+05	2.75E+05	2.68E+05	2.69E+05	1.04	**0.0247**	0.879
Q9DCC4	Pyrroline-5-carboxylate reductase 3 OS=Mus musculus OX=10090 GN=Pycr3 PE=1 SV=2	8.68E+04	8.86E+04	8.65E+04	9.02E+04	8.97E+04	9.15E+04	1.04	**0.0192**	0.855
**Downregulated (115)**										
Q8BWM0	Prostaglandin E synthase 2 OS=Mus musculus OX=10090 GN=Ptges2 PE=1 SV=3	3.16E+05	3.08E+05	3.06E+05	2.96E+05	2.97E+05	3.00E+05	0.96	**0.0171**	0.831
Q78IK4	MICOS complex subunit Mic27 OS=Mus musculus OX=10090 GN=Apool PE=1 SV=1	1.61E+05	1.64E+05	1.65E+05	1.58E+05	1.57E+05	1.52E+05	0.95	**0.0259**	0.879
F6ZDS4	Nucleoprotein TPR OS=Mus musculus OX=10090 GN=Tpr PE=1 SV=1	5.86E+05	5.98E+05	5.92E+05	5.54E+05	5.58E+05	5.79E+05	0.95	**0.0283**	0.879
O35841	Apoptosis inhibitor 5 OS=Mus musculus OX=10090 GN=Api5 PE=1 SV=2	1.93E+05	1.90E+05	1.97E+05	1.85E+05	1.84E+05	1.80E+05	0.95	**0.0154**	0.806
Q9D7X8	Gamma-glutamylcyclotransferase OS=Mus musculus OX=10090 GN=Ggct PE=1 SV=1	1.60E+05	1.68E+05	1.70E+05	1.56E+05	1.58E+05	1.56E+05	0.94	**0.0427**	0.922
Q99K48	Non-POU domain-containing octamer-binding protein OS=Mus musculus OX=10090 GN=Nono PE=1 SV=3	3.22E+05	3.27E+05	3.31E+05	3.10E+05	2.96E+05	3.17E+05	0.94	**0.0497**	0.958
Q3UA37	Glutamine-rich protein 1 OS=Mus musculus OX=10090 GN=Qrich1 PE=1 SV=1	1.96E+05	1.96E+05	2.03E+05	1.87E+05	1.89E+05	1.85E+05	0.94	**0.0114**	0.778
Q99JX7	Nuclear RNA export factor 1 OS=Mus musculus OX=10090 GN=Nxf1 PE=1 SV=3	1.01E+05	9.97E+04	1.03E+05	9.61E+04	9.18E+04	9.80E+04	0.94	**0.0425**	0.922
Q9Z2U1	Proteasome subunit alpha type-5 OS=Mus musculus OX=10090 GN=Psma5 PE=1 SV=1	8.78E+04	8.93E+04	8.91E+04	8.16E+04	8.63E+04	8.23E+04	0.94	**0.0251**	0.879
Q8R007	Nectin-4 OS=Mus musculus OX=10090 GN=Nectin4 PE=2 SV=1	8.86E+04	8.42E+04	8.85E+04	8.19E+04	8.09E+04	8.24E+04	0.94	**0.0230**	0.879
Q60902	Epidermal growth factor receptor substrate 15-like 1 OS=Mus musculus OX=10090 GN=Eps15l1 PE=1 SV=3	7.69E+04	7.66E+04	7.75E+04	7.07E+04	7.24E+04	7.31E+04	0.94	**0.0032**	0.483
Q80UM7	Mannosyl-oligosaccharide glucosidase OS=Mus musculus OX=10090 GN=Mogs PE=1 SV=1	6.15E+04	6.37E+04	6.16E+04	5.78E+04	5.90E+04	5.78E+04	0.93	**0.0078**	0.669
Q9CVD2	Ataxin-3 OS=Mus musculus OX=10090 GN=Atxn3 PE=1 SV=2	3.58E+04	3.43E+04	3.53E+04	3.26E+04	3.22E+04	3.37E+04	0.93	**0.0208**	0.861

Table S33. miR-183C target genes among upregulated proteins in the cornea of miR-183C KO vs WT control mice.

Accession	Gene Symnol	Description	WT Cornea1	WT Cornea2	WT Cornea3	KO Cornea1	KO Cornea2	KO Cornea3	Fold Changes	pVal	q-value
Q60952	Cep250	Centrosome-associated protein CEP250 OS=Mus musculus OX=10090 GN=Cep250 PE=1 SV=4	1.04E+04	1.01E+04	1.04E+04	1.27E+04	1.19E+04	1.26E+04	1.20	**0.0018**	0.483
Q9CWS0	Ddah1	N(G),N(G)-dimethylarginine dimethylaminohydrolase 1 OS=Mus musculus OX=10090 GN=Ddah1 PE=1 SV=3	1.27E+05	1.13E+05	1.22E+05	1.63E+05	1.46E+05	1.55E+05	1.28	**0.0052**	0.555
Q8BFW7	Lpp	Lipoma-preferred partner homolog OS=Mus musculus OX=10090 GN=Lpp PE=1 SV=1	1.51E+05	1.40E+05	1.42E+05	1.66E+05	1.57E+05	1.64E+05	1.13	**0.0114**	0.778
Q9DBC7	Prkar1a	cAMP-dependent protein kinase type I-alpha regulatory subunit OS=Mus musculus OX=10090 GN=Prkar1a PE=1 SV=3	2.05E+05	1.79E+05	2.21E+05	2.55E+05	2.35E+05	2.51E+05	1.22	**0.0286**	0.879
Q9QYS9	Qk	Protein quaking OS=Mus musculus OX=10090 GN=Qki PE=1 SV=1	4.51E+05	4.45E+05	4.33E+05	4.58E+05	4.76E+05	4.68E+05	1.05	**0.0342**	0.881
Q6X893	Slc44a1	Choline transporter-like protein 1 OS=Mus musculus OX=10090 GN=Slc44a1 PE=1 SV=3	4.29E+04	4.19E+04	4.57E+04	4.73E+04	4.74E+04	5.06E+04	1.11	**0.0338**	0.881
Q921Z5	Tnfaip8	Tumor necrosis factor alpha-induced protein 8 OS=Mus musculus OX=10090 GN=Tnfaip8 PE=1 SV=1	4.99E+04	5.30E+04	5.10E+04	5.62E+04	5.63E+04	5.86E+04	1.11	**0.0084**	0.678
